# Genome-wide identification of MAPK, MAPKK, and MAPKKK gene families and transcriptional profiling analysis during development and stress response in cucumber

**DOI:** 10.1186/s12864-015-1621-2

**Published:** 2015-05-15

**Authors:** Jie Wang, Changtian Pan, Yan Wang, Lei Ye, Jian Wu, Lifei Chen, Tao Zou, Gang Lu

**Affiliations:** Department of Horticulture, Zhejiang University, Hangzhou, 310058 China; Key Laboratory of Horticultural Plant Growth, Development and Quality Improvement, Ministry of Agriculture, Hangzhou, 310058 China

**Keywords:** *Cucumis sativus*, MAPKKK, MAPKK, MAPK, Abiotic stress, Biotic stress, Plant hormones

## Abstract

**Background:**

The mitogen-activated protein kinase (MAPK) cascade consists of three types of reversibly phosphorylated kinases, namely, MAPK, MAPK kinase (MAPKK/MEK), and MAPK kinase kinase (MAPKKK/MEKK), playing important roles in plant growth, development, and defense response. The MAPK cascade genes have been investigated in detail in model plants, including *Arabidopsis*, rice, and tomato, but poorly characterized in cucumber (*Cucumis sativus* L.), a major popular vegetable in Cucurbitaceae crops, which is highly susceptible to environmental stress and pathogen attack.

**Results:**

A genome-wide analysis revealed the presence of at least 14 MAPKs, 6 MAPKKs, and 59 MAPKKKs in the cucumber genome. Phylogenetic analyses classified all the CsMAPK and CsMAPKK genes into four groups, whereas the CsMAPKKK genes were grouped into the MEKK, RAF, and ZIK subfamilies. The expansion of these three gene families was mainly contributed by segmental duplication events. Furthermore, the ratios of non-synonymous substitution rates (Ka) and synonymous substitution rates (Ks) implied that the duplicated gene pairs had experienced strong purifying selection. Real-time PCR analysis demonstrated that some MAPK, MAPKK and MAPKKK genes are preferentially expressed in specific organs or tissues. Moreover, the expression levels of most of these genes significantly changed under heat, cold, drought, and *Pseudoperonospora cubensis* treatments. Exposure to abscisic acid and jasmonic acid markedly affected the expression levels of these genes, thereby implying that they may play important roles in the plant hormone network.

**Conclusion:**

A comprehensive genome-wide analysis of gene structure, chromosomal distribution, and evolutionary relationship of MAPK cascade genes in cucumber are present here. Further expression analysis revealed that these genes were involved in important signaling pathways for biotic and abiotic stress responses in cucumber, as well as the response to plant hormones. Our first systematic description of the MAPK, MAPKK, and MAPKKK families in cucumber will help to elucidate their biological roles in plant.

**Electronic supplementary material:**

The online version of this article (doi:10.1186/s12864-015-1621-2) contains supplementary material, which is available to authorized users.

## Background

Cucumber (*Cucumis sativus* L.) is one of the most economically important vegetable crops worldwide. Moreover, cucumber has been used a model system for studies on plant vascular biology and sex determination [1]. However, its growth and production are hindered by multiple abiotic and biotic stresses, such as inappropriate temperature [2], drought [3], and pathogens [4]. Therefore, the systematic identification and functional study of stress response and tolerance genes in cucumber are required to elucidate the molecular mechanisms of cucumber tolerance and susceptibility. A draft of the cucumber genome sequence has been reported [5], which conveniently allowed the comprehensive overview of several gene families at the genomic level [6–11].

To regulate plant development and deal with environmental stress, plants have acquired complex mechanisms during their evolution to sense and transmit environmental stimuli. The mitogen-activated protein kinase (MAPK) signaling cascade has emerged as a universal signal transduction module that connects diverse receptors/sensors to cellular and nuclear responses in eukaryotes [12]. MAPK signaling modules are evolutionarily conserved in eukaryotes, including yeasts, animals, and plants. The classical MAPK signaling cascade is minimally composed of three kinases, namely, MAPK, MAPK kinase (MAPKK), and MAPKK kinase (MAPKKK) [13]. These kinases operate as sequential signal transducers that channel, integrate, and amplify information from the cellular environment to transcriptional and metabolic response centers via phosphorylation. MAPKs are activated by MAPKKs via the phosphorylation of conserved threonine and tyrosine residues in the Thr-X-Tyr (T-X-Y) motif that is located in the activation loop (T-loop) between the catalytic subdomains VII and VIII. MAPKKs, in turn, are activated by MAPKKKs when the serine and serine/threonine residues in the S/TXXXXXS/T motif are phosphorylated [12].

In plants, MAPK cascades participate in numerous processes, including cell division [14], developmental programs [15], hormonal responses [12], and signaling responses to various forms of biotic and abiotic stress, such as pathogen infection [16], wounding [17], drought, salinity [18], UV irradiation [19], ozone [20], and reactive oxygen species (ROS) [16]. To date, several plant MAPK signaling cascades have been characterized in details. The first signaling module that was identified in plant is *Arabidopsis* MEKK1-MKK4/5-MPK3/6 cascade, which plays an vital role in plant innate immunity [21, 22]. Another module of *Arabidopsis*, MEKK1-MKK1/2-MPK4, was shown to positively regulate defense responses against necrotrophic fungi while negatively regulating defenses against biotrophic pathogens [23, 24]. The MEKK1-MKK2-MPK4 cascade is also activated during cold acclimation, and contributes to the acquisition of freezing tolerance [25]. In addition, the ANP3-MKK6-MPK4 cascade facilitates male-specific meiotic cytokinesis [26], whereas the YDA-MKK4/5-MPK3/6 module participates in regulating stomatal development in *Arabidopsis* [27]. In tobacco, the NPK1-MEK1-Ntf6 cascade regulates the resistance to the tobacco mosaic virus mediated the resistant protein N [28]. Moreover, the NPK1-NQK1/NtMEK1-NRK1 cascade positively regulates tobacco cytokinesis during meiosis and mitosis [29].

Recently, a large number of genes encoding proteins involved with the MAPK signaling cascade have been identified in various plants after the completion of their whole genome sequence. A total of 20 MAPK, 10 MAPKK, and 80 MAPKKK genes have been reported in the *Arabidopsis* genome [30-32], whereas the rice genome contains 17 MAPK, 8 MAPKK, and 75 MAPKKK genes [33–35]. Recent studies demonstrated that 19 MAPK, 9 MAPKK, and 74 MAPKKK genes can be found in maize [36–38], whereas 16 putative MAPK, 6 MAPKK, and 89 MAPKKK genes are in tomato [39, 40]. Despite being a model vegetable crop for functional genomics, to date, only few MAPK cascade genes were cloned and identified in cucumber, such as *CsMAPK1* [41], *CsTIPK* (*CsNMAPK*) [42–44], *CsMAP3Ka* [41] and *CsCRT1* [45]. However, no systematic and genome-wide investigations of the MAPK signaling cascade gene families have been reported in cucumber yet.

In the present study, 14 MAPK, 6 MAPKK, and 59 MAPKKK genes were identified in cucumber. They were classified into different subfamilies based on phylogenetic trees. The predicted gene structures, chromosomal locations, gene duplicates, and evolutionary mechanism were subsequently analyzed. Finally, their transcript profiles in different organs and in response to different stresses, as well as plant hormones, were analyzed by quantitative real-time reverse transcription PCR (qRT-PCR). Our data provide a basis for further research on the precise roles of the MAPK signaling cascade in cucumber development and in responses to abiotic and biotic stresses. Moreover, the findings will contribute to understanding the expansion and evolution of the MAPK, MAPKK, and MAPKKK gene families in plant.

## Results and discussion

### Identification of the MAPK, MAPKK, and MAPKKK families in cucumber

To identify MAPK, MAPKK and MAPKKK family genes, we conducted respective BLASTP searches against the cucumber protein database using query protein sequences including 143 MAPKs, 67 MAPKKs, and 534 MAPKKKs from seven plant species, which resulted in 602 hits. Meanwhile, a HMM search was also employed to identify all potential MAPK cascade sequences containing the serine/threonine-protein kinase-like domain (PF00069) in cucumber, which resulted in a total of 727 hits. The comparison of the sequence from BLAST and HMM hits were completed and the number of hits was reduced to 498 after reduce redundancies and alternative splices. Sequences that did not contain the known conserved motifs of the MAPK, MAPKK, or MAPKKK family proteins, respectively, were excluded from further analysis. After multiple steps of screening and validation of the conserved domains, we finally identified 14 CsMAPK, 6 CsMAPKK, and 59 CsMAPKKK genes, respectively. The sequence data of all above MAPK cascade genes were downloaded from the cucumber Genomics Database (http://www.icugi.org/cgi-bin/ICuGI/genome/index.cgi?organism=cucumber) (Additional file 1). Each gene was named according its homology with *Arabidopsis* MAPK, MAPKK, or MAPKKK proteins as suggested by the MAPK research community [30, 46] (Table 1-3). If two or more cucumber genes had the same homolog in *Arabidopsis*, they were distinguished by an extra number. For example, Csa1M024990, Csa5M002030, and Csa1M042720 are the homologs of AtMPK9, so they were named CsMPK9-1, CsMPK9-2, and CsMPK9-3, respectively. Given the alternative mRNA splicing in the cucumber MAPK and MAPKKK gene families (Additional file 2), the subsequent analysis of each gene was restricted to the longest encoding protein. Notablely, CsMAPK1 (CsMPK1) was previously identified to be involved in brassinosteroid-induced stress tolerance in cucumber [41] and CsMPK3 was characterized as CsNMAPK or CsTIPK [42–44]. Meanwhile, CsRAF1 and CsMEKK3 belonging to CsMAPKKK family was previously named as CsCTR1 [45] and CsMAP3Ka [41], respectively. In order to keep consistent with the new nomenclature of other MAPK cascade genes in cucumber, all these genes were renamed according to the orthologous sequence similarity with *A. thaliana* (Additional file 3).

**Table 1:** Characteristics of the MAPK genes in cucumber

Gene name	Deduced polypeptide			Type	Number of ESTs	GENE ID	Chromosome number	Location	Strand direction	Subcellular location
	Length	Molecular weight (kDa)	PI							
CsMPK1	560	64.3	6.19	TEY	14P	Csa2M361890.1	2	17433185–17436835	+	Plasma Membrane
CsMPK3	370	42.7	5.46	TEY	4C	Csa1M479630.1	1	17309272–17312689	+	Nuclear, Cytoplasm
CsMPK4-1	383	44.0	6.09	TEY	12P	Csa5M152810.1	5	4829455–4832852	+	Nuclear, Cytoplasm
CsMPK4-2	370	42.9	6.32	TEY	11P	Csa6M006730.1	6	532209–536191	-	Nuclear, Cytoplasm, Mitochondria
CsMPK6	405	46.3	5.48	TEY	10P	Csa6M365750.1	6	16570859–16583545	+	Cytoplasm
CsMPK7	368	42.4	6.70	TEY	4P	Csa4M045070.1	4	3555287–3556820	-	Nuclear, Mitochondria
CsMPK9-1	647	73.4	6.89	TDY	10P	Csa1M024990.1	1	2638051–2643197	-	Nuclear
CsMPK9-2	467	53.8	7.04	TDY	17P	**Csa5M002030.1**	5	81271–86265	-	Nuclear, Cytoplasm
CsMPK9-3	490	56.4	9.03	TDY	4P	**Csa1M042720.2**	1	4454081–4457590	-	Nuclear, Cytoplasm
CsMPK13	370	42.6	5.06	TEY	5P	Csa1M077220.1	1	7929029–7931830	+	Nuclear
CsMPK16	566	64.4	8.81	TDY	12P	Csa6M061230.1	6	4626299–4630958	+	Cytoplasm
CsMPK19	497	57.3	9.25	TDY	2P	**Csa4M082320.2**	4	5468502–5472301	-	Nuclear, Cytoplasm
CsMPK20-1	620	70.5	9.28	TDY	27P	Csa6M179480.1	6	11690390–11694812	-	Nuclear, Mitochondria
CsMPK20-2	606	68.4	9.19	TDY	3P	Csa6M423420.1	6	19655012–19658623	-	Nuclear

C and P: The ESTs which contain the whole and partial ORFs of relevant CsMAPKs, respectively. Gene ID highlighted in black contains more than one copies

To assess whether the genes identified in this study had existing support, we performed BLASTN searches against the cucumber expressed sequence tag (EST) and unigene database. The existence of all the predicted members of the MAPK and MAPKK families in *C. sativus* was supported by EST or unigene hits (Tables 1 and 2). Similarly, most of the MAPKKK sequences matched EST hits and cDNA sequences (Table 3) except 12 MAPKKKs representing 20.3 % where no such data were available.

**Table 2:** Characteristics of the MAPKK genes in cucumber

Gene name	Deduced polypeptide			Number of ESTs	Gene ID	Chromosome number	Location	Strand direction	Subcellular location
	Length	Molecular weight (kDa)	PI						
CsMKK2-1	355	39.9	5.21	12P	Csa1M589750.1	1	22388880–22391657	-	Cytoplasm
CsMKK2-2	353	39.4	5.33	4P	Csa2M000340.1	2	186835–189195	+	Cytoplasm
CsMKK3	518	57.8	5.67	1P	Csa3M839800.1	3	33665265–33669765	+	Cytoplasm
CsMKK4	368	41.4	8.91	4P	Csa3M651720.1	3	25634070–25635176	-	Nuclear
CsMKK6	358	40.3	6.42	1P	Csa2M000780.1	2	443066–445832	+	Cytoplasm, Nuclear
CsMKK9	320	35.9	8.25	4P	Csa1M042980.1	1	4620708–4621670	-	Nuclear

C and P: The ESTs which contain the whole and partial ORFs of relevant CsMAPKKs, respectively

**Table 3:** Characteristics of the MAPKKK genes in cucumber

Gene name	Deduced polypeptide			Number of ESTs	Gene ID	Chromosome number	Location	Strand direction	Subcellular location
	Length	Molecular weight (kDa)	PI						
CsMAPKKK1	678	74.9	5.62	1P	Csa2M021750.1	2	2688606–2693210	+	Nuclear
CsMAPKKK3	632	67.3	9.14	9P	**Csa6M483320.1**	6	22175469–22179587	-	Nuclear
CsMAPKKK4-1	896	96.8	9.47	5P	Csa3M182770.1	3	12717187–12722466	-	Nuclear
CsMAPKKK4-2	889	96.0	9.38	0	Csa5M166980.1	5	6401871–6406567	+	Nuclear
CsMAPKKK5-1	769	83.7	9.22	2P	Csa6M490220.1	6	23292023–23297308	+	Nuclear
CsMAPKKK5-2	704	77.3	9.07	0	Csa2M360650.1	2	17054822–17058775	-	Nuclear
CsMAPKKK8	566	62.8	5.66	1P	Csa5M385380.1	5	14250357–14255268	-	Nuclear
CsMAPKKK12	636	70.3	6.43	1P	Csa6M425140.1	6	19885541–19890321	-	Nuclear
CsMAPKKK13	491	54.4	5.15	1P	Csa1M532310.1	1	18801570–18803045	+	Chloroplast
CsMAPKKK15	402	44.7	5.38	2P	Csa6M513560.1	6	26600235–26601519	+	Cytoplasm, Nuclear, Chloroplast
CsMAPKKK17-1	435	49.1	5.80	15P	Csa2M416770.1	2	21586777–21588084	+	Cytoplasm, Nuclear
CsMAPKKK17-2	372	41.9	4.82	0	Csa7M043040.1	7	2351691–2352809	+	Chloroplast
CsMAPKKK20	336	37.4	9.18	0	Csa7M430790.1	7	16849784–16851015	-	Cytoplasm, Mitochondria
CsMAPKKK21-1	350	38.9	4.99	17P	Csa6M490950.1	6	23482398–23483450	+	Cytoplasm
CsMAPKKK21-2	345	37.9	4.96	0	Csa2M278170.1	2	13289977–13291014	-	Chloroplast
CsMAPKKK21-3	331	38.1	6.98	0	Csa7M378450.1	7	13904002–13904997	+	Cytoplasm, Nuclear
CsMAPKKK21-4	353	40.5	6.24	0	Csa7M407720.1	7	15756947–15758008	+	Cytoplasm, Nuclear
CsMAPKKK21-5	330	37.8	8.87	1P	Csa3M829110.1	3	33117599–33118591	+	Cytoplasm, Nuclear
CsRAF1-1	852	94.5	5.77	0	Csa6M450400.1	6	21398481–21404987	+	Cytoplasm, Nuclear
CsRAF1-2	860	95.0	5.49	4P	Csa3M749850.1	3	29127779–29136084	+	Nuclear
CsRAF2	946	104.2	5.66	12P	Csa1M574260.1	1	21515696–21525432	-	Cytoplasm, Nuclear
CsRAF3	966	106.5	5.15	7P	Csa4M646020.1	4	21910209–21918810	-	Cytoplasm, Nuclear
CsRAF4	1011	110.3	5.51	5P	**Csa1M042730.1**	1	4465512–4476571	+	Nuclear
CsRAF6	902	100.0	6.56	2P	Csa3M892210.1	3	38100218–38108126	-	Chloroplast, Nuclear
CsRAF10	686	75.7	6.17	5P	Csa6M330990.1	6	15249399–15253602	-	Cytoplasm, Nuclear
CsRAF15	799	89.9	6.00	22P	Csa6M154510.1	6	10910852–10919554	-	Nuclear
CsRAF16	1162	129.7	5.40	1P	Csa3M133150.1	3	8680433–8685859	-	Nuclear
CsRAF18	1207	133.9	5.33	8P	Csa6M136540.1	6	9552953–9558532	-	Nuclear
CsRAF19–1	363	41.5	9.14	0	Csa6M511830.1	6	26450395–26451769	-	Nuclear
CsRAF19-2	361	41.2	8.99	5P	Csa1M046040.1	1	5272153–5274259	+	Plasma Membrane
CsRAF22	413	46.7	6.90	52C	Csa2M070870.1	2	5495966–5503657	-	Cytoplasm
CsRAF24	1291	142.5	5.17	44P	Csa1M057040.1	1	6296180–6304520	+	Nuclear
CsRAF25	473	53.6	9.05	3P	Csa7M051390.1	7	3242880–3247120	-	Nuclear, Mitochondria
CsRAF27	458	51.7	6.61	1P	Csa1M467120.1	1	16725450–16729393	-	Cytoplasm, Nuclear
CsRAF29	542	61.8	6.57	23P	Csa3M002480.1	3	321724–328150	-	Nuclear
CsRAF30-1	536	60.1	5.85	11	Csa7M017160.1	7	990917–998921	-	Nuclear
CsRAF30-2	555	62.5	5.46	8P	Csa6M058190.1	6	4524498–4538278	+	Cytoplasm
CsRAF31	373	42.0	9.31	1P	Csa3M728150.1	3	27247215–27250387	-	Cytoplasm, Nuclear
CsRAF34	353	39.8	7.16	0	Csa1M003510.1	1	589408–591750	+	Cytoplasm
CsRAF35	1192	133.9	5.78	5P	Csa2M049880.1	2	4087262–4098054	+	Nuclear
CsRAF36-1	492	55.9	9.28	2P	Csa6M517390.1	6	27240673–27243011	-	Mitochondria
CsRAF36-2	476	54.3	9.44	2P	Csa1M074900.1	1	7637332–7639059	-	Mitochondria
CsRAF37	374	42.6	5.17	2P	Csa6M502000.1	6	25234973–25238101	+	Cytoplasm, Nuclear, Plasma membrane
CsRAF38	385	42.3	7.06	1P	Csa3M836460.1	3	33384154–33387500	-	Cytoplasm, Nuclear
CsRAF39-1	398	44.4	8.50	9P	Csa7M387170.1	7	14180645–14183905	-	Cytoplasm, Nuclear, Chloroplast
CsRAF39-2	387	43.1	8.37	25P	**Csa3M146410.1**	3	9774770–9777297	-	Cytoplasm
CsRAF41-1	352	39.5	8.73	9C	**Csa3M840390.1**	3	33755408–33757740	+	Cytoplasm
CsRAF41-2	353	39.3	6.61	2P	Csa5M523010.1	5	18399026–18401943	-	Cytoplasm
CsRAF47	882	101.2	9.38	31P	**Csa6M520410.1**	6	27781623–27798089	+	Nuclear
CsZIK1	598	69.3	5.51	0	Csa4M332110.1	4	13437265–13441770	-	Nuclear
CsZIK2	645	72.9	6.52	2P	Csa6M212860.1	6	12899750–12903082	+	Nuclear
CsZIK4-1	732	83.6	5.34	12P	Csa2M012110.1	2	2162909–2166218	+	Nuclear
CsZIK4-2	740	84.2	4.88	3P	Csa7M234730.1	7	8351879–8354665	-	Nuclear
CsZIK4-3	610	70.1	5.65	0	Csa1M695390.1	1	27876401–27879136	+	Nuclear
CsZIK5	733	82.3	5.41	1P	Csa3M119370.1	3	6656546–6660203	-	Cytoplasm, Nuclear
CsZIK6	691	76.5	5.18	17P	Csa5M148620.1	5	4317304–4320333	-	Cytoplasm, Nuclear, Chloroplast
CsZIK8-1	300	34.2	5.84	13C	Csa3M062560.1	3	3573880–3576131	+	Nuclear
CsZIK8-2	376	42.5	5.31	3P	Csa6M110320.1	6	7511957–7515007	-	Nuclear
CsZIK11	601	68.0	4.91	2P	Csa1M046910.1	1	5508743–5512465	-	Nuclear

C and P: The ESTs which contain the whole and partial ORFs of relevant CsMAPKKKs, respectively. Gene ID highlighted in black contains more than one copies

The 14 CsMAPK genes were randomly distributed on 6 cucumber chromosomes, except chromosome 3 (Table 1). These CsMAPK genes were predicted to encoding 368 to 647 amino acids in length, with putative molecular weights (Mw) ranging from 42.4 to 73.4 kDa and theoretical isoelectric points (pIs) ranging from 5.06 to 9.28. The subcellular localization was predicated and the CsMAPK were located in the nucleus and cytoplasm, except CsMPK1, where it was present in plasma membrane (Table 1). Meanwhile, the 6 CsMAPKK predicted proteins contained 320–518 amino acids, with the Mws ranging from 35.9 to 57.8 kDa and pIs ranging from 5.21 to 8.91. They were predicted to be localized in the cytoplasm and nucleus (Table 2). All the 59 CsMAPKKK genes were randomly distributed on all the cucumber chromosomes. The polypeptide lengths of these CsMAPKKK genes ranged from 300 to 1291 aa with Mws ranging from 34.2 to 142.5 kDa. Similarly, 54 out of the 59 CsMAPKKK proteins were located in the cytoplasm or nucleus. The remaining proteins were present in plasma membrane, mitochondria, or chloroplast (Table 3).

Sequence similarity analysis of MAPK cascade genes between *Arabidopsis* and cucumber was conducted using predicted protein sequences (Additional file 4). The amino acid sequences of the homologous MAPK gene pairs in these two species showed high similarity (>70 %) with each other, whereas, the protein sequence similarities between gene pairs ranged from 62.9 % to 83.1 % in MAPKK family. However, most of the homologous MAPKKK gene pairs showed lower similarities compared to those of MAPK and MAPKK family genes. Given that the number of MAPK and MAPKK members are much less than that of MAPKKKs [12, 32], it is suggested that MAPK and MAPKK genes tend to be more conserved in evolution than MAPKKKs in plant.

### Phylogenetic relationship and conserved domain analysis

**Fig. 1:**
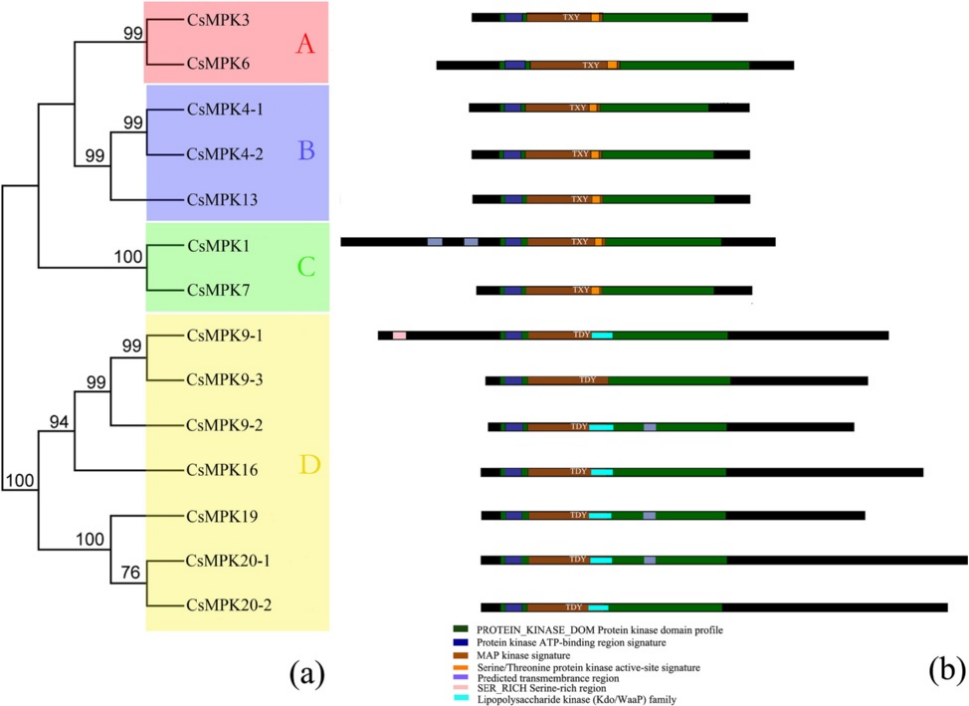
Phylogenetic analysis and domain organization of cucumber MAPKs. **a** The unrooted phylogenetic tree was generated based on the amino acid sequences by the NJ method using MEGA 5. Bootstrap supports from 1000 replicates are indicated at each branch. The members of each subfamily are indicated with the same color. **b** Domain organization was analyzed by scanning the protein sequences for the presence of known motifs and domains with PlantsP. Different subgroups of CsMAPKs are represented by the capital letter A-D

**Fig. 2:**
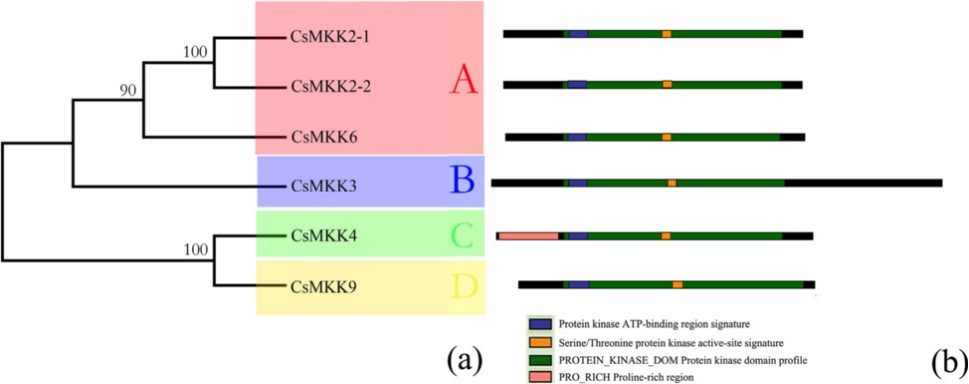
Phylogenetic analysis (**a**) and domain organization (**b**) of cucumber MAPKKs. Different subgroups of CsMAPKKs are represented by the capital letter A-D. For other details, see Fig. 1

To further characterize the MAPK cascade genes, unrooted phylogenetic trees were produced by aligning the full-length protein sequences of all 14 CsMAPKs, 6 CsMAPKKs, and 59 CsMAPKKKs using the neighbor-joining (NJ) method. Plant MAPK genes have diverged into four major subfamilies (A, B, C, and D) based on their phosphorylation motifs and the phylogenetic relationships of their amino acid sequences [30]. Similarly, all the CsMAPKs were also classified into groups A, B, C and D (Fig. 1), furthermore, all the CsMAPKs of groups A, B, and C harbor the TEY (Thr-Glu-Tyr) motif at the phosphorylation site, whereas the members of group D contain a TDY (Thr-Asp-Tyr) motif and form a more distant clade, which is consistent with those in *Arabidopsis* and rice [47]. Moreover, those CsMAPKs belonging to group D have an extended C-terminal region compared with the other three groups (Fig. 1), which is also present in *Arabidopsis* and *B, distachyon* [30, 48]. Likewise, CsMAPKKs were divided into four groups, namely, the A, B, C, and D subfamilies, which are consistent with the MAPKKs in *Arabidopsis*, rice, *B. distachyon* and canola [47–49] (Fig. 2a). All the CsMAPKKs contained a kinase domain, adenosinetriphosphate (ATP) binding site, and serine/threonine protein kinase active site. In particular, a proline-rich region and a long C-terminal region were found in the members of group B, such as CsMKK3 (Fig. 2b).

**Fig. 3:**
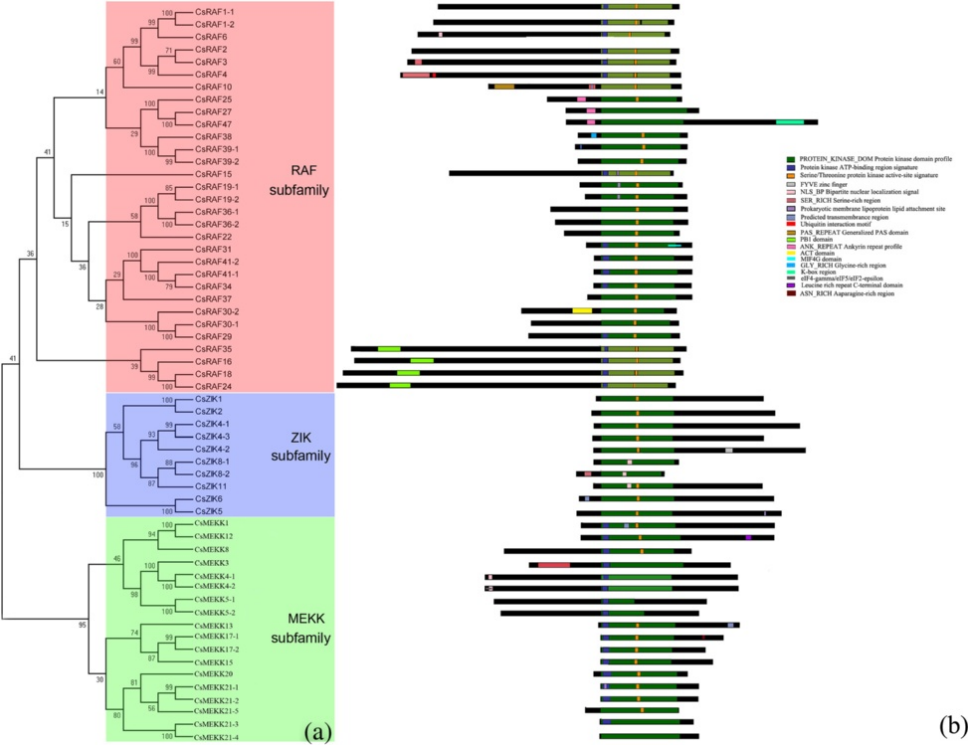
Phylogenetic analysis (**a**) and domain organization (**b**) of cucumber MAPKKKs. For other details, see Fig. 1

The MAPKKK gene family in plants is usually divided into three categories, namely MEKK, Raf, and ZIK subfamily [50]. Similarly, the CsMAPKKK genes in cucumber were clustered into three groups (18 MEKKs, 31 RAFs, and 10 ZIKs) (Fig. 3a) corresponding to other plants [35, 38, 40, 51, 52]. All the CsMAPKKK proteins have a kinase domain, and most of them have a serine/threonine protein kinase active site. In the cucumber RAF subfamily, most of the proteins have a long N-terminal regulatory domain and C-terminal kinase domain. By contrast, majority of the members in the ZIK subfamily have an N-terminal kinase domain. However, members of the MEKK subfamily have a less conserved protein structure, whose kinase domain is located either at the C- or N-terminal or in the central part of the protein. A NLS-BP region functioned as a bipartite nuclear localization signal, and was found to be distributed among the members of all the three subfamilies. However, an ubiquitin interaction motif and ACT domain were only present in CsRAF4 and CsRAF37, respectively (Fig. 3b), which are involved in the regulation of a wide range of metabolic enzymes by responding to amino acid concentrations. Interestingly, all the results were consistent with the previous findings in *Arabidopsis*, rice, and tomato [35, 40].

### Multiple sequence alignment and motif analysis

**Fig. 4:**
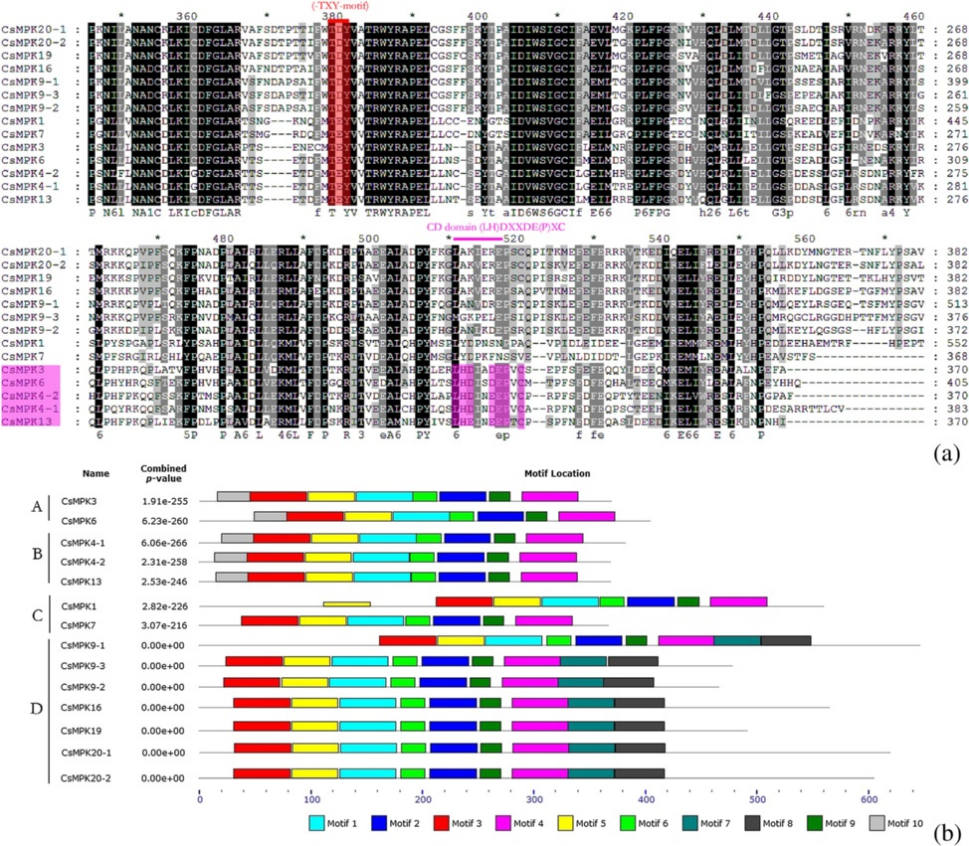
Sequence alignment and motif analysis of CsMAPKs. **a** Multiple sequence alignment analysis of the peptides of MAPK proteins in cucumber. The highlighted part shows the conserved signature motif obtained with the ClustalX program. **b** Schematic diagram of amino acid motifs of CsMAPKs. Motif analysis was analyzed by MEME program online. Different colors of the boxes represent different motifs in the corresponding position of each CsMAPK proteins. Different subgroups of CsMAPKs are represented by the capital letter A-D. The detailed information of 10 motifs was illustrated in Additional file 6

Multiple sequence alignment of the predicted amino acid residues was conducted on the CsMAPK cascade gene families. Data showed that all the CsMAPKs contain a classical TXY motif (Fig. 4a) which is located in the activation loop between the subdomains VII and VIII of MAPKs [30]. Meanwhile, the sequences in the T-loop motif were highly conserved in *Arabidopsis*, rice and cucumber (Additional file 5). Moreover, MAPKs may have a CD domain which is defined as (LH) DXXDE (P) X and functions as a docking site for MAPKKs. The two adjacent acidic residues D (aspartate) and E (glutamate) have been shown to play critical roles in interacting with a cluster of the amino acids K (lysine) and R (arginine) in MAPKKs [53]. In the present study, five MAPK genes from groups A and B (CsMPK3, CsMPK4-1, CsMPK4-2, CsMPK6, and CsMPK13) were found to possess a CD domain or modified CD domain in their C-terminal region, whereas groups C and D did not contain such a CD domain (Fig. 4a). This result was consistent with previous findings from other species, such as *B. distachyon* [48]. However, in *B. napus L.*, the CD domain was also found to exist in group C [49]. Meanwhile, no CD domain appeared in group D in cucumber or any other species [48, 49, 53]. The conserved motifs of the 14 CsMAPK proteins were also analyzed with the online tool MEME. Thus a schematic of the motifs is presented in Fig. 4b and Additional file 6. Seven out of 10 motifs, except 7, 8, and 10, were conserved in all the CsMAPK proteins. All the members identified in the same subfamily shared similar conserved motifs. For instance, besides all the conserved motifs, MAPK proteins in groups A and B had specific motif 10 at the N-terminal region, whereas those in group D contained motifs 8 and 9 at the C-terminal region (Fig. 4b and Additional file 6). In addition, the annotated motifs from MEME were analyzed. The data revealed that eight out of 10 motifs (1, 2, 3, 4, 5, 6, 7, and 9) corresponded with 11 subdomains (I-XI) of the kinase domains of MAPKs [30].

**Fig. 5:**
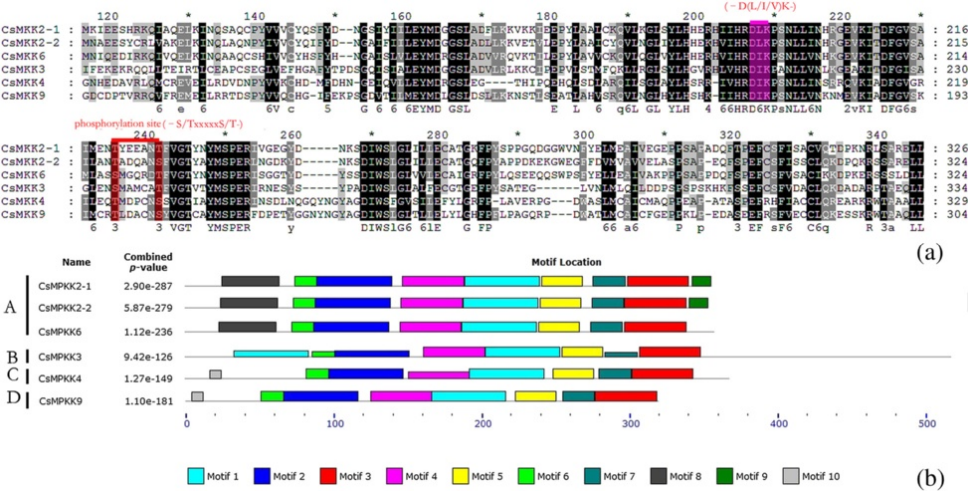
Sequence alignment and motif analysis of CsMAPKKs. Different subgroups of CsMAPKKs are represented by the capital letter A-D. The detailed information of 10 motifs was illustrated in Additional file 7. For other details, see Fig. 4

Multiple alignment of the CsMAPKKs showed that each contained the conserved lysine (K) and aspartate (D) residues within the active site motif D (L/I/V) K, as well as a highly conserved phosphorylation target site within the activation loop, which has a consensus sequence S/T-x5-S/T (Fig. 5a), as previously identified in *Arabidopsis* and some other plant MKK proteins [30, 48, 49]. Comparison analysis between the protein sequences of *Arabidopsis*, rice and cucumber uncovered that the D (L/I/V) K motif was highly conserved among these three species (Additional file 5). Moreover, the motif S/T-x5-S/T was conserved in subgroup A, B and C, but showed different degrees of divergences in the S/T site for four members in the D subgroup (*AtMKK10*, *OsMKK10-1*, *OsMKK10-2* and *OsMKK10-3*). The similar result was also found in *B. distachyon* [48]. More interestingly, it seemed that all the detected divergences were found in *AtMKK10* and its orthologs. Since there were no orthologs of *AtMKK10* in cucumber, this observation was not present. Further studies are needed to elucidate whether *AtMKK10* and its orthologs will display new functions compared to other MKKs. The schematic overview of identified motifs of CsMAPKKs from MEME analysis revealed that 7 motifs were conserved in all the cucumber MAPKK proteins, whereas the motif 8, 9, and 10 were group-specific. Similar to the CsMAPKs, the members in the same subfamilies contained almost all the common motifs. Group A had motif 8 in the N-terminal and motif 9 in the C-terminal, whereas groups C and D (BnaMKK9) had an extra motif 10 in the N-terminal sequence (Fig. 5b). We also observed that CsMKK3 had a long C-terminal region; similar findings were previously found in BnaMKK3 and BdMKK3 [49]. The motifs annotation revealed that motif 1 not only had the active-site signature IiHrDLKpsNLLV of serine/threonine protein kinases, but also contained the phosphorylation target site S/TXXXXS/T and signature VGTxxYMSPER, which was conserved in the catalytic domain [30]. However, motif 6 contained the protein kinase ATP-binding signature that required a glycine-rich loop (GxGxxG) for ATP binding (Additional file 7).

**Fig. 6:**
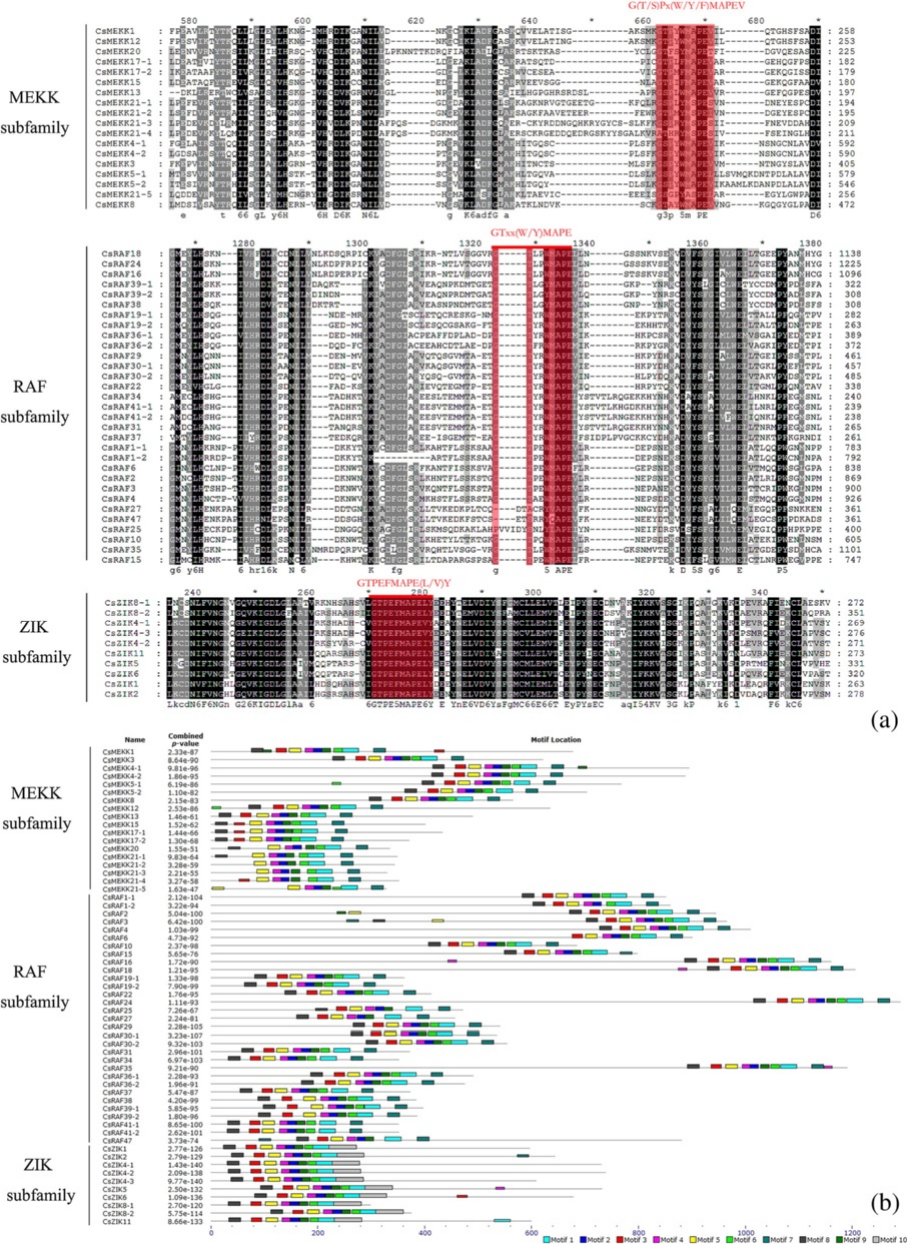
Sequence alignment and motif analysis of CsMAPKKKs. The detailed information of 10 motifs was illustrated in Additional file 8. For other details, see Fig. 4

As reported in *Arabidopsis* and other species MAPKKKs, the MEKK subfamily in cucumber includes a conserved signature G (T/S) Px (W/Y/F) MAPEV. The Raf-like subfamily in cucumber has the GTxx (W/Y) MAPE signature, whereas the ZIK subfamily has the GTPEFMAPE (L/V) Y signature [32, 35] (Fig. 6a). Multiple sequence alignments of MEKK, RAF and ZIK subfamilies from *Arabidopsis*, rice and cucumber showed that most of MAPKKKs have the conserved motif (Additional file 5). However, examinations of orthologous genes between *Arabidopsis* and cucumber showed that some variations existed in the conserved signature of some MAPKKKs. For example, the valine in the typical G (T/S) Fx (W/Y/F) MAPEV motif of MEKK subfamily is replaced by threonine and cysteine in *CsMAPKKK20* and *AtMAPKKK20*, respectively. Interestingly, similar observations were also reported in maize [38] and canola [51]. Motif annotation showed that motif 2 contained a protein kinase ATP-binding site, whereas motif 8 contained the serine/threonine protein kinase active site. In addition, motif 9 had a tyrosine kinase phosphorylation site (Additional file 8). Motif analysis revealed that nine out of 10 motifs (motifs 1–9) were conserved across all the subfamilies, whereas motif 10 was ZIK subfamily-specific (Fig. 6b). The ZIK proteins are known as WNK (without lysine) that have not been previously demonstrated to phosphorylate MAPKKs in plants. The ZIKs have been reported to be involved in internal rhythm. The ZIK protein WNK1 (At3g04910) in *Arabidopsis* was involved in the control of circadian rhythms [54]. The WNK2/5/8 proteins are found to regulate the flowering time in *Arabidopsis* by modulating the photoperiod pathway [55]. Recently, rice WNK1 was reported to be involved in internal circadian rhythm, and also differentially responded to various forms of abiotic stress [56]. However, the existence of such roles for CsZIK proteins in cucumber and specific motif 10 in CsZIKs associated with their specific roles have yet to be studied.

### Analyses of gene structures and promoter regions

**Fig. 7:**
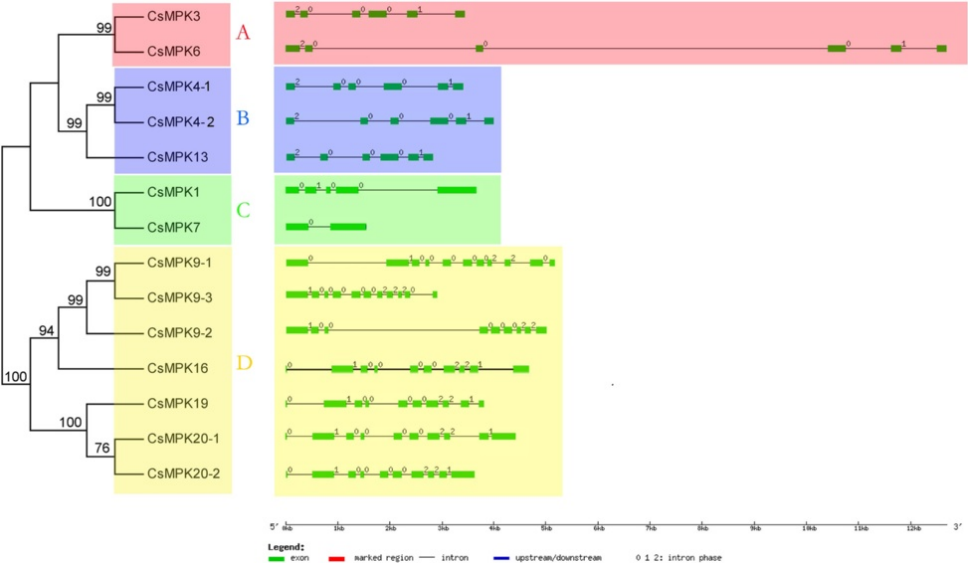
Phylogenetic analysis and gene structure of CsMAPKs in cucumber. Right part illustrates the intron/exon configurations of the corresponding CsMAPK genes. The green boxes indicate the exons, and lines indicate the introns. Gene structures of CsMAPKs in different subgroups are shaded by different colors. Different subgroups of CsMAPKs are represented by the capital letter A-D

**Fig. 8:**
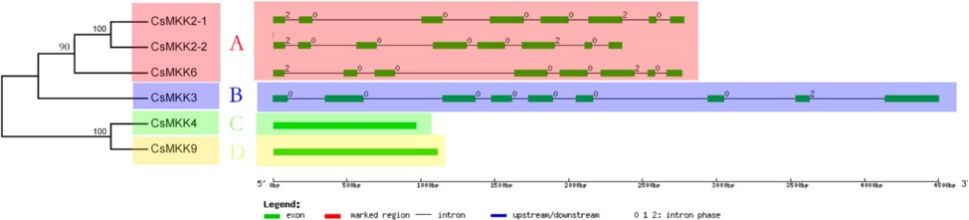
Phylogenetic analysis and gene structure of CsMAPKKs in cucumber. Different subgroups of CsMAPKKs are represented by the capital letter A-D

**Fig. 9:**
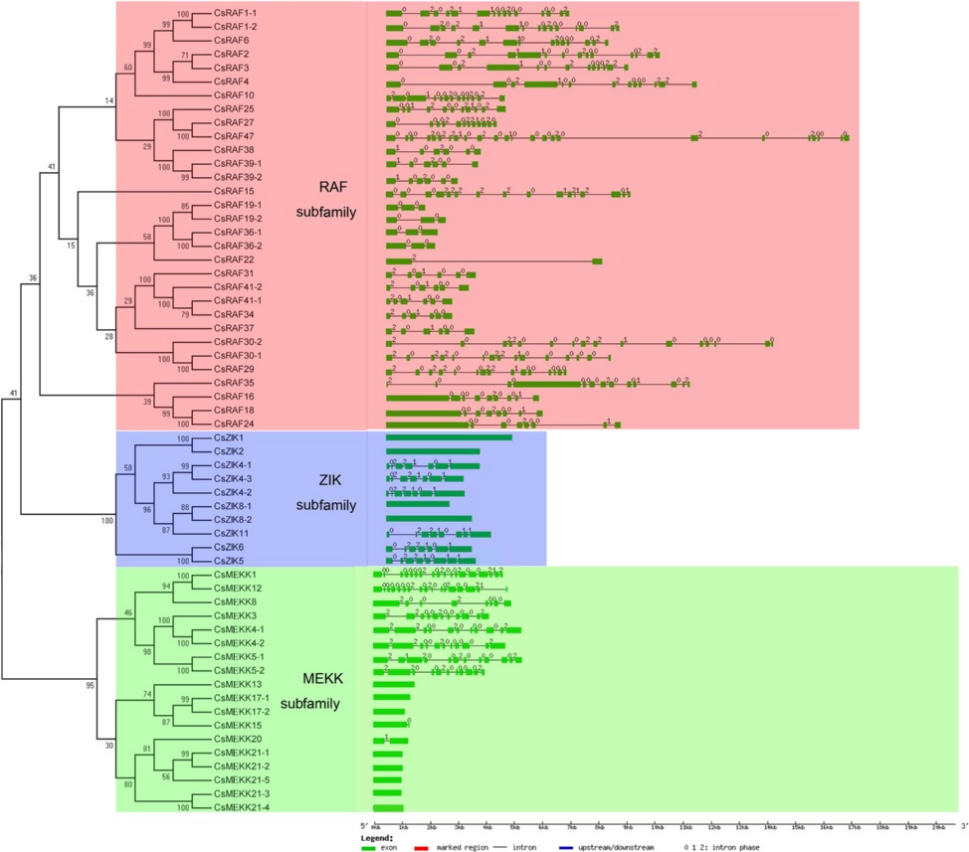
Phylogenetic analysis and gene structure of CsMAPKKKs in cucumber

Gene structure divergence plays an important role in the evolution of gene families and provides additional evidence to evaluate phylogenetic relationships. Therefore, the exon/intron organizations of all the MAPK cascade genes in cucumber were analyzed using the Gene Structure Display Server. The number of introns in the CsMAPK gene family varied from 1 to 10 (Fig. 7), which was consistent with the finding in tomato [39]. In the CsMAPKK gene family, members from subgroups C and D had no intron, whereas members in subgroups A and B had seven and eight introns, respectively (Fig. 8). Moreover, the exon/intron structures and intron phases in the MAPK and MAPKK gene families were conserved within the same subgroup but divergent between different subgroups. However, the numbers of introns were highly variable in CsMAPKKK (Fig. 9), even in the same subfamily, and ranged from 0 to 23 introns. In the MEKK subfamily, eight genes had no intron, whereas *CsMEKK15* and *CsMEKK20* only had one intron each. However, the other genes in the MEKK subgroups had 7 to 17 introns. A certain degree of conservation could be observed in the 10 CsZIK genes. Apart from four genes (*CsZIK1*, *CsZIK2*, *CsZIK8-1* and *CsZIK8-2*) without any intron, the ZIK genes had six or seven introns, which were consistent with studies on *Vitis vinifera* [52]. The RAF genes showed a variable intron number that ranged between 1 and 23 (Fig. 8). Most interestingly, we found that paralogous gene pairs in these three gene families generally shared highly similar gene structures (Figs. 7, 8 and 9). Collectively, the divergent exon-intron structures between the different phylogenetic subgroups showed that duplication events were likely to have occurred in ancient years, and the offspring genes evolved into diverse exon-intron structures to accomplish different functions in the cucumber genome. Comparison the number of introns in all the MAPK cascade genes in cucumber with their orthologs in *Arabidopsis*, rice showed that most of the orthologs contained the same number of introns (Additional file 9). It has been reported that orthology is based on evolution of introns in plants [57, 58]. Accordingly, our findings are consistent with previous studies and confirm that most of the orthologs of MAPK cascade genes in *Arabidopsis*, rice and cucumber are evolutionarily conserved in gene structure.

To further understand the potential functions and transcriptional regulation of these MAPK cascade genes, 1500 bp upstream regions of the transcriptional start site (ATG) were applied to identify the cis-regulatory elements. A large amount of stress-related (e.g., drought, extreme temperatures, high salinity, wounding, and disease) and hormone-related (e.g., auxin, abscisic acid, ethylene, and gibberellin) cis-elements were found in the putative promoter regions of the MAPK cascade genes in cucumber (Additional file 10). The existence of these cis-elements suggested that these MAPK cascade genes might have potential functions in stress adaptations and various hormone signaling pathways. A large number of similar cis-elements were found in MAPK cascade genes of tomato [39, 40] and *B. distachyon* [48].

### Chromosomal mapping and gene duplication

**Fig. 10:**
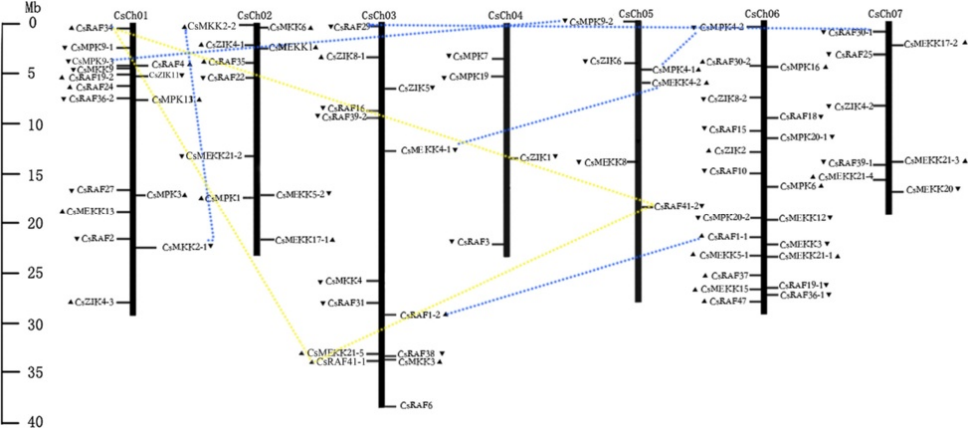
Chromosomal distributions and gene duplications of CsMAPKs, CsMAPKKs and CsMAPKKKs in cucumber genome. Dotted lines connect different MAPK, MAPKK, or MAPKKK genes that are present as duplicated gene pairs. Triangles indicate the upward or downward direction of transcription

To determine the chromosomal distribution and transcriptional direction of the MAPK cascade genes in cucumber, BLASTN searches were performed against the cucumber genome database using the DNA sequence of each MAPK cascade gene. All the MAPK cascade gene members were physically mapped to cucumber genome (Fig. 10). They were separately distributed on each chromosome individually, and no gene clusters were found based on Holub’s definition of the gene cluster [59]. Chromosome 6 had the highest number of MAPK cascade genes (21 genes), whereas chromosome 4 only had four genes (two MAPKs and two MAPKKKs). Moreover, the distribution of the three gene families was not random in cucumber chromosomes. For example, nine MAPK genes were located on chromosomes 1 and 6, but chromosomes 3 and 7 did not contain MAPK genes. Interestingly, all the CsMAPKK genes were present on chromosomes 1, 2, and 3. Although the CsMAPKKKs were distributed over all the seven chromosomes, the number on each chromosome differed, ranging from 2 (chromosome 4) to 16 (chromosome 6) (Fig. 10).

Gene duplication events, including tandem and segmental duplications, are thought to have important functions in the expansion of the MAPK, MAPKK, and MAPKKK gene families [35, 38-40, 48]. In this study, tandem and segmental duplication events in the CsMAPK, CsMAPKK, and CsMAPKKK gene families were investigated using the method reported by Gu et al. [60]. In general, we detected nine segments and no tandem duplication events in the cucumber MAPK, MAPKK and MAPKKK gene families, namely, two in CsMAPKs, one in CsMAPKK, and six in the CsMAPKKK gene family. These genes concerned all the chromosomes, except chromosome 4 (Fig. 10). The number of gene duplication events that occurred in the CsMAPK, CsMAPKK, and CsMAPKKK gene families were much fewer than those in other plants, such as rice [35, 61], maize [38], grape [52], and *B. distachyon* [48]. The recently reported whole-genome duplication event was absent and only a few tandem and segmental duplications have been shown to exist in the cucumber genome [5]. These phenomena may partly account for the small number of gene duplication events in the cucumber MAPK cascade gene families.

To elucidate the mechanisms of gene divergence after duplication of the MAPK cascade gene families in cucumber, the ratio of non-synonymous substitution rates (*K*_a_) and synonymous substitution rates (*K*_s_) was calculated. This ratio reflects the selective pressure acting on the protein. Generally, *K*_a_/*K*_s_ < 1 indicates negative or purifying selection; *K*_a_/*K*_s_ = 1 indicates neutral selection; and *K*_a_/*K*_s_ > 1 indicates positive selection [62]. In this study, the values of *K*_a_/*K*_s_ for the nine duplicated orthologous gene pairs were all lower than 1 (Additional file 11), which indicated that the MAPK cascade genes from cucumber had mainly experienced purifying selection pressure after the segmental duplications. These results demonstrated that functions of the duplicated gene pairs in the CsMAPK, CsMAPKK, and CsMAPKKK gene families did not diverge as much from each other during subsequent evolution.

### Evolutionary patterns and divergence of MAPK cascade genes in angiosperms

To reveal the evolutionary relationships of the three gene families in angiosperms, we further explored the duplication and diversification of MAPK cascade genes after the divergence of the dicots and monocots (approximately 160 million years ago). The sequences from two dicotyledonous plants (*Arabidopsis* and tomato) and two monocotyledonous crops (maize and rice) were obtained to compare the duplication and divergence of these three gene families with that of cucumber. The number of cucumber genes in the three gene families was lower than that of the other angiosperms (Additional file 12). Previous studies revealed that *Arabidopsis*, rice, maize, and tomato have 20, 16, 19, and 16 MAPKs and 10, 8, 9, and 5 MAPKKs, respectively [36, 37, 39, 40, 47], as well as 80, 75, 74, and 89 MAPKKK genes, respectively [32, 35, 38, 40]. However, the cucumber genome only encodes 14 MAPKs, 6 MAPKKs, and 60 MAPKKKs (Additional file 12). Moreover, in these three gene families, most of these genes were single-copy and/or located as a singleton. Huang et al. [5] reported that cucumber has seven pairs of chromosomes and a haploid genome of 367 Mb that encodes approximately 26, 682 genes; its genome is much smaller than that of maize (2500 Mb) [63] and tomato (950 Mb) [64]. However, the size of cucumber genome was similar with that of rice (389 Mb) [65], and three times that of *Arabidopsis* (approximately 120 Mb) [66]. Although the differences between the genome size and total number of predicted protein-encoding genes among the five sequenced plant species were apparent (Additional file 12), the number of MAPK cascade genes did not increase or decrease proportionally. Similar phenomena have been reported for other gene families in cucumber, such as WRKY [10] and NBS [7]. Based previous reports [7, 10], this phenomenon can be explained by the absence of the recent whole genome duplication events in cucumber genome. Another possible explanation is the limited number of segmental and tandem duplications in this genome [5].

To further investigate the molecular evolution and phylogenetic relationships among MAPKs, MAPKKs, and MAPKKKs in angiosperms, unrooted phylogenetic trees were constructed based on the full-length protein sequences of 84 MAPKs, 38 MAPKKs, and 318 MAPKKKs sequences from cucumber, *Arabidopsis*, tomato, maize, and rice (Additional files 13, 14 and 15). The phylogenetic tree revealed that MAPKs and MAPKKs in angiosperm clearly belonged to four distinct subgroups (A–D) (Additional files 13 and 14). Meanwhile, the MAPKKKs formed three subfamilies, namely, RAF, MEKK, and ZIK (Additional file 15). All the subgroups or subfamilies in MAPKs, MAPKKs, or MAPKKKs contained members from all five species (Additional files 12, 13, 14 and 15).

### Expression profiles in different tissues or organs

Increasing evidence has shown that MAPK cascade genes are widely involved in the growth and development of higher plants. In *Arabidopsis*, MPK3 and MPK6 play important roles in anther cell differentiation and normal anther lobe formation [67]. Meanwhile, AtMPK4 is required for male-specific meiotic cytokinesis [26]. The MAPK cascade YDA-MKK4/MKK5-MPK3/MPK6 regulates *Arabidopsis* inflorescence architecture by promoting localized cell proliferation [68]. AtMPK6 has been demonstrated to be involved in seed formation and the modulation of lateral and primary root development [69]. Another important *Arabidopsis* MAPK cascade MKK9-MPK6 plays an important role in leaf senescence [70]. GmMPK4 and GmMPK7 regulate plant growth and development in soybean [71, 72]. NPK1 in *Nicotiana* has been suggested to regulate cell size, cytokinesis, and plant growth [29]. Duan et al. [73] identified OsMKK4 as a factor for grain size in rice, and suggested a possible link between the brassinosteroids (BRs) and MAPK pathways during grain growth. However, as of this writing, the involvement of MAPK cascades in cucumber has not been demonstrated in the regulation of plant growth and development.

**Fig. 11:**
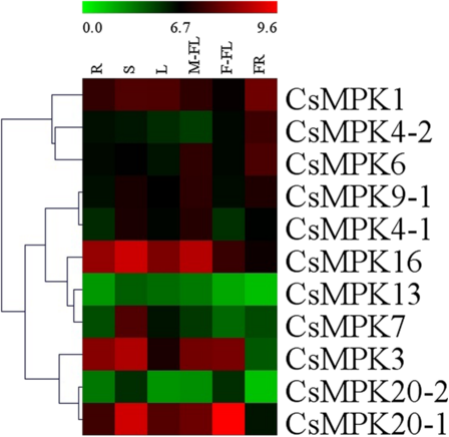
Expression profiles of CsMAPKs in different organs/tissues using qRT-PCR analysis. R: roots, S: stems, L: leaves, M-FL: male flowers, F-FL: female flowers, FR: fruits. All samples were run in triplicate and the data were normalized relative to the *EF1a* (accession number *EF446145*) related protein transcript levels. The expression levels of genes are presented in heatmap using fold-change values transformed to Log2 format by MeV4.8. The color scale and Log2 values (fold-change values) are shown at the bottom of heatmap. Genes were clustered according to their expression profiles

**Fig. 12:**
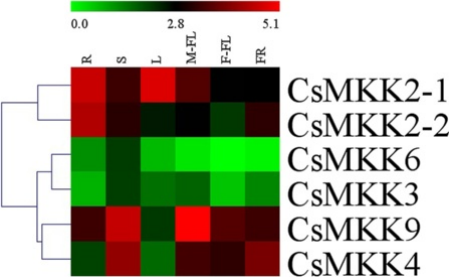
Expression profiles of CsMAPKKs in different organs using qRT-PCR analysis

**Fig. 13:**
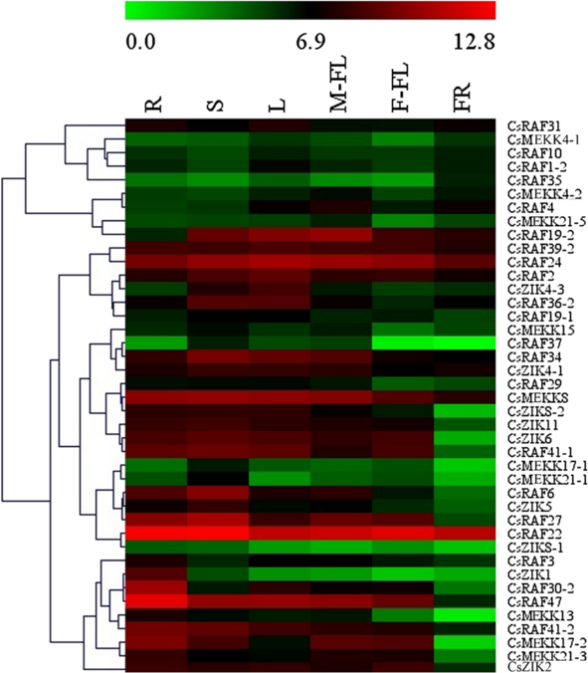
Expression profiles of CsMAPKKKs in different organs using qRT-PCR analysis

To gain insight into the temporal and spatial transcription patterns and putative functions of CsMAPK cascade genes in cucumber growth and development, qRT-PCR was performed to analyze the transcription levels in various tissues or organs, including the root, stem, leaf, flower, and fruit of plants. Given the high similarity of the nucleotide sequences, only 11 CsMAPKs, 6 CsMAPKKs, and 41 CsMAPKKKs were selected for expression analysis (Additional file 16). The expression levels of these genes are clustered and presented in heatmaps (Figs. 11, 12 and 13). The results revealed high alterations in transcript abundance among different MAPK cascade genes in cucumber. All the MAPK cascade members were expressed in at least one organ. Several proteins did not show striking differences in their expression levels among different organs or tissues. However, a small number of genes *(CsMPK13*, *CsMKK3*, *CsMKK6*, and *CsZIK8-1*) presented very low expression in all the tested organs (Figs. 11, 12 and 13). Specifically, *CsMPK4-2* and *CsMPK7* showed preferential expression patterns in the fruit and stem, respectively. Similarly, *CsRAF3* and *CsZIK1* of the CsMAPKKK gene family were predominantly expressed in the root, whereas *CsZIK5* had a relatively high expression level in the stem (Fig. 13). Therefore, these genes may mainly function in organ- or tissue-specific development in cucumber. Notably, *CsMPK4-1*, the duplicated gene of *CsMPK4-2*, exhibited relatively high transcript abundance in the stem and male flowers, suggesting the different expression patterns between duplicated gene pairs (Fig. 11). Similar results were found in other duplicated gene pairs. For example, *CsMKK2-1* of the CsMAPKK gene family was expressed in all tested organs with relatively higher abundance, whereas *CsMKK2-2* was mainly expressed in the root, stem, and fruit (Fig. 12). Although the duplicated gene pairs had higher similarity in terms of their amino acid and nucleotide sequences, they may not be involved in the same pathway or do not have similar functions. Meanwhile, several paralogs showed highly similar expression patterns. For example, *CsMEKK4-1/CsMEKK4-2* and *CsRAF34/CsRAF41-1/CsRAF41-2* demonstrated subfunctionalization in the course of evolution. Interestingly, most MAPK cascade genes in cucumber presented similar expression profiles with their homologs in *Arabidopsis* or rice [48]. For example, *CsMPK13*/*AtMPK13* and *CsRAF10*/*OsMAPKKK5* were expressed in nearly all the detected organs/tissues with low abundance (Figs. 11 and 13), suggesting they might have conserved functions retained from the same ancestral gene [48, 35]. However, some orthologous genes displayed quite different expression patterns among cucumber, *Arabidopsis* and rice. For instance, *CsMPK7* had higher expression in stem than that of other organs (Figs. 11), whereas *OsMPK7* was constitutively expressed in nearly all the organs with high abundance [48]. The divergences in expression profiles between paralogs or orthologs revealed that some of them may acquire new functions after duplication in the evolutionary process.

### Expression profiles under various stress conditions and plant hormone treatments

**Fig. 14:**
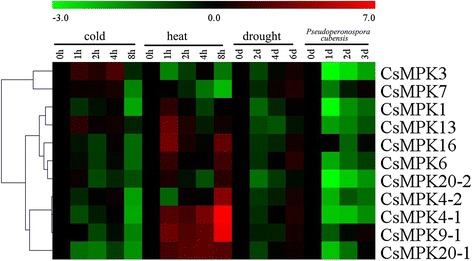
Expression patterns of CsMAPKs under abiotic and biotic stress treatment in cucumber by qRT-PCR analysis in heatmap. Details of the treatments are reported in Materials and Methods

**Fig. 15:**
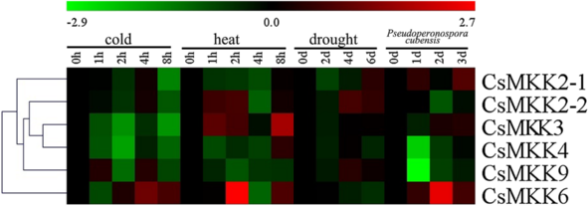
Expression patterns of CsMAPKKs under abiotic and biotic stress treatment in cucumber by qRT-PCR analysis in heatmap

**Fig. 16:**
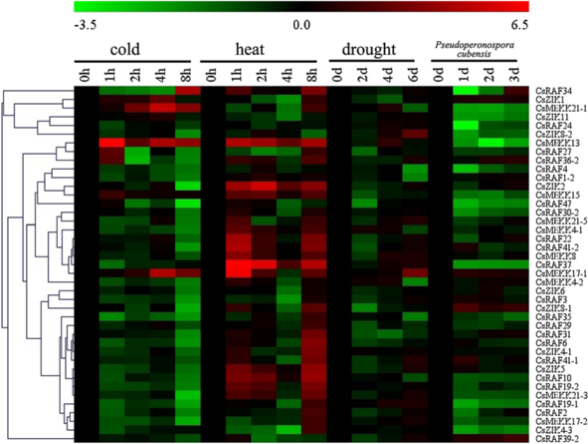
Expression patterns of CsMAPKKKs under abiotic and biotic stress treatment in cucumber by qRT-PCR analysis in heatmap

Numerous members of the MAPK pathways have been well characterized in terms of their response to various biotic and abiotic stresses in plants [16]. An cascade with MEKK1, MKK2, MPK4, and/or MPK6 in *Arabidopsis* has been shown to respond to salt, drought, and cold stress [74]. The MEKK1-MPK4 cascade is an essential component of ROS metabolism [75], whereas the MKK1-MPK6 cascade is involved in H_2_O_2_ metabolism [76]. Recent studies demonstrated that MAPK proteins in cucumber participate in the signal transduction of stress response. A *Trichoderma*-induced MAPK (TIPK) was involved in fungal defense responses [44], whereas CsNMAPK regulated NO_3_^−^ stress [42], ROS, and osmotic adjustment under salt stress [43]. In the present study, we conducted qRT-PCR analyses to examine the expression levels of the CsMAPK cascade genes in response to three different abiotic stresses (cold, heat, and drought) and one biotic stress (*Pseudoperonospora cubensis*). All the 58 detected CsMAPK cascade genes showed differential expression patterns in response to more than one stress (Fig. 14,15 and 16, Additional files 17, 18 and 19). All the CsMAPKs were downregulated after *P. cubensis* treatment whereas the majorities (except *CsMPK3*, *CsMPK7*, and *CsMPK13*) were downregulated after cold treatment. However, most of the CsMAPKs were upregulated under heat stress, except *CsMPK3* and *CsMPK7*. Meanwhile, all the CsMAPKs were initially downregulated for the first 2 d before they were significantly upregulated after drought treatment (Fig. 14, Additional file 17). Interestingly, similar expression patterns were observed for CsMAPKKKs in the present study (Fig. 16, Additional file 20). The expression levels of CsMAPKKs irregularly increased or decreased following after cold, heat, drought, or *P. cubensis* treatment (Fig. 15, Additional file 18). For example, *CsMKK4* transcripts decreased in abundance when plants were subjected to cold, drought, and *P. cubensis* stress. However, the *CsMKK4* transcripts exhibited a pronounced increase at the last time point (8 h post-treatment), which was induced by heat stress. By contrast, the expression pattern of *CsMKK6* was dramatically different from that of *CsMKK4* or any other CsMAPKKs. The expression profiles of the CsMAPK cascade duplicated gene pairs were also compared. Most of the detected duplicated gene pairs (*CsMPK4-1/CsMPK4-2*, *CsMEKK4-1/CsMEKK4-2*, and *CsRAF34/CsRAF41-1/CsRAF41-2*), excluding *CsMEKK2-1/CsMEKK2-2*, showed similar expression profiles under cold, heat, drought, or *P. cubensis* treatment (Figs. 14, 15 and 16). That is worth mentioning that some homologous genes among cucumber, *Arabidopsis* and rice showed quite different expression patterns under the same stress conditions. For example, *AtMPK7* was significantly up-regulated whereas *CsMPK7* was markedly down-regulated under the cold stress conditions. Moreover, *OsMKK4* was up-regulated under the drought and cold stress conditions, while *CsMPKK4* was down-regulated under the same stress conditions [48].

Several examples implicate plant MAPKs in hormone signaling by regulating phytohormone synthesis and mediating antagonistic or synergistic effects between different hormones [77]. The MKK3/MPK6 module was proposed to participate in jasmonic acid (JA) signaling [78]. The relationship of MAPK signaling pathways and abscisic acid (ABA) in plant abiotic stress responses was recently characterized [76]. Unfortunately, studies on the cross-talk between CsMAPK pathways and hormone signaling in cucumber have been limited. In the present study, changes in the transcription of CsMAPK cascade genes in response to the plant hormones JA and ABA were compared (Additional files 20, 21 and 22). The transcript levels of most CsMAPK cascade genes considerably varied after exogenous JA and ABA treatment, thereby implying their involvements in plant hormone signaling. However, *CsMPK3* transcript abundance decreased after exogenous JA treatment, but increased after exogenous ABA treatment (Additional file 20). Therefore, CsMPK3 may be associated with different biological pathways in response to JA and ABA. Interestingly, a previous study showed that the expression levels of the MPK3 gene from rice were upregulated in response to treatment with JA [79], which indicated the different roles of MPK3 in cucumber and rice JA signaling. Lu et al. [80] found that MPK3 is activated by ABA in *Arabidopsis* seedlings, which implied that CsMPK3 might play a role similar to AtMPK3 during ABA signaling. Evidence of the involvement of MAPKKKs in cucumber stress and hormonal response is limited. The known functions of CsMAPK cascade proteins or predicted biological roles based on the experimentally characterized MAPK cascade genes in *Arabidopsis* were summarized in Additional file 3. Further analysis is required to elucidate the exact functions of these cucumber MAPK cascade genes in response to various stress and plant hormones. Our aforementioned observations provide the first genome-wide survey on the expression patterns of specific cucumber MAPKs, MAPKKs, and MAPKKKs in various biotic and abiotic conditions, as well as plant hormone signaling.

## Conclusions

The present study is the first to provide a full list of the MAPKs, MAPKKs, and MAPKKKs in cucumber. A genome-wide search of the cucumber protein database by BLASTP identified 14 MAPKs, 6 MAPKKs, and 59 MAPKKKs in cucumber. EST hits or full-length cDNA sequences supported the existence of these genes. CsMAPKs and CsMAPKKs were grouped into four subgroups (A, B, C, and D), whereas CsMAPKKKs were divided into three subfamilies (MEKK, ZIK, and RAF). The conserved domains/motifs, phylogenetic relationships, and gene structures strongly supported the identity of each subgroup. Our results demonstrated that segment duplications could be the main factors influencing the expansion of the MAPK cascade gene families in cucumber. The expression profiles of MAPK cascade genes in various organs or tissues were discussed, as well as their responses to stresses and exogenous plant hormones. Most of the selected genes were expressed in all the analyzed organs, whereas some genes were preferentially expressed in one or more specific organs. Furthermore, a majority of the MAPK cascade genes could be induced by biotic and abiotic stress treatment; most of the genes could interact with plant hormones, such as ABA and JA, during plant development or in defense pathways. Therefore, the information generated in this work provides a significant foundation for further investigation of the regulatory mechanism of the cucumber MAPK cascade in response to extracellular or intracellular stimuli for various biological functions.

## Methods

### Identification of MAPK, MAPKK, and MAPKKK gene families in *C. sativus*

The method used to identify all the putative MAPK, MAPKK, and MAPKKK family genes was similar to that used for rice and *B. distachyon* [35, 48]. The predicted peptide sequences were downloaded from the cucumber Genomics Database (http://www.icugi.org/cgi-bin/ICuGI/genome/index.cgi?organism=cucumber). Meanwhile, 143 MAPK, 67 MAPKK, and 534 MAPKKK protein sequences from seven plant species (*A. thaliana*, *Populus trichocarpa*, *Oryza sativa*, *Zea mays*, *Glycine max*, *Solacum lycopersicum* and *Brassica napus L*.) were retrieved from the PHYTOZOME v9.1 database (www.phytozome.net). These sequences were used as queries to search against the cucumber protein databases with the BLASTP program with an e-value of 1e^−10^ as the threshold. A further search was performed with Hidden Markov Model (HMM) analysis using HMMER 3.0 programme [48]. The HMMER hits containing the serine/threonine-protein kinase-like domain (PF00069) were compared with BlAST results and parsed by manual editing. All data were checked for redundancy by self-BLAST and no any alternative splice variants were considered. MAPKKK genes were only accepted if they displayed one of the three consensus sequences: 1) GTXX (W/Y) MAPE, 2) GTPEFMAPE (L/V/M) (Y/F/L), 3) G (T/S) PX (F/Y/W) MAP EV [35]. MAPKK genes were only accepted if they contained the conserved sequences D (L/I/V) K and S/TxxxxxS/T, while MAPK genes should contain the TXY motif [48]. Then, the online software Conserved Domain Database of NCBI (http://blast.ncbi.nlm.nih.gov) and SMART database (http://smart.embl-heidelberg.de/) were used to further confirm the predicted MAPK cascade genes. Finally, all the sequences were further verified with similarity searches by BLASTN at the cucumber Genomics Database against the EST sequences and unigenes of cucumber.

The isoelectric point (pI) of the obtained proteins was predicted using Compute pI/Mw software (http://web.expasy.org/compute_pi/). Subcellular localization prediction of each gene was conducted using the TargetP software of the CBS database (http://cello.life.nctu.edu.tw/) [81].

### Multiple sequence alignment, phylogenetic analysis, and gene structure construction

Multiple sequence alignments were generated using ClustalX v1.81 [82]. The PlantsP database (http://plantsp.genomics.purdue.edu/index.html) and online MEME software (http://meme.sdsc.edu/meme/meme.html) were used to conduct domain and motif detection [83]. Phylogenetic analysis was performed based on the full-length protein sequences using the MEGA 5 program by the NJ method, and a bootstrap test was carried out with 1000 interactions [84]. The exon-intron distribution pattern and splicing phase were obtained by the Gene Structure Display Server (http://gsds.cbi.pku.edu.cn/index.php).

### Cis-element analysis of putative promoter regions

To investigate cis-elements in the promoter regions of all the obtained genes, we downloaded 2 kb of the genomic DNA sequences upstream of the initiation codon (ATG) of each gene from the cucumber database. The putative cis-regulatory elements in the promoter sequences were analyzed via the PLACE database (http://www.dna.affrc.go.jp/PLACE/).

### Chromosomal location and gene duplications

The nucleotide sequences of all these genes were further used as query sequences for BLASTN searches against the cucumber chromosomes. The precise locations of these genes in cucumber were then detected.

Gene duplication events were defined based on the following three criteria: (a) the alignment covered >80 % of the longer gene; (b) the aligned region had an identity >80 %; and (c) only one duplication event was counted for the tightly linked genes [38, 60]. A block of duplications was defined if more than one gene was involved in the duplication.

### Estimating *K*_a_/*K*_s_ ratios for duplicated gene pairs

The *K*_s_ and *K*_a_ were calculated by the DnaSP v5.0 software (DNA polymorphism analysis) [85]. Subsequently, the *K*_a_/*K*_s_ ratio was analyzed to assess the selection pressure on duplicated genes.

### Plant materials, growth conditions, and treatments

Cucumber plants of the ‘Jinyan No4’ cultivar were reared in growth chambers at 28 ± 1 °C with a photoperiod of 16 h light/8 h dark. Plants were illuminated with a light intensity of 400 μmol m^−2^ s^−1^. The roots, leaves, stems, female flower buds (approximately 3 d before anthesis), male flower buds (approximately 1.0 cm in length), and fruits (10 day after pollination) were collected from flowering plants for tissue expression analysis.

Three-week-old seedlings were used for all abiotic and biotic treatments. For heat or cold treatment, the seedlings were subjected to 35 ± 1 °C or 4 ± 1 °C conditions, respectively. The samples for RNA extraction were collected at 0, 1, 2, 4, and 8 h after treatment. For dehydration treatment, the plants were treated according to the methods of a previous study [10]. For disease treatment, *P. cubensis* was used to infect the seedlings, and leaves were collected at 0, 1, 2, and 3 days after infection. For hormone treatments, the seedling leaves were sprayed with 100 mM methyl JA or 100 mM ABA and then sampled at 0, 1, 2, 4, and 8 h intervals [86].

All the samples from three biological replicates were frozen in liquid nitrogen immediately and stored at −80 °C.

### RNA extraction and qRT-PCR expression analysis

Total RNA was extracted with TRIZOL reagent (Invitrogen, USA) according to the manufacturer’s instructions. The first cDNA strand was generated using a Takara Reverse Transcription System (Japan) following the manufacturer’s protocol. Real-time PCR analyses were performed using the primer pairs designed using the Primer (version 5.0) software (Additional file 16). The specificity of each primer to their target genes was checked using the BLASTN program of the cucumber genomic database. A sample of cDNA (1 μg) was subjected to each qRT-PCR reaction in a final volume of 20 μl containing 3 pmol specific primers and 12.5 μl SYBR Green Master Mix Reagent (Takara, Japan). Two biological and three technical replicates for each sample were performed in the real-time PCR machine (BIO-RAD CFX96, USA). The cucumber *EF1a* (accession number EF446145) gene was used as an internal control to calibrate relative expression. The 2^-ΔΔCT^ method was used to calculate the relative expression level of the target gene [87]. The qRT-PCR data were clustered with Pearson correlation distance metric using the average linkage method by MeV 4.8 [88].

### Availability of supporting data

The data set supporting the results of qRT-PCR assay is available in the Gene Expression Omnibus (GEO) repository. The accession numbers are GSE68436 (http://www.ncbi.nlm.nih.gov/geo/query/acc.cgi?acc=GSE68436) and GSE68438 (http://www.ncbi.nlm.nih.gov/geo/query/acc.cgi?acc=GSE68438). The sequences information of plant MAPK cascade genes to construct phylogenetic trees was deposited in the LabArchives under the DOI ‘10.6070/H4DV1GV5’ (https://mynotebook.labarchives.com/share/ganglu/MjQuN3w5MTM0My8xOS9UcmVlTm9kZS8zNDg0NTc1MDEzfDYyLjc=).

## Additional files

Additional file 1:
**Gene sequences of CsMAPK cascade genes.** A: The coding sequences of CsMAPK caascade genes. B: The protein sequences of CsMAPK caascade genes. C: The 2 kb genomic DNA sequences upstream of the initiation codon. Additional file 2:
**Sequence alignment analysis of the CsMAPK cascade genes with two or more copies.** A: Alignment analysis of the nucleotide sequences of the CsMAPK cascade genes with two or more copies. B: Alignment analysis of the peptides sequence of the CsMAPK cascade genes with two or more copies. Additional file 3:
**Classification and summary of the functions or predicted fuctions of the cucumber MAPK cascade gene families.** The putative functions of cucumber MAPK cascade genes were predicted based on the experimentally characterized homologues from *Arabidopsis*. Additional file 4:
**Sequence similarity analysis of MAPK cascade genes between** ***Arabidopsis*** **and cucumber.**
Additional file 5:
**Alignment of multiple cucumber,** ***Arabidopsis*** **and rice MAPK, MAPKK, and MAPKKK domain amino acid sequences.** Alignment was performed using ClustalX. The conserved amino acid signature of each subgroup is highlighted in red box. Additional file 6:
**The ten conserved motifs of MAPKs detected by the online tool MEME.**
Additional file 7:
**The ten conserved motifs of MAPKKs detected by the online tool MEME.**
Additional file 8:
**The ten conserved motifs of MAPKKKs detected by the online tool MEME.**
Additional file 9:
**The nomenclatured gene name, locus ID and intron number of MAPK cascade genes from cucumber,** ***Arabidopsis*** **and rice.**
Additional file 10:
**The cis-elements in promoter sequences of MAPK, MAPKK, and MAPKKK genes in cucumber.**
Additional file 11:
**Ka/Ks and selection type analysis for the duplicated gene pairs in CsMAPKs, CsMAPKKs, and CsMAPKKKs.**
Additional file 12:
**The numbers of CsMAPK, CsMAPKK, and CsMAPKKK in cucumber, Arabidopsis, rice, tomato, and maize.**
Additional file 13:
**The phylogenetic tree of MAPK genes from cucumber,** ***Arabidopsis*** **, tomato, rice, and maize.**
Additional file 14:
**The phylogenetic tree of MAPKK genes from cucumber,** ***Arabidopsis*** **, tomato, rice, and maize.**
Additional file 15:
**The phylogenetic tree of MAPKKK genes from cucumber,** ***Arabidopsis*** **, tomato, rice, and maize.**
Additional file 16:
**Primer sequences of MAPK, MAPKK, and MAPKKK genes for qRT-PCR expression analysis.**
Additional file 17:
**Change of Expression levels of CsMAPKs under abiotic and biotic stress treatment in cucumber by qRT-PCR analysis in line map.**
Additional file 18:
**Change of expression levels of CsMAPKKs under abiotic and biotic stress treatment in cucumber by qRT-PCR analysis in line map.**
Additional file 19:
**Change of expression levels of CsMAPKKKs under abiotic and biotic stress treatment in cucumber by qRT-PCR analysis in line map.**
Additional file 20:
**Expression patterns of CsMAPKs with exogenous JA and ABA treatments by qRT-PCR analysis.**
Additional file 21:
**Expression patterns of CsMAPKKs with exogenous JA and ABA treatments by qRT-PCR analysis.**
Additional file 22:
**Expression patterns of CsMAPKKKs with exogenous JA and ABA treatments by qRT-PCR analysis.**

## Abbreviations

BLAST: Basic local alignment search tool
EST: Expressed sequence tag
JA: Jasmonic acid
*K*_a_: Non-synonymous substitution rate
*K*_s_: Synonymous substitution rate
MAPK: Mitogen-activated protein kinase
MAPKK: MAPK kinase
MAPKKK: MAPK kinase kinase
TAIR: The arabidopsis information resource
MEME: Multiple expectation maximization for motif elicitation
NCBI: National Center for Biotechnology Information
QRT-PCR: Quantitative reverse transcription polymerase chain reaction
ROS: Reactive oxygen species
