# Supplementary material for: Genome-wide identification of MAPK, MAPKK, and MAPKKK gene families and transcriptional profiling analysis during development and stress response in cucumber
Source: BMC Genomics. 2015 May 15;16(1):386. doi: 10.1186/s12864-015-1621-2 (PMC4432876; doi:10.1186/s12864-015-1621-2)
Supplement: Additional file 1: — Gene sequences of CsMAPK cascade genes. A: The coding sequences of CsMAPK caascade genes. B: The protein sequences of CsMAPK caascade genes. C: The 2 kb genomic DNA sequences upstream of the initiation codon. [file 12864_2015_1621_MOESM1_ESM.docx]

**A: Coding sequences of CsMAPK cascade genes.**

>Csa2M361890.1

ATGGGAGCTTTATCCATATCTTTTATGGACATGTTCACAAAATCCACCAGATATTTCTCCATCGACGTCAGTGTAAACAAAATGTCAGCACAAGAAAACGGGACCCATCTAGAAAGCGAGATTAAAGAAGTAGAACAAGAAGTAGTAGAAGAGGGTAATAATCCAAATCCACAAATCATTGATTATATTACATACCCAAATTGCAAATTTACCGAAGGTGGAATCAAAAGCTGCGCGCGTGAGCTTCCCCATCTAGTTGCCAAGAAATTTCCACTTCCACACTCTCAATTCACTCGATTCCACGCTTTTCAGCTTCCAATTCGAGCTGGGGTTCTGTTAATTTTGGGGTTTTTCTTCCTTTCCCCATCTGGGGTTTCTCTGTTCCTTCTGATTCTATCATGGAATCTACTGATTTCCACTTTCTTCAAGGTTTTGGAGGCTAATTTTGAGCTGGATCAGTGTTCCTTGTCTGTTCTAATGTTGTACCTTACATCTGTTTGGCTTTTGTTGAGCTTGGTGGGAATGGCGACCCCTGTTGAGCCTCCAAATGGGGTTAGATCTCAAGGAAAACATTACTATTCAATGTGGCAGACATTGTTCGAGATTGATACTAAATATGTACCTATCAAGCCTATTGGTCGAGGGGCATACGGTATTGTGTGTTCTTCTGTGAATAGGGAAACAAATGAGAAAGTTGCAATAAAGAGAATACATAATGCGTTTGAGAATCGTATCGATGCACTCCGGACTTTGCGGGAACTGAAGCTCCTTAGGCATCTTCGCCACGAAAACGTCATATGTTTGAAAGATGTGATGATGCCTATCCATAGGAGAAGTTTCAAAGATGTCTATTTGGTTTATGAACTGATGGATACTGATCTGCATCAGATTATTAAATCTTCTCAAACTCTTACCAATGACCATTGCCAATATTTCCTCTTTCAGTTGCTCCGAGGCCTAAAGTATCTGCATTCTGCAAACATTCTACATCGGGACTTGAAGCCGGGGAACCTTCTTGTCAATGCAAATTGTGATCTTAAGATATGTGATTTTGGTTTAGCACGTACAAGCAATGGGAAGAATCAATTTATGACTGAGTATGTCGTAACTCGCTGGTATCGAGCCCCAGAACTGCTGCTCTGCTGTGAGAATTACGGGACGTCAATTGATGTGTGGTCTGTAGGATGCATCTTTGCTGAGCTTCTCGGTAGGAAACCTATCTTCCCTGGTACAGAATGTTTGAATCAACTCAAATTGATAATTAACCTACTGGGAAGCCAGAGAGAGGAAGATCTTGAGTTCATAGACAATCCAAAGGCAAGGAGGTATATAAAATCCCTTCCATACTCTCCTGGAGCTCCTCTTTCCCGTCTTTATCCGAGCGCTCATCCTCTCGCAATTGATCTGCTACAAAAGATGCTTGTCTTTGACCCATCAAAGAGGATTAGTGTAACTGAGGCATTACAGCATCCTTATATGTCCCCACTGTATGATCCAAACAGCAATCCTCCAGCTCAGGTGCCAATCGATCTTGAGATAGATGAGGAACTTGGAGAAGAAATGATACGAGAGATGATGTGGAAAGAAATGCTTCATTACCACCCCGAAGACCTGGAAGAACACGCAGAAATGACACGTTTCCACCCCGAACCTACTACAAGTAGTGCCGCAGTGTATTCTTAA

>Csa1M479630.1

ATGGCTGATGTTGGTCAGAACAACCCCGCTGATTTTCCAGCTCTCCCAACCCATGGTGGCCAATACGTTCAGTATAACATTTTTGGGAATCCCTTCGAAATCACCTCCAAATATCGTCCTCCGATTATGCCTATTGGTCGCGGCGCATACGGAATCGTTTGTTCTGTTTTGAATTCGGAGACCAACGAAATGGTTGCGGTTAAGAAGATTGCTAACGCGTTTGATAACCATATGGATGCGAAGAGAACGCTCCGTGAGATTAAGCTTCTACGCCATTTGGATCATGAAAATGTAATAGGCATAAGAGATGTGATTCCTCCACCTTTACGGAGAGAATTCAATGATGTCTACATTTCGACTGAACTAATGGATACTGATCTTCACCAAATAATCCGCTCCAACCAAAGTTTATCAGAAGAGCATTGTCAGTATTTCCTTTATCAGATTCTCAGAGGACTGAAATACATACATTCGGCAAATGTCATTCATCGAGACTTGAAACCAAGCAATCTATTGCTTAATGCAAACTGTGATCTTAAAATATGTGACTTTGGTCTTGCTCGACCAACTTCTGAAAATGAATGCATGACGGAATATGTTGTGACAAGATGGTACAGAGCACCTGAGCTTCTTTTGAACTCTGATTATACAGCTGCAATCGATATATGGTCTGTTGGTTGCATCTTTCTGGAGCTTATGAATAGAAGGCCTTTATTTCCAGGCAGGGATCATGTGCATCAGATGAGATTATTGACTGAGCTTCTTGGCACACCAAGCGAGTCAGATCTTGGTTTTATTCGAAATGAAGACTCGAAAAGATATCTTCGACAACTACCTCCACATCCTCGTCAGCCATTAGCAACGGTTTTTCCACATGTTCATCCATTAGCAATTGATCTTGTGGATAAAATGTTGACATTTGACCCAACAAAGAGAATTACTGTTGAAGAAGCGTTAGCACATCCGTACCTAGAAAGATTGCATGACATAGCTGATGAGCCGGTTTGCTCAGAGCCATTCTCGTTTGAGTTTGAGCAACAATACTTAGACGAAGAACAGATGAAGGAGATGATTTACAGAGAGGCATTGGCACTCAATCCAGAATTTGCATGA

>Csa5M152810.1

ATGGCTACTAAAGAATCGAGTTCTACCACTGCCACTGAAGGCAAGATTAAAGGCGTTCTTACTCATGGTGGGCGATATGTGCAGTATAATGTGTACGGTAACTTGTTTGAGGTTTCAGCCAAGTATGTTCCTCCTTTGCGACCTATTGGTAGAGGTGCTTATGGTCTTGTTTGTGCTGCTGTAAATTCAGAGACCCATGAAGAGGTTGCCATCAAGAAAATTGGAAATGCATTTGATAACATAATCGACGCCAAAAGGACATTGAGAGAAATTAAGCTTCTTTGCCACATGGAACATGAAAATATTATTGCTATTAGAGACATCATTAGGCCGCCAAAAAGAGAGGTTTTTAATGATGTGTATATTGTTTACGAATTGATGGACACTGATCTTCATCAAATAATTCGTTCTGACCAACCACTGACGGATGATCATTGCCAGTACTTTTTATATCAGTTATTGCGTGGGCTGAAATATGTACACTCGGCCAAGGTCCTGCACCGTGATCTCAAGCCGAGCAACCTGCTTCTGAATGCAAATTGCGACCTTAAAATTGGAGACTTTGGATTGGCAAGGACGACTTCTGAAACCGACTTCATGACTGAATATGTGGTTACTCGCTGGTACCGAGCACCAGAATTGCTCCTCAATTGTTCAGAATACACTGCTGCTATTGATGTTTGGTCTGTAGGATGCATACTTGGTGAGATAATGACCAGAGAACCTCTCTTCCCTGGCAAAGATTATGTTCATCAACTCAGACTTATAACTGAGCTACTAGGATCACCAGATGATGCCAGCCTAGGATTTCTCCGAAGTGATAATGCTCGAAGATATGTCAAGCAGCTTCCACAATACAGAAAACAACAATTCTCAGCTAGATTTCCCAACATGTCTCCATCTGCCCTCGATCTTCTCGAAAAGATGCTCGTATTTGATCCCAACAAACGCATCACAGTGGAGGAAGCACTTTGTCACCCATACTTGCAATCTCTTCACGACATCAATGACGAGCCAGTCTGTGCCAGGCCTTTCAATTTTGATTTCGAGCAACCGTCGTGCACTGAAGAGCATATTAAAGAACTGATCTGGAAAGAATCCGTGAGGTTCAACCCCGACGAATCCGCTAGGAGGACGACTCTTTGCGTTTGA

>Csa6M006730.1

ATGGAAAACAGCTCTTTTTATCAGAACATCAGGGGAGAGCCCACTCATGGTGGTCAATACATTCAATACAATGTGTATGGGAATCTGTTTGAAGTTTCTAGGAAGTATACTCCACCCATCAGGCCAGTTGGGAGAGGAGCTTATGGAATTGTGTGTGCTGCTTTGAACTCAGAAACAAATGAAGATGTTGCAATTAAGAAGGTTGGCAAGGCATTTGACAATAGAATTGATGCTAAAAGGACCTTACGAGAAATCAAGCTTCTTCGACACATGGATCATGAAAATATTATTGCTCTCAGAGATATTATAAGACCTCCTCAGAAGGAGAATTTCAATGATGTATACCTTGTTTATGAATTAATGGACACAGATCTAAATCAGATTATACGGTCCAACCAATCATTGACTGATGACCATTGCCGGTACTTTCTATATCAGTTGTTACGAGGGCTAAAATATGTGCATTCTGCTAATGTTCTACACCGTGATTTGAAACCAAGCAATTTGTTCCTCAATGCAAATTGTGACTTAAAAATTGGAGACTTTGGCCTTGCAAGGACAACTTCTGAAACTGATTTTATGACCGAGTATGTTGTTACTCGGTGGTATCGTGCACCCGAATTGCTCCTTAATTGTTCAGAATACACTGGAGCAATAGATATTTGGTCTGTTGGCTGTATACTTGGTGAAATTATGCACAGAAAACCATTGTTTCCAGGAAAAGACTATGTTCATCAGCTAAAACTTATCACTGAGCTCATTGGGTCACCAGATGAATCGAGTCTTGGATTCTTACGAAGTGACAATCCAAGAAGATATTTTAGGCATCTTCCTCACTTCCCCAAGCAACAGTTTTCCTCTAAATTTCCCACCATGTCTCCTGCTGCTATTGACTTGCTTGAGAAGATGCTAGTCTTTGATCCTACTAAGCGCATTACAGTTGATGAGGCGTTGTGCCATCCATATTTAGCACCTCTTCATGATATCAATGAGGAGCCAGTATGTCCAAGGCCTTTCAGTTTCGATTTCGAGCAACCAACGTACACCGAAGAAAACATCAAGGAACTTGTATGGAGGGAATCCTTGAGGTTCAACCCCGGTCCCGCTTTTTGA

>Csa6M365750.1

ATGGACGACGGAGGAGCTTCTCAGCCGGACGACACCGTCATGTCGGAGGCGGCGTCTGTACCTCCACCACAGCATGACCCGGCGGCGCAACAGCAACATCAGCATCAGCCGCCGTCGATGGGGATGGAAAATATTCCGGCGACTTTGAGCCATGGAGGGAGATTTATTCAGTATAATATATTTGGTAACATCTTTGAAGTTACGGCCAAGTACAAGCCTCCTATTATGCCTATTGGCAAAGGCGCTTACGGCATCGTCTGTTCTGCTCTCAACTCTGAGACGAACGAGCATGTGGCGATTAAGAAGATTGCTAATGCGTTTGATAACAAGATCGATGCTAAGAGAACTCTTCGTGAGATCAAGCTTCTTCGGCATATGGATCATGAAAACGTTGTTGCGATTAGGGATATCATACCTCCACCTCTAAGGGAAACATTTAATGATGTTTATATCGCATATGAGCTAATGGATACCGACCTTCATCAAATAATTCGTTCAAACCAAGCATTATCAGAGGAGCATTGTCAGTATTTCCTGTACCAGATACTTCGTGGATTGAAGTACATACATTCGGCTAATGTTCTGCACAGAGATTTGAAACCTTCCAATCTGCTATTAAATGCGAACTGCGACCTGAAAATATGCGATTTCGGACTTGCTCGTGTTACTTCTGAAACTGACTTCATGACAGAATATGTGGTTACTAGATGGTACCGTGCACCAGAGCTCTTACTTAATTCATCTGATTACACTGCAGCTATTGATGTCTGGTCTGTTGGTTGTATTTTTATGGAACTAATGGATCGGAAGCCCTTGTTTCCTGGTCGAGATCACGTGCATCAGTTACGCTTGCTTTTGGAGCTGATTGGCACTCCATCAGAGGCTGATCTTGGTTTTTTGAACGAGAATGCTAAAAGATACATACGGCAATTACCTCATTACCATCGTCAATCATTCACCGAAAAGTTTCCACATGTCCATCCTGCAGCCATTGATCTGGTGGAGAAGATGCTAACATTTGATCCAGGACAGAGAATTACCGTTGAAGACGCTCTAGCTCATCCTTATTTGACTTCATTACACGACATTAGTGATGAGCCTGTCTGCATGACACCCTTCAGCTTTGATTTCGAGCAGCATGCGCTTACCGAGGAACAGATGAAAGAGCTGATCTATCTAGAGGCGCTTGCATTTAACCCCGAGTATCATCACCAATAA

>Csa4M045070.1

ATGGCTACGTTTGTGGAGCCACCGAGCGGAATCAGATCGATGGGGAAGCATTATTATACAATGTGGCAAACTCTGTTTGAAGTTGATACTAAATATGTCCCGATCAAGCCGATTGGGCGAGGTGCTTATGGTGTAGTGTGTTCTTCGATTAACCGAGAGACTAACGAGAAAGTGGCGATCAAGAAAATTCATAATGTGTTTGAGAATCGTACGGACGCCATGAGAACACTGAGGGAGCTGAAGCTATTGAGGCACATACGGCATGAGAATGTGATTGCTTTGAAAGATGTGATGATGCCAATTCATAGGAAAAGCTTTAAAGATGTTTATTTGGTTTATGAACTCATGGATACAGATCTTCATCAGATTATCAAGTCCCCTCAGCCACTTTCCCATGATCATTGCAAGTACTTTATCTATCAGTTGCTTTGCGGGTTGCAGCATCTTCATTCAGCTAACATTCTTCACCGGGACTTGAAGCCTGGGAACCTCCTTGTCAATGCTAACTGTGATCTTAAGATATGCGATTTTGGGTTGGCACGAACTAGCATGGGCCGTGATCAGTTCATGACTGAGTATGTTGTTACTCGCTGGTATCGGGCTCCAGAACTTCTTCTCTGCTGTGACAACTATGGGACTTCTATTGATGTATGGTCTGTGGGATGCATCTTTGCTGAGATTCTTGGTCGGCAACCTATCTTCCCAGGGACAGAGTGCCTTAACCAACTTAACTTAATTATCACCATTCTTGGTAGTCCAAAGGAAGCAGATGTTGAGTTCATCGACAATGTAAAGGCCAGAAATTATATTAAGTCAATGCCCTTCTCAAGGGGAATACGCCTTTCTCATCTTTACCCACAAGCTGAACCTTTAGCTATAGACTTGTTACAGAAGATGCTTGTGTTTGATCCAACTAAGAGAATTACTGTTGACGAAGCACTTCAACATCCATACATGTCGGGGCTATACGATCCGAAGTTCAATTCTTCTGTTGAGGTTCCTCTCAATCTCGACATTGACGACACACTTGGGGAGCCTAAGATCAGGGAAATGATGTTGAATGAGATGCTGTATTACCATCCTGAAGCTGTTTCAACATTTTCTTAA

>Csa1M024990.1

ATGGGGAGTGGAACTCTCGTGGACGGTGTTCGTCGCTGGTTTCAACGTCGTACTTCTTCTTCTTCTTCTTCTACTTCTAATTCTAATTCTAATTTTAGCTCTAATTCTGATTCTTCTGACCCTAATCTCAATTACCCAAATCTCCACAAGTTTGATTATGTTGATAATGGTGGTGTTAGTGGTGACCAATTGTTGAGTAGCGATTTACGCGCTCAATCGTCCATTGCCCACAAACGCAAACCTCTAAGGAAACAAACCCAACTCGGAGAAGGAGGGATTCTTGAGCAATTACCTGAGGAGGAGGACGACGATCTTGATTACTCTGCCTTGAAGCTCATTAAAGTTCCTAAACGGATCAATCACTTCAGAAATCCTCCTCCTCCTCTTCCTTCTGCTTTAATGGACTCTCACAAGAAGGGTGGGTTGGAAACTGAGTTTTTCACAGAGTATGGAGAGGCCAGCAGATATCAGGTTCAAGAAATAATCGGTAAAGGCAGTTATGGGGTTGTTGGTTCCGCTGTTGATACCCACACTGATGAGAAGGTTGCAATTAAGAAAATTAACGATGTTTTTGAGCACGTTTCTGATGCCACAAGGATTTTAAGAGAAATTAAACTCCTACGGCTGCTCCGTCATCCAGATATAGTAGAAATAAAGCACATTATGCTTCCTCCTTCACGGAGAGAATTTAGAGATATTTATGTTGTTTTTGAGTTGATGGAATCTGATCTCCACCAAGTAATTAAGGCCAATGATGATCTTACTCCTGAGCACCATCAGTTTTTCTTGTATCAGCTTCTTCGTGGTCTGAAATATATTCATACAGCAAATGTATTTCATCGTGATTTAAAGCCCAAAAATATTTTAGCTAATGCTGATTGCAAACTGAAAATATGTGATTTTGGACTTGCTCGTGTATCATTTAATGATGCACCATCTGCTATTTTCTGGACTGACTATGTTGCAACTCGGTGGTATCGTGCTCCCGAACTTTGTGGCTCTTTTTTCTCAAAATACACTCCAGCAATTGATATTTGGAGCATTGGATGCATTTTTGCGGAAATGCTTACTGGAAAGCCACTGTTTCCTGGGAAAAACGTGGTGCACCAACTGGATCTGATGACTGATGTGCTTGGCACACCTTCTTCTGAGTCCATTGCTAGGATTCGGAATGAGAAGGCAAGAAGATACCTTAGTAACATGAGGAGAAAGCAGCCTGTTCCTCTCACACAAAAGTTTCCCAATGCTGACCCCTTAGCTCTCCGATTGCTTCAACGCTTGCTTGCATTTGATCCCAAAGACCGTCCCACTGCCGAAGAGGCATTAGCTGATCCGTATTTTCAAGGTTTGGCTAACGTGGATCGAGAACCATCAACACAACCAATTTCAAAACTTGAGTTCGAATTTGAGAGAAGAAAGTTAACAAAAGATGATGTTAGAGAATTAATTTATCGAGAGATATTGGAGTATCATCCTCAGATGCTACAGGAATATCTCCGCAGTGGAGAACAAACTAGCTTCATGTACCCAAGTGGTGTTGATCGCTTCAAACGCCAGTTTGCTCATCTAGAGGAACATTATGGCAAGGGTGAAAGAAGCACTCCACTTCAAAGACAGCATGCTTCATTGCCCAGGGAAAGGATTCCTGCACCAAAAGATGAAGCTGGACAACACAATGATTTAGAGGGAAGAAATGTTGCTACATCTCTTCAGAGTCCTCCAAAGTCGCAAGGGGATGGTTCCGAAAATGCAAACGGTAACGAACAAAATGGACAAAACAAACCAAATTACAGTGCTCGTAGCTTGTTGAAGAGTGCCAGCATTAGTGCCTCTAAATGTATAGGTGTTAAACCAAGAAAAGACCTAGAGGAGGAACCAATTTCAGAGACGAATGACGAGGCAGTTGACGGAGTGTCTCATAAGATGTCAGCCTTGCATACCTGA

>Csa5M002030.1

ATGATTGAAAAGGAATTTTTCACTGAATATGGTGAAGCTACTCAATACGAGATTGAAGAAGTTGTTGGTAAAGGGAGCTATGGAGTTGTGGCATCTGCCATTGACACTCATTCTGGTGAGAAAGTTGCTATCAAGAAGATAAATAATGTGTTTGAGCATGTTTCTGATGCCACGCGGATTCTAAGAGAAATTAAACTTCTTCGGTTTCTTCGACATCCTGATATTGTCGATATAAAACATATAATGCTCCCTCCATCCCGAAGGGAGTTCAAAGATTTATACATTGTTTTTGAGTTGATGGAGTGTGATCTTCATCATGTACTAAAGACTAATGATGATCTCACTCCTCAGCATCATCAATTTTTCTTATATCAACTTCTTAGAGCATTGAAGTATATACATTCAGCTCATGTGTTCCATAGAGATTTGAAGCCGAAGAATATACTTGCAAATGCAGACTGCAAGCTGAAGATCTGTGACTTTGGACTTGCACGTGCATCTTTCAGTGATGCCCCGTCTGCTATATTTTGGACTGATTATGTCGCTACTCGATGGTACCGTGCTCCTGAACTTTGTGGTTCTTTTTTCTCAAAGTATACACCAGCTATTGATATTTGGAGCATAGGATGTATATTTGCTGAAATGTTGGGAAGCAAGCCTTTGTTCCCAGGGAAAAGTGTTGTCCATGAGCTGGATCTAATAACTGACTTACTTGGTACCCCTTCAGCTGAATGTATTGCTAAGATTCGTAATGAAAAGGCTAAAAGGTATTTGAGTGGCATGAGAAAAAAAGATCCAATACCTCTATCGAAAAAGTTTCCTAATGCAGACCCACTAGCTCTTCGTTTACTTGAACGTCTGCTTGCATTTGATCCTGATGATCGCCCTTCTGCTGAGGAGGCATTAGCTGATCCTTATTTCCATGGACTGGCTAATCTGAAGGATGAACCATCCAGGCAACCCATTTCAAAACTCGAGTTTGAGTTTGAAAAGAGAAAGTTGACCAAAGATGATGTCAGGGAACTTATATATAGAGAAATTTTGGAGTATCACCCTCAAATGCTTAAGGAATACCTTCAAGGCTCCGGGAGCCACTTTTTGTATCCCAGTGGAATTGACCGATTCAAGCGACAATTTGATCATCTTGAGGAACGTTCTGGTAAAGGTGAGAGAGGCAGTCCACTTCTAAGGAAGCATGCCTCCTTGCCTAGGGAGCGAATATATACGCTGGGATATGAAGATGATGATGATGAAAAACATAGAACAGGATACCGAAATGCAGCTTCAATTGAACGTGCAGCGGTTCATAGCCCACCTGCTTATCCATTAACTGCTCGGAACGATTGCAATAGTTATAACCTGTTGAGGAGTGCTAGTATAAGTTGTTCTAAATGGGTGGATTAA

>Csa5M002030.2

ATGATTGAAAAGGAATTTTTCACTGAATATGGTGAAGCTACTCAATACGAGATTGAAGAAGTTGTTGGTAAAGGGAGCTATGGAGTTGTGGCATCTGCCATTGACACTCATTCTGGTGAGAAAGTTGCTATCAAGAAGATAAATAATGTGTTTGAGCATGTTTCTGATGCCACGCGGATTCTAAGAGAAATTAAACTTCTTCGGTTTCTTCGACATCCTGATATTGTCGATATAAAACATATAATGCTCCCTCCATCCCGAAGGGAGTTCAAAGATTTATACATTGTTTTTGAGTTGATGGAGTGTGATCTTCATCATGTACTAAAGACTAATGATGATCTCACTCCTCAGCATCATCAATTTTTCTTATATCAACTTCTTAGAGCATTGAAGTATATACATTCAGCTCATGTGTTCCATAGAGATTTGAAGCCGAAGAATATACTTGCAAATGCAGACTGCAAGCTGAAGATCTGTGACTTTGGACTTGCACGTGCATCTTTCAGTGATGCCCCGTCTGCTATATTTTGGACTGATTATGTCGCTACTCGATGGTACCGTGCTCCTGAACTTTGTGGTTCTTTTTTCTCAAAGTATACACCAGCTATTGATATTTGGAGCATAGGATGTATATTTGCTGAAATGTTGGGAAGCAAGCCTTTGTTCCCAGGGAAAAGTGTTGTCCATGAGCTGGATCTAATAACTGACTTACTTGGTACCCCTTCAGCTGAATGTATTGCTAAGATTCGTAATGAAAAGGCTAAAAGGTATTTGAGTGGCATGAGAAAAAAAGATCCAATACCTCTATCGAAAAAGTTTCCTAATGCAGACCCACTAGCTCTTCGTTTACTTGAACGTCTGCTTGCATTTGATCCTGATGATCGCCCTTCTGCTGAGGAGGCATTAGCTGATCCTTATTTCCATGGACTGGCTAATCTGAAGGATGAACCATCCAGGCAACCCATTTCAAAACTCGAGTTTGAGTTTGAAAAGAGAAAGTTGACCAAAGATGATGTCAGGGAACTTATATATAGAGAAATTTTGGAGTATCACCCTCAAATGCTTAAGGAATACCTTCAAGGCTCCGGGAGCCACTTTTTGTATCCCAGTGGAATTGACCGATTCAAGCGACAATTTGATCATCTTGAGGAACGTTCTGGTAAAGGTGAGAGAGGCAGTCCACTTCTAAGGAAGCATGCCTCCTTGCCTAGGGAGCGAATATATACGCTGGGATATGAAGATGATGATGATGAAAAACATAGAACAGGATACCGAAATGCAGCTTCAATTGAACGTGCAGCGGTTCATAGCCCACCTGCTTATCCATTAACTGCTCGGAACGATTGCAATAGTTATAACCTGTTGAGGAGTGCTAGTATAAGTTGTTCTAAATGGGTGGATTAA

>Csa5M002030.3

ATGATTGAAAAGGAATTTTTCACTGAATATGGTGAAGCTACTCAATACGAGATTGAAGAAGTTGTTGGTAAAGGGAGCTATGGAGTTGTGGCATCTGCCATTGACACTCATTCTGCTCATGTGTTCCATAGAGATTTGAAGCCGAAGAATATACTTGCAAATGCAGACTGCAAGCTGAAGATCTGTGACTTTGGACTTGCACGTGCATCTTTCAGTGATGCCCCGTCTGCTATATTTTGGACTGATTATGTCGCTACTCGATGGTACCGTGCTCCTGAACTTTGTGGTTCTTTTTTCTCAAAGTATACACCAGCTATTGATATTTGGAGCATAGGATGTATATTTGCTGAAATGTTGGGAAGCAAGCCTTTGTTCCCAGGGAAAAGTGTTGTCCATGAGCTGGATCTAATAACTGACTTACTTGGTACCCCTTCAGCTGAATGTATTGCTAAGATTCGTAATGAAAAGGCTAAAAGGTATTTGAGTGGCATGAGAAAAAAAGATCCAATACCTCTATCGAAAAAGTTTCCTAATGCAGACCCACTAGCTCTTCGTTTACTTGAACGTCTGCTTGCATTTGATCCTGATGATCGCCCTTCTGCTGAGGAGGCATTAGCTGATCCTTATTTCCATGGACTGGCTAATCTGAAGGATGAACCATCCAGGCAACCCATTTCAAAACTCGAGTTTGAGTTTGAAAAGAGAAAGTTGACCAAAGATGATGTCAGGGAACTTATATATAGAGAAATTTTGGAGTATCACCCTCAAATGCTTAAGGAATACCTTCAAGGCTCCGGGAGCCACTTTTTGTATCCCAGTGGAATTGACCGATTCAAGCGACAATTTGATCATCTTGAGGAACGTTCTGGTAAAGGTGAGAGAGGCAGTCCACTTCTAAGGAAGCATGCCTCCTTGCCTAGGGAGCGAATATATACGCTGGGATATGAAGATGATGATGATGAAAAACATAGAACAGGATACCGAAATGCAGCTTCAATTGAACGTGCAGCGGTTCATAGCCCACCTGCTTATCCATTAACTGCTCGGAACGATTGCAATAGTTATAACCTGTTGAGGAGTGCTAGTATAAGTTGTTCTAAATGGGTGGATTAA

>Csa1M042720.1

ATGGGTGACAAGCAATCAGAATTTTTCACAGAGTATGGAGAAGCAAGCCGATACCAAATTCAAGAAGTTATTGGGAAAGGAAGCTACGGAATTGTTGGTTCTGCCATTGACACCCAGACCGGTGAAAGAGTTGCCATCAAGAAAATTAATGATGTGTTTGAGCATGTATCTGATGCCATACGGATCCTCAGAGAAATTAAGCTTCTTCGGATGCTTCATCATCCAAATATTGTAGAGATAAAGCATATTATGCTTCCTCCCTCACAACGAGAATTCAAAGATATATATCTTGTTTTTGAGTTAATGAAGTCTGATCTTCACCATGTAATTAAGACAAATAATGATCTTTCTCCTCGGCAGCATAAATTTTTTCTGTACCAGCTTCTTAGTGGCCTAAAATATATTCATACTGCAAATGTCCTTCATCGAGATTTGAAGCCAAAAAACATACTTGCTAATGCGGACTGCAGACTCAAGATATGTGATTTTGGACTTGCTCGTGTATCTTTTAGCGATGCACCATCTACTATTTTCTGGACAGATTATGTTGCAACTCGATGGTATCGTGCTCCTGAACTCTGTGGATCATTTTTCTCGAGATATACCCCTGCTATAGATATATGGAGTATCGGATGCATATTTGCAGAAATGCTGACAGGGAAACCTTTGTTTCCTGGAAAAAATGTGGTTCACCAATTAGATCTAATCACCGATCTGTTTGGCTCTCCTGAACCCGAGGCCATTGCAAAGATTCGTAATGAGAAGGCGAGAAGGTATCTTGGAAACATGCGTAAAAAACAACCAGTTCCATTCTCACGAAAATTTCCTAATGTTGACCCAATGGCACTTTGTTTACTGGAACGTCTCCTAGCATTTGATCCCAAATGTCGTCTAACAGCTGCAGAGGCCCTTGCTGATCCTTACTTCAACGGCATGGGGAAACCAGAACTTGAACCTTCCATTCAACCAATTTCAAAACTTGAGTTTGAGTTTGAAAGGAGGAAGTTATCAAAAGATGATGTTAGAGAGTTGATTTATGCAGAGATTTTAGAGTATCATCCCCAGATGCGTCAGGGTTGTCTACGTGGTGGAGACCATCCAACTACCTTTATGTATCCAAGTGGGGTTGATCGATTTAAGCTTCAGTTTGCACATCTGGAAGAGCACCACGGTAAAGGTGAAAGAAGAAGCCCACTTCAAAGGCAGAACATTTCTTTACCTCGGGAGCGGGTTAGGCCTACCGAGCAGAACAACACTGAGAATAGCATCGATTCCGAAAGAGGGAAAGATAAGAGTGCTCATCTCTTGAAAAGTGCAAGTATCAGTGCATCGAGATGTGTTGGGGTAATACCAAAGGAGACATATGAGGTGGAAGAAACTGAAGTGAAAAATGAAGCAGTGGATGGCATGTCTCAAAAGATTGCAGTCCTTCAAACTTAA

>Csa1M042720.2

ATGGGTGACAAGCAATCAGAATTTTTCACAGAGTATGGAGAAGCAAGCCGATACCAAATTCAAGAAGTTATTGGGAAAGGAAGCTACGGAATTGTTGGTTCTGCCATTGACACCCAGACCGGTGAAAGAGTTGCCATCAAGAAAATTAATGATGTGTTTGAGCATGTATCTGATGCCATACGGATCCTCAGAGAAATTAAGCTTCTTCGGATGCTTCATCATCCAAATATTGTAGAGATAAAGCATATTATGCTTCCTCCCTCACAACGAGAATTCAAAGATATATATCTTGTTTTTGAGTTAATGAAGTCTGATCTTCACCATGTAATTAAGACAAATAATGATCTTTCTCCTCGGCAGCATAAATTTTTTCTGTACCAGCTTCTTAGTGGCCTAAAATATATTCATACTGCAAATGTCCTTCATCGAGATTTGAAGCCAAAAAACATACTTGCTAATGCGGACTGCAGACTCAAGATATGTGATTTTGGACTTGCTCGTGTATCTTTTAGCGATGCACCATCTACTATTTTCTGGACAGATTATGTTGCAACTCGATGGTATCGTGCTCCTGAACTCTGTGGATCATTTTTCTCGAGATATACCCCTGCTATAGATATATGGAGTATCGGATGCATATTTGCAGAAATGCTGACAGGGAAACCTTTGTTTCCTGGAAAAAATGTGGTTCACCAATTAGATCTAATCACCGATCTGTTTGGCTCTCCTGAACCCGAGGCCATTGCAAAGATTCGTAATGAGAAGGCGAGAAGGTATCTTGGAAACATGCGTAAAAAACAACCAGTTCCATTCTCACGAAAATTTCCTAATGTTGACCCAATGGCACTTTGTTTACTGGAACGTCTCCTAGCATTTGATCCCAAATGTCGTCTAACAGCTGCAGAGGCCCTTGCTGATCCTTACTTCAACGGCATGGGGAAACCAGAACTTGAACCTTCCATTCAACCAATTTCAAAACTTGAGTTTGAGTTTGAAAGGAGGAAGTTATCAAAAGATGATGTTAGAGAGTTGATTTATGCAGAGATTTTAGAGTATCATCCCCAGATGCGTCAGGGTTGTCTACGTGGTGGAGACCATCCAACTACCTTTATGTATCCAAGTGGGGTTGATCGATTTAAGCTTCAGTTTGCACATCTGGAAGAGCACCACGGTAAAGGTGAAAGAAGAAGCCCACTTCAAAGGCAGAACATTTCTTTACCTCGGGAGCGGGTTAGGCCTACCGAGCAGAACAACACTGAGAATAGCATCGATTCCGAAAGAGGGAAAGATAAGAGTGCTCATCTCTTGAAAAGTGCAAGTATCAGTGCATCGAGATGTGTTGGGGTAATACCAAAGGAGACATATGAGTGGCGATTGCCGATCGGCTCCTCAAATTTTAAAACTGATACAAGGGACATGTGTGACTTTTTTCCATCTCGAACATGTATTCGAGTTATTTCTAAGCCTTTTTAA

>Csa1M077220.1

ATGGAGAATGATTCTTCTTCCGCCATGGATATCAAAGGTACTCCCACTTACGACTCCAAATACTTACTCTACAATGTTCTTGGCAGCTTCTTCGAGGTCTCCGCCAAATACTCCCCTTCAATTCAACCTGTTGGACGTGGTGCTTACGGCATTGTTTGTTGTACGACTAATTCCGAGACAAAAGAAGAGGTTGCGATTAAGAAGATTGGGAATGCTTTTGATAATAGGATTGATGCTAAGAGGACGCTTCGTGAGATTAAACTGCTTTGCCATATGGATCATGACAATATTATAAAAATCAAAGATATTATTCCACCTCCAGATAAGGAGAAATTCAATGATGTGTATATTGTGTATGAATTAATGGACACTGACTTACACCAGATCATTCGCTCTTCCCAGGCTTTGACAGACGATCACTGTCAGTACTTCTTATACCAATTACTGCGAGGTTTGAAGTATTTACATTCTGCAAATGTCTTGCACCGAGATCTTAAACCAAGCAACCTGCTCCTTAATGCAAATTGTGACCTCAAGATTTGTGACTTTGGGTTAGCAAGAACAACATCAGAGACAGATTTCATGACGGAATATGTTGTAACACGATGGTATCGAGCCCCTGAGCTACTGCTCAACACTTCTGAATACACTGCAGCGATAGATATTTGGTCTGTTGGATGTATTCTCATGGAAATTCTTAGGAGGGAGCCACTGTTCCCTGGTAAAGACTATGTGCAGCAATTGGGGCTTATAACTGAGTTGCTAGGTTCACCTGATGATTCAGATCTCGGGTTTCTTAGAAGTGATAACGCAAGGAAGTATGTTAAGCAACTTCCTCATTTCCCAAAACAACCGCTGATCGAAAAGTTCCCAGATCTTCCTCCACTGGCTGTTGATCTTGCAGAGAGGATGCTACTCTTTGATCCAAGCAAGAGAATAACTGTGGAGGAGGCCATGAATCACCCATATATAGTAAGTCTTCATGAGATCAATGAAGAACCAACCTGCCCTTCTCCTTTCAACTTCGATTTTGAACAGGCATCCTTGGATGAAGAAGACATAAAAGAACTTATATGGAGGGAGTCTATCAAGTTCAATCCGAATCACATTTAG

>Csa6M061230.1

ATGCAGCCCGATCAGAGAAGAAAGTCGTCCATAGATGTGGATTTCTTCACAGAATATGGTGAAGGGAGCAGGTATAGAATAGAGGAAGTAATTGGTAAAGGAAGTTATGGTGTTGTTTGCTCTGCGTATGACACTCATACTGGAGATAAAGTTGCAATTAAGAAAATCAATGATATCTTTGAGCACGTTTCTGATGCAACTCGTATTCTTCGTGAGATAAAACTTTTAAGGCTATTGAGACATCCAGACATCGTGGAGATAAAGCACATCCTACTACCTCCATCTAGAAGGGAATTCAAAGACATTTATGTCGTGTTTGAGCTCATGGAATCTGATCTACATCAAGTTATCAAAGCAAATGATGATTTGACTCCTGAACATTATCAGTTCTTTCTTTACCAGCTTCTTCGAGGCTTGAAATACATACACACAGCAAATGTCTTCCACCGGGACCTAAAACCAAAAAACATATTAGCAAATGCCGACTGCAAACTCAAGATCTGTGACTTTGGTCTAGCACGAGTTGCTTTTAATGATACTCCTACTGCTATTTTCTGGACTGACTATGTAGCAACAAGGTGGTACAGAGCTCCTGAACTCTGTGGTTCATTTTTCTCAAAGTATACACCAGCAATTGATATATGGAGCATTGGCTGCATCTTTGCGGAGCTTTTAACTGGAAAACCACTTTTCCCTGGAAAGAATGTTGTCCACCAATTAGATTTGATGACGGACTTTTTGGGAACCCCAAATGCAGAAGCCATTGCCAGGGTACGAAACGAGAAAGCTCGAAGATACTTGAGCAGTATGCGAAAGAAGAAGCCTGTTCCTTTCTCCCAGAAATTCCCTCATGCAGATCCACTTGCACTTCGCTTGTTGGAAAGAATGTTGGCTTTTGAGCCAAAGGATCGACCTACAGCAGAGGAGGCCCTTGCAGATCCATATTTTAAAGGCTTAGCGAAGGTTGAGAGAGAGCCATCTGCTCAACCCGTTACTAAGATGGAATTTGAATTTGAAAGACGAAGGATAACTAAGGAAGATGTCCGGGAGCTTATTTACCGTGAGATTCTCGAGTACCATCCAAAAATGTTGAAGGAGTTCTTAGATGGATCAGAACCTACCGGTTTCATGTATCCAAGTGCGGTTGACCATTTTAAGAAGCAATTTGCGTTCCTTGAGGAACACTATGGAAATGGTGCACCTGTAGCTCCTCCTGAAAGACAACATGCATCCTTGCCTAGGCCATGTGTTTTATACTCAGATAACATGGTGCAGAACCCAGCCCAAGTTGCAAATGACCTATCCAAATGTTCCATCAAAGAAGTTGAGAGGCCGCCAGTGGATAGGACTTGCAATATTCCTTTGGCTAGAGTTCCTATTCAAGTTCCTCAATCTATTCAAGCAGGCAATGGTGCAAGGCCTGGAAAAGTTGTTGGCTCTGTGTTGCGATACAACTGTGGAGCAGCAGCAGCGGCAGTGGCACCTGAAGTTCTTGAACAGCGGAGAATGACCAGAAACCCTTCCATTCCTCCACAGTACGCCGGTAATAACTGTTCGTATACGAGGAGAAACTCATCCTGTAAAAATGAAAGAGCTGATGAAGAAGCCATTGAAGGTCCAAATGGGTTGCAGCCTAAACCTCAGTACATAGCTAGAAAAGTTGCTGCTGCCCAAGGTGGACCAGGAAATAACTGGTACTGA

>Csa4M082320.1

ATGCCACAAGATCATCCAAAGAAGGAAGCCAAAGAAGTAAACTTTTTTACTGAATATGGGGATGCTAACAGATATAAGATTCTTGAAGTTGTTGGAAAGGGAAGTTATGGAGTTGTTTGTTCTGCTATTGACATGCAAACTGCGGAGAAAGTTGCAATAAAGAGAATTCATGATATTTTTGATCACGCATCTGATGCTATTCGAATCCTTCGTGAAGTTAAGTTGCTTAGATTGTTGCGGCATCCTGATATTGTTGACATCAAGCGTATTATGTTACCACCTTCTAAAAAGGAATTTAGGGACATTTATGTGGTTTTTGAGCTCATGGAATCTGATCTTCATCAAGTTATTAAAGCAAATGATGACTTGACGCGTGAACATCATCAATTCTTTCTCTATCAAATGTTACGTGCATTGAAATTCATGCATACAGCGAATGTGTATCATAGAGATCTGAAGCCAAAGAATATATTAGCAAATGCCAATTGCAAGCTTAAAATTTGTGATTTTGGACTTGCAAGAGTTGCTTTCAGTGATACCCCAACTACTGTATTTTGGACAGACTATGTTGCTACTAGATGGTACAGAGCTCCGGAGTTGTGTGGATCGTTCTGTTCCAAGTACACTCCTGCTATAGATATTTGGAGTGTTGGCTGCATATTTGCTGAGGTTTTGATGGGGAAGCCACTATTTCCGGGTAAAAGTGTTGCACATCAGTTGGATTTGATTACTGATCTTCTTGGGACCCCCTCAATGGAAACCATTGCAGGAGTTCGGAATGAAAAGGTCAGAAAATATTTGACAGAAATGAAAAAGAAATCTCCAGTGCCATTTTCACAAAGATTTCCCAAGGTGGATCCTACAGCCATCCGCTTATTGGAAAGACTATTAGCATTTAATCCAAAGGATCGACCATCTGCTGTGGAGGCTCTGGCTGATCCTTACTTTAAAGGCCTTGCCAAAGTTGAAAGAGAGCCTTCTTGTCAGCCAATCTCAAGATCGGAATTTGAGTTTGAGAGACGGAAATTAACAAAGGATGATGTTAGGGAATTGTTGTACAGAGAAATATTAGAATATCATCCTCAAATTCGTGACGATTATTTGAATGGAACTGAGACTACAAAGCTACACTATCCTAGTGTTACAGGTCATTTTAAAAGCCAATTCACCTTCCACAAGGAGAACAATGGTAAAAGCGCGCCTGTTTTACCTCTAGAACGGAAACACTTCTCACTGCCACGGTCCACTGTTTGCACAAATTTGGTTTCACCTGATCATGAACCCGTTCGGCGAAATCCCAAAGTTTGTAATAATAGTATGGGATTACCAGATAGAACATTTGGGAATCCATCAAAGGCTCATCATCCACCTAAAGTGCCTACTGGACGAGTTGCTGGATCTATTCTCCCATATGAGCACCGAAACATAAAAGATGTTTATTCAAAGCTAACGTCACAAATTAGAAGTTTGGATTTCTGA

>Csa4M082320.2

ATGCCACAAGATCATCCAAAGAAGGAAGCCAAAGAAGTAAACTTTTTTACTGAATATGGGGATGCTAACAGATATAAGATTCTTGAAGTTGTTGGAAAGGGAAGTTATGGAGTTGTTTGTTCTGCTATTGACATGCAAACTGCGGAGAAAGTTGCAATAAAGAGAATTCATGATATTTTTGATCACGCATCTGATGCTATTCGAATCCTTCGTGAAGTTAAGTTGCTTAGATTGTTGCGGCATCCTGATATTGTTGACATCAAGCGTATTATGTTACCACCTTCTAAAAAGGAATTTAGGGACATTTATGTGGTTTTTGAGCTCATGGAATCTGATCTTCATCAAGTTATTAAAGCAAATGATGACTTGACGCGTGAACATCATCAATTCTTTCTCTATCAAATGTTACGTGCATTGAAATTCATGCATACAGCGAATGTGTATCATAGAGATCTGAAGCCAAAGAATATATTAGCAAATGCCAATTGCAAGCTTAAAATTTGTGATTTTGGACTTGCAAGAGTTGCTTTCAGTGATACCCCAACTACTGTATTTTGGACAGACTATGTTGCTACTAGATGGTACAGAGCTCCGGAGTTGTGTGGATCGTTCTGTTCCAAGTACACTCCTGCTATAGATATTTGGAGTGTTGGCTGCATATTTGCTGAGGTTTTGATGGGGAAGCCACTATTTCCGGGTAAAAGTGTTGCACATCAGTTGGATTTGATTACTGATCTTCTTGGGACCCCCTCAATGGAAACCATTGCAGGAGTTCGGAATGAAAAGGTCAGAAAATATTTGACAGAAATGAAAAAGAAATCTCCAGTGCCATTTTCACAAAGATTTCCCAAGGTGGATCCTACAGCCATCCGCTTATTGGAAAGACTATTAGCATTTAATCCAAAGGATCGACCATCTGCTGTGGAGGCTCTGGCTGATCCTTACTTTAAAGGCCTTGCCAAAGTTGAAAGAGAGCCTTCTTGTCAGCCAATCTCAAGATCGGAATTTGAGTTTGAGAGACGGAAATTAACAAAGGATGATGTTAGGGAATTGTTGTACAGAGAAATATTAGAATATCATCCTCAAATTCGTGACGATTATTTGAATGGAACTGAGACTACAAAGCTACACTATCCTAGTGTTACAGGTCATTTTAAAAGCCAATTCACCTTCCACAAGGAGAACAATGGTAAAAGCGCGCCTGTTTTACCTCTAGAACGGAAACACTTCTCACTGCCACGGTCCACTGTTTGCACAAATTTGGTTTCACCTGATCATGAACCCGTTCGGCGAAATCCCAAAGTTTGTAATAATAGTATGGGATTACCAGATAGAACATTTGGGAATCCATCAAAGGCTCATCATCCACCTAAAGTGCCTACTGGTATGAACATATTTCTCATGGTTTTCCCCTCAAAGTTTCTCTCTTTTCTTTTCAATGGGCAAATTCTGTCTAGAGTATCTTTTGAGCGAGAATCTTGTAAGCTTGTATAA

>Csa6M179480.1

ATGCAGCAAGATCAAAGCAAAAAGAATTCAACCGAAGTGGAGTTCTTTTCTGATTATGGTGATGCCAGTAGATACAAAATTCAGGAAGTAATTGGCAAAGGAAGCTATGGGGTTGTCTGTTCTGCAATTGACACCCGCACCGGTGACAAGGTTGCAATAAAGAAGATTCACAATATCTTTGAGCATATATCTGATGCGGTTAGAATTCTTCGTGAGATAAAGCTGCTTAGACTTCTTCGCCATCCTGATATTGTTGAAATTAAACACATTATGCTGCCACCTTCAAGGAGGGATTTCAAAGACATCTATGTTGTTTTTGAGCTGATGGAATCAGATCTCCACCAAGTTATTAAAGCAAACGATGATCTGACTCGAGAACATTATCAGTTTTTCCTTTACCAACTTCTTCGTGCATTAAAATATATCCATACAGCTAATGTCTACCACCGTGATTTAAAACCAAAGAATATATTAGCAAATGCAAATTGTAAACTTAAAATTTGTGATTTTGGATTGGCAAGAGTTGCGTTCAGTGACACACCAACAACAATTTTCTGGACGGATTATGTTGCTACTAGATGGTACCGAGCTCCGGAGCTATGTGGTTCATTTTTCTCGAAGTATACACCTGCAATTGATATATGGAGTATTGGTTGCATATTTGCCGAAGTATTGATGGGGAAACCACTTTTTCCTGGTAAAAACGTTGTACACCAGCTCGATTTAATGACGGATCTCCTTGGAACTCCTTCATTAGATACAATTTCCAGGGTACGTAATGACAAGGCTAGAAGATATTTAACTACTATGAGAAAAAAGCAGCCGGTGCCATTTTCTCAAAAGTTCCCTAATGCAGATCCTTTAGCACTGAGGCTACTGGAAAGACTGCTTGCTTTTGATCCAAAAGATAGACCAACTGCTGAAGAGGCACTGGCTGATCCATACTTCAAGGGGTTGGCAAAAATTGAGAGGGAACCTTCCTGCCAGCCAATCACAAAAATGGAATTTGAATTTGAGAGACGGAGGGTCACTAAAGAAGACATTCAGGAGCTAATCTTCCGGGAGATACTAGAATATCATCCTCAGCTACTAAAAGACTACATGAATGGGACTGAGAGAACAAATTTTCTATATCCAAGTGCTGTCGATCAATTTAGAAAGCAGTTTGCTCATCTGGAGGAAAATGGTGGTAAAAGTGCACCGGTAATTCCTCTTGACAGAAAGCATGTGTCCCTTCCAAGGTCAACAGTTGTTCACTCGAACCCAGTCTATTCTAAGGACCAAGTAAACAATATACCTCTCCAAGATGGCAAGATCTCTGAAGACGCATACAGCAAAAATTCCCGAGATAGTGAAGGACGGCTAACAAATATATCGAGGACCATGCAGGCACCACAAAAAATCCCGTTTGCAGCTAAGCCTGGAAGAGTGGTTGGACCGGTTATAGCAGACGAGAATGGGAGGCTAGTTAAGGAACCATATGATCCAAGAACGTTGATCAGAGGTGCCATTCTTCCTCCTGCATATCACTACCACCAAAAACCTATTGTTGGAAATCAAGAAAGATCTGCAGCAGAAACAAAACTTGATATTTCTTTAAGGGCCGCTAAGCAAGCTTCCCAGTGCGGCATGGCTTCGAAATTAGGATCAGATATAGCCATCAGCATTGACTCAAACCCTTTTTACATGACACGTGCCGGAGTGAACAAGGTCGAACTCAAGGACCAAATATCCATCAATGCAAACTTTCTGCAGGCCAAAGCCGCCCAATACGGTGGTCTTAGTGCAGCAACAGCCACCACCACATCGGTTGCTCACAGAAAGGTGGTTGCTGGTCAGTTTAACATGACAAAGATGTACTAG

>Csa6M423420.1

ATGCAGACCGATCACCGTAAGAAGAATTCTGCAGAATTGGACTTTTTCTCTGAATATGGTGATGCCAACAGATTCAAAGTCCGCGAAGTTATTGGAAAGGGGAGTTATGGTGTGGTTTGTTCAGCTGTTGACACTCTCACTAATGAAAAAGTGGCAATAAAGAAGATACATGATATTTTTGAACATGTATCTGATGCTGCCCGGATTCTTCGTGAGATAAAGCTCCTCAGGCTTCTGCGCCATCCCGATATTGTTGAAATTAAACACATTATGTTACCACCTTCTCGTAGGGGGTTCAAAGATATTTTTGTTGTGTTTGAGTTGATGGAATCAGATTTGCATCAAGTCATCAAGGCCAATGATGATTTAACAAAAGAGCACTATCAGTTTTTCCTCTACCAGCTACTACGTGCACTGAAATTTATTCACACAGCAAATGTCTACCATCGGGATTTAAAACCAAAGAATATATTGGCAAATGCGAATTGCAAACTTAAAATCTGTGATTTTGGATTGGCTAGAGTTGCTTTCAGTGACACACCGACAACAATATTTTGGACGGACTATGTTGCTACTAGATGGTATAGGGCTCCAGAGCTATGTGGTTCTTTCTTCTCTAAGTATACTCCTGCCATTGACATATGGAGTATTGGCTGCATATTTGCTGAAGTACTGACCGGGAAACCACTTTTTCCTGGTAAAAATATTGTTCATCAGCTTGATTTGATGACAGATCTGCTTGGAACACCTTCATTAGATACCATTTCTCGGGTAAGAAATGAAAAGGCCAGGAGGTACTTAACTAGTATGAGGAAGAAGCAGCCAATACCCTTTTCTCAGAAGTTCCCAAATGCTGATCCGCTAGCTCTACAATTGCTACAACGGTTGCTTGCCTTTGATCCAAAGGATCGGCCTACTGCTGAAGAGGCGTTGGCAGATCCTTACTTCAAGGGGCTGGCTAAAGTTGAGAGGGAACCTTCTTGTCAGCCAATCTCAAAGGTGGAGTTTGAATTTGAGAGGAGAAAAGTCACAAAGGATGACATTCGCGAGTTGATATTCCTTGAGATACTTGAATACCATCCTCAACTGCTGAAAGACTACTTAAATGGAACCGAGAGATCAAATTTTCTTTATCCAAGTGCACTAGATCAGTTCAAAAAGCAATTTGCTCATCTTGAAGATAATGGAGGGAAAAGTGGACCAGTTTATCCTCTAGAAAGAAAACATGCATCTCTTCCTAGGTCTTCAGTGCAGTCAAATACCATTCCTCCTAAAGTAACATCAAATATTGTTTCCTTTAAAGATCGATATGCGCCAACAGCTCCGTTCGGCAGTCAGCTTTACAAAGATTCGGCAGCACAGAGGATTGCTGCTGCTCAAGCCAAGCCTGGAAGAATCTCAGGCCCGGTTGTGCCATATGACAGTGGAAGTATCATTAAAGATGCTTACGACCCACGGATGTTAATTAGAAGTGCCTTCCCTTCTCATGCTATCCATCCAACATATTATTACCAGCAATCTTGCGGCCAAAATGAAGAAAGATCAGCAACAGGGGCTGAGAAGGACACGTCCATGCAATGCAAACAATCCCCTCAATGTGGAATGGCTGCCAAATTAGCAGGAGATACAGCTGCTGCCACTGGTGCTTTCTCAAATTCTTTCTTTATGGCACGTGTAGGCATGCCCAAGATGGGAAACAATGACCGTGCTGCACATTTGCAGGTAAGAGCCCAATATGATGCTGGAGCTGTTGCTGCTGCAACTACCACTACCCATAGAAACACTGGTGTGGTCGATTATGGCATGACCAGAATGTGTTAG

>Csa1M589750.1

ATGAGGAAAGGAGGCTTCAGCAACAACCTCAATCTCAAGCTCAATCTTCCTAAAGAAGACCAATCCATAGCGACGTTCCTAACGCAGAGTGGCACGTTTAAAGATGGTGATCTGCTTGTAAACAGAGATGGGGTTCGGATTGTTTCTCAAAGCGAAGTTGAAGCACCACCTCCGATTAAGCCTACAGATGATCAGTTGAGTTTAGCGGACATAGACATTATTAAAGTCATTGGAAAAGGAAATGGTGGTACTGTGCAATTAGTTCAGCACAAATGGACTGCTCAGTTTTTTGCATTGAAGGTAATTCAGATGAAAATTGAAGAGTCTCACCGCAAGCAGATTGCACAAGAACTGAAAATCAATCAATCAGCGCAGTGCCCTTATGTTGTTGTCTGTTACCAGTCTTTCTATGATAATGGATCAATATATATCATCCTAGAGTACATGGATGGAGGATCTTTAGCAGATTTTTTAAAAAAGGTTAAAAAAATTGAAGAACCATATCTTGCTGCCCTTTGTAAGCAGGTACTGAAGGGGTTGTCTTACCTTCACCATGAAAGACACATCATCCATAGGGACTTGAAGCCTTCAAATTTATTAATAAACCATAGAGGGGAAGTCAAGATTACTGACTTTGGTGTGAGTGCAATTATGGAAAACACATATGAAGAGGCTAATACTTTTGTTGGCACGTATAACTATATGTCCCCAGAGAGAATTGTGGGAGAGGGATATGACAATAAAAGTGACATTTGGAGCTTGGGTCTGATATTACTTGAGTGTGCAACCGGAAAGTTTCCTTATTCTCCACCCGGGCAAGATGGAGGATGGGTTAATTTTTATGAGCTTATGGAAGCCATTGTTGAAGGCGAACCTCCTTCTGCTCCGGCTGACCAATTTACTCCTGAATTCTGTTCATTTATTTCTGCATGTGTGCAAACAGACCCAAAGAATAGACTGTCAGCACGTGAACTTTTGGAACATCCTTTCATCAAGATGTATGAAGATAAGGATATTGATCTATCATCTTACTTCAATGATGCAGGATCTCCACTTGCAACTTTCTAA

>Csa2M000340.1

ATGAAGAAGGACACCTCCATGAATTCTAACTTAAAGCTTATTCTACCTCCTCCCGACGAACTTACCTTTGGCTTCATAACTCGAAGCGGTACTTTCACCGATGGCGATTTGCTTGTTAATAAGGACGGCGTGCGGATTGTTTCTCAAACGGACGACGAAAGTCCTCCACCAATAAAGCCCTCAGACAACCATTTGTCTTTAGCAGATTTAGACTCGATAAAAGTCATTGGGAAAGGAAATGGTGGAATTGTTCAATTAGTCCGACATAAATGGACCAACCAGTTTTTTGCTCTAAAGGTGATTCAAATGAATGCCGAGGAATCTTATTGTCGGTTGGTTGCTAAGGAGCTAAAAATTAATCAGTTGGCACAAAACCCTTACATCGTTGTCTGTTATCAAATCTTCTATGACAATGGTGCCATTTTCATTATCTTGGAATACATGGATGGTGGATCGCTTGCAGATCTTCTGAAAAAAGTTGAAACAGTCCTTGAGCCGTATCTTGCAGCCATTTGTTACCAGGTACTAAACGGGTTAATATACCTTCACCATGAAAAACATGTCATCCACAGAGACCTTAAGCCTTCAAATTTATTGATAAATCATAGAGGCGAAGTTAAGATTACTGACTTTGGTGTAAGCGCTATACTGGCGAATACGGCCGATCAAGCAAATTCTTTTGTTGGCACATATGCTTACATGTCTCCGGAGAGACTCAATGGGGACAAATACGACAACAAAAGTGACATATGGAGTTTGGGTTTGATTTTGCTTGAATGTGCGACGGGTCAATTTCCATATGCTCCACCAGATAAAGAAAAAGGATGGGAAGGCTTTTTTGATGTTATGGTGGCCGTAGTAGAACTAGCATCACCTTCTGCGCCCGAACAATTTTCTCCAGAGTTTTGCTCTTTCATTTCTTCATGCTTACAGAAAGACCCACAGAAGAGAAGCTCTGCACGTGAACTTCTGGTGCATCCTTTCATCAAAAAGTTCGAGAACTTCGATGTAGATCTCGCAGCTTACTTCAAAGATGCTGGTTCTCCATTGGCAACCTTCTAA

>Csa3M839800.1

ATGGCGGGTCTTGAGGAACTCAAGAAGAAACTTACTCCATTGTTTGATGCTGAAAAGGGGCTTTCTATGGACTCCCCAGTGGACCCGTCTGATTCTTATACGTTTTCAGACAATGGGACTGTTAATTTGCTAAGCCGATCGTATGGGGTTTATAATTTCAATGAGCTAGGGTTACAAAAATGCACATCTTGGCTGGCTGATGATTCGGGGAGCAGTGAGAGGACATATCGATGTGCTTCCCGTGAAATGAGGATATTTGGAGCCATCGGTAGTGGTGCTAGCAGTGTTGTTCAGAGAGCTATCCACATTCCTGCTCATCGAATCATGGCACTAAAGAAGATTAATATCTTTGAAAAGGAGAAAAGACAGCAGCTTCTTACTGAAATACGGACACTATGTGAAGCGCCCTGTTCTGAGGGTCTTGTGGAGTTTCATGGAGCATTTTATACCCCTGATTCTGGGCAAATAAGCATCGCTTTGGAATACATGGATGGAGGGTCATTGGCAGATGTCCTGCGTTTGAAAAAATGTATACCTGAGCCTGTCCTTTCTACTATGTTTCAGAAGCTTCTTCGAGGCTTAAGTTATCTGCATGGAGTAAGACATTTGGTTCACAGAGATATAAAGCCAGCTAATTTACTTGTAAATCTAAAGGGTGAAGCAAAGATTACGGATTTTGGCATAAGTGCTGGCTTAGAGAACTCGATGGCAATGTGTGCTACTTTCGTTGGAACTGTCACGTACATGTCACCGGAGCGAATTCGAAACGAGAGCTATTCTTATCCAGCTGACATTTGGAGCCTTGGGCTTGCACTCTTTGAGTGTGGTACTGGTGAATTTCCATATTCAGCTACTGAAGGTCTAGTGAACCTTATGTTACAGATTTTGGATGACCCGTCTCCATCGCCATCAAAACATAAGTTTTCGTCGGAGTTTTGCTCGTTTGTCGATGCCTGCTTGCAAAAAGATGCAGATGCTAGGCCAACAGCAGAGCAGCTGCTTTCACACCCATTTATCAAAAAATATGAGAATGAACAAGTAGACTTAGCAGCATTTGTCCAGAATGTGTTTGATCCAACACAGAGGATGAAGGACTTGGCAGATATGTTGACAATTCATTATTACTTGCTTTTTGATGGACCTGATGACTTCTGGCACCATACAAAAGCTTTATTCCATGAAAGCTCAACTTTAAGTTTCTCAGGGAAGCAATTTTCTGGTCCAAATGATATCTTTGGCAAACTATCAGAGATTCGAAGTACGTTAGCAGGAGATTGGCCTCTTGAAAAACTTGTTCACGTTGTCGAGAAACTCCAATGTCGAGCCCACGGTCGGGACGGAGTTGCCATAAGGGTGTCAGGATCCTTCATCCTTGGGAACCAGTTCCTCATATGTGGAGACGGTGTACAAGTAGAGGGACTGCCAAATTTTAAGGATCTCTCCATTGACATGGAAAGCAAGAAAATGGGATCCTTCCGGGAGCAGTTCATCATTGAACCGAGCAATCTCATCGGTCGTTATTTCATTGCTAAACAAGAGCTTTATATAATCCAATAG

>Csa3M651720.1

ATGAGACCTTTCCAGCCGCCTCCAGCTACAAATCCTCCCACCGATCGTACTCGTCGCCGTCCCCATCTAAATCTCCATCTTCCTCAACGTGACAACACATCTTTGGCTGTTCCTCTTCCTTTGCCTCCTACCTCCTCCAACTCTGCCCCTCCTCCTTCCACCAGTCAACTCCACAACGCCAATCGCCCCCCTGACCCTCTTCCTCCCCAACGTCACCCTTTCACCCTTTCTGACTTTGAGCGTGTCAGCCGAATCGGAAGTGGATGTGGAGGTACCGTTTACAAAGTCCTTCACCGTCCCACCGGTCATGTTTATGCTCTCAAGGTCATTTACGGAAACCATGAAGATGCGGTTAGGCTTCAGATGTGCCGCGAGGTCGAGATCCTTCGTGACGTTGACAATCCCTATGTTGTTAAGTGCCACGATATGTTTGATCATAACGGTGAAATCCAGGTTCTTCTTGAGTATATGGATCGGGGTTCTTTGGAAGGAACACACATCCCTCAAGAACATCAACTCTCTGATTTGGCTCGCCAGATTCTTAGCGGTCTTGCTTATCTTCATAGCCGACGTATCGTTCATCGCGATATCAAACCCTCCAATCTTCTCATCAATTCTCGAAGGCAGGTTAAGATCGCTGATTTTGGCGTCGGTCGAATCTTGGAACAGACTATGGACCCTTGTAATTCCTCGGTTGGGACTATTGCGTATATGAGCCCTGAGAGAATCAATTCCGATCTAAATCAGGGTCAGTACAATGGTTATGCAGGGGATATTTGGAGCTTTGGGGTTAGCATATTGGAATTCTATCTGGGTCGCTTTCCTCTTGCTGTTGAGAGGCCTGGGGATTGGGCTAGCTTGATGTGTGCTATATGTATGGCTCAGCCACCGGAGGCCCCGGCCACTGCTTCACCGGAGTTTCGTCACTTTATTGCTTGTTGTTTGCAAAGAGAAGCTCGGAAGAGATGGACTGCAGCTGCATTACTGGAGCATGCGTTCATAACAAGGAAAAATGGAGCAAGTCAGTACCAGAACAAGCAAGCTCATCATCAGAATCTTCGTCAACTTTTACCTCCTCCGCCTCTTCATCCTCCCAGCCTTTCTTGA

>Csa2M000780.1

ATGAAGACCAAGACGCCATTGAAGCAGCTCAAGCTCTCTGTTCCCGTTCAAGAAACTTCCATCCGTTCTTTCCTGACTGCAAGTGGAACATTTCATGACGGCAATCTACTCCTGAATCAGAAAGGAATGCGGCTTATATCTGAAGAGAAAGAATCGCAGACTACAGATTCTAAGGAGCTTGATGTTGATTTCTCGTTGGAAGATCTAGAGACTGTGAAAGTTATTGGGAAAGGAAGTGGAGGTGTAGTACAACTTGTTCGACACAAATGGGTTGGAAAACTTTTTGCCTTAAAGGTAATCCAGATGAACATACAAGAGGATATCCGTAAACAGATAGTACAGGAACTGAAAATAAATCAAGCAGCACAATGTTCACATATTGTGGTCTGCTATCATTCTTTCTACCACAACGGTGCCATTTCTCTTGTGCTAGAATACATGGATCGTGGTTCTTTAGCCGATGTAGTCAGACAAGTCAAAACAATTCTTGAACCATATCTCGCAGTTGTTTGTAAACAGGTTTTACAAGGTCTGGTATACTTACATCATGAGCGACATGTTATACACAGAGACATCAAGCCATCCAATCTACTTGTGAACCATAAAGGTGAGGTCAAGATCACTGATTTTGGAGTAAGTGCAATGCTAGCAAGTTCTATGGGTCAGAGAGATACATTTGTAGGGACTTACAATTACATGTCGCCTGAACGAATTAGCGGAGGAACATATGACTACAGCAGTGATATATGGAGTTTGGGTTTGGTAGTACTTGAATGTGCAATTGGAAGGTTCCCTTATTTGCAATCTGAAGAGCAGCAAAGTTGGCCAAGCTTTTATGAACTCCTTGAGGCAATTGTAGCAAAGCCACCGCCTTCAGCTCCACCGGATCAATTCTCCCCCGAATTCTGTTCCTTCGTTTCTGCTTGCATAAAGAAGGACCCAAAAGAAAGATCATCATCATTAGATCTTCTGAATCATCCTTTCATCAAGAAATTCGAAGATAAAGACATCGACGTCGGGATTCTCGTCGCCAGCTTGGATCCACCGGTAAGTTTTCCCAGACAACAACAACAATAA

>Csa1M042980.1

ATGGCTTTGGTCCGTGATCGCCGCCACCTCAACCTCCGTCTCCCCGATCTCTCCGACTGTCGTCCTCGCTTCCCCCTTCCACTCCCTCCTTCCTCTGCTCCCCCTGCTCCTGCTGCTCCCTCCGCCATCTCCTCCTCCGACCTCGACAAGCTCCAAGTCCTCGGTCACGGTAACGGCGGCACCGTCTACAAAGTCCGTCATAAACGCACCTCCACTACTTACGCACTCAAGGTCGTCCACGGCGACTGCGACCCCACCGTTCGTCGCCAAGTTTTCAGAGAGATGGAGATTCTTCGTCGGACTGATTCTCCTTATGTTGTTCAGTGTCATGGAATCTTTGAGAAACCTTCTGGAGATGTAACGATTTTGATGGAGTATATGGATCTTGGATCTCTTGATTCGTTATTGAAGAAGAACTCGACTTTGTCTGAAGCGACGCTGGCTCATGTATCGCGTCAGGTTCTTAATGGTCTTCATTATCTTCACTCTCATAAAATCATTCATCGTGATATTAAACCGTCGAATCTGTTGGTGAATAAGAATATGGAGGTTAAGATTGCCGATTTTGGAGTTAGTAAAATTATGTGCAGGACTTTGGATGCTTGCAATTCTTACGTCGGAACTTGTGCTTATATGAGTCCAGAGCGGTTCGATCCCGAGACGTACGGCGGAAATTACAACGGTTATGCCGGAGATATTTGGAGTTTAGGCCTTACTCTTCTAGAACTTTACTTAGGTCATTTCCCGTTTCTTCCGGCCGGACAGAGACCGGATTGGGCGACTCTGATGTGTGCGATTTGCTTCGGTGAACCACCGAAGCTGCCAGAGGATGCATCGGAGGAGTTTCGGAGCTTTGTTGAGTGTTGTTTGCAGAAGGAATCGAGTAAAAGATGGACGGCGGCACAATTGTTGACGCATCCGTTTGTATGCAGGGAATCATCGAGATCATCGGATAATCGATGA

>Csa2M021750.1

ATGCAAGATATCTTCGCTTCTGTTCGCCGATCATTGGTCTTTCGTCCTCCTCTCGACAACGATGATTCCCATTCCCCTGCAATTGGTGTTGGAGCCCTAGTTGATAAGATCAACTCCAGCATCCGCAAATCCAGAGTCTTCTCTAGACACTCCCCTTCCTCTTCCTCTCTCCCTCCCATTCCTAAAGACACCGACCCTCCCATTCGATGGCGCAAAGGCGAATTGATTGGCTGTGGCGCTTTTGGTCGCGTTTATATGGGCATGAATCTTGGCTCTGGAGAGCTTCTTGCTGTCAAACAGGTTTTGATTGCTGCAAATGGTGCTTCGAAGGAGAAAGCGCAGGCTCATGTTCAGGAGCTTGAGGAAGAAGTGAAACTTCTGAAGGATCTTTCTCATCCAAATATTGTTAGATACTTGGGCACAGTCAGAGAGGATGACTCTTTAAATATATTATTGGAATTTGTCCCTGGTGGATCGATAGCATCACTTCTGGGGAAATTTGGAGCCTTCCCTGAAGCAGTTTTAAGAACATATACAAAACAGTTATTATTGGGATTGGAGTATTTACACAAGAACGGTATCATGCACAGGGACATTAAGGGGGCAAATATCCTTGTAGATAACAAGGGATGCATTAAGCTTGCTGATTTTGGGGCTTCGAAACAGGTTGTCGAGCTGGCTACAATTTCAGGAGCAAAGTCTATGAAGGGTACTCCATATTGGATGGCTCCTGAAGTAATTCTGCAGACTGGTCATAGTTTCTCTGCTGACATATGGAGTGTTGGATGCACCTTTATTGAGATGGCTACAGGAAAGCCTCCTTGGAGCCAACAGTATCAAGAGGTTGCTGCTCTTTTTCATATAGGGACGACAAAGTCTCATCCACCAATCCCTGAGCAGCTTTCGGTTGAAGCTAAAGATTTTCTGTTGAAATGTTTGCAGAAGGAACCAAACTTAAGACCAACTGCCTCTGAACTCTTGAAGCATCCTTTTGTTATAGGGGAGGAGACACAATCTCAACTTATGTCACGTGATGCATGCACGGAGCCCCTGGAAACCCATTCACCACAATGTACTTCAGAACTTGAAATGAGTAAAACTCCTACACATCCTGGATCAAGCGATATTTGTAATTTGGATAGTTTGAGATGCTCAAAGGTATACTCTACAAACAAACTAGAAAGTGATATGTGGGGAAGAAACAGTGATGATGAAATGTGTCAGATTGATGACAAGGATGATTTTATGTTAGATGAAGTAAAAATTGGCTCTTCTATTATACATGAGAATATGAAGAGTTATAATCCAATTTGTGAGCCCTCTGATGATGAGGATTGCAAATTTGATAGAAGTCCAGTAGTAGACCGAGGAAGCAGTTTACATGAAGAAGCTCTTGCACCTGGAAGCTGTTCTGGAGCTTTTGACGAAGAACAAAACTTCTCATTTCCTAGTGGGCGATCACTTTCTGAAGATGAGGATGAGCTTACTGAATCAAAAATTAAAGCTTTCTTGGATGAAAAGGCTCTTGAATTGAAGAAACTGCAGTCACCGCTTTATGAGGAGTTCTACAATAGTTTGAATGCATCCTGCTCTCCAGTTTTTATGGAGAGCAAACAAGATGAAAGCACTCCTAAATACTTGAAATTGCCCCCAAAAAGCCGGTCACCAAGTCGAAGTCCTGGTAACCCATCCCCAGCGCTTGATGCTTTTGGCACTGGTAGCCCAGGGAGCGGTAGCAGGGGTAATGCAAATGATCAAAGGTCACAACTCAACGATTGGAAAGGACTTCATGGTCAGTCAGAAGCTGGTAGCCCAAGTAAGAATTATTCTGAAATACAGAGGAAGTGGAAAGAAGAGCTTGATCAAGAGCTCGAGAGAAAACGAGAGATGATGCGTCAAGCTGGCGTGGGAGCCAAAACTTCATCTCCAAAGGATAAAGCCATGGGCAGGCCAAGAGAGCGAACACGGTTTGCATCTCCATTCCGTGATGATGTGTCGGGTGCTGGGAGGGACAACAGGGAAAACTTCATCTCCTGA

>Csa6M483320.1

ATGCCTCTTGGGTGGGTTAAAAAATTGAGTAGAAATAAGGACCATCACAATCAAAATCACCCCACTTCTCTCAACCTCTTCAAGTCTTCTTCTTCTTCTTCTTCTTCTTCTTCCCCCAAAACTCAACCTAATAACACCATCACCCACAAACCCAAGAGCTTTGATGAGGTTTCTGCTTTGATTTTCTCTCGTAATTCTCCCAGGTCTAGCAGGGATCTGGGCTCTTCTGGTACTGCCTCTTCTGGGTTTTCTGGTTTTGACTCTGATAGTGGGCATAAGAGTCTTCCTCTTCCTCGACCTGCTACTTCTGGCCTTGGGATTGACCATGGAGCTGGGAATGGATCTGGGTCCAGTTCGGTTTCTAGTGATATCTCTTCTGGCTCTTCAGATGATCAACCATCTGCTCAAGAACAGCTTCAATTTGGAGCTTACAGAGGATTTGCCGATAACAGAATCGAGACAAGAGCAAGAAGTCCAGGTCCAGGATCAAAAGGACCCACTAGTCCTACATCGCCTCTGAACCCACGTTTCTGTGGCATGAGTCTTGAGTCTCCTCCAACTCATAAGCTGCCGCTTCCTCCAAGTGCTCCCACCAGCCCTTCTTCCTTGACCAGCATGAGAGCTATCAATATCGGCGATAACAACGCAGCTGTACAATCGAAATGGAAGAAAGGAAGGCTTCTAGGGAGGGGAACATTTGGGCATGTTTACCTTGGATTTAACAGTGTAAGTGGCCAAATGTGTGCAATTAAGGAGGTTAGGGTTATAAGTGATGATTCAACATCAAAGGAATGTCTCAAGCAATTGAACCAGGAGATTACTGTGCTCAGTCAGCTGTCGCATCCGAACATTGTCCGGTACTATGGTAGTGAAATGGGTGAAGAGTCACTCTCAGTCTACTTAGAATACATTTCCGGCGGTTCAATTCACAAACTACTTCAAGAATATGGCGCCTTCAAAGAACCTGTTATAAGAAATTATACCCGGAAGATTCTTTCTGGGCTTGCTTATTTGCATGGGAGAAATACAGTGCATAGGGACATCAAAGGGGCAAATATCTTAGTAGATCCGAAAGGGGAGGTCAAGTTGGTGGACTTTGGCATGGCAAAACATATAACGAATTGTACTTCGATGCTTTCCTTCAAAGGGAGTCCTTATTGGATGGCCCCTGAGGTTGTGATGAATACAAATGGCTACAGTCTTGCAGTAGACATTTGGAGTTTAGGATGTACTGTTCTTGAAATGGCAACGTCTAAACCGCCTTGGAACAGATACGAAGGGGTGGCTGCCATTTTCAAAATTGGGAATAGTAAAGACATTCCCGAAATTCCAGACTCTCTCTCCAGTGATGCTAGAAGTTTCGTGCAATTATGTTTGCAACGGGATCCATCTGCCCGTCCTTCTGCTGCTGAACTACTGGACCACCCTTTTGTTCAAGATGCAGTAACACCAAGAGCATCTGATGTTAACTTATCTGTGGATGCATTCCCCTTTAGCTTCGATGGAATCCAGACTTCGCCATTGTTAGATCGTCATCCAAACAGAAAAAGTATAAGCATATGTGATGGAGATTATGTGACAAACCCAACATTCTCTTCCAGAGCTCCGAGTCCTAGGGGAAATGGAAGATTGATCACATCCTTGCCTGTCTCTCCATGTTCGAGTCCATTACGGTCATATGGCCCCACACATCAGAGCTGTTATCTTTCACCACCTCACACGTCTTACATGGGGGTGGGTCAAAGTGGATACAATTTGAATGAATATGCATATAACACCAGACCAAACACATTGTTCACCCTTGATCCTTCGCGAGAGTCATCCCTCTTGAAAGTGCAGACTCATCTTGGATCACCAAGAAGACCTCTTTGA

>Csa6M483320.2

ATGCCTCTTGGGTGGGTTAAAAAATTGAGTAGAAATAAGGACCATCACAATCAAAATCACCCCACTTCTCTCAACCTCTTCAAGTCTTCTTCTTCTTCTTCTTCTTCTTCTTCCCCCAAAACTCAACCTAATAACACCATCACCCACAAACCCAAGAGCTTTGATGAGGTTTCTGCTTTGATTTTCTCTCGTAATTCTCCCAGGTCTAGCAGGGATCTGGGCTCTTCTGGTACTGCCTCTTCTGGGTTTTCTGGTTTTGACTCTGATAGTGGGCATAAGAGTCTTCCTCTTCCTCGACCTGCTACTTCTGGCCTTGGGATTGACCATGGAGCTGGGAATGGATCTGGGTCCAGTTCGGTTTCTAGTGATATCTCTTCTGGCTCTTCAGATGATCAACCATCTGCTCAAGAACAGCTTCAATTTGGAGCTTACAGAGGATTTGCCGATAACAGAATCGAGACAAGAGCAAGAAGTCCAGGTCCAGGATCAAAAGGACCCACTAGTCCTACATCGCCTCTGAACCCACGTTTCTGTGGCATGAGTCTTGAGTCTCCTCCAACTCATAAGCTGCCGCTTCCTCCAAGTGCTCCCACCAGCCCTTCTTCCTTGACCAGCATGAGAGCTATCAATATCGGCGATAACAACGCAGCTGTACAATCGAAATGGAAGAAAGGAAGGCTTCTAGGGAGGGGAACATTTGGGCATGTTTACCTTGGATTTAACAGTGTAAGTGGCCAAATGTGTGCAATTAAGGAGGTTAGGGTTATAAGTGATGATTCAACATCAAAGGAATGTCTCAAGCAATTGAACCAGGAGATTACTGTGCTCAGTCAGCTGTCGCATCCGAACATTGTCCGGTACTATGGTAGTGAAATGGGTGAAGAGTCACTCTCAGTCTACTTAGAATACATTTCCGGCGGTTCAATTCACAAACTACTTCAAGAATATGGCGCCTTCAAAGAACCTGTTATAAGAAATTATACCCGGAAGATTCTTTCTGGGCTTGCTTATTTGCATGGGAGAAATACAGTGCATAGGGACATCAAAGGGGCAAATATCTTAGTAGATCCGAAAGGGGAGGTCAAGTTGGTGGACTTTGGCATGGCAAAACATATAACGAATTGTACTTCGATGCTTTCCTTCAAAGGGAGTCCTTATTGGATGGCCCCTGAGGTTGTGATGAATACAAATGGCTACAGTCTTGCAGTAGACATTTGGAGTTTAGGATGTACTGTTCTTGAAATGGCAACGTCTAAACCGCCTTGGAACAGATACGAAGGGGTGGCTGCCATTTTCAAAATTGGGAATAGTAAAGACATTCCCGAAATTCCAGACTCTCTCTCCAGTGATGCTAGAAGTTTCGTGCAATTATGTTTGCAACGGGATCCATCTGCCCGTCCTTCTGCTGCTGAACTACTGGACCACCCTTTTGTTCAAGATGCAGTAACACCAAGAGCATCTGATGTTAACTTATCTGTGGATGCATTCCCCTTTAGCTTCGATGGAATCCAGACTTCGGTAAGCCTTTCGGCATCACTTGGCTTACTTAGTGAATCGCAAAGCCAAGATGAAATTATTTCTTTATAA

>Csa6M483320.3

ATGCCTCTTGGGTGGGTTAAAAAATTGAGTAGAAATAAGGACCATCACAATCAAAATCACCCCACTTCTCTCAACCTCTTCAAGTCTTCTTCTTCTTCTTCTTCTTCTTCTTCCCCCAAAACTCAACCTAATAACACCATCACCCACAAACCCAAGAGCTTTGATGAGGTTTCTGCTTTGATTTTCTCTCGTAATTCTCCCAGGTCTAGCAGGGATCTGGGCTCTTCTGGTACTGCCTCTTCTGGGTTTTCTGGTTTTGACTCTGATAGTGGGCATAAGAGTCTTCCTCTTCCTCGACCTGCTACTTCTGGCCTTGGGATTGACCATGGAGCTGGGAATGGATCTGGGTCCAGTTCGGTTTCTAGTGATATCTCTTCTGGCTCTTCAGATGATCAACCATCTGCTCAAGAACAGCTTCAATTTGGAGCTTACAGAGGATTTGCCGATAACAGAATCGAGACAAGAGCAAGAAGTCCAGGTCCAGGATCAAAAGGACCCACTAGTCCTACATCGCCTCTGAACCCACGTTTCTGTGGCATGAGTCTTGAGTCTCCTCCAACTCATAAGCTGCCGCTTCCTCCAAGTGCTCCCACCAGCCCTTCTTCCTTGACCAGCATGAGAGCTATCAATATCGGCGATAACAACGCAGCTGTACAATCGAAATGGAAGAAAGGAAGGCTTCTAGGGAGGGGAACATTTGGGCATGTTTACCTTGGATTTAACAGTGTAAGTGGCCAAATGTGTGCAATTAAGGAGGTTAGGGTTATAAGTGATGATTCAACATCAAAGGAATGTCTCAAGCAATTGAACCAGGAGATTACTGTGCTCAGTCAGCTGTCGCATCCGAACATTGTCCGGTACTATGGTAGTGAAATGGGTGAAGAGTCACTCTCAGTCTACTTAGAATACATTTCCGGCGGTTCAATTCACAAACTACTTCAAGAATATGGCGCCTTCAAAGAACCTGTTATAAGAAATTATACCCGGAAGATTCTTTCTGGGCTTGCTTATTTGCATGGGAGAAATACAGTGCATAGGGACATCAAAGGGGCAAATATCTTAGTAGATCCGAAAGGGGAGGTCAAGTTGGTGGACTTTGGCATGGCAAAACATATAACGAATTGTACTTCGATGCTTTCCTTCAAAGGGAGTCCTTATTGGATGGCCCCTGAGGTTGTGATGAATACAAATGGCTACAGTCTTGCAGTAGACATTTGGAGTTTAGGATGTACTGTTCTTGAAATGGCAACGTCTAAACCGCCTTGGAACAGATACGAAGGGGTGGCTGCCATTTTCAAAATTGGGAATAGTAAAGACATTCCCGAAATTCCAGACTCTCTCTCCAGTGATGCTAGAAGTTTCGTGCAATTATGTTTGCAACGGGATCCATCTGCCCGTCCTTCTGCTGCTGAACTACTGGACCACCCTTTTGTTCAAGATGCAGTAACACCAAGAGCATCTGATGTTAACTTATCTGTGGATGCATTCCCCTTTAGCTTCGATGGAATCCAGACTTCGCCATTGTTAGATCGTCATCCAAACAGAAAAAGTATAAGCATATGTGATGGAGATTATGTGACAAACCCAACATTCTCTTCCAGAGCTCCGAGTCCTAGGTACTAA

>Csa3M182770.1

ATGCCTTCATGGTGGGGGAAGTCGTCCAAAGACGTGAAGAAGAAAACAAGTAAAGAAAGTTTCATCGACTCATTACACCGAAAGTTTAAGAATTCACCTGAAGGTAAAGTAAACAGCAGATCAGGAAGTTCTCGTAAACGTGGTGGTGACACTGTATCTGAGAAGGGGTCCAAATCACCAATATCAAGATCACCATCACCTTCCAAAGAAGTAGCTAGATGTCAAAGTTTTGCTGAAAGAACGCATTCTCACAAACTTCCACTTCCAGATTTGCGACCTGTAGGTGTAGGTCGCACAGATTCTGGGATTAGTGTGGCAGCAAAATCCAAATTAGAGAGAAGCTCCAAGACATCCTCATTCCTACCCCTTCCTAGACCTGCTTGTATTCGAAGTAGGCCAGATCCTGCAGATTTGGATGGAGACTTGGTCACTGGTTCAGTCTTTGGTGAGTCCTCAAGTGATAGTGATGATCCCAATGATTCACGTCAACGTAGTCCTCCAGCAACTGACTATGACATTGGGGCTAGAACTGTGATAGGTTCTACTGAACCTAGTGAAACTCTCAAAGATCAATCTCCTACTGTCGTGCAAAAAAATTTAAAAGAAGGCAAGAAAGCGGAAAGTCTTCCATTTCCTCATAAGAATTCTTCAATACCTAAACGGAGGCCTTTAAGCAGCAACGTAACAAATCTACAAGTTCCTCGTCATGGGGCCTTCTTTAGCGCACCAGATAGTTCTATGTCTAGTCCCTCTAGAAGTCCTATGAGGATATTTAGTACTGAGCAAGTCATGAACGCCGCTGTTTGGGCTGGAAAATCTCACCCAGATGTCATTTTAGGTGGATCTGGCCATTGTTCTAGCCCGGGTTCTGGTCACAATTCAGGGCACAACTCTATGGGAGGTGATATGGCGGGCCACTTTTTTTGGCAACAAAGTAGGGGTAGCCCTGAATATTCTCCTGTACCTAGTTCCAGAATGACTAGCCCTGGTCCTAGTTCCAGAATTCAAAGCGGTGCTGTCACACCTATTCATCCTAGAGCAGGAGCTCCACCTGCTGAGTCACAAACATGCTGGCCTGATGAGAAACAAACTCATCGTTTGCCTCTTCCTCCCATAGCAATCTCAATCTGTTCTCCATTTTCACATTCGAATTCGGCAGTGACATCTCCTTCAGTGCCTAGAAGTCCAGGAAGGACAGAGACTCCAGCAAGCCCAGGTCCACGCTGGAAAAAGGGGAAGCTACTGGGTCGGGGCACATTTGGGCATGTATATGTCGGTTTTAATAGCGAAAGTGGAGAGATGTGTGCAATGAAGGAGGTCACATTATTTTCTGATGATGCGAAGTCTAGGGAAAGTGCTAAGCAATTAATGCAGGAAATTGCTTTACTGAGTCGTTTGAGGCATCCCAACATCGTGCAGTATTATGGTTCCGAAACAGTGGGTGATAAATTCTATATATATTTGGAATATGTATCTGGTGGTTCCATTTATAAATTACTTCAAGAGTATGGACAATTTGGGGAGCTAGCAATCCGTAGCTATACTCAACAAATACTATCAGGACTTGCATATTTGCATGCTAAAGCTACAGTGCACAGGGATATTAAAGGAGCAAACATACTTGTTGACCCGAACGGTCGTGTTAAACTGGCAGACTTTGGGATGGCGAAGCATATCACAGGTCAATCATGTCCACTATCGTTTAAGGGTAGCCCTTACTGGATGGCACCTGAGGTAATAAAGAACTCAAATGGTTGCAACCTTGCCGTTGATGTTTGGAGTCTTGGATGTACTGTTCTGGAGATGGCTACTACAAAGCCACCATGGAGCCAGTACGAAGGGGTTGCCGCGATGTTTAAGATTGGTAACAGCAAGGAACTCCCTGTAATTCCAGAGCACCTCTCAGATGATGGGAAGGATTTTGTTAGACTATGTCTGCAACGAAACCCACATCATCGACCTACAGCTGCTCAACTCTTGGAGCATCCTTTTGTCAAACATGCTGCACCTGTAGAGAGGCCTATTTTGATCTCTGAACCTTCTGATACAACTCCTGGAGTTACAAACGGAGTGAAGATTTTGGGTATTGGTCAATCACGGACTACTTCTATGGACTCGGATGGTAGACTTGCAGTTCATTCTTCAAGAGTCTCAAAAGCTGTTCTTCATGCCAGTGAAATCAATATTTCCAGAAACATATCATGTCCTGTTTCGCCAATCGGAAGCCCTCTTTTGCATTCACGATCTCCTCAACACCCTAGTGGAAGAATGTCTCCTTCACCCATTTCAAGTCCACGAACCATGTCGGGATCATCCACACCTTTGACAGGATGTGGTGGTGCAATTCCTTACAACCACCTGAAACAAACTATATACTTGCAAGAAGGATTTGTAAGCATGCCAAAATCCTTAAATAGCAGTCCATACAGCAGCGGTATCTCCTTCCATGATTCAAATCCCGATATTTTCCGAGGGTTGCAGCCTGGGGCTCACATCTTCTCTGAAATGATACCAGAAAACGAAGTCTTGGGAAAGCAGATCGGACGGCCTGCTTACAGTGAGGTGTATGATGGCCAACACATTTTGGCCGACCGAGTGTCTCGGCAGCTCCTGAGGGATCATGTGAAAGCAAATCCTTCTCTTGATTTGAGTCCTAGCGCTACATTGTCGGGTCGCATGAATGGTATTTGA

>Csa5M166980.1

ATGCCTTCATGGTGGGGGAAGTCATCATCAAAAGAAGTAAAGAAAAGCAAGGAAAGTTTAATCGACACGTTGCAGAGAAAACTTAGAACTACCGATGGTAAAACAAACAGCAAATCAGGAGAGTCTCCAAGACATTGTAATGACACAATTTCTGAGCAGGGATCTCGATCTCCTATTCTTTCAAGATCAGTTTCCCCTTCCAAACAAGTTTTAAGATGTCAAAGCTTTTCTGAAAGGCCGCAAGCACAACCTCTTCCACTTCCTGGTGTGCAGCCACCAATTGTAGGTCGGACAGACTCTGGGATTAGTATTTCACCAAAACCAAGATCTGAAAGGGGCTCCAAGCCAACATCATTTCTACCACTTCCAAGACCAGCATGCATCCGTGGGCGGCCAAACCATGCAGATTTAGATGCAGATGTTGGTGTTGGCTCAGTGTCCAGTGAGAGCTCAACTGATAGCACAGATCTATTGGATTCACGCCATCGTAGTCCTCGGGCAACTGACTATGATCTTGGGACTAAAACTGCTGCAAGCAGTCCTTCCAGTGTCATTCTCAAGGATCAGTCTTCTACTGTCACCCAACCAAGTTTGCAAAAGGCCAGAAAACCGGCTAATATCTCATTGAGCAACCACATTTTCTCAACATCACCCAAGCGCAGACCTTTAAGCAGTCATGTTCCAAATCTGCAAGTTCCATATCATGGGAATGTATGCATTGCTCCTGATAGTTCAATGTCAAGTCCTTCTAGGAGTCCCATAAGGGCATTTAGCTCCGAGCAAGTTATTAATAATGCTGTCAGTACTGGAAAATTCTATATGGATGTCACATTTCCTGGGTCAGGCCATTGTTCCAGTCCTGGTTCTGGTTACAATTCTGGACATAATTCTATGGGTGGGGATTTGTCAGGGCAGTTATTTTTGCAACAAAGCCGGGGTAGCCCTGAATATTCTCCAGTACCCAGTCCCAGAATGACCAGCCCTGGCCCAAGCTCCAGAGTCCATAGTGGTGCAGTGACCCCAATTCATCCTAGGGCGGGAGGTATACCAACTGATTCACAGACAAGCTGGCCTGATGAGAAGCAAACTCACCGCCTGCCTCTACCTCCCGTTGCAATTTCCAACGCTCCTTTTTCTCATTCCAATTCAGCTGCAACTTCTCCCTCTGTTCCAAGAAGTCCTGGAAGGGCTGATAATCCGGCAAGCCCGGGCTCCCGTTGGAAAAAGGGGAAGCTCTTGGGTAGGGGCACTTTTGGACATGTGTATGTTGGTTTTAACAGTGAAAGTGGTGAAATGTGTGCAATGAAGGAAGTTACATTATTTTCTGATGATGCGAAGTCCAAGGAGAGTGCCAAGCAATTAATGCAAGAAATTACCTTGTTGAGTCGTTTACGACATCCAAATATTGTGCAGTATTATGGATCTGAAACGGTTGGGGACAGGTTTTACATTTACCTTGAATATGTATCTGGTGGCTCTATTTACAAGCTTCTCCAGGAATATGGACAGCTTGGAGATTCAGCACTTCGTAGTTATACTCAGCAAATATTGTCGGGGCTTGCATATTTACATGCTAAAAGTACAGTTCACAGGGATATCAAAGGAGCAAATATACTTGTTGATCCTACTGGTCGTGTTAAGTTGGCTGACTTTGGGATGGCAAAACATATCACTGGCCAATCGTGCCCTTTGTCATTTAAAGGAAGCCCATATTGGATGGCACCCGAGGTTATCAAAAACTCAAATGGTTGCAACCTTGCGGTAGATATTTGGAGTCTTGGATGCACTGTTTTGGAGATGGCAACAACAAAACCTCCTTGGAGTCAATATGAGGGAGTCGCTGCGATGTTCAAGATTGGCAACAGCAAAGAACTTCCTGAAATCCCAGATTACCTTTCACATGATGGAAAAGATTTTGTTAGACAATGTCTGCAACGGAATCCTGCTCATCGTCCTACAGCTGCTCAGCTTTTGGAACATCCTTTTGTGAAACATGCTGCACCTCTTGAAAGACTGATTTTAGGTTCTGAACATTCAGATCCAACTCCAGGAATTACAAATGGAGTAAGAACATTGGGTATTGAACAAGGAAGGAATCCCAGCTTCTTGGATTCTGATAGATCTGCAGCTCATTCATCTAGACTCCCAACAGCTGCTTTCCATTCCAGTGAAATTCATATTCCAAGGAACCTATCATGCCCTGTTTCGCCCATCGGAAGCCCACTGGTGCACTCACGGTCGCCACAACATCCGAGTGGAAGAATGTCTCCGTCACCCATATCTAGCCCTCGTAACATGTCGGGCGCATCTACTCCTCTCACAGGAGGAAGCGGTGCCATTCCACATCAGCATCTCAAGCAATCACTGTACCTACAGGAGGGTTTTGGGAACTTGCCAAAACCTTCAATGGCTCCTTATAGTAATGGTCCTTCCTTTCACGATACTAACCCTGACATCTTTCAGGGGATTCAGCCAGGCTCACACATCTTTTCTGAGCTTGTACACCATGAAACCGATTTTCTGGGCAAGCAGTTTGGAAAGCCTGCCTGGGAATTGTATGACGGGCAGGCGGTCTTGGCTGATCGTGTTTCCAGGCAGCTGCTGAGTGATCACATAACAACTCCCTCCCTGGATTTAAGTCCAAGCTCTCTTTTGACCAACCGCAAATAG

>Csa6M490220.1

ATGCGTTGGCTGCGTAATATCTCCTTTACCCCTTCTTCAATGGTGAGGCTTCCGGGTTCTACAACTGGCGGTGGTGATTCTCCCACCCGAAGATCTACTACTCGAGGTTCTGCTGAAAATAACTACCGGGGAATTGTTTGGCGTTTTGGTGCCTCTAGGTATTCCCGCCATAGGAAGCTTCGTAACTTAAGTGGACGTGAACACGTCGATTCTTCTTTGGCTAAATGGTCTGATACTGCGCCTGAACCTGTCTCTTTGTCGCGATCACCCAGCACCTCTGATCACCCTGCTGTTCCCTTACCACTCCCTGAGGTTTCTCCCTTATTTCAGCCTCGTGAAAGGATCTCCACTTCCAATTCTGCTGGGGGAGAGGGTGATTGCCCTCTTCCATCGCCCAAAGGTTCGCGTGGACGCGCCGGGGATGAACGTGATGTAGATAGAGATAGAAATGCACCCCCTCAGAAGATTGGGGGTGGAATCTCCCCTAACGCTTCCATTAAAAGTGTATCTGATTCTGTGGGGGAAAGACATAAGAAAGAAGGTCAGATTGAAGCAAGGTTGTCAGGTAGGGCGAATCAGGATGCAAGGAGATATCCCGAAAATTCTCGGAATGGTTTTTGGATTGATGTTCCTAGTAGGAGTGCTCCTACTAGTCCTTATACAAGTCCTACACCTAGTCCTCAAAGAAATATTTCGGTTGGAAATCACGTCTGGTCTGCACCTGAGCTGCCATCCTCGGCAATGATGCGGGGGGTTCCTCCTGCATTCTTTGATTGCTCTACTCTTAGTACTGAGAGTTCTCCCATGCATAGCCCAAGAGGTAAAAGTCCCCACCTAGATCCCAGAAGTCCAACAGGACCTACATCTCCATTGCACGCCAAGATTTCCCATGAAACACATGCTATGCGCCGTGAGGGTTCTGGTCATGTCAGTGTCCACCCTTTGCCTCTTCCTCCTGGAGTGCCAATGCCTTCAGCGTCAATCCCAACAATGGCTTCAGCACCAACCTCCATCAATCTATCATCACCCCCTGTGTCTTCACCATCACATTCAATACCTTCGGCATCATGCTCGATGGCTCTACCGTCAACCCCGATGGCATCACCATCAACCCCAATTTCACAAGCTAACACTAAGAGCGAATCAATCTCAATGAAAAATCAATGGCAGAAAGGAAAGCTTATTGGTCGGGGCACATTTGGAAGTGTGTATGTTGCTAGCAACAGACAAAATGGAGCTTTATGTGCAATGAAGGAAGTTGAACTATTTCACGACGATCCAAAGTCAGCTGAATCTATAAAGCAATTAGAACAGGAGATCAAACTTCTGAGCCAGCTAAAGCATCCAAATATTGTTCAATACTATGGTAGTGATATTATTGACGATCGATTATATATATACCTGGAGTATGTACATCCTGGTTCAATTAATAAATATGTTCGTGAACATTGTGGAGCTATGACAGAATCTGTTGTTCGAAATTTTACTCGACACATCCTCTCAGGGTTGGCCTACTTGCACAGCACAAAGACAATACACAGGGACATTAAAGGTGCTAATTTGCTGGTAGATTCTTGTGGGGTTGTCAAGCTTGCTGACTTTGGAATGGCTAAACACCTTACAGGACAAGTTGCTGATCTTTCTTTGAAGGGAAGTCCATATTGGATGGCTCCTGAGCTCCTTCTGTCTGTGATGCAGAAAGATAACACCCCCGATCTAGCCCTAGCAGTTGATATTTGGAGCCTTGGTTGCACTATTATAGAAATGTTCACTGGCAAACCACCTTGGAGTGAGTATGAAGGGGCTGCAGCTATGTTTAAAGTCATGAAAGATACCCCACCAATGCCTGAATCATTGTCATACGAAGCGAGAGATTTCTTAAAATGTTGCTTTCAAAGAAATCCTGCAGAGAGGCCGACCGCTGCCATGCTGCTTGAACATCCGTTCATGAAAAACTTGCAGTATACAGATGCTTCATCTTGCTCTCAGGTTGTTGCTGGAGCAAGTTTGATGGATAAATGTTATAGCCCAAGTAAACAATATTCAAGTAAATCTGATCAGTCTTCCATGCTTCCAAGCCCACAAAATTCCAAGGGAAAGTTAGCGGCCGATAATGTGATTGGCCCACTATCTCATCATGAAACCTCTGACTTAACAGTAATGTCTCGTTATTCCCCCCGTTCTACCCTTGAAGCTCTTCCAATGGTGTCTCCTCTGCGCTCGGTTCCCAATGCGCATCATTATGGTTCCCCTACAAATGCAGCTGATATTGTAAATCAAATCAATAGGAAGAACCATACATTAATATGA

>Csa2M360650.1

ATGCCTTCCTTTAACAAAAGCGCTTCAAATTCTCGGCTTCAACAGAAGGTACCTGATTCTGATTTTTTTGATTTTTCTGTTGGTCTACCGGAACCGTTTATTCAGCGGCGGCTTACCAGACAGAGGAAGCTCCGGCATCTTACGGATCAAGACGTTGGCTTTGAACACCCCCGATCGCTTCCTGATTCACCTGATATATCGGCAAAACCTAAATCGCCTCTTGGGGGTTCTGAACGTTGGTCCTCCTCGCCTTCGCCCCAGCCTTTGCCGTTGCCGGAGCTCTTTCCGGTTCGGAGCCCGGAGTTCGGTTCTAATTATGGTCAAGGGCGTGTTGGATCGCCTGTTGAAACTTCGGTCCGGAAGTGCTCTGATCATGCGACAACGAATGTAAGTAGAAGTTTTGGGCACAATCAAAGGCGGGTTGCTACAGATTTGACCCTGGAAGTTGTTGATGGCAATTCAAGGACTGGGGCTACCGCATTTACGAGCCCTCAAAGTCTCTCGGCGAATTCAGGGAAATTGAATCCGAATAAGGAGTTTCTTTTTGGTTGTTCGGATCAAACTCCAAACAACAAAGGCAGGGGATCCCCTACGCATCCGAGTGCTGAAAGGGGTAACTACAATTTGACACCTAAGAAATCGTCGTCAAAGAGTGCACCAACGAGCATTTTACCAAGTCCTGTTGTTAGCCCACGAAAATCATATAATGGAAATCATTTTGTTCCTGGCTTGAGTCACCATGAACATCAGGAATCTCCTTCCAATAATTCTCCTAAAGTACCACCCCTTAAAACTGCACTTAGTTCTCAGCCCTTTCCTCTCCATAGCCCGACAGCTCGAAGTTCCATAAGCAATTCCCGATCTCATAACGATATGACATTTCCCCTGCACACTAAGTTACAAAAAGATAATAGCATAGATCGGTCTGAAAGTCATGCTCATGTCAATGCCCACCCATTGCCCCTTCCTCCTCCATTAGTGGCCTCTTCACAAGCATCTGCACAGTCTCTTCCGTCAAATGTTCATCATGTTATTGAGAAGCCATTCATTTCATCGATGAAAGGTCAGTGGCAAAAAGGAAAGCTAATTGGGCGTGGTACGTTTGGAAGTGTTTATCTTGCAACCAACAGAGAAACTGGAGCTTTATGTGCAATGAAAGAAGTTGATCTCATTCCTGATGATCCTAAATCTGCTGAATGCATAAAGCAACTAGAGCAGGAAATTGAAGTTCTTAGTCATCTTAAACATCCGAATATTGTACAGTATTATGGAAGTGAAATAATTGGTGACTGTTTCTATATATATTTGGAGTACGTTTATCCTGGGTCAATAAATAAATATGTCCGTGAACGTTGTGGAGCCATTACTGAATCTATTGTTCGCAATTTCACTCGCCATATTCTATCTGGACTGGCTTACTTGCATAGCACAAAAACAATTCACAGAGATATCAAAGGTGCCAACTTGCTCGTTGATTCATCAGGCGTCGTCAAACTTGCCGATTTTGGGATGGCAAAACATCTGACAGGGCAGTATGATCTTTCTTTGAAGGGTAGTCCGTACTGGATGGCCCCAGAGGTCATAAAAGCTGCAATGTTGAAAGATGCCAACCCTGATCTTGCTTTGGCAGTTGACATTTGGAGTCTGGGTTGCACCATAATTGAAATGCTGAATGGAAAACCTCCTTGGTGTGAGTTTGAAGGGCATCAAGTTATGTTCAAGGTGTTGAATAAAACCCCACCTATCCCAGAAAAGCTATCTCCTGAAGGGAAAGACTTCCTCCAGTGTTGCTTTCAGAGGAATCCAGCTGACAGACCAACTGCCATGGTGCTCCTTGACCATCCTTTCTTAAGATCCTCAAGTGACTCGAATGCTTCGATCCCAACGTCAGCATTTTCAACAATGAATCTCTTGGAAAAATTGTTGAGTCCGAAGGATCCCCTTAATCCTAAAGGAGACCAGGCACAAAACTCTTCTGGAACTTTGACTTCAAATGATTATTTGTCGTGCCGTAGCCCTTCAACAAACATTTCAAGCAATGTTCCTGCAAGTGCAGTCGCCAACCACCACTTTTCTATTTCAAGGACTCATAAAAGGGAAGTGCCGCACCTCTGA

>Csa5M385380.1

ATGAATCGTCTCCCTCAGTTTTTCTCTCCTAGGAACAAAAGAAAACCCATGGATCCCAGGAAAATTCCAGGGAAACCCAAGCTTGGCCGACTTAATGCGGCCAAGAATATTGATTATGATGCTCCTTCCTCGTCTTCTTCGCTCGAAGATTCTTCTGGGTCTCTTTACACTCGTTCCATGGAAAACCCCGATCCCTCCAGTTTTCGGATTAAAGGGCTTGACGGTGAAGTTGATCTCATATGTCGGACCCTAGGGCTAGCTGGCCCTGACGACTTTGCTATTCCGATGGAGGCTTGGGAGGCTAGAAAGGTTCGTTCATCTTCCGAGCTTCTACCGAGGTCGAGGCTGTATCCTATGGATACCTCTCCGAAGACAGAGGAGATTAGTGAGGATAAGGAAGATAAAGAAATTCAAGATGAATTATGTCGGAGGGTTAAGGATTCAGTTCGAATTAGCGTCGACCTTTCCAAGACAAAGACCGAATTTGCAGAGTTGAATGAACGCCGGATGGCTACTGCTACTGGATGTAGTTCGAGAAGTGGAATCAATGGTGCCCGGCCTCCTTTACTTAAGCCGCCGCCGTCCATGCGGCTACCAAATTTTGATAATGCTTACTCTACTTGGGACATTTTGAAAGGTTTTGCTCCATTAGTTGAGGATGAACATCAAGAGGAAGTAGGAGAACGTGTGGAACCTTTAGTTGAAGTAGAAGGAGAAGGAGAAGGAAATACAGTAAGGCCTGTGGAGAACGCATCGCTCATAGGGTCTTGGGGTTCCTTCACGACATCGAATGATGATGATTCTTCAAGCAGTACTACGGAGCCTGCAAATATTTCACCCAATATGAGAGTTAATCCCATTATCACATCCTGGGTTTTAGGCAGGCTTCTTGGGCGCGGTTCATTTGGATCAGTATACGAAGCAATTTCAGAGGATGGGACATTTTTTGCACTCAAAGAAGTTTCACTGCTCGATGAAGATAGTCAGGGAAGACAAAGTATCTATCAACTTCAGCAGGAGATAGCTCTTCTAAGTGAGTTTGAGCATGAGAACATAGTTCAGTACTATGGCACACATTCGGATGGGTCAAAACTATATATCTTCCTCGAGCTTGTATCTCAAGGCTCCCTTATGAGTCTATATCAGAGGACAAGCCTTATGGATTCCATTGTTTCTGCCTACACAAGGCAGATCCTATCGGGTTTAAAGTATCTTCACGAGCGAAATGTGATTCACAGGGATATTAAATGTGCTAATATATTGGTGGACGTGAATGGATCTGTGAAGCTTGCAGATTTTGGTTTGGCAAAGGCAACAAAATTGAACGATGTTAAATCTTGTAAGGGAACTGCGTATTGGATGGCACCTGAGGTGGTTAATGGGAAGGGTCAAGGATATGGTCTACCTGCCGATATCTGGAGCCTTGGGTGCACAGTATTGGAAATGTTAACCCGAAAGCTTCCATATTCAGAATTTGAATCTCATATGCAGGCACTATTTAGGATCGGAAAAGGCAAACCGCCTGCAGTTCCTGAGTCTCTCCCAAAAGATGCACAGGATTTCATTCTGCAGTGCCTACAAGTTAATCCAAAGGACCGTCCTACTGCTGCTGATCTTTTAAATCACTCGTTTGTGAAAAGACCAGTCTCCAGTTTATCAGGGTTGGCATCTCCTTATAACAGGCCAGGCAGGAGGATTTAA

>Csa6M425140.1

ATGCAAGATTTCTTCGGATCTGTTCGTCGATCCTTGGTATTTCTAGCTCCCGACGGTGACGACGGTGGGAGGTTCGGTGGCCTTGTTGAGAAGATCGGCTCTAGCATCCGTAAATCTCGAAATGGACTGTTCTCTAAGCAGTCACTCCGGGCTCTGCCTCCGGTTGCAAAGGAAGATGCGCCTCCGATTTGGTGGCGTAAGGGAGAGTTGATTGGATGTGGTGCTTTTGGTCGAGTTTATATGGGAATGAATCTCGATTCTGGAGAGCTTCTTGCAGTCAAACAAGTCTCAATTGCTGCTAATAGTGCTTCAAGGGAGAAAGCCCAAGCTCACATACGGGAGCTGGAGGAAGAAGTGAGGCTTTTGAAGAACCTTTCTCACCCCAATATTGTGAGATATCTGGGAACTGCTAGAGAGGAAGATTCATTAAATATTCTATTGGAATTTGTTCCTGGAGGATCAATATCATCTCTCTTAGGGAAATTTGGGTCTTTTCCTGAATCTGTTATAAAGACCTACACGAAGCAGCTATTATTGGGGCTTGAGTACCTTCACAAAAACGGCATCATGCATAGGGATATAAAGGGTGCAAATATTCTTGTAGACAATAAGGGGTGTATTAAACTTGCAGATTTTGGTGCATCCAAGAAAGTGGTTGAGCTGGCTACTATAAATGGAGCCAAATCAATGAAGGGTACTCCATATTGGATGGCTCCTGAAGTTATTCTCCAGACTGGACATAGCTTCTCTGCTGATATATGGAGTGTGGGGTGTACTGTCATTGAAATGGCAACAGGGAAACCTCCATGGAGCCAACAGTATCAAGAGGTTGCTGCTCTTTTCCATATAGGGAATACCAAGTCCCATCCGCCCATTCCTGAGCATCTATCTGCTGAGGCTAAAGACTTTCTGTTAAAATGTCTACAAGAGGAACCAAACTCACGGCCTACTGCATCAGACTTGCTGCAGCATCCATTTGTCTCATGTGAGTACCAAGAACCTATTGCAGCAGTCCGTGCCTCTTCTATGGAATCTGGGAAACAAATGGCTGATTCTAGGCTGAACTCTAATGATCTGAAAAAGTCTACAATCCTACGTTCAACCTGTGAGGGACTGAAGGATATTTGTGAAATGGGTAGCTTGAGGTGCTCTTCTGTATTTTCTGGGAACTATGGGTCACGGTCCAACTGGGGCTCGAGCAATTTTGAGGATGACATGTGCCAGATAGATGATAAAGATCTGTTTGCTACTTCATCAATGAAGTACAATTCTATTATATCATCAAATGATTTGAATAAGAGTTTCAATCCTATGTGTGAACCCACTGATGACTTGGATGAGAGTTCAGAGTTGGGAGGCAATCTGATGGAGTTGTCCAGCGTCCAAACTGTCAAGGGGAACGACTCCACTTTTCCATGTGGTCAATCAGCAGCTGAGGATGATGAAGAGGTTACAGAATCAAAGATTAAAGCCTTCCTGGATGAGAAGGCTCTAGAACTGAAGAAGCTCCAAACACCTTTATACGAAGAGTTCCGCAGCACAGTTAATGCAGCTAATGCCATAGGAACAGTTGGAATTGAGAATATCAAAAGTGTATCTAATTTTTTGAAATTGCCTCCCAAATGTGGATCACCAAGTAAGCTGCGTGGTAAAAGACTCTCATCAGTTGATGTTGGTAACTATTCTAGCCGTCAGAGCCGTATAAAACAAGCTAGTATATTGCAAGATCGAGCTCTGCAGGAAATTCAGTCACCTCAACTTGGGGAATGGAACAAATTACTTCAAAACCAGCCAGACTCTTCCACACTAAGTTTCTCTGAAAGACAAAGGAAATGGAAAGAGGAGCTTGAGGAAGAACTTAAAAGGAAACGAGAAATGATGCGGAGAGGAATTGTAGGAGCAATTCACTAG

>Csa1M532310.1

ATGCTCAAAACCACCGCGCTTTCTTGGACTCGAAGTAATTCTCCCATCGGAAAAGGCTCCTTTGCCACCGTCTCTCTCGGAATTCGAAAACCCGACGCTCGGATTTTCGCCGTCAAATCTGTCCAGCAGACTCAGACACTCCGCCCACAAATCGACTGCTTAGAGAATGAAATTCGAATCCTCCGATCCTTGAACTCGCCTTACGTGGTTGCCTTTCTCGGCGATGATGTTTCCCATGAATCTCCCACCACCTCCTTTCGTAACCTTCATATGGAGTATTTGCCAGGTGGCACCGCAGCAGATGACCCCACTGGCACTCGCGATGATAAGCTTTTACGGGAGAGGACATGGTGCCTCGTTTCGGCTTTGAGTTACATTCATTCCAAAGGAATTGTTCACTGTGATGTTAAAGGGAGGAACGTTTTGATCGGATTGAATCCCGGGTTTCTGAAATTGGCTGATTTCGGCTCTGCAATTGAACTTCACGGCCCCGGCCACAGGTCTCGGGATTCGCTTGCGCCACGTGGGAGTCCACTATGGATGGCGCCAGAGGTAGTCCGCGGAGAATTTCAGGGCCCGGAATCGGACGTTTGGTCCCTTGGGTGCACGGTCATTGAAATGGTCACCGGGAAACCCGCATGGGAAGATTTCGGAGCCGATACGCTCAGTCGAATCGGCTTTTCCGACGATTTGCCAGACTTTCCAACATGCTTATCGGAAGTCTGCCGGGATTTTCTTCGGAAGTGTCTCAGAAGAAATCCTAGTGAACGGTGGAGCTGTGATCGGCTGCTGCAGCATCCATTTCTGGCGGCGGCGGCGGCGGCGGCGGCCTCGCCGAAGATTGCTGTAGAGAATTCCCCCAGGTGCGTTCTTGACTGGGTCAACGTCAGTTTCTCCGACGACGAGGAGGAAATTCCACACGCCGACGAAGCCTCCGGATCCGGCGGCCAGGAAAATGAGATATATGGTAAGGAAAGAATTGGGAAATTGAGTACTACGAGTGAATGGCCAAATTGGGAATCAGACGGTTGGTCGGCGGTGAGAAGTAGTTACAGTGAAGCGGCGGCGGAGACAGAGGCGAGCTGCCGGAAAGAAGAAGAAGAAGAAGAAGGCGGAGGGGCAGAATGGGAATGTGGAAATTTGAGAAGGGTAGAAGGGGAAATGGAAGGGAGAAGTTGGGAATATTCGGAGTTTGTAAGAAGAGACAATCACGGAAAATTGGGGGCCGAATATTCAAATCCTGGCGGTATGATCATTCCGGAGCGGCCACGTAATAATTTCGGCGGTGGTGGCGGTTGCGGTAGCGGTGGATTAAGTTTCCGGCGGTTGGGATATGAAATATCTGAAATTACAACAATAATAACCTCATGGATATACTCAATTGAATTGATATTATGTTGTTACTATTGGAATATATTAATGAAGAAATTGGTGTTATTTGGGAATTACACTTTCTTACCTTTCTTTTCTGCCTAA

>Csa6M513560.1

ATGGAGTGGATCAGAGGCCGGACCATCGGCCGTGGCTCCTCCGCCGCGGTCTCCGTCGCTACTGATATTCGGTTTGGTCAAGTGATGGCGGTTAAGTCAGTTGAGTTCTCTCACTTGGATTTTCTCAAGAGGGAACAGAGGATTCTTTCTCAATTGAATTGTTCACGTGTTATTGGTTATAAGGGGTTTGATGTTACGTTGGAAAATGGGAATTTGATGTGTAATCTTTTGATGGAGTTTGCGCCTGGTGGGTCGATTTTGGATGCGATGGAGAAGGCGGGTGGCCGATTAGATGAAGCGACGGCTCAATTTTATACTCGTGAGGTTTTAAGTGGGCTTCAGTATGTTCATTCTAATGGTGTGGTACATTGTGATATTAAGTGTTGTAATATTTTGATGGGTGAAGATGGGATCAAGATTGCGGATTTCGGTTGTGCACGGCGGGTGGAAGAGGTTTCCGGTGGAAATTTGGCTGGGACGCCGATATTCATGGCGCCTGAGGTGGCGCGTGGAGAAAAACAGGGGTTTGCAGCTGATGTTTGGTCAGTGGGTTGCGCAGTGATCCAGATGGTGACCGGCCGAGTTCCTTGGGCGAATTTGTCGGATCCGTTGGCTGCCATTTACAGAATTGGGTCTGGCGATGATTTGCCGGAGATTCCAAGGATCATGTCGGAACAGGGAAAGGACTTTTTGCGGCGGTGTTTGATCAGAGACCCGGAAGAACGGTGGTCAGTTAACGAGCTTTTGAAGCACCCTTTTGTTCAAGAGCAGAAATCTCATCCCAAACAGAATTCAAGAACTCCAACGAGTATTTTGGATCAAGGAATATGGGACACGGTGAACGATCCGGAAACGGTTGAAAGTCCGATCAGACCAAAAATTCAAAGAACACCATTGCAGAGGATACAACAACTGAACGAAGTGTCAACGATTGGAATACCCAATTGGGAGTGCGACGAAGATTGGATAACAGTGAGAAGCATTGGCTTAGAAGAAAACGATATTGTTTCTGTAATGGAAACGCCCTCTTCCTTTCAATCCATCAAAATGGAAACTAACAACGGAGTTGGTGATCAGGACTATATTAATGTTAGTAGAAGTAGAAGTAGTGACTATGGTAGCAGAAAAAGCAGTAAGGCTAAGAACAGAGACTTTCCTGCTATGTCCTCTGTTTTCAACCAAATTCCAGGGGTTTATTTTGTCTTATAA

>Csa2M416770.1

ATGGACTGGACTAGAGGCCACGTCATCGGCCACGGCTCCTCCGCCACCGTCTTCCTCGCTACCGATTCTCCCTCTCGCCATGTCTTCGCCGTCAAAACTGCCCAGCTTTCCCACTCCCAATCTCTACGAAAGGAGCAACAGTTTCTTTCTTCTTTAGCCTCTCCTTATATTGTTTCTTATAGAGGCTTTGAAGTCAGTAGAGAACAGAGTGGGGTCACGATGTTCAATCTTTTCATGGAGTATTTACCCAACGGCTCACTCGCCGACACAATTCGCCGCCGTGGAGGACAACGGCTCGACGAGGCAACCATTGTCATCTACACACGGCAGATACTAATGGGTCTACAGTACATTCATTCCAAAGGCATAGTACATTGCGACATTAAAGCCCGAAACATTTTGATTGGTCTAGACGGCGAGGCTAAATTGGCTGATTTCGGTTGCGCCAAGCGGGCAACAAGCCAAACGGATCCAATCTGCGGCACGCCGTTGTTCATGGCGCCGGAAGTAGCTCGTGGTGAACACCAAGGTTTCCCCTCCGACATTTGGTCAATTGGGTGTACAATTATCGAAATGGCCAGCGGTGGCGGCTCGCCTTGGCCAAAAACAACCGACGACACCGACCCGATTTCTGCTCTGTATAGAATTGGGTATTCCGGGGAGTCGCCGGAAATTCCATGTTATTTATCTGAAGAAGCAAAGGATTTCTTGGAGAAGTGTTTGAAAAGGAATCCAAGTGAGAGATGGACGGCGAGTGAGTTAATGAATCATCCATTTTTAAGGGAATTGAATTGTAGAAGGGAATGGAAAACGGAGGAAGTTCATTCAGAATCGCCGACGAGTATTCTAGAACAGGGGATATGGAGATCGATTGAAGAAAGTGAAATTAGAGGAAGGGAATTGGTAAGATCGAATGGTTGGGAGGCAGCGGCAGCAGAGGAGCAGATCCGACGGCTGTGGATGATTTCAGGGGAGCCCAGATGGGAAGAGGATGAGAATTGGATTACAATTAGAAGAAAAGAAGAAGGGGAGAAGAATGGTGGGGCAGATGAGTCAGAAGTGAAAAAATGTAGTAATTATTCTAACAATAATAATAATAATAATAGTAATTGGGGTGACAAAAAGAGAGGTAGTGGAGGGAAAGAGTGGTTGCAAATGGAATTAGGGAATATTAGTTGTAGAATTAGTAGGAATGATTTGGGAGACTATAGTTGTAGGAATGTTATTAGTCTTGTAAATAATAATTATCCTTTATCTTTTCATACACTTACACCTATATTTCTTCCTTTAATGCCAACAATATTATGA

>Csa7M043040.1

ATGGCCTGGACCCGACGATCCCCCGTCGGCAACGGCTCCTCCGCCACCGTCTACCTCGCCTCCACTGCCTCCTCCGGCCAACTATTTGCTGTCAAGTCCGCTGAGCTTCTCAAATCAGACTTCTTGAAAATAGAGCGAAGGGTTCTTTCTTCCCTATCCAACCCTTCAATTGTGGGCTACAAGGGATTCGATGTAACTAGAGAAAATGGGAAGCTAATGTACAATCTCTTCATGGAGTACGCTGCCGGTGGTACTCTCGCTGATGAGATATTCCGGCGTGGTGGCCGGATTAAGGAGGCAACGGCGGCGTTTTACACTCGTGAAATTGTTAGAGGATTGGAGTACTTGCACAAACAGGGGTTGGTGCATTGTGATATCAAAGCTAAGAACATTTTAATCGCTGGAGATGGTTTGAAGATTGCTGATTTCGGATGCTCCAGATGGGTTTGTGAGTCCGAGGCAGTAATAGGCGGAACGCCGATGTTCATGGCACCGGAGGTGGCGCGTGGGGAAAAACAGGGGATTTCCTCTGACATATGGGCACTTGGGTGTACATTAATCGAAATGGTCACCGGCGCTCCACCATGGAAAATTACCGACGACCCAGTTTCGGTGTTGTACCGGATCGGATATTCTGGCGAGTCTCCGGAGATTCCGAGCTTTCTGTCGGAGAAAGGAAAGGATTTTTTAAGGAAATGCTTGAGAAGGGAAGCAACAGAAAGATGGAGTGCGAGTCAACTTCTTGAGCATCCATTTTTGGGGGAATTGAGTTCTGGGTTGGAGGAAATCAAGGAATTACATTTATATTCTGAATCTCCGACGAGTATTTTAGATCAAAGCCTCTGGAATTCCTTAGAAGAAGAATCAGAAACTTTGTTGAGAACAGAGCAATGGGACGATGACAGAATCGAACGGCTGGCTACGTTTTCAGGAGAAATTAAGTGGGAATTGGGAGACGAGAATTGGATCACAATCAGAAGCTATGTAGATGGTGAAGACGAAGACGAATTAAATTTTAGTAGAAATGATTTAGACTTAGTGGAAGAGGGAAGAATAGTTAGTGAGGAAATCCAGTATTTGGAATTGTTAGATAAAACAGTTAGCTTTAGAGTAAGGTAG

>Csa7M430790.1

ATGAAGAGAAATTTAACATTGGGAGGATTAGAAGTTGAGGTAGAAGATGGCTCGTCGGAGTTCAATAATGGAGTCCAATGGAAACGAGGTCGGCTCATCGGAAAAGGAAGCTTTGGATCAGTTTTCTTGGCTTCTCTCAAACCGCACATCACTATTAAGTACTGCACTTTCCCTTCCGTAATGGCTGTTAAGTCCGCGGAAATTTCCGTTTCCGAAACCCTACAAAAGGAAAAGCAAAATTATGATAGCTTGAAAGGATGCAATTCATTGATCAAATGCTTCGGCGAAGAGATTACTACTGATCATAATGGTCACATGATCTATAATTTGTTGCTTGAAGTTGCCACTGGAGGAACCCTAGCTCACCATATTAAAAACACTGGTGGAAAGGGTTTAGAAGAAAATGTGGTTCGGAATTACACAAAATCAATAATCAAAGGATTGATTCACATTCATCGATCTCAATACGTTCATTGTGATTTGAAGCCTGCAAATATTCTATTGCTACCAAAGAATAATACAACAAAGGATCGCCAGTTCATTGCAAAGATCGCCGATCTAGGGCTGGCAAGAAGAACAAGCAAGACAAAGGCAAGCTATTGTTTAGGAGGGACATTCTCCTACATGGCGCCGGAGACGTTTATTGATGGCGTACAAGAATCAGCCAGTGATATTTGGGCACTTGGGTGTGTCGTGCTCGAAATGCTAACCGGGAACCGCGCTTGGGCAGCCACCAACAAGGTTGGGATTATGAAAGAGATGACGGAGAATTTCCTTGGAATGCCAAAGATTCCAGAAGGCCTATCAGCGGAGGCAACCATGTTCTTGAAGAATTGTTTTGTGAGGAAGCCGGAGTTCAGGTTCACGGCGGAAATGCTGATGATTGTGCCGTTTGTGGCGGCGGTTGAAGATCAAGAACAAAATTTTAATACTGTGAAAGCTCCAACATTTGTGACAAAGTGGCCTATGCAGTTTAAGAGACAAAGAATAATTCCAATCAAAGCGGTGTGA

>Csa6M490950.1

ATGGATTGGGTTCGAGGAGATGAGATTGGATATGGAAGTTTTGCTACCATCAATTTAGCCACGTCTTGTTCCGGTGATCGATTTCCTCCATTGATGGCCGTTAAATCATCTGGCCTTGTTTGCTCTGCCTCCCTCAAGAACGAGAAGCAAGTTTTGGATCTTCTTGGTGATGATTGCCCACAAATTATTAGGTGTTTCGGGGACAGTTGTAGTGTTGAGAACGGTGAGGAACTTTACAATCTGTTCCTCGAGTATGCTAGTGGTGGAAGTTTAGCCGATAGAATACAGAGCCGCGGTGGTCGGCTGCCGGAATTTGAGGTTCGACGGTACACGAGGACTATTCTTGAAGGGCTTCGGTATATTCACGGAAAGGGATTTGTTCATTGCGATGTTAAGCCTAGGAATATTCTCGTGTTTGGTGACGGTGATGCTAAGATTGCCGATTTCGGATTGTCGAAGAAGGCGGGAAAGAACAGAGTGGGAACAGGGGAAGAGACAGGGAAATTTCAATTGAGAGGTTCTCCTCTGTACATGTCACCGGAATCGGTCAACGATAATGAGTATGAGTCGCCGTGTGACATATGGGCCGTCGGATGCGCGGTGGTGGAGATGTTGACAGGGAAGCCCGCGTGGAATTGTCGGCCGGAATCCAACGTGTTTGCTTTGTTAATCAAAATTGGGATTGGAGAAGAATTGCCGGAAATTCCTAAAGATCTATCGGAAGAAGGGAAAGATTTTCTCAAAAAATGTTTGGTGAAGGATCCATTAAAGAGATGGACGGCCGATATGCTTCTAAAACACCCATTTGTGGCAGAGTCCGGGCGGTGTGTTCCATTGGCGGGCGTGGAGGAAGTATCAACGTCGCCAAGATGTCCTTTCGATTTCGAAGATTGGGCATCGATTCATTCACAAGAGAGCGATCCTAGAAATGAAGAGGAAGCAAATTGTTGGTTGAATAATTGGTCGTGTTCTCCAAGGGAGAGGCTGCTTGAACTAGTGGGGAATGGGGCAGTAGAGTGGTCGGTCACTGACAATTGGGTCAGGGTCAGATGA

>Csa2M278170.1

ATGGAGTGGATTCGAGGGGATCAACTCGGCCGTGGCAATTTTGCTACAATCAATTTAGCAAAGTTAACTAAAGGGTTCGATCAGTTCCCGCCATTAATGGCGGTCAAATCCTCTGTTTCTTCTCTATCCTCTGTTTCATCACTCAAGAACGAGAAGCAAATTCTTGATCGAATTGGGGTTTGCCCACAGATTATCACTTGTTATGGCGATGGATTCAGTGTTGAAAAAGATGGGGATAAGTGTTATAATTTGTTCTTGGAGTATGCTAATGGCGGAAGTCTTGCGGATGCTTTGAGAATCCATGGCGGTGGGTTGTCGGAATTTGACGTTCGAAGGTATACGAGAGCCATTCTTTGTGGACTACAGCATGTTCATGGCAATGGGTTTGTTCATTGCGACTTAAAGCTTTCGAATGTGTTGATTTTCGGGAATGGTGAAGTTAAGATCGCGGATTTTGGGCTTGCTAAATCGGCTGGAAAATTTGCGGCAGTGGAAACAGAGGAGAGATTTGAGTGGAGAGGGACTCCGATGTATATGTCGCCGGAGATTGTAAACGACGGTGAGTATGAGTCGCCGTGTGATATTTGGGCGTTGGGTTGCGCCGTCGTCGAGATGGTGGTTGGAAAGCCGGCGTGGAGGGTTGGACCGGAAACGGATATGTTTGGCTTGATGATGAGGATTGGAGTCGGAGATGAAGTACCGGAGGTACCAGAGAATTTATCGGCGGAGGGGAAAGATTTTATCCGGCGGTGTTTTGTAAAGGATCCAAGTAAGAGATGGACGGCGGAGATACTTTTGAATCACCCATTCGTCGCCGGTGCCGGTGACACTGTTACATTGAAGGAAGTAGAATTGGCGACGGAATCACCAACGGGGCCTTTCGATTTCCCGGAATTCGTTTGTTCCGGGCAAGGTTCGGATGAGTGGAGTTTTTGTTCTTCCAGTTCGTCGCCAGAGGTGTTGAGTAGGGTCCGGCAGCTGATGACGGGGAAGCCTTTGGATTGGTCTGTCATGGATAGTTGGGTGACAGTTAGGTGA

>Csa7M378450.1

ATGGAGAATTGGGTTCCAGTGAAGGTCTTAGGTCAAGGTTCTTATGCTGTAGTTTGCTTAGCAAAGCAATCCATTAGAAAATGTTCTGATAATAATCTTCCCTATTATTTTGCTCTCAAAATTTATCCTCTTCAACATAATTCTTCCTTGTTGTGGGAAGAACAAGTACTGAAACAGTTCAAGGGCTGTCCAGAAATTGTTCAATATTTTGGTAGTGAGATAACCAGAGGGGGAAGTTTTTGTAATGATAAGGATTTTTACACTTTGAAGTTGGAGTACGCTGCGGGAGGGACTTTGGATGACTTGATCAAACAAAGAGACAAGTTACCTGAGGATGAGGTGAAAGATTATCTTCGAATGATACTTAAAGGTCTCTCATGTATTCATAGTAAGGGATTTGTTCACGTTGATCTTAAGCCTAACAACATTCTTGCATTCCCTCAAAGTGATGGTAAGATGAAGTTAAAAATTGCTGACTTTGGACAGGCAGAGAGATGCAAGTATAGGGATGATAATGGTCAACATAAAAGGTACGGGTATTGCAGTTCATTGAAGTTTAAAGGATCACCAAGATATATGTCACCTGAATCCATCATCTTCAACGAAGTCGATGACGCGCACGATATTTGGTCGTTAGGTTGCATTTTGGTTAAAATGATATCTGGAAAGTGTGTGTGGGATGGTTATACAGATTCTAAACAATTGATGATAGAAGTTTTGGATAACAAGATTATGGCAACAATACCTGGGGAGTTATCCGAACAAGGTAAGGACTTCATTAGAAAATGCTTTATTCGAAGTTATAAACAACGGTGGACGGCTGATATGTTACTCCAACATCCCTATCTCAATCAAGAAAATGAAGCTCCAATGAAAGAGGATGAAGCTACGATGAAAGATGATGAAGCTACGATGGACGGCGGCTCATATTCTTTCAATAGATTGATTTTAAAATTCCCAATAGCAAAATTATTTTTAACTTGTTTTAACCAATAG

>Csa7M407720.1

ATGGATCAGGATTGGGTTCTAGTGAAGGCTTTAGGCGAAGGTTCTTGTGGCTTAGTTTGCTTAGCAAAACAAATCACTAAAGAAGAATCTGATCTTCATTATTATTTTGCTGTCAAGCGTGCTTCTCTTCGATATAATTCTTCTTCATTGTTGTGGGAAGAACATGTCTTGAAACATTTCACGGATTGTCCAGAAATTGTTCAATATCTAGGAAGTGAGGTAACTGGAGGAGGAGATTTTCTTGATGATAAGGAACTTTACAATTTGAAGCTAGAGTATGCTGCTGGAGGGACATTAGCTGATTTGATCAAACAAAGAAATAAGTTACCTGAGGATGAGGTGAAGAAGTATCTTCAAATGATTCTTAAAGGCCTTTCATGTATTCATCGTAAAGGATTTGTTCATGTCGATCTGAAGCCTGATAATATTCTTGCCTTTCCTCAAAGTGATGGAAAGATGAAATTGAAAATTGCTGACTTTGGACTTGCTGAGAGATCATGTAAACGTGGGGAGGATGATCAAGAAGACAGAGGGAGCAAGTACTACTCTGGCGCGTTGAAGGTTAGAGCAACACATAGATACATGTCACCTGAATCCATCGTTTTCAGTGAGATCAATGGTTTGCATGACATTTGGTCGTTGGGTTGCACTTTGGTTCAAATGGTTTCTGGAGAGCGAGTGTGGAATGATTGCAAAAGTTATGAAGAATTAATAACAAAACTTTTGATTAGCGAAGAAATACCAACAATACCTGAGGAATTATCCAAACAAGGTAAAGATTTTCTTGAAAAATGTTTTGTTCGAAACTATGAACAACGGTGGACAGCTGATATGCTACTCCAACATCCCTACCTCAATGAAGAAAATAAAGATACGAAGAACGGTGATGAGAAGCTTAAACTTCCAAAGGCCATAGTTTTTTTGCCTCATCAATTCTTTCAGAGCAAGACTGATATGCTACTCCAACAGACGAAGAACGGTGATGAGAAGCTTAAACCTTCAAAAGCCATTTTTTTGCCTCATCAATTCTTCAAGAGCAAGGCTCAATCTTGTAATAATTAG

>Csa3M829110.1

ATGACAAAATCCTGTGTTGTTTCACAAGTGCAAATCTCTGGCAACCTGCACGAATTCTTGAAAACAAAGAGAAGAATTCTGAAGATTGCAAAGAAATCGGCACCAAGAAACAATTGGAATGACACCATCAAACATAATTATGAAAAATTTGGCGAAGACTTCACAATCGAAGAATTCCATGGCTCAATTTGGACTGGTTCTCTTCTCTTTCGTAAAACAGAATCTACCCTAGTTTTCTTGGCCAAGAAGAACAGAGCACCCATCAACAACTCAAATTCAAATCTCCCTGAAGAATTTCTTGTCAAATCTTCTTTAATGGAGTCCTCTTCTTCGTTACGTCATGAGAAGGAAGTGCTATCCAATCTCGGACCATTCACTAATCTAGTGGATTGTTATGGAGATGAAATTACAGTAACCAAATCCGGAGAAGAAGTTTATAATGTCTTTTTCGAGTATTGTTCAGGGTCGAGCTTGCGTAATCACATTCTTAAATTTGGACCTAATGGGTTGCAAGATGATGAAGTTCGTAGATATACTAGAGATATCGTACGTGGACTTTATTACATGCATTGTAATGGTCGATACATTCATGGTGACATAAAATCAAGAAATATTTTGCTGTCACATGGCATGGCTAAGTTGGCTAGTTTTGGATTGGCGAGGAAGCTCACTGCTGAAGTAATATGTGAGGAGGAAATTAGTGGGTCGGGACCGTACGCATCTCCGGAGTTGGCTAGAGAAGGTTATTTGGGGTGGCCTGCTGATATTTGGGCACTTGGATGCGTGGTGTTGGAGATGTTTACGGGAAAATCTGCATGGAGCTTTGAAGATGCATATAGATATCTGATGGATTCTAACAATGAGAAGATACCAGAAATTCCTAAGAACATATCAAGGGAAGGAAGAGATTTTATTAAGAAGTGTTTGATTAGAAGTCCTTATAAAAGAAGACCAATTTGGTTGTTGATCAAACATCCTTTTGTTTGCCAATGA

>Csa6M450400.1

ATGGAAATGCCTGGACGGAGGTCAGATTACTCGCTTTTAAGTCAAATTCCGGACGAGGAAGTTGGAACGGGAGTTTCCACTTCCTTTTACGACTCTATAGCAGCTGGGGGAAACGTTATTAAAGGGAGAACCGATAGAGTTTTTGATTGGGATGGGATTGGTGATCACAGGTTAAACACGCAGGCGTATCGAACAGGGAACCTGTATTCATGGATTGGTTTGCAGAGGCATTCCAGTGGAAGCAGCTACGATGATAGCTCTCTCTCTAGTGATTACTACGCACCGACGCTATCAAACGCTGCAGCAAATGAGATCAATGCATTGGAATACATCCACGATGATGATTTCAGAGTGATGAAAGCTGTGGGAAGTGGAGGTTCGTCTGGAAAGAGCTGGGCCCAGCAGACGGAAGAGAGTTATCAGTTGCAGCAGGCCTTGGCTCTTAGGCTTTCTTCAGAGGCTACTTGTGCTGATGATCCCAACTTTATGGATCCGTTGCCAGACGAGGCAGCTTTAAGGTCGTTATCCATTTCAGCTGAGGCCATCTCGCATCGATTCTGGGTAAATGGATGTATGTCATATTTTGAGAAAGTGCCAGATGGTTTTTATCTAATTCATGGGATGGACCCATATGTATGGTCATTATGCACCAATCTGCAAGAGGATGGGCGTATACCATCATTTGAATCTCTGAAAACTGTTGACTCCAGCATCGCTTCATCAATTGAAGTAGTTTTGATAGATCGGCATAGTGATGCTAGCTTAAAAGAACTGCAAAACAGGGTGCATAATATTGCTTCTAGTTGTGCAACTACAAAAGGGGTTGCAGATCATTTAGCGAAGCTGGTATGCAATCACTTGGGGGGTTCAGTTTCTGAGGGAGAAGATGACTTGGTTTCTTCCTGGAAGGAATGCAGCGATGACTTAAAGGAATGTTTGGGATCTGCCGTTATTCCCTTATGCAGCTTATCTGTTGGCCTTTGCAGACATCGTGCTCTTTTATTCAAAGTCCTAGCTGATTCAATTGATTTGCCCTGTCGAATTGCTAGGGGTTGTAAATATTGCACTAGAGATGATGCTTCATCTTGCCTTGTTAGGTTCGGGCTTGATAGGGAATATCTCATTGATCTGATTGGGAGGCCAGGTTGCTTATGCGAACCTGATTCTTTGCTCAATGGTCCATCATCCATCTCAATTTCTTCACCATTGCGATTTCCAAGACTAAAACCTATTGAATCTATCATTGATTTCAGGTCACTGGCCAAACAGTATTTCTTGGATAGCCAATCACTTAATGTTGTATTTGATGAAGCTTCTTCAGGGAATGTTGTATCTGGGAAGGATGCTGCGTTCTCTGTCTATCAGAGGCCATTAAATAGGAAGGATGGAGATAGAAAAATCATAGTGGTTACTGGTGACAAGGACAGAAATTCTCAGTTATTGAATAAAAAAGCAGCCCAACTGAATACTCAAGATGGAAAGTCTGAGCAATTTAGATCATGTGTTACTTCTCAATATAGTGTACAGTCGACCCCTTTAGTAGAAAACGTAGTCCCTTTGAACCATATCTCACCCATTGGTTCCAAAGATTCTGAGCATCTCTTAGCATTGTCTCATCCAAGGGTGGATCATGCTAACAATTTACCATTTGTTGACGGTAGTCAGTTGATTAGAAAACCAAATGATCTTTCCCTTGGCTTAGAAGATTTGGTTATTCCATGGAAAGATCTTGATTTGAGAGAGAAAATTGGAGCAGGTTCTTTTGGGACTGTATATCATGCTGATTGGCATGGCTCAGATGTTGCTGTGAAGATTCTGATGGAACAAGACCTTCATGCAGAACGTTTTGATGAATTTCTTAGGGAGGTTGCCATAATGAAATGTCTACGACATCCAAACATCGTTCTCTTTATGGGTGCAGTCACAGAGCCTCCAAACTTGTCCATTGTAACAGAATACTTATCAAGGGGTAGTTTGCACAGGCTTTTGCACAGACCTGGGGCACGTGAAGTCTTAGATGAGAGACGACGGTTAAACATGGCATATGATGTTGCAAAGGGAATGAATTATCTTCACAAACGTAATCCCCCAATTGTTCATCGCGATTTGAAATCACCAAATCTTTTGGTTGATAAGAAGTACACTGTGAAGGTTTGTGATTTTGGGCTTTCACGCTTGAAGGCACACACATTTCTTTCGTCAAAATCAGCTGCTGGGACTCCTGAGTGGATGGCACCAGAAGTTCTCCGTGATGAGCCATCAAATGAGAAATCTGATGTTTATAGTTTTGGTGTGATACTATGGGAGCTTGCTACATTGCAGCAACCATGGGGCAACATGAACCCACCGCAGGTTGTGGCAGCTGTTGGTTTCAAAGGTAAAAGGCTTGAGATCCCATGTGATTTAGATCCTCGAGTTGCTACTATTATAGAGGCTTGCTTTGCCAGTGAGCCTTGGAAACGCCCTTCCTTTTATGAAATAATGGAATCGTTGAAGCCATTGATCAAACCTGCTACACCTCATCAAGTTCGCTCAAACGTGTCATTAGTTACTCAGTGA

>Csa3M749850.1

ATGGAAATGCCCGGCAGGAGGTTGAACTATACGCTTCTAAGTCAAATTCCAGACGACCAATACAGTGGCGGCGTTGCCGGAGCTTCGACATCTTTCATAGAAACGTCATCGGGAGAGGGGAAGAACGATAGGAGGAAGTTAGAAAGAGGATTGGATTGGGAAGTTGCTGGTGATCATAGGGCGGGCCAGCAGCAGCAGGTTAATTGGATCGGCAATATGTACTCGGCGTTTGGTTTGCAGAGGCAGTCCAGTGGAAGCAGCTTTGGAGAGAGCTCGATATCCGGGGAATACTATGCACCGACACCATCTACTACGGTGGCTAACGAGACAGATGTATTTGGTTGTACGCACGATGATGTGCTTAGAGTTGGAGGAGATTCGAGAGCACAGGCAGGCGAGATGGCCGCTGGGGCTGGAGGTTCTTCTGCTAAGACCTGGGCCCAACAGACGGAGGAGAGTTATCAGCTGCAGTTGGCATTGGCGCTACGGCTTTCATCTGTGGCGACTTGTGCTGATGATCCCAATTTCTTGAATCCATTTCATGATGATTCAGCTTTGAGGAGGCTGATAGGTTCTGCTGAGAGTGTGTCACATCGATTTTGGGTGAATGGCTGTCTATCGTACTTAGACAAAGTCCCTGATGGGTTTTATCTAATCCATGGAGTGGATCCGTATGTATGGACTGTGTGCACTAGCCTGCCTGACAATGATCACATGCCATCAATTGAATCTCTAAAGTCTGTTGATCCCAGTACAGATTCGTCAATTGAAGTTGTTCTGATTGATCGGTGCACTGATCCCAACTTAAAAGACCTCCAAATTTGGGTCCAAAACATTTCTTGCAGCTCTATAACTACAGAAGAGGTTGTAGACCAGCTTGCAAAACTTGTCTGCAGGAGTTTGGGTGGCTCAGTTTCTGGAGAAGACGCGTTGGTGTCCATCTGGAAGGAGTGCAGTGATAACTTGAAGGAGACCTTAGGCTCAGTGGTTATTCCTCTTGGTGGACTTTCTGTAGGACTCTGCCGGCATCGTGCTCTTCTTTTCAAAGTGTTAGCTGACACAATTGATTTACCCTGTCGTGTAGCCAAGGGGTGTAAATATTGCTCATGCCATGATTCTTCTTCTTGTCTAGTTCAGTTTGGGCTTGACAAGGAGTATCTGGTTGATTTAATTGGCAATCCTGGTTGCTTATATGAGCCTGATTCTTTACTCAATGGTCCATCCTCCATTTTGATCTCCTCACCATTGCGATTTCCACGTCTAAATCCAGTAGAGCCTGCCACCGATTTCAGGTTACTGGCTAAACAGTACTTTTCAGATTGCCAGTTGCTCAATGTTGTTTTCGATGAAGCTTCTTCATGTAATCATTCAGAAATTACTGTGGATGGAGAAGATGGCGCACTTCCATTGTATCCAAAGCAGTTTGATAGGAAATTCACAAACAGAAGCAACCAAATGCTTGTCACTGGTGATAGTGATGAAAAATCCATTTTACTGCATCCAAAAACTTCACAGCCTAACTCCCATGACAGAGATTTCCAACTGTATAAACCACGTGATAATTCTCATAGTGTTATTCAACCAACTGTCCTGGTTGAGGATTCAATCCCACTGAAGTACATTCCACATAATAACCGTGGAAGCATGCAGTCACTTTTGGATATGTCCCAGCCAAGGATGGATTCTACTATGGATGTAAGGTTTGCAGCGGGAGGTCAGCTAATACCAAGCAATCGAAGTAATACACTTCCCCTTGGTGCGGAGGATTTGGACATTCCATGGGGTGATCTTGTTTTAAAAGAAAGAATAGGAGCAGGTTCCTTTGGAACTGTGCATCGTGCTGATTGGCATGGATCGGAAGTTGCTGTGAAGATCCTCACAGAACAGGACTTCCATCCTGAACGTGTTAATGAGTTTCTGAGAGAGGTTGCTATCATGAAATCTCTACGACATCCTAATATTGTACTGTTTATGGGTGCGGTGACCAAGCCACCAAACCTGTCCATTGTAACCGAATATCTATCGAGAGGTAGCTTGTATAGGCTTTTGCACAAGTCAGGTGTCAAAGACATAGATGAAACACGTCGGATAAATATGGCTTTTGATGTGGCAAAGGGAATGAACTATCTCCACAGACGTGATCCTCCAATTGTTCATCGTGATTTAAAATCACCAAATCTTTTAGTTGACAAGAAGTATACAGTAAAGGCACGCACATTTCTTTCATCCAAATCTGCAGCTGGAACGCCTGAATGGATGGCACCAGAAGTACTACGTGATGAACCATCAAATGAAAAGTCAGATGTTTATAGCTTTGGAGTGATTTTGTGGGAGTTGGCAACTTTGCAACAGCCATGGTGTAATCTAAACCCAGCTCAGGTTGTCGCAGCTGTTGGATTTAAGGGCAAAAGGCTTGACATCCCACGTGATGTAAATCCCAAATTGGCTTCCTTAATAGTGGCTTGCTGGGCCGATGAGCCGTGGAAACGTCCATCTTTTTCCAGCATAATGGAAACCTTGAAACCAATGACTAAACAAGCACCACCTCAACAAAGTCGTACAGACACACTCTCAGTTATGTGA

>Csa1M574260.1

ATGAAACACATTTTCAAGAAGTTTCATATAGGAAGCAATCACGAGCCGAATCGGTCCAACGAGAATCCGTCGCCTGTAGCAGCAGCGTCGTCTTCACCATGTGTTTCTGATAACCGTCCTGCAACTGCTCCGGGTCAGACTTCCGGTAACTCTCCCCCCAGTCCTTCATCGTCGCCGTCGCTGGCAACAACTTCTCCAGGGGGTGGAAATGTAACTCAGGTTTCGGTACCTCCCAATCGGTCCGATTACTTTTCTTCAGAGGAGGAGTTTCAGGTTCAGCTTGCCCTAGCTATTAGCGCATCCAATTCGGACTTCCGGGATGATCCGGAGAAGGATCAAATTCGAGCTGCGACACTTTTGAGCTTGGGAAATCATCGGATTGATTCTACTGCCAGGGACCAGGGAGATGCTGCCGAGGTGCTCTCGAGACAATATTGGGAATACAATGTGCTTGACTATGAAGAAAAAGTGGTCAATGGGTTTTATGATGTTCTTTCTACGGATTCAGCAGTCCAAGGAAAAATTCCATCGCTATCTGATATTGAAGCAAGCTTTGGTAGTTCTGGCTTTGAAGTTGTGATGGTCAATATGACTATTGATCCTGCTCTAGAAGAGCTAGTGCAAATTGCTCAATGTATTGCAGATTGCCCTGGGACTGAGGTCAGGGTTTTGGTTCAGAGGCTTGCCGAGCTTGTTATGGGACATATGGGGGGACCTGTAAAGGACGCCCATTTTATGCTAGCAAGGTGGATGGAAAGAAGCACAGAGTTAAGGACTTCTCTTCACACCAGTGTATTGCCTATCGGTTCCATTAATATTGGCCTCTCAAGACATCGTGCATTGCTTTTCAAGGTGTTAGCTGACAGTATCAAGATGCCTTGTAGGCTTGTTAAAGGTAGTCACTATACCGGTGTTGAAGAAGATGCTGTCAACATTATAAAGTTGGAGGATGAAAGGGAGTTTTTGGTTGATCTAATGGCAGCTCCTGGAACACTTCTACCAGCAGACATATTTAATGCAAAGGACACTACTAACTTTAAGCCTTACAACCCTAAAGTAAGCAGAATTCCTTCTCTTCACCACTCCAATGATGTTGGAATTTCTTCTGCGAAACCAACATCGGGACTTGAGGAAGGCAGCAGTCAAAACTTTGGAGCAGAGGCCATCTCACTGATGGATGGGAAACTGGGCTATGGAAGGACAGAGTCTGTGCCATCAAGCTCAGGTACTGGGACTTCTCGATACAAAGGGGCCCATTTTGGTGACGGTAATGTTCGACTGAATGTCAATGTAGTTCCATTTGGTCAAAGTTCCGAGGATTCCAAAAATCTTTTTGCGGATCTTAATCCTTTCCTAATAAGAGGAACTGGAAAAAGTTTCATTCCTAACAAGTTCTCAGATAATAAAAGTGAGGAGCTCCAGAAACCTACTATTGGGCATCCTCCTGTACCATTATGGAAAAATCGGTTTGCTTTTAATGCAGTTCCCAATAAAAATGAGTACGATTATATGGAGGGCCGTTTTCCAAGAATCAGCCGTGGGCCTAATGATCAAAACATGGCATTATCTTCTTCCAATTCTACTGGCTCTGAAAGTGTTAAACCTGGTGGTTCAGGAACATCTAATGACTTGAGTGCATCGGTTAGAAGTGCTGAAGTTGGAAGTTCTTCATCAAATATGTACGCACAGCCGGCATTTGGAATGATGGAGCCTAATATTTTGCCCTTCATTGACGAACAGAACAGAAAGTCCAATGGAGAACATTCTGGAAATACAGACATGGAGGATGAGAAGGTAGATGCTGTTGATGGACGAGACAATTTAATCAGATTTGATAACCGTAGAAAGTTCACATACGAGAGATCTGTTGGAACCAATTTGATATTGAAGGATTCGGGAAATCCTGGCCTATTGGTCAATCCAAGTTCAAATAGGTTTGAGCAAGTTTATGATGATGTGGACGTAGGTCAATGTGAAATCCAATGGGAGGACCTCGTTATTGGTGAAAGGATTGGACTAGGTTCATATGGAGAAGTCTACCATGCTGATTGGAATGACACAGAGGTTGCTGTGAAGAAATTCTTAGACCAGGATTTTTCTGGTGCTGCTTTAGCTGAGTTCAAAAGAGAAGTATTGATAATGCGGCAGCTGCGTCATCCGAACATTGTTCTTTTTATGGGTGCTGTCACTCGTCCTCCCAACCTTTCCATTGTTACTGAGTTTCTTCCAAGAGGAAGCTTGTACCGGATCATTCACCGTCCAAATTGTCAAATTGATGAAAAGCGCAGAATAAAAATGGCCCTGGATGTGGCAAGGGGCATGAATTGCTTGCATACAAGTAATCCAACAATTGTTCACCGAGATCTGAAGTCACCAAATCTTTTGGTTGATAAGAACTGGAATGTGAAGGTGTCAGATTTTGGGTTGTCACGCCTAAAGCACAATACATTTTTATCATCCAAATCAACTGGAGGAACGCCTGAGTGGATGGCACCAGAAGTTCTTCGGAATGAGCCTTCAAATGAGAAGTGCGATGTTTATAGCTTTGGAATCATTCTATGGGAACTTGCTACATTGAGACTGCCTTGGAGTGGGATGAATCCTATGCAAGTTGTTGGAGCTGTAGGTTTCCGTAATCAGCGACTCGAAATACCCAAGGAAGTGGATCCCACGGTTGCAAGGATAATCTGGGAATGCTGGCAAACTGATCCGAACCTGCGTCCCTCATTCTCACAATTAGCCAACATTCTGAAGCCACTGCAGCGGCTCGTCCTCCCACCACATTCGGACCAGCCAAGTTCGTCAGTACTGCAAGAGATCTCTGTAAATTCTACACCTTAG

>Csa4M646020.1

ATGAAACATCTTCTTCGAAAGCTTCACATCGGTGGAGGACTTAACGAGCATCAGCGATTGAGCGATGCTCGACCTGTAACACGACCAAGTTCGAGCCCCAGTCCAGGCCCAAGCCCCAATAGTAATCCATCGGGTTCGTCGTCATCTGGGTCTTCTTCGTCTTTGTCTATGGCGTCTTCTACTACAATGGGGAGATTGGAAGCTGTTGAATCAGTCGTTGATCCGGCGGCTTCTGGGGATGTGGGCGGTGGGTGCGTGGATTTCAATGCCTTGGAAGAGGAGTTTCAGGTGCAATTGGCTATGGCGATTAGTGCTTCCGATCCTGATTCTAGACAAGATACTGAATCAGCTCAGATCGATGCTGCCAAGCGGATGAGTCTTGGCTGTTCGCCTTCAGTCTCCGGCAGTAAAGCCCTCGCTGAATTCCTCTCGCTTCAGTATTGGAGCTATAATGTTGTAAATTATGACGAGAAAGTGATGGATGGATTTTATGACTTATATGGCATTACTGCAAGTTCTAGCACCCGTGGAAAGATGCCACTGTTAGTTGATCTTAAAGAAATATGTGTGACGAGTGATATTGATTATGAGGTGATTTTGGTAAACCGCTTGCTTGATCCTGAGCTTCAACAGCTGGAGAGACAAGCATATAACATATTCATGGAATGCAGAGTTTCTGAATATGGCTTCATTTTAAGTGGCCTGGTTCAAAAAATTGCTGATATGGTTGTTGCTAGAATGGGTGGCCCTGTTGGTGATGCTGAAGAAATGTTGAGAAGGTGGACACGCAGGAGTTATGAGATGCGTAGTTCTCTGAACACTATTATTCTACCACTTGGTCGTCTTGATATTGGACTTGCACGTCATAGAGCCTTGCTTTTTAAGGTGCTAGCTGATAGGATTAATCTTCCATGCATACTTGTCAAAGGAAGCTACTACACTGGTACTGATGACGGAGCTGTGAACATGATCAAAATTGATAATGGAAGTGAATATATTATTGATCTTATGGGTGCTCCGGGCACGTTAATTCCCTCAGAGGCGCCCAGCGGTCAATTTTCGAATTATGGATTTGATAGAAGGCCAGCAGATGTTATAGAAGTTCCCGAAGATACTCCAATATTACAAAATGAAGGAGCTGAAGCAGTGTCAATTTCATCTACTCAAGATGAAGTAGCAGATGTTTGCAACTTAATATCCAAAGAAGCTTCAGATTTAGATGCTCAATCAAAAGAAAACATTAGGAACTTTATTGAAGAAATTCAAAGTGGGAGTTCCGGTTATGATTTTGCAAAGCTTCTAGAATCAGAAAGCAGTGCCTGTGAAGGTTCATTGGGTGCATTTGCACAGTCCGCTTCTGCACAAAAGAAGAAAGTGAAAAAAGTGTCAAAGTATGTCATAAGTGCTGCAAAGAACCCAGAGTTTGCTCAGAAATTACATGCTGTCTTGTTAGAGAGTGGTGCGTCACCTCCTGCAGATTTGTTTTCAGATATAGAATCACAGGACAACGGTGAAAGCAAAGAAACTTTTCAAATGTATCCAATCAATGGGAAAGGGATAGATGTTGGGCTCCAGAGCCACTCCTATATTTTAGCAAGCCACGGACAGTCTTCTGCTACTTCTACTGAAGCGGAGTATTTGAACAACGTTGTCCATGAAAATAAACAGAAAGTGCCTTCTGGGGGATTGTCTGAAGAACAAATGGCCAATACAAATGCCAACAACCACAGCATTTTTTGGCCTCACAGTATGAAAAACGAGGGATTTGTGTTTGTTGATGTTAATGGTGAAGCAGGAAAATTGGTTGATGTTAATGGAACTTTTCATCGTGAACATATGGATGATGTTTTGTTGACATCTGATACTGACTCTCATAAGAAACTAGGAAGTGCCTTAGTGAGTGAAGAAAGAAGATTGTTGCAAGATAAGAGCGGTGGAACTCTTCAATGCTTTGATTTGTGTGAAAAGCCACTTGAAAATTTGTTACAAACTGATGATAGTAAACTGCATGCTAGTGATGAGCACAATGAGACAATTAACCCAATATTAGGTGAAGTTGCAGAATGGGAAATTCCTTGGGAGGATCTTCACATTGGTGAACGTATTGGTATTGGCTCATATGGCGAGGTTTATCGAGCAGATTGGAATGGCACTGAAGTTGCTGTGAAGAAGTTCTTGGATCAAGATTTTTCTGGTGCTGCTTTGGTTCAATTAAAATGTGAAGTTGAAATCATGTTAAGGTTGAGGCACCCTAATGTTGTCCTTTTCATGGGAGCTGTGACTCGCCCTCCTCATTTCTCTATCCTTACAGAGTTTCTTCCCAGGGGAAGTTTGTATAGGTTACTGCATCGGCCCAACTCTCAGCTAGATGAAAGGAGACGATTGAAAATGGCTCTTGATGTGGCCAAAGGAATGAACTACTTGCACACAAGTCATCCGACTATTGTTCATAGAGACCTAAAGTCCCCCAATCTCCTTGTTGACAAAAACTGGGTTGTCAAGGTTTGCGATTTTGGTTTGTCACGTGTGAAGCAGAATACATTTCTGTCTTCAAAATCAACTGCTGGAACGCCTGAATGGATGGCACCAGAAGTTCTAAGGAATGAACCAGCCAATGAAAAGTGTGATGTGTATAGTTTTGGTGTAATATTGTGGGAGTTGACGACATGCCGCATACCGTGGAAAGGTTTGAACCCAATGCAGGTCGTAGGAGCTGTTGGATTCCAGAATAGGCGCCTGGAAATCCCACAAGATGTTGATCCAGCAGTTGCACAGATCATTTGCGATTGTTGGCAAACGGACTCACAATTGAGGCCGTCGTTTTCTCAGCTTATTACTCGATTGCGGCGCCTGCAACGGCTTGTTCAGAAAACAGACTCCGGGAATCAAATATCGGAGTGA

>Csa1M042730.1

ATGAAGAACCTCCTTAAGAAGTTCCACATCATGTCCAGTGGTCAGTCTGACGATGTAGCTGAAGGGTCTACTTCATCGAGGAGCAATAAAGTAATGGAGGTTTCATCGCCTGATAAGCTGCCATCTCGCTCCCGGCCGACCCATTTCAGTTCGGAGCATAAACCCTTCTCGGGAATATCCGGTTGGTTGAACTCTGTTACAAATAGGCGTAGCCCCAGTCCTCCATCATCTGCGGATCCTACTGCAGGCGAGATAATGGAGCCATCGGATTCAGTATCTAGCAGAGACGCTGCAATGGATACATCTAGGCATGATTCAGGGTCGAGTAATTCGAGGGATCCTGATATAGAAGAAGAGTATCAGATACAGCTTGCTTTGGAAATGAGTGCTCGAGAGGATCCTGAAGCGGCTCAGATTGAGGCTGTGAAGCAGATCAGTTTGGGGTCATGTGATCCTGATAACACTCCAGCTGAAGTTATTGCCTTCAGATATTGGAATTACAATTCTCTTAGCTATGATGACAAGATCTTGGATGGCTTCTATGACCTATATGGAGTCTTCACCAGATCCACTTCAGAAAGAATGCCTTCCCTAGTTGATCTGCAAGGAGCACCAATGTCTGACAGTGTCACTTGGGAAGCAGTTCTGATAAATAAAGCTGCTGATGCTAACTTATTGAAACTTGAACAGACAGCATTGGAGATGGCTATCAAGATGCAGACAGAGTCTCCAATTTCTGTAAATCATTATTTGGTGCGGAAACTTGCTGCTTTAGTTTCAGATCATATGGGGGGACCAGTGGGTGATCCTGAAAAGATGTTGAGAAAATGGAGAAATCTTAGTTACAGCTTGAAAGCAACCCTTGGGAGTATGGTTTTGCCACTTGGCTCCCTTACTGTTGGGTTGGCCCGTCATCGTGCATTGTTATTCAAGTTTTTGGCAGATGGCGTGGGTATTCCTTGTAGGTTGGTGAAAGGACCACAGTACACAGGTTCTGATGATGTGGCAATGAACTTTGTAAAGATTGATGATGGAAGGGAGTACATTGTGGATTTAATGGCAGATCCTGGTGCACTTATTCCAGCTGATGTTGCTGGATCGCATGTAGAGTATGATGGATCTCCTTTTTCGGCCAGTCCAGTTTCTAGAGATGTTGATTCCTCTCAGGCAGCATCTTCAAGCAGTGGGGTTGGCAGTTCATTAGAGGGAAATTCAGATTTTGGGATATCAGATAGGAAACCAAAGGCCCGCAATCTCAGTGCCACAAAGGAATACGATTCTCCCAATATCGACAAGGTCCCATCACGTGATTTTGCTAGCAAATCAAATTATCCTGGTATGCACACTAGATCACCATCTTGGACTGAGGGTGTTAGTTCACCTGCAGTGCGCAGAATGAAAGTAAAGGATGTTTCACAATACATGATTGATGCTGCCAAAGAGAATCCACGGTTGGCTCAGAAGCTTCATGACGTGTTGCTTGAAAGTGGTGTAGTTGCTCCCCCAAATTTGTTTACCGAGGCATACCCTGACCAAATAGATGTTATTGTCGAGTCTAAGTCACCAACTGAAGACAAGGATCAAAGTAGAAAGCTGCCTGGTATCTGTGAAAGTGCAGATAAGAATGATCCTCGTCTATCTAACTTTTTGCCTCCCTTGCCTCAGCCCAGACTGCATTCTAGAGCCAGCCCTACCCATGGCCAACAACTGTACATTAAACCTCTGGAATTCAACCTTTCTCTTGATTCAAGAGAAGCAGGCGGACAACCTATACCCTTACCATTTGAGGTAACTCCAGTGAAGTATGGGAGAAATGTACCTGTTGCTGCAGCAGCAGCAGCTGCCGCAGCTGTTGTTGCATCGTCAATGGTGGTAGCTGCAGCAAAGAGTAGCGATGCAAATCTTGAAATTCCTGTAGCAGCTGCTGCCACTGCAACCGCTGCTGCAGTGGTTGCAACAACTGCAGCAGTCAACAAGCAATATGAGCAGGTTGAGGCTGATGCTGCTCTTTATGAGCTGCGTGGAAGTGGAGATAGGGAGCATGATGCCTGTGGAGACAATTCAGAGGGTGAACGTATATCAGATAGATCAGCTGGTAACGAAAGCACAAAATCTGACATTACCCTTGATGATGTTGCAGAATGTGAGATCCCATGGGAAGAAATTTCCTTGGGAGAGCGTATTGGACTTGGATCGTATGGGGAGGTCTATCGTGGAGATTGGCATGGAACTGAGGTAGCTGTGAAGAGGTTTCTAGACCAGGACATTTCTGGTGAATCTCTTGAAGAATTCAAAAGTGAGGTTCGAATTATGAAAAGGCTTCGGCATCCAAATGTTGTTCTCTTCATGGGAGCTGTGACACGTGCTCCCCACCTTTCAATTGTTACAGAATTTCTTCCCAGAGGTAGCTTGTACAGGTTAATTCACCGTCCCAACAATCAATTAGATGAACGAAAGCGATTGAGGATGGCTCTTGATGCAGCTCGGGGAATGAACTATTTGCACAACTGCACGCCTGTTGTTGTACATCGTGATTTGAAGTCCCCAAATCTACTTGTTGATAAAAATTGGGTTGTGAAGGTATGCGATTTTGGTTTATCAAAAATGAAGCATAGCACATTTCTTTCTTCAAGGTCAACTGCTGGAACCGCTGAGTGGATGGCTCCAGAAGTGCTGAGAAATGAACCTTCCGATGAAAAGTGTGACGTTTATAGTTATGGAGTCATACTATGGGAGCTTTCTACAATGCAACAGCCATGGGGAGGAATGAACCCAATGCAAGTTGTTGGTGCTGTTGGCTTTCAGCATCGCCGTCTTGACATCCCAGACAATTTGGATCCTGCCATTGCTGACATTATTAGGAAATGCTGGCAAACAGATCCAAGATTAAGACCTTCATTTGCCGAGATTATGGCTGCACTAAAGCCGCTGCAAAAGCCATTGTCGAGTTCTCAAGTACCGAGACCAAATGCTCCTGCAGGTAGTGGTCGTGACAAGGCTAGATTATTGCAAGTCACCGAAGACCCATCAGGCTAG

>Csa1M042730.2

ATGAAGAACCTCCTTAAGAAGTTCCACATCATGTCCAGTGGTCAGTCTGACGATGTAGCTGAAGGGTCTACTTCATCGAGGAGCAATAAAGTAATGGAGGTTTCATCGCCTGATAAGCTGCCATCTCGCTCCCGGCCGACCCATTTCAGTTCGGAGCATAAACCCTTCTCGGGAATATCCGGTTGGTTGAACTCTGTTACAAATAGGCGTAGCCCCAGTCCTCCATCATCTGCGGATCCTACTGCAGGCGAGATAATGGAGCCATCGGATTCAGTATCTAGCAGAGACGCTGCAATGGATACATCTAGGCATGATTCAGGGTCGAGTAATTCGAGGGATCCTGATATAGAAGAAGAGTATCAGATACAGCTTGCTTTGGAAATGAGTGCTCGAGAGGATCCTGAAGCGGCTCAGATTGAGGCTGTGAAGCAGATCAGTTTGGGGTCATGTGATCCTGATAACACTCCAGCTGAAGTTATTGCCTTCAGATATTGGAATTACAATTCTCTTAGCTATGATGACAAGATCTTGGATGGCTTCTATGACCTATATGGAGTCTTCACCAGATCCACTTCAGAAAGAATGCCTTCCCTAGTTGATCTGCAAGGAGCACCAATGTCTGACAGTGTCACTTGGGAAGCAGTTCTGATAAATAAAGCTGCTGATGCTAACTTATTGAAACTTGAACAGACAGCATTGGAGATGGCTATCAAGATGCAGACAGAGTCTCCAATTTCTGTAAATCATTATTTGGTGCGGAAACTTGCTGCTTTAGTTTCAGATCATATGGGGGGACCAGTGGGTGATCCTGAAAAGATGTTGAGAAAATGGAGAAATCTTAGTTACAGCTTGAAAGCAACCCTTGGGAGTATGGTTTTGCCACTTGGCTCCCTTACTGTTGGGTTGGCCCGTCATCGTGCATTGTTATTCAAGTTTTTGGCAGATGGCGTGGGTATTCCTTGTAGGTTGGTGAAAGGACCACAGTACACAGGTTCTGATGATGTGGCAATGAACTTTGTAAAGATTGATGATGGAAGGGAGTACATTGTGGATTTAATGGCAGATCCTGGTGCACTTATTCCAGCTGATGTTGCTGGATCGCATGTAGAGTATGATGGATCTCCTTTTTCGGCCAGTCCAGTTTCTAGAGATGTTGATTCCTCTCAGGCAGCATCTTCAAGCAGTGGGGTTGGCAGTTCATTAGAGGGAAATTCAGATTTTGGGATATCAGATAGGAAACCAAAGGCCCGCAATCTCAGTGCCACAAAGGAATACGATTCTCCCAATATCGACAAGGTCCCATCACGTGATTTTGCTAGCAAATCAAATTATCCTGGTATGCACACTAGATCACCATCTTGGACTGAGGGTGTTAGTTCACCTGCAGTGCGCAGAATGAAAGTAAAGGATGTTTCACAATACATGATTGATGCTGCCAAAGAGAATCCACGGTTGGCTCAGAAGCTTCATGACGTGTTGCTTGAAAGTGGTGTAGTTGCTCCCCCAAATTTGTTTACCGAGGCATACCCTGACCAAATAGATGTTATTGTCGAGTCTAAGTCACCAACTGAAGACAAGGATCAAAGTAGAAAGCTGCCTGGTATCTGTGAAAGTGCAGATAAGAATGATCCTCGTCTATCTAACTTTTTGCCTCCCTTGCCTCAGCCCAGACTGCATTCTAGAGCCAGCCCTACCCATGGCCAACAACTGTACATTAAACCTCTGGAATTCAACCTTTCTCTTGATTCAAGAGAAGCAGGCGGACAACCTATACCCTTACCATTTGAGGTAACTCCAGTGAAGTATGGGAGAAATGTACCTGTTGCTGCAGCAGCAGCAGCTGCCGCAGCTGTTGTTGCATCGTCAATGGTGGTAGCTGCAGCAAAGAGTAGCGATGCAAATCTTGAAATTCCTGTAGCAGCTGCTGCCACTGCAACCGCTGCTGCAGTGGTTGCAACAACTGCAGCAGTCAACAAGCAATATGAGCAGGTTGAGGCTGATGCTGCTCTTTATGAGCTGCGTGGAAGTGGAGATAGGGAGCATGATGCCTGTGGAGACAATTCAGAGGGTGAACGTATATCAGATAGATCAGCTGGTAACGAAAGCACAAAATCTGACATTACCCTTGATGATGTTGCAGAATGTGAGATCCCATGGGAAGAAATTTCCTTGGGAGAGCGTATTGGACTTGGATCGTATGGGGAGGTCTATCGTGGAGATTGGCATGGAACTGAGGTAGCTGTGAAGAGGTTTCTAGACCAGGACATTTCTGGTGAATCTCTTGAAGAATTCAAAAGTGAGGTTCGAATTATGAAAAGGCTTCGGCATCCAAATGTTGTTCTCTTCATGGGAGCTGTGACACGTGCTCCCCACCTTTCAATTGTTACAGAATTTCTTCCCAGAGGTAGCTTGTACAGGTTAATTCACCGTCCCAACAATCAATTAGATGAACGAAAGCGATTGAGGATGGCTCTTGATGCAGCTCGGGGAATGAACTATTTGCACAACTGCACGCCTGTTGTTGTACATCGTGATTTGAAGTCCCCAAATCTACTTGTTGATAAAAATTGGGTTGTGAAGGTATGCGATTTTGGTTTATCAAAAATGAAGCATAGCACATTTCTTTCTTCAAGGTCAACTGCTGGAACCGCTGAGTGGATGGCTCCAGAAGTGCTGAGAAATGAACCTTCCGATGAAAAGTGTGACGTTTATAGTTATGGAGTCATACTATGGGAGCTTTCTACAATGCAACAGCCATGGGGAGGAATGAACCCAATGCAAGTTGTTGGTGCTGTTGGCTTTCAGCATCGCCGTCTTGACATCCCAGACAATTTGGATCCTGCCATTGCTGACATTATTAGGAAATGCTGGCAAACTTAA

>Csa3M892210.1

ATGCCGCATAGAACGACTTACTTCTTCCCCAGGCAATTTCCGGATCGCGGATTTGACTCGGCGTCAACCTCCAAACACATTTTGGATCACGAGAAGAAAATCAACAAAGACACTTTTAGTACGGAAAGCGACGCGAAACCGACTCCGAGGCCAGCGCGCGACTTTAGCGTCACGAAGAGTTCGGCTGTATCGGATCTTTTCACGGGGGACAAGGCACAAACCAATAAGAAACTGCCTGCTTTTTACGATTGGTTGGTGGACAAGAAAGCAACGCGGTCGGCAACAGCTCACGTGAAAACTTGGCTTTCCAACTGCGATGAGGACCGCGAACTTTTGCTTCCGCCACCTACCTCGGAGCCCGAGCACGATACAACGTCGGTTAAGGATCGGAGTGTTGACCGGAACTTTGACCGGCAAGTTTCACTGCCTAGATTATCAAGCGGGAGTAGCTATGCGGGGAGTTTGTTTTCAGGCACGGGAACGGGGACAGTGGATGGAAACTTTTCCAGCGACGTCAAAGATTCATCGGCGTCGAAGATATTGTCCTCGCACACGGCCAGGCCGGAAGAGATTGAGGTTGGAGATGATAAGGAAAACATAGCACAGAAAGCGACAGAGAGTTATTACCTGCAACTTGCTTTAGCTGCAACGCTTCGTTCTCATGCTAATCTCGCCGGCGATCCTGTGCTTATGGAGGAAGGCAGGGTGGAAATTACGGACGCAGAAACAGTTTCTTATCGACTTTGGGTAAGTGGTTGTCTGTCTTACTCCGACAAAATATCAGATGGTTTCTACAATATTCTGGGTATGAACCCGTATCTCTGGGTTATGTGCAATGATTTTGAGGAAGGTAGACGGCTACCTTCTCTGATGTCGCTTAGAACCATTGAACCAAGTGAGACGTCAATGGAGGTGATTCTTGTTGATAGACGTGGGGACTCTCGACTGAAAGAGCTTGAAGACAAAGCACAGGAATTATATTGTGCTTCGGAGAGCACCTTAGTGTTGGTGGAGAAACTAGGAAAGCTTGTTGCGATCTACATGGGGGGCACTTTTCCGGTGGAGCAAGGGGGGTTACACCTCCATTGGAAAGTGGTGAGCAAAAGATTGAGGGAATTTCAGAAATGCATAGTTCTTCCAATTGGTAGTCTTTCTATGGGACTATGCAGGCATCGTGCAATCCTTTTCAAGAAATTGGCTGATTATATAGGTTTGCCATGTCGGATAGCTAGAGGTTGCAAGTACTGTGTTGCAGATCATCGATCCTCTTGCCTTGTCAAAATTGAAGATGACAAGAAATCGTTGAGGGAATATGTAGTTGATTTAGTGGGGGAACCGGGAAATATACATGGTCCAGATTCATCAATTAATGGAGGGTTCCAATCTTCAATGCCTTCACCGCTCCAGATTTCTCATTTAAAAGAGTTTCAAGAACCTTACGTGGAAAGCTATTTCAATCATCAAACTGTCGGGTCAAAGCAAATCTGTGGTTTTCCTGAATATCCTCTACGTTCAGGTTTTGGACAGTACCAGATGAAAGGGGGAAGCACTTTACGTATGAGTTCAGGGGCTGAAACTGATAAATTGGTTGATCAAGCTTGTATGGGTATAGGATCGACTCAGTTGTGTTTGGAGACAAAAGTAAGCAAGGAGTGTGTGCTTCAGAATCATATAATGCCATCCACTGGGGCTGATGCCTCCGAGGTGTTGAGTTCTGTTGGTGGTGCATCTTTATGTGAAAACAAAGTTGTCATTGAAGAAATATACCAAGAAGAGGCAGTTGTAGCAGCAGGGATTTCAGTGAATGAAACCATTAATCCGTCCAAATTAACCTTGTCAACTCAAACTGATTCGAAGGAGATTGTGGGTAGATCTCAAAATTGTTCAGCATCAACATATCCAAAATATTTGACACTTGAACCATCACTTGCAATGGACTGGCTGGAAATTTCATGGGATGAATTACATATAAAGGAGCGTGTCGGAGCTGGTTCATTTGGTACAGTGCATCGTGCTGAATGGCATGGATCGGTTGCAATAATGAAGCGGGTGCGCCATCCGAATGTGGTACTTTTTATGGGTGCAGTTACAAAGCGACCTCATCTTTCGATAGTGACTGAATACCTGCCTAGGGGTAGTCTATACCGCCTAATCCACAGGCCATCTTATGGTGAATTGATGGATCAAAGGAAACGATTGCGCATGGCATTGGATGTGGCAAAGGGAATCAACTACCTACATTGTCTCAATCCTCCTATAGTACACTGGGATCTTAAGTCTCCCAACTTGTTGGTTGACAAAAATTGGACAGTGAAGGTTTGTGATTTTGGGTTGTCCAGATTCAAAGCAAACACTTTCATATCATCAAAATCTGTTGCTGGAACGCCTGAGTGGATGGCTCCAGAATTCCTTCGTGGAGAGCCTTCAAATGAGAAGTCTGATGTCTACAGTTTTGGAGTGATCCTCTGGGAACTAGTCACCATGCAACAACCATGGAGTGGGCTTGGCCCGGCTCAGGTTGTGGGAGCTGTCGCTTTCCAGAACAGAAAGCTTTCTATCCCATCAAGTACTTCTCCGTTGTTGGCTTCCCTTATTGAATCTTGCTGGGCAGATGATCCTGTTCAGCGCCCATCCTTTGCAAGCATAGTCGAGTCCCTAAAAAAATTGCTGAAATCTCCACAGCAGTTGATAGCCATGGGTGGAACGTGA

>Csa6M330990.1

ATGAATCCCGCCGCCGCCGCCGCCGCAGACGGCGGCGGCGGCTGCTCTGGCCCCGCCGCTGTGAATTTCACCAACAAACAGTATCTAAATATCTTACAGTCACTGGGACAGCCTGTTTATATTTTTGACCTTAATTATCACATAATTTACTGGAACCGAGCTGCTGAAATTGTATATGGGTATTCTGCGGCGGAGGCTCTTGGTCAGGATGCCATTGAACTTCTAGTAGATCCTGAGGATTTCGCCATTACAAACCATGTCATTCTTCGTGTAATGGCGGGTGAGAATTGGACTGGTCATTTACCTGTAAAGAACAAGATGGGGCAAAAATTTGTAGTTGTTGCTACCAACACTCCATTCTACGACGATGGTGCATTGATTGGGATTATTTGTATATCCAGCGATTCCCGGCCATTTCAAGATTTGAAGATTCCTTTATCAATAGGTTCTAAACAGCAGGATGCAGATTCAAGCATGGTTCGATCACGAGTCCCGGTTTCGGTTAAGCTTGGTCTTGATCCTCAGCAGCCTCTTCAAGTGGCAATCGCTTCGAAGTTATCGAATTTGGCATCAAAAGTAAGCAACAAAGTCAAGTCCAAAATTCGAACTGGAGAAAACAGCTTGGATCGTGAAGGTGGAAGTGGAGATGGTTATCACTCTGATCATGGCCATCCGGATGCAGTTCTTTGTGATAATCGGGATGATGCAAATTCGAGTGGAGCTAGCACGCCACGAGGAGATTCCACGGCTCATGGTGCATTTTCCCAGGTTGAAGAGAAGTTATCTGGGAGACTGGTGAGGGATTCTAGTGATGAGGGTAAGGGAAAACCTACCATCCAGAAGATTTTATCCTCAAAGGCAGAAGAATGGATTGCCAAGAAAGGCTTATCGTGGCCGTGGAAAGGGACTGAGCAGGAAGGAGGATCTGAAACAAGAGCAGCCCGTTTCGTTTGGCCTTGGGTACAAATTGATCAAGAAGCTGAACCAGCTAATCATAAGAGTTCATCTATATCTGGTAAACTAGAAATGCAGCAAAATGACGGTCATCGAGCAGTCAATAATGAGGCATCGGGATCTTGGTCATCGATCAATATTAACAGCACTAGCAGTGTAAGTAGCTGTGGGAGTACCAGTAGTAGTGCTGTCAACAAGGTTGAGTCGGACATGGACTGCTTGGATCATGAAATCTTGTGGGAAGACCTTACCATTGGGGAGCAAATAGGACAAGGTTCTTGTGGAACTGTCTATCATGCACTGTGGTATGGATCAGATGTTGCCGTTAAAGTGTTCTCCAAACAAGAGTATTCAGATGATGTGATTCTCTCCTTCAAACAGGAGGTATCCCTGATGAAAAAGCTTAGACATCCCAATATTCTTCTCTTTATGGGAGTAGTGACTTCACCTCAGCGTCTCTGCATTGTCACAGAATTCCTTCCACGTGGAAGTTTGTTTCGTTTACTACAGAGGAACACAGGCAAATTAGATTGGAGACGCCGTGTTCATATGGCTTTGGACATTGCGCGAGGCATGAACTATCTTCACCATTGCAATCCACCCATTATTCACCGAGACTTGAAATCGTCGAACCTCTTAATCGATAAAAACTGGACCGTGAAGGTTGGAGATTTTGGTCTATCTCGACTTAAGCATGAGACTTATCTAACAACTAAGACCGGAAAGGGCACGCCTCAATGGATGGCGCCCGAAGTTCTTCGTAACGAGCCCTCAGATGAGAAGTCTGACATATACAGTTTTGGAGTCATATTGTGGGAGCTTGCAACTGAGAAGATCCCTTGGGAAAATCTCAATTCAATGCAGGTCATTGGTGCTGTTGGTTTCATGAACCAACGTCTTGAAATCCCAAAGGACGTGGATCCACAATGGATTTCAATTATTGAAAGCTGCTGGCACAGTGAACCTTCAAACCGGCCATCATTCCAAGTTTTGATAGAAAAGCTGAGAGACTTACAAAGGAAGTATACTATTCAATTACAAGCAGCTCGTTCGGGTGGAGATAACAGCAACAGCAACAACAACAATATCCCTCAAAAGGAAACATAA

>Csa6M154510.1

ATGGAGGATCAGCGAGATGATGTTGCACCATCAGAGCAAGCCCCATCCAATGCTTCTTCTTGGTGGTCTTCTGATTTTGAGGACAAATTTGGATCTGTTTCTTTGGGCCCTCGGGAAGATATTGTAAATGAAAAGGAAGAGATCATCAATTCTGACCAAGATGTGTTGTTCTCGCCTCAGACAGCATCTCAAATTCTCTGGCGTACTGGAATGCTTTGTGAACCTATTCCAGATGGGTTCTATTCTGTTATTCTGGATAAAAGGCTAAAAGACCGGTTTCACAGCATTCCTTCCCTGGACGAGCTTCGTGCTTTGGAGGTGGAAGGTTACAGGAATGATGTTATTCTTGTGGAGACAGAGAAAGATAAAAAGCTTTCTATGCTGAAGCAACTGATCTTGACACTGGTTAAAGGATTGAATTCAAATCCAGCTGCAATTATCAAGAAGATCGCTGGACTGGTTTCTGATTTTTATAAACGACCAATCTTAGAAAGTCCAGCAAAAGGTGCTCTTGAAGAAACCTCACACTTGTTCGAGGATAGAGGCATTCAATTGCTTGGACAAATAAAATTTGGTTCTTGTCGTCCTAGAGCTATCTTATTTAAGGCTCTGGCAGACACCGTAGGGCTTGAAAGCCGGCTCATGGTGGGCTTGCCAAATGAGGGGGCTACTGGGTGTGTAGATTCATACAAGCATATGTCTGTGACAGTTGTATTGAATTCTGTGGAACTAGTTGTTGATCTGATGCGGTTTCCTGGCCAGTTGTTACCTCGGTCAACTAAGGCCATTTTTATGACACATATTTCCGCTGCTGGTGAAAGCGATTCTGCTGAAAACGACTCCTGTGATTCACCACTAGAACCTAATAGTCCTCTTTATGGTTTCTCAGAAAGAGTTGATCCTGACAGTGTCGAGAAAGATGAGAGCCTCCAATTCCACAGAAAATTTGATGCAACTTCAAATGCACATGGTAATTCATTGCGCAACATGATGTTGCGATCAAGTACAGCACTTGACAGAAAACTGAGTTTATCACATAGTGAACCTAACATTGCAAATGCATTTTGGCGACGTAGTCGGAGAAAGGATATTGCTGAACAGCGGACTGCTAGTTCAAGTCCAGAGCATCCTTCATTTCGGGCACGTGGCCGGTCTATGCTTAGTGGGGATAGGAAAGCCTTCAGAGATTTTTCTGACGATGTCTCTACTTCAAGATCAGATGGTGCTTCAACTTCAACTTCAGAAGCACGTCGATTAAGAAGAAGGAGCATCAGCATTACTCCAGAGATTGGTGATGATATCGTGAGGGCTGTACGAGCAATGAATGAAACACTGAAGCAAAATCGTCTTTTGAGAGGACAAGAAGATGACAGGTCGTTCTCCCATCCTTCAAATGAAAGAAATAGTAGTTCAGATGTTCGGAGAAATGATCAAGTGGGTTCTCAAAGAGCAATATCATTACCATCATCGCCTCACGTGTACAGGGGTCAAACCTCTGATGGAATTGGGCATTCAGCATACGGGAATGATGAATTAACCTTTAAATGGACTAAAGTTCTTGAATCTTTCTCCTTGAATGACAAACCACTATTACCTTACCCAGAGTGGAACATCGATTACTCAGAACTGACAGTTGGTATCCGTATTGGAATTGGGTTTTTTGGAGAAGTTTTCCGTGGAATTTGGAATGGAACAGATGTCGCAATCAAAGTTTTCCTAGAACAAGATTTAACTCCTGAGAACATTGAGGACTTTTGTAATGAGATATCAATTCTTAGCCGTCTTCGACATCCTAATGTTATACTATTTCTGGGGGCATGCACAAAGCCGCCACGCTTATCAATGATCACCGAATACATGGAAATGGGTTCCTTGTATTCCTTGATTCATTTGAGTGGTCAGAAAAAAAAACTTAGCTGGCGGAGGAGATTGAAGATGTTGCGTGACATATGCAGGGGTTTGATGTGCATACACCGAATGAAAATAGCTCATCGAGACCTAAAAAGTGCAAATTGCCTAGTGAACAAGCATTGGACCGTTAAGATATGCGATTTTGGGCTTTCAAGAATATTGACAGATGCTCCAGCTAGAGGGTCTCCATCTGCAGGAACTCCAGAATGGATGGCTCCTGAGCTCTTTCGAAACGAGCCCTTCACTGAAAAGTGTGATATCTTCAGTCTGGGGGTCATTATGTGGGAGCTATGCACGCTAAACAGACCATGGGAGGGTGTCCCACCGGAGCGGGTAGTTTATGCTGTTGGCACTGAGGGGTCCCGCCTCGAGATTCCCGAAGGGCCACTTGGCAGGCTTATATCAGATTGTTGGGCAGAACCAAATGAACGGCCAAGCTGCGAAGAGATCCTCTCACGCTTGCTGGACTGCGAGTACTCACTTTCTTGA

>Csa3M133150.1

ATGGCTAGGGAAGTACATGGCGTTTTAAGCCAACAATTATACATGGAAAGACCAAGTGTTGTTTCGGATGTACGGATTACTGCGGATCATAGTGTGAGTGATGTTTGTGTACAGACGGGTGAGGTATTTTCTCCACAGTTTATGCGAGATCGTGTTGCGTTAAGAAGATTTTCTGACATGAGTGATGGAGATCAACAACAACAGCAGCAAAAGAGAAAAGGTTTTGGGTTCAATCCGAGCAACCAATTGGTGTATGAGGATCTTAGTGGAATTCTTGGACTCAAGAGGATGAATTCTGAAAGTAGTTCAGAAATGTCATCAACGCCAATGACTGCTTATGCAGCTGAAAAGGACAACAAGGTTTATCCTAATACCACAAGCAAATGTCAGTGGGAATATAACAATGGTACTGGACAAGCATCTGCCGCATATGCCGACGAAACTAATCGAGGAGTTCAAATTGGCCCCATGATGTCGGCCTTGTATCCATTGGATTCGCCTCATTCATGTTATCCTTGTGGGGCTGGATTTGGAGATTTCTCTGCCAATGACAAGATGAAATTTCTCTGTAGTTTTGGAGGTAGAATATTACCGAGGCCTAATGATGGGAAGCTTAGATATGTAGCGGGAGAAACGCGCATCATATCCATTAGGAAAAACATCTCATATGAAGAACTTACCAAAAAGACATACGCTGTCTGTAAATATGCCCACACAATAAAGTACCAGCTTCCTGGTGAAGATCTTGATTCACTTATCTCTGTCTGCTCTGATGAGGATCTTCATCATATGATAGAGGAATATCAGGAGCTGGAAAACGCAGAAGGTTCTCAGAGACTTAGAATTTTTCTTATTTCTTCGAATGATTGTAGTGAGAGTCCTACTTCTATTGAAGGAAGGGTAGTTCCACCAATTGATGTGGATTATCAATATGTTGCGGCTGTGAACGGTATTCTAGACCCGAGTCTTCAAAGGAGCTCCAGTGGGCAGAGTTTCACAAGCCAGAATAGCCAGGTGGGAGCCATCTCAGATCATAGTCCCAATTTTCGTACTGATTCGTCACATGCTACAGACGTGAAAGATGTCAGTTCACTAATGCCAAATTTGATGGGAATGTTACCTAGACCAGGTGGTCAATTGTTAAACCCCATCCAAGTCCCACGCAAGTCATTAAATCAATCACCTTTAATTTCTCCGGTTACTGTAATGCAAAAAGATTTTAGGAACGTGGATGCAACATATGCAGAAGACGCTAGAAACTTCTCTCCAATTGTTTCAGGAAAACATCCATGTGATTCAGTTTATTATGTTGATGCAATGGGCCGTCACAATTATCTTTACCATGGTTCTCCGTTGATGAATTATTATCATGAAAAATCTACTGCGGAAACTGATGAGACATACAAAGTTCTTAATGTTCATTTCCCACGAAGCTCCAGTGAGGATTTTGTTCCTGCACCCATCTGGGGTCTAAGTGATACACATCCAATGAAAACTATGCTGAAGGAACGGGCAGTTAATTATGAACAACTATGCTCTGACGCGGAGTATTTGATGCAGCTAAGATCTGGAACCACCCATATGGGACAAAGAATTATCCATTCGCATTCTGAGCCCCTACTGCTGGAGCAGGATCAAAAGCCGAATCATGGAGGGCCCTATCCACTAACCTCATTCAATGATAGTGATCAGTCACCTTCACTTGCAATGTCAAGTTCCTTGCAGGATTTGCCAACGTTGTGGAAACAGAGAGATGGCGTGGAATTTCAATATGCTAAATATGAGAATCATCGAAAGTTGGCATCTGGTAGTGATAATGAAACATATGAAGAATGCAATTTTGATGGAAAGAAGAATAATTTCAATGGAATTATTTATGCTCCATCACTTAATGATGAAGAAAAATACAGATATTTGCAACATGCTGGCTACCGACAAAATGGTTGCCCCCCCAAAGAGGTTCAAAACCTTAGAGGTAGATCTTCTGCTGAACGAGGTATTGAATTAGAAAATTCTGCAGATACCACGGGTGCCCCTTCCCTCGTTTACCATTTTGAAAGAACTGCTCCAAAAGATTTCGAGGAGAGCCAATATTCTACCAAGGATCAACCAACTACTTCTGATATAGTAAGGAGCCAGCCACTATCGTGCACTTCTTCCGATCTACTACCTCATACAATTCAAGCTTTCGACGATGTAAAGATCATTAATCAGAAACCAACATGGGATAGTTCTGCTTCAGGTATAGAGATTTCTCTAGGCGATGAGAACTTTGTAACCTGTCATTACTGCAAGGTTGCAGCCCATAGCAGAAGAAAGAGCAACTGTGATGATGCTATATCACACTCAGATGATTCTCATGGCAATGAGGATGAGGATTTAGCTGTAATAGTTGAAGATGTAACTCATAGTCTGCCTCCTGACATACCCTTGGCTTCAGGAATTGTCCCGCGGGTTGAAAATGAGGCCAGCGATGAATTTCCATCTTCAAGAGGAAATGATGCTCTGAGTTCTAGTTCAGAAACTGACCATGAGGATGCCGACAGTATTCTTAGTTCAAGGGACGAGTCTATGAGTGAGGCAGCAATAGCTGAAATCGAAGCTGGCATCTATGGCCTGCAGATAATAAAGGATGCAGATCTTGAAGAATTGCAAGAGTTGGGATCTGGGACATTTGGTACCGTTTTTCATGGAAAATGGAGGGGAACAGATGTTGCTATTAAGAGAATAAAAAAGAGTTGCTTCTCAGGCAGTTTTTCAGAGCAAGAACGACTGACTAGAGATTTCTGGAGGGAAGCAAGGATTCTATCGACCCTTCACCATCCAAACGTTTTGGCTTTTTATGGGGTGGTTCCTGATGGACCTGATGGAACATTGGCAACTGTAACCGAGTACATGGTAAACGGTTCGTTAAGGCATGTCCTTCTAAGGAAAGATAAAGTGCTTGATCGTCGGAAAAGGCTTATAATTGCAATGGATGCGGCTTTTGGAATGGAATATTTGCATTTGAAAAATATTGTTCATTTTGATCTGAAATGTGATAATTTGTTGGTCAACTTGAGGGATCCTGAACGACCTATATGCAAGGTTGGAGATTTTGGGTTGTCAAGAATTAAACGCAATACACTTGTTTCTGGAGGTGTGCGTGGAACTCTTCCATGGATGGCACCAGAATTGTTAGACAGTACCAGTTCTAAGGTCTCTGAGAAGGTTGATGTTTTCTCATTCGGTATTGCAATGTGGGAGATTTTGACAGGCGAGGAGCCATATGCTAACATGCATTGTGGTGCCATCATCGGTGGAATTGTAAGTAATACTCTTAGACCGCCAATTCCGAAACGCTGCGATCCCGAATGGAAAAAGCTAATGGAAGAGTGTTGGTCTCCTGAACCTGCAGCAAGACCATCATTCACAGAGATAACAAATAGACTTCGCAGCATGTCGGTAGCGCTTCAGATCAGAAAGAGGCCAAATGTAGCAAGTAGATGA

>Csa6M136540.1

ATGGAACAGTCAAGAAATGACCAGTTCCAATGCACTCCCATGCATTATGGATTTGGGGAACTCCAACCTGCGTCTAAATCATCCATAGAGAACCGTGTGAGCATTGTTAACATGCAAAGTGATGGAAGAACCATGGACCTTAGGATGTCAGAGGTTAAGCCCGTCCTTAATTACTCCATACAGACAGGTGAGGAATTCTCTTTTGAATTTATGCGCGACCGAGCAAATCCCCGGAAACCACTCGTTTCGGACTCTGTTAGTGACCCCAGTTGTGCATCACGTTATATGGATTTAAAAGGAATTTTAGGCCTTAGTCGTACTGGGTCTGAGTGTGGCTCAGATAATTCTATGATCATTTCAATGGAAAAAGGGTCAAAAGATTTTGAGCGGACAAACTCTTCATTACATGGAGGAGACAGAAATAACCTTGGATCAGCTCATCAAAAATCACCGGAGCTATCAAGATATGATAGTGGTCGTGCAATTGGTCATGGCTATGCATCTTCTGGAACTTCTGATGGCTCATCAGCAAAGATGAAAGTCCTCTGCAGTTTTGGTGGGAAAATTTTACCTCGTCCAAGTGATAGTAAGTTGAGATATGTTGGGGGTGAAACTCGTATCATTCAGATAAAAATGGACATCTCTTGGCAGGAGCTTATGCGGAAAACATCATCAATCTATAATGAAACATATGCAATTAAATATCAGCTTCCCGGCGAGGAGCTTGATGCTTTGGTTTCAGTTTCTTGTGATGAGGATCTTCAAAATATGATGGAGGAGTGCAATGAATTCAAAAATGATAAAGGATCCAAAAAACTTAGAATTTTTTTGTTTTCAATGAGTGATTTGGATGAAGGGCATTTTTCCATGGGTAATGTTGATAATGACTCTGAGATCCAGTATGTTGTTGCAGTCAATGGCATGGACAGGAAAAACTCAAACCTGCATGGTTTATCAAGTTTTTCTGCCAATAACCTGGATGAGGTAGATGGACAGAGTATTGAGAGGGGTACAGTTCTTAAGGACTTGGTCGGTGTAAATGCATCGGCCTTAACTGCTAATGTTGCATCGTCATCCTTACAGTCTTCACAACCTGTTCGGGCAAGTGCATCGAATGCTTATGAAACCTTTTTACAGGCTTATCATGAACCTCAAGGACAAAATTCAGAGATACCATCAACACAGTTGAAAGGTAAGTTCAAGGATTCATTTGAGAAAGAAACTCATGATGCAAGTGGCTGCTCTTCAAATCCTTCTCACTTTTTTGATGGCAATCTCATGACTTCGGATAAAAAATCAACACCAGTTTCCATCGCGCAAGGTGAGTTTCCCTTTTTAACTCACAAGAATGAGACCGAGCTCCAGAGTTCTGAAGGGCTTTCTTCGATGCTTGCTTCTGGAAATCCTATAGTTTCTAGATCAAATGATATGGATAACATTATTCATAACATGCCATCAAATGCATATCCACATGGTCATACAGACAGCGAGTCAAAGATTGTTGATTTGAGTCTCCTTGAGCCTCCAGCTGTTGCGCAAAGAGTCTATTACTCTGAGAGAATTCCTAGGGAGCAGGAGGAGTTACTGAATCGGTTGTCTAAATCTGATGATTCGTATGGTTCCCAGTTTCTAATCTCTCATTCACAGTCTGATCAGGACCAAATTCCAGACTCTGCTGTCAAATTGCAAGATAGCAGTAATTACGAATCTGAAAATTCCATTCCCATGGAAAAATCATCACATAATGCTACTAAAGTCCGGAATGATGAGCTTTCCCATATTCAAGATGGCAGAAATGTTAATGAGGCAGTTAGTGGAAGGAATTGGAATATTTCACATGATGGTGATACCGAGTTGAAGTTGCAGAACAACTTTGATGTTACACTTGATTCAAAAGTAGATGGGGTTGTTAAGGCTGGCAAGGACCTGAATTGCCCAGTGAATAACAATGAGAAGCTTGCAGGTCCTAAATTAAGTAGACCTGAATCAGAATTACCTGCTCTTGGCCAAGTATCTTCTTTAAAGAATCATGAGGATTCTGCGTTGGATCTTTTGCAGCTCAACTTGGGCGAGGTTGTTGGAATGAGGTGTACCGATGATAATAGTCTTAAGCAAACTCAGTTGGCTTATAAGGAAGAAAGTTTAATTAATCATGTTAATGAAAGACCATCCACGGGAAATGTTTCCAAGCCAGTGCAAGGAGACATTGTTATAGATATTGATGATCGATTTTCTCGTGATTTCTTATCTGATATATTCTCCAAAGCAATACCTTTCGAGAATTCATTAGATAGTCGGAGCCAACTGCACAACGATGGAACTGGTTTGAGTCCTGATGTGGACAATCATGAACATAAGGGCTGGTCATATGTCCATGATTTGGCACAAGAAAAGTATGTTCAAAATGATGTTTCTCTTATTGACCAAGATCACATTGTTTTTCCTTCTGCTCCTAAGACTGCGGGAGATGATTTTACACCTTTAACAACTATACTTCGTGAAGATTCCCAGCTCAATTTTGGTGATGATCAGAAAGTACATAGGATAAGTGGAAATGATGCCACCAATTTTCTTTCACGTTGTGATCATTCCCGTATGAATGGCATTGATAGTTCGCAATTTGATGCTATGATGGAAAACCTAAAGACATTGGAGTATAGGCATGAGAATGTTAAGGTAGCATCTAAGGACAGTGGCCTACCTCCCAATGATCCTTCTTTGGGAAATTTTGATCCTAATTCATTACAGATCATAATGAACGATGATCTTGAAGAGTTGAAGGAATTGGGTTCTGGTACTTTCGGCACTGTCTATCATGGAAAGTGGAGAGGAACTGATGTCGCGATTAAGAGGATAAAAAAAACCTGCTTTATGGGTCGATCTTCAGAGCTAGAGAGATTGACTGTAGAGTTCTGGAGGGAAGCCGACATCCTTTCAAAACTTCACCATCCCAACGTGGTAGCATTTTATGGTGTTGTGCAAGATGGTCCTGGCGGAACATTAGCTACTGTGACAGAGTACATGGTTGATGGTTCCCTTCGACATGTTTTGCTTTCCAAGGATCGGCATCTTGATCGACGTAAGAGGCTAATAATTGCTATGGATGCAGCATTTGGAATGGAATATTTACACTCAAAAAATATTGTCCATTTTGATTTGAAATGTGATAATTTGCTTGTTAACTTGAAGGATTCTCAAAGACCAATTTGCAAGGTAGCTGATTTCGGCCTCTCAAAGATCAAGCGAAATACCTTGGTGTCTGGTGGTGTAAGAGGAACCCTCCCATGGATGGCACCTGAGCTACTAAATGGCAGCAGCAATAAGGTCTCTGAGAAGGTTGATGTGTTCTCCTTCGGTATTGTCTTATGGGAGATTCTCACAGGAGAGGAACCATATGCGAACATGCACTATGGGGCTATCATAGGGGGTATTGTGAATAACACACTGAGACCAACCATACCAAGTTACTGTGATTCTGAATGGAGAAGGTTGATGGAACATTGCTGGGCACCAAATCCTACAGATAGGCCATCCTTCACCGAAGTCGCTGGCAGGCTGCGCGTTATGTCAACTAGTGCTAGTAGCCAAACAAAAGCACAAGGTCCCAAAATAGCAAGGTCATAA

>Csa6M511830.1

ATGGCGACCTCATGTTTCGGTTCACTTAGGATTCGTAAATCAAAAGGCAAAACCTTATCAACCCCTTCCTCTTTGAAATCCCAGATGAATTCTGAAATGGAAAATATGGAAAGGAGAAGATTTGATAGTTTGGAATCGTGGTCGATGATCCTAGAGTCTGAAAATGTTGAAACTTGGGAGACATCAAAGGAAGATCAGGAAGAGTGGACAGCTGATTTATCTCAACTCTTCATTGGTAACAAATTTGCCTCCGGCGCTCACAGTCGGATATACCGTGGAATTTACAAGCAGAGAGCCGTTGCTGTGAAGATGGTGAGAATTCCAAACCAAAAGGAGGAAACAAGAGCCAAACTCGAGCAACAGTTCAAGTCCGAAGTTGCCTTGCTTTCTCGTCTCTTTCATCCCAACATAGTTCAGTTCATTGCAGCCTGTAAAAAGCCACCTGTATACTGCATAATTACAGAGTATATGTCACAAGGAACTCTGAGGATGTATCTAAACAAGAAAGAGCCGTATTCACTCTCAACAGAAACAATACTGAGGTTAGCTCTTGACATATCAAGAGGAATGGAATACCTCCATTCCCAAGGAGTAATCCACAGAGATCTCAAATCAAACAACTTGCTTCTAAACGATGAAATGAGAGTAAAAGTAGCCGATTTCGGAACCTCCTGCCTCGAAACACAGTGCCGAGAATCAAAAGGGAACATGGGAACTTACCGATGGATGGCACCGGAGATGATCAAAGAGAAGCCTTATACTCGCAAAGTTGATGTGTACAGCTTTGGGATTGTGCTGTGGGAACTCACCACGGCTCTGCTTCCCTTTCAAGGAATGACCCCCGTGCAAGCTGCCTTTGCCGTCGCCGAGAAGAATGAGAGACCACCATTGCCGGCGAGTTGTCAGCCGGCGCTAGCCCACCTGATTAAACGGTGTTGGGCGGCGAATCCGTCGAAGCGGCCGGATTTCAGCGACATTGTGGCGGCGTTAGAGAAATACGACGAGTGTGTGAAAGAGGGACTTCCTCTTGCACACCATAGAAGATTGGTAAACAAAAACGCAATTATTGAACGCTTGAAAGCTTGTGCTTGA

>Csa1M046040.1

ATGAGGAATTTAAACTGGTTTAAGCCAATTTCGATTAATGGGAAGCCTGGGAGGAGGCTTTCACTTGGAGAGTACCAACGGGCTGTGTCGTGGTCTAAGTATTTGGTGTCTTCAGGAGCTGAGATAAAGGGAGAAGGAGAAGAGGAATGGAGTGCTGACATGTCCCAGTTGTTCATTGGCTTCAAATTTGCTACTGGAAGGCATAGTAGGATTTACAGAGGTGTCTATAAGCAAAGGGATGTTGCAATTAAGCTGATAAGCCAGCCTGAGGAGGATGAAAACTTGGCTAATTTTCTTGAGAATCAGTTCATTTCAGAGGTGGCATTGCTGTTTCGATTGAGACATCCCAATATCATCACTTTCATTGCAGCTTGCAAGAAACCTCCAGTGTTTTGTATTATCACGGAGTATATGACAGGTGGTTCGTTAAGAAAATATCTCCATCAACAAGAGCCACATTCCGTTCCGCTGAACTTGGTTTTGAAACTAGCTCTCGACATCTCACGTGGGATGCAGTACCTTCATTCTCAGGGAATACTTCACAGAGATCTTAAATCAGAAAATCTCTTACTTGGTGAAGATATGTGTGTTAAGGTAGCAGATTTTGGTATCTCGTGCTTAGAATCTCAATGCGGAAGTGCAAAGGGATTCACCGGAACTTACCGCTGGATGGCACCTGAAATGATCAAAGAAAAACACCACACTAAGAAAGTTGATGTCTATAGCTTTGGCATTGTCTTGTGGGAGCTCTTAACTGCATTGACACCATTTGATAACTTGACTCCCGAACAGGCAGCATTTGCAGTCTGCCAGAAGAATGCAAGACCACCTCTGCCTTCTGCGTGCCCGCAGGCATTTCGTCATCTGATTAAGAGATGCTGGTCGAAAAAACCCGACAAGCGACCGCATTTTGACGAGATCGTTTCAATTTTGGAAACTTATGTGGAGTCTTACAATGAAGATCCAGAATTTTTTTGTCATTATGTCCCCTCATCTAGCAGATACATTGCTTGGAAATGCTTACCAAAATGTATTACCAAACAATCATCTGCTTCCTTGAAGCCTAGGAATTCTTCATCCTCTTGA

>Csa2M070870.1

ATGTTGGAGGGTGGTCAAAAATTCCCTGGAATGATCGATTTAAACGAGCATGCATATGATCTATCGCAAGGGTTTTACCATAAACTTGGTGAGGGAACCAACATGTCTATTGACAGCTTTGCAAGCTTGCAAACGAGCAACGATGGAGGGTCAGTTGCCATGTCTTTAGATAACAGCAGTGTAGGATCGAACGAGTCTCACACTCGGATCTTAAATCACCAAGGCTTGCGACGGCGTGCAAATGACAACCATACTTTTCAACATAGTGTTAACCGTCGTGGAAGAGTTACACATCACCTGAGTGATGATGCGCTAGCCCGAGCTTTATTTGATAGCAATACACCCACTCAGGGTCTTGAAAATTTTGAGAAGTGGACTCTTGATTTAAGAAAGCTTAATATGGGAGAGGCTTTTGCTCAAGGTGCCTTTGGGAAACTCTACAGAGGTACTTATGATGGTGAAGATGTTGCTATCAAGATCTTGGAGAGGCCAGAGAATGACCTGGAAAAGGCTCAGTTAATGGAGCAGCAATATCAACAGGAGGTGATGATGCTTGCTACTCTAAAGCACCCAAATATTGTCCGGTTTATTGGATCATGCCATAAGCCGATGGTTTGGTGCATTGTTACTGAGTATGCGAAGGGCGGTTCAGTTAGGCAGTTCTTGATGAGGCGACAAAGTCGTTCTGTCCCGCTGAAATTAGCTGTCAAACAAGCTTTGGATGTTGCCAGGGGGATGGAATATGTTCATGGCCTTGGGCTGATACACAGGGACTTGAAATCAGACAACCTTTTGATTTTTGCTGACAAGTCTATCAAAGTTGCTGACTTTGGAGTGGCCCGGATTGAGGTGCAGACAGAAGGAATGACACCTGAGACTGGAACCTATCGCTGGATGGCTCCGGAGATGATCCAACACAGGCCTTACACACAAAAGGTGGACCTGTATAGCTTTGGTATCGTTCTGTGGGAGCTCATAACTGGAATGCTTCCGTTCCAGAACATGACAGCTGTTCAGGCTGCCTTTGCAGTTGTAAACAAGGGAGTCCGTCCTATCATTCCCAATGATTGCTTGCCTGTTTTGAGCGACATCATGACCCGGTGCTGGGATCCCAATCCTGATGTTAGGCCATCATTCACCGAAGTCGTCAGAATGCTTGAGAATGCACAAACTGAGATCATGACGACGGTTCGTAAGGCTCGTTTCAGGTGTTGCATTACGCAGCCGATGACAACAGACTGA

>Csa1M057040.1

ATGGAAAGAAATGTGAAAAAAAGTACCCTAGATCAACCAAGTAATTATGAACAAATTCGACTTACTAGTATGGAAGGAAGAAATCAGGGACTTGGGTCTACAAATCAAAGAACTTTCCATGATCCATCGAGTAATATCAGCACTAACATTCGCCCCCCTGAATATAATATGCTAGTTGTAGGTGTAGCTAGTCCTGGCCATAACTACTCCATTCAAACTGGCGAGGAATTCGCACTTGAATTTATGCGAGAAAGGGTGAATGCCAAGCATCATTTTGTTCCCACCAATAGTCCTGACCCAGGTGTTTCAACTGGGTACATGGATTTAAAAGGCATGCTGGGAATTCCCCATGCAAGTTCAGAAAGTGGATCTAGCATTGCCATGCTCAACCCTGTAGAAAAAGATCACGTCCAACATTTTGAAAGAGGGAGTTTGCCACATGAAGAAAAAAGTTCATATAATTCAATGAGGTTTGTCCCAAGAGCCTCATCGAGAAATGATGTCAGTCGGCTTCACAGTTTTACATCTTCAGGAGCATCTGATAGCACATCAAGAAAGGTGAAATTCCTGTGCAGTTTTGGTGGTAAAGTCATGCCGCGGCCTAGTGATGGTAAACTTCGGTATGTTGGGGGTGAAACACGTATTATCCGTATAACCAAGGATATTTCTTGGTCAAACCTGCTGCAGAAAACATCAACAATTTACGATCAAGTTCACACTATTAAATATCAGCTACCTGGTGAGGATCTTGATGCCTTGGTTTCTGTATCTTGTGATGAGGATTTGCAGAACATGATGGAGGAATGTAATATACCAGAAAATGGGGGTTCAACAAAACCAAGAATGTTCTTGTTTTCTATTTCTGATTTGGAGGATTCTCAAATGGGTGTTGGGAGTGCAGAGGGTGGTTCAGAGATTGAATATGTGATTGCTGTAAATGGTATGGACCTCAGTTCAAGAAGGAACTCAACGCCCTTGGGTAATACTTCAGGAAACAATTTGGATGAGTTACTTGCCCTTAATGTTGGATTAGAGAGTGGTCAAGTTGCGCCATTGTCTGATAACATGAAATCATCTTTGACCATTACTCCTTCTTTTCCTCAGTCTTCTCAAACAATTTGGACAAATTCATCCAGTGGTCTTAAGTCCAGTTTGCAGCCATTGTCAGGACAAAAGTTGCAGCAAGGTGAGCTTGGGCCTCCCCAACCCTCTTCTTTCCGCCCCATGCAAAGCTTTCCTGAAAAACTTGGAAAGACGTCAGTCTCTTCATCTATTCAGTCACAACATGATTACGTTCTTAATACCAATGCAACATCTGTAGAAAATGTACCTCCAATGCCCAGCAAAGGGTATTTGAATCAACACTATCCAGTCAGTGGTTTTCACACACAAGATCCAGATTCATCGTCAAGGGAGGGGAAAATTACAGAGATCTCAACTTCAAAGCTGAGTGAACCTGATGAAATTCAATCGTTGGAGAAGGAGGTTTCATTTAATGATGCACAGATGAAAAGGGAAAGCTCACTCCATAAGATTGATGAAGCTAATGAAAGTCCAAATTTTGAACATGAATGTGGAGTTTCATCCAACCTAAATGATGCTTCTGTTTTGAACTATAACACAAAAGGAATGCAAGTAATTAATTCAGACACAGATGTAGGATCAAGTTTACTGCTTACGAAAAACAACAAGCATCAAGATCCTGCTCCAGAATCTGTGTCCTTGGAAGCAAGTAATGAAGGAAACAGGGGAACTAAAGAAGATAAATTTTCATCTGACGAACTTCCAACTTCTGGGTTTGGTGCCTCCAAGGCTGATGAAACAGGGTTTAGCTACCTTGAGCCAATTTTACCTCAGCGTGTTTTTCATTCTGAAAGGATTCCAAGGGAGCAGGCTGAATTGAACCGTTTATCAAAGTCCGATGATTCTTTTGGCTCTCAGTTTCTGCGAACTCAGGGAAACTCAGATTATTCTCAGACAATTATTGAATCAGCTGAAACATTGCTGGATGGGAATATGACTTTGGAGTCTGAGCAATTTGTTTCATCATCAAAGTTACCATGTGGAAATCATCAAACTATAGAAGATGGATTGGAACCATTTGAAAAGTACAAAACATCAGCAGATAAGAATAGTAAAACTATGAATATTTCGGGTGAGCATGATGGGTCCGAAGTCAGTGATATGTCCAATATTAAAAGTCCATCTGCCTGTAGGAAGGAAGCAGAAGGTTTGGCTCATCTTACAGCAGGTGAAGAAGTTCCAGACAAGCATAAGGAGGAATCTTTGATGGGACCACTAGAATCAGGCTGGATTGAAGGAAGTACACATAATAACCATGGAAATGAAACTCAGGAGCAACCAGAGCCTTCATCATTGACAGAGAACCCTGGTAAGAATGCTACTCAAGTTGAGCCTGGAGTTGGTATTGGCACTTCAGAGCACGGGGACATTCTTATTGACATTAATGACCGGTTTCCACGCGATTTCCTTTCCGATATATTCTCCAAGGCCAGAAACTCTGAAAACATTTCTGGTATCAATCCATTGCATGGTAATGGAGCTGGCTTGAGCGTGAATGTGGAGAATCACGAGCCTAAGCGTTGGTCATACTTTAGAAACTTGGCTCAGGAGGAGTTTGTTGGAAGAGATGTTTCCCTTATGGACCAAGATCACCTGGGCTTCTCTTCCTCACTTGGAAATGTTGAAGAAGGGGGTACCGTGAACAGGTTTCCCCTTTTAAATTCAGATGTAGGTGCCATTTATGAAAAAGAATCTCATAATTTTGATGACAACATTCAGCCGGAATCACGTCTCCTCACTGGTCCCAGCACCACAAATCTGTACACAGAATATAATTCTTCACAGCTCAAGGGCAATGAAACCATGCATGAACCTAGCTCTAAGTCCCCACAAGATGAGAATGTGGATGCAAAGTTGGATGGCCAAGATATTGGTGTACCTCTTGTTGATTTTTATCTTAAAGATTTTGATATTAGTACATTGCAGATTATTAAGAATGAAGATCTTGAGGAGCAACGAGAATTGGGTTCTGGCACATTTGGTACAGTGTATCACGGGAAGTGGCGAGGAACAGATGTAGCTATTAAGCGGATAAAGAAAAGTTGTTTCACTTGTAGATCCTCAGAGCAAGAAAGATTGACGATAGAGTTTTGGCGTGAAGCTGAGATTCTTTCAAAGCTTCACCATCCAAATGTGGTGGCCTTCTATGGTGTGGTACAGGATGGACCGGGTGGGACATTGGCTACAGTGACTGAATTCATGGTCAACGGCTCCTTGCGGAATGTTTTACTCAGCAAAGAACGGTACCTGGATCGACGGAAGAGGCTCATTATTGCAATGGATGCAGCATTTGGAATGGAATATCTGCATTCAAAGAATATTGTGCACTTTGATTTGAAATGTGACAACTTGCTTGTCAACTTGAAAGATCCTTTTCGGCCAATTTGTAAGGTTGGAGATTTTGGCTTGTCAAAAATAAAAAGGAATACCTTAGTTACTGGTGGTGTGAGAGGGACCCTTCCTTGGATGGCTCCTGAACTACTTAATGGTAGCAGCAGCAAGGTTTCCGAAAAGGTTGATGTATTCTCCTTTGGTATTGTCCTGTGGGAGATCCTTACTGGAGAGGAACCTTATGCAAATATGCACTACGGAGCAATCATAGGAGGCATAGTAAACAACACATTGAGACCACCCGTCCCAAGCTTCTGCGACCCTGACTGGAGATTGCTGATGGAACAATGCTGGTCTCCTGACCCAGTAGCCCGACCATCTTTCACAGATATAGCAAGGCGTTTGCGTGTGATGTCTACAGCTGCCCAGACAAGATCACCACAGAATCAGATGCCCAAGTAA

>Csa7M051390.1

ATGGACAACATTACTGCTCAATTAAAGCGAGGTATCTCCCGCCAATTCTCCACCGGTTCTTTACGCCGTACCTTAAGTCGTCAGTTCACCCGTCAATCCTCTCTTGATCCTAGAAGGAACAATTTGAGATTTAGCTTTGGGAGGCAATCTTCTTTGGACCCTATTCGCCGCTGTCCCGATGAAGATAATGAGCTTTCTATTCCTGACAATTTGGATTCCACCATGCAGCTTCTCTTTATGGCTTGCCGTGGCGATGTTAGAGGTGTTGAGGATTTGCTTAATGATGGTACTGATGTTAATAGTATTGATTTGGATGGCCGTACTGCTTTGCATATTGCTGCCTGTGAAGGTCATGCTGCTGTTGTTAAGCTCTTGCTTAGTCGTAAGGCTAATATTGATGCTCGTGATCGTTGGGGGAGCACGGCAGCTGCAGATGCTAAATATTATGGAAATACAGAGATTTACAATATTCTCAAGGCACGAGGAGCAAAAGTTCCGAAATTTAGGAAAACGCCAATGACTGTTGCAAATCCTAGGGAAGTTCCAGAGTATGAGCTTAATCCATTGGAGCTTCAGATTCGAAGGAGTGATGGTATATCAAAGGGGGCATATCAAGTTGCCAAATGGAATGGTACAAAAGTCTCTGTAAAGATTCTTGATAAAGATTGTTATTGTAACCCAGATTCTATAAATGCATTCAAACATGAGTTGACATTATTGGAAAAGGTCCGACATCCAAATGTAGTGCAGTTTGTTGGTGCTGTTACCCAGAATTTGCCAATGATGATTGTTTCTGAATATCATCCGAAGGGTGATTTAGGGTGCTATCTTCAAAAGAAAGGTCGTTTATCTCCATCTAAAGCCTTAAGATTTGCCCTCGATGTGGCTAGGGGAATGAACTATCTTCACGAATGCAAACCAGATCCAATTATCCACTGTGATTTAAAGCCAAAAAATATTTTGCTGGATAATGGAGGACAACTCAAAGTTGCTGGATTTGGCTTGATAAGATTGTCTAAAATGTCACAGGACAAAGCAAAATTAGCTCACCCAGTTGTTATTGACTATTCAAACTTGTACTTAGCACCCGAAATTTACAACAATGAAATATTTGACAGAAGCGTCGATTCATTTTCATTCGGTCTCATTTTGTACGAGATGGTTGAAGGTATTCAACCATTCCATCCCAAGCCTCCAGAAGAGGTTACCAGAGCTATTTGTGCAGAAGGAAAGAGACCTCCATTTAAGATCAAATCAAAAAGTTATCCACCTGACCTAAAAGAATTGATTGAGGAATGCTGGGATCCCGAACCTGTTATGAGGCCTACTTTCTCCGAAATTATTGTAAGGTTGGATAAAATAGTTGCCAACTGCTCAAAACAAGGGTGGTGGAAAGACACATTCAAACTTCCCTGGAAATAA

>Csa1M467120.1

ATGGAGACGAATAACAGTAACAACAACGGGGTGAGGTTTTTGTTAGGGAAGCAATCGTCGATGGCGCCGGATCGCCAGCCGGAGGAGGCGGAGTTGGCGGAAGACGGTGAGGAGATCGATCCAGGGGTGCGATTGATGTATTTGGCGAATGAAGGCGATTTGGAAGGGATTAAGGAGCTTTTAGATTCAGGAATTGATGTTAATTTTCACGATATTGATAATCGGACGGCGCTTCATATTGCTGCTTGTCAAGGCTGCAACGAGATCGTCGATTTACTGCTCCGCCGTGGCGCTGAAATTGACCCCAAAGATCGCTGGGGCAGTACGCCTCTTGCAGATGCTATATTCTACAAAAACCATGAAGTTATCAAATTGCTGGAAAAAAGGGGCGCAAAACATCTGATGGCTCCCATGCATGTCAAACATGCTCGTGAAGTCCCAGAATATGAAATTGATCCTAAAGAATTTGACTTCACAAACAGTGTCAACTTGACCAAGGGAACCTTCCATCTTGCATCATGGCGCGGAATCCAAGTTGCAGTCAAAGAGCTTCCTGAGGATGTGATTTCAGAGGAGGATAAAGTGAACGCCTTCAGAGATGAGCTTGCATTGCTTCAGAAGATTCGGCATCCTAACGTGGTTCAATTTCTTGGTGCCGTCACTCAAAGTAGTCCCATGATGATTGTCACTGAATATTTGCCTAAGGGAGATCTTTGTCAACTTTTACACAAAAAAGGACCACTAAAACCAATTGTAGCTGTGAAATTTGCCCTTGACATTGCTAGGGGAATGAACTATTTGCACGAAAATAAACCTGCACCGATCATCCACCGTGATCTTGAGCCATCAAATATATTGCGAGATGACACTGGGAACCTGAAAGTTGCAGACTTTGGAGTTAGCAAGTTGCTCACAGTCAAGGAGGATAAACCTTTAACATGTCAAGACACTGCTTGCCGATACGTCGCTCCAGAAGTTTTCAAAAATAATGGATACGACACCAAAGTAGACGTCTTCTCCTTTGCTTTAATTCTACAAGAGATGATCGAAGGACAACCACCATTTTCAAACAAAAAAGAAAATGCCATTTGTAAAGGGTATGCAGCCGGAATGCGCCCTCCTTTCAAAGCTCCAGCTAAATGCTACGCCCATGGAATTAAAGAGTTGATTGAAGCTTGCTGGGATGAGAGGCCATCAAAGAGACCAACTTTTAGACAGATAATCACAAGGTTGGAAACAATTCATCACAGTCTTAGCCATAGAAGGCGTTGGAAGCTCCCAACGTTGAGATGTTTTCAGGATCCTGATGCCAAGATAAGAAGAGACCATCTCTCCAGTAGCCGGAGCCTTTCATCGCGATCTGCAAGCAGCATATGA

>Csa3M002480.1

ATGCCGATTGAAGATGATGTTGAGAGCTGCGGTAGCAGAGCCACCGACTTCTCCTCTTCCCATGTCAACCCGAGGCATCATCGTCAGAAGCTTGAAGTTTACAACGAAGTTCTTCGACGAATCCAGCAATCCAATTTCCATGAAGCTAATCTACCTGGGTTTGATGATCAGCTTTGGCTTCATTTCAATCGCCTCCCTGCTCGATACGCATTGGATGTGAATGTCGACAGGGCAGAAGATGTCCTTACCCATAAGAGATTACTTCAATTGGCTGTGGATCCGTCTAACCGACCTGTATTTGAAATTCGTTCTGTGCAGGTTTATCCTTCTGCTAATGAAAATTTTATCGATTCTTCTTGTTTGGATGCTTCTATGATGGAAGATGCTCAGAGTTCTTTGAACTATTCTAACAGACAGGGGAACCATCCACCTCCAACCTTTGGCTCTTCACCTAATCTTGAAGCCCCTACATTTCAGGGCAGCAAGTACGGTGTTGAAGATAGGGACAGTGCGCCAAATGTTACATCCAGCTTTTCTCGGCCAATGCATGAGATCACATTTGCAACAAGTGACAAGCCTAAACTCCTTAGTCAGGAAACTGAGGAGCTGAAAAGGGTATTAGAAAAAGAAATTTTAAATTTTAAGGAGCAGTGTTGGTCCGAAAAGCAGCCCAGTTCTGCTTTGGGTAAGCATAACCAGAATAGAGTTGAATCTTTTCCTAGTTGTGTTGGAATACCTACTGATGGTACTGATGTCTGGGAAATGGACATCAGTCAACTAAAATTTGAGAATAAAGTTGGATCTGGGTCATTTGGGGACCTGTATCGGGGCACGTATTGTAGTCAGGAAGTAGCTATTAAAGTACTGAGGCCTGAGCGTATTAATGAAGAGATGCTCAAAGAATTCTCTCAAGAAGTTTATATAATGAGGAAAGTTCGGCACAAAAATGTTGTTCAATTTCTTGGTGCTTGTACTAAACCTCCAAACCTCTGTATTGTGACAGAGTTTATGTCGAGAGGAAGTGTATATGACTTCCTTCATAAGCAAAGAGGTGTATTTAACCTTCCATCTTTACTTAAAGTGGCCATTAATATTTCAAGAGGGATGAACTATTTGCATCAAAATAATATAATCCATCGAGACCTCAAGACTGCAAATCTTCTGATGGATGAAAACATGGTTGTCAAGGTTGCTGATTTTGGAGTTGCCAGAGTTCAAACTCAATCTGGAGTGATGACTGCTGAAACTGGAACATACCGGTGGATGGCTCCTGAAGTTATTGAGCATAAACCATATGATCATAAGGCAGACGTTTTCAGCTTTGGAATAGCCTTATGGGAGCTTTTAACTGGAGAAATTCCATACTCATCCATGACCCCACTACAAGCAGCAGTTGGCGTGGTCCAGAAGAGACTGCGGCCTACCATACCAAAGAATGCCCATCCTGTTCTTGCTGAACTGCTTGAAAGATGCTGGAGGCACGATCCAACCGAACGGCCAAACTTCTCTGAAATTTTGGAGATCCTCAAGCAGATAGCTGAGCAGGTTGACAACAGCGGGGAAAATCGACGCAAGAAGGATAAGTTATCCGGTGCATTATTCTCAGCCTTCAAGAAGAGGCACCACTGA

>Csa7M017160.1

ATGGTTATGGAGGATAACGAGAGCTGTGGAAGTAGAGCGTACGACTTGTTATCACCGGCGCAAAGTCGGCAACAGAGGCAGAAGTTTGAGGTCTACAATGAGGTTCTACGGCGTTTGAAGGATTCCAACAACGAGGAGGCTATTCAGCCTGGTTTTGATGACGAACTATGGGTTCACTTCAATCGTCTTCCCACTCGATATGCTCTAGACGTAAATGTGGAGAGGGCAGAAGATGTTCTCATGCATAAAAGATTATTGCAATTCGCTCACGATCCTGCAAATCGACCTGCAATTGAAGTCCGCCTTGTGCAGGTCCAAGCCGTTTCTGATGAGCATTCTGCTGACTTTGCTGATTCATGTCCTGTAAAAGACACTGATCATAATTCTTCAAATTGCTTGAGCAGACAGAGCATGCATCCACCACCTGCCTTTGGGTCATCTCCTAACCTCGAGGCCTTGGCTCTTGAAGCTAATAATACTCAGGATCTGGAGGTTGATCAGTCTGTACATGCCAGAACACAGTTTTTCCGGCCAATGCATGAAATCACCTTCTCTACGGATGATAAGCCGAAACTTCTTAGTCAGGAAACAGAGCGGCTGAAGACTGCATTAGAGAGTGAAGTTTTACTGGTTGAGAGGCGAGGTTGGCCAAATCAGAAATCCTCGTCTCCTGTGGGGGAACTCGATATAACAGCTAAATGTGAGTCTGATCGGGTAGAAATACCCACAGATGGGACTGACGTATGGGAAATTAATCCTAGACATTTGAAGTTTGAGCATAAAGTTGCATCTGGTTCATATGGGGATCTGTATAAAGGTACATACTGCAGTCAGGAAGTGGCTATTAAAGTTCTCAAAACTGAACGTGTAAATACAGATATGCAAAGTGAGTTTGCCCAGGAGGTATATATTATGAGAAAAGTAAGGCACAAGAATGTCGTACAGTTTATAGGTGCATGTACTAAGCCCCCAAGCTTGTGCATTGTAACAGAATTTATGTCTGGTGGTAGTGTCTATGACTATCTACATAAACAAAAGGGGACTTTTAGGCTTCCATCTTTGCTTAAGGTAGCTATTGATGTCTCCAAGGGAATGAACTATTTGCACCAAAATAATATAATTCACAGAGATCTAAAAGCTGCCAATCTTCTGATGGATGAAAATGAAGTTGTCAAGGTTGCTGATTTCGGTGTTGCCAGAGTAAAAGCTCAATCAGGAGTTATGACCGCAGAAACCGGGACATATCGGTGGATGGCTCCTGAGGTTATTGAACACAAACCCTATGATCACAAGGCTGATGTTTTCAGTTTTGGAATTGTCTTGTGGGAGCTGCTAACTGGAAAGCTACCTTATGAATTCCTAACACCATTACAAGCCGCAGTCGGAGTAGTACAAAAGGGTTTACGGCCTACTATGCCTAAGCACACTAATCCCAAGCTAGCTGACTTGCTAGAAAAATGTTGGCAGCAAGACCCATCTTGCAGGCCTGACTTTTGTGAAATTATCGATATATTGTTGCAGATAACTAAGGAAGTTGCTGAAGAGGGTGAGGATCGAAGAAAGGAGAAAAGTGGAGGATTCCTTTCTGTGTTGAGACGAAACCATCACTGA

>Csa6M058190.1

ATGGCGGATATGGATTTTGTTGAAGGCGTTGGTGAGAGTTCTTCGCCGCCTCGAAGCTTTGCTGCTGGATTCTGTCCTGCCCACGATGTTCGGAACGATGTTTATACTAGGTTGGTTGAGTGTGGACATGAGGAGGCTGTTTCTAATCCTCAATTTCGCGAGAACTTGGATGCTCACTTCAATCGTTTGCCTCCGAGTTACGGACTTGATGTTAACATGGAGAAGGTGGAAGATGTTTTGTTACATCAAAAGCTTCTTTCCCTAGCAAAGGATCCAGAGAAGTGCCCTGTTTACCACATTCGTTTTCTTGAGCACATAAGTACTAAGTCAGATGGCAATGATGATCACGTTTTTCTTGATAGTATTCTTTTAAGTGGTTCATCTAATGAAGCAGCCGATAGAAGGCTTCCATTGTCACACAAAAGAACTCGAGGAAATATAATTGACTTTGAGGCTTGTTCCAAGCTTGAGGGCTTAAATTTGGATGTTAGAAAAAACTCGAAGCCCATGGATAGGAGGCCTGGTAATATTGGACATGTTCTGATACACGAAGTGATATTTTCAACGGTTGACAAGCCAAAGCTTCTTAGCCAGCTTTCTGCTTTGCTTTCTGATATAGGACTTAACATTCGAGAAGCACATGTATTTTCAACAACTGATGGTTACTCCTTGGATGTATTTGTGGTGGATGGATGGCCTATTGAGGAAACTGATGGCTTGTACGAAGCTATGGAAAAAGCAGTGGCTAGATATGAGGGATCGTGGTCTGGGTCTTCACATTCTCATCCAGTGGTCAAGAAGACATTAGACGCCCAGGTAAAATCAGCAGATTGGGAAATAGATAGACGGTTACTGAAGATAGGTGAAAGAATTGCATCAGGATCTTGTGGTGATTTGTACCATGGATTTTATCTTGGTCAAGATGTAGCTGTCAAGATATTAAGGTCTGAAGATCTAAATGCTGATTTAGAGGATGAGTTCAATCAAGAAGTGACTATTCTCAGGAAGGTCCAGCACAAAAATATAGTTCGTTTTGTAGGTGCTTGTACAAGCTCCCCACATTTGTGTATTGTAACAGAGTATATGCCAGGAGGGAGTCTTTATGATTACTTGCACAAGAATCATTGCGTGCTGAAGCTCTTGCAACTGCTGAAGTTTTCTATTGATGTTTGTGAAGGAATGGAGTACTTACATTTAAATAACATTATTCATAGGGACTTGAAGACAGCAAATCTGCTAATGGACACTCAACAGGTTGTAAAGGTGGCAGATTTTGGAGTTGCACGATACCAGAGTCAAGGAGTAATGACAGCAGAAACTGGAACATACAGATGGATGGCTCCTGAGGTAATAAATCATCTGCCATATGATCAGAAAGCAGATATTTTCAGTTTTGCGATTGTGTTGTGGGAACTAGTAACAGCAAAGGTTCCTTATGATTCCATGACACCATTACAAGCTGCCCTTGGAGTAAGACAGGGACTACGTCCAGATCTTCCCAAAAATGTTCACCCTAAATTGTTGGATATGATGCAAAGATGTTGGGATGCAGAACCTGTAAATCGGCCTCCATTTACCGAGATAAAAGTTGAACTCAAAAGTCTCCTAGAAGAAGTAGAGATGTGTACAAAATATGAAGAAAATTGTGCAGCAGTTAATGGGAGCTAA

>Csa3M728150.1

ATGAGTTGTAACGAGAAGAATAGAGGGGTAAATGATAGGGAAACCGAACACCCAGTTTTGACCAAGCCACATCAGAAACCAGTCATCCAGAATGGATCCATAACGGCCCAACACTTGACCATCGATAATAATCTTCTTGTTGACCCCAAATTGCTATTTATTGGATCCAAGATTGGCGAGGGCGCTCACGGAAAAGTTTATGAAGGCAGGTACCGGAATGAAATTGTCGCCATTAAAGTTCTCCATCGAGGGAGTACTCCAGAAGAAAGAGCAGCACTTGAAAGTCGTTTTGCCCGTGAAGTAAATATGATGTCCCGAGTAAAACATGAAAATCTTGTCAAGTTTATTGGAGCTTGTAAAGAACCTCTAATGGTGATAGTTACAGAGCTATTACCAGGGATGTCACTCAGGAAGTATCTGATGAATAATCGTAAACAACAGCTGGACCCTCGGATGGCCATTAACTTTGCTTTGGATGTTGCTCGTGCTATGGATTGTCTACATGCAAATGGGATTATACATAGAGATCTGAAACCTGATAATTTGTTGCTTACTGCAAATCAAAGGTCTGTGAAGCTTGCAGACTTTGGACTTGCTAGAGAAGAATCTGTGACTGAGATGATGACTGCAGAAACAGGGACTTACCGCTGGATGGCTCCTGAGTTGTATAGCACTGTTACATTGCGCCAGGGAGAGAAAAAGCATTACAACAACAAAGTTGACGTATACAGCTTTGGAATTGTCTTATGGGAGCTGTTGACCAACCGAATGCCATTTGAAGGGATGTCCAATCTGCAAGCTGCGTATGCGGCTGCTTTCAAGCAAGAGAGACCCAGTATTCCAGGCGACATATCTCCCGAGCTGGCATTTATAGTACAGTCGTGTTGGGTTGAAGATCCTAACATGAGGCCCAGCTTCAGCCAGATCATCCGCATGCTTAATGCTTATCTATTTACACTCCCACCTCCTTCACAATCTTCACCATCGTCACCAAAATCTGACACAACAGAGACAGCAACAACTAGCAATAGTGCCATTACCGAGTTCTCTTCTCGTGCAAGAGGAAAGTTTGGATTCCTTCGCCAACTGTTTGCTGCTAAGAGAGCTAAGAACTCGCAATAA

>Csa1M003510.1

ATGGAAGCTGGGAGTAGATTTTACTCTGCTACAGATGAGTTCAGATTGGAAGCCAAATGGTTGGTTGATCCCAAACATCTATTTGTTGGACCTAGAATTGGAGAGGGAGCCCATGCCAAAGTTTACGAGGGCAAATATAAGAACCAGACTGTTGCTATCAAAATTGTTCATAAAGGGGAAACAGTTGATGAGGTTGCAAAGAAAGAGGCTCGGTTTGCTCGTGAGGTTGCAATGTTGTCCAGAGTACAACATAAAAATCTTGTCAAGTTTATTGGTGCCTGCAAGGAGCCTGTAATGGTGATAGTAACCGAACTTTTATTAGGAGGGACCTTACGAAAGTACCTGCTCAACATGCGCCCACGGTGCTTGGACACACGTGTGGCAGTTGGTTTTGCACTTGATATTGCTCGTGCTATGGAATGCCTTCACTCACATGGCATCATACACCGTGATTTGAAGCCTGAGAACCTTCTGTTGACAGCAGACCACAAAACAGTTAAATTAGCTGATTTTGGTTTGGCAAGAGAAGAGTCGTTGACGGAGATGATGACTGCAGAGACAGGAACCTACCGTTGGATGGCTCCAGAGTTGTACAGCACTGTTACCCTAAGGCAAGGGGAGAAGAAACACTACAACCATAAAGTGGATGCCTATAGCTTTGCTATTGTGTTATGGGAACTGCTGCACAACAAGTTGCCATTTGAAGGCATGTCAAATCTTCAAGCAGCATATGCAGCTGCTTTTAAGAATGTAAGGCCTAGCGCAGAGAATCTTCCCGAGGAACTGGCTATCATTCTAACATCATGCTGGCAAGAGGATGCAAATGCTCGTCCAAATTTCTCCCAAATTATCCAAATGTTACTTAATTACCTTTATACTATTTCACCCCCTGAACCGGTGATTCCTTCCCGAATTTTCACTTCTGAGAACACGGTTTTTCCTCCTGAATCTCCTGGAACAAGCTCGCTGATGGCAGTCCGTGACGATTCAGGGGACACTCCCAAAGCTAAAATGGAAAATAATCCCAGAGGTTGTTTCTTCTGCTCCAATGACTGTTACTAA

>Csa2M049880.1

ATGTTTCGTTATAACCATCTCCACAGGGCATCGGGTTTAGCTATTCTTCAGTGTAAGGATGTTTTTTTGATTCCTTACAATGTGCCAAATGGATTTGGGAAAACTGAAGAATTTGGTTCGTTGAAAACTGAGAAACTTGGGAGTTGCTTTTTGGATGGTTACAAATTTTTGATGGATAGCCCTTTGACGCGTAGCTCTACCCCTAATTCCTCACCTATCTCAAATCCTGGTTCACATGATGAAAACCCACGCGTTAAGTTCCTGTGCAGCTTCTTGGGAAGTATAATGCCTCGCCCCCAAGATGGCAAGCTTCGGTATGTTGGGGGAGAGACAAGGATTGTGAGTGTTCCGAGGGATATTACCTATGAAGAGTTGATGGTTAAGATGAGGGAACTTTATGATGGGGCTGCAGTGTTGAAATACCAGCAACCAGATGAGGATCCTGATGCTCTTGTGTCAGTTGTCAATGATGACGACGTGATTAACATGATGGAAGAGTATGATAAGGTGGGTTCTGGTGATGGGTTCACTAGGCTCAGGATATTTTTGTTTTCGCATCCTGAACAAGATGCTTCTTTGCCTTTTGTGGATGGGGATGAGAGGGATACTGAGAGGCGATATGTAGATGCTCTTAATAATTCAAATGACATGAATGATTTTGTTAGACAACAACAACAAAATTCCCCTGCACTTAGTGGCATAGATGATATGCACGGGACTGAACATTTTTTGAATCCAATGAATATTGAAGGTAGTCTTCACACTCAGAGGAGTTGTGAGCCCTTGTCACAATACCACCTGCATCAGCTCACAATCCCTCATGTTGGGTCTGGGGGGCAACAACAATCTGTGGCTCAAAGGTATAGTGAAATGGAAGCTCCATGGAGTCCTGCTTTACTTTCACCTAGGCATCACGGTCCATATGATTCAAGACCAATGGGAGACTATCCATCTTCTCCATTTGCACGGTACCGTATGCCATTCCCCGATTTGCCAGATAAATATTTGGAAAGAATGCCTGAAGACTATGTCAGGCAACAAATGAATCACCAACATATGTATGAACATCAACCACAGTATAATGAGAATATTGTATGGCTGCCAAATGGAACAATTAATGAAGAATCGGGTTTCCCAGGTAATATATTACATGGACATGGTGTTCCAGATGGGAATAGTTCTTGTGAACACTGTCGAGCAAATTTTCATCGGTATCAAGCACACATGGAGCAAGTTAATACCTTAAATGGGCTTCCCCTTGAATACACTCAGAATAGGGAGGCCTTGATGCAGAAGGCAGACACAAAGTTCCATCATGGTATTTTTCCAAACGAGCAGAATATTAATGATCATCGATCTGCTTATAATGAGACACCACCCCATGAGAAAGGATGGATTATGCAACACCAAATGAGTGTGCGAGGGGATGACACGAGAACACATGTTTCTGGGACTGGGCGATTAACTGATCACTACATTGTGGATGGTTCTGGCTCAAATTTGCCTTCTACACAAAGTAATGTTGCTGATGGCTACCATGCTTCAACAAATTTTCATGATGAGGTATTTCGTGATCAGGTTGTGCCTAGTGGGCAACATATGTGTGTTCCTCCTCCTGAAGATCGTGGTGTTGGTTATATGCCTTATGGATATGGAGGAGAACCTCATTATCCTCCAATGGCACAAAGGCATATGCCCGGAAACGCCTCTTGGCGAAATGTTCAGAATCCATTGCATGTTGCTCCCCCATATGAAGCATCTGTTTTCCATCAACAAGGGAATGCAAGTATAAATCCAGGATATATCAAAGCCATGCAAGATGGTAGTCCACGAATTCATATGGGTGTGGACCACCAAAATCCATGGCATGAATCCTCACAAAAAGTTTTGGGTGTTGATGGAGCAACTGGTACAGAGCATTTACCTGCACATGTTTTGAAGACTAACTCGACCACAGTTGGCCATGACAATCAACAATTCACAAGTTTAGAACACATCCAACCACATCTTGATAAAATAAACCTTGTTGCTTCTCCTATGCAGAGGTCAGATTCATCTTCAGCATTCATTCAAGAAAAAATGGTTGCACCTTTTCATCCTAGCCAAAATCCACAACTAAGGGCTGTTAGTGCGGTTAATGAAGCTATGATGATGGAAAGGAAAGTTGTGCATGGGGAAGGAAATGGTCACATGATAAAGGATATGGGGAAACCTGACATCTCTGAAGCACACACTGCGTCTCATCCAGGGCAGAATAACACTGATGATACATATTCCAAGGTTGCACCCCTTGAATTGTTAAATTCAACTTGCACAAACTCTGCAGTAGAAAATGGTGATGGCTTGAAACCTTCTGTAGAGACTCTAGAAAAACCTAAACTATCTGTCAGCCGTCTGAGTTTCTTACCTGAGTTGATTGCTTCTGTGAAAAGAGCAGCACTAGAAGTATCTGAAGAGACAATGGTTGAAGAAACTGCTCTAAGGAGACCAGATTCAATAGAGAAAAAAGAGACAACAAATGAGCAACACTCCTCGAATAACCATGTGGAACCTGAGTTGGAAACGGAGAGTGAAAATCAAAGCTCCAGAATTGAGCCAACAAAAGCTGAAGAGGAAGCCATTTCAAGAGGATTACAAACAATTAAAAATGATGATCTGGAGGAGATTCGTGAACTAGGGTCTGGGACTTATGGTGCTGTTTATCATGGGAAGTGGAGAGGTTCTGATGTTGCAATAAAGAGAATCAAAGCTAGTTGTTTTGCTGGACGGCCTTCGGAAAGAGAACGTTTGATTGCTGATTTTTGGAAGGAAGCTTTGATATTAAGTTCTTTACACCATCCAAATGTTGTTTCCTTCTACGGCATTGTCCGTGATGGTCCTGATGGTTCCTTAGCTACAGTGACAGAATTCATGGTTAATGGATCATTGAAGCAGTTTTTGCACAAAAAGGACAGGACCATTGATCGTCGTAAGAGGCTCATTATAGCTATGGATGCTGCATTTGGGATGGAATATTTACATGGGAAGAATATTGTACATTTTGATTTGAAATGTGAAAACCTTTTGGTGAATATGAGAGACCCTCAGAGGCCTGTTTGCAAGATTGGTGATTTGGGATTGTCAAAGGTGAGACAACACACGTTAGTATCGGGAGGGGTTCGTGGAACTTTGCCTTGGATGGCGCCAGAGCTTCTTAGTGGGAAAAGTAACATGGTGACAGAGAAGATTGATGTTTACTCATTTGGAATTGTTATGTGGGAACTACTCACAGGAGATGAACCATATTCAGATATGCATTGTGCTTCAATAATCGGAGGAATTGTGAACAATTGTTTACGCCCTGAAATTCCGACATGGTGCGACCCAGAATGGAAGGCTTTAATGTCAAGCTGTTGGGACTCGGATCCAGCTAAGAGACCATCATTTTCAGAAATTTCTCAGAAGCTAAGGAATATGGCAGCTGCAATGAATAGACTGCATTGCTGGAAGACTACAAGTACATATATGAAGGAGATCCATGTAGTCATTGGAATGATTTCACTTGGGATTCTGCCGTTGGAAGCGTACGCCTTACAGTAA

>Csa6M517390.1

ATGGACGAAGAGGCTAATTCTTGGCTTAGGAGAACGAAGTTTTCTCACACGGTTTATCATCGATGGGACTCATTAAAATTAAACCCAGTTCCTTTCATCGTCGAACCACCTCGCAATTCTGGCTTGAAATCGAGGCCTCCATCGGCTTCATCTGCTCAGAAGCCGAATCCTGATATTTCGAAGATTCAGCGAAGTTTCATTTCCAATAAACAGAGGTCTCTGTCTCCTCTCCCTGAGAGCAACCTCTCTGAAGTTTTCAAGGAAGCAAAATCCGAGAGTAAGAGATTCTCAACTCCAACTCCTCGACTTAGAGAACGAACTAAGGAATTCAAGAACAAGCTATTCAATAAGGACCCTCAAGATTCAAAATCATCCAATTCCAAGTCATCGTTGAATACCAGTCCCTTGAAACAACTATCTTCAGGGAAAGGGGGTGATAAGTCAAAGTTGAAGAAGGACTCTTCCTGGACTAAATATTTCGATTCTGGAAAAGTCACTGCGGTTGAAACAGCGGATGACTGGACTGTTGATCTCTCCAAGTTGTTTGTTGGATTAAGGTTTGCTCATGGAGCTCATAGTCGACTTTATCATGGAAAGTACAACGACGAACCTGTTGCTGTTAAAATTATTAGAGTGCCTGATGATGACGAAAACGGAACGCTTGCGGCTCGATTGGAGAAGCAATTCACCAGAGAAGTTACCCTTTTATCGCGTCTCTATCATCCAAATGTTATAAAGTTTGTAGCTGCTTGTAGGAACCCACCTGTATACTGTGTCATTACTGAATATCTGTCTCAAGGATCATTAAGGGCATACTTACACAAACTTGAGCACCAATCCCTCCCTTTGCAAAAACTAATTAAATTTGCTTTAGATGTCGCTCGTGGCATGGAATACCTTCACTCACAAGGTGTAATTCATCGGGATCTTAAGCCTGAGAATGTTCTTATTGATGAAGACATGCACTTGAAAATTGCAGATTTTGGTATAGCTTGTCCTGAGGCCTTTTTTGACCCATTAGCTGATGATCCCGGAACTTACCGATGGATGGCACCGGAGATGATCAAACACAAACCTTGTAGTCGAAAGGTTGACGTGTACAGCTTTGGGCTTATGTTATGGGAAATGGTATCTGGTGCAATTCCATACGAGGATATGACTCCCATTCAAGCAGCCTTTGCTGTTGTGAATAAGAATCTTAGGCCTGTTATCTCAAGTGATTGTCCATTAGCAATGCGAGCTTTAATCGAGCAATGTTGGTCGCTACAACCGGACAAGAGACCTGATTTCTGGCAGATTGTGAAGGTATTAGAGCAATTCGAATCTTCGCTTGCTCGTGATGGGACACTGAATTTATTAGGTAACCCTCTGTCTAGTTTCCATGACCATAAGAAAGGGCTCCTCCATTGGATTCAAAAGCTTGGCCCCCTACATCCCGAAGTTTCATCCTCGCCCGTGCCAAAACCAAAATTCTCATAA

>Csa1M074900.1

ATGGAGGAAGATCCTAATTCTTGGTTAAGAAGAACTAATTTTTCTCACACAATATGCTATCGTTTAGAGTCCTTAAGTTTAGCTTCCTTTCCTGTCACCACTCAGCCACGTCCCAAATCACTTGTGCAATCAAAACCGAACCCTCGTTATAATCTAACAAAGCAGAGGTCTTTATCTCCTTCTCCACAAACGAATCTCTCCAATGCATTTAAGGATGCACGTATCAATCAGAAGCGATTTTCGACTCCACAGCCTCAGAGAAAAGAACCTCTAAAAGAGAAGAGTAAAAGGCTGTTTTGTAAAAGAGCCAAAGTGCAGAATTCCCTCAAGGAAGAGAAGTTAAAAGGACCTTTGAGGAATCTTGTCTCTTTTAAAGGTTGCGAGAAGTTCAAATTCAAGGAGTCTTCTTGGAGTAAGCTTTTTGAGCACGGTGGAGGGAAGGTCACGGCTGTTGAAGCTGTAGACGAATTGTCAATTGATCTGTCTAAGTTGATGTTTGGACATAGATTTGCTTTTGGAGCTCATAGTCGACTTTACCACGGAATTTATGAAGATAAAGTTGTTGCTGCTAAGATGATCAATCTACCAGCCAATGATGAAAATGGCGATCTCGCAGGACGCTTAGTAAAGCAATTTGGTAGAGAAGTAACCCTTTTATCTCGTCTTCACCATCCCAATGTTATAAAGTTAGTAGCAGCTGTGAAAAAGCCACCTGTTTATTGCATCATCACTGAGTATCTACCTCAGGGTTCCTTGAGAGCATATCTTCACAAACTAGAGAAAAAATCTCTCCCATTGCAAAAACAAATTGCCATTGCCTTAGACATTGCTCGTGGGATGGAATACATTCACTCACAAGGTGTCATTCATCGGGATCTGAAACCTGAAAACATTCTTATTGATCAAGACTTTTGTCTAAAGATCGCTGATTTTGGCATTGCTTGTGAGGAGGCACATTGTGATACACTGGCCGAGGACCCCGGAACGTTCCGTTGGATGGCCCCGGAAATGATCAAACGCAAACCCTATGGAAGAAAGGTTGATATCTACAGCTTTGGACTATTGCTATGGGAATTGGTAGCTGGAAAAATCCCTTATGAGGATATGACTCCAATCCAAGCAGCTTTTGCAGTTGTAGATAAGAATATTAGACCGGTCATACCGAGCGAGTGTCCTCCAGTGATACGGGTGTTAATCGAGCAATGCTGGTGCGAGAAACCGGAAAAGAGGGTGGAGTTTTGGCAGGTGGTGAAAGTGTTGGAGCAAGTTGAGAGTTGTATTGGGGGAGATGGTACATTGATGACGAGTGTAGAGTTAAAGGGGAAGGCAAGTTGGGAAGATCACAAGAAAGGTTTAAAGCATTGGATTCAGAAGCTTGGTCCTCTTAATTCTCATAATTCTCTCAACTCCTCCAGATCTAAATTCATATGA

>Csa6M502000.1

ATGTCGGACCATTCCGATGAACAACACTCCTTCAGAACAATTACCACTGCTTTCATTCCGTTCATTGGAGACGATGATTCTGATGATTCGGGTTCTGACTTCGTCTTCAGCATCGAACCTACTTTGCTCATTGACCCTCATTGTTTGAAGATTGGTGAAGTTATTGGGGAAGGTTCATGCTCCATTGTCTATGAAGGATTGTATGATTATCAGCCTGTCGCCGTGAAGATTATACAGCCAATTAGAGCATCAGCTATAAGTCCTGAAAAGAAGGAGAGATTTCAGAGGGAGGTTACGTTGCTAGCTAGACTGAATCATGAGAACATTATTAAGTTCATTGGTGCTTCCATAGAGCCAACATTGATGATAATCACTGAGCTTATGAGAGGTGGAACACTCCAGAAGTACTTATGGAGCATCCGGCCAGAGACCCCAGATTCGAAGTTTTCCTTAAGTCTTGCTCTAGATCTATCACGAGTAATGACATACCTGCATTCAAATGGCATCATTTATCGCGATTTAAAGCCAAGCAATCTACTGCTGACAGAAGACAAGCAACGGATTAAACTGGCAAACTTTGGGTTGGCTAGAGAGGAAATATCTGGTGAAATGACTACTGAGGCTGGTACTTACCGTTGGATGGCCCCTGAGTTATTTAGCATAGATCCATTGCCTGTTGGATGTAAGAAATGCTACGATCATAAGGCAGATGTCTACAGCTTCTCAATAATTCTTTGGGAATTGCTGACCAACAAGACTCCATTCAAAGGCAGAAATGACATAATGGTTGCATATGCCGTAGCCAAGAACATAAGACCATGTCTTGAAGAAATCCCAGAAGATATGGCTCCTCTATTGCAGTCATGTTGGGCTGAGGATCCCAATAGCCGTCCAGAATTTACAGAAGTCACAGATTATCTTAGTAATCTTCTTCAATCATTTGTCTTAAAAGAATCTTCACTTCCTAACATGGATGACAAAACAGAGGAGAAGGAGGAGGAGGAGAAGGTCAAGTGTAGATCAAACACATCGTTTTCTCAGAGAAAGAGAGAGCCAAAGGCTGGAAGATATAGAAACTCTTCATTCTGCTTCAAATGTTGCCACAACTCCTGCTTGTCTGATTAA

>Csa3M836460.1

ATGAAAGAAAAGAGTGAAACTGGAGGTGGGTATGTGAGAGCAGATCAAATAGATCTGAAGAGTTTAGATGAGCAGTTACAGAGACATTTGAGCAAAGCATGGACTATGGAGAAGAACAAGAGGAGGGAAGATGAGGAAGGTGTCGGCATCGGCGGCGGCGGCGGCGGCGGCGGTGGTGGAGGAGGTGTTGGCGGTGGAAGACCCGCAATCACTAGACAGGAGTGGGAGATTGACCCTTCCAAACTCATCATCAAAGCTGTCATAGCTCGTGGCACTTTTGGAACTGTTCATCGTGGAGTTTATGATGGTCAAGATGTTGCTGTTAAACTTCTTGACTGGGGAGAAGAGGGTCATAGGTCAGAGGCAGAAATCGCGTCCCTTAGAGCGGCTTTTACGCAAGAAGTTGCTGTTTGGCACAAGCTTGACCATCCTAATGTTACAAAGTTTATAGGAGCAACAATAGGCTCCTCAGATCTACACATACAAACAGAAAATGGCCAAATTGGCATGCCCAGCAATATTTGTTGTGTTGTTGTTGAATATTGTCCTGGAGGTGCTCTGAAATCCTACCTCATAAAGAACAGAAGGAAAAAGCTGGCTTTCAAAGTAGTCGTCCAGCTAGCTTTAGACCTTGCAAGAGGGTTGAGCTATCTTCATTCACAGAAGATTGTTCATAGAGATGTCAAAACAGAAAATATGCTTCTGGACAAAACGAGAACGGTAAAAATTGCAGACTTTGGCGTTGCTCGTGTTGAAGCCTCAAATCCCAATGATATGACTGGTGAGACTGGAACACTTGGGTATATGGCTCCGGAGGTTCTCAATGGCAATCCATATAACAGGAAATGTGATGTCTACAGCTTCGGCATCTGTTTATGGGAGATATACTGCTGCGATATGCCGTATCCCGACCTTAGCTTCTCAGAAGTTACATCAGCTGTTGTCCGACAGAATCTGAGGCCAGAGATTCCTCGGTGTTGCCCCAGCTCGCTTGCAAATGTAATGAAGCGATGCTGGGATGCTAACCCGGACAAGCGTCCAGAGATGGATGAGGTTGTAACCATGTTGGAAGCAATTGATACATCGAAGGGCGGAGGTATGATCCCCCTCGATCAGTCACAAGGCTGTTTTTGCTTCCGTAGGTACCGGGGGCCTTGA

>Csa7M387170.1

ATGGATTTAGCGAATGGGGGCGAGGCCGGCAAGGCTCTGGTGGCTAATCAAGTAGCTGATATTGTTTCTGGTAAATCTAGAAATACCCAAGAGAATGATTTGGGTTCAAAGTTGGGGACAGGAAGCAAAAGCAATAGGGATATGGTGTTTCGTGCTGATAAGATTGATTTCAAGAGTTGGGATATTCAGCTGGAGAAGCACTTGAGCAGGGCTTGGTCAAGGGACAGGGAAGTGCCTGCTAAAAAGGAAGAATGGGAGATTGACTTGTCTAAACTTGATATCAGATATGTTAAAGCTCATGGAACTTACGGTACTATTTACAGAGGAAACTATGACGGCAACGATGTTGCAGTGAAAGTGCTTGATTGGGGGGAGGATGGTGTTTCCTCAGTCGCTGAAATTGCTGCTCTTCGAACATCTTTTCGCCAGGAAGTTGCTGTATGGCATAAGCTTGACCATCCCAATGTTGCAAAGTTTTATGGAGCATCAATGGGGACGTCAAACCTTAAAATCCCTCCCAAAAGCTCATCATTCGACAGTAACCAAACTTTTCCTTCAAGGGCTTGCTGCGTTGTTGTTGAGTATCTTCCAGGTGGAACCCTAAAAAGCTTTTTAATCAAAAATAGGAAAAGGAAACTTGCATTTAAGGTTGTGATTCAACTTGCATTGGATCTTTCTAGAGGTTTGAGTTATCTACACTCCAAAAAGATTGTACACCGTGATATTAAAACAGAGAATGTTCTGCTAGATGCTCAGAAGACTCTGAAAATTGTTGACTTTGGCGTTGCTCGAGTCGAAGCACAGAACCCAAAGGATATGACTGGAGAGACTGGCACCCTTGGTTACATGGCCCCCGAGGTCCTTGATGGTAAGCCTTATAATCGGAAATGTGATGTTTACAGTCTTGGTATATGTTTGTGGGAAACATATTGCTGCGATATGCCTTACCCAGATCTCAGTTTTGCTGAAGTGTCTACTGCAGTTGTGCGGCAGAATTTACGACCAGATATCCCTAGGTGCTGCCCAAGTTCGTTTGCAAATATCATGAAAAGATGTTGGGACGCAAATCCAGAAAAGCGACCCGACATGGATGAGGTCGTAAAACTGTTGGAAGCCATTGATACAAGTAAAGGGGGTGGCATGATTACTGAAGATCAGATTAGCTGTTTCTGTTTCCGCCCGGCTCGTGGTCCATAA

>Csa3M146410.1

ATGGATTCGAAAGGAAATGTTATGGGGGGTGCTGCGATCCCAAAGGAGACACAGAACCAGGATAGAACTCCGAACTCAAAGGTGGCTGGTATGGGAAGCATTAGCAGCAAAGATATGATATTCAGAGCAGACATGATTGATTTGAAAACTCTAGATATACAGCTAGAAAAGCACTTGAGCCGTGTTTGGTCAAAAAGCATTGATAATCAAATGCCTAAGGAGCCGTGGGAGATTGATTTGTCTAAACTTGATATGATAAAACAAATTGCTCAAGGGACTTACGGAACAGTATACAGGGGTAAATATGACAACCAAGAGGTTGCAGTTAAGATATTGGACTGGGGAGAGGAGGGTCTAGCCACGATGGCTGAAACTGCTGCTTTGCGGGCATCATTTCGGCAAGAGGTTGCTGTTTGGCACAAGCTTGACCATCCTAATGTTACAAAATTTATCGGAGCTTCAATGGGAGCTACAAATCTTAAAATTCCAATGGATGGTCAAAATTCGTTTCCATCTAGGGCCTGTTGTGTTGTTGTTGAGTACGTTCCTAGCGGGACATTAAAAGATCATTTGATTAGATACTGGACAAAGAAACTTGCCATTAAGGCTGTAGTTAAATTAGCTTTGGATCTCTCTAGAGGGCTTAGCTATTTACACTCCAAGAAGATTGTGCATCGTGATGTCAAAACTGAAAATATGTTGATGGATATTAACGATAATGTCAAAATTGCTGATTTTGGGGTTGCCCGCGTTGAAGCTCAGAATCCAAGAGATATGACTGGGGCAACTGGTACCCTAGGATACATGGCCCCGGAGGTTCTTCAAGGGAAGCCGTACAATAGAAGTTGTGATGTCTATAGCTTTGGGATCTGCTTGTGGGAAATTTACTGCTGTGATATGCCATATGCCGATCTTAGTTTCGCTGATGTGTCATCTGCAGTTGTGAGACATAATCTGCGGCCTAGTATCCCGCGATGCTGTCCAAGCTCTCTAGCAAATGTGATGAAGAAATGCTGGGATGCTAACCCAGAGAAACGCCCGGAAATGCACGAGGTTGTCAGAATGTTGGAGGCCATTGATACAAGCAAAGGAGGAGGGATGATTAATCCCGATGACATTAAATGCTTCTGTCTAGGGCCTTTCGGCAAGCTCCGAGGTCTGTGA

>Csa3M146410.2

ATGGATTCGAAAGGAAATGTTATGGGGGGTGCTGCGATCCCAAAGGAGACACAGAACCAGGATAGAACTCCGAACTCAAAGGTGGCTGGTATGGGAAGCATTAGCAGCAAAGATATGATATTCAGAGCAGACATGATTGATTTGAAAACTCTAGATATACAGCTAGAAAAGCACTTGAGCCGTGTTTGGTCAAAAAGCATTGATAATCAAATGCCTAAGGAGCCGTGGGAGATTGATTTGTCTAAACTTGATATGATAAAACAAATTGCTCAAGGGACTTACGGAACAGTATACAGGGGTAAATATGACAACCAAGAGGTTGCAGTTAAGATATTGGACTGGGGAGAGGAGGGTCTAGCCACGATGGCTGAAACTGCTGCTTTGCGGGCATCATTTCGGCAAGAGGTTGCTGTTTGGCACAAGCTTGACCATCCTAATGTTACAAAATTTATCGGAGCTTCAATGGGAGCTACAAATCTTAAAATTCCAATGGATGGTCAAAATTCGTTTCCATCTAGGGCCTGTTGTGTTGTTGTTGAGTACGTTCCTAGCGGGACATTAAAAGATCATTTGATTAGATACTGGACAAAGAAACTTGCCATTAAGGCTGTAGTTAAATTAGCTTTGGATCTCTCTAGAGGGCTTAGCTATTTACACTCCAAGAAGATTGTGCATCGTGATGTCAAAACTGAAAATATGTTGATGGATATTAACGATAATGTCAAAATTGCTGATTTTGGGGTTGCCCGCGTTGAAGCTCAGAATCCAAGAGATATGACTGGGGCAACTGGTACCCTAGGATACATGGCCCCGGAGGTTCTTCAAGGGAAGCCGTACAATAGAAGTTGTGATGTCTATAGCTTTGGGATCTGCTTGTGGGAAATTTACTGCTGTGATATGCCATATGCCGATCTTAGTTTCGCTGATGTGTCATCTGCAGTTGTGAGACATAATCTGCGGCCTAGTATCCCGCGATGCTGTCCAAGCTCTCTAGCAAATGTGATGAAGAAATGCTGGGATGCTAACCCAGAGAAACGCCCGGAAATGCACGAGGTTGTCAGAATGTTGGAGGCCATTGATACAAGCAAAGGAGGAGGGATGATTAATCCCGATGACATTAAATGCTTCTGTCTAGGGCCTTTCGGCAAGCTCCGAGGTCTGTGA

>Csa3M840390.1

ATGGGTTCTGGTAATGGTTTTTATTCCACTAGTGAATTCAATTTGGATGCCAAATGGTTGATTGATCCTAAGCATCTTTTTGTTGGGCCAAAGATTGGGGAAGGTGCCCATGCCAAGGTGTATGAGGGAAAATACAAAAATCAGATTGTGGCTATCAAAATGGTGGGTAAAGGAGATACTCCTGAGAGGATGGCCAGAAGAGAAGCCCGGTTTGCAAGAGAGGTTGCAATGTTGTCCAAAGTGCGACACAAGAACTTAGTGAAGTTTATTGGCGCTTGCAAGGAACCTATGATGGTTATTGTGACTGAATTACTCACAGGTGGCACATTGCGCAAATTTCTCTTGAATTTGAGGCCAAGGAGCTTGGAACTTGATGTGGCAATTGGGTTTGCGCTTGATATTGCTCGCGCAATGGAGTGTTTACACTCCCATGGAATTATACACCGTGACCTTAAACCAGAAAACTTGATCTTGACTGCAGACCATAAAACAATTAAGCTTGCTGATTTTGGTTTGGCAAGAGAAGAATCAGTAACAGAGATGATGACTGCAGAAACAGGAACGTATAGATGGATGGCTCCAGAGCTCTATAGTACGGTCACTTTAAAGCATGGAGATAAGAAGCATTATAATCACAAAGTTGATGCCTACAGCTTTGCCATTGTATTGTGGGAGCTCATCCTTAATAGGTTACCTTTTGAAGGCATGTCGAATTTGCAGGCCGCGTATGCAGCTGCTTTTAAGAACATGAGGCCCAGTGCTGAAAACCTTCCAGAGGACTTAGCTTTAATAGTGACATCATGTTGGAGAGAAGATCCAAATACAAGACCAAACTTCAGCCAGATTATACAGATGCTATTGCAGTCCCTTTCTAGAATATCACCACGATCACCTGTGATCCCACCTCGGATATGTGCTTCTGAAAACGTTGTGATGCCACCCGAATCTCCCTGTACAAATTCCTTGATGGCTGTTAGACATGGCTCAGGGGAAGCCCCACATGGCATGATCGAAGAAACACCAACAACCTCCTTCTTCTGCTTTAACAAATGTTACTGA

>Csa3M840390.2

ATGGTGGGTAAAGGAGATACTCCTGAGAGGATGGCCAGAAGAGAAGCCCGGTTTGCAAGAGAGGTTGCAATGTTGTCCAAAGTGCGACACAAGAACTTAGTGAAGTTTATTGGCGCTTGCAAGGAACCTATGATGGTTATTGTGACTGAATTACTCACAGGTGGCACATTGCGCAAATTTCTCTTGAATTTGAGGCCAAGGAGCTTGGAACTTGATGTGGCAATTGGGTTTGCGCTTGATATTGCTCGCGCAATGGAGTGTTTACACTCCCATGGAATTATACACCGTGACCTTAAACCAGAAAACTTGATCTTGACTGCAGACCATAAAACAATTAAGCTTGCTGATTTTGGTTTGGCAAGAGAAGAATCAGTAACAGAGATGATGACTGCAGAAACAGGAACGTATAGATGGATGGCTCCAGAGCTCTATAGTACGGTCACTTTAAAGCATGGAGATAAGAAGCATTATAATCACAAAGTTGATGCCTACAGCTTTGCCATTGTATTGTGGGAGCTCATCCTTAATAGGTTACCTTTTGAAGGCATGTCGAATTTGCAGGCCGCGTATGCAGCTGCTTTTAAGAACATGAGGCCCAGTGCTGAAAACCTTCCAGAGGACTTAGCTTTAATAGTGACATCATGTTGGAGAGAAGATCCAAATACAAGACCAAACTTCAGCCAGATTATACAGATGCTATTGCAGTCCCTTTCTAGAATATCACCACGATCACCTGTGATCCCACCTCGGATATGTGCTTCTGAAAACGTTGTGATGCCACCCGAATCTCCCTGTACAAATTCCTTGATGGCTGTTAGACATGGCTCAGGGGAAGCCCCACATGGCATGATCGAAGAAACACCAACAACCTCCTTCTTCTGCTTTAACAAATGTTACTGA

>Csa5M523010.1

ATGGGGTCCGGTAATGAGTTATGTTCACAAGAGTTTGATTTGGATGCTAAGTGGCTGGTCGATCCGAAGCAAATCTTTGTTGGGCCAAGGATTGGGGAGGGCGCGCATGGCAAAGTGCACAAGGGAAAATATAAAGACCAAAATGTTGCAATTAAAATAATTCGAAAAGGTGAAGCCCCAGAAGAAATTGCGAAAACGGAGGCACGCTTTGCTCGGGAGGTCGCTATGTTATCCAAAGTTCAACACAAGAATCTTGCAAAGTTTATAGGTGCTTGCAAGGAGCCTATTATGGTGATAGTAACTGAGCTTCTTTCAGGCGGAACTTTACGGAAGTACCTGTTGAGTATAAGGCCAAGGTGCTTGGACTTCAGTGAGGCAGTTGGCTTTGCACTTGATATTGCCCGTGCAATGGACTGCTTACATTCCCATGGGATCATTCACCGTGACCTCAAACCTGAGAATTTGATCTTGACTGCAGATCACAAAACTGTTAAACTTGCTGATTTTGGCCTTGCTAGAGAAGAGTCAGTAACAGAGATGATGACAGCTGAAACTGGGACTTACAGATGGATGGCTCCAGAGCTATACAGTACAGTCACTCTACGAAATGGAGAGAAGAAGCATTACAATCACAAGGTGGATGTTTATAGCTTTGGAATTGTATTCTGGGAGATTATCCAAAACAAGTTGCCTTTTGAAGGCATGTCAAATCTACAAGCCGCATATGCGGCTGCTTTTAAGAATTTACGACCGAGTGCCGAGAACCTCCCAGCGGATTTGGCACCAATTGTGACTTCCTGTTGGAAAGAGGATCCGAATGATCGGCCTAACTTCAACCAAATCATACAGATGCTCTTTAAATGTCTATCCACCATTCCACAACCAGAATATGTTCCACCACCAACTATGCACCCACCTGATAATGCAGTGCTGCCACCAGAGTCTCCTGGAACAAGTTCTTTGATGGCAACCACGAGACATGGCACCGGGGAAGTCATGAACAGTGAAATAGGAGAGAAACCGACCGGTTTATTCTCGTGTTTCGCTGGTAACTGTTACTGA

>Csa6M520410.1

ATGACGAGTCCAGTTAAGTTTAAGTTGGGGAAACAGTCGTCCCTAGCGCCGGATGGAGATGTCCATCTTGAAGATTTGGAGGAGCTGGTCAAGCAGCATCAGACTGAGGAGGGGATAGATTCGAGGGTGAGGTTGATGTATTTGGCCAATGAAGGTGATTTGGAAGGGATTAATGAAGTCTTGGATTCGGGAGTTGATGTTAATTTCCGTGATATCGACAACCGGACGGCTTTGCATATTGCTGCTTGCCAGGGATTTGCCGACGTTGTCGCTTTGTTGCTCGAACGTGGCGCTGAAGTTGACTCCAAAGATCGCTGGGGGAGCACGCCTCTTAGAGATGCTATACATTACAAAAACCACGATGTGATCAAACTTCTGGAGAAGCATGGCGCAAAGCCTCCGGTGGCCCCCATGCTCGTCAAGAATGCCCGTGAAGTGCCAGATTATGAAATTGATCCTAAGGAACTTGATTTTACTAACAGTGTGAACATTACCAAGGGAACATTCCGCAGAGCATCTTGGCGTGGAACTGAGGTTGCTGTGAAAGAGCTTGGAGAAGATCTATTCACCGATGAGGAGAAAGTGAGGGCTTTCAGAGATGAGCTTGCATTGCTTCAGAAAATACGACACCCCAACGTTGTCCAGTTTCTAGGTGCTGTTACTCAAAGTTGGCCGATGATGATCGTTACAGAATATTTACCAAAGGGAGATCTTGGAGCATTATTGAGTAGAAAGAGAGAAATAAAAACAATGTCTGTTGTGAGACTTGCCCTTGATATTGCAAGGGGGATGAACTACTTGCATGAGAACAAGCCAGCACCAATTATTCATCGTAATCTTGAGCCTTCAAACATTCTGAGGGATGATTCTGGCCACCTGAAAGTAGCAGATTTCGGAGTTAGCAAGTTATTGACAGTTAAAGAAGATAAATTTTCAACTTGTTCAGAGACTTCACGAAGATACCAAGCTCCAGAGGTTTTCAAAAATGAAGAATATGACACAAAGGTGGACGTGTTTTCATTTGCTTTGATTTTGCAAGAGATGCTTGAAGGCTGTTCTCCATTTCCTGACAAAGCAGATAGTGAAGTTCCTAAATTGTACGCAGCAGGAGAACGTCCCCCATTTGGAGCATTAATTAAACGTTACGCAAATGGACTAAAAGAGCTAATTGAAGAATGTTGGAACGAGAAGCCAAATAAGAGACCAACTTTTAGGCAGATAATAACCCAGCTGGAATTCATTTACAACAGATTCTGTCATAAGAGGCGCTGGAAGGTCAGGCCATTGAAATGCTTCCAGAATATTGAGGCAATGCTGAAGAAAGATCGTCTCCGCCGAAGCAGTTTCAATCTATCTTCACATTCTTCTGCCAGCAAATTTGCGAGCTTCTGGGATGAGAAACATGGCAATCAACAGTATCAAATCCACATGGGAAATCGGATTTTCGCTATGTTTGTTCATCACATGGCCTTCAGAAATTCGAAGCTTTTAATCCTCACGATTATGGATCTATTTTTTCCATGCCACTGTTTGGTCAACATTGTGAATAGGAGTGTCACTGTTAGACCTACTTTCAACGTCACTGTTAGACCTACATTCAACATTGTGAAAATTACCCATCTCTTTGTTAAAAATCAGAAAAATATGCAACAGAGCAAGCAACCTTCACTGAAATCTACTGCTCTTAGAAGTGGTGGCAGTTCATTCATGTACTTCTCCCGTACACAAGGGAAGCCTTTGGGAAGAGAAGGTGCGCAGTGTTTATATGGGTTGAAAGACAACCTCTCTTATGAGAGGTATAGAGCTCAGGCATGGTGGAAAGGAAAAAGTATGTCAGTAAAGCAGAGTTTCTTTCTGGCCCAAAACTGCTACTCAAGAGCTTTAGCAACTCAGGCCATTGCATACCTGCAACAAGGTAAGTTGCAAATAAAGGGGATGTTCCAGAATCAAGAAGAGAAGATGTCAGACTCGCCTCAGAGGAAGATGGGAAGAGGAAAGATTGAGATTAAGAGGATTGAAAATACAACAAATCGTCAAGTCACTTTCTGTAAGAGAAGAAATGGGTTGCTTAAAAAAGCTTATGAACTTTCTGTTCTTTGTGATGCTGAAGTTGCTCTCATCGTTTTCTCAAGCCGTGGCCGCCTCTATGAATATGCTAACAACAGTGTGAAGGCAACAATTGATAGATATAAGAAAGCATCCTCAGATTCCTCCAACACTGGATCTACTTCTGAAGCTAACACTCAGTTTTATCAACAAGAAGCTGCCAAACTCCGAGTTCAGATTGGTAACTTACAGAACTCAAACAGGAACATGCTAGGCGAGTCTCTAAGTTCTCTGACTGCAAAAGATCTGAAAGGCCTGGAGACCAAACTTGAGAAAGGAATTAGTAGAATTAGGTCCAAAAAGAATGAACTCCTGTTTGCTGAGATTGAGTATATGCGAAAAAGGGAAATTGATTTGCACAACAACAATCAGATGCTTCGGGCAAAGATAGCTGAGAGTGAAAGAAATGTGAACATGATGGGAGGAGAATTTGAGCTGATGCAATCTCATCCGTACGATCCAAGAGACTTCTTCCAAGTGAACGGCTTACAGCATAATCATCAATATCCACGCCAAGACAACATGGCTCTTCAATTAGTATAA

>Csa6M520410.2

ATGATGATCGTTACAGAATATTTACCAAAGGGAGATCTTGGAGCATTATTGAGTAGAAAGAGAGAAATAAAAACAATGTCTGTTGTGAGACTTGCCCTTGATATTGCAAGGGGGATGAACTACTTGCATGAGAACAAGCCAGCACCAATTATTCATCGTAATCTTGAGCCTTCAAACATTCTGAGGGATGATTCTGGCCACCTGAAAGTAGCAGATTTCGGAGTTAGCAAGTTATTGACAGTTAAAGAAGATAAATTTTCAACTTGTTCAGAGACTTCACGAAGATACCAAGCTCCAGAGGTTTTCAAAAATGAAGAATATGACACAAAGGTGGACGTGTTTTCATTTGCTTTGATTTTGCAAGAGATGCTTGAAGGCTGTTCTCCATTTCCTGACAAAGCAGATAGTGAAGTTCCTAAATTGTACGCAGCAGGAGAACGTCCCCCATTTGGAGCATTAATTAAACGTTACGCAAATGGACTAAAAGAGCTAATTGAAGAATGTTGGAACGAGAAGCCAAATAAGAGACCAACTTTTAGGCAGATAATAACCCAGCTGGAATTCATTTACAACAGATTCTGTCATAAGAGGCGCTGGAAGGTCAGGCCATTGAAATGCTTCCAGAATATTGAGGCAATGCTGAAGAAAGATCGTCTCCGCCGAAGCAGTTTCAATCTATCTTCACATTCTTCTGCCAGCAAATTTGCGAGCTTCTGGGATGAGAAACATGGCAATCAACAGTATCAAATCCACATGGGAAATCGGATTTTCGCTATGTTTGTTCATCACATGGCCTTCAGAAATTCGAAGCTTTTAATCCTCACGATTATGGATCTATTTTTTCCATGCCACTGTTTGGTCAACATTGTGAATAGGAGTGTCACTGTTAGACCTACTTTCAACGTCACTGTTAGACCTACATTCAACATTGTGAAAATTACCCATCTCTTTGTTAAAAATCAGAAAAATATGCAACAGAGCAAGCAACCTTCACTGAAATCTACTGCTCTTAGAAGTGGTGGCAGTTCATTCATGTACTTCTCCCGTACACAAGGGAAGCCTTTGGGAAGAGAAGGTGCGCAGTGTTTATATGGGTTGAAAGACAACCTCTCTTATGAGAGGTATAGAGCTCAGGCATGGTGGAAAGGAAAAAGTATGTCAGTAAAGCAGAGTTTCTTTCTGGCCCAAAACTGCTACTCAAGAGCTTTAGCAACTCAGGCCATTGCATACCTGCAACAAGGTAAGTTGCAAATAAAGGGGATGTTCCAGAATCAAGAAGAGAAGATGTCAGACTCGCCTCAGAGGAAGATGGGAAGAGGAAAGATTGAGATTAAGAGGATTGAAAATACAACAAATCGTCAAGTCACTTTCTGTAAGAGAAGAAATGGGTTGCTTAAAAAAGCTTATGAACTTTCTGTTCTTTGTGATGCTGAAGTTGCTCTCATCGTTTTCTCAAGCCGTGGCCGCCTCTATGAATATGCTAACAACAGTGTGAAGGCAACAATTGATAGATATAAGAAAGCATCCTCAGATTCCTCCAACACTGGATCTACTTCTGAAGCTAACACTCAGTTTTATCAACAAGAAGCTGCCAAACTCCGAGTTCAGATTGGTAACTTACAGAACTCAAACAGGAACATGCTAGGCGAGTCTCTAAGTTCTCTGACTGCAAAAGATCTGAAAGGCCTGGAGACCAAACTTGAGAAAGGAATTAGTAGAATTAGGTCCAAAAAGAATGAACTCCTGTTTGCTGAGATTGAGTATATGCGAAAAAGGGAAATTGATTTGCACAACAACAATCAGATGCTTCGGGCAAAGATAGCTGAGAGTGAAAGAAATGTGAACATGATGGGAGGAGAATTTGAGCTGATGCAATCTCATCCGTACGATCCAAGAGACTTCTTCCAAGTGAACGGCTTACAGCATAATCATCAATATCCACGCCAAGACAACATGGCTCTTCAATTAGTATAA

>Csa6M520410.3

ATGACGAGTCCAGTTAAGTTTAAGTTGGGGAAACAGTCGTCCCTAGCGCCGGATGGAGATGTCCATCTTGAAGATTTGGAGGAGCTGGTCAAGCAGCATCAGACTGAGGAGGGGATAGATTCGAGGGTGAGGTTGATGTATTTGGCCAATGAAGGTGATTTGGAAGGGATTAATGAAGTCTTGGATTCGGGAGTTGATGTTAATTTCCGTGATATCGACAACCGGACGGCTTTGCATATTGCTGCTTGCCAGGGATTTGCCGACGTTGTCGCTTTGTTGCTCGAACGTGGCGCTGAAGTTGACTCCAAAGATCGCTGGGGGAGCACGCCTCTTAGAGATGCTATACATTACAAAAACCACGATGTGATCAAACTTCTGGAGAAGCATGGCGCAAAGCCTCCGGTGGCCCCCATGCTCGTCAAGAATGCCCGTGAAGTGCCAGATTATGAAATTGATCCTAAGGAACTTGATTTTACTAACAGTGTGAACATTACCAAGGGAACATTCCGCAGAGCATCTTGGCGTGGAACTGAGGTTGCTGTGAAAGAGCTTGGAGAAGATCTATTCACCGATGAGGAGAAAGTGAGGGCTTTCAGAGATGAGCTTGCATTGCTTCAGAAAATACGACACCCCAACGTTGTCCAGTTTCTAGGTGCTGTTACTCAAAGTTGGCCGATGATGATCGTTACAGAATATTTACCAAAGGGAGATCTTGGAGCATTATTGAGTAGAAAGAGAGAAATAAAAACAATGTCTGTTGTGAGACTTGCCCTTGATATTGCAAGGGGGATGAACTACTTGCATGAGAACAAGCCAGCACCAATTATTCATCGTAATCTTGAGCCTTCAAACATTCTGAGGGATGATTCTGGCCACCTGAAAGTAGCAGATTTCGGAGTTAGCAAGTTATTGACAGTTAAAGAAGATAAATTTTCAACTTGTTCAGAGACTTCACGAAGATACCAAGCTCCAGAGGTTTTCAAAAATGAAGAATATGACACAAAGGTGGACGTGTTTTCATTTGCTTTGATTTTGCAAGAGATGCTTGAAGGCTGTTCTCCATTTCCTGACAAAGCAGATAGTGAAGTTCCTAAATTGTACGCAGCAGGAGAACGTCCCCCATTTGGAGCATTAATTAAACGTTACGCAAATGGACTAAAAGAGCTAATTGAAGAATGTTGGAACGAGAAGCCAAATAAGAGACCAACTTTTAGGCAGATAATAACCCAGCTGGAATTCATTTACAACAGATTCTGTCATAAGAGGCGCTGGAAGGTCAGGCCATTGAAATGCTTCCAGAATATTGAGGCAATGCTGAAGAAAGATCGTCTCCGCCGAAGCAGTTTCAATCTATCTTCACATTCTTCTGCCAGCAAATTTGCGAGCTTCTGGGATGAGAAACATGGCAATCAACAGTATCAAATCCACATGGGAAATCGGATTTTCGCTATGTTTGTTCATCACATGGCCTTCAGAAATTCGAAGCTTTTAATCCTCACGATTATGGATCTATTTTTTCCATGCCACTGTTTGGTCAACATTGTGAATAGGAGTGTCACTGTTAGACCTACTTTCAACGTCACTGTTAGACCTACATTCAACATTGTGAAAATTACCCATCTCTTTGTTAAAAATCAGAAAAATATGCAACAGAGCAAGCAACCTTCACTGAAATCTACTGCTCTTAGAAGTGGTGGCAGTTCATTCATGTACTTCTCCCGTACACAAGGGAAGCCTTTGGGAAGAGAAGGTGCGCAGTGTTTATATGGGTTGAAAGACAACCTCTCTTATGAGAGGTATAGAGCTCAGGCATGGTGGAAAGGAAAAAGTATGTCAGTAAAGCAGAGTTTCTTTCTGGCCCAAAACTGCTACTCAAGAGCTTTAGCAACTCAGGCCATTGCATACCTGCAACAAGGTAAGTTGCAAATAAAGGGGATGTTCCAGAATCAAGAAGAGAAGATGTCAGACTCGCCTCAGAGGAAGATGGGAAGAGGAAAGATTGAGATTAAGAGGATTGAAAATACAACAAATCGTCAAGTCACTTTCTGTAAGAGAAGAAATGGGTTGCTTAAAAAAGCTTATGAACTTTCTGTTCTTTGTGATGCTGAAGTTGCTCTCATCGTTTTCTCAAGCCGTGGCCGCCTCTATGAATATGCTAACAACAGTGTGAAGGCAACAATTGATAGATATAAGAAAGCATCCTCAGATTCCTCCAACACTGGATCTACTTCTGAAGCTAACACTCAGTTTTATCAACAAGAAGCTGCCAAACTCCGAGTTCAGATTGGTAACTTACAGAACTCAAACAGGAACATGCTAGGCGAGTCTCTAAGTTCTCTGACTGCAAAAGATCTGAAAGGCCTGGAGACCAAACTTGAGAAAGGAATTAGTAGAATTAGGTCCAAAAAGAATGAACTCCTGTTTGCTGAGATTGAGTATATGCGAAAAAGGGAAATTGATTTGCACAACAACAATCAGATGCTTCGGGCAAAGATAGCTGAGAGTGAAAGAAATGTGAACATGATGGGAGGAGAATTTGAGCTGATGCAATCTCATCCGTACGATCCAAGAGACTTCTTCCAAGTGAACGGCTTACAGCATAATCATCAATATCCACGCCAAGACAACATGGCTCTTCAATTAGTATAA

>Csa4M332110.1

ATGGCACAATCGAATATTGGGTATGCTGAAACAGATCCTTCTGGTCGCTATGGACGGTTTAGAGAAATTCTAGGCAAAGGAGCCACGAAAACAGTATATAAAGGCTTCGATGAGGTTCTTGGAATTGAAGTGGCTTGGAACCAAGTCCATCTCAAAGATGTCTTCCATTCCCCTGAAGAACTACAACGCTTATACTCAGAAGTTCATCTCCTCAAGAACCTCAACCACGACTCGATCATCCGATTCTACACTTATTGGATCGATACTCATCGCCGAACGTTCAACTTCATCACAGAAATGTTCACATCTGGCACCCTTAGAGAGTATCGACAAAAGTATCGAAACATCGACATCGAAGCAATCAAGAACTGGGCTCGTCAAATCCTACATGGTCTTGTTTATCTTCATGGTCATGATCCACCCATAATCCATAGAGACCTAAAGTGTGATAATGTATTCATCAATGGCCATCTTGGGCAAGTTAAGATTGGTGACCTTGGACTTGCAGCAATCCTTCATGATTCTCAACATGCTCATAGTGTCATAGGTACACCAGAGTTCATGGCACCAGAACTTTATGACGAGGAATATAATGAACTTGTGGATGTTTATTCGTTTGGGATGTGTATGATTGAGATGCTTACTTTGGAGTATCCTTATAGCGAATGCTTTAATCCTGCTCAGATATACAAAAAAGTCACTTCAGGAAAATTGCCAAATGCATTCTATGAGATCAAAGACTTGGAGGCCCAAAGATTTGTAAGAAAATGTTTGGAGAATGTTTCAAAGAGGGTGTCTGCTAGAGAGCTCTTGCTTGATCCATTTCTAGCACCTTCCAACGCTAACAATGCCTCTCACAATGAAGAGTTGTTGTCTTCATCTTTATCTCCAGAGAAATCAATAATGGCAAGAAGAACAGATTTAGCCATTTCAGGATCAATCAATCCCAAGGATGATTCAATTTTCCTTAAAGTGCAGATCAAAGTGAAGAACGGTAAATCTAAGAACGTATATTTTGCATTCGACATCTTAAACGACACTACAATCGACGTTGCGACCGAGATGGTTAAAGAGTTGGAAATTATCAGTGATTGGGATCCATTGGAGATAGCTGTGATGATAGAGAAGGAGATATCCTCATTGATTCCAGATTGGGAGGAATGGAAGTTACCAAAAATCCAACATCAAGATAGCTTCAACTATGAACAAGATCACGATGGCGACAACGATAACGACAATGACGATGACGACGAAAACTATGCAACACCACATCCTTTCTATTATTGCGGCTCTTCTCATGGCTCCTCCTCAGATTCTCTTCATGCTTTCTACTCTTCCCGTGAGAACCCCAATCATTATTTTGGAGGGATGAAGGACACTTCTAATACTACTGAGTGGTTTCGAGAGGATGATACAAGTTCTTGTTGCTCTCTCAATTCCTTCAATTACTCTGATTTAAGTTTTTACTCAAACAATGAAGATGAGTATGAATATGACTCAAATATCAAAGGAAGAGAGCCTCAATATGTCTCAACAACCAAACAACCTACAAGATTTTGCCCAACCATGAAGATAGACTCACACCATTTGAGGCACAAGGACAATAAAATTATCCCCAATCGAGAAGTATTCGAGAGTCGATCTCGATCAAATAACTCGCCTAGACTTACAAGGGTGAAATCGATGGTGAACCTTCGTAGTGAGACGTTGCACCGGTACTTAGTAGAGATGTTGTTGAAGAAGCGCTTGTTCAATACTGTGGGTGCAATGGAGAATATTGGATACCAAAAGCCATAG

>Csa6M212860.1

ATGTTTAAGAATACTCGAGCTGGGAGTCCCAGCAGCAACCTCCATGATTGCAAATCAGAATTTGGGTACGTTGAGACCGATCCGACATGTCGTTACGGCCGTTTCGAAGAAGTTTTGGGAAAAGGGGCCATGAAGACAGTGTACAAAGCCATTGATGAGTTTCTTGGAATAGAGGTGGCGTGGAGTCAGGTGAAACTCAACGAGGTCCTCCGATCGCCCGAGGATTTGCAACGGCTGTATTCAGAGGTTCATCTACTTAGCACGCTTAAGCACGAATCGATCATGCGATTCTACACCTCATGGATTGATGTTGATAAAAAAACCTTCAACTTCATTACTGAACTGTTTACTTCTGGAACGCTCAGAGAATACGGAAAGAAATATAGGCGAGTTGATATCAGAGCCATTAAGAGCTGGGCGAGGCAGATTCTGCAGGGACTCATTTATTTGCACGAACACGACCCTCCAATAATACACAGAGATCTGAAGTGTGATAATATCTTTGTGAATGGCCATCTTGGGCAAGTCAAGATTGGTGACTTGGGGCTTGCAGCTATACTTCATGGTTCCAGATCTGCTCACAGTGTTATAGGCACACCGGAGTTTATGGCACCGGAATTATACGAGGAGAATTACAATGAGCTAGTTGATGTCTACTCGTTTGGCATGTGTGTCCTAGAGATGCTTACTTCGGAGTACCCTTATAGCGAATGTTCTAATCCTGCACAAATTTACAAGAAAGTCACGTCGGGGAAGCTACCAGCAGCATTATACAAGATTCAAGATGTGGATGCGCAGAGATTTATTAAGAAATGCTTGGTACCAGTTTCGATGAGGGCGTCTGCGAAGGAACTCTTGGCTGATTCTTTTCTCAAAGTTGATGGAAATAGACCTTCATCGGTGGGGAGGACTCAAAACCAGAAGCCATTTCTAAATGCCAAAGAAATGGAGAATTTTCACTTGAGTGAAGGTTTAAGCAGGACCAACATGACGATCACGGGAAAGCTGAACCCCGAAGACGATACCCTCTTTCTTAGAGTTCAGACTGCCGATAAAGATGGCTCGCTTAGAAACATATACTTCCCTTTCGACATAGTTAATGACACAGCATTAGATGTGGCAATGGAGATGGTGAAAGAATTGGAGATCTCTGATTGGGAGCCGTTTGAAATTGCGGACATGATTGAAGGTGAGATATCGGCTCTGGTTCCAAACTGGAATAGAAGTGAGTTAACCAACCATAGCCTCGGCTTCAGCTGTGCAGAAGAAGATGATAATGTATCTCATCATACTTTCCGCTCGATCTCCTCGTCGTCCCAAGCAACAACTTTGGGTCTTATCTCCTCCCCAAGAACAAACCAAAACATTTCAAATGGTTTTAGTTGGTTCCCAGATGATACGCTTGATGATAGCAGTTCACAATGCTCATCAGCGTCGGGAAAATACTCCAACTTGAATTACATCAGCAGCGACGAGTATGAGACCAGCATGAGTTCAGTCCAGACAGATCAACATAACAACATCAATAAGATTCACAATTCCTCAAGATTTTGTCCTATTGAGAACCGCAAAAGCAAGGATTTTTTGGCTCAACTTCTATACAAGCAGAGCCAATGTGCGATAGCAGGGTCGTCTCAAGGAGTTGCCTCTGGTCGCAAAGACAAGAAAGGAACAGATGGTCGTAAATTAACTAGGAACAGATCACTAGTAGATGTACATAGCCAATTGCTGCATCGTTCGTTAGTGGAGGAAGTGAATAGAAGACGGTTATTCAAGACAGTTGGAGCAGTCGAAAGTATAGGCTTTCAAGCACCTTGTGAGGTTTCTTCGAGCAAAAGGGTCTCGAGTAGGCAACCGATTGGGAACCGAAGTAGTGATGTAGCAAGAACGAGGAGAAACGAGGATATTAGATGGCAAGATGTTGGAAGGAGAACATGA

>Csa2M012110.1

ATGGATGGTGTCGTCTCAGATCTTGAGCTTGATTGCTCTGAATTTGTTGAAGTTGATCCCACTGGCAGATATGGCAGATACAATGAAATTTTGGGAAAAGGGGCTTCAAAAACAGTTTACAGAGCATTTGATGAATATGAAGGAATTGAGGTGGCTTGGAATCAAGTGAAACTTTATGATTTCTTGCAAAGTCCTGAAGATCTGGAAAGACTTTACTGTGAGATTCATCTTTTGAAGACATTGAAGCATAGAAATATTATGAAATTCTATACTTCTTGGGTTGATATTGCCAATAGAAACATCAATTTCGTTACTGAAATGTTCACTTCCGGCACTCTTAGACAGTATAGGCTGAAACATAAGAGAGTTAATATTAGAGCAGTGAAGCATTGGTGCAGGCAGATTTTGAGAGGGCTTCATTATTTGCATAGCCATGAGCCTCCTGTAATCCATAGAGATCTCAAGTGTGACAATATCTTTGTTAACGGGAATCAAGGGGAAGTTAAGATTGGTGATCTTGGACTTGCAGCAATCCTCAGGAAATCTCATGCTGATCATTGTGTGGGGACCCCTGAGTTTATGGCTCCAGAAGTGTATGCAGAGGCGTACAACGAATTAGTCGATATATACTCATTTGGAATGTGCATTCTGGAGATGATAACTTTCGAATATCCATACAGCGAATGCACACATCCCGCTCAAATCTATAAGAAAGTCATATCTGGGAAAAAACCAGATGCCTTGTACAAAGTGAAGGATCCTGAAGTGCGGCAGTTCATCGATAAGTGTTTGGCTACAGTTTCATATCGGCTCTCGGCAGCAGAGCTTTTGAATGACCCTTTTCTTCGAACTGACAATGGCGAATATGATCTAAGACCTGTCGATTATGGAAGAGGATTGGACGATGTTTGCCCTCTTATAAGACAGCCTTATCTAGAACTTCATAGAAGTGACAGCTCCTTTTGTACTGGATATCCATATGATTACAGCTTTGAAGCTTCAAGTGAATCTGGTTATCATCCAATTGATAATGGAATTGAACTTTTTGAGTATTGTGAAGGTGAACATTCTGAGGATGTTGATATTAGCATTAAAGGGAAGATGAGTGAAGATGGTGGCATCTTCTTAAGACTTAGAATTGCAGATAAAGAAGGGCGTATCAGGAACATTTATTTTCCATTTGATGTCGAGACAGATACAGCATTGAGTGTTGCGACAGAAATGGTTGCGGAGCTAGATATGACAGATCAAGACGTGACAAGAATTGCTGATATGATCGATGGAGAAATTGCTTCCTTGGTGCCTGAATGGAGGCCAGGTCCGGGAATTGAGGAAACGCCCCGCTTTGCAAACCAAAGCTATTGTCATAATTGTGCTCCTTCTACTTACAACAGTGCTTCAAATGGTCTTATGTTGAGAAATCATGATGGAAAGAACTCAGAGGTAGCTCAATGTTGTGGACATCGATATGCTTCTATGCATGGTCGTTTTGAGGAGATAATGTATCATGCTGACGAGCCAGAGCATCATACAGCAGAGGATGCACCAAATGTGTCGAGCCATCCTGATGGGCTGAGTTATCCTGAAATATGGGGACATCATGAAAGCCGTGAACTTAGTTCAATGAGCTCACGACAAAGCCATTCAGATGAAGATTACGAGAAAACAGACCGACCAATTACAGACACAGACACAAAAGAAATTATCATGGAAAGTAAAACTGCTCCTAATACGAGACGTACGCTTAGGAGCCTGATGAATTCTCTTTCTTTCTCAGAAACCCCTTCTCCTCCCGATATTAATGAGATTGATGTCCAACAAGAAATGAGATGGATTAAGGCCAAGTACCAATTAGAATTAAGCAAGCTCAGAGATCAACAGTTAAATCTTTCTTCAAAATCTTCAAGTTCTGAAGACAGACAACAAAAAATGGAAAATGCAACTCCTAGAGGCAACCACAACCAAATCCTTGACAGTTCTGGTCGAGACATGAACCGAAGTAGTACTGATAGCCATGTTTATATCAATAATAGCTGTTACAGTACAGACATGCCCAAGCAAAGGTCTCGAAATCGCAAGGCTGTGGAGTCCTCTATTGTGGATAAAGTTGTCACTGCTAAAAATGCCTGCAATGGTTCGTTGCTTCCAAGCTCCCTCCATAGAACAATCTCTCTCCCAGTTGATGCTGTTCATATATAG

>Csa7M234730.1

ATGAATGGTGCCACAGTAACAAATTCTGAACCAGACAACAACTCTGAATATGTTGAAGTTGATCCCACCGGAAGATATGGAAGATACAATGAAGTTCTTGGCAAAGGGGCATCGAAGACAGTTTACAGAGCTTTCGATGAATACGATGGCATTGAAGTGGCATGGAATCAAGTGAAGCTTAGTGATTTTCTTCAAACTCCTGAAGATCTTGAAAGGCTGTACAGAGAAATCCATTTGTTGAAGACATTAAAACATAACAACATAATGAAATTTTATTCTTCTTGGGTTGACATTGCTAATAGAAACATTAACTTTGTTACAGAGATGTTCACTTCTGGGACGCTTAGACAATATAGGTTGAAGCATAAGAAAGTGAATATCAGAGCTGTGAAGCATTGGTGTAGACAGATTTTAAAAGGGCTTCTCTATCTTCACAGCCATGATCCTCCTGTAATCCATAGAGACCTTAAATGTGATAATATTTTTGTAAATGGGAATCAAGGGGAAGTCAAAATTGGTGATCTTGGTCTTGCTGCTATTCTTCGTAAATCTTATGTAGCTCGATGTGTTGGAACACCAGAGTTTATGGCTCCAGAGATTTATGAAGAGGAGTATAATGAATTGGTTGATATATATTCTTTTGGAATGTGCATATTAGAGATGGTTACTTTTGAATATCCATACAGTGAATGCACCCACCCTGCTCAGATTTACAAGAAAGTTATCTCTGGGAAGAAACCAGATGCTTTATATAAGGTAAAGGATCTCGAGGTTCGGTGTTTCGTTGAGAAATGCTTGGCTACTGTGTCAACTAGGCTGTCAGCTAGGGAGCTTTTGAATGATCCTTTTCTTCAAATTGATGGTTGTGATTCTTTATTGAGGCCAATAGATTACTATTCAGAATATGATGAAGTGAACAATTCCCTTATCAGAGGAGGGCCTTTTTATGGAACTTCTCATGGTCCTTTGGATAATGGGTATGCGAATTATTTTAGTCATGAGGCTGGGAATGGCTTGGATTACTGTCCCATTGATAACGAGGCGAGCGAAATCGATCTTTTCTCCTGTCAAGAGGATGAGCATTTGGAAGATGTTGATATCACTATTAAAGGGAGAAGAAGAGACGACGATGATATCTTTCTACGACTTCGGATTGTAGATAAAGAAGGTCGAATTCGAAACATCTACTTTCCCTTTGATTTAGAGAACGATAGTGCTTCGAGTGTTGCTAATGAGATGGTTTCTGAGCTTGACATTACTGATCAAGATGTGAAGAAGATTGCTGATATGATAGATGGTGAGATTGCTACATTGGTACCAGAGTGGAAGAAGGGAAAAAGTTTAGAGGAAACTCCAAATTGCAGTGATTCTAATGTTTGTCACAATTGTTCTTTGAACAGTTCTCTTTTGGATTACGTTTCGCCACATAATCTTGCGAAGAAGAACCTGCACATTCTTCAGTGTTCTGAAGAGCATGGTTGTGCTTCTATCCATGGACGATTTGAAGAGATTACATACCAAGTTGAGGGGTCGGAACAATTTAATGGAGACGAAAACTTGCATAGAACAACAGGAAATTCAAGTGATATCCATTATGCCGATATTTGGGCGCAACGAGATGGACCCGATGTAGTTTCTCCAGAATCTTTAGAAGCTTGCAATGAATTTGGAGCGTCGGAACAGCCGAAACTCGAGAAGGAAGAAAGCAATGTAAATATGGATGATAATGATCATCAAATGGAATTCCAAACAAGAAACTCTAGCTCATCAAATCCTTCGGAGTCCTTTGTCGATGATCACGAGAACGAAATTCGACAAGAACTAAGATGGCTCAAGGCTAAATATCAAATGCAGTTAAGAGAGCTAAGAGATCAACAATTAGGAGTCAAGACCAAATCTCTTAGCTTGCACCCAATTTCCAACCTCACCGAGACCGATAACGGAGCTTCGGTATCTTATCTTTCGCCAAACTTCAATGAAGCAGCAAAAAATAAGACTGTCCAAACATCTCTCTCCTTTGGCAAGAATATCACTTCACATTCCCCCTATGTTGCTGCTGATAACATTTTGGAGAACAAAACCTTTCAAGACAACAATGTCATTGTTGACGAGTTGAGTAGTCCCGAGCTGATTGTTACGGCCAAGAGTTTCTATACAGGAGCATTGTTTCCACATTCTCTTCAAAGAGCAACCTCACTTCCCGTTGATGCTATAGACTTTTAG

>Csa1M695390.1

ATGGACACAACCGATTCAATCACTGCTTTCTCTCATCTTCTTCAACCTCCGGATTTCTCTCACTATGTTGAAATTGATCCCACTGGCAGATATGGAAGATATGATGAAATCCTCGGCAAAGGAGCTTCCAAGACAGTTTACAGAGCATTTGATGAGTATGAAGGGATTGAAGTTGCTTGGAATCAGGTCAAACTCTGTAACTTCCTTCAATGTCCTGAAGATCTTGAGAGGCTATACTCTGAGATTCATCTCTTAAAAACATTGAAACATAAGAACATTATGAAATTCTATACCTCTTGGGTCGATACTGCAAATAAGAACATCAACTTTGTAACTGAAATGTTCACTTCTGGGACTTTGAGGCAGTATAGGCTAAAGCATAGGAGAGCTAACATTAGAGCAGTGAAGCGATGGTGTAGACAAATTTTGAGGGGTCTTCACTATCTCCATAGCCAAGACCCCCCTGTGATCCACAGAGATCTCAAGTGTGACAACATTTTTGTTAATGGTAACCAGGGAGAAATTAAAATTGGAGACCTTGGTCTTGCTGCTATCCTCCGGAAATCACATGCTGATCATTGTGTTGGGACACCAGAGTTCATGGCTCCGGAAGTATATGAAGAGGCATATAATGAATTGGTAGACATTTATTCTTTTGGGATGTGTGTTTTGGAGATGGTTACGTTTGAATATCCATATAGTGAATGTAATCATCCTGTTCAAATCTATAAGAAAGTAATTTCTGGTAAAAAACCAGCTGCCTTGTATAAAGTGAAGGATCCAAGCATGCGACAATTCGTTGAGAAATGCTTGGCACCAGTCTCTTGTAGACTTTCAGCAAGGGAGCTTTTGAGTGACCCTTTTCTCGAAATTGATGGTTGTGAAAGCAAGTTGAAAATTTCAGATTCTCGGAGAGAACTGGACGATTTTGCTTCCACTATAGTACGCCCTTTCCTTGAACGAGAAAAAAGATTTAGCTCAATTAGTTACAGTTTGGAGGGTTCAGATGAATGGAGGTACCGTTCGGTCCAGAAGGAACCTGATGGGATTGAACTTTTTGAGGACAATGATAATGACCAATTGGTAAGTCTTGATAACAATATTAAGGGGAAAATACGTGAAGATGGCAGCATTGTTTTAAGACTTAGGATTACGGATAAAGAAGGGCTTATTCGGAACATATATTTCCCATTTGACACCAAGAATGATACAGCATTAACAGTAGCCACCGAAATGATAGCGGAGCTTGACATTACTGACCAAGATGTGATCAAGATAGCTGAAAAAATTGATGGGGAAATTTCTTCCTTGGTTCCCGAGTGGAAGCCAGGGCCTGGCATAGACGAAACACCTCGCATCTCTTACGACGGTGGCTCTCAAAGCTACAACGCTTGCAATCAACCATCTGATAATATTTTGATAGAGAATAAAGGAAATGGAATCAAACTCTATCAGATTTTAAACTTAAGCACAGATGGGCATGCTTTGGCACACGAACATTTCGAGCAAGAGCAGTTCTCGCTCAAAGCAGATCGACCTACACAACCAAATGTGTCAAGCCAGCACTATCAGCCAGATTCTGTCCTCAACGAAAACCAAGCTCTTAGTTCTCATAGTTTTAGGCAAAGACATTCTGATGATAATTACAAGAAAATTGACCAATCACTCACTGTTGGGTATAACAAAGAAAAACTACCAGTTAACAAAGCCACTGTCATTGACACTTCACAAAGAAGCTTATTGGGTTCCCGATCACTGTCCACGGTTTCATCCTATTGCGAAGATAAATTTTCCTCTCAAATTCATTGGGAAATTAGATGGCTGTGGAATTGA

>Csa3M119370.1

ATGGAAACAGGGCCTGCCAGTTCTAAGGATTCATCGCCATTGCCCTTTGATATGGAATGCCGATTTCGCTTGCGATCGGCATTCGATTCTTTTTGCATTGCCTCTGAAGCCTTTTCAGAGCATCGTCGTTGTCGAATTCCAATCTGGCGACATGTTTTTTGTAGCAAAAATGAGTGCCTAGCTGATCCGATTAGTGCGTTATTTGGAAGTATGGAGTCTCCTGATAATGCTGCTGAAAAAGATCCTACCGGTCGATACGTTCGGTACGATGAAATCCTGGGAAGGGGCGCTTTCAAGACAGTTTACAAGGCATTCGACGAAGTGGATGGAATTGAAGTTGCCTGGAATCAAGTTAGGATTGATGGTTTCTTGCAATCACCTGAGGATTTGGAAAAGTTGTACTCTGAAGTACATCTACTTAAATCATTAAAACATGAGAATATTATCAAGTTCTATAATTCTTGGGTGGATGATAAGAAGAAAACAGTCAACATGATCACTGAGCTCTTCACATCTGGGAGTTTGAGGCAATATCGTAAAAAGCATAAACATGTTGATATGAAGGCCATAAAGAATTGGGCGCGACAGATTCTCCGAGGGTTGGTTTATCTTCACAGTCATGATCCTCCCATTATTCATAGAGATTTGAAAGGCGATAACATTTTTATTAATGGAAATCATGGAGAAGTTAAAATTGGAGATCTTGGATTAGCAATTGTTATGCAGCAGCCTACCGCTCGTAGTGTAATTGGAACTCCTGAGTTCATGGCTCCTGAGCTTTATGAAGAGGAGTACAATGAGCTTGTTGATGTATATTCTTTTGGGATGTGCATGCTGGAGATGGTGACCTTTGAGTATCCATATAGTGAATGTAAAAATCCAGCTCAGATCTTTAGGAAAGTTACATCTGGTATTAAACCTGCTTCCCTTGCTAAGGTGAGTGATCCACGGACCATGGAATTCATCAATAAATGTTTGGTTCCAGTGCACGAGAGGTTGTCTGCAAAAGAGCTTCTTAAAGACTCGTTCCTTCAAGTTGAGAACCCAAAGGAATCGGCACGCAATCCCCTACAGCTATCTAATCAGGTTTCTAAATCAATAAATTTGCCCAAGAGTGGACCTATTTCCATGGACATTGACATTGACCAAAAAATACATTCTTTAAGCACTTATGCTGAAAGCAATAGCGGGAGTCCACGCTTTCCAGTCGTGGAATTCCAGACAATGAATAAGAACAATGAGTTCAGGTTACGTGGAAATAAGAATGACGATAACTCTGTGGCATTAACTCTGCGTATTGCAGACTCAAATGGTCGAGTGAGGAATATACATTTTACGTTTTATCTCGATTCTGATACTGCATTGTCAGTTGCTGCTGAAATGGCTGAACAGTTGGAGTTGATAAATCATGATGTGGATTTCATAGCTGAATTTATTGATTTTTTGATAACAAAGCTTATACCTGAATGGAAGCCCTTGTCTGTTTATTCCTCAAATGGAGAATTGAGTCTTTTCAGCGCCCCCCCTTTCCTTAAAAGTGCTAAATCATCTATAGGATCTGCTTGGGGTTCAATCTTAACTGGGAGTCATGATGGATTGGTCGCACAAGACATTTCTTCTGGGTTGGGGTGTGGTACTCAAAAGGATTGTCTGCAATCTGAAGAAGATGGCTGGACTACTGATATTTCCGCTGGTCACATTTTTGATACTTGCCCTTCTTCACCTAGTTTGGCTAATTTTGAGGACTTGAATTCACATGCTTCATTTGCGTTGGAGTTACTGGTCGACGATTGCTCTACCAAAAGTGCTAAGGTGTTTGATTGTTCCAATATCGATGGAAGTTCAAAAGGCTCGAGCTGGTCGATTGCAGAACTAGAGCACCACGGTTCATCATATGTTGTTGAAGACAAATTTCAAAGAAATGTTGGTGATGTTGGAATCTTCACTCCCATGGATTATTTTGCGAAGAACTCGGTGGTGTCAATGCCTGCTCCAAGTGAAGCATCTAATGTGATGAGCTTGACAAGCAGTTGCTCTTCGTTATCGTTAACAGACAAGGATCTCGATGCTGAACTGAAGATGGAAATCGATGCTATCGAGACACATTATCGGCAACTGTTTGATGAGCTTTCTCGAATGAGAGAGGAGGCACTAGAGGCCACCAGAAGGAGATGGATTGCAAAGAAGAAACTGATTCATTGA

>Csa5M148620.1

ATGTGGCTTCTGAGGCCTTTTCTGAACACCTTTCTCCTTTGGTCGTCCTTATCTGACTCTTTGCTCTTTCCAGTAACTGGGTTTTCTTCTCTCTTTAACTTCTTTCTTTTTCTCTTTCGTCTGTCTTTCTCCGTTACGTTGAGTTGCGGTTCGGATTTGATCATGTCTGGGAATTCTGAGGCTGATGATCGTGGGATTGTCGAGAAGGATCCATCTGGACGATATATACGGTATGATGAGATATTGGGTAAAGGGGCATTCAAGACAGTTTACAAAGCATTCGATGAAGTTGGTGGAATCGAAGTTGCTTGGAGTCAGGTGGACATTGAAGATGTCTTGCAGTCCCCAGAGCAACTCCAAAGATTATATTCTGAGGTCCATCTGTTGAAGTCACTGAAACATGAAAATATCATCAAGTTCTATAGCTATTGGGTTGATGATAAACATAAGACTATCAACATGATTACAGAATTGTTCACTTCTGGGAGTTTGAGGCAATATCGAAAGAAGCATAGGAAAGTTGACTTGAAGGCCTTCAAGAACTGGGCAAGGCAGATACTGCGAGGTTTAACGTATTTGCATGGTCACAACCCTCCAATCATTCACAGGGATTTGAAATGTGATAATATATTTGTCAATGGAAATACTGGAGAAGTTAAAATTGGAGATCTCGGATTGGCAATTGTCATGCAGCAGCCTACTGCTCGAAGTGTAATTGGCACTCCAGAGTTCATGGCTCCAGAACTTTATGATGAGGACTATAATGAACTTGTTGACATATATTCTTTTGGCATGTGCATGTTGGAAATTGTTACTTGTGAATACCCATATAATGAGTGCAAAAATTCTGCCCAAATATTCAAGAAGGTTACTTCAGGTATAAAGCCGGCTTCCCTTGAGAAAGTACTGGATCCTCAAGTTAAGCAATTTATTGAGAAGTGCCTGGTTCCTGCATCCACGAGATTACCTGCTAGTGAACTCTTGAAAGATCCTTTCCTTGCGGCTGAAAGTCCTAAGGACAATAGCTCTGAACTTTCAAGGTCATTGAATGAGCATTTCAAATCTGTGAATCCTCCACTGCTTGGTTCTCATCCAATGGAAACAGACCATAACTGCACCAAGCTCTCAGGTTCAGTGGCCTCATCTGTCAAAAGCAATAATGGAATTTCTCACTTTTCAACTCAAGAACTTCAGAGGCTCACTGAAAATAATGAATTGACACTGAAAGGAGACATGACTGATCATAATACGATGTCATTTCATTTGCGTATTGCTGAACTATATGGTAAATCTAGGAATATTCATTTTGCCTTCTATCTCGACTCTGATACTTCTTTGGCAATTGCTTTGGAGATGGTTGAACAACTTGAACTGTCGAATGAAGATGCGACTATCATAGCAAAGCTAATTGATGAGTTAATTGCTAAGTTTGTTCCTAGCTGGAAACCATGTCCAAACTATTGTGAAGAACAACAACAAAATACTCCTCATAGTCCTGAAGCTCAAGAGGATAAGACCTTTATTTCTCCTTTCTTCTCTGAGCTGGTACTGTCATCTCCCATGGTGGCTGCTGCAAGGAATAATCTGACAGGTTTGGCAAAGGTCGAAGATCAAGAAAACCAGCAGTCGATTATTTCGTGTGCTTCCGTGGAATACATTTACTCAACTGTCTCAGACTACAGCATAGGAAAAGGATCAGAGTGTGGTGAGTTTGGTCACCCTGATTGTGAAAAAGCTTATATTAGCAGTGGAACAATTGATTTAGATGCAGAAGCTGTTGGATCTCTTTCGACGACAATTGATTTTGCAAAACCGTCACTAATCTCTTCCTGCTCAGAAATGTCGAAAGAATTGAGTTTGTCTAGCTTTTCAACTCTTTCTATGGAAGAAAGAGATCACCAAGATGAACTGAAGATGGAGATTGATGCTATTGATTTGCAGTATCATCAGTGCCTTTGTGAACTGTCAAGAATGAGAGAGGAAGCAATAGAAAGTGCTAAGAAGAGGTGGATGTCTAAGAAGAAAGCTACGGGTATTTGA

>Csa3M062560.1

ATGATGCCTAGTGTAATGACAGAGTCATCAGATAAAGAGACAGAGGCATTTGTAGAGGTGGATCCAACGTGCCGATATGGGCGCTATCCCGAGCTTCTGGGAACAGGGGCGGTAAAGAAAGTGTACAGAGCATTTGATCAAGAAGAAGGAATAGAGGTAGCTTGGAACCAAGTGAAATTAAGGAGTTTTTCAAATGATCCATCCATGATAGATAGGTTGTATTCAGAGGTGAGACTGTTAAGAAGCTTAAAGAACAATAACATAATAGCCTTGTATGATGTTTGGCTTGACAAACTTCATGGAACCTTAAATTTCATTACTGAGGTTTGTACAAGTGGGAACCTTAGAGAGTACCGGAAGAAACATAGACAAGTCTCTTTGAAAGCTTTGAAGAAATGGTCCAAACAAATTCTCAAGGGTTTGCATTATTTGCATTCTAATGACCCTTGTGTAATTCATAGAGATCTCAATTGTAGCAACCTCTTTGTCAATGGCAATGTTGGTCAGGTAAAGATTGGTGACTTGGGTTTGGCAGCAACAGTAAGAAAGAATCACTCAGCTCATTCAGTGCTTGGAACACCAGAATTCATGGCACCTGAGTTATATGAAGAGCATTACACCGAGCTTGTGGATATATATTCATTTGGAATGTGTTTGCTTGAATTAGTGACTCTGGAAATTCCCTATAGTGAATGTGACAATGTTGCCAAAATCTACAAGAAAGTCTCCTCAGGCATCAAGCCTCAAGCCCTTGGCAAGGTCAAGGACCCTGAAGTCAAGGCTTTCATTGAGAATTGCCTTGCCGAATCCAAAGTCCGGCCTTCAGCCGCCGACCTCCTCCGCCACCCCTTTTTCAGAGAAATTGATGATGACGAAAACGAAGACGACAATAACGATCATTAG

>Csa6M110320.1

ATGGATTACGCGCCACGTATAAAATCAGAATCCTCCTGCCGCCTCGACCTGACGCAAGAGAAAAACCAACTAAACGGAACACAAATAGAGATACAAGTTTTTGTTTCTCGCTCCGTTTCTTCGGAGATTTTCCCTCTCCGGCATCAACCCCAAGGTATTCTCTCCTCCTCTTCTTCTTCTTCTTCTTCTTCTTCTTCAAATTATACTGCTTCTCATGTTAATGGATGTTTGATGTTTGATATGCCAGCTGAAAACTCAATTCCATATGATCGTGATGCAGAGCCCTTTGTTGAGGTCGATCCAACTGGAAGGTTTGGACGATACGATGATCTTCTTGGGTCTGGAGCCGTAAAGAAAGTTTATAGGGCATTTGATCAGGAGGAAGGAATAGAGGTTGCTTGGAATCAAGTTCGATTGAGAAATTTTAGTGGGGATCCAGTGTTCATCAACCGTCTGAGGTCTGAGGTTCAGCTGCTGAGCACTTTGAACAACAAATATATCATCGTTTGTTACAGTGTTTGGAATGATGATGAGCATAATACATTGAATTTTATCACCGAGGTGTGCACATCAGGAAATCTAAGGGATTATCGTAAGAAACATCGACATGTGTCAATTAAGGCCTTGAAAAAATGGTCAAAGCAAGTGCTCGAGGGATTGGATTATCTTCATACTCATGAGCCTTGCATTATTCACAGAGACCTCAATTGCAGTAACATTTTTGTCAATGGGAATATTGGCCAGGTTAAAATAGGCGATCTAGGGTTTGCAGCTATAGTTGGCAGGAGCCATGCTGCACATTCAATCATAGGAACACCCGAGTATATGGCACCAGAGCTGTATGAGGAAGACTACACAGAGATGGTGGATATATACTCCTTTGCAATGTGTTTGCTCGAGATGGTTACTATGGAGATACCATACAGTGAATGCGATAGTGTTGCCAAGATATACAAGAAGGTAACAACTGGAATTAAGCCCCAAGCAATTACCAAAGTAACTGATGCTGAAGTCCGAGCTTTCATTGAGAAGTGCATTGCACAGCCAAGAGCAAGACCATCCGCTTCTGAACTTCTCAAGGATCCCTTCTTCGACGAAGTCAGGGATGAAGATTCTGAACAGACTTCTTAA

>Csa1M046910.1

ATGTCTCAGGATTTGTCACCAGATCAAGACCTGGATGAATCTGATCCCGAGTTTGTTGAGATTGATCCTACTGGTCGATATGGAAGGTATAAGGAAATTTTGGGCAAAGGGGCATTCAAAAGGGTATATCGAGCTTTTGATGAGTTGGAAGGAATCGAAGTAGCTTGGAATCAGGTTAAGGTGACAGATCTATTGCGAAACTCTGAAGATTTGGAGCGGCTTTATTCAGAAGTTCATTTGCTTAAGACATTGAAGCACAAGAACATAATCAAATTTTACAATTCATGGGTTGATACAAAAAATGAAAACATTAACTTCATTACTGAGATATTCACCTCTGGAACACTAAGACAATATAGAAAGAAACACAAGCATGTTGATGTGAGAGCATTGAAAAAGTGGTCCAGGCAGATATTAGAGGGCCTTCTCTATCTTCATAGTCACGACCCTCCAGTTATACATAGGGATTTAAAATGTGACAATATCTTTGTAAATGGCAACCAAGGTGAGGTGAAAATTGGTGACTTGGGACTTGCTGCCATTCTTCAACAAGCTCGTTCAGCCCACAGTGTCATTGGGACACCTGAATTTATGGCTCCGGAGTTGTATGAGGAGGAATACAATGAACTTGTGGATATTTATGCCTTTGGCATGTGTTTGCTTGAGCTTGTAACATTTGAATATCCATATATTGAATGTGCAAATGCTGCTCAAATCTATAAGAAGGTCACATCAGGGATTAAACCAGCTTCGTTGGCAAAAGTCACTAACTTAGGAGTGAGAGCATTTATAGAAAAATGCATCGCAAATGTCTCTGATCGCTTACCAGCAAAAGATCTTTTAAGGGATCCATTTCTCCAAGCAGATGATGATCATGAAAGTATATCTCGTCATTTACGATCAAAGACTCAACCTACAGAAAAAAAAGAGCAAATTGATTTCGATCGAAGTGTAGATTATTCTCCGGCTGAGACTAGTAGAGATTTCAGTATGCATGGCGAGAGAAAAGATGTTAATAAGATATTTCTCAAACTACGAATTGCAGATTCTATGGGTAATTTTCGCAATATCCATTTCCCGTTTGATATTGAAGCCGACACAGCAATTTCTGTTGCCAGCGAAATGGTCGAGGAGTTGGATCTTTCTGATCAAGATGTCTCCACAATTTCTGAAATGATTGAAACAGAAATTCGATCATACATTCCAGATTGGATATCGGTTGAGTATTCTGGAGATAATGTTGGAGCTGATGCTCCAGTTTCTGATAGCTCTCCATCTGAAACGAGAAACGTTGCATCTCCGTTGTCTATTGAATCTGGTAACCTTGCATTAGAGGTAATGCCTTCAGGCCGCAAGTATTGGTCAGACTCTCCAAAAGGAATTGGTGGATGTTCTCCTATTAAGCCAGGTCCTTCAAACTTGTCTTTTGCCTCAGATCAGAATGTTGAATCCTCTAACAGCCACATACATGGGGACAATCTAGATCATGCTGCTATAATCAAGGGACTGGAAAATGAGCTTTTGTCGGAGGGTGGTGATCATGATGGGCAAGATGAGAGTAGCATTCATACTAGCTCTGAGACACATCATTCGGAAGAGAACAATTATGATGAATCAGTTGATCTCAAAATAGTAGCTGAGAAACTTGAAAATTTGTTGACACAACAACAAAAAGAATTAGATGAACTAAGGAAAAAACATAAATTGGATATTTCAGAACTTTTGACTAAACTCACACCTGAATCCTATCAGAAAGTTATAGAGATGTGTCAGCTACAGCATCCTGATTTTGAGTTGGTTCTCTAA

**B:** **The protein sequences of CsMAPK cascade genes**

>Csa2M361890.1

MGALSISFMDMFTKSTRYFSIDVSVNKMSAQENGTHLESEIKEVEQEVVEEGNNPNPQIIDYITYPNCKFTEGGIKSCARELPHLVAKKFPLPHSQFTRFHAFQLPIRAGVLLILGFFFLSPSGVSLFLLILSWNLLISTFFKVLEANFELDQCSLSVLMLYLTSVWLLLSLVGMATPVEPPNGVRSQGKHYYSMWQTLFEIDTKYVPIKPIGRGAYGIVCSSVNRETNEKVAIKRIHNAFENRIDALRTLRELKLLRHLRHENVICLKDVMMPIHRRSFKDVYLVYELMDTDLHQIIKSSQTLTNDHCQYFLFQLLRGLKYLHSANILHRDLKPGNLLVNANCDLKICDFGLARTSNGKNQFMTEYVVTRWYRAPELLLCCENYGTSIDVWSVGCIFAELLGRKPIFPGTECLNQLKLIINLLGSQREEDLEFIDNPKARRYIKSLPYSPGAPLSRLYPSAHPLAIDLLQKMLVFDPSKRISVTEALQHPYMSPLYDPNSNPPAQVPIDLEIDEELGEEMIREMMWKEMLHYHPEDLEEHAEMTRFHPEPTTSSAAVYS

>Csa1M479630.1

MADVGQNNPADFPALPTHGGQYVQYNIFGNPFEITSKYRPPIMPIGRGAYGIVCSVLNSETNEMVAVKKIANAFDNHMDAKRTLREIKLLRHLDHENVIGIRDVIPPPLRREFNDVYISTELMDTDLHQIIRSNQSLSEEHCQYFLYQILRGLKYIHSANVIHRDLKPSNLLLNANCDLKICDFGLARPTSENECMTEYVVTRWYRAPELLLNSDYTAAIDIWSVGCIFLELMNRRPLFPGRDHVHQMRLLTELLGTPSESDLGFIRNEDSKRYLRQLPPHPRQPLATVFPHVHPLAIDLVDKMLTFDPTKRITVEEALAHPYLERLHDIADEPVCSEPFSFEFEQQYLDEEQMKEMIYREALALNPEFA

>Csa5M152810.1

MATKESSSTTATEGKIKGVLTHGGRYVQYNVYGNLFEVSAKYVPPLRPIGRGAYGLVCAAVNSETHEEVAIKKIGNAFDNIIDAKRTLREIKLLCHMEHENIIAIRDIIRPPKREVFNDVYIVYELMDTDLHQIIRSDQPLTDDHCQYFLYQLLRGLKYVHSAKVLHRDLKPSNLLLNANCDLKIGDFGLARTTSETDFMTEYVVTRWYRAPELLLNCSEYTAAIDVWSVGCILGEIMTREPLFPGKDYVHQLRLITELLGSPDDASLGFLRSDNARRYVKQLPQYRKQQFSARFPNMSPSALDLLEKMLVFDPNKRITVEEALCHPYLQSLHDINDEPVCARPFNFDFEQPSCTEEHIKELIWKESVRFNPDESARRTTLCV

>Csa6M006730.1

MENSSFYQNIRGEPTHGGQYIQYNVYGNLFEVSRKYTPPIRPVGRGAYGIVCAALNSETNEDVAIKKVGKAFDNRIDAKRTLREIKLLRHMDHENIIALRDIIRPPQKENFNDVYLVYELMDTDLNQIIRSNQSLTDDHCRYFLYQLLRGLKYVHSANVLHRDLKPSNLFLNANCDLKIGDFGLARTTSETDFMTEYVVTRWYRAPELLLNCSEYTGAIDIWSVGCILGEIMHRKPLFPGKDYVHQLKLITELIGSPDESSLGFLRSDNPRRYFRHLPHFPKQQFSSKFPTMSPAAIDLLEKMLVFDPTKRITVDEALCHPYLAPLHDINEEPVCPRPFSFDFEQPTYTEENIKELVWRESLRFNPGPAF

>Csa6M365750.1

MDDGGASQPDDTVMSEAASVPPPQHDPAAQQQHQHQPPSMGMENIPATLSHGGRFIQYNIFGNIFEVTAKYKPPIMPIGKGAYGIVCSALNSETNEHVAIKKIANAFDNKIDAKRTLREIKLLRHMDHENVVAIRDIIPPPLRETFNDVYIAYELMDTDLHQIIRSNQALSEEHCQYFLYQILRGLKYIHSANVLHRDLKPSNLLLNANCDLKICDFGLARVTSETDFMTEYVVTRWYRAPELLLNSSDYTAAIDVWSVGCIFMELMDRKPLFPGRDHVHQLRLLLELIGTPSEADLGFLNENAKRYIRQLPHYHRQSFTEKFPHVHPAAIDLVEKMLTFDPGQRITVEDALAHPYLTSLHDISDEPVCMTPFSFDFEQHALTEEQMKELIYLEALAFNPEYHHQ

>Csa4M045070.1

MATFVEPPSGIRSMGKHYYTMWQTLFEVDTKYVPIKPIGRGAYGVVCSSINRETNEKVAIKKIHNVFENRTDAMRTLRELKLLRHIRHENVIALKDVMMPIHRKSFKDVYLVYELMDTDLHQIIKSPQPLSHDHCKYFIYQLLCGLQHLHSANILHRDLKPGNLLVNANCDLKICDFGLARTSMGRDQFMTEYVVTRWYRAPELLLCCDNYGTSIDVWSVGCIFAEILGRQPIFPGTECLNQLNLIITILGSPKEADVEFIDNVKARNYIKSMPFSRGIRLSHLYPQAEPLAIDLLQKMLVFDPTKRITVDEALQHPYMSGLYDPKFNSSVEVPLNLDIDDTLGEPKIREMMLNEMLYYHPEAVSTFS

>Csa1M024990.1

MGSGTLVDGVRRWFQRRTSSSSSSTSNSNSNFSSNSDSSDPNLNYPNLHKFDYVDNGGVSGDQLLSSDLRAQSSIAHKRKPLRKQTQLGEGGILEQLPEEEDDDLDYSALKLIKVPKRINHFRNPPPPLPSALMDSHKKGGLETEFFTEYGEASRYQVQEIIGKGSYGVVGSAVDTHTDEKVAIKKINDVFEHVSDATRILREIKLLRLLRHPDIVEIKHIMLPPSRREFRDIYVVFELMESDLHQVIKANDDLTPEHHQFFLYQLLRGLKYIHTANVFHRDLKPKNILANADCKLKICDFGLARVSFNDAPSAIFWTDYVATRWYRAPELCGSFFSKYTPAIDIWSIGCIFAEMLTGKPLFPGKNVVHQLDLMTDVLGTPSSESIARIRNEKARRYLSNMRRKQPVPLTQKFPNADPLALRLLQRLLAFDPKDRPTAEEALADPYFQGLANVDREPSTQPISKLEFEFERRKLTKDDVRELIYREILEYHPQMLQEYLRSGEQTSFMYPSGVDRFKRQFAHLEEHYGKGERSTPLQRQHASLPRERIPAPKDEAGQHNDLEGRNVATSLQSPPKSQGDGSENANGNEQNGQNKPNYSARSLLKSASISASKCIGVKPRKDLEEEPISETNDEAVDGVSHKMSALHT

>Csa5M002030.1

MIEKEFFTEYGEATQYEIEEVVGKGSYGVVASAIDTHSGEKVAIKKINNVFEHVSDATRILREIKLLRFLRHPDIVDIKHIMLPPSRREFKDLYIVFELMECDLHHVLKTNDDLTPQHHQFFLYQLLRALKYIHSAHVFHRDLKPKNILANADCKLKICDFGLARASFSDAPSAIFWTDYVATRWYRAPELCGSFFSKYTPAIDIWSIGCIFAEMLGSKPLFPGKSVVHELDLITDLLGTPSAECIAKIRNEKAKRYLSGMRKKDPIPLSKKFPNADPLALRLLERLLAFDPDDRPSAEEALADPYFHGLANLKDEPSRQPISKLEFEFEKRKLTKDDVRELIYREILEYHPQMLKEYLQGSGSHFLYPSGIDRFKRQFDHLEERSGKGERGSPLLRKHASLPRERIYTLGYEDDDDEKHRTGYRNAASIERAAVHSPPAYPLTARNDCNSYNLLRSASISCSKWVD

>Csa5M002030.2

MIEKEFFTEYGEATQYEIEEVVGKGSYGVVASAIDTHSGEKVAIKKINNVFEHVSDATRILREIKLLRFLRHPDIVDIKHIMLPPSRREFKDLYIVFELMECDLHHVLKTNDDLTPQHHQFFLYQLLRALKYIHSAHVFHRDLKPKNILANADCKLKICDFGLARASFSDAPSAIFWTDYVATRWYRAPELCGSFFSKYTPAIDIWSIGCIFAEMLGSKPLFPGKSVVHELDLITDLLGTPSAECIAKIRNEKAKRYLSGMRKKDPIPLSKKFPNADPLALRLLERLLAFDPDDRPSAEEALADPYFHGLANLKDEPSRQPISKLEFEFEKRKLTKDDVRELIYREILEYHPQMLKEYLQGSGSHFLYPSGIDRFKRQFDHLEERSGKGERGSPLLRKHASLPRERIYTLGYEDDDDEKHRTGYRNAASIERAAVHSPPAYPLTARNDCNSYNLLRSASISCSKWVD

>Csa5M002030.3

MIEKEFFTEYGEATQYEIEEVVGKGSYGVVASAIDTHSAHVFHRDLKPKNILANADCKLKICDFGLARASFSDAPSAIFWTDYVATRWYRAPELCGSFFSKYTPAIDIWSIGCIFAEMLGSKPLFPGKSVVHELDLITDLLGTPSAECIAKIRNEKAKRYLSGMRKKDPIPLSKKFPNADPLALRLLERLLAFDPDDRPSAEEALADPYFHGLANLKDEPSRQPISKLEFEFEKRKLTKDDVRELIYREILEYHPQMLKEYLQGSGSHFLYPSGIDRFKRQFDHLEERSGKGERGSPLLRKHASLPRERIYTLGYEDDDDEKHRTGYRNAASIERAAVHSPPAYPLTARNDCNSYNLLRSASISCSKWVD

>Csa1M042720.1

MGDKQSEFFTEYGEASRYQIQEVIGKGSYGIVGSAIDTQTGERVAIKKINDVFEHVSDAIRILREIKLLRMLHHPNIVEIKHIMLPPSQREFKDIYLVFELMKSDLHHVIKTNNDLSPRQHKFFLYQLLSGLKYIHTANVLHRDLKPKNILANADCRLKICDFGLARVSFSDAPSTIFWTDYVATRWYRAPELCGSFFSRYTPAIDIWSIGCIFAEMLTGKPLFPGKNVVHQLDLITDLFGSPEPEAIAKIRNEKARRYLGNMRKKQPVPFSRKFPNVDPMALCLLERLLAFDPKCRLTAAEALADPYFNGMGKPELEPSIQPISKLEFEFERRKLSKDDVRELIYAEILEYHPQMRQGCLRGGDHPTTFMYPSGVDRFKLQFAHLEEHHGKGERRSPLQRQNISLPRERVRPTEQNNTENSIDSERGKDKSAHLLKSASISASRCVGVIPKETYEVEETEVKNEAVDGMSQKIAVLQT

>Csa1M042720.2

MGDKQSEFFTEYGEASRYQIQEVIGKGSYGIVGSAIDTQTGERVAIKKINDVFEHVSDAIRILREIKLLRMLHHPNIVEIKHIMLPPSQREFKDIYLVFELMKSDLHHVIKTNNDLSPRQHKFFLYQLLSGLKYIHTANVLHRDLKPKNILANADCRLKICDFGLARVSFSDAPSTIFWTDYVATRWYRAPELCGSFFSRYTPAIDIWSIGCIFAEMLTGKPLFPGKNVVHQLDLITDLFGSPEPEAIAKIRNEKARRYLGNMRKKQPVPFSRKFPNVDPMALCLLERLLAFDPKCRLTAAEALADPYFNGMGKPELEPSIQPISKLEFEFERRKLSKDDVRELIYAEILEYHPQMRQGCLRGGDHPTTFMYPSGVDRFKLQFAHLEEHHGKGERRSPLQRQNISLPRERVRPTEQNNTENSIDSERGKDKSAHLLKSASISASRCVGVIPKETYEWRLPIGSSNFKTDTRDMCDFFPSRTCIRVISKPF

>Csa1M077220.1

MENDSSSAMDIKGTPTYDSKYLLYNVLGSFFEVSAKYSPSIQPVGRGAYGIVCCTTNSETKEEVAIKKIGNAFDNRIDAKRTLREIKLLCHMDHDNIIKIKDIIPPPDKEKFNDVYIVYELMDTDLHQIIRSSQALTDDHCQYFLYQLLRGLKYLHSANVLHRDLKPSNLLLNANCDLKICDFGLARTTSETDFMTEYVVTRWYRAPELLLNTSEYTAAIDIWSVGCILMEILRREPLFPGKDYVQQLGLITELLGSPDDSDLGFLRSDNARKYVKQLPHFPKQPLIEKFPDLPPLAVDLAERMLLFDPSKRITVEEAMNHPYIVSLHEINEEPTCPSPFNFDFEQASLDEEDIKELIWRESIKFNPNHI

>Csa6M061230.1

MQPDQRRKSSIDVDFFTEYGEGSRYRIEEVIGKGSYGVVCSAYDTHTGDKVAIKKINDIFEHVSDATRILREIKLLRLLRHPDIVEIKHILLPPSRREFKDIYVVFELMESDLHQVIKANDDLTPEHYQFFLYQLLRGLKYIHTANVFHRDLKPKNILANADCKLKICDFGLARVAFNDTPTAIFWTDYVATRWYRAPELCGSFFSKYTPAIDIWSIGCIFAELLTGKPLFPGKNVVHQLDLMTDFLGTPNAEAIARVRNEKARRYLSSMRKKKPVPFSQKFPHADPLALRLLERMLAFEPKDRPTAEEALADPYFKGLAKVEREPSAQPVTKMEFEFERRRITKEDVRELIYREILEYHPKMLKEFLDGSEPTGFMYPSAVDHFKKQFAFLEEHYGNGAPVAPPERQHASLPRPCVLYSDNMVQNPAQVANDLSKCSIKEVERPPVDRTCNIPLARVPIQVPQSIQAGNGARPGKVVGSVLRYNCGAAAAAVAPEVLEQRRMTRNPSIPPQYAGNNCSYTRRNSSCKNERADEEAIEGPNGLQPKPQYIARKVAAAQGGPGNNWY

>Csa4M082320.1

MPQDHPKKEAKEVNFFTEYGDANRYKILEVVGKGSYGVVCSAIDMQTAEKVAIKRIHDIFDHASDAIRILREVKLLRLLRHPDIVDIKRIMLPPSKKEFRDIYVVFELMESDLHQVIKANDDLTREHHQFFLYQMLRALKFMHTANVYHRDLKPKNILANANCKLKICDFGLARVAFSDTPTTVFWTDYVATRWYRAPELCGSFCSKYTPAIDIWSVGCIFAEVLMGKPLFPGKSVAHQLDLITDLLGTPSMETIAGVRNEKVRKYLTEMKKKSPVPFSQRFPKVDPTAIRLLERLLAFNPKDRPSAVEALADPYFKGLAKVEREPSCQPISRSEFEFERRKLTKDDVRELLYREILEYHPQIRDDYLNGTETTKLHYPSVTGHFKSQFTFHKENNGKSAPVLPLERKHFSLPRSTVCTNLVSPDHEPVRRNPKVCNNSMGLPDRTFGNPSKAHHPPKVPTGRVAGSILPYEHRNIKDVYSKLTSQIRSLDF

>Csa4M082320.2

MPQDHPKKEAKEVNFFTEYGDANRYKILEVVGKGSYGVVCSAIDMQTAEKVAIKRIHDIFDHASDAIRILREVKLLRLLRHPDIVDIKRIMLPPSKKEFRDIYVVFELMESDLHQVIKANDDLTREHHQFFLYQMLRALKFMHTANVYHRDLKPKNILANANCKLKICDFGLARVAFSDTPTTVFWTDYVATRWYRAPELCGSFCSKYTPAIDIWSVGCIFAEVLMGKPLFPGKSVAHQLDLITDLLGTPSMETIAGVRNEKVRKYLTEMKKKSPVPFSQRFPKVDPTAIRLLERLLAFNPKDRPSAVEALADPYFKGLAKVEREPSCQPISRSEFEFERRKLTKDDVRELLYREILEYHPQIRDDYLNGTETTKLHYPSVTGHFKSQFTFHKENNGKSAPVLPLERKHFSLPRSTVCTNLVSPDHEPVRRNPKVCNNSMGLPDRTFGNPSKAHHPPKVPTGMNIFLMVFPSKFLSFLFNGQILSRVSFERESCKLV

>Csa6M179480.1

MQQDQSKKNSTEVEFFSDYGDASRYKIQEVIGKGSYGVVCSAIDTRTGDKVAIKKIHNIFEHISDAVRILREIKLLRLLRHPDIVEIKHIMLPPSRRDFKDIYVVFELMESDLHQVIKANDDLTREHYQFFLYQLLRALKYIHTANVYHRDLKPKNILANANCKLKICDFGLARVAFSDTPTTIFWTDYVATRWYRAPELCGSFFSKYTPAIDIWSIGCIFAEVLMGKPLFPGKNVVHQLDLMTDLLGTPSLDTISRVRNDKARRYLTTMRKKQPVPFSQKFPNADPLALRLLERLLAFDPKDRPTAEEALADPYFKGLAKIEREPSCQPITKMEFEFERRRVTKEDIQELIFREILEYHPQLLKDYMNGTERTNFLYPSAVDQFRKQFAHLEENGGKSAPVIPLDRKHVSLPRSTVVHSNPVYSKDQVNNIPLQDGKISEDAYSKNSRDSEGRLTNISRTMQAPQKIPFAAKPGRVVGPVIADENGRLVKEPYDPRTLIRGAILPPAYHYHQKPIVGNQERSAAETKLDISLRAAKQASQCGMASKLGSDIAISIDSNPFYMTRAGVNKVELKDQISINANFLQAKAAQYGGLSAATATTTSVAHRKVVAGQFNMTKMY

>Csa6M423420.1

MQTDHRKKNSAELDFFSEYGDANRFKVREVIGKGSYGVVCSAVDTLTNEKVAIKKIHDIFEHVSDAARILREIKLLRLLRHPDIVEIKHIMLPPSRRGFKDIFVVFELMESDLHQVIKANDDLTKEHYQFFLYQLLRALKFIHTANVYHRDLKPKNILANANCKLKICDFGLARVAFSDTPTTIFWTDYVATRWYRAPELCGSFFSKYTPAIDIWSIGCIFAEVLTGKPLFPGKNIVHQLDLMTDLLGTPSLDTISRVRNEKARRYLTSMRKKQPIPFSQKFPNADPLALQLLQRLLAFDPKDRPTAEEALADPYFKGLAKVEREPSCQPISKVEFEFERRKVTKDDIRELIFLEILEYHPQLLKDYLNGTERSNFLYPSALDQFKKQFAHLEDNGGKSGPVYPLERKHASLPRSSVQSNTIPPKVTSNIVSFKDRYAPTAPFGSQLYKDSAAQRIAAAQAKPGRISGPVVPYDSGSIIKDAYDPRMLIRSAFPSHAIHPTYYYQQSCGQNEERSATGAEKDTSMQCKQSPQCGMAAKLAGDTAAATGAFSNSFFMARVGMPKMGNNDRAAHLQVRAQYDAGAVAAATTTTHRNTGVVDYGMTRMC

>Csa1M589750.1

MRKGGFSNNLNLKLNLPKEDQSIATFLTQSGTFKDGDLLVNRDGVRIVSQSEVEAPPPIKPTDDQLSLADIDIIKVIGKGNGGTVQLVQHKWTAQFFALKVIQMKIEESHRKQIAQELKINQSAQCPYVVVCYQSFYDNGSIYIILEYMDGGSLADFLKKVKKIEEPYLAALCKQVLKGLSYLHHERHIIHRDLKPSNLLINHRGEVKITDFGVSAIMENTYEEANTFVGTYNYMSPERIVGEGYDNKSDIWSLGLILLECATGKFPYSPPGQDGGWVNFYELMEAIVEGEPPSAPADQFTPEFCSFISACVQTDPKNRLSARELLEHPFIKMYEDKDIDLSSYFNDAGSPLATF

>Csa2M000340.1

MKKDTSMNSNLKLILPPPDELTFGFITRSGTFTDGDLLVNKDGVRIVSQTDDESPPPIKPSDNHLSLADLDSIKVIGKGNGGIVQLVRHKWTNQFFALKVIQMNAEESYCRLVAKELKINQLAQNPYIVVCYQIFYDNGAIFIILEYMDGGSLADLLKKVETVLEPYLAAICYQVLNGLIYLHHEKHVIHRDLKPSNLLINHRGEVKITDFGVSAILANTADQANSFVGTYAYMSPERLNGDKYDNKSDIWSLGLILLECATGQFPYAPPDKEKGWEGFFDVMVAVVELASPSAPEQFSPEFCSFISSCLQKDPQKRSSARELLVHPFIKKFENFDVDLAAYFKDAGSPLATF

>Csa3M839800.1

MAGLEELKKKLTPLFDAEKGLSMDSPVDPSDSYTFSDNGTVNLLSRSYGVYNFNELGLQKCTSWLADDSGSSERTYRCASREMRIFGAIGSGASSVVQRAIHIPAHRIMALKKINIFEKEKRQQLLTEIRTLCEAPCSEGLVEFHGAFYTPDSGQISIALEYMDGGSLADVLRLKKCIPEPVLSTMFQKLLRGLSYLHGVRHLVHRDIKPANLLVNLKGEAKITDFGISAGLENSMAMCATFVGTVTYMSPERIRNESYSYPADIWSLGLALFECGTGEFPYSATEGLVNLMLQILDDPSPSPSKHKFSSEFCSFVDACLQKDADARPTAEQLLSHPFIKKYENEQVDLAAFVQNVFDPTQRMKDLADMLTIHYYLLFDGPDDFWHHTKALFHESSTLSFSGKQFSGPNDIFGKLSEIRSTLAGDWPLEKLVHVVEKLQCRAHGRDGVAIRVSGSFILGNQFLICGDGVQVEGLPNFKDLSIDMESKKMGSFREQFIIEPSNLIGRYFIAKQELYIIQ

>Csa3M651720.1

MRPFQPPPATNPPTDRTRRRPHLNLHLPQRDNTSLAVPLPLPPTSSNSAPPPSTSQLHNANRPPDPLPPQRHPFTLSDFERVSRIGSGCGGTVYKVLHRPTGHVYALKVIYGNHEDAVRLQMCREVEILRDVDNPYVVKCHDMFDHNGEIQVLLEYMDRGSLEGTHIPQEHQLSDLARQILSGLAYLHSRRIVHRDIKPSNLLINSRRQVKIADFGVGRILEQTMDPCNSSVGTIAYMSPERINSDLNQGQYNGYAGDIWSFGVSILEFYLGRFPLAVERPGDWASLMCAICMAQPPEAPATASPEFRHFIACCLQREARKRWTAAALLEHAFITRKNGASQYQNKQAHHQNLRQLLPPPPLHPPSLS

>Csa2M000780.1

MKTKTPLKQLKLSVPVQETSIRSFLTASGTFHDGNLLLNQKGMRLISEEKESQTTDSKELDVDFSLEDLETVKVIGKGSGGVVQLVRHKWVGKLFALKVIQMNIQEDIRKQIVQELKINQAAQCSHIVVCYHSFYHNGAISLVLEYMDRGSLADVVRQVKTILEPYLAVVCKQVLQGLVYLHHERHVIHRDIKPSNLLVNHKGEVKITDFGVSAMLASSMGQRDTFVGTYNYMSPERISGGTYDYSSDIWSLGLVVLECAIGRFPYLQSEEQQSWPSFYELLEAIVAKPPPSAPPDQFSPEFCSFVSACIKKDPKERSSSLDLLNHPFIKKFEDKDIDVGILVASLDPPVSFPRQQQQ

>Csa1M042980.1

MALVRDRRHLNLRLPDLSDCRPRFPLPLPPSSAPPAPAAPSAISSSDLDKLQVLGHGNGGTVYKVRHKRTSTTYALKVVHGDCDPTVRRQVFREMEILRRTDSPYVVQCHGIFEKPSGDVTILMEYMDLGSLDSLLKKNSTLSEATLAHVSRQVLNGLHYLHSHKIIHRDIKPSNLLVNKNMEVKIADFGVSKIMCRTLDACNSYVGTCAYMSPERFDPETYGGNYNGYAGDIWSLGLTLLELYLGHFPFLPAGQRPDWATLMCAICFGEPPKLPEDASEEFRSFVECCLQKESSKRWTAAQLLTHPFVCRESSRSSDNR

>Csa2M021750.1

MQDIFASVRRSLVFRPPLDNDDSHSPAIGVGALVDKINSSIRKSRVFSRHSPSSSSLPPIPKDTDPPIRWRKGELIGCGAFGRVYMGMNLGSGELLAVKQVLIAANGASKEKAQAHVQELEEEVKLLKDLSHPNIVRYLGTVREDDSLNILLEFVPGGSIASLLGKFGAFPEAVLRTYTKQLLLGLEYLHKNGIMHRDIKGANILVDNKGCIKLADFGASKQVVELATISGAKSMKGTPYWMAPEVILQTGHSFSADIWSVGCTFIEMATGKPPWSQQYQEVAALFHIGTTKSHPPIPEQLSVEAKDFLLKCLQKEPNLRPTASELLKHPFVIGEETQSQLMSRDACTEPLETHSPQCTSELEMSKTPTHPGSSDICNLDSLRCSKVYSTNKLESDMWGRNSDDEMCQIDDKDDFMLDEVKIGSSIIHENMKSYNPICEPSDDEDCKFDRSPVVDRGSSLHEEALAPGSCSGAFDEEQNFSFPSGRSLSEDEDELTESKIKAFLDEKALELKKLQSPLYEEFYNSLNASCSPVFMESKQDESTPKYLKLPPKSRSPSRSPGNPSPALDAFGTGSPGSGSRGNANDQRSQLNDWKGLHGQSEAGSPSKNYSEIQRKWKEELDQELERKREMMRQAGVGAKTSSPKDKAMGRPRERTRFASPFRDDVSGAGRDNRENFIS

>Csa6M483320.1

MPLGWVKKLSRNKDHHNQNHPTSLNLFKSSSSSSSSSSPKTQPNNTITHKPKSFDEVSALIFSRNSPRSSRDLGSSGTASSGFSGFDSDSGHKSLPLPRPATSGLGIDHGAGNGSGSSSVSSDISSGSSDDQPSAQEQLQFGAYRGFADNRIETRARSPGPGSKGPTSPTSPLNPRFCGMSLESPPTHKLPLPPSAPTSPSSLTSMRAINIGDNNAAVQSKWKKGRLLGRGTFGHVYLGFNSVSGQMCAIKEVRVISDDSTSKECLKQLNQEITVLSQLSHPNIVRYYGSEMGEESLSVYLEYISGGSIHKLLQEYGAFKEPVIRNYTRKILSGLAYLHGRNTVHRDIKGANILVDPKGEVKLVDFGMAKHITNCTSMLSFKGSPYWMAPEVVMNTNGYSLAVDIWSLGCTVLEMATSKPPWNRYEGVAAIFKIGNSKDIPEIPDSLSSDARSFVQLCLQRDPSARPSAAELLDHPFVQDAVTPRASDVNLSVDAFPFSFDGIQTSPLLDRHPNRKSISICDGDYVTNPTFSSRAPSPRGNGRLITSLPVSPCSSPLRSYGPTHQSCYLSPPHTSYMGVGQSGYNLNEYAYNTRPNTLFTLDPSRESSLLKVQTHLGSPRRPL

>Csa6M483320.2

MPLGWVKKLSRNKDHHNQNHPTSLNLFKSSSSSSSSSSPKTQPNNTITHKPKSFDEVSALIFSRNSPRSSRDLGSSGTASSGFSGFDSDSGHKSLPLPRPATSGLGIDHGAGNGSGSSSVSSDISSGSSDDQPSAQEQLQFGAYRGFADNRIETRARSPGPGSKGPTSPTSPLNPRFCGMSLESPPTHKLPLPPSAPTSPSSLTSMRAINIGDNNAAVQSKWKKGRLLGRGTFGHVYLGFNSVSGQMCAIKEVRVISDDSTSKECLKQLNQEITVLSQLSHPNIVRYYGSEMGEESLSVYLEYISGGSIHKLLQEYGAFKEPVIRNYTRKILSGLAYLHGRNTVHRDIKGANILVDPKGEVKLVDFGMAKHITNCTSMLSFKGSPYWMAPEVVMNTNGYSLAVDIWSLGCTVLEMATSKPPWNRYEGVAAIFKIGNSKDIPEIPDSLSSDARSFVQLCLQRDPSARPSAAELLDHPFVQDAVTPRASDVNLSVDAFPFSFDGIQTSVSLSASLGLLSESQSQDEIISL

>Csa6M483320.3

MPLGWVKKLSRNKDHHNQNHPTSLNLFKSSSSSSSSSSPKTQPNNTITHKPKSFDEVSALIFSRNSPRSSRDLGSSGTASSGFSGFDSDSGHKSLPLPRPATSGLGIDHGAGNGSGSSSVSSDISSGSSDDQPSAQEQLQFGAYRGFADNRIETRARSPGPGSKGPTSPTSPLNPRFCGMSLESPPTHKLPLPPSAPTSPSSLTSMRAINIGDNNAAVQSKWKKGRLLGRGTFGHVYLGFNSVSGQMCAIKEVRVISDDSTSKECLKQLNQEITVLSQLSHPNIVRYYGSEMGEESLSVYLEYISGGSIHKLLQEYGAFKEPVIRNYTRKILSGLAYLHGRNTVHRDIKGANILVDPKGEVKLVDFGMAKHITNCTSMLSFKGSPYWMAPEVVMNTNGYSLAVDIWSLGCTVLEMATSKPPWNRYEGVAAIFKIGNSKDIPEIPDSLSSDARSFVQLCLQRDPSARPSAAELLDHPFVQDAVTPRASDVNLSVDAFPFSFDGIQTSPLLDRHPNRKSISICDGDYVTNPTFSSRAPSPRY

>Csa3M182770.1

MPSWWGKSSKDVKKKTSKESFIDSLHRKFKNSPEGKVNSRSGSSRKRGGDTVSEKGSKSPISRSPSPSKEVARCQSFAERTHSHKLPLPDLRPVGVGRTDSGISVAAKSKLERSSKTSSFLPLPRPACIRSRPDPADLDGDLVTGSVFGESSSDSDDPNDSRQRSPPATDYDIGARTVIGSTEPSETLKDQSPTVVQKNLKEGKKAESLPFPHKNSSIPKRRPLSSNVTNLQVPRHGAFFSAPDSSMSSPSRSPMRIFSTEQVMNAAVWAGKSHPDVILGGSGHCSSPGSGHNSGHNSMGGDMAGHFFWQQSRGSPEYSPVPSSRMTSPGPSSRIQSGAVTPIHPRAGAPPAESQTCWPDEKQTHRLPLPPIAISICSPFSHSNSAVTSPSVPRSPGRTETPASPGPRWKKGKLLGRGTFGHVYVGFNSESGEMCAMKEVTLFSDDAKSRESAKQLMQEIALLSRLRHPNIVQYYGSETVGDKFYIYLEYVSGGSIYKLLQEYGQFGELAIRSYTQQILSGLAYLHAKATVHRDIKGANILVDPNGRVKLADFGMAKHITGQSCPLSFKGSPYWMAPEVIKNSNGCNLAVDVWSLGCTVLEMATTKPPWSQYEGVAAMFKIGNSKELPVIPEHLSDDGKDFVRLCLQRNPHHRPTAAQLLEHPFVKHAAPVERPILISEPSDTTPGVTNGVKILGIGQSRTTSMDSDGRLAVHSSRVSKAVLHASEINISRNISCPVSPIGSPLLHSRSPQHPSGRMSPSPISSPRTMSGSSTPLTGCGGAIPYNHLKQTIYLQEGFVSMPKSLNSSPYSSGISFHDSNPDIFRGLQPGAHIFSEMIPENEVLGKQIGRPAYSEVYDGQHILADRVSRQLLRDHVKANPSLDLSPSATLSGRMNGI

>Csa5M166980.1

MPSWWGKSSSKEVKKSKESLIDTLQRKLRTTDGKTNSKSGESPRHCNDTISEQGSRSPILSRSVSPSKQVLRCQSFSERPQAQPLPLPGVQPPIVGRTDSGISISPKPRSERGSKPTSFLPLPRPACIRGRPNHADLDADVGVGSVSSESSTDSTDLLDSRHRSPRATDYDLGTKTAASSPSSVILKDQSSTVTQPSLQKARKPANISLSNHIFSTSPKRRPLSSHVPNLQVPYHGNVCIAPDSSMSSPSRSPIRAFSSEQVINNAVSTGKFYMDVTFPGSGHCSSPGSGYNSGHNSMGGDLSGQLFLQQSRGSPEYSPVPSPRMTSPGPSSRVHSGAVTPIHPRAGGIPTDSQTSWPDEKQTHRLPLPPVAISNAPFSHSNSAATSPSVPRSPGRADNPASPGSRWKKGKLLGRGTFGHVYVGFNSESGEMCAMKEVTLFSDDAKSKESAKQLMQEITLLSRLRHPNIVQYYGSETVGDRFYIYLEYVSGGSIYKLLQEYGQLGDSALRSYTQQILSGLAYLHAKSTVHRDIKGANILVDPTGRVKLADFGMAKHITGQSCPLSFKGSPYWMAPEVIKNSNGCNLAVDIWSLGCTVLEMATTKPPWSQYEGVAAMFKIGNSKELPEIPDYLSHDGKDFVRQCLQRNPAHRPTAAQLLEHPFVKHAAPLERLILGSEHSDPTPGITNGVRTLGIEQGRNPSFLDSDRSAAHSSRLPTAAFHSSEIHIPRNLSCPVSPIGSPLVHSRSPQHPSGRMSPSPISSPRNMSGASTPLTGGSGAIPHQHLKQSLYLQEGFGNLPKPSMAPYSNGPSFHDTNPDIFQGIQPGSHIFSELVHHETDFLGKQFGKPAWELYDGQAVLADRVSRQLLSDHITTPSLDLSPSSLLTNRK

>Csa6M490220.1

MRWLRNISFTPSSMVRLPGSTTGGGDSPTRRSTTRGSAENNYRGIVWRFGASRYSRHRKLRNLSGREHVDSSLAKWSDTAPEPVSLSRSPSTSDHPAVPLPLPEVSPLFQPRERISTSNSAGGEGDCPLPSPKGSRGRAGDERDVDRDRNAPPQKIGGGISPNASIKSVSDSVGERHKKEGQIEARLSGRANQDARRYPENSRNGFWIDVPSRSAPTSPYTSPTPSPQRNISVGNHVWSAPELPSSAMMRGVPPAFFDCSTLSTESSPMHSPRGKSPHLDPRSPTGPTSPLHAKISHETHAMRREGSGHVSVHPLPLPPGVPMPSASIPTMASAPTSINLSSPPVSSPSHSIPSASCSMALPSTPMASPSTPISQANTKSESISMKNQWQKGKLIGRGTFGSVYVASNRQNGALCAMKEVELFHDDPKSAESIKQLEQEIKLLSQLKHPNIVQYYGSDIIDDRLYIYLEYVHPGSINKYVREHCGAMTESVVRNFTRHILSGLAYLHSTKTIHRDIKGANLLVDSCGVVKLADFGMAKHLTGQVADLSLKGSPYWMAPELLLSVMQKDNTPDLALAVDIWSLGCTIIEMFTGKPPWSEYEGAAAMFKVMKDTPPMPESLSYEARDFLKCCFQRNPAERPTAAMLLEHPFMKNLQYTDASSCSQVVAGASLMDKCYSPSKQYSSKSDQSSMLPSPQNSKGKLAADNVIGPLSHHETSDLTVMSRYSPRSTLEALPMVSPLRSVPNAHHYGSPTNAADIVNQINRKNHTLI

>Csa2M360650.1

MPSFNKSASNSRLQQKVPDSDFFDFSVGLPEPFIQRRLTRQRKLRHLTDQDVGFEHPRSLPDSPDISAKPKSPLGGSERWSSSPSPQPLPLPELFPVRSPEFGSNYGQGRVGSPVETSVRKCSDHATTNVSRSFGHNQRRVATDLTLEVVDGNSRTGATAFTSPQSLSANSGKLNPNKEFLFGCSDQTPNNKGRGSPTHPSAERGNYNLTPKKSSSKSAPTSILPSPVVSPRKSYNGNHFVPGLSHHEHQESPSNNSPKVPPLKTALSSQPFPLHSPTARSSISNSRSHNDMTFPLHTKLQKDNSIDRSESHAHVNAHPLPLPPPLVASSQASAQSLPSNVHHVIEKPFISSMKGQWQKGKLIGRGTFGSVYLATNRETGALCAMKEVDLIPDDPKSAECIKQLEQEIEVLSHLKHPNIVQYYGSEIIGDCFYIYLEYVYPGSINKYVRERCGAITESIVRNFTRHILSGLAYLHSTKTIHRDIKGANLLVDSSGVVKLADFGMAKHLTGQYDLSLKGSPYWMAPEVIKAAMLKDANPDLALAVDIWSLGCTIIEMLNGKPPWCEFEGHQVMFKVLNKTPPIPEKLSPEGKDFLQCCFQRNPADRPTAMVLLDHPFLRSSSDSNASIPTSAFSTMNLLEKLLSPKDPLNPKGDQAQNSSGTLTSNDYLSCRSPSTNISSNVPASAVANHHFSISRTHKREVPHL

>Csa5M385380.1

MNRLPQFFSPRNKRKPMDPRKIPGKPKLGRLNAAKNIDYDAPSSSSSLEDSSGSLYTRSMENPDPSSFRIKGLDGEVDLICRTLGLAGPDDFAIPMEAWEARKVRSSSELLPRSRLYPMDTSPKTEEISEDKEDKEIQDELCRRVKDSVRISVDLSKTKTEFAELNERRMATATGCSSRSGINGARPPLLKPPPSMRLPNFDNAYSTWDILKGFAPLVEDEHQEEVGERVEPLVEVEGEGEGNTVRPVENASLIGSWGSFTTSNDDDSSSSTTEPANISPNMRVNPIITSWVLGRLLGRGSFGSVYEAISEDGTFFALKEVSLLDEDSQGRQSIYQLQQEIALLSEFEHENIVQYYGTHSDGSKLYIFLELVSQGSLMSLYQRTSLMDSIVSAYTRQILSGLKYLHERNVIHRDIKCANILVDVNGSVKLADFGLAKATKLNDVKSCKGTAYWMAPEVVNGKGQGYGLPADIWSLGCTVLEMLTRKLPYSEFESHMQALFRIGKGKPPAVPESLPKDAQDFILQCLQVNPKDRPTAADLLNHSFVKRPVSSLSGLASPYNRPGRRI

>Csa6M425140.1

MQDFFGSVRRSLVFLAPDGDDGGRFGGLVEKIGSSIRKSRNGLFSKQSLRALPPVAKEDAPPIWWRKGELIGCGAFGRVYMGMNLDSGELLAVKQVSIAANSASREKAQAHIRELEEEVRLLKNLSHPNIVRYLGTAREEDSLNILLEFVPGGSISSLLGKFGSFPESVIKTYTKQLLLGLEYLHKNGIMHRDIKGANILVDNKGCIKLADFGASKKVVELATINGAKSMKGTPYWMAPEVILQTGHSFSADIWSVGCTVIEMATGKPPWSQQYQEVAALFHIGNTKSHPPIPEHLSAEAKDFLLKCLQEEPNSRPTASDLLQHPFVSCEYQEPIAAVRASSMESGKQMADSRLNSNDLKKSTILRSTCEGLKDICEMGSLRCSSVFSGNYGSRSNWGSSNFEDDMCQIDDKDLFATSSMKYNSIISSNDLNKSFNPMCEPTDDLDESSELGGNLMELSSVQTVKGNDSTFPCGQSAAEDDEEVTESKIKAFLDEKALELKKLQTPLYEEFRSTVNAANAIGTVGIENIKSVSNFLKLPPKCGSPSKLRGKRLSSVDVGNYSSRQSRIKQASILQDRALQEIQSPQLGEWNKLLQNQPDSSTLSFSERQRKWKEELEEELKRKREMMRRGIVGAIH

>Csa1M532310.1

MLKTTALSWTRSNSPIGKGSFATVSLGIRKPDARIFAVKSVQQTQTLRPQIDCLENEIRILRSLNSPYVVAFLGDDVSHESPTTSFRNLHMEYLPGGTAADDPTGTRDDKLLRERTWCLVSALSYIHSKGIVHCDVKGRNVLIGLNPGFLKLADFGSAIELHGPGHRSRDSLAPRGSPLWMAPEVVRGEFQGPESDVWSLGCTVIEMVTGKPAWEDFGADTLSRIGFSDDLPDFPTCLSEVCRDFLRKCLRRNPSERWSCDRLLQHPFLAAAAAAAASPKIAVENSPRCVLDWVNVSFSDDEEEIPHADEASGSGGQENEIYGKERIGKLSTTSEWPNWESDGWSAVRSSYSEAAAETEASCRKEEEEEEGGGAEWECGNLRRVEGEMEGRSWEYSEFVRRDNHGKLGAEYSNPGGMIIPERPRNNFGGGGGCGSGGLSFRRLGYEISEITTIITSWIYSIELILCCYYWNILMKKLVLFGNYTFLPFFSA

>Csa6M513560.1

MEWIRGRTIGRGSSAAVSVATDIRFGQVMAVKSVEFSHLDFLKREQRILSQLNCSRVIGYKGFDVTLENGNLMCNLLMEFAPGGSILDAMEKAGGRLDEATAQFYTREVLSGLQYVHSNGVVHCDIKCCNILMGEDGIKIADFGCARRVEEVSGGNLAGTPIFMAPEVARGEKQGFAADVWSVGCAVIQMVTGRVPWANLSDPLAAIYRIGSGDDLPEIPRIMSEQGKDFLRRCLIRDPEERWSVNELLKHPFVQEQKSHPKQNSRTPTSILDQGIWDTVNDPETVESPIRPKIQRTPLQRIQQLNEVSTIGIPNWECDEDWITVRSIGLEENDIVSVMETPSSFQSIKMETNNGVGDQDYINVSRSRSSDYGSRKSSKAKNRDFPAMSSVFNQIPGVYFVL

>Csa2M416770.1

MDWTRGHVIGHGSSATVFLATDSPSRHVFAVKTAQLSHSQSLRKEQQFLSSLASPYIVSYRGFEVSREQSGVTMFNLFMEYLPNGSLADTIRRRGGQRLDEATIVIYTRQILMGLQYIHSKGIVHCDIKARNILIGLDGEAKLADFGCAKRATSQTDPICGTPLFMAPEVARGEHQGFPSDIWSIGCTIIEMASGGGSPWPKTTDDTDPISALYRIGYSGESPEIPCYLSEEAKDFLEKCLKRNPSERWTASELMNHPFLRELNCRREWKTEEVHSESPTSILEQGIWRSIEESEIRGRELVRSNGWEAAAAEEQIRRLWMISGEPRWEEDENWITIRRKEEGEKNGGADESEVKKCSNYSNNNNNNNSNWGDKKRGSGGKEWLQMELGNISCRISRNDLGDYSCRNVISLVNNNYPLSFHTLTPIFLPLMPTIL

>Csa7M043040.1

MAWTRRSPVGNGSSATVYLASTASSGQLFAVKSAELLKSDFLKIERRVLSSLSNPSIVGYKGFDVTRENGKLMYNLFMEYAAGGTLADEIFRRGGRIKEATAAFYTREIVRGLEYLHKQGLVHCDIKAKNILIAGDGLKIADFGCSRWVCESEAVIGGTPMFMAPEVARGEKQGISSDIWALGCTLIEMVTGAPPWKITDDPVSVLYRIGYSGESPEIPSFLSEKGKDFLRKCLRREATERWSASQLLEHPFLGELSSGLEEIKELHLYSESPTSILDQSLWNSLEEESETLLRTEQWDDDRIERLATFSGEIKWELGDENWITIRSYVDGEDEDELNFSRNDLDLVEEGRIVSEEIQYLELLDKTVSFRVR

>Csa7M430790.1

MKRNLTLGGLEVEVEDGSSEFNNGVQWKRGRLIGKGSFGSVFLASLKPHITIKYCTFPSVMAVKSAEISVSETLQKEKQNYDSLKGCNSLIKCFGEEITTDHNGHMIYNLLLEVATGGTLAHHIKNTGGKGLEENVVRNYTKSIIKGLIHIHRSQYVHCDLKPANILLLPKNNTTKDRQFIAKIADLGLARRTSKTKASYCLGGTFSYMAPETFIDGVQESASDIWALGCVVLEMLTGNRAWAATNKVGIMKEMTENFLGMPKIPEGLSAEATMFLKNCFVRKPEFRFTAEMLMIVPFVAAVEDQEQNFNTVKAPTFVTKWPMQFKRQRIIPIKAV

>Csa6M490950.1

MDWVRGDEIGYGSFATINLATSCSGDRFPPLMAVKSSGLVCSASLKNEKQVLDLLGDDCPQIIRCFGDSCSVENGEELYNLFLEYASGGSLADRIQSRGGRLPEFEVRRYTRTILEGLRYIHGKGFVHCDVKPRNILVFGDGDAKIADFGLSKKAGKNRVGTGEETGKFQLRGSPLYMSPESVNDNEYESPCDIWAVGCAVVEMLTGKPAWNCRPESNVFALLIKIGIGEELPEIPKDLSEEGKDFLKKCLVKDPLKRWTADMLLKHPFVAESGRCVPLAGVEEVSTSPRCPFDFEDWASIHSQESDPRNEEEANCWLNNWSCSPRERLLELVGNGAVEWSVTDNWVRVR

>Csa2M278170.1

MEWIRGDQLGRGNFATINLAKLTKGFDQFPPLMAVKSSVSSLSSVSSLKNEKQILDRIGVCPQIITCYGDGFSVEKDGDKCYNLFLEYANGGSLADALRIHGGGLSEFDVRRYTRAILCGLQHVHGNGFVHCDLKLSNVLIFGNGEVKIADFGLAKSAGKFAAVETEERFEWRGTPMYMSPEIVNDGEYESPCDIWALGCAVVEMVVGKPAWRVGPETDMFGLMMRIGVGDEVPEVPENLSAEGKDFIRRCFVKDPSKRWTAEILLNHPFVAGAGDTVTLKEVELATESPTGPFDFPEFVCSGQGSDEWSFCSSSSSPEVLSRVRQLMTGKPLDWSVMDSWVTVR

>Csa7M378450.1

MENWVPVKVLGQGSYAVVCLAKQSIRKCSDNNLPYYFALKIYPLQHNSSLLWEEQVLKQFKGCPEIVQYFGSEITRGGSFCNDKDFYTLKLEYAAGGTLDDLIKQRDKLPEDEVKDYLRMILKGLSCIHSKGFVHVDLKPNNILAFPQSDGKMKLKIADFGQAERCKYRDDNGQHKRYGYCSSLKFKGSPRYMSPESIIFNEVDDAHDIWSLGCILVKMISGKCVWDGYTDSKQLMIEVLDNKIMATIPGELSEQGKDFIRKCFIRSYKQRWTADMLLQHPYLNQENEAPMKEDEATMKDDEATMDGGSYSFNRLILKFPIAKLFLTCFNQ

>Csa7M407720.1

MDQDWVLVKALGEGSCGLVCLAKQITKEESDLHYYFAVKRASLRYNSSSLLWEEHVLKHFTDCPEIVQYLGSEVTGGGDFLDDKELYNLKLEYAAGGTLADLIKQRNKLPEDEVKKYLQMILKGLSCIHRKGFVHVDLKPDNILAFPQSDGKMKLKIADFGLAERSCKRGEDDQEDRGSKYYSGALKVRATHRYMSPESIVFSEINGLHDIWSLGCTLVQMVSGERVWNDCKSYEELITKLLISEEIPTIPEELSKQGKDFLEKCFVRNYEQRWTADMLLQHPYLNEENKDTKNGDEKLKLPKAIVFLPHQFFQSKTDMLLQQTKNGDEKLKPSKAIFLPHQFFKSKAQSCNN

>Csa3M829110.1

MTKSCVVSQVQISGNLHEFLKTKRRILKIAKKSAPRNNWNDTIKHNYEKFGEDFTIEEFHGSIWTGSLLFRKTESTLVFLAKKNRAPINNSNSNLPEEFLVKSSLMESSSSLRHEKEVLSNLGPFTNLVDCYGDEITVTKSGEEVYNVFFEYCSGSSLRNHILKFGPNGLQDDEVRRYTRDIVRGLYYMHCNGRYIHGDIKSRNILLSHGMAKLASFGLARKLTAEVICEEEISGSGPYASPELAREGYLGWPADIWALGCVVLEMFTGKSAWSFEDAYRYLMDSNNEKIPEIPKNISREGRDFIKKCLIRSPYKRRPIWLLIKHPFVCQ

>Csa6M450400.1

MEMPGRRSDYSLLSQIPDEEVGTGVSTSFYDSIAAGGNVIKGRTDRVFDWDGIGDHRLNTQAYRTGNLYSWIGLQRHSSGSSYDDSSLSSDYYAPTLSNAAANEINALEYIHDDDFRVMKAVGSGGSSGKSWAQQTEESYQLQQALALRLSSEATCADDPNFMDPLPDEAALRSLSISAEAISHRFWVNGCMSYFEKVPDGFYLIHGMDPYVWSLCTNLQEDGRIPSFESLKTVDSSIASSIEVVLIDRHSDASLKELQNRVHNIASSCATTKGVADHLAKLVCNHLGGSVSEGEDDLVSSWKECSDDLKECLGSAVIPLCSLSVGLCRHRALLFKVLADSIDLPCRIARGCKYCTRDDASSCLVRFGLDREYLIDLIGRPGCLCEPDSLLNGPSSISISSPLRFPRLKPIESIIDFRSLAKQYFLDSQSLNVVFDEASSGNVVSGKDAAFSVYQRPLNRKDGDRKIIVVTGDKDRNSQLLNKKAAQLNTQDGKSEQFRSCVTSQYSVQSTPLVENVVPLNHISPIGSKDSEHLLALSHPRVDHANNLPFVDGSQLIRKPNDLSLGLEDLVIPWKDLDLREKIGAGSFGTVYHADWHGSDVAVKILMEQDLHAERFDEFLREVAIMKCLRHPNIVLFMGAVTEPPNLSIVTEYLSRGSLHRLLHRPGAREVLDERRRLNMAYDVAKGMNYLHKRNPPIVHRDLKSPNLLVDKKYTVKVCDFGLSRLKAHTFLSSKSAAGTPEWMAPEVLRDEPSNEKSDVYSFGVILWELATLQQPWGNMNPPQVVAAVGFKGKRLEIPCDLDPRVATIIEACFASEPWKRPSFYEIMESLKPLIKPATPHQVRSNVSLVTQ

>Csa3M749850.1

MEMPGRRLNYTLLSQIPDDQYSGGVAGASTSFIETSSGEGKNDRRKLERGLDWEVAGDHRAGQQQQVNWIGNMYSAFGLQRQSSGSSFGESSISGEYYAPTPSTTVANETDVFGCTHDDVLRVGGDSRAQAGEMAAGAGGSSAKTWAQQTEESYQLQLALALRLSSVATCADDPNFLNPFHDDSALRRLIGSAESVSHRFWVNGCLSYLDKVPDGFYLIHGVDPYVWTVCTSLPDNDHMPSIESLKSVDPSTDSSIEVVLIDRCTDPNLKDLQIWVQNISCSSITTEEVVDQLAKLVCRSLGGSVSGEDALVSIWKECSDNLKETLGSVVIPLGGLSVGLCRHRALLFKVLADTIDLPCRVAKGCKYCSCHDSSSCLVQFGLDKEYLVDLIGNPGCLYEPDSLLNGPSSILISSPLRFPRLNPVEPATDFRLLAKQYFSDCQLLNVVFDEASSCNHSEITVDGEDGALPLYPKQFDRKFTNRSNQMLVTGDSDEKSILLHPKTSQPNSHDRDFQLYKPRDNSHSVIQPTVLVEDSIPLKYIPHNNRGSMQSLLDMSQPRMDSTMDVRFAAGGQLIPSNRSNTLPLGAEDLDIPWGDLVLKERIGAGSFGTVHRADWHGSEVAVKILTEQDFHPERVNEFLREVAIMKSLRHPNIVLFMGAVTKPPNLSIVTEYLSRGSLYRLLHKSGVKDIDETRRINMAFDVAKGMNYLHRRDPPIVHRDLKSPNLLVDKKYTVKARTFLSSKSAAGTPEWMAPEVLRDEPSNEKSDVYSFGVILWELATLQQPWCNLNPAQVVAAVGFKGKRLDIPRDVNPKLASLIVACWADEPWKRPSFSSIMETLKPMTKQAPPQQSRTDTLSVM

>Csa1M574260.1

MKHIFKKFHIGSNHEPNRSNENPSPVAAASSSPCVSDNRPATAPGQTSGNSPPSPSSSPSLATTSPGGGNVTQVSVPPNRSDYFSSEEEFQVQLALAISASNSDFRDDPEKDQIRAATLLSLGNHRIDSTARDQGDAAEVLSRQYWEYNVLDYEEKVVNGFYDVLSTDSAVQGKIPSLSDIEASFGSSGFEVVMVNMTIDPALEELVQIAQCIADCPGTEVRVLVQRLAELVMGHMGGPVKDAHFMLARWMERSTELRTSLHTSVLPIGSINIGLSRHRALLFKVLADSIKMPCRLVKGSHYTGVEEDAVNIIKLEDEREFLVDLMAAPGTLLPADIFNAKDTTNFKPYNPKVSRIPSLHHSNDVGISSAKPTSGLEEGSSQNFGAEAISLMDGKLGYGRTESVPSSSGTGTSRYKGAHFGDGNVRLNVNVVPFGQSSEDSKNLFADLNPFLIRGTGKSFIPNKFSDNKSEELQKPTIGHPPVPLWKNRFAFNAVPNKNEYDYMEGRFPRISRGPNDQNMALSSSNSTGSESVKPGGSGTSNDLSASVRSAEVGSSSSNMYAQPAFGMMEPNILPFIDEQNRKSNGEHSGNTDMEDEKVDAVDGRDNLIRFDNRRKFTYERSVGTNLILKDSGNPGLLVNPSSNRFEQVYDDVDVGQCEIQWEDLVIGERIGLGSYGEVYHADWNDTEVAVKKFLDQDFSGAALAEFKREVLIMRQLRHPNIVLFMGAVTRPPNLSIVTEFLPRGSLYRIIHRPNCQIDEKRRIKMALDVARGMNCLHTSNPTIVHRDLKSPNLLVDKNWNVKVSDFGLSRLKHNTFLSSKSTGGTPEWMAPEVLRNEPSNEKCDVYSFGIILWELATLRLPWSGMNPMQVVGAVGFRNQRLEIPKEVDPTVARIIWECWQTDPNLRPSFSQLANILKPLQRLVLPPHSDQPSSSVLQEISVNSTP

>Csa4M646020.1

MKHLLRKLHIGGGLNEHQRLSDARPVTRPSSSPSPGPSPNSNPSGSSSSGSSSSLSMASSTTMGRLEAVESVVDPAASGDVGGGCVDFNALEEEFQVQLAMAISASDPDSRQDTESAQIDAAKRMSLGCSPSVSGSKALAEFLSLQYWSYNVVNYDEKVMDGFYDLYGITASSSTRGKMPLLVDLKEICVTSDIDYEVILVNRLLDPELQQLERQAYNIFMECRVSEYGFILSGLVQKIADMVVARMGGPVGDAEEMLRRWTRRSYEMRSSLNTIILPLGRLDIGLARHRALLFKVLADRINLPCILVKGSYYTGTDDGAVNMIKIDNGSEYIIDLMGAPGTLIPSEAPSGQFSNYGFDRRPADVIEVPEDTPILQNEGAEAVSISSTQDEVADVCNLISKEASDLDAQSKENIRNFIEEIQSGSSGYDFAKLLESESSACEGSLGAFAQSASAQKKKVKKVSKYVISAAKNPEFAQKLHAVLLESGASPPADLFSDIESQDNGESKETFQMYPINGKGIDVGLQSHSYILASHGQSSATSTEAEYLNNVVHENKQKVPSGGLSEEQMANTNANNHSIFWPHSMKNEGFVFVDVNGEAGKLVDVNGTFHREHMDDVLLTSDTDSHKKLGSALVSEERRLLQDKSGGTLQCFDLCEKPLENLLQTDDSKLHASDEHNETINPILGEVAEWEIPWEDLHIGERIGIGSYGEVYRADWNGTEVAVKKFLDQDFSGAALVQLKCEVEIMLRLRHPNVVLFMGAVTRPPHFSILTEFLPRGSLYRLLHRPNSQLDERRRLKMALDVAKGMNYLHTSHPTIVHRDLKSPNLLVDKNWVVKVCDFGLSRVKQNTFLSSKSTAGTPEWMAPEVLRNEPANEKCDVYSFGVILWELTTCRIPWKGLNPMQVVGAVGFQNRRLEIPQDVDPAVAQIICDCWQTDSQLRPSFSQLITRLRRLQRLVQKTDSGNQISE

>Csa1M042730.1

MKNLLKKFHIMSSGQSDDVAEGSTSSRSNKVMEVSSPDKLPSRSRPTHFSSEHKPFSGISGWLNSVTNRRSPSPPSSADPTAGEIMEPSDSVSSRDAAMDTSRHDSGSSNSRDPDIEEEYQIQLALEMSAREDPEAAQIEAVKQISLGSCDPDNTPAEVIAFRYWNYNSLSYDDKILDGFYDLYGVFTRSTSERMPSLVDLQGAPMSDSVTWEAVLINKAADANLLKLEQTALEMAIKMQTESPISVNHYLVRKLAALVSDHMGGPVGDPEKMLRKWRNLSYSLKATLGSMVLPLGSLTVGLARHRALLFKFLADGVGIPCRLVKGPQYTGSDDVAMNFVKIDDGREYIVDLMADPGALIPADVAGSHVEYDGSPFSASPVSRDVDSSQAASSSSGVGSSLEGNSDFGISDRKPKARNLSATKEYDSPNIDKVPSRDFASKSNYPGMHTRSPSWTEGVSSPAVRRMKVKDVSQYMIDAAKENPRLAQKLHDVLLESGVVAPPNLFTEAYPDQIDVIVESKSPTEDKDQSRKLPGICESADKNDPRLSNFLPPLPQPRLHSRASPTHGQQLYIKPLEFNLSLDSREAGGQPIPLPFEVTPVKYGRNVPVAAAAAAAAAVVASSMVVAAAKSSDANLEIPVAAAATATAAAVVATTAAVNKQYEQVEADAALYELRGSGDREHDACGDNSEGERISDRSAGNESTKSDITLDDVAECEIPWEEISLGERIGLGSYGEVYRGDWHGTEVAVKRFLDQDISGESLEEFKSEVRIMKRLRHPNVVLFMGAVTRAPHLSIVTEFLPRGSLYRLIHRPNNQLDERKRLRMALDAARGMNYLHNCTPVVVHRDLKSPNLLVDKNWVVKVCDFGLSKMKHSTFLSSRSTAGTAEWMAPEVLRNEPSDEKCDVYSYGVILWELSTMQQPWGGMNPMQVVGAVGFQHRRLDIPDNLDPAIADIIRKCWQTDPRLRPSFAEIMAALKPLQKPLSSSQVPRPNAPAGSGRDKARLLQVTEDPSG

>Csa1M042730.2

MKNLLKKFHIMSSGQSDDVAEGSTSSRSNKVMEVSSPDKLPSRSRPTHFSSEHKPFSGISGWLNSVTNRRSPSPPSSADPTAGEIMEPSDSVSSRDAAMDTSRHDSGSSNSRDPDIEEEYQIQLALEMSAREDPEAAQIEAVKQISLGSCDPDNTPAEVIAFRYWNYNSLSYDDKILDGFYDLYGVFTRSTSERMPSLVDLQGAPMSDSVTWEAVLINKAADANLLKLEQTALEMAIKMQTESPISVNHYLVRKLAALVSDHMGGPVGDPEKMLRKWRNLSYSLKATLGSMVLPLGSLTVGLARHRALLFKFLADGVGIPCRLVKGPQYTGSDDVAMNFVKIDDGREYIVDLMADPGALIPADVAGSHVEYDGSPFSASPVSRDVDSSQAASSSSGVGSSLEGNSDFGISDRKPKARNLSATKEYDSPNIDKVPSRDFASKSNYPGMHTRSPSWTEGVSSPAVRRMKVKDVSQYMIDAAKENPRLAQKLHDVLLESGVVAPPNLFTEAYPDQIDVIVESKSPTEDKDQSRKLPGICESADKNDPRLSNFLPPLPQPRLHSRASPTHGQQLYIKPLEFNLSLDSREAGGQPIPLPFEVTPVKYGRNVPVAAAAAAAAAVVASSMVVAAAKSSDANLEIPVAAAATATAAAVVATTAAVNKQYEQVEADAALYELRGSGDREHDACGDNSEGERISDRSAGNESTKSDITLDDVAECEIPWEEISLGERIGLGSYGEVYRGDWHGTEVAVKRFLDQDISGESLEEFKSEVRIMKRLRHPNVVLFMGAVTRAPHLSIVTEFLPRGSLYRLIHRPNNQLDERKRLRMALDAARGMNYLHNCTPVVVHRDLKSPNLLVDKNWVVKVCDFGLSKMKHSTFLSSRSTAGTAEWMAPEVLRNEPSDEKCDVYSYGVILWELSTMQQPWGGMNPMQVVGAVGFQHRRLDIPDNLDPAIADIIRKCWQT

>Csa3M892210.1

MPHRTTYFFPRQFPDRGFDSASTSKHILDHEKKINKDTFSTESDAKPTPRPARDFSVTKSSAVSDLFTGDKAQTNKKLPAFYDWLVDKKATRSATAHVKTWLSNCDEDRELLLPPPTSEPEHDTTSVKDRSVDRNFDRQVSLPRLSSGSSYAGSLFSGTGTGTVDGNFSSDVKDSSASKILSSHTARPEEIEVGDDKENIAQKATESYYLQLALAATLRSHANLAGDPVLMEEGRVEITDAETVSYRLWVSGCLSYSDKISDGFYNILGMNPYLWVMCNDFEEGRRLPSLMSLRTIEPSETSMEVILVDRRGDSRLKELEDKAQELYCASESTLVLVEKLGKLVAIYMGGTFPVEQGGLHLHWKVVSKRLREFQKCIVLPIGSLSMGLCRHRAILFKKLADYIGLPCRIARGCKYCVADHRSSCLVKIEDDKKSLREYVVDLVGEPGNIHGPDSSINGGFQSSMPSPLQISHLKEFQEPYVESYFNHQTVGSKQICGFPEYPLRSGFGQYQMKGGSTLRMSSGAETDKLVDQACMGIGSTQLCLETKVSKECVLQNHIMPSTGADASEVLSSVGGASLCENKVVIEEIYQEEAVVAAGISVNETINPSKLTLSTQTDSKEIVGRSQNCSASTYPKYLTLEPSLAMDWLEISWDELHIKERVGAGSFGTVHRAEWHGSVAIMKRVRHPNVVLFMGAVTKRPHLSIVTEYLPRGSLYRLIHRPSYGELMDQRKRLRMALDVAKGINYLHCLNPPIVHWDLKSPNLLVDKNWTVKVCDFGLSRFKANTFISSKSVAGTPEWMAPEFLRGEPSNEKSDVYSFGVILWELVTMQQPWSGLGPAQVVGAVAFQNRKLSIPSSTSPLLASLIESCWADDPVQRPSFASIVESLKKLLKSPQQLIAMGGT

>Csa6M330990.1

MNPAAAAAADGGGGCSGPAAVNFTNKQYLNILQSLGQPVYIFDLNYHIIYWNRAAEIVYGYSAAEALGQDAIELLVDPEDFAITNHVILRVMAGENWTGHLPVKNKMGQKFVVVATNTPFYDDGALIGIICISSDSRPFQDLKIPLSIGSKQQDADSSMVRSRVPVSVKLGLDPQQPLQVAIASKLSNLASKVSNKVKSKIRTGENSLDREGGSGDGYHSDHGHPDAVLCDNRDDANSSGASTPRGDSTAHGAFSQVEEKLSGRLVRDSSDEGKGKPTIQKILSSKAEEWIAKKGLSWPWKGTEQEGGSETRAARFVWPWVQIDQEAEPANHKSSSISGKLEMQQNDGHRAVNNEASGSWSSININSTSSVSSCGSTSSSAVNKVESDMDCLDHEILWEDLTIGEQIGQGSCGTVYHALWYGSDVAVKVFSKQEYSDDVILSFKQEVSLMKKLRHPNILLFMGVVTSPQRLCIVTEFLPRGSLFRLLQRNTGKLDWRRRVHMALDIARGMNYLHHCNPPIIHRDLKSSNLLIDKNWTVKVGDFGLSRLKHETYLTTKTGKGTPQWMAPEVLRNEPSDEKSDIYSFGVILWELATEKIPWENLNSMQVIGAVGFMNQRLEIPKDVDPQWISIIESCWHSEPSNRPSFQVLIEKLRDLQRKYTIQLQAARSGGDNSNSNNNNIPQKET

>Csa6M154510.1

MEDQRDDVAPSEQAPSNASSWWSSDFEDKFGSVSLGPREDIVNEKEEIINSDQDVLFSPQTASQILWRTGMLCEPIPDGFYSVILDKRLKDRFHSIPSLDELRALEVEGYRNDVILVETEKDKKLSMLKQLILTLVKGLNSNPAAIIKKIAGLVSDFYKRPILESPAKGALEETSHLFEDRGIQLLGQIKFGSCRPRAILFKALADTVGLESRLMVGLPNEGATGCVDSYKHMSVTVVLNSVELVVDLMRFPGQLLPRSTKAIFMTHISAAGESDSAENDSCDSPLEPNSPLYGFSERVDPDSVEKDESLQFHRKFDATSNAHGNSLRNMMLRSSTALDRKLSLSHSEPNIANAFWRRSRRKDIAEQRTASSSPEHPSFRARGRSMLSGDRKAFRDFSDDVSTSRSDGASTSTSEARRLRRRSISITPEIGDDIVRAVRAMNETLKQNRLLRGQEDDRSFSHPSNERNSSSDVRRNDQVGSQRAISLPSSPHVYRGQTSDGIGHSAYGNDELTFKWTKVLESFSLNDKPLLPYPEWNIDYSELTVGIRIGIGFFGEVFRGIWNGTDVAIKVFLEQDLTPENIEDFCNEISILSRLRHPNVILFLGACTKPPRLSMITEYMEMGSLYSLIHLSGQKKKLSWRRRLKMLRDICRGLMCIHRMKIAHRDLKSANCLVNKHWTVKICDFGLSRILTDAPARGSPSAGTPEWMAPELFRNEPFTEKCDIFSLGVIMWELCTLNRPWEGVPPERVVYAVGTEGSRLEIPEGPLGRLISDCWAEPNERPSCEEILSRLLDCEYSLS

>Csa3M133150.1

MAREVHGVLSQQLYMERPSVVSDVRITADHSVSDVCVQTGEVFSPQFMRDRVALRRFSDMSDGDQQQQQQKRKGFGFNPSNQLVYEDLSGILGLKRMNSESSSEMSSTPMTAYAAEKDNKVYPNTTSKCQWEYNNGTGQASAAYADETNRGVQIGPMMSALYPLDSPHSCYPCGAGFGDFSANDKMKFLCSFGGRILPRPNDGKLRYVAGETRIISIRKNISYEELTKKTYAVCKYAHTIKYQLPGEDLDSLISVCSDEDLHHMIEEYQELENAEGSQRLRIFLISSNDCSESPTSIEGRVVPPIDVDYQYVAAVNGILDPSLQRSSSGQSFTSQNSQVGAISDHSPNFRTDSSHATDVKDVSSLMPNLMGMLPRPGGQLLNPIQVPRKSLNQSPLISPVTVMQKDFRNVDATYAEDARNFSPIVSGKHPCDSVYYVDAMGRHNYLYHGSPLMNYYHEKSTAETDETYKVLNVHFPRSSSEDFVPAPIWGLSDTHPMKTMLKERAVNYEQLCSDAEYLMQLRSGTTHMGQRIIHSHSEPLLLEQDQKPNHGGPYPLTSFNDSDQSPSLAMSSSLQDLPTLWKQRDGVEFQYAKYENHRKLASGSDNETYEECNFDGKKNNFNGIIYAPSLNDEEKYRYLQHAGYRQNGCPPKEVQNLRGRSSAERGIELENSADTTGAPSLVYHFERTAPKDFEESQYSTKDQPTTSDIVRSQPLSCTSSDLLPHTIQAFDDVKIINQKPTWDSSASGIEISLGDENFVTCHYCKVAAHSRRKSNCDDAISHSDDSHGNEDEDLAVIVEDVTHSLPPDIPLASGIVPRVENEASDEFPSSRGNDALSSSSETDHEDADSILSSRDESMSEAAIAEIEAGIYGLQIIKDADLEELQELGSGTFGTVFHGKWRGTDVAIKRIKKSCFSGSFSEQERLTRDFWREARILSTLHHPNVLAFYGVVPDGPDGTLATVTEYMVNGSLRHVLLRKDKVLDRRKRLIIAMDAAFGMEYLHLKNIVHFDLKCDNLLVNLRDPERPICKVGDFGLSRIKRNTLVSGGVRGTLPWMAPELLDSTSSKVSEKVDVFSFGIAMWEILTGEEPYANMHCGAIIGGIVSNTLRPPIPKRCDPEWKKLMEECWSPEPAARPSFTEITNRLRSMSVALQIRKRPNVASR

>Csa6M136540.1

MEQSRNDQFQCTPMHYGFGELQPASKSSIENRVSIVNMQSDGRTMDLRMSEVKPVLNYSIQTGEEFSFEFMRDRANPRKPLVSDSVSDPSCASRYMDLKGILGLSRTGSECGSDNSMIISMEKGSKDFERTNSSLHGGDRNNLGSAHQKSPELSRYDSGRAIGHGYASSGTSDGSSAKMKVLCSFGGKILPRPSDSKLRYVGGETRIIQIKMDISWQELMRKTSSIYNETYAIKYQLPGEELDALVSVSCDEDLQNMMEECNEFKNDKGSKKLRIFLFSMSDLDEGHFSMGNVDNDSEIQYVVAVNGMDRKNSNLHGLSSFSANNLDEVDGQSIERGTVLKDLVGVNASALTANVASSSLQSSQPVRASASNAYETFLQAYHEPQGQNSEIPSTQLKGKFKDSFEKETHDASGCSSNPSHFFDGNLMTSDKKSTPVSIAQGEFPFLTHKNETELQSSEGLSSMLASGNPIVSRSNDMDNIIHNMPSNAYPHGHTDSESKIVDLSLLEPPAVAQRVYYSERIPREQEELLNRLSKSDDSYGSQFLISHSQSDQDQIPDSAVKLQDSSNYESENSIPMEKSSHNATKVRNDELSHIQDGRNVNEAVSGRNWNISHDGDTELKLQNNFDVTLDSKVDGVVKAGKDLNCPVNNNEKLAGPKLSRPESELPALGQVSSLKNHEDSALDLLQLNLGEVVGMRCTDDNSLKQTQLAYKEESLINHVNERPSTGNVSKPVQGDIVIDIDDRFSRDFLSDIFSKAIPFENSLDSRSQLHNDGTGLSPDVDNHEHKGWSYVHDLAQEKYVQNDVSLIDQDHIVFPSAPKTAGDDFTPLTTILREDSQLNFGDDQKVHRISGNDATNFLSRCDHSRMNGIDSSQFDAMMENLKTLEYRHENVKVASKDSGLPPNDPSLGNFDPNSLQIIMNDDLEELKELGSGTFGTVYHGKWRGTDVAIKRIKKTCFMGRSSELERLTVEFWREADILSKLHHPNVVAFYGVVQDGPGGTLATVTEYMVDGSLRHVLLSKDRHLDRRKRLIIAMDAAFGMEYLHSKNIVHFDLKCDNLLVNLKDSQRPICKVADFGLSKIKRNTLVSGGVRGTLPWMAPELLNGSSNKVSEKVDVFSFGIVLWEILTGEEPYANMHYGAIIGGIVNNTLRPTIPSYCDSEWRRLMEHCWAPNPTDRPSFTEVAGRLRVMSTSASSQTKAQGPKIARS

>Csa6M511830.1

MATSCFGSLRIRKSKGKTLSTPSSLKSQMNSEMENMERRRFDSLESWSMILESENVETWETSKEDQEEWTADLSQLFIGNKFASGAHSRIYRGIYKQRAVAVKMVRIPNQKEETRAKLEQQFKSEVALLSRLFHPNIVQFIAACKKPPVYCIITEYMSQGTLRMYLNKKEPYSLSTETILRLALDISRGMEYLHSQGVIHRDLKSNNLLLNDEMRVKVADFGTSCLETQCRESKGNMGTYRWMAPEMIKEKPYTRKVDVYSFGIVLWELTTALLPFQGMTPVQAAFAVAEKNERPPLPASCQPALAHLIKRCWAANPSKRPDFSDIVAALEKYDECVKEGLPLAHHRRLVNKNAIIERLKACA

>Csa1M046040.1

MRNLNWFKPISINGKPGRRLSLGEYQRAVSWSKYLVSSGAEIKGEGEEEWSADMSQLFIGFKFATGRHSRIYRGVYKQRDVAIKLISQPEEDENLANFLENQFISEVALLFRLRHPNIITFIAACKKPPVFCIITEYMTGGSLRKYLHQQEPHSVPLNLVLKLALDISRGMQYLHSQGILHRDLKSENLLLGEDMCVKVADFGISCLESQCGSAKGFTGTYRWMAPEMIKEKHHTKKVDVYSFGIVLWELLTALTPFDNLTPEQAAFAVCQKNARPPLPSACPQAFRHLIKRCWSKKPDKRPHFDEIVSILETYVESYNEDPEFFCHYVPSSSRYIAWKCLPKCITKQSSASLKPRNSSSS

>Csa2M070870.1

MLEGGQKFPGMIDLNEHAYDLSQGFYHKLGEGTNMSIDSFASLQTSNDGGSVAMSLDNSSVGSNESHTRILNHQGLRRRANDNHTFQHSVNRRGRVTHHLSDDALARALFDSNTPTQGLENFEKWTLDLRKLNMGEAFAQGAFGKLYRGTYDGEDVAIKILERPENDLEKAQLMEQQYQQEVMMLATLKHPNIVRFIGSCHKPMVWCIVTEYAKGGSVRQFLMRRQSRSVPLKLAVKQALDVARGMEYVHGLGLIHRDLKSDNLLIFADKSIKVADFGVARIEVQTEGMTPETGTYRWMAPEMIQHRPYTQKVDLYSFGIVLWELITGMLPFQNMTAVQAAFAVVNKGVRPIIPNDCLPVLSDIMTRCWDPNPDVRPSFTEVVRMLENAQTEIMTTVRKARFRCCITQPMTTD

>Csa1M057040.1

MERNVKKSTLDQPSNYEQIRLTSMEGRNQGLGSTNQRTFHDPSSNISTNIRPPEYNMLVVGVASPGHNYSIQTGEEFALEFMRERVNAKHHFVPTNSPDPGVSTGYMDLKGMLGIPHASSESGSSIAMLNPVEKDHVQHFERGSLPHEEKSSYNSMRFVPRASSRNDVSRLHSFTSSGASDSTSRKVKFLCSFGGKVMPRPSDGKLRYVGGETRIIRITKDISWSNLLQKTSTIYDQVHTIKYQLPGEDLDALVSVSCDEDLQNMMEECNIPENGGSTKPRMFLFSISDLEDSQMGVGSAEGGSEIEYVIAVNGMDLSSRRNSTPLGNTSGNNLDELLALNVGLESGQVAPLSDNMKSSLTITPSFPQSSQTIWTNSSSGLKSSLQPLSGQKLQQGELGPPQPSSFRPMQSFPEKLGKTSVSSSIQSQHDYVLNTNATSVENVPPMPSKGYLNQHYPVSGFHTQDPDSSSREGKITEISTSKLSEPDEIQSLEKEVSFNDAQMKRESSLHKIDEANESPNFEHECGVSSNLNDASVLNYNTKGMQVINSDTDVGSSLLLTKNNKHQDPAPESVSLEASNEGNRGTKEDKFSSDELPTSGFGASKADETGFSYLEPILPQRVFHSERIPREQAELNRLSKSDDSFGSQFLRTQGNSDYSQTIIESAETLLDGNMTLESEQFVSSSKLPCGNHQTIEDGLEPFEKYKTSADKNSKTMNISGEHDGSEVSDMSNIKSPSACRKEAEGLAHLTAGEEVPDKHKEESLMGPLESGWIEGSTHNNHGNETQEQPEPSSLTENPGKNATQVEPGVGIGTSEHGDILIDINDRFPRDFLSDIFSKARNSENISGINPLHGNGAGLSVNVENHEPKRWSYFRNLAQEEFVGRDVSLMDQDHLGFSSSLGNVEEGGTVNRFPLLNSDVGAIYEKESHNFDDNIQPESRLLTGPSTTNLYTEYNSSQLKGNETMHEPSSKSPQDENVDAKLDGQDIGVPLVDFYLKDFDISTLQIIKNEDLEEQRELGSGTFGTVYHGKWRGTDVAIKRIKKSCFTCRSSEQERLTIEFWREAEILSKLHHPNVVAFYGVVQDGPGGTLATVTEFMVNGSLRNVLLSKERYLDRRKRLIIAMDAAFGMEYLHSKNIVHFDLKCDNLLVNLKDPFRPICKVGDFGLSKIKRNTLVTGGVRGTLPWMAPELLNGSSSKVSEKVDVFSFGIVLWEILTGEEPYANMHYGAIIGGIVNNTLRPPVPSFCDPDWRLLMEQCWSPDPVARPSFTDIARRLRVMSTAAQTRSPQNQMPK

>Csa7M051390.1

MDNITAQLKRGISRQFSTGSLRRTLSRQFTRQSSLDPRRNNLRFSFGRQSSLDPIRRCPDEDNELSIPDNLDSTMQLLFMACRGDVRGVEDLLNDGTDVNSIDLDGRTALHIAACEGHAAVVKLLLSRKANIDARDRWGSTAAADAKYYGNTEIYNILKARGAKVPKFRKTPMTVANPREVPEYELNPLELQIRRSDGISKGAYQVAKWNGTKVSVKILDKDCYCNPDSINAFKHELTLLEKVRHPNVVQFVGAVTQNLPMMIVSEYHPKGDLGCYLQKKGRLSPSKALRFALDVARGMNYLHECKPDPIIHCDLKPKNILLDNGGQLKVAGFGLIRLSKMSQDKAKLAHPVVIDYSNLYLAPEIYNNEIFDRSVDSFSFGLILYEMVEGIQPFHPKPPEEVTRAICAEGKRPPFKIKSKSYPPDLKELIEECWDPEPVMRPTFSEIIVRLDKIVANCSKQGWWKDTFKLPWK

>Csa1M467120.1

METNNSNNNGVRFLLGKQSSMAPDRQPEEAELAEDGEEIDPGVRLMYLANEGDLEGIKELLDSGIDVNFHDIDNRTALHIAACQGCNEIVDLLLRRGAEIDPKDRWGSTPLADAIFYKNHEVIKLLEKRGAKHLMAPMHVKHAREVPEYEIDPKEFDFTNSVNLTKGTFHLASWRGIQVAVKELPEDVISEEDKVNAFRDELALLQKIRHPNVVQFLGAVTQSSPMMIVTEYLPKGDLCQLLHKKGPLKPIVAVKFALDIARGMNYLHENKPAPIIHRDLEPSNILRDDTGNLKVADFGVSKLLTVKEDKPLTCQDTACRYVAPEVFKNNGYDTKVDVFSFALILQEMIEGQPPFSNKKENAICKGYAAGMRPPFKAPAKCYAHGIKELIEACWDERPSKRPTFRQIITRLETIHHSLSHRRRWKLPTLRCFQDPDAKIRRDHLSSSRSLSSRSASSI

>Csa3M002480.1

MPIEDDVESCGSRATDFSSSHVNPRHHRQKLEVYNEVLRRIQQSNFHEANLPGFDDQLWLHFNRLPARYALDVNVDRAEDVLTHKRLLQLAVDPSNRPVFEIRSVQVYPSANENFIDSSCLDASMMEDAQSSLNYSNRQGNHPPPTFGSSPNLEAPTFQGSKYGVEDRDSAPNVTSSFSRPMHEITFATSDKPKLLSQETEELKRVLEKEILNFKEQCWSEKQPSSALGKHNQNRVESFPSCVGIPTDGTDVWEMDISQLKFENKVGSGSFGDLYRGTYCSQEVAIKVLRPERINEEMLKEFSQEVYIMRKVRHKNVVQFLGACTKPPNLCIVTEFMSRGSVYDFLHKQRGVFNLPSLLKVAINISRGMNYLHQNNIIHRDLKTANLLMDENMVVKVADFGVARVQTQSGVMTAETGTYRWMAPEVIEHKPYDHKADVFSFGIALWELLTGEIPYSSMTPLQAAVGVVQKRLRPTIPKNAHPVLAELLERCWRHDPTERPNFSEILEILKQIAEQVDNSGENRRKKDKLSGALFSAFKKRHH

>Csa7M017160.1

MVMEDNESCGSRAYDLLSPAQSRQQRQKFEVYNEVLRRLKDSNNEEAIQPGFDDELWVHFNRLPTRYALDVNVERAEDVLMHKRLLQFAHDPANRPAIEVRLVQVQAVSDEHSADFADSCPVKDTDHNSSNCLSRQSMHPPPAFGSSPNLEALALEANNTQDLEVDQSVHARTQFFRPMHEITFSTDDKPKLLSQETERLKTALESEVLLVERRGWPNQKSSSPVGELDITAKCESDRVEIPTDGTDVWEINPRHLKFEHKVASGSYGDLYKGTYCSQEVAIKVLKTERVNTDMQSEFAQEVYIMRKVRHKNVVQFIGACTKPPSLCIVTEFMSGGSVYDYLHKQKGTFRLPSLLKVAIDVSKGMNYLHQNNIIHRDLKAANLLMDENEVVKVADFGVARVKAQSGVMTAETGTYRWMAPEVIEHKPYDHKADVFSFGIVLWELLTGKLPYEFLTPLQAAVGVVQKGLRPTMPKHTNPKLADLLEKCWQQDPSCRPDFCEIIDILLQITKEVAEEGEDRRKEKSGGFLSVLRRNHH

>Csa6M058190.1

MADMDFVEGVGESSSPPRSFAAGFCPAHDVRNDVYTRLVECGHEEAVSNPQFRENLDAHFNRLPPSYGLDVNMEKVEDVLLHQKLLSLAKDPEKCPVYHIRFLEHISTKSDGNDDHVFLDSILLSGSSNEAADRRLPLSHKRTRGNIIDFEACSKLEGLNLDVRKNSKPMDRRPGNIGHVLIHEVIFSTVDKPKLLSQLSALLSDIGLNIREAHVFSTTDGYSLDVFVVDGWPIEETDGLYEAMEKAVARYEGSWSGSSHSHPVVKKTLDAQVKSADWEIDRRLLKIGERIASGSCGDLYHGFYLGQDVAVKILRSEDLNADLEDEFNQEVTILRKVQHKNIVRFVGACTSSPHLCIVTEYMPGGSLYDYLHKNHCVLKLLQLLKFSIDVCEGMEYLHLNNIIHRDLKTANLLMDTQQVVKVADFGVARYQSQGVMTAETGTYRWMAPEVINHLPYDQKADIFSFAIVLWELVTAKVPYDSMTPLQAALGVRQGLRPDLPKNVHPKLLDMMQRCWDAEPVNRPPFTEIKVELKSLLEEVEMCTKYEENCAAVNGS

>Csa3M728150.1

MSCNEKNRGVNDRETEHPVLTKPHQKPVIQNGSITAQHLTIDNNLLVDPKLLFIGSKIGEGAHGKVYEGRYRNEIVAIKVLHRGSTPEERAALESRFAREVNMMSRVKHENLVKFIGACKEPLMVIVTELLPGMSLRKYLMNNRKQQLDPRMAINFALDVARAMDCLHANGIIHRDLKPDNLLLTANQRSVKLADFGLAREESVTEMMTAETGTYRWMAPELYSTVTLRQGEKKHYNNKVDVYSFGIVLWELLTNRMPFEGMSNLQAAYAAAFKQERPSIPGDISPELAFIVQSCWVEDPNMRPSFSQIIRMLNAYLFTLPPPSQSSPSSPKSDTTETATTSNSAITEFSSRARGKFGFLRQLFAAKRAKNSQ

>Csa1M003510.1

MEAGSRFYSATDEFRLEAKWLVDPKHLFVGPRIGEGAHAKVYEGKYKNQTVAIKIVHKGETVDEVAKKEARFAREVAMLSRVQHKNLVKFIGACKEPVMVIVTELLLGGTLRKYLLNMRPRCLDTRVAVGFALDIARAMECLHSHGIIHRDLKPENLLLTADHKTVKLADFGLAREESLTEMMTAETGTYRWMAPELYSTVTLRQGEKKHYNHKVDAYSFAIVLWELLHNKLPFEGMSNLQAAYAAAFKNVRPSAENLPEELAIILTSCWQEDANARPNFSQIIQMLLNYLYTISPPEPVIPSRIFTSENTVFPPESPGTSSLMAVRDDSGDTPKAKMENNPRGCFFCSNDCY

>Csa2M049880.1

MFRYNHLHRASGLAILQCKDVFLIPYNVPNGFGKTEEFGSLKTEKLGSCFLDGYKFLMDSPLTRSSTPNSSPISNPGSHDENPRVKFLCSFLGSIMPRPQDGKLRYVGGETRIVSVPRDITYEELMVKMRELYDGAAVLKYQQPDEDPDALVSVVNDDDVINMMEEYDKVGSGDGFTRLRIFLFSHPEQDASLPFVDGDERDTERRYVDALNNSNDMNDFVRQQQQNSPALSGIDDMHGTEHFLNPMNIEGSLHTQRSCEPLSQYHLHQLTIPHVGSGGQQQSVAQRYSEMEAPWSPALLSPRHHGPYDSRPMGDYPSSPFARYRMPFPDLPDKYLERMPEDYVRQQMNHQHMYEHQPQYNENIVWLPNGTINEESGFPGNILHGHGVPDGNSSCEHCRANFHRYQAHMEQVNTLNGLPLEYTQNREALMQKADTKFHHGIFPNEQNINDHRSAYNETPPHEKGWIMQHQMSVRGDDTRTHVSGTGRLTDHYIVDGSGSNLPSTQSNVADGYHASTNFHDEVFRDQVVPSGQHMCVPPPEDRGVGYMPYGYGGEPHYPPMAQRHMPGNASWRNVQNPLHVAPPYEASVFHQQGNASINPGYIKAMQDGSPRIHMGVDHQNPWHESSQKVLGVDGATGTEHLPAHVLKTNSTTVGHDNQQFTSLEHIQPHLDKINLVASPMQRSDSSSAFIQEKMVAPFHPSQNPQLRAVSAVNEAMMMERKVVHGEGNGHMIKDMGKPDISEAHTASHPGQNNTDDTYSKVAPLELLNSTCTNSAVENGDGLKPSVETLEKPKLSVSRLSFLPELIASVKRAALEVSEETMVEETALRRPDSIEKKETTNEQHSSNNHVEPELETESENQSSRIEPTKAEEEAISRGLQTIKNDDLEEIRELGSGTYGAVYHGKWRGSDVAIKRIKASCFAGRPSERERLIADFWKEALILSSLHHPNVVSFYGIVRDGPDGSLATVTEFMVNGSLKQFLHKKDRTIDRRKRLIIAMDAAFGMEYLHGKNIVHFDLKCENLLVNMRDPQRPVCKIGDLGLSKVRQHTLVSGGVRGTLPWMAPELLSGKSNMVTEKIDVYSFGIVMWELLTGDEPYSDMHCASIIGGIVNNCLRPEIPTWCDPEWKALMSSCWDSDPAKRPSFSEISQKLRNMAAAMNRLHCWKTTSTYMKEIHVVIGMISLGILPLEAYALQ

>Csa6M517390.1

MDEEANSWLRRTKFSHTVYHRWDSLKLNPVPFIVEPPRNSGLKSRPPSASSAQKPNPDISKIQRSFISNKQRSLSPLPESNLSEVFKEAKSESKRFSTPTPRLRERTKEFKNKLFNKDPQDSKSSNSKSSLNTSPLKQLSSGKGGDKSKLKKDSSWTKYFDSGKVTAVETADDWTVDLSKLFVGLRFAHGAHSRLYHGKYNDEPVAVKIIRVPDDDENGTLAARLEKQFTREVTLLSRLYHPNVIKFVAACRNPPVYCVITEYLSQGSLRAYLHKLEHQSLPLQKLIKFALDVARGMEYLHSQGVIHRDLKPENVLIDEDMHLKIADFGIACPEAFFDPLADDPGTYRWMAPEMIKHKPCSRKVDVYSFGLMLWEMVSGAIPYEDMTPIQAAFAVVNKNLRPVISSDCPLAMRALIEQCWSLQPDKRPDFWQIVKVLEQFESSLARDGTLNLLGNPLSSFHDHKKGLLHWIQKLGPLHPEVSSSPVPKPKFS

>Csa1M074900.1

MEEDPNSWLRRTNFSHTICYRLESLSLASFPVTTQPRPKSLVQSKPNPRYNLTKQRSLSPSPQTNLSNAFKDARINQKRFSTPQPQRKEPLKEKSKRLFCKRAKVQNSLKEEKLKGPLRNLVSFKGCEKFKFKESSWSKLFEHGGGKVTAVEAVDELSIDLSKLMFGHRFAFGAHSRLYHGIYEDKVVAAKMINLPANDENGDLAGRLVKQFGREVTLLSRLHHPNVIKLVAAVKKPPVYCIITEYLPQGSLRAYLHKLEKKSLPLQKQIAIALDIARGMEYIHSQGVIHRDLKPENILIDQDFCLKIADFGIACEEAHCDTLAEDPGTFRWMAPEMIKRKPYGRKVDIYSFGLLLWELVAGKIPYEDMTPIQAAFAVVDKNIRPVIPSECPPVIRVLIEQCWCEKPEKRVEFWQVVKVLEQVESCIGGDGTLMTSVELKGKASWEDHKKGLKHWIQKLGPLNSHNSLNSSRSKFI

>Csa6M502000.1

MSDHSDEQHSFRTITTAFIPFIGDDDSDDSGSDFVFSIEPTLLIDPHCLKIGEVIGEGSCSIVYEGLYDYQPVAVKIIQPIRASAISPEKKERFQREVTLLARLNHENIIKFIGASIEPTLMIITELMRGGTLQKYLWSIRPETPDSKFSLSLALDLSRVMTYLHSNGIIYRDLKPSNLLLTEDKQRIKLANFGLAREEISGEMTTEAGTYRWMAPELFSIDPLPVGCKKCYDHKADVYSFSIILWELLTNKTPFKGRNDIMVAYAVAKNIRPCLEEIPEDMAPLLQSCWAEDPNSRPEFTEVTDYLSNLLQSFVLKESSLPNMDDKTEEKEEEEKVKCRSNTSFSQRKREPKAGRYRNSSFCFKCCHNSCLSD

>Csa3M836460.1

MKEKSETGGGYVRADQIDLKSLDEQLQRHLSKAWTMEKNKRREDEEGVGIGGGGGGGGGGGGVGGGRPAITRQEWEIDPSKLIIKAVIARGTFGTVHRGVYDGQDVAVKLLDWGEEGHRSEAEIASLRAAFTQEVAVWHKLDHPNVTKFIGATIGSSDLHIQTENGQIGMPSNICCVVVEYCPGGALKSYLIKNRRKKLAFKVVVQLALDLARGLSYLHSQKIVHRDVKTENMLLDKTRTVKIADFGVARVEASNPNDMTGETGTLGYMAPEVLNGNPYNRKCDVYSFGICLWEIYCCDMPYPDLSFSEVTSAVVRQNLRPEIPRCCPSSLANVMKRCWDANPDKRPEMDEVVTMLEAIDTSKGGGMIPLDQSQGCFCFRRYRGP

>Csa7M387170.1

MDLANGGEAGKALVANQVADIVSGKSRNTQENDLGSKLGTGSKSNRDMVFRADKIDFKSWDIQLEKHLSRAWSRDREVPAKKEEWEIDLSKLDIRYVKAHGTYGTIYRGNYDGNDVAVKVLDWGEDGVSSVAEIAALRTSFRQEVAVWHKLDHPNVAKFYGASMGTSNLKIPPKSSSFDSNQTFPSRACCVVVEYLPGGTLKSFLIKNRKRKLAFKVVIQLALDLSRGLSYLHSKKIVHRDIKTENVLLDAQKTLKIVDFGVARVEAQNPKDMTGETGTLGYMAPEVLDGKPYNRKCDVYSLGICLWETYCCDMPYPDLSFAEVSTAVVRQNLRPDIPRCCPSSFANIMKRCWDANPEKRPDMDEVVKLLEAIDTSKGGGMITEDQISCFCFRPARGP

>Csa3M146410.1

MDSKGNVMGGAAIPKETQNQDRTPNSKVAGMGSISSKDMIFRADMIDLKTLDIQLEKHLSRVWSKSIDNQMPKEPWEIDLSKLDMIKQIAQGTYGTVYRGKYDNQEVAVKILDWGEEGLATMAETAALRASFRQEVAVWHKLDHPNVTKFIGASMGATNLKIPMDGQNSFPSRACCVVVEYVPSGTLKDHLIRYWTKKLAIKAVVKLALDLSRGLSYLHSKKIVHRDVKTENMLMDINDNVKIADFGVARVEAQNPRDMTGATGTLGYMAPEVLQGKPYNRSCDVYSFGICLWEIYCCDMPYADLSFADVSSAVVRHNLRPSIPRCCPSSLANVMKKCWDANPEKRPEMHEVVRMLEAIDTSKGGGMINPDDIKCFCLGPFGKLRGL

>Csa3M146410.2

MDSKGNVMGGAAIPKETQNQDRTPNSKVAGMGSISSKDMIFRADMIDLKTLDIQLEKHLSRVWSKSIDNQMPKEPWEIDLSKLDMIKQIAQGTYGTVYRGKYDNQEVAVKILDWGEEGLATMAETAALRASFRQEVAVWHKLDHPNVTKFIGASMGATNLKIPMDGQNSFPSRACCVVVEYVPSGTLKDHLIRYWTKKLAIKAVVKLALDLSRGLSYLHSKKIVHRDVKTENMLMDINDNVKIADFGVARVEAQNPRDMTGATGTLGYMAPEVLQGKPYNRSCDVYSFGICLWEIYCCDMPYADLSFADVSSAVVRHNLRPSIPRCCPSSLANVMKKCWDANPEKRPEMHEVVRMLEAIDTSKGGGMINPDDIKCFCLGPFGKLRGL

>Csa3M840390.1

MGSGNGFYSTSEFNLDAKWLIDPKHLFVGPKIGEGAHAKVYEGKYKNQIVAIKMVGKGDTPERMARREARFAREVAMLSKVRHKNLVKFIGACKEPMMVIVTELLTGGTLRKFLLNLRPRSLELDVAIGFALDIARAMECLHSHGIIHRDLKPENLILTADHKTIKLADFGLAREESVTEMMTAETGTYRWMAPELYSTVTLKHGDKKHYNHKVDAYSFAIVLWELILNRLPFEGMSNLQAAYAAAFKNMRPSAENLPEDLALIVTSCWREDPNTRPNFSQIIQMLLQSLSRISPRSPVIPPRICASENVVMPPESPCTNSLMAVRHGSGEAPHGMIEETPTTSFFCFNKCY

>Csa3M840390.2

MVGKGDTPERMARREARFAREVAMLSKVRHKNLVKFIGACKEPMMVIVTELLTGGTLRKFLLNLRPRSLELDVAIGFALDIARAMECLHSHGIIHRDLKPENLILTADHKTIKLADFGLAREESVTEMMTAETGTYRWMAPELYSTVTLKHGDKKHYNHKVDAYSFAIVLWELILNRLPFEGMSNLQAAYAAAFKNMRPSAENLPEDLALIVTSCWREDPNTRPNFSQIIQMLLQSLSRISPRSPVIPPRICASENVVMPPESPCTNSLMAVRHGSGEAPHGMIEETPTTSFFCFNKCY

>Csa5M523010.1

MGSGNELCSQEFDLDAKWLVDPKQIFVGPRIGEGAHGKVHKGKYKDQNVAIKIIRKGEAPEEIAKTEARFAREVAMLSKVQHKNLAKFIGACKEPIMVIVTELLSGGTLRKYLLSIRPRCLDFSEAVGFALDIARAMDCLHSHGIIHRDLKPENLILTADHKTVKLADFGLAREESVTEMMTAETGTYRWMAPELYSTVTLRNGEKKHYNHKVDVYSFGIVFWEIIQNKLPFEGMSNLQAAYAAAFKNLRPSAENLPADLAPIVTSCWKEDPNDRPNFNQIIQMLFKCLSTIPQPEYVPPPTMHPPDNAVLPPESPGTSSLMATTRHGTGEVMNSEIGEKPTGLFSCFAGNCY

>Csa6M520410.1

MTSPVKFKLGKQSSLAPDGDVHLEDLEELVKQHQTEEGIDSRVRLMYLANEGDLEGINEVLDSGVDVNFRDIDNRTALHIAACQGFADVVALLLERGAEVDSKDRWGSTPLRDAIHYKNHDVIKLLEKHGAKPPVAPMLVKNAREVPDYEIDPKELDFTNSVNITKGTFRRASWRGTEVAVKELGEDLFTDEEKVRAFRDELALLQKIRHPNVVQFLGAVTQSWPMMIVTEYLPKGDLGALLSRKREIKTMSVVRLALDIARGMNYLHENKPAPIIHRNLEPSNILRDDSGHLKVADFGVSKLLTVKEDKFSTCSETSRRYQAPEVFKNEEYDTKVDVFSFALILQEMLEGCSPFPDKADSEVPKLYAAGERPPFGALIKRYANGLKELIEECWNEKPNKRPTFRQIITQLEFIYNRFCHKRRWKVRPLKCFQNIEAMLKKDRLRRSSFNLSSHSSASKFASFWDEKHGNQQYQIHMGNRIFAMFVHHMAFRNSKLLILTIMDLFFPCHCLVNIVNRSVTVRPTFNVTVRPTFNIVKITHLFVKNQKNMQQSKQPSLKSTALRSGGSSFMYFSRTQGKPLGREGAQCLYGLKDNLSYERYRAQAWWKGKSMSVKQSFFLAQNCYSRALATQAIAYLQQGKLQIKGMFQNQEEKMSDSPQRKMGRGKIEIKRIENTTNRQVTFCKRRNGLLKKAYELSVLCDAEVALIVFSSRGRLYEYANNSVKATIDRYKKASSDSSNTGSTSEANTQFYQQEAAKLRVQIGNLQNSNRNMLGESLSSLTAKDLKGLETKLEKGISRIRSKKNELLFAEIEYMRKREIDLHNNNQMLRAKIAESERNVNMMGGEFELMQSHPYDPRDFFQVNGLQHNHQYPRQDNMALQLV

>Csa6M520410.2

MMIVTEYLPKGDLGALLSRKREIKTMSVVRLALDIARGMNYLHENKPAPIIHRNLEPSNILRDDSGHLKVADFGVSKLLTVKEDKFSTCSETSRRYQAPEVFKNEEYDTKVDVFSFALILQEMLEGCSPFPDKADSEVPKLYAAGERPPFGALIKRYANGLKELIEECWNEKPNKRPTFRQIITQLEFIYNRFCHKRRWKVRPLKCFQNIEAMLKKDRLRRSSFNLSSHSSASKFASFWDEKHGNQQYQIHMGNRIFAMFVHHMAFRNSKLLILTIMDLFFPCHCLVNIVNRSVTVRPTFNVTVRPTFNIVKITHLFVKNQKNMQQSKQPSLKSTALRSGGSSFMYFSRTQGKPLGREGAQCLYGLKDNLSYERYRAQAWWKGKSMSVKQSFFLAQNCYSRALATQAIAYLQQGKLQIKGMFQNQEEKMSDSPQRKMGRGKIEIKRIENTTNRQVTFCKRRNGLLKKAYELSVLCDAEVALIVFSSRGRLYEYANNSVKATIDRYKKASSDSSNTGSTSEANTQFYQQEAAKLRVQIGNLQNSNRNMLGESLSSLTAKDLKGLETKLEKGISRIRSKKNELLFAEIEYMRKREIDLHNNNQMLRAKIAESERNVNMMGGEFELMQSHPYDPRDFFQVNGLQHNHQYPRQDNMALQLV

>Csa6M520410.3

MTSPVKFKLGKQSSLAPDGDVHLEDLEELVKQHQTEEGIDSRVRLMYLANEGDLEGINEVLDSGVDVNFRDIDNRTALHIAACQGFADVVALLLERGAEVDSKDRWGSTPLRDAIHYKNHDVIKLLEKHGAKPPVAPMLVKNAREVPDYEIDPKELDFTNSVNITKGTFRRASWRGTEVAVKELGEDLFTDEEKVRAFRDELALLQKIRHPNVVQFLGAVTQSWPMMIVTEYLPKGDLGALLSRKREIKTMSVVRLALDIARGMNYLHENKPAPIIHRNLEPSNILRDDSGHLKVADFGVSKLLTVKEDKFSTCSETSRRYQAPEVFKNEEYDTKVDVFSFALILQEMLEGCSPFPDKADSEVPKLYAAGERPPFGALIKRYANGLKELIEECWNEKPNKRPTFRQIITQLEFIYNRFCHKRRWKVRPLKCFQNIEAMLKKDRLRRSSFNLSSHSSASKFASFWDEKHGNQQYQIHMGNRIFAMFVHHMAFRNSKLLILTIMDLFFPCHCLVNIVNRSVTVRPTFNVTVRPTFNIVKITHLFVKNQKNMQQSKQPSLKSTALRSGGSSFMYFSRTQGKPLGREGAQCLYGLKDNLSYERYRAQAWWKGKSMSVKQSFFLAQNCYSRALATQAIAYLQQGKLQIKGMFQNQEEKMSDSPQRKMGRGKIEIKRIENTTNRQVTFCKRRNGLLKKAYELSVLCDAEVALIVFSSRGRLYEYANNSVKATIDRYKKASSDSSNTGSTSEANTQFYQQEAAKLRVQIGNLQNSNRNMLGESLSSLTAKDLKGLETKLEKGISRIRSKKNELLFAEIEYMRKREIDLHNNNQMLRAKIAESERNVNMMGGEFELMQSHPYDPRDFFQVNGLQHNHQYPRQDNMALQLV

>Csa4M332110.1

MAQSNIGYAETDPSGRYGRFREILGKGATKTVYKGFDEVLGIEVAWNQVHLKDVFHSPEELQRLYSEVHLLKNLNHDSIIRFYTYWIDTHRRTFNFITEMFTSGTLREYRQKYRNIDIEAIKNWARQILHGLVYLHGHDPPIIHRDLKCDNVFINGHLGQVKIGDLGLAAILHDSQHAHSVIGTPEFMAPELYDEEYNELVDVYSFGMCMIEMLTLEYPYSECFNPAQIYKKVTSGKLPNAFYEIKDLEAQRFVRKCLENVSKRVSARELLLDPFLAPSNANNASHNEELLSSSLSPEKSIMARRTDLAISGSINPKDDSIFLKVQIKVKNGKSKNVYFAFDILNDTTIDVATEMVKELEIISDWDPLEIAVMIEKEISSLIPDWEEWKLPKIQHQDSFNYEQDHDGDNDNDNDDDDENYATPHPFYYCGSSHGSSSDSLHAFYSSRENPNHYFGGMKDTSNTTEWFREDDTSSCCSLNSFNYSDLSFYSNNEDEYEYDSNIKGREPQYVSTTKQPTRFCPTMKIDSHHLRHKDNKIIPNREVFESRSRSNNSPRLTRVKSMVNLRSETLHRYLVEMLLKKRLFNTVGAMENIGYQKP

>Csa6M212860.1

MFKNTRAGSPSSNLHDCKSEFGYVETDPTCRYGRFEEVLGKGAMKTVYKAIDEFLGIEVAWSQVKLNEVLRSPEDLQRLYSEVHLLSTLKHESIMRFYTSWIDVDKKTFNFITELFTSGTLREYGKKYRRVDIRAIKSWARQILQGLIYLHEHDPPIIHRDLKCDNIFVNGHLGQVKIGDLGLAAILHGSRSAHSVIGTPEFMAPELYEENYNELVDVYSFGMCVLEMLTSEYPYSECSNPAQIYKKVTSGKLPAALYKIQDVDAQRFIKKCLVPVSMRASAKELLADSFLKVDGNRPSSVGRTQNQKPFLNAKEMENFHLSEGLSRTNMTITGKLNPEDDTLFLRVQTADKDGSLRNIYFPFDIVNDTALDVAMEMVKELEISDWEPFEIADMIEGEISALVPNWNRSELTNHSLGFSCAEEDDNVSHHTFRSISSSSQATTLGLISSPRTNQNISNGFSWFPDDTLDDSSSQCSSASGKYSNLNYISSDEYETSMSSVQTDQHNNINKIHNSSRFCPIENRKSKDFLAQLLYKQSQCAIAGSSQGVASGRKDKKGTDGRKLTRNRSLVDVHSQLLHRSLVEEVNRRRLFKTVGAVESIGFQAPCEVSSSKRVSSRQPIGNRSSDVARTRRNEDIRWQDVGRRT

>Csa2M012110.1

MDGVVSDLELDCSEFVEVDPTGRYGRYNEILGKGASKTVYRAFDEYEGIEVAWNQVKLYDFLQSPEDLERLYCEIHLLKTLKHRNIMKFYTSWVDIANRNINFVTEMFTSGTLRQYRLKHKRVNIRAVKHWCRQILRGLHYLHSHEPPVIHRDLKCDNIFVNGNQGEVKIGDLGLAAILRKSHADHCVGTPEFMAPEVYAEAYNELVDIYSFGMCILEMITFEYPYSECTHPAQIYKKVISGKKPDALYKVKDPEVRQFIDKCLATVSYRLSAAELLNDPFLRTDNGEYDLRPVDYGRGLDDVCPLIRQPYLELHRSDSSFCTGYPYDYSFEASSESGYHPIDNGIELFEYCEGEHSEDVDISIKGKMSEDGGIFLRLRIADKEGRIRNIYFPFDVETDTALSVATEMVAELDMTDQDVTRIADMIDGEIASLVPEWRPGPGIEETPRFANQSYCHNCAPSTYNSASNGLMLRNHDGKNSEVAQCCGHRYASMHGRFEEIMYHADEPEHHTAEDAPNVSSHPDGLSYPEIWGHHESRELSSMSSRQSHSDEDYEKTDRPITDTDTKEIIMESKTAPNTRRTLRSLMNSLSFSETPSPPDINEIDVQQEMRWIKAKYQLELSKLRDQQLNLSSKSSSSEDRQQKMENATPRGNHNQILDSSGRDMNRSSTDSHVYINNSCYSTDMPKQRSRNRKAVESSIVDKVVTAKNACNGSLLPSSLHRTISLPVDAVHI

>Csa7M234730.1

MNGATVTNSEPDNNSEYVEVDPTGRYGRYNEVLGKGASKTVYRAFDEYDGIEVAWNQVKLSDFLQTPEDLERLYREIHLLKTLKHNNIMKFYSSWVDIANRNINFVTEMFTSGTLRQYRLKHKKVNIRAVKHWCRQILKGLLYLHSHDPPVIHRDLKCDNIFVNGNQGEVKIGDLGLAAILRKSYVARCVGTPEFMAPEIYEEEYNELVDIYSFGMCILEMVTFEYPYSECTHPAQIYKKVISGKKPDALYKVKDLEVRCFVEKCLATVSTRLSARELLNDPFLQIDGCDSLLRPIDYYSEYDEVNNSLIRGGPFYGTSHGPLDNGYANYFSHEAGNGLDYCPIDNEASEIDLFSCQEDEHLEDVDITIKGRRRDDDDIFLRLRIVDKEGRIRNIYFPFDLENDSASSVANEMVSELDITDQDVKKIADMIDGEIATLVPEWKKGKSLEETPNCSDSNVCHNCSLNSSLLDYVSPHNLAKKNLHILQCSEEHGCASIHGRFEEITYQVEGSEQFNGDENLHRTTGNSSDIHYADIWAQRDGPDVVSPESLEACNEFGASEQPKLEKEESNVNMDDNDHQMEFQTRNSSSSNPSESFVDDHENEIRQELRWLKAKYQMQLRELRDQQLGVKTKSLSLHPISNLTETDNGASVSYLSPNFNEAAKNKTVQTSLSFGKNITSHSPYVAADNILENKTFQDNNVIVDELSSPELIVTAKSFYTGALFPHSLQRATSLPVDAIDF

>Csa1M695390.1

MDTTDSITAFSHLLQPPDFSHYVEIDPTGRYGRYDEILGKGASKTVYRAFDEYEGIEVAWNQVKLCNFLQCPEDLERLYSEIHLLKTLKHKNIMKFYTSWVDTANKNINFVTEMFTSGTLRQYRLKHRRANIRAVKRWCRQILRGLHYLHSQDPPVIHRDLKCDNIFVNGNQGEIKIGDLGLAAILRKSHADHCVGTPEFMAPEVYEEAYNELVDIYSFGMCVLEMVTFEYPYSECNHPVQIYKKVISGKKPAALYKVKDPSMRQFVEKCLAPVSCRLSARELLSDPFLEIDGCESKLKISDSRRELDDFASTIVRPFLEREKRFSSISYSLEGSDEWRYRSVQKEPDGIELFEDNDNDQLVSLDNNIKGKIREDGSIVLRLRITDKEGLIRNIYFPFDTKNDTALTVATEMIAELDITDQDVIKIAEKIDGEISSLVPEWKPGPGIDETPRISYDGGSQSYNACNQPSDNILIENKGNGIKLYQILNLSTDGHALAHEHFEQEQFSLKADRPTQPNVSSQHYQPDSVLNENQALSSHSFRQRHSDDNYKKIDQSLTVGYNKEKLPVNKATVIDTSQRSLLGSRSLSTVSSYCEDKFSSQIHWEIRWLWN

>Csa3M119370.1

METGPASSKDSSPLPFDMECRFRLRSAFDSFCIASEAFSEHRRCRIPIWRHVFCSKNECLADPISALFGSMESPDNAAEKDPTGRYVRYDEILGRGAFKTVYKAFDEVDGIEVAWNQVRIDGFLQSPEDLEKLYSEVHLLKSLKHENIIKFYNSWVDDKKKTVNMITELFTSGSLRQYRKKHKHVDMKAIKNWARQILRGLVYLHSHDPPIIHRDLKGDNIFINGNHGEVKIGDLGLAIVMQQPTARSVIGTPEFMAPELYEEEYNELVDVYSFGMCMLEMVTFEYPYSECKNPAQIFRKVTSGIKPASLAKVSDPRTMEFINKCLVPVHERLSAKELLKDSFLQVENPKESARNPLQLSNQVSKSINLPKSGPISMDIDIDQKIHSLSTYAESNSGSPRFPVVEFQTMNKNNEFRLRGNKNDDNSVALTLRIADSNGRVRNIHFTFYLDSDTALSVAAEMAEQLELINHDVDFIAEFIDFLITKLIPEWKPLSVYSSNGELSLFSAPPFLKSAKSSIGSAWGSILTGSHDGLVAQDISSGLGCGTQKDCLQSEEDGWTTDISAGHIFDTCPSSPSLANFEDLNSHASFALELLVDDCSTKSAKVFDCSNIDGSSKGSSWSIAELEHHGSSYVVEDKFQRNVGDVGIFTPMDYFAKNSVVSMPAPSEASNVMSLTSSCSSLSLTDKDLDAELKMEIDAIETHYRQLFDELSRMREEALEATRRRWIAKKKLIH

>Csa5M148620.1

MWLLRPFLNTFLLWSSLSDSLLFPVTGFSSLFNFFLFLFRLSFSVTLSCGSDLIMSGNSEADDRGIVEKDPSGRYIRYDEILGKGAFKTVYKAFDEVGGIEVAWSQVDIEDVLQSPEQLQRLYSEVHLLKSLKHENIIKFYSYWVDDKHKTINMITELFTSGSLRQYRKKHRKVDLKAFKNWARQILRGLTYLHGHNPPIIHRDLKCDNIFVNGNTGEVKIGDLGLAIVMQQPTARSVIGTPEFMAPELYDEDYNELVDIYSFGMCMLEIVTCEYPYNECKNSAQIFKKVTSGIKPASLEKVLDPQVKQFIEKCLVPASTRLPASELLKDPFLAAESPKDNSSELSRSLNEHFKSVNPPLLGSHPMETDHNCTKLSGSVASSVKSNNGISHFSTQELQRLTENNELTLKGDMTDHNTMSFHLRIAELYGKSRNIHFAFYLDSDTSLAIALEMVEQLELSNEDATIIAKLIDELIAKFVPSWKPCPNYCEEQQQNTPHSPEAQEDKTFISPFFSELVLSSPMVAAARNNLTGLAKVEDQENQQSIISCASVEYIYSTVSDYSIGKGSECGEFGHPDCEKAYISSGTIDLDAEAVGSLSTTIDFAKPSLISSCSEMSKELSLSSFSTLSMEERDHQDELKMEIDAIDLQYHQCLCELSRMREEAIESAKKRWMSKKKATGI

>Csa3M062560.1

MMPSVMTESSDKETEAFVEVDPTCRYGRYPELLGTGAVKKVYRAFDQEEGIEVAWNQVKLRSFSNDPSMIDRLYSEVRLLRSLKNNNIIALYDVWLDKLHGTLNFITEVCTSGNLREYRKKHRQVSLKALKKWSKQILKGLHYLHSNDPCVIHRDLNCSNLFVNGNVGQVKIGDLGLAATVRKNHSAHSVLGTPEFMAPELYEEHYTELVDIYSFGMCLLELVTLEIPYSECDNVAKIYKKVSSGIKPQALGKVKDPEVKAFIENCLAESKVRPSAADLLRHPFFREIDDDENEDDNNDH

>Csa6M110320.1

MDYAPRIKSESSCRLDLTQEKNQLNGTQIEIQVFVSRSVSSEIFPLRHQPQGILSSSSSSSSSSSSNYTASHVNGCLMFDMPAENSIPYDRDAEPFVEVDPTGRFGRYDDLLGSGAVKKVYRAFDQEEGIEVAWNQVRLRNFSGDPVFINRLRSEVQLLSTLNNKYIIVCYSVWNDDEHNTLNFITEVCTSGNLRDYRKKHRHVSIKALKKWSKQVLEGLDYLHTHEPCIIHRDLNCSNIFVNGNIGQVKIGDLGFAAIVGRSHAAHSIIGTPEYMAPELYEEDYTEMVDIYSFAMCLLEMVTMEIPYSECDSVAKIYKKVTTGIKPQAITKVTDAEVRAFIEKCIAQPRARPSASELLKDPFFDEVRDEDSEQTS

>Csa1M046910.1

MSQDLSPDQDLDESDPEFVEIDPTGRYGRYKEILGKGAFKRVYRAFDELEGIEVAWNQVKVTDLLRNSEDLERLYSEVHLLKTLKHKNIIKFYNSWVDTKNENINFITEIFTSGTLRQYRKKHKHVDVRALKKWSRQILEGLLYLHSHDPPVIHRDLKCDNIFVNGNQGEVKIGDLGLAAILQQARSAHSVIGTPEFMAPELYEEEYNELVDIYAFGMCLLELVTFEYPYIECANAAQIYKKVTSGIKPASLAKVTNLGVRAFIEKCIANVSDRLPAKDLLRDPFLQADDDHESISRHLRSKTQPTEKKEQIDFDRSVDYSPAETSRDFSMHGERKDVNKIFLKLRIADSMGNFRNIHFPFDIEADTAISVASEMVEELDLSDQDVSTISEMIETEIRSYIPDWISVEYSGDNVGADAPVSDSSPSETRNVASPLSIESGNLALEVMPSGRKYWSDSPKGIGGCSPIKPGPSNLSFASDQNVESSNSHIHGDNLDHAAIIKGLENELLSEGGDHDGQDESSIHTSSETHHSEENNYDESVDLKIVAEKLENLLTQQQKELDELRKKHKLDISELLTKLTPESYQKVIEMCQLQHPDFELVL

**C: The 2 kb genomic DNA sequences upstream of the initiation codon.**

>Csa2M361890.1

TGAGTGAAGTAAGATTTTTAAAATATGCATTGGGATGAAGAATTGACATTAAACTAGCTCATCATACATCATGGTTCAAATTGGTATTGATATTGTAAAAAGTTAATAAACTATGAAAGATTGATGTTTTTTAGCCATTGGAATCTGCAAGAACTAAGTGAAATTTAATTAACCTTCCCCTAATTTCTACTCACTTGAATCAAACATGTTCATGAATACAATATTAGAATATGATTGAAAATAAAAAGAAACTATATAGTTTTGATATAATTCAGTATTGAGGATCTTCCCAATAAAATTTGTTCCAATTTAAAATTGAAATGGAATAATAATGAGAGCACGAGGAGGGATTAAATTCTTCACAAAGTTGAGGACAAATAAGACATCCCAATGTGGTCAACCACTCCCCCTCATTCCAATATTATATACTTATACTATGGTCCACATTTACTATACACATATATGCCTTCTTCATCTCACATTAACCTCTCCAACAACAATTCTATCTCTTTCTATATTCTCAATCAAAACATATATACCTCAACAAATATTACATCACTTCTTATAATATAAATCATTTGTGTTTCTTGTTTCTCTTGTTTAGTTCAATACAATGACTTATAACATGAGATTCTCTTTTATTTTAGTTTAGGGTTTAAGATTTCATCTAATCATCAGGGCCATTGTCATGGTTGTGTTTTGTACAAAAGAAAAAAAAAAAGTTTGATCTCTTAGTTTTGACATCTTTGAACGTGTAAACTAAAATAAGAAATTGGGTGGGATAGGTTTGTGTTAGACTTATAGTTTTAATATATACTATCTATGTATACCCAAGTTAAAAAGTAAGAAAAAGATATAAATTAATGGGGAATCTTAATCTACAATAGTTAGATTTTTGGAGGAAAAAGAAAATAACTTTTCTGTATGGAAAAAAAGTTATTATCTTGACCAATTTAAATCACACTTTCTCAATAAATTAATCTGCTTTAGAATAAATTCACACACACACTCATATATATATATAAAGTAAAATTTGATCACTTTTTGTGTTTTGGCTTTTAATTTATTTTAAATTTAGTTTCCAATTAAGTTATTAACATCTTAATTAATTACCACATCCATGAATAAATAATTATAAACAAAATCCTGTCATGTTAACCATATATACATACACAAAAAGTATGAAAAGGATTTAATTTTCGAATTTAAATCACACCTCCTCCAATTTGTCCTGAAAAAATATATTAATAAAAGAATATATTATTTTTTTGAACCACAAAACATAAATTAAGAAATAAATAAATAAACAAATTGCATAATATTTAGAGTGTATTGTTCACCTGTTTGGGCTTGGGCTTGGGCTTGGGCTTTGGGATTATTCTATTATTATTATTATTATTATTTTACAAATTTTCAATTTATAGGAAAATTAAGTTTTAGTTTAGGCATTTAAGCTCAAACATTAAAAGAAAAAGGAGTTACGTTTTCCATCAAAATTTTAATTTTTTAAAATTGGAAATGTCATATTATCTCTAACTTTGTACTCTTAATAGTGTTTAAGAACCTAATAATTAAAACCATTTCTCATTAATGATATTTTAAAAAATATATTAGTTTTTCTAGTTCAACTTCAAGAAGAAGACATATTATAGGTTGTGCATGACTAAATAGAGAATGTGTTTTTGTTTTTATTTTCTCTTAAAGATATATGAATAGTGAAAACATAAATTAATTTGATGCATTTATTGATAAAATAATTATGAAGAACTTATATTTAAAATTGTTTTATAATGTTTATTGAAGATGAAAATAACTTGAAACTTAAAAAAAGATATATATATGTTTTTTTTTAATAAAAGAAGTCAAATTCATTATATTATGTAGTGAAGAAAGAAAAGGGGATATAAAGAAAAAAGAGTATAAATCACAAAACTTATTCCCAAAAAAAGTTAAAGAGCAAAAAGAAAAAAGTTTAGTAAAATGGAATTGGGAAGGTAAAA

>Csa1M479630.1

GCGTAGATATATGTTTACAGAAGATTGAAGGAGCTCTCAAGACTTTTCTTCTTCTTTCTTTCTTTGCCTCTGTTTTCTAGGTTTCTTCTTTTGGTGTCCTTTAGATGATGGTGAGATGAAAAGAAGATGAAGAGAGGAAGAAGGAGAAGATGAACACACAATGTTTTAAAGTGGTTCGACAAAATGCCTGCGTCCACTGTGCTTTGGGAGCAAGATATTATTTCTTTTCAGTTTTTGTTGTGTATGAAATATCAAAGTGATAGAAGATATATTTATCGATTTTTCCATGTCCCTTAGTTAAAACCTAGTTAGACCAGAAGTTAAAACTAACAGATTTAACTATATGTCTTATACAACAAGAGATAACTGTTTTAGTAAGAGTTTCCCACCATTACTTTAGATACATTATTGAAATTCTCTTATTTTTGTTAGCATTATTCATCATAAGGAGGATGGATTTTAGTGTTAATGCACTTTTTTTTATTTCTTTATTTTACAATATATTTGATTTGCAAATAATATTTTAAAATCAAATTATAACCATATACATATGTATAGAAGTCGTTTTCCTAAGCGAAGAAAGATGTAATTCACATTTCAACAAAATCTACAATAATAATTATAATGAAAATGTTAAAATTTCTAACATAAAAAAATAGCACTATATTCATTCAAAATGTTTGATATAATCATCAAAAGTATGTTCTAATAAAACTTTCGTACAATTCTTTTCCTTCATCATGATGGATTCAATCGAAGCTCCATTATTTAATTTTGGATTTTTCTTTAGAGAGATTTCAACTACAACCATGAGTTCAGGATTCCATTAGTGTTCACCATCAAGCCTTTCAATAAAAAACCATATAAATGATAAACAAAATCAAAATTACTGCGTCAAGTTAGGATTCAGAACAAGCAAGAGACAATCCATGAATGACTGGACTAGTATCCTAAACAACAAAAAATACGATCATATTTCACCTAAAAGAAACAACACAAAACAAACACGTTTAGAAACTGTTAAGCATTATATATTCTCCTTTCCTTTCTAAGTTGACTAAGTAGGTAAAACAATATCCAATCAATTTACCTATAAATAGAGAAAGCGGAAGTGGGTGATAGGGATGAAGATTGAGGAAGTATTTTTACCTAAAAAAGCATTCACATTTAGCTAGATATTTCCTTATAAAAAACTTGTCCTGCAACAACAACAAAACATGCCAAGAATAAGAAGTAAGCATTTTTACTCTTATCTTTCTCATATTCTGCCACCCAATAACATATGTGACTTTATAGCATGTTATGCCCCTTTGTTACAAACAAAAATATCGAAAATGGACCAAACTCATTAATTTTAAAAGTTAGGATTGAAGGTAGTTGGTGCACAAGACCATACTTAGGAATGGGTGTTAAAATTGGCATCAGTAAATGTGTATACTTGAATGGTGAACACAAATGAGTTATTTAGTTGATTGAAAAAAAACAAATGGAATGGTGAAATGAAAGAAGTCAAAAAATGAATAAAATAAAAGAAAGCACAATCGTAATGATAGAGTAAAATAGAATATTGAATTTATCATTAAACTAACCCAATCTTTGAATCAATAATGAAAGCAATAAAGAATTTCCAAATTTTCCAAAACAAACCCCAATGGAAGAAGATGACGAAGGGAAGAAAGCCGTTGAAAAACGAAAAACGAACTTTGACCGTCGATCAAAGGCGGCGGAAATCTAAACCGTCCGATCTTCCGTCAGAAGCGCGTGACTCATTTTTCTCCCGCGCCCGAGCCTTCGTTTCCCTTTCCTCGCATTTACTCTTCACTCTCTCTCCATTTCCCCCTTTGACAATTCTTATAAATATTGATCCAAATTCTTTAATTCTTCTTCAAACTCTTGGTCTTGGTCTTCATCTTCATCTTCATTCTGGGGTTTCGTTTTCTTGACTCATAATCGGAGAATTTGATTCAGAAGGCGTTTCTGAGCAAGGGATTTCGAAGAAT

>Csa6M006730.1

TAACGCTCCCAATCGAGATTAAGGGTAAAACCCAAATAGCAAATTGGAAAAAGGAAGATGACCCAATTGAGATTGAGAGTAACAGTGAAATGGGGTGTTGGAGAAATGGGAAAATTCGAAGGGGTGAGTTGAAATTAGCAGGAAAAAAGCGCTATAGGCGAACAGTTTGTGTTGAAGTATTGAGCGAGTTATAAGAGAGATGGTGAAAGAGAGAAAAATGGGGAGAAGTTGAAAGGAATACGGAGTATAATTTGGAAGACTCGGAGGAGACTGGGCGGCCATCGTGTGTATTTGCTTTCTCGTTCGCACTTCCATGGACGACTTCGATCAAACTCTCTTTTTTTTTTATAATTTGAACGAGACTTTGATTCTTTTTTCTTTTTCATTTTCATGATTGACTACCTAATCTTCAGTTTACTTTTTTGAATAATTATCTCTTCTAAATATTGGAATGAGATCAACATTCACAAAATCTAATTAATCTAATTGTATCAAAACCAGTGAAATTCAAATTTATTTTAGCATCTTAGTTTTAAATAAATAAAAATAAGTAATACGATAGTAAAATAAACTTCCTTCAACGATAATCGATATTGACTTTACGTAAAAAATTCAATCTCTAAATTTATAAATTTAATATCTTATTTTATATCGAAATCTAAAAAGATATGTAAAGCAATTAATAAACTTAAATTAAACGTTAATTAATAATGAATGATTAACACAAACCACATCATCAGATTCTTTTAATAAAGAAAAGTTATGTTGTTGTGTGGTATGTCATATAGAAATTAATGTAGTCATTGAACTAATACTACAATAAACTAATTAATGCTAAAATGACGTGGTTTTTAAGACGCGACTTACTTACAAAAATATGCATAAAAAGGTAAAAATAATCTAAGAATATTGGAATAAAGTTATAAATACATTTAAGAAAAATTTCACAAAATCTATATTTTATCTAATATTTCTAATTCAATACAAACTAATGTGAAGAAGACGTCAAAAGTGGATGATAAAAGAGAGAAAGAGAGAACACGAATTTATATTTTATAGCATATATAATAAGGATTAAAAATGCACTTCTTTGACAATATAGCAATGCTTGTTATTATGATTATAAAAAGGAAATTAATAAGAGGAGTGGAAAAAGGTCAATGTAAATGGCTGACCAAATTTGGTGGAAACCAAATTGTAAATATAGAAAGTAGGAATTCAACAAAGGAGTTGGAATTGTGGAAACTAAGGGAACTTTGATTGAAATTTCACTTCCTTTGAATGGAATTGACTCTTATGTTATTTAATTTAGGTCTAAATCAACTTTAAACTTCAATCATGAATGTGATTATAATATAATGAAATTTGTTAAGAATGTCATTTTTTATATTTACTTCTATAAAAATGACGATAATTGAATTTAACCCACATGATAGATATATTTGATGCACTATTGACATTCACTTTAACATACATGTGATAAACATTTGACACATATTTGAAGATCTTGATATACTAAATATTTGAATTGATGATAAAAGATTGGAAAAAGAAAAATATATGCTTATGTGGTTTTATTTTAAGATAGAATAGAAGAGTTCAAACATCTAACATCTTAGTTGAGGGAATATGTGTTAACGTATCTTTGTATTTGAGAAAAAATTTCCCTAACGCTATTCTGAATGGACCCTTCTAATGTATATCTTCGAAAATGATTACATCCAAATAGGTAGATTATTTCTCAAAGTCCAATTAAACTAAAGGATATATAAAAAGTTATACTCTCAAAACTTAATTTGGAATCAACATAGACAATATTATGTCAATGGACATAATCAACCATTACATGCATGTGCAATGAATTGATTTTTTCATATAAGTGATCGTGTTTCATGAACATAATTTGTCACTAATATATCTTGCTTTCATATTATGTCAAACACACGTAAAATCTTCAAAACATTATACATATGATTTTGTTAGTCTTTGATATTAAATAC

> Csa6M365750.1

TGTTCATTGGCATGAATTATTTGAAATGTTCGTACTCTCGATTGTTGCTTTATTTCTTTCATTTTTTCCCACTTATTTTGGAAACCTTAAAAATAGTGTATTTGATATAAGTATGACAAGTGGGCCATATTTGGTGTATAGATTTTATACGAGGGCGGTTGCAAATATAACAACAAAATTTATGATAATAAAACTCATGTTAAAAGTAGCAAATCTAAAAATCGATAACCATCCAACAGCCCATGCTAACCGATCTGATAGCCATTTGATATAACTAATTATTTGTCATATTCAGAGTATGAACAAAATATGTGTCATTTACTGTTTTTTTCTAAATGTTTTTGTCTTATGACGCAATTTTCCTTTTATATACTCATATGAAGAGTTCATCGATGGAAGTGTAGAAAATGGTATTCAAATCTACGGCTACTTAACTGAGCATAGAATGCTATTGAGATGCAAACCATTAGAGAAGACGGGTTGTTAATTTCGAGAAAAATTTAGCTAAGAAAATGAAAAAAAATCGTACACAGTTTTGTTTGTCGTAATGCCTCTATACTCGGGTTGAAGCATAGTTTCAAGAATATAGTTAGAGTTTAGAATAATTAAGTGAAAATTGCCTCCAAATTATGTTACAATTATTTTTTTATTTAATATTTTATAGTTAAAGTTATGTATACGCAAATAATACACCCTAAAAGAAGAGAGTCCACCTCGACCGACGGCCTTGTCAAGGCCCGTGGATATCGAGAATATGAGTTTCTAGACTTTGGTTGTGACTTGTCGTGATGTTTGTTTATTTATTAATTTATGCAATATCTATTATGATACAATTCGAAAATCTGACATTTTAACTGTAAATATATCCGTAATCAATTGAGTTATTTTTAAGTTGGTCGATATTATTTGTTTCTCTCATAAATTCATGGTTATTATTATTCATGAACGATGGATGTGTATATATATATTATTTTTTAAATGTCCAATAGATTTGTTTGTTTGTTCTCTCTTTTTATTATTGTTGAAATTTTTGACATTTTTAAAAATATATTTTAAAGCTACGAAACATTTTAAACACAATATTGAAATTTAGATTTTTGAGACATTTTTCTAAAATTCAAAAGTTAAACACAATATTTATTTTATTGTACTTTATTTAACTTATTTTTATACTATCAGACTAATTACAATATATACTCTCTGACCTAAAATATTCCATATCAAATATGGTTCAATTTTATTTTAAAAATAAAATTTCATTTTCTTAGGAATCAATCCATTAAAAAGTAAAGTTCAAATACATAGCATTGAAGACAAATATTTACATTTACCAAAAAGAAATAATACAAAAATCACATTGAAACAATCTCCCCTAAACAAATAATAAAAAGAATAGATTTTTTTAAAATATATTTTAGAGATGAGATTGCAATGCGAGAGTGCCTTAAAGACCACAGATATATAACAAAAATTAGATTGCAATAGATCAAAATAGTCATATACATTACTACAATATTTATGTTGGAAAATATATTTCTTTCTTTAAAATATTCAAAATTTGAGTTATTTTTAAATATAATAAAACGAATCAAAATATTCAAATAATATAACAAAAAAATTTGAATTAATCAACGTCTATAGTTGTCTGTCAATATTATCTAGAAAGTGAGAGTTGATAAATAGTAAATTTTTTTTGTGTAATGAATAAATATTTTGATAAAGTTGGGTAACTTTTCTAAATTATTATAACAATAGTCCAAAAAAAGAAAAAAAGAAGGAAAGAAATTAAATAGAAGCAATGAATTAACTAATTAGTTTGTTAGTATTTTGGATCCCTTTGTTGTGGGAAATGTTGTGTTATTATTATCTGTTGATTATTAAAGGATAAAATATAACTTTTACAACCAAAAAAGGAAGAAGAAGAAGAAGAACAACAACAACAAGGGCTGCGCCAAAACTTTTTATAAGCAGGAACGGTCAGATCATCAGATCCATTCGAT

>Csa4M045070.1

ATACCTTCAAATTCAACAACTATATTATTAATCGGTACAATACACATTCATCAGAGAACACAAATCATTCAAAAACGAAAAGGGGTTTTTCAAGAATCTCCATAAAACAAAAAAGAACACACACACACACACACACATATATATATAAAACAGAGAGATTCAAATTGTAAAAGGGGGTTTTAGAATTGATCCTTACTTGTCGCGGCAACTGTTTGTGATTGAGAAAAGGAACGTCTGAAAGAGTGATCTGTTCCCAATTTCGTGCACGTATTGAGGTCTTATCCACCGATCATCCAAATGTCAATAATCCTAAGGGGTTTCGTCGGAAAATTTGGTAACGGAAAGTTGAGGTTTTTGATCTAGCAACGAAATTGGGTAGGGTAAAGGAGAAGAGGAAAATTGTTTGACCCAGAAAAAGATTTCGCAAACCCACCACGAGAAAAGGGGAAAGAAAGCGAAAGGAGGCTTAAAACTTAGTGAGAGAAGAGAAGCATGAGCATCAAATTATAGAAGTTTTGAGAAACAGAGGAATAAGAAGAAGAGAGAGATGTAAAAGGAAAAGGTAGAGGGAATGGAAGCAAGAGATAAAGAGAGGAGATCGGATGGGTTTGAAGGCTGAGGCGGAATTTATAGAAGTAACTCCCGCGCAGGCGGAGCACAGTCCGACGGCTACAAGGAATTGGAAAAGGAAAGTTTTGTTTATTTATTTGCTTTTGGAGTGAAATTAGGAAAATGTAAAGTTAAATGGAAGATTTAAGTCACTTCCATTGTTAGGTTTCTTTTTTTTTTCCTTTTTTTCTTCTTCCCTAAAATTCCAAGTTTTTAGCTTTACATTTCATTTTATTATTTTATAAACTGTTTATCACAATTATATTTTACTATATTTCGAAATTTTGGCTCAACACTCATTATTACTCAACCTATAATAATCACTCTACACTTATAATTCATCTCCTCAACCTCCACCACATGTTTATAACCAAAAACAAACAACAATATATTTCTTACTTATCATCTTATTCATTTCCCCAATCCAAACTCTTATTACTAACTTGATTTTAGGTCATTTAGATCATCATAGTTTCATGATTCATCTCACTTTTGTAAACATACAAGAAACTCTACAAATAATGTGAACAACACAAACTCAAAATGAATAACATGGTCGAATTGTGTTGACAAATAGTACCACAATATTTAATAGTTTATCCTTTTAGCTTGTACCTATGAAGAATGCGAACAACACTATGTATACACGTTATTGTTTTTCAAAATAATGTTATATATGTTAAAATAATATATATCAAATGACTTATTTGTCAAATTATTACTGTAAAATAATTTTTCTTTCGAAAATATGTTTTAAATACAACTTGGACGAAGATTTGAACGTAAAATCTATTTACCTCTAATCAATTAAACTAAGTTAAAGTTGTCTAAATAGTTGTATAAGAAGTAAATATGTTTTTGTGAATATAATTTCATTGGGTGTACTTGAAAACAAATATATAATAGAACTTATTTGGCCACAAAAGTAAGTGGATAGTTGAACAAACTAAAATGGTACCTATTAATAAAAATAGGTTTGGTATAGTACTTAAGAAGAAAATAAAAAGTACCTTCCAATCCACATGATACTTTGTATCTTTTTTCAAGTAGTGTCCTATCACAATAATCAACTATAAATTTTAACTATTTCAATTAGAATATATATATATATATAACCTAGCCGCTTGTCGCTCNNNNNNNNNNNNNNNNNNNNNNNNNNNNNNNNNNNNNNNNNNNNNNNNNNNNNNNNNNNNNNNNNNNNNNNNNNNNNNNNNNNNNNNNNNNNNNNNNNNNNNNNNNNNNNNNNNNNNNNNNNNNNNNNNNNNNNNNNNNNNNNNNNNNNNNNNNNNNNNNNNNNNNNNNNNNNNNNNNNNNNNNNNNNNNNNNNNNNNNNNGTATATATATATATATACTTTTTTCAAGTAAAAACAGATTTTACAAAAATCCACTCC

>Csa1M024990.1

AAAAGAAAGATCAAAGAAATCGGGTTTGATTCAAATCACAACCACTCGATCGAAGCTCCAGCATTGCAATGCCACGCTCTGCGCTACAAAAATTGTTTGATCTCTCTATTATCTCGCTGGCTCTGCCATCTATACATTATTTCTTCCCATTTCCAAATATTCACTCTTCTCTCTCTCTCTCTCTCTCTCCCTCCCTCCCTCTCCGCTACAGAATTTTGATTTTATCTTTACACTTTACATCCCTTTAGTATAAATCTAAATAGATTACATTAAACCATTATTAGTTTTCTACTTTTGGTGTGGGGCACTCTATAGTTTCAAATCCATAATAGATTTGAATCATTCATTCACAATCGTAATTCATTTCTTTATCCATGTTTGAATTAATAAAGTTTTATAAATTGCTTCATATATACCGATCTTGTTCTCGTTTGTGTGATAGTGAGGTGATGTTTTTTATTTTTCACATCGTTGACAAAAGAGAAAAGATAAACTTTTCGAGTAGAAGAAAATTGCTTTTGAGAATAATTTTCAAGTTTGTTGAACTAAATTAAATTAAGGAACGATAAATCCAAAATTTTAAAATATAAGAACCAAGTCGATGATACAAAATAGGTAATTTTAATCTTTTTTTTTCTAATAGTTAATATCTCATATATATTAGATTAACAATTTTTTACCAAGTCGTGAATGGAGAATCGATTTAATTTTTTATTTTAAAGTTAATAGTACATGTTTTATACTTTAATCTAGCACGAATATTTTACAAAAGAAATGTTCCATTTGGTAAGGTAAAAAGTACCAAAACTAGATTTTCATAATTCTCTTAGTTTTATATTCTTTCTTTTTTTTTTAATTTTATTTATACTTTCTAGGTGACATTAATAAATAAAAGTTTGATATGGTAGGGGAACAGTTCCAAAATGAATGTTGTACTATTTCATTAATATTTAAACATACCATATAGTTTGTGCATTTAAAACACGTAACTAACAATAAATAATAATGGTAGGAAAAAATAATAGTACATAAGTTTCTCCGAACATGACGTGACTAAAGAATAAAATGATCAATACGCTTTTTGATCCATAACACACCTATGTGTTAGTTGGACGAATCTCATTTTTGTTAGTAGATGGTTTTTTTACATTACTTTTCTAGTTCAACAATTACAACTTTATACAACATGAAAACCGAGCATTTGACCAATTAAACGTGAATTCATACATAAAGGGTTGAATTAGCTTTTATAGTTTGGGTTGATTTTGAATTCCAACTTGTTTTAAACCAAATTGATTAAATACATTAAAGTGGGTGGAATTATTCTGCTTTCATTAGTTAACACAAAGTTTTCTTGTTGGTTAGTTTTATAATTTGGACAAAACATTTGTAAGGGGAGAGTGTAATTATAATTTAAAGATTTTTATTTAGTTTATTAAACAAAGATTTGGGTTTTAAATATGAGAATGATGGGTTGGAAAAATCATATAAAAAAAAGGTTTATTTAATTGATCAAATTGACCTTGATTGGAGGCTATAAGACTTTTTGCAAAGTATTTTAATCATAATCAAGATTAATATATGTAATCAACTAAGATGTATCAACATGATATGCCATGTCTCGTTATCAAAGGTTTGTCTGTACAATTAACAATTCCACATGAATTAGAGTATATATCACAATTCACATGTCCCACATATTTAATTCCTTCATAATCAATTATTTTTCTTATTGTTATTATTTCTATTTAATTACATTAAGAAAAACAAAATAACTAGATATTGTGTGTGATGATTGACGAATAGGATGAGTTGAAAAATAGGATGGAAAAGAAAAGGACAAATTTGACATTCAATAAGAGAGAACAACATTTTGGACTAATAATTTGAGAGTTGAATTATTAAAGAAGCATCTCTAAGTTTGGATCTGTTAATCAGATCTATTTTTTCAAAATAACAATTTAATATACATACATATATATATATAATGGAGAAATTGTC

>Csa1M042720.2

ATAAAACTGGCCTTCATCGGTTCTGATTCAGCTCTCTGCCATTGAATTCCACCATTTTCCCTTCTTCATCTTTGGAGATTTTCTCGGTGGCCAATCATCATCAGCGCCACAAAATCTGGCCTAAACTAAATCTGGCGTAAACTAAACTAAATAAATAAAAGATGATCTCCAATTAATTCAACATTTTTTTAAAAAAATTAAATGCTATGTTAGAAATTATGAACAAAATTGTTACAAAAACTTGCATGGGAACAGCCTTCAACCTTCAAACCCCCATTTATCCAACATTTTTTTCCTTTTGCAATGATCTAATAGTTTTAACTTGAGGAACAATCTTATGCTTTCAAATTTGAAACCATTCTATCACGATAATTTAACAAAACCAAGTATACTTTTTAATAATGAAACAACTTTTTGTTTTAAGTTTATAAAAAAATGATTGTTCATGTACCAATGAGTTAAGTTCTATATTTTGATTGAGTGTCATCAAATAATTTATAAAATTTTCCATTAATGACGTATTTGAAAACAAAATTATGGTCATCCATTTAGTTCTTCAACTTTAATTTACAATATTATTTAGTATGTATATCTTAAACGTCAAATAATTTAGTCTTGAAATTTTACCACGTCAATATGTAGTTCTTATTGTCACAATTCTCATCGAACTTTACAAAATAACTCAGTTTTATATTTAATAAACAAACATGGTTCTCTAATTAATCTAACAATTTAGACTTTTAGTATTTTATTAAATTTTAATATTATTTTAACATTAAAAGCTGAATCATTATAAATTACAACATATAATAACTCACTAAAAAAAATTCGTTATTCTCCATTTTTCTCTGCACGAATGAATTTGTTAAAGTGATAGCCGCCAATTTAACAATTATATTCAAAATAACTAAGTATATAACAACATTTTAAAAAATTTGCAAATATAGCAACACCGATATACCATGTTGAAAATATTAGTTTATCACCGATACAAGTCTATCACCGATAGTTTTACTATATTTGCAATTTTTAAATTTTTTTGCTATACCTTTAATTATTATTGCTGAAATGGTTTCTCACCTTTTTTTGCAACTATTTTTACAAAATATTTTTTATTATCCACTATTATAGTTTTTTTTCTTCTTATTTTTCTAAAATCAAATCTAAATTGTCCCAAAACTATTATAATTTTGTTCTTATATTTCTAAAGTCGGTGATCTAAACTATCACTCTCTCAACCATATTTCATGAAAAAATATTAATTTTTTTGATGCAGTGTATTTATTATATAGGGTCTAGTACCCTATCTATATAAAATTTATAGATCTGTCACTGCTTTTTAAGTGTTCGGTTTTTTTAAAAGAAAAGTAATGTTACTCAGTCAAAGTAAAAGAAGATAACATTTATTATTGTATTTACGAATATTAGTTTAGGTGGAAGAGAAAAAAAAAAGTGATATTAAAAAATAACAATTAAAAAAAAAAAAAAACCTCTATCTTTCACTAATTCGAGATTACAACCCTAGGTTTTGCATTAAAATAAAAGAGAAAAAACAACAACTTTAAGTTGTACGAAAAAGAGTTTATTTTTTCTTTCTCCATAAAATAAGTAAACAAAAAAGAAAAAAGTTGATTTTAATGTGTAAAGAAGATAAGATATTATGCGCCTTTTGTCTCAACTTATTTATTGTATTTTTCTAAAAGCACTGACCATTCCCATTTCCCATTTCCCATTTCCCATTACCCACTCCCTCGTGCGCGGCTCTTGACCCTCACGCGCTTCTCCGCCTCTCCTTAAAGTTTTACATTCTTAGATCCTTTTCCATTGGATCACACCACGCCACGTACGCAATAAGTCGTCGAATTTTCCCCACTCGCATCCACGTATAAATCGTCCCGTAAAGGCGCGCGTGAGTGGATATTTATGTCCAAGATTTCTTCTGTCCAATAAGAAGCAACCGAAATTGGCTTTTCGCATTAAAGAAGCAAAGCCGCCGTAAA

> Csa5M152810.1

ATATCTTCAAAAAGAAGTTGAAAAAAGTGCTTCTGCTTTCTCTCAAACTTTGAGGGAGTCTACACTTTCTTCTATCCTAAACTTTCTAGTTCTAGTAACCTTTTTTGTTTTATATCATTTTGTTATCAATTAACCTTTTATCCCTAGTTTTAGGTCGAACTAGTATATATGGTAGAAAACTGAGTTATTTTGATCGAAGAGGATTAACCAGTGAACTAATAGTAAATGATGTGTATGAATCTATTTAGAATTTCATGATTTATTGTTGTCTTCTTATTGTTAGGTATAGTTCAACTTGGTTTGAAATTACTTGGATATTAAATTGTTGGAATGTTTGGCAAGAGGGTGTAAACAAGTTTCCTTAATATAGAAAGGACAACAACTAAACTCTAATAAAAACTAAATGAATGTTATCAAATCAATCAAACCAACCACATTTTGGCAACACTAGTGCTGGAGAACAACCTCATATTTTGAACTTATGCGGAGCGAACTATTCGAACTATCTTGAATTGAGTAAAAGAGAGAAAAAGGGAGTGGTTTGAGAAGAAAAAAAATGCTGAAGATTTTGAAAAAGAAAGAAAAAGAGAACATGAACATATTGTGAAAACTTTTGGGGACTGATGGGGAAATCTTGGATGAAGTGAATTTAATGCATTGGGGGGACAAATCACAGCACTTTGTTATTAATTTCAAAGACACCCACAGGGTAACCTTTGCTGGAGATTGGGCTAAGATCTTATTTCTTCTAAACTATGAGGCCTTTTCTGCAATTTCCCAAATAAATATTAATTTATTTTTAGGGTCCCTTTTGTATTTATAAGCCTCAACTATCTCAAGAGCAGTTGTGAAAGAAAGATCCCTTCTCTTTTCTTTTAATTCTTTTGTATAGTTTTGATTATATATACATATACATGAAACAACCCCTTTTGTTTTAATTAATTATCTTTGTTTATATAGTCAACAACTCAACTCTCTTCTAGAAAGTTGAAGTTCAAATCTTCCCATCTATTATAATTGATTTAAAAAAAATAACAATAATCTTTTTCTAATAATTTTTTAATACAATTATAAAATCTTGGTAGAGTGCAATCTTATATAGAAAAAATAGGAAACCAAAAATTATAATAGATGACAGGTTTGAGTTGATAAATAAATGGAATAGTTGTACTATATGTTATTAGAATGTTAATACAAATCATAATTTCAAGCCAAATAAGTTAAATGGTTAAACAAATAACTAAAATTTGAGTTAATTTGTGTGTATTTTGACTAATTTTAGCAACTCATTCTCTTTTGTTTTATAAAGTTATAATCTCTATTGACTATGGACCAATTCATGATAGTTAGCTTTAGGATTTACCATTTTTTTTTTTAAAAAAAAAGCAATTGACAATTCCAACTCTTTTTTTTAATTAATGAATCCAAATTGTTGAACAACAAGCAACTGTATCATTTTTTTTAAATAGAAAGGAGGAGATTTTAAGAATGTTGGATAAGGAGGAGAAAGAAAAATAAGGAAGTTGAAAGAGCAAATTTCTTAAAGTAGGGCTGAGCTGACCCAACTGCAACAGCAACAGCAACATTGTGTATGTAATCGATTTAAAGAAGGGAAAAAAATAAAAGGTAAGAAATTGCATTAATGGTGGTTTTAATTACATCTTATTGGAGTAGAGAAGGGCGTTTGTTTTTGGTGGAGGGAAAAAAGTCAGCTCTGATTCACGGATTTTCAAACCATTTTCAGATCAAAAAGGAAGAACACAGAAGCCAAGCAATCAAGCAATCGCCATTGCCAATCGCTCTTCACATTTTTCAGACCTTACTTTTTCAAAAAGCCCCAAATTTTCACCTTTTTTTCAGAAACCCCATTTCTCATTTCTCTTCCAATGCTTCCCCAAATATAGCATTCGTTTTAGATGTCAGTAACTGAGAATAAACAAACTTCCCCCAGTTGCTGATTCCCTTTGAAACCGCTTCTGGGTTTTCTTTCAATTCCTAA

>Csa1M077220.1

AGATTAGTTGATTTTAAGGTGGAAAGAAAGCTTACATATAGATTATCAGTTGTATTGTGAAGCTAAAGTGTATTATAACATTAAATTGATTTGCATTGGAAGGGTTGCCATACTTGGTAGAACCTTTTGATGACGTTGAAGTGTTGGAGTGTTTTCTTTTTTATAAGTTTTAGGGATGAGCTAAATTTTGATTATTTCAAGTTTAGGAGAGCATGTGGAATATTTTTGCACGATTATATCCGTGTGTTGATTATGTTGTATGTCAAGAACATTCAAGTGAGATTAATAATTATATTTTGCTTAGTTTTCGAGAGTTTTGGATCAAGGAACTTGTTCTTTGTCTCAAAATCTTTGATCCTAAAAGAGATGTCAATTCTAATTTTTTATAATTATTTTCGTATCAATTATCGTTTTCCTCTAGTTCTATACGATCTTCTGTTTGTCTCATTCAGTAATGGATTGAAGGGGATCACTAGCTCACATGCATATCTTGGATCTTGATACATGGTGCTTGTTTAAGTATAAGCTTCTTGCCAAGAAGTTGAGTAGGTTTATCATGAATTACATCAACATGACTTGTAGATTAATCGATATTATTATTTATTAGAAGAAGAAAAAAACTTTTCGTATATTGGGAACAACAAAAGGTAGATCTAGACATTACAACAATGACTCAAATGAGGCTACAAATTGAGTTACGTTACTTGGGGCATAAATTACAAAATATATCTTTCATTTAACGTGTGTTTGGTTTGACTTTTCAAGTGTTTAATTTTTTGTAAATATGTCATTTTGAAAAGAAATCAAAATCTCGTACCAAATTAAGAAAAAATAGTTATGTTAGATTAAAAAGAGGTAAAATTTGATCGTAAGTCCAACCTTGGGAATAATATTCAACTGAGGACCAATTACCTTAAACTTTTCCAATCTTTTGGGTGAGTTGCTATCATATAATGACTTAATGAGAGACATTATTCAATAACTCCCATCACTGCTCCATCTTTCACTATCAACTTCATAAATATGAACAAAACAACACATATTATGTATTTATTGTAACCTCTCATCCAAAATTAAACTTTAAAAAATGCTCATCGGCTTCCTTTCTTTCGTAATTATTTGACTTTTAGTTTTTAGGTTTTCAAAAACTAAGTTTATTAATATGCATACGGCGGTATTTTTATGTTTTCAAGAGTTCGACATTGACATATAGAGATTAATTGATATATTTTTATATTGTAGAAAAATCCACCATACATAAAAAAAGAATCAGAATACCACCATACAAATATAATATAAAATAATAACATATTTACTTGTTTAAAAATAATAAAAATGTTTATTTATAAATTAAAAATTTTAAAAAATATCCATTAAAATCATAATTTTTATCGAAATTTAATTTATGTAAAATCAAGATATTGATCTCAATATTTTAAATTTTAGTAACTTTGTACTAAAAGAATGATACAAAAGTAGAACAACCCTAAAGTGGGTTTGGGAGTGATAGTTATTACACTCATTTTAGAAAAAAGATTTAGTTTACTGTTAATTCAGAACTCTTGTTTTGAATTGTTGTGTTATAAAAGTTAATTTAGAGTAATGAATTTAGTAATTATATTTTATTTGCTTATGGTATATTTGAAAATTTGCGTTTAGAGAAAAAGAGAATAATATGTAAACTCTTTCCTTTGTCATAATTCTATTGAGTAATGAAGAAAAAAAAAGAAAAAGAAAAGTGAGTGAGTGAGTGAATATTCAAATTTCAAAGTTTGAAAACATGAAAGACGCGCAAAGTAAAAATTCCAGATGAGATGGCGATTACACTTTTTCTGCTTCTCCGTCTTCCTTCCATCAATCCCAAACCAAACCCTTTCTTTATAACTCTCTCTCTCTTCCGCCATTATCACTGACTACAAAACGACCTTCATTTCTTCTTCCTTCTTCACCCCATTTTCATACCCATCATCAACAATAACAACAACAACAACAACAACAA

>Csa5M002030.1

ATTCTGATTGCAATCACAAATATCAAATCAGAACCACAGAAAAAAAAAATGTAAATAGTAACATAAGAAAAGAAAAAAAAAAAGGACATAAATTCAACCAAAACCCTTCTTCCTTGATCTATAGATTCAATAAGACAACTACAAGAATCAAACGATAAGAGATTTCAGCAAGAATGTCTTTTAAATTCCAACAAAAGACACCGAAAAGCATAAAAAAAATAACCCAATAATATCTCCAAAGAGTCAATAAACCTTTGAATAGTTGAAGTCAATTATAATCACAAACCACGGGAGCACTATTGAACTGTTCTTCAATTTCCATACTTTCCCCCAACAAGGGAGAGTCAACCCAGATGAAATCGGAAAGATTCAACACAAGAAACTAGAAATATAATCAAACACAATCGTGAATTAGAGAGTGTTATCAGTTGCGTAAAACGACAAGAACAATAAAACCCTTAATAGCTAGAAACAGACAAAATGCCTCGAAATGCGGGCCTTATCCTGTGAAATTCTGATGAGGAGAAACCGAATTAATGGGGAATAAACAAACCTTGTAAAGGGGTCCACAGTCCAGACAGAGAGGCGTATTTTGATGGTTATCAAAGAGAAAAGAGATGGGAATCGAAATCGAAGAACGTATGAATATGAAGATGAAAATGAAGCCCAGTTTTAAGGGCAGATGAAGAAGAAGAAGAAGAAGAAGAGGAAGAGGAGGAGAAGGGGAAAAAGGAATGAAGAAGAAGAAGAGTGATCGATGACTCCATTAAAGGACTAAGTTGAAGGTCTGGTAAAACCGAGTCTCCCATTTTATTTGTTCGCTTTTGGCTGCGTACTTTTCAAATAAAGTTGGAAACTTTTGAATGATTTTATTTATTTTTTTATTTTTATTTATTTATTTAAAAATTCACCATCAAGTTTCTTTTTTTTTTTTTGGATGAGAATGAACGCAAATGTGCCACGTCAATGTAACGTGTTTGAGTTTTGGTATTAATTTTTTTCTCTACCAACTTTAATCTTTTAAAATACTCTATTATAATATAACTTCGTTCATATTTATTATTTCTACCATCAAAGTTATTATTATTTAGATCCATTCGACTGAAAATTAACTTTTGGGAGACAAATTCTTATAGATAGAAAAGAAATATAGTAAAATACTTAAACGAATAGATTTTGTTTTTTTTTTAAATAGTTATTGGATTTGGATTTTTAGTGTTATTAAATTTTAATTTAATTAAATATTTTATATGTATATGTGGATATATATATAAAATTTTTATGTGAAAAATGAAAATATAATTATTATGAAAAATATATTTATATTTTGAAAAAAAAAAGTTTGATGTGATTGGAAAATAGTTATTGAAGAAAGAAAAGGATTTGGAGGAGGAAAGAGAAAGATTAAGACTTAATAAGGATGAATAAATTTATTCTTTTCCTCCCTTAATCTTTGTCTTATAAGCTTTTCTTTTCCTGAACGGTTGTTTGGAGAGTTGGCATGTAATTAGCATTATACGAAATTAGAAGAGTGTAAAAAAGCAGAGTAATCAAAGATAGAACATGAATAAGATCCAAAATAAATTGACTAAAAAATTAGGGGTGTTCAAATAACGTAACAACTCGAACCACCTGACTCAGATTGCAAGGGTTAGGTCAAATTTAGGTTGGTCCAAAAAACTATCTCAAACCTAACCCAATTCTTTTACACTCCTATCCAAAATGTTCGATCAAAATATGAATTTTAGATTTGAGTACAAATTCGATTTGATACGTCAGTAAACATCGTGTTTCTTAAATGTACGCCACAAGTTATTTATTTATTTTTGTCAACTTTAATCTTTTAAAAGAATTGAATAATTGAAATACGTTACTTGGTATTGTCAAAAAAGAAGAAAAAGAGAAATGTTTTAGTCTTTGCTTGGAACTCAATATTGGCTACTCATATATTCGAAGGTATCAATAATTGTAGCTTTCTATTACCAAATTTCAACCATTTA

>Csa6M061230.1

CTTTTAAGATATTTTAACCATCTCTAAAATATAAATACACCTTGTTTTTTTAAATACATTTGGAAAGTGTTTGTAGTTTTTATCATACATTGGAAAAAGAAAAATACTTTTCATTTCTAGAATTTAACATTGGCTTCAAGTTTGTTCGTGTTCATTGAAATATGGATTTAGAGGGTATATGAACCACCAACTTAAAAGTTGATCGTTGAGCCTCGAACAAACATTAGGATCATACTAAAATAGTTTGCACTCCAAACACAAACTGTTATTGAATTTTTTTCACTCAAATATAAAGTTATAGTCTTAGTAAATCAAGTTGTATCTATGTTTTAAATCACTGAAGTCAAGTTGATCCATTGTGTTATTTGAGTCAACTTTTCATTCTACTCATGTAATTATACCTTTCTTTAAGTACTATTGATCCTTTATAATCCAATAACACATTTAAGTAACTAAACCAGTGGATTCTATTGTTCTAGTCTTTAAAGCAACACTTAGGAGGACTATTTATCTTCTTGAAGAAGTTAAAGTTTAATGGGTGGGACTATAGGATAAAATTTTATTTAAAATGTCAATATTTTAATTGTTATTTTTCGTACTTTCATGTAGTGGACAACGACATCATAAGTGAAATTGTTGTTGTGAAAATATCATACATTGTTGTGAAAATAGCAAGTAACCAAGGACTATATATGGTTCCGTTTGATTAGAAATAGTATGGGCATCCTTTATAACAACGAGATTTGGACAAAACCATGGCCATTGATCGGCATATTTGGAGGCTTAGTAGTAACCGGTTTTTGATCTAAAGACAACAACTCTCGATTCTTGTTTAATTTTGTGCATTAATTGAAAATCATCGATGAACTTGAAGACAAGTACACATGGTTGTCCAAAACCTCATGTTAGCTACTCTATTTTCTATTGGCTAGAAGACCCTTCTTCCCTCAAATTTGTGGATTCAAAGTCAACGGTACAATAGTATGTTAATTGAGCTATGTTCGTTTAATTTTACTCTTCTTACCATGATTTGTTTGTTCTCCGATTCCATTGGACAATATATATTTGTGGTTAATGGTCAGAAATAAAAATTATATATATAAAAAAAAGAAGAAGGGAATGTCAATAATTATTTGTGAAAAACTAAAAAACTAAAAAAATCAATTATTATTATCCATTTCAGGCACGAGCGAGACTCTCTACTTCTCCCTTCTCTCTTCTTTCTCTAACTCTGCGCCATTGCCTCTTCACTATACCTTTCTTTTGTTTTCTTCATTTTTTCTCCGTTCCGATCTTTCTTCTTTCTTCAAGATCCGCTTCCATTTTTCTCTCACAATCTCCCTTTCTTTGCTCTAATCAACTATAACCAATCTGTTCAAAGAACCCCACATCTCAGTCTCTCTTTTCCTCCTATTTTCCGATTTTCTTGTTCCAATTTTTTTTCGGGTTCTCTCGAGATCGTCGCTATCTGGGATTCTCCAAATTTTAGTTCTTTCCGCCACTTTTCTTCTTTTAAAAAAATTTCAGTACCATCGAACAACTCTGACCCTTGCTTTGATCTGTGTACAGTATTGTTCTCCAGCCATTTTTTTGGAGACATCGCCGAGTTTTGTTGTGGGCGATACTTGTGTTTATTCACCATTTTTACATTTCAGTTTGATCCCAGCTATTGATTAAGTATTTTGTTTTTATTGAACTTTGAAAGTAAAGTTTTTGCTATTGTCTTAGTTGAGTTTGCCTTTTGGGACTGCGCCTTTTGGTTTCTTTCTTTCGGGTTTGAGTTTGAAATTGGGTTTTCTGTAGCTTGTTTTAAAGCTGTAGGTTAGTGATCTTAAGAGCTTACTGATTAGTTAAGTTATTGGATTACTTACTTGTTGTATACTTGATTCCAATTGACATTTGGTGCTGCGGATCTCTGATTCTGCTCGAGCTTGAAGCTACAGCGTTAGATGCCCTAATTGTTATATCGATCTGGGAACTTGGAAAGCTTTGTTGTTAAT

>Csa6M179480.1

TCTGAGCAAAAATGTGAGGGAAGTCCAAATTTATGAAGCAAGAACAGTTTCAAAAGATTTTGAGAAAAAGGGAAAAATAGTCACTAAACGACGAAGCAGGAAACCGATTGGTTATCAAAAACCCACCCACATTTGTGCTGCTGTTGCCCGTCTACCTACCGGATCACAAGTCTAGAAGCCCGTTTTTCCATTAATCTTCCCCAATCCTCCATGAAACCAAAACCCAATAAACCTAAAGCAAATCGATCCCTCTCCCCTTCTATTTTCACAAAAAGAAATACCAATGACACCTAGAAATCTACACCTCCTGTAACAGTGGACATAAAATAATCAACACAGATCTAGAAAGAACTAAAAAGAAGAAATATATATCTTCCAAATTCCAGCAGCTAGTAAAACCAAACCTATCCAAAATCCAAAAGAAAAAAATAGCCTCAATCCCCCTCAATCTCAGATTCCCTTTGAAATTAGATTAGAGCGGCGTAATAACTCGGAATCCGTCGATGAAAAGGAAGCTCCGAGATCTGCACCGCACCGACAAAAAAATGGGAAGAGAGAAAGAGGAAATGGGAGGAGGAAAATCCGGAGGTGTAGAAAGGAATAAATCTATTAGTAATGGAGCATATAAGGAGCCTTGGCCGATTTTTTTCTTTCTTTTTTCTTTTTTTCCTTTTTTAATTTATATATACTTTTTTCTTTTTAGGAGGACTAAATTTAATTTTTATAAAATACACTTCAACTTCGTCATTAATTTAAAATATATTTATATATTCTTAAAAAAAAATTAATATTGCTTACCTTTCATCCACCTCTCAATATTAACCGTGATTGATATTATCTTTCATCTCTTCTCAAAATTAACTTTGATTTAATTGAATTTTTAAAAATTAAGTCGAATTTTAAAAACTAAACATATAATAGTAATTTAAACTTTGTGTCTTGTGTTCTAAAATTTGAGTAATTGATACTAAAAATTTGAAGAAAAAAAATGATTCTTACTCCTCAAAACCAAATGATTAAAAAAAAAGAGTAAAAAAAATCTCATAATCTTTATAAAAGAAATAAATTGTCCAATGGGTAATTAATTTTGAGACATAATACATATTATTGTATATGTGGGACGATTTTAATTTCTATGTGATATTATTGTCCCACCATTTTAGTTCTGAATTTGTATAAACATATTTTTGTATAAATGATAAGTTGAAATGTTTAGTATTATACATAATTTAAAAAGAAAAAGTAAGAGTATTTTTGAAGTAAATGGTTTTATTTTATTATTTATACCTGTAAATTTGAATATTACGAAAGGCACGCACCATGCATGTTAAAAAGGGGGAAAAGGAAAGGAAGGGGGAGGGCATGATTGTAATATGAGGAGAAAGTAGGGTTATTTTTTGGGAAGAATGGGTATTAATTTAATTTTGTTAATTTTGAGTGAGAAAAGCCAAGGGAGAAGGGAGATTGTAATGTATTGTGCGATTCCATTGAGGTACCGATGGGTGTTCCCAGATTTAGGTGCAGATGGGGTTGGGGAAGTAGGGGCTACGGCCTTTTGTTATTACCACGTCATTCTGACACGTGTTCTATTTCTAAATATAAATAGTCGTTGCTCAGTCCTTCCTCCTGGCCCCACTTTGGACGTTCACCTGTCAATACACTTTCTCTTCTCCCAACTCCTACCCAATTTTTTATTTCCTTTTCTTTTTCTTTCTTTTCTTCTTTTAATCTTTATACAAATAACTATTCTTATTTTTCTCTAATTAATTACAACAACGCCTTCCACTTAATTAAATCCTCCTTTCACCCATCAAATTCTCTCTCTCTCTCTCTCTCTCTTTTTTATAATTGATTAATGACAAACGTACCTCAACTTGCACTCCCATGACTCCCGAGAGATTTCCACTACAAAAAAATTATAATTCAACATGAGTAGACTAACAAACAAATGAAATATAAAAATAAAAATAAAAATGAATCTTGTCAAAACTTATTAAAAT

>Csa4M082320.2

CTTCTTCTTCCTCCTCGCTCCTATTTCACTTGTTAAATCTTCAGAAAAGGCTCGCCCACATTGACCAAACACAAAACCAGAGACAAGTAAAAAGCCCAAGATTGGTCCCAAAAACCCACAAACCAAAAACAAAAAACCCCAGATCGAAAACCCACTGAACCCTTAAATTTCCACAAACAAGACAACGAACAGAAACTGAAAAGAAGCTTCAAACGCCTCAACAAAAGAAGCCCAGCAAAATGGGCGGCAAAAAACAGACGCAGATCGGAGGGGAAGAGAGAGAGAGGGGAATTAAGAAATGAAATTGAAAGGCGAATGGAATATATAAATATATAGTGAGAGAGGGAATTTTAATTGGAGTGTTGCGTGGTTTCTTCCTTATCCACACTCGCAAATTCCTTCCTTTGTTTCTACTCTCTGTGATTTTTCAAATTTGGTTGCGGTGATGACTGACTCTACGCCAGTAGTGGTTTCCATTGTAATTTTTCGTTAAAAACGCCTAATTTTTATACCAATTCTACGTTATTTTAATTTCATTTAATTTAAGTTTAATAGAATTGAAAAAGCTTTCACTAAATATTGTTATAACAAAATTTCCCATTCCATAAATAATAAAGACTTTATAGCTACGCTTTTATGAATATACACTATTGGATAGAATTCAAAAATTTTACTACAATTTTAAATATTTTGATTTATTTTACTATTTTAAAAATGGTTCATAATCTTATCCCATTTTCTATACTAAACGTTCTTTTTTAAAAAAAAACAAATTTATTACCTCACATTTTGCAAATAAATCAACATTGTTAGGCAATTAAAGTTCTCTTATAGATAGTTTACTTTGTCAAGCGGTTTCACCATCTCATTTATCATCATGTTTTTTTTAGTTGAAAATGTGAACAACTTGTAGGCAGTCTAATTCAATGATTATTTGCTATCAGTAAATAGAATCAACTAATTTTATACCTTGTCAGCATTTTAGCTATTGGAGGGGTAAAGTCGATCTCATAACACATGGCAGTGGTAGTAGAAATATTTTCATTTGAATTTCCTGTTATCACTCCAATACTTGTGTGAGGGGTGGATAACCAAGCTGCATCAACATTATAGTCGCAAAATTTTGTGGAGGAGGATGCCAGCCTTTGTTCCCAGAACAATGGAATGCAACAACTGACAGAGTGGCCCTTCTTTTATAGAGCAAAGGATTTCAAACCTTTGTTCGTATAATGAACAAATTCAAATGCATTTTAATTGAGTGGAGCTAACTTATTTGTCCTTAAAAAATTGTTTCGGAACAGCAACTTATCGCGATTAAGCTTAAAGACTTAAGAGACAGAGAATCATTCAACGCTCTCCAATGATCGATGAAGCAATCAATGAAATTACTTTTTGTCAAGTAAGAGCTTGAAATCTTCTTTTAGATTGATTTAGACCTACAGCAAAAAAAAAAAAATACATGATCAGTGATTTCAAAATCCTGAGAACAAATAGTGCAAAAGGGCACATTGATTCGTTTTTCTCATAAACCCATATAAGTGGGTAAGATGTTTTTTAGAGCTCTCCATGCGAACATCCTAATTTTTTAGAGGACTTTTAACTTCCAAACATTGTCCCACACGATATTTTTCAAAATGCCTGATTATTTTGTCTTTTGGGAGTATCTGTGCACAAATATTTTTCTAATAATTTCAAAATCTTCATTCGTCACAACCTTTTTTTAAGTAGATCAAGATTCCAACTTCCATTTTGATGGATGAAATCAGCCACAAGCTGAGAATTGAAATGGTCATTTATGCATATTAGTTTTAAAAGTTGACACTTTAGGAATCCATGAATATGAGAACATCTTACTCGATTTGTCATTTGCAACCTCAAATGGAAGGCCCTCCTTTGTAAAGGAGCAACCCCAACGCAAGCTTTTCCATAAAAAAAATAAAAAAAAAAGAGTCGTTCTTCCCTTCCTTTGCTTGAAGGATATTACAAATTTTAAAGTAAAT

>Csa6M423420.1

TTTCCTTAAAACTCTAAAACCCAGTTTGGAGAAGAGTGAAGACGAAATCTGAAATCCAGTTCAGAAGAGAAAATAGAAAAGTTGAATTGTTAATTGACTAAAAGAAGACTCAACCACATTTGCCCCATCTACCCCTGCTCCGGATCACAAACCCACAATCCCTCCTTATGCTCCTTCCCGAATCCTCCACGGAACATAATAAAATTACCCAAATCAAATCCCTCATACACTTCACTACCTGAAATCTCAAAACCTGAACCAAAAACTCAATCACCAAAACAGAACAACCGACACCACCAGCTACCATAAAGTAACCAGAATCAGATCGAGGATAGAAACTTTTTGCATTCCTACTGGATGAATGGCCGTAAACCCAATTGGTAGAGGATGAAGAAGGAGATTAAAAAGAAGGGAAGAAGAGAATCGGAGATCTACAGGAAAAGGGGAAGGGGAAGAAGAAAGAGGAAAGTTTGAGGAGAAGAAGGAAGGAGCGGCGAAGGCTTTGAGAGTGGATATATAAAGTATTAAGATAATTTGAAGTGAAAAAGTAGAAATCTAGGAGACCCTCTTGCTTCCTTTTGCTTTTTCAATCCTTTTTTCTTTCCATGGGCCTCTCTTTCTTCTTTTAGGTTTAAATATCATTGTGTTATGTATTATTCCCACTTTTATTTTCTTTTTAAATACTTGCTATCCAGGTATCATGTCAATTATCCTTTTTTTATTGGTAAAACACTTTTATTAAACTTTGATCTCTCTAGTATTGATTATATCATTTTTAAAATCAGACTTACTTAATAGATATTTAGTAACATTTGAAAATAGTTAATTTTGAAATTTCGATTTTCATTCGTTTTCTAAAGCTTATTTTAAACATTTTCTTTATCCAAAAACAGTTTAAATGAAAATTATATTTTAAAAACATTTTCTCTAATCCATCCGAGTTTTTACTTTTTCATTTTCATGATTTTCTTTTATACACCTTTCCTCTATAAATTCTTTTTATATATTTACTCCTCTTTTATTTGATTAGACAAATATTTAGTAACATTTCTTTTTTATTTTTTTCTTGCGTAAAGAAGAAAATGGAATAAAGATCAAACCTTAATAACTCAAAATAACATTATAAGTAGTAAAGTAGTTTTTAAACAAAAAGAAAGAAAAACGTATAATGTTAGTTTGAATTTTGTAACGGATGAACGCTTTAAGTTCAAGTTGTTATTAAAATTTATTCAATTATGTCCGTTTTGGATTAAACTCTATTTCTCATTGTTATCACACTTTATAATTACGTTTTATTTTTTTGTTTCGTTCTCAATTTGACACATATTTCAACACCTTTGTGATTTTTATGAATACATCTCTCTCCAAATACCCTTAGTATCCATAATTTAAATTTTGCTTTATTAATTATGTTTTTCGTTACCATCAAAATATTTATTTCAACCCTAAGTGCTTCCACCTTTAATTTACTTTGATTATTCAACTGTTACAAGGTATTTATTTTAGTCTATTTATTCCAAGGGAATCAAAATAACCATTCAATAAAAAGTTTAAAAACCAAAACGAATACTTTTAAAGTACGATGATTAAAATATAAAGTTAAAAATAAGAAAAGTCGTTTTAAATGACATAACTATCATATAGTGCAGTAGACTACTATTTAAATTTACCACAACACAAATGTCTATTGTGATTTTATTATCAACAAAATGTAAAATTTTGCCTCATTTATAAATATTTTGTTTAATTTTATTATATTTGAAAACATTCATAAAAATATATCAAAATACATAGTTTAAAGTTAATATTAATAAATATAACTTAACACTCAAATATTTACGAAGCGTCTAACAAAATTAAAAAACTCTGCGGATCATTTTTTTAAAATATTTTAGGTTTGTCTTTCATTTTCATCATTTTTCTTTCTCTTGTTGTGCACATCGTGTATACCAAATATAAAAAGAAATTGAAAGAAAGATATTGGTTTAGTCTAAACACCT

>Csa1M589750.1

TTGATGGCTGATCTCTTCCAGACTACCACCGTGTAGAATTGATACTCGGATTGAAGCCTTTTGCGATGTAGAGAGAGAAAGAGAGAGATAATTGCAGAGAAATGTCAAGAATTTGTTTATATATATGAATTAATCAAGTGTTGAGCAGTAGTTGAGAATGAAACTGATAGAATTTACAAAAAATCCAAACTCAAAAACCCTAATTACACCATTTTTGTGACACTAAGAGCTGATTTAAACATTGAAAAGAAAAAGAAAAATAGGGCTGTTCTTGGTTGGATTGGATTGGAGGATGCGGGACCGTTTACTTTTCTTCTTTTGAATCTTTGACTTTTCTTTTTCCTTTGATTTTTCTTACAAAATTCTAACTTTTCAAACTCCCAATATTGCAAAATCCAATTTAAATATGCCTCAACTACATTCATACCATATCAAATTTCTAACTTTTTAAATTGTTAGTGTTCAATTTCATCTATGAACCTAATACCTCTCTTCCCATTTAGAAGAAATAAATATAAAACATCGACGTAAGAATAACAATTTAGAGATTGTTTATTATATTGTCATAGATTATAATAGTTTGTGTTTTGTGATGCATGTTATTTTTGTTTTGATTATAATATTATGTGTTTGAGATGTGAATATTTTAGTTTTAGAAGAAAAAAATAGTAAACAAGTAATTAAAAAAATGATAAAATTCATTAATGTTTATCTTTATAAAATCTGAAATTTTGTATATCTAGTAAATATATTTTTTTAGTTCATTGTATTATATTTAAAAATATCCGGATAATGATGCATGTAAAGTAATAAAACTGCCCCAAGTATGGAGATTTGAAGTAGTGTTGATTGTAAATAACTTAAAAGTAAAAAATACCCCTAAACTCAACTTAAAACCAATTAGTCACGAAAATAACTAATGTATCTAATAATAAATTTGTGGGATCTTATAAGGATTGTGAGGTATTTACATTGTGAGATATTCATTCATTGGAATGCACCGGTGACGAATGTAGTGGACTCTACGATTTAGTGAAAACAATCTAAAAAAGAAATGTAGAAACTTAATGAGTCTAGAGGGAACTGTGAGACCAATTTACCACTATTGAGGGCAGTAGCCAAGTATACATAATGTAATGCAATAAATAGCAGCGGTAATAGTAGTGACAACAGTAACGATAGTAGTGGTAAGAGCGGGAAATAGCAAACAATAAACAACAATTGCAATATAGAAACAGTATGAGTATGTGGCTGAGTCGTGGAACACGCTCTTTTTAAAACGTTTCGCACATCTGCTAAATAATGTGCTAACAGATGGTGTCCGTCATTTCCAAGATAAAACAATCACTCGTTTATAACTGACTTTTCACACGAATCAGTCACTTGTATTCATTCAAAAACATACACAATGTTTGCTCGAAAAGTTATTTTTGGAGATAGTTTGGGAAGTAATTTTGTAAAACTGATATATATAAATGAGAAGTGAACACACAAGTATATATTGACTTTGAGGAGAAAACAGTAAAGAAATAAATAGGGGTTTTGATTGAGAGAAAATAGTGGGTGAATTATAACGTTTTGATTAGAAGAATCACGTAGAGATATAGACATATCTATATTTATCTATCTTCTTCCTCTTTCAAAAACTTAGGTGCCTTAGGGACCAAAACACACCCATCAACCAATGAGTATAAAATTTATTTGAGATTAGAATAATATTATAAATTCCTTTCACAAATTCTTCGTCCAACAACCATTGAACCAATATATAGCTATTAAACTTGGGACTCCATTTTTACGTAGTAGTAAAAGAAAAAAAATGCTGCTTTTACTTAGAAGCCCATACATAAGCCCAAATTTGAAATGGGCTGGCTGGGACCTGGAAGTGTTCTAAAAAGTTGGTGAAAATATGAGTGTGCTTTTTATGAAATTTTAGCACAATTTACAAAAATACCCTCGTAAAATGGTATGGAATTTATTGTAAAAAAAAAGATAAAAAA

>Csa2M000340.1

AAGACATCGGGCTTTTGTACTCTAATTTTATTTTCGTAAAATATGCAGTTTTTTCTGTGTTCAAAATTAGGCAAGTGATCCTTCTTTTGTTGGATTTTATGCTTGGGTGAACTTGAATCGTAAGGATTATTTGTGAAGTTTTATTAAATTATAAGGATAAGGGCTAGATTTAAATTTTCTTTTAATTTTAGGGCCATATTTGCAATTAAACTTTGTCTGTCTTTTTCACACGTCGCACACAAAGATTTAATACTAGAGATACTCATAATTAATACATTCCTCATCTCACATTTAATATTGTATTTTCTTCATGATCTCAAAAAAGCATGAGAAACAAAGTTTTATTCATTTTTTAGTACTATATGTTTTTAAAGTAATTTAAAATTTTGATTACACAATATAAAAATGTTTTCTTTTTCATTATTTCTTTTTTTGAGTTATAGAATTTAGAAATAGTTTAGAGATCGAATGTGTAAGTATTTGCAAAATCTATTTGATTTACAGAATCAGTAGAATTTAATTTTTCTATCAAACAAACTAATTTGACATGTTTCCAAAACAATTTTATATTATGTAACCATATCTCTGTTCTTTTAAAAATCATGTCTATATTGATGTACCAAAAATATATAGTATTACATCACTTACATCTAATAGGTTAGTAATTAAATCACTAGTTATTTTTATTCTAAATAAACTTTAGGCAACTAATTCTAGATTTAAATACAATTCATACAGAAACAAACAAACAAATAGATTGCACTCTAGTTTCTAATCAGTTGTTAATTTCCAAGAGATAGACATGATGTGGGTTAAATAAACAGTTCAAAAGGGGTATGGTATAAACATCAACATCATCATCTCTTGGACTATGATTAATATTTAATTAGAGGAGATTAAGCTATTTAACAATTTAATAATCGTAGGTGGAGACTTGGAATATGACAAAATTATAAATATATATATATAGTCTAAGTAAACACGGTAGCTATGGTTTAAACTTAAAAGGGAGAATTTTCCAATTACATTAAAGTCAAAGACACGTGTGACGTGTGAGAGACTTTGGTTATGAATGAATGAAGTAAAACCAAATACTAATCTCTAAAATAAATCGAGTTTGAAACACGTGTGAACTGAAATGTTTTCACACCACTGAAAAAGAAAGCACTACTAACTATATATGCTGAAAAGGAGAAAAAGAAAAGGAACGTTGAAAGAGAAGTGAAGAATATTGTAATTTTCTTTCTAAATTAATTCACATGTTTGGTAAAGATTACAACTTAGTAACATCGTAAGAGTATTTTATAGTGTGTTTGGATTACATTCATAAGTATTTAATGTAGAAAATAAATGTTTGAAAGCAATTAAAATAGATTTTTAAGCGTATTTTAAACAATTTTTAGAAAAAAAGTGTTTAAACAAAAATGAGTTTCTTAAACAATAATTTTTTTAAAAAATTCAATCCAAACAAATCTTTTAATGTTTAGTGTGCAAGTGAATTATAAATCTTATATTGTATGTTAGATGTCTCCGAGAAAAAAGAAATCTAGAGTGAACTTATTTTTTTTCTTATAATGTCAAAAGAACATTATACATAACTTCAAAAGAATAAAGTAATGTTCAAAAGAAAAGCTTTTACATTACTTTAATTTTGTGCTTAACTCAACTATACTCAAAATTGGAAAGTGTACTTATTTTAATAATTGGAAGTGTATATATACATATAAATTGGAGATTCAAAATAATGATAAAAAAGAAAAAAATCCTTTTTAAAATGAAATATATATACAAAATTTGGGGAAGTAAAAAGGGGAGAGGGAGAGGGAGAGGCAGTGAAGAAGGAAACGCGGGTTAAATAGGAAGAGGTCAGCGGTCGTCTTCTTCACGGCCGGGTCCTCCACCAGACCGGTCGACGCTATTCCTTTCCTTCCCCAATTCCCAATATTTATACTATTGGGTATTAGATAATAAATTTTAATTAATTGAAGGGCCAAATTGCA

>Csa3M839800.1

AGATAGAGAAACTGAGTACTAATTTAAATAATTTTTAAAAACATTTTGATATTATCTGTAACTAATAAAGTTTGTTGTAATTAACCATTAAAGATGATTATAATTTTTTTTAAATATTAATTTAGTTTTTTTTAGCATGAATTAAAAGAATTTGAACCTCAAACCTTATATTAATTGTGTACTTATTATTTTTGAATATTATATGATTGATAGTAGATTAAATTATATATGTTTTAATTTAACAAAAAATTGATTTGATTTCATGATCTTACACCGTGTAGTTTTAAATGTTTTTATTTTGTACAGTGTTATGTTATATTCCAAAATTTTCATAGAAAAATTAAATGAACGTTTTTTAAAATAGCAAATAATAAAAAAAATTCATAAACTTCTACTAAAATGATTTTTTTATTATATTTTGTAAATAGTTTTATATCTATAACAAATCTTGGAAATGGGAATATAAAATTTTCGTTTTTATAAATTGGATGTTGTTATTTTATTACTTTAGTTTGAAGAATATTTTATTATACACATATCTACTCAACCCAATCATTTTTAAAACATAAAATATAGTACACTCTCAAACTTTTTTAGAATCGTATCTATTTGTATTTCTATTTTCTGTAAAAGAAAAAAAACATTTAATACCCTTTTAAACTTTCACACCATATTTATTCCTTCCAATTTTATACCATGTTTTTTTTTTATTTTATTAAAATCATCAACTTTTCAATTTTGTACTTAAAACTTCTAACATTTAATTTGATTAGTTTAATGTTATGGCCATTAAATATATCAATGATCTATATGATTGAAAATAGACCCTAACATGATTTTGTTTTAAAAGAATTATAAAAAGTAACAAATTTAATAAAATATTTATAACATATAGTACAATTTTTCAGACTCTATCAATAATAGACATTGATAAACAGTAAAAGTTTATCGGTAGTTATCGTTCATATAATTCAAAATTTTATTATAATTTATTTTATTATTTTAAGAAATGATCATTTCATTTAAAAATTAAAATTAGCACGTGGAATAATAAATGATTGAAAGGTGTAACAAGTATGGACTTAGACATAATCTGTTTTCAAAAGATATTAGAGACTTCTGAAACTAAAACTGTTTTAGTGGTAAATATATCAAAATTTTAAAGTTCGTGTAACTATTAAAAAAAAAAAGTAAATAGGTAGATTTACTATTAAAAGAATTTAACAACTCACGTAAACATAATTAATTTGGTTAAAGCATATATCATCAATTCTTTATAAAATTAATTTGTTCTATAATATAATTCAGAGTTTATATTTTGATTGAGTTATTTTTCAGCTTTTTGAGGTTTTAAATTAATACTATCATTAGTGAAGATTTTAAATGGATATTTTCGTGGGAACAAATAACGCAAGGAACAATCCAAACTAACTAAAACGACGTGGTTTCAAATTGATATAGAAAGAGAAACGACTGATTAGTCAAAGTCGTAAATCATTTTACTTACGAGAATCCACGTTCCGTCACCGTCAAGAAACGAAGATTTTAAAATTAGAAAACTCCCATTCTACGGCGTCGTTTCCCAGGTAACCAATAACCAAAGCGTTCTTAACCGACATTTGCTTTGTTCCACGTGGCTGTATTATATTGGTCAAATCAAAGGCGGAGGCCAATAACAAAGCAAATCAGCAACCCGAATTTCTCGATTCGGCGCGCAGTGGGACATGCTCGAATCTTGGCGACCAAATTTATCGGAGATAAAGCGATTTTGGGGAATTTTTGAAGAGGTTTTTCCGATTGTGGGTTAGTGTTTCGATCGCGTTTTTTTCGCTAATTTTAATCTATATAATTTTTTTTTTCAAAATTCTCACCTCATTGAATGCTTTGCTCTGTTGTACATTTACTACATTGTTCGTTTTACTTTAATCTCTTACATTTAGTCAATCTTGAGCTTGAAACTGTGTTGATCACGCTGTGAAATAGTGTAGAAATTGTTCGAA

>Csa3M651720.1

CGGCTCTGATGAAGAAGAAGAAGAAGAGGAGGAATCGAAATAGAATAAAAATGAAAAGGAAGAAAGAAAATTGAAGAATAAAGATAGAGAAAATGGATCGAAGAAGGAAGAAGAAGAAGAAGAAGAAGAGATTGATTGATTATAGTGGGAGGGATTAAAAAGAGAAAGTTCTTGGAAGAGGGGAAAGGAGGGGGAAGAAAAATGAGGGAAACCAAATTAACTTTTTTTTTTTTTTTTCCTTTCTTTCTTTCTTTCTACAAAATGAAACGGAGGAAGGAAAGGGAAGGAAAGGGTAAGTGATGTGTAATATGTATTTAACTTTTATATTTATATATTTATATATTATATATTTTATATATATATAAAAAAAAAAAGAATGAATATATTAGATCGAGGGAGAGGTCGAAATCAAAAGAGGGAATCATATAGATATTATGGAGGAAGAGGGGGTGAGGGTTGACGCGACTGAAACCGGAGTGTACAACACGGACCAATGGCCTTTATTAATTATTGGAAACACTCCATTATTTCAGAATGGGAGCGTGTTCTCTTCTTTTGGACCTTTCTCTACGTATTTCGTATTTCATATTTCATTATGTATCCATCTCTCTCTCTCTCTCTCCTTTCGGTGCACTTTTCTTTTCTTTTCTTTTACATCTCTATCACTGGATCATACAATTATACCCTCTTTGTTTCTTTCTTTCTTCAAACCCACCAAACCCCTTTTTCCCAAGCCCAAGCCCAAGCCCAATCCCATTCCCTAACCCTCCCACTTTGATTCTTACGGAAATTAGATCGAATACCAGACACAAATGCTGATGTATGCCTAAATTAAAGGATATCACCTACTTCAACTATATAATCTTATATTTATTACCTTTTTATTTCTCTTTTTAATATAATTTTCCATCTTTTTTTTTTATTATTATCTTTCTTTTGCTTCATTCAAATCCATTTTCCTTTGTATTTTATGATTTTTTTTTACTATACATCTCCCTCTTAAAATTCAATAAAACCATTCAAGTTCCCTCCATGTCACACATCTATTCTTCTCTCCTTTTCACAAACTTTTGTACTAATTTCCTCCTATTTTTGCAAGCTTTTGAACCAGTCTAGGAGAGTAGATCAAGTTAGAGACAACAATCCACATGTCAAAACTACCATCCAATTACTTACTCAAGATGAGGACATGATTCAAATTGAAATTCATTTTTGCAAATGGATGAGTTCAACAGGGGTTTGCGGTTAGGTTAGAAAAGGCGTGAGGTTTTTCTATATTAAAAATGTTTACAAAAGTCATATGCTATAAAAGGAGTTACGTCTTTGGTTCTACACGGTCTATGTTGGAAACACTTGGCTTTAGTATGCTAACTCTAATTAAATTATTTTTGTACTCTTTAGTTGGAGAGTGGAAAAAATGTGTGGGTAGCAAAAAACTTTGAGAGGGACTTCTGTAAGTGAAGTCATGATAATAAAAATTATTCTTTAAGATTGCAACAGTTTTTCTTCGACTTAGAAGTTTTTGTACCTAGATTCTTTGTTTTTAGTTTTCATTGTTTGCTTTATTAATTCCCTATTATTTTCTGCCTAATGGGGTATTAGAGTCATGCCTCATACTTAACCATGCAAATAGAGATCTTCAGGTGTCGAACAAAGATATAATAAAGTCTCAAAAGTAGATCATGATGTGATATGTGGTTGAACTCTATTTAACAATATCATACCATGATGGAGATATATAGAGAGTATGTTGTACTAACAAATAGTATTGGAGCATCAAAATGAGAAGCAGATAGTGTCATATGTCGAGTTTTATAAATGTTGCAGAAGCTATGGATTCTTTGAATATCTTATCAATGTGGAAGTGGTTGATTTTTTGTTGTCACATAAAGGTCCTACGTTGAAAAAGTTTAAGAAAGCCATGAGTATTTCCTATCTTAAAAAGGTTTATGGAAGCCATAAACTATAATAGAAAAAACTATGCCTTTGGTAATTTTCAA

>Csa2M000780.1

CCAAATATATCAATCAAATTCGAAGTATTAGCAAATCTAACATGAGGTAAAAAAAAAAACTGCCAATACAGCAAACTTTAGATCTAGCTCTTGGAGTCCTAAAAGTTCTACTTATTGGAATTATTCTCTTAAAAACTAATTATCTCTTGATTTTCAGGGCCCAGGTGGGGTTACTGTTAAGAAGACTGGTCTGGCTTTAGTCATTGGTATCTATGACGAACCAATGACTCCTGGTCAATGCAATATGATTGTTGAAAGGCTTGGGGATTATCTTATGGATCAGGGTCTCTAAATATCGGTTTGTTTCCTACTGCTACACTCTGTCCAAGTTCTTATTTGCTTCCAGCTTTCTCTCATTGCTTATACGATATATCTTTTGGATTGCTGCTAATTTCAGGGAAACCATGTATTGTTTGTACAAGTCTATGGACTCTTGAATGGAAATGGGTTTGATTGATGCTTGAGGAGTGATGCTTATGTTTCTCGTTATTAGTGTTGTCTCTTTCACTTTTTTATTTTTATTTTTGTCCCTTTGAAATATTGTGGTGGTTTTCAAGTGAGGTCCATGTTGTAGCATCAAATATGGATTTGGTAAACCAATCAGCATAAATTTATAATTACTCTTCTCTATTTTATCTGTTGATTTTTGCAGCGGGCTCCTGTATAACATGAGCAGATATATAGTTTCTTTTAACCCAAATGTGGGATGTTTGCACATTTTAGTCCATCTTGTTCAACATGAATTCATTTCCTTTTCTTCTCTTCTATTTGTTATGATTACACTTCTCCACAACACTTTAGAGCTTAATCCTGCCAAAGACCTAAAAGCAACAATTTAGAGTGTATGTATTCTTGTAGTACTGAGAACGCACGTGAATGCTTCTATGTTGCTACCAGCCATTGTAACAACAATTTTTTTTTTCCGTATGACCAATTAACTTATGGTTGTAGACATTTTATCCGTTAAGATGTACTCAAGTTTGCAGCATCGTGCATTAACATACCCGCCTACCGCTTTTGTTTCAACGTATGAATTACCTTAAACTATATCTTTGGGGAGTAAAATATGGACAATCTTGATTGTTCTTTTCTATCAATTTTATTATACATTCACCACTCATTCTAAGAGGGTTCAACTTCTTAAAATTTCTTGAGGTTTACAATTTCAATCAAATAAAACTTTAGTAAAACACAATGATAATATAAGTCAATATCTCTTAGGATTTTATTTTCAAAATCAAATAAAACTCCTTAATAAGATGTAAAACATAATAATAATATAAGTCAACATCTCTCTTAAACAATCTAAACAATCTATTGGTATGGTTGGATTATATTTTCAAATGTAAATAAAGTTATTTTAAAAAAAAAATACGTTTAAAATAGATTTTAAGTATATTTTAAATCATTTTTAGGAAAAATGAGTTATTTGACAAACATTTTTTTCCTTAATCCAAACGAATCTTATATCATTTTAAAAAAAATTGATTGTGTGAGTCAAATAGATGTTGATTGCTAGTGTTAGGTTGGGCGTTGCAATACTTGCCATTGGCTAAAGCTTCCAAATGAAAAGGTGGGTCTGTCAATGATACCTTGTGATAAAGTCCAAATATCTCATCATAGTGTATGGGCCCTGTGATCGGTTTATATGAAAGAAAATATGGTTTAGTGAAGAAGTGGGGATGACTATGACTTTTTTTTTTGTTTGGCTGATCCGGTTTGGTTTAGTTTGTTAAAGAGCGGTTCCGTTTGGTTTATTTAACCCCCCAAAACCGAACCGATTTATATTCCCTAACGTCTATAATAATATTTACGCGCTCCTCAATGTTTTGAATTTCTTTGATTTATATTCTTTTTGCTTCTTTGAAGAATCAGATCGTCGCTTTCCTCACTGTCTCTATAGACAAAAAGAAAAAGGAAAAACCTCAATCCCACATTTCTCTTCTTTCAATCTCCTTTTCCTCACCAACCGCTATTCAAACCCTACGATCTCATCAAAG

>Csa1M042980.1

AAGAGAGAAGGGGAGGGAAGGAATTGGAAAAACGGAGGATTTAGGGAATTAGGATAAAAGTGAAATGGGGAAAGGAATTTGTATAAATAAGTGAGTGAGGGGAAGCACGTGGGAAAGTGGAAGAAATGGAGAAATTAAAGGAAGTTGCCACGATGGACACCATCATTGTGGGCTGTGGTGAGGTCCGGCGTCGTTTTGATTTAAAAATACAACTAACTACTTAATTAAGGTTTTAATTAATTAATCATAATTCTATTATTACTTTATATTTAGCCCAACTCTAATTAACACAGCAATATTGGGTTTTTTATGTTAATATGTTTAATTATTTATTTTTTTGTGACAAATTGACAGTAGGTATAGTACAATTATTATGCAAAAGCTAGCCATTAATTTTCTAATAATGCATATATATGTTTCCATTAAAAGAATGGAATTTTCCATCCTATTTTCTATGTTTTGTGCATGTTTAAATTAAGAAAAAGTAGGTGGGAAATGTGATTTAAATTTGGCTTAAATATTAGTTTGTTTCATGTTGGTTGGATTTTAGGGTTTTCTATTTTCTTTTTTTCTTTTTTTCAAATTCTTTCACTCATTATGATATAATAGATTTTCTATTGTTAATATTACAAAAATATTCACGTCTTCTTGCGAGAAAATTATTATTATCATTTCAATAAAATTGACTAAAAACTAGAATTGGGATTAGATCAATAAAAGAACTAAAAATAAACAAATTTGAACTTTATCCCCAACACTAATATATTTTTTACAAATACACTTTTTGAGTTTTTTTTTTTTAAATGGCTTATAAAAATTTGGAAGCCAATATATTTTATTAAAAACTAAAGAAATAGTTTTCAAGCTTTTTGTATTAATTTTAGATATATAATAAAAGTGAAAGTTAGAATCGAATAATTGATAGACAATCAACATAAATATAAAAAAAAGAAAAATTAATAAAAGAAAAAACTTACCAAAGCAACCAAATATTACCTTTTTTTCAAAAGATGTATATATTAAACAACATCTATAAGAGAACTCCACAATCAAAGCCAAAGGAGGAAGCAAATGACACAAAAACAAGCTAGATGAGAAGCATAATACAAATAGAAGAATCAAACAAGAACATAGAACATGACAAAACAAACCCGACTAGAGCAAGCCAACAATATCCCCAGTCCATAAGGTATAGCACCACGATCACATGCAAACAGTAGAAAAATGAAACTTCATAACTACAAATGAGCTACCAGCAAGTCGATAAAGACCATAATATTAAATAAATCATTAAAGAAAAATCAAAACAAGTAAATAAATAAATAAATCTTGAAAGAAAAAAAAAAAGCTTAAAAAATTAAATGTTAGGGTAAAAGATAAAAGGTGTCACAAAGATTTGGTTGAGTAGATAAGCTTAAAGCTTGACTTCATTATATCACGTGAGGTTATTTTCGTCAATTTCAAGAAGCCGCCGACGACGTTTTCCCTAAAAATCGCCATCTTTTCCTCCGCTAATAAATAAATAAATTACCATAACCTTTTCAACTAATAATATAATAAATAAATGAGCAAAAACCTACCCATAAAAGATACTTGTAAATCCACCTATTATTTTAACAAATGGCAAAATTTTTAAAAATATCTATAAATATTTTGTTATTTTACTATACTTTTTATTTTTCAGATCAACGAACAATCCCAAGAGAATGTCTTAGTTAACATGAACTTGAATTCATTTATAAAATTCAAGTGATCTATGATAGCTGTATACAATTAATTAACAACGTTTAATTAGGTACGTAAGAAATGATGAACATATTAAAAGATAATAATATAAATTGTGAATATAATGACAGGTAGATAGTAAAATGTCACATTCTTTGAATGACCCATCAATAAGAAAGGATGTGGAAATATGGGGGAAAGATAAAGACACATAAATGTATATGGGGCAAAGACAAAGAGGGATGCTTCCATAGTGGAGGCTTTCTCTCTATACA

>Csa1M042730.1

ACTTAGTTCCCAACATAACACATTTGAAATTTTACTAATACAAGAAATATCGTTGTTATTTTCTTTCCAACCACGTGTCATCAAACTATACATACTCAATAGGCCATAATTATTTTAGGAGAGTAGAGTGATTGTAACGATCATATACATTCAATATATTTTTCTTTCTATAAATTAAATTAATGACAAATTTGTTTGACTTTTTAAATTTAATTATTGAAACATTGCATTATATTGTGTTTTCAACCACTAAAATTCACAGAAACCATCTCCCCATAATAATAAAGAAATAAATAAAAGAAACTTCATTGTTTACACAATTACAACATATGAAAAGAAGAGGGTCGTCATCATCTATCACTTAGAAGTTTTGCAAGTTCCACAAGCATGAAGATGTGATATCCAATAGAATCAAATGTCAACCATAATGTTCAAATTTAATAATTTGTATTGAGGCTTACAGCTGCCCACATATCCTACCAAAGTTTTTTCTTGGTGCACCTTAGTTCTTAGCCATTCACTCTTTTGTTGCACCAATAAATACAATATTTTAAGATCAGTAGTTACAGTTTCCAAATTCACACATGTGCAAAACCAACAAGGGACTGATTTTTTACTTAATTATGCCACAAAGAGGAGTCAGTAAATAGTATTGGGCACCAAAGTATTGTGTTTTAAGCTCATTAGTTACACTGCCCAATTTCATATGTGCGCATAAAATTAATTAAGGGGCCTTTTGATGATAAAAAAATAGGAAATTTCCATACAATTTACTAAAGAAAATCAAACTCTTGTTGAGACCCAACAAATAGATATCCACAAAGAATTGAAACCCAACATGTAAATATCTGTCTGTAAGTTCTGGCACAAAGGGTAATGAGTAAAATGAATTAGGCAAAAGAGAAGAATAAAGGTCAAATCGGAGTTTAAATGAGGAAAGATGGATGAAAGTCTTTTGGTGTTGGCTAAGCCTAGTAGGTACAGCCACAGCCAGAAGCAGCGTTTTCTTTTTTTATCATTTCAAAGTGTGAAACTGATTTTGGAGTTGCCAGTTAAAACAGTTTTTTGCTGTGTTGATTATAGAAGAAGTTGAAATAAAATTGGAAGGGATGATATCCCTTTTGCTTGAAATAGTAAAAAGTGGAATTCAGCGGTCTGTTTCTCCAACTTGTTCATTTTTATATTCCATTTATGCGCAGTCCGCCATAATTTTCCAACTCAGACCGTCACACTTGGAATTTTGGATTCCAAGAAGAGATAGAGATATAGTAGAGAAGCGATTTTGCTGGGTGCTAAGAAAAATTTCTTTTTCCCCCCAGTTGGGTTTGTGTTTTCACAACAGTTGAGAGCCATTTTTTCCCCTTCTGGGACCGCGAAATCCTTCTCCGTCTCTCAAACTCAAGTGGGTCACTCTTAAAAAGGTCCTACTCTAACCCACTTGCTTGCTTCCGGTGTCTTAGCCTGCGTTCACCTGCCCCTTTATTCAACCTTTCTTCCCACTTTTCAATGAACTACACACCCTTTTGAGTCTGAATTAAGATCTATCTATCGGGGTTTTCATTCGTTACTCACATTCCGATTGCGTTCTCAAGTTGGTAATACGATTAAGACTTCGATTTCGTTATTGCTGCTGGTGTTTATGTGTCTGTTCTTTGAGATTTAGTATCTGTTTCTGTTTTCAATTCGAGTTTGGAGTGTATTCGTTTGTTAATCACAATTTAGGGAGTTGTCGTTCAATATAAGCATCTGGGTTAGTCGATAATTCTTGCTGCTTGACTTTGAAGTGCTTGAAATTTGGTTATGGCTTCTGGGTTTGACGTGATGGTTTAAGTTTAACTCTGTGTTTTTAGCAAAAGGGGGTTTTCGTTAATGGTTTGAACTTTCGACGACTTTTAGTGGTGACTCAGGGATTGAAGTTTGATGCTAATTCTAAGACAATGGAGAGGCTTATGGCCGGATTTTTACTCTAGCATTCGAGTCTTTGACTTGGTGAAGCTAAT

>Csa1M046040.1

TTCTAAAGTATAAAGAGACCTTTGTTTGCTTGTAATCCATGAGGATGTAATCTAAAATCTAGGTTTTGGAGGATATTTTAAATGCAATTTATTTTATTAAAATAATTTAACTATCCAACTTATATTATATGGTTTGGATTGGAATAAAATTTTGACCGGGTTTGAATCTAATTCAGTCAAATGTGAATTAGAAAAAGAAAAGTAAAGGATGATTTAGAAATGTAAAGAGAATTGAAGTGATATTTGAGGGGAGTAGGATTGGTAGGCTATACAATATAATTAATATTGTTGTTGAAAGAAAAAGGGTTTGTGGTTTTGGATGAGACGGGGGGTGAGATCATGTAATGGACACGCGTTGAAAAAGTTGGTGGGAGCCACTGTCCAGGGCAGCTTTCTTTTTCTTTTCTTTTGTTTTTAAATTAGATGAGAGGTTAAAATAAATAAATAAATAAAAATAGAGAGAGATAGAGAGAGAGAGAGACCGTATACGGAAGAGGACCATACGGCTTTGTCCGACAAACCCACGGATACCTTCCTGTAATGTGGTCCCCACTCCTTCTGTTTCCGTTACTTCACTTAACTACCTCTCTCCCCCTCTCTCCCTTTTTTACCCCTCTCTTTTTCCCTTTCTTCCCCATTCTACCCCCTACCCCCTTCCCTTCCCTTCCATTCCCTTCCCTTCCCTTCATCCATCTATCTCTCATTCTTTTCATTTTTCATTTCTATTTCCTTTTTAATTTCTAATATATGCATTTCTTTATTATTATCTAATCTTTTTCCAATTTCATCCATTTTGTTTTTCCCTCTCTTCTAACCTTATATCATCCTAAAATCTATCCTCACATCATTATTACTACTCTCTGTATTGAAATTTCTTATTAATTGCTTCTACATAGTTTTTTATATGACATACAACAATGTGTGAAGTGTGAAGTGTGAAGTGTAAACTTCAAACTTTCTGATTTCTCGGTGGAGGATAACTTGATGATTTGATAGTTATATAATCAGGTATTCAACAATTAAGAGTGAAACAACAGAGGTCACATGGAGTAGGCATTTTTATTATTTTAATTTTTGGAAGGGTGGTAAAAATATGGGCTAATATATTTACAATCATAGACTCAATAATTAAGAAAGGATGTACTATATTCATAATTGTAGGTACATATGGGGTAGGGATAGGAATTTGAGAGGAAAGGGGAGGTGAAGATGAGGGGCAGTATGGAAAAAATGAAGAAGGTAAGGGGTGAAAGAGGGAGAAAATGAAAGGGTAGGGGTCATAATCAAGAGTGGACCCATAATGTCCCCTAAGAAGAGGATCTTTACACGTTATGAATGTAAAGGGGCATAAACGAAAAGAAAGGTATTTCCATTAACTTTGGCTTTGCCACAACCCCCAAGAAGAAAAGAAACAAGCCAAAATATGGCTTAACAAGACGAGTCTTTCAAGTCTTTTAAGTCTTTTACATAGACACACAGACAGAGAGAAACCCGCCAGCTCTTCCCCCATATGCTTTCACTGCACCACCAGAAACAACAACTACATCTCAACCGACCCTTTTAGCTGTAAACCCTCTCTCAATCTCTATGAACAACAAAACCCATCTTCTTCTTTTCTGTTTCACCTCCATATTTCTATTCTAGTCTTTCAAATACATCACTCTTTGCTCTTTTTCATCTTAGTTTGTGGTGTATGTGTGTTTAATTTGAATGATTCTGAGGTGGGTTTTCCTTCTTTTGTTGGGATGACTTCCATTATTGGTCTGCCTTTTTCTTCTTTCTTTCTTGCATGAGTATCAACAAAGGAAAATGTAGCTAAAACAGTGAAACCCATTAGACAAGAGGTTAGTTCTGTTTTCTGTAGCTATAGATCTCGTTCTTGCATCGATTGATTTAAGCAAAATTTTTAGTGGATTTAGAGTCTACATTGGGAAGTGGGAAGGATTCAAGTGAGGGAAATTCATGAGATTGATGATATTCGTAAGATTTGGCCACACCCC

>Csa1M046910.1

TCTCGTCTCGGATCAATAGCTGAATAAAAAGAAACCCAAGTCCCTCGAACAATGGAGAAACAAAGTCTACAGATTTTCAATACAACCCCACTTCAAAAGCACCTCAAACAATAACCAATGTCGATTCAAGGAAAACCCAAAATAAAGAAAAAAAAACTTTCACCAGATTCGAACAAAACCCGAATAAAAAAAAAGGTTTTTAGGATCAATGAATAAACAAAGGCAAAGAGAGAGAGGGAAAAAGGAAGAGTTTCGTCAAAGAGGCGCTTATTTGGGTTGGAGGCTGGAGCGGGATTAACCGACTGTGACTGACCGTGACAGATTACTCGCCTATCCAATTTCCAATTTCCAATTTCTCTTTTTATTTGTTTGATTTTTCTCCAAATTGAAAACTAAAAAAGTCAGCTTTTTTGGCTCATCATCCAAAAAAACAGAATCCTAATAATCTTTTGAAAATATTCTGACGTTTTAATCTCATCTTAATCAACTTTTTATTTTTCAATATCAATTTTTTTAGTGTAACAATTAAGCATATAAGCAAAATATAAGATCCCTATCAAAATGAAAAGAAAAAAAAAAAAATCTTCATACACAATTTCTATTGGGTCAATTCTATCATATATTAATCAAAGGCTATGCATATAAGTCTCATTATTGAAATAGAATATTCATTCTACTATTAATTCGACCCTTATTATTGAAATAGAATATTCTTATTGTTTTTTTTTCCTTATATCTATGAGTATATATGTTTGTGGGGAGTAATTGGACTATTTTTATGAGATACTGACCTAAATCTAACTGCATTTAGTTTATGATGACATCTTTTCCAAGTATTATTGACCATTTGGCCTAAGAGATAGTGTTGAATAGTTTCAAAAATATATCAAAATATTACAATATTACAATATATTTGATATTTTGCTTTACTTGTAAACATTTTACAAAACAATTTGTCAATTTAAAATTACTTTAAATACTAGTTTTAAATTACTATTGTGTTAGCTTTGATTGATTACTCTTTGAGTATGTTCATAACTTGCATAGCTGTTTCCAATTCAAAACAATCACAAATACATTTGGCATGGAATTATACGCAGTACATACTTTTTACAAAAGCATCATTCGTGATACCAATTACGAAGTACAAAAATCTGAATTTCCAGCATCTAGTGATAACAAAGAACAACATAAATGGATGTTTACGAATCTGCACTTCAGAAGAATCATCAAAAGGCTCTACTGTTACTACAAATTCCTAATACATTGTAAAGCCAAGAACAACATATTTTATGAAAATGTGGGACAAAAACTCGGATCCATAGCCAAAAACTTGAACCAATATACTAATCATCCAGATATGCATCTCTGAACTCAACTAATAAACTTCGAAAACAAACCAAGCCAATGTTATCTCATTTCGATCCAAAACCGAACCATTTTGCCAAGAAGTTTTTACTCTTTCCACGGGAGGGGTTGGATGCTTCTCTAGCTGGTGTTTGAAAAGGTAACGAACTGGGTTTAGCTGCAACAGAAAGATTAGAGACAGTTTCCTTGTTAATAAAATCTCTTATCTTCATAGAAAGCAATATGAACATTAATATTCAAAAGGGATATGGTGAAATGGTGAAATAGTGAAATAGTATGCTTTTACACTTGTACCGGATTTTGGCGAAACCTTGGAAGTTTTGGCAGTTCCAATATCACTAGATTTCTTTTGTTGATTAATTGCATTACCATGGGGTGGGCTGGATGGTTGATTCCTCTCCTTGATATGATTTGTATTCCCCACGTGGTTGTTGCTACCTATAGAAAGGTTACAAAATCATATGTAAACACAAACTTGATACAAGAAACCCAATTTAAGAGATCAGAACTATGATCAGAATACTGGGATTGCAGCAACAGATCAGAGAACATGATCAAGAAAAAGTTTGTATGCAAAAAACTTTCAAGATGAAAGAATATGAAAGCTTACCATCACTGGGAGGCTTTCCAT

>Csa1M057040.1

CTGTAGACATTATTTCAAGACCCTTTATTTGTTTCGGTTTTTAATATTTTATAGCGTCTTTACTATTTTACTTTTGTCATTTTTGTTATTCTTTGCTACATATTTACTATTTTATCATTAAACTATTATAACCTAATCATAATATTATAAGAACACGAAAACTTACTGATTATCACTTATTAGTAAGGACATGCCATTTTTTTCTTAAAATTTAGAGACTACAAACTACATAATTTAAGGGTAATTAAATTCTTAAAAAATATTAAAGGGTAATTACAAAGTTCTAAAAATTGGAGATGTAATCCAAAGGTGTAGAATGCCATTTTCCTCCTAAATGTAGTGTACAATTAGAAAAATAGACCAAAATATAAAGAAAGTGGAGACTTGGAGTTTGGGAGGATAAAGTACAAACGAAGGCGCACACTCTCGTGGAAAAGAGAGGGACGACACAGACCTTCTTTGTTTTGCAGCAAAATTCAAATCTCACACTCCATTGTTGTTTTTGGGATCTGCAGGTAAGTTCTCGAAACGGTGTTTTCTTCTTCTTCTTCTTCTTCCCGTTCGTTGCTCATGACTTTAAAACGGTAGCTTGATCATCTCCGAAGCAAACTAATTAAATCTTCGTATTCTGATTTGTGATTCGATTTATGATGTCCGCTTCTCTAATCCGACATGCGATTCGGCATTTGAGGTTGATATTTGATACATTTCTTGGGTCAATTGAGCTGGTTGATAGCTAACAGTTGAAGAATCGAGTCTATTTTATGCGTGTTGAAGGCGTTGGATGAGACGGCTGATGTTATGCAATCTGGGTTAGCGTCGGCCAGCTTTGGGAATATCTTATTAGCCTACTCAATTGGGTTTTCGTTCAGGTTTTTGCTTGATTGGTTACGAGAGGCTGAAGGCTTACTCGGTCTAATATATACTGTTCTTCGGTATATTATTGTTACAGTTTTCTAAAGATTCGAAGGGCGTTACATAATCTTGAGTTTTTCTTTCATGCATCTTATTGCCCATTAACGGACTACACAAATTTGTACCGAGAAAAATATTGATGCTAGAGTTCTCTTATACAGTAAATTTTATTGGAGATGGATATAGAATTCCACTCGAGGTGAAGAGAGCAGAATGGTAGGAGAATGGAACCTCTGTTTAGTAGCTAAACTACATCTTTTCCCTTCTAAACTTTGGAGTTTAAGAGTTCAATCTTTCCCTGAAAGGCATCTTAGTAGCATTGTTGACGCTCACTGAACACTTTGTTTCTGCATTACGTATTTGCATCACTTGAACTAGGAGCCACAAGCTCGATCCCTGTTATTGATAAGAATGAATCTCACCATTTTATTCACCGAAACATCGGCGGCACTAGCATTTCAGGTGGTAAGGAAAGTTCAGCAACGTCCCTGAATGCTCCTTCAGCATTCTCGAGCCATTGCTTATATTACATATACTATAGTCTATTCAAGTGGAATGCCTATCACAATTGTCATTGCCCAATGGAGACCGAGTTAACACATATAAAATGAAAAAGTAACATAGTTAAGATAAAACTCTTATCAGACGATATATCTTTTTGTTCACTATATTATTTATACTATTAGAATTTAGATGTATACGATTATACGTGCCAGTTTCTACCATACATGTGTGCTGTTCTTCATGTTATTGTTATCTGTAGCCCAAATTATTATTTCAGTAAAGTCGGTATAAATCTAGGGCATGGCCCTCTGGTATGAATCAAGCAAGCTGATTTATGATTCTCTTCTATTCTCTCTAATTGTTGACTATCCCACCATACCTTTTTCTAACTTTTGAATACATGAATCCCAGGTTTTCAACTGAGTACTGAAGCTCTCCATTGCCGGACATGATTACCATCTGCAAGATGGTTATCTGAACGCTGAAGCTTACTGCAACTTAAAGTGCATGTTTTAACCAGTACGCAGTGTGTCTTATTCATGGCATTTTCGGTTGAGTTTGTCACCATCAAGTCTAACTAG

>Csa1M074900.1

TAGTTTTTCTAAGGCCTGAAAACACAGAAACCAATCGATCTCGTACTCAAAAACCTTTATGGTTTTCTCCTTCTGCTTCACCAGATGAGAAGAAGGCTTGAACTCAAAAGTGTAATTGTGAGACAAATTATTTTGAATCATACAGCTCCAGAATCCCAAATCACAATTAGGAAAACAGGAATCAACAGAAACTCTTCTTCTTCAGCAAGAGCTAATCAAAGCTGTGAAGACCGATCAAGAACAAGATCGTTTCAACCAATAATCTCATCTCCATCAAAACCAAAATCCCTTGAAACCCAATATATCCCATGAAACACCAAGCGAGAAAAGAAAAAAAAAAAAGCCTCAATCTCGGAGCACCCAAATCACAGTAATCTCTCCCCGAACCTTCCGCCACATTGTCTGAAAATTCAACAAATAATTCCTCCCCCTGTTTTCAAACCTAAACTTCTAGCTTCAGAAAGAAAAGTCAAAGCACTTCGCGTTCCTACGTCTCTGAAAAATTCCACAACAGAAATCCAATAAACAAATGGAAGAAAAAAAACAGAGAAACCGATTAATAACAATACCCCGTTTTCCAACCCCACCAAGAAAGCAAAAAAGAAAAAGAAAAAGTCTATCCTCAATCGCATTCATACTAACAACAACCCCAATCCTTGTTCAAACACAAAAGAAAAGTCATATTCGAAGTTTCCCACAAGAGAAAATGCAATAAAGAAAAGACCCACAAAAACGAAAACTCACCAAGAGATCAACAAAAGGGTGGATCCACCCAGCAACAATGGAAGGCAAGCCATGAAATTATGAAATGGGGGAAAAGAGGTATATACTTTATAATTTGAAAAAGGAATGGATGAATATAGAATCTTATGGTTATGGGATTGGGAATGACAGATGTTAATTGAGATGGAGAAGAAAACAAACAGACAAACAAACAAAGGAACAAGAAACAAAGAAAAGAAAAGATCAAGTGTGGTACGCACGCAGAAGAAACCGGTGTTGGTTCTCCGAGCATCTCGTGGAGCCTATGCCTTTTTCTCCATTGTTAAAAAAAAAAAAAAAAAAAGTTCAACTGCGGCGTGGCGGCATGAAGGTGTAGGACCCAAAATAGCCACAAAAAGTGAAGGGGAAAGTCGACGTGGCAGAATCCTATGGAATTACAAAAATAAAAAAGGTCAAACCTTGACTCTCTTTCGATTTCCGGCGTGGCACCAAACCAACCCTTCCTTACCGCTGCCATCAAACTGCAAATCAATCAAAACTATTCCTTCTCATTACGTCCACATGTGGTTTTTTTAGATATTTGGGTGAACTCTCCTCTTTTCTGTGCTCACATCACAATTTTTTTTAAGAAATTAACCAACTATTAGTTTTTCAGTTATAATCAATATAAATCGCAAATGTAATGTAGAATTGTATCACCAAATATTAGTGTTTTCGTTTCATTTATCTTTTGTTTAATATAAACTATATTTTTTTAGATTAAAGTTTCTTTAATCCCTTGAACGTAAATTGAAATGTAAATTTGAAATCAAAATGATTAAATATATTTTATAGTGATTCAAAAAATGGAAGGAAAAGAAAATGTTGTTTTAACAAATGGATCTGAGGGTTTGGGCTTGGCTGTTGTGTATGGGGTGGAAAATGGGCTTGGAATGTACGGAATTGGATCCATCCTATAGTCAAATATGAATTGACATTATAACATTATAATATATGCATTAAATGACCTTTTCCTTTCTTTATCTTTTTTTGTGTGTGTTTAAAGTGAAGCATTAAATTTCAGATCCTTACGTAGCTTTAAAAGGCAATAACGTTGAATTGAATTTCAAAATATGAATGTGGGTACTGAAATCATTGATCTCGAGAACGCATTTGGGAATGCATTGCCATTTACTTTCGTAGGATTGAAGACAAAGTCTTTTTTTTTTTTTTTTTTTTTTTATCTATAAACCAATAGAATGAATTCAATTGGAAAAACAACAGAGTTTAATACGAGA

>Csa1M467120.1

TTGTTTCTAAAACCCTAAGTAAGAAGCAGTTCTCTGCTTTCCGACGAATCTCCGGCAATGAAAAACAATATTTTCGGTCAACTAAATTTAGAAAAATGAGTTGGAGTATCACAATTTTACAGCTAGGTTTTGGGAGTGAAATGAATGGAAGAATTCCCGGTGAGAACAAGAACTCGTTTTTTGTGTCGTTTCAACTTTCAGCAGCTTTTTGAAGCAGTTACGATTCCCCTTTATAGTGGGAAAAAGAAGATGAAGTCTCTTTTTCTCGTCGGTACGGCGTAGTATAGTAATTACGTCATTAAGTTCTTCTAAAATACGCTTCCCTTTAATTTCGCCGTCCTTACTTAACGGCTTCCGTAACTTTCGTCACTTTGCGTGACGGCTGGATGACGACGAACTTGTTCTTTTACGCGGATGGGTGTTCTTTTCGTGTGGGGAGTGATTATTGATTAAGATTATGAATGCAATCTACACCGTTGATTTGGTTAATTTTTTTGATTTGAATATTACCTACGGTTTGGATTAAATGGTATAGTGGATGTGGCGCGGGCGAGATAACCGATGTTGTATGAATACGTCGTCGTGTTGGGGTGAGTAGATGCCAACTTGGCGGTGACGTATTGAGTGGAGCCCATGGCTGAGGTGGATTTGGGAAAACGAAAATATCACACTGCTGGAACATTCCAACTATATGATATAAAAATTTATTTGATTAATAAATGAAGCCTTTTTTTTTGGTTATTTATGGTATAATGTAATCTTTGATGTAAATGTGTAGGATAAGACTAAGTCTATTTCAATCCCATTTTTTCTATCATCGATTTAATTTAAGGATTATCTATTAAAATAATGGAATTTAATGGTTTTCAAAATGTTATATTTTTAGAAAAATTTTAAATCAATCAAACTTTGAGTTTTTGCTTAATACTTCTTCTTTTTATTCTTAATCAATTAATTAAAAATTCTTATGATGGGGATCTTTTAATTGAAATTAATTTAGTTATAATCAATTGTACTTTTATTAGATCAAGTAATACACATGACTAAAGCAGTAATAATGAGTAGAAATGTAAAGTCTTAAATAGAAATTATAGCAAAATTTAATTAGGATGTTAAGTGACTAAATTAAAAATACAACTCATGCAAGATGTTGAAAGCTAAAGAAAAATGTAAAACTAGAATTTTAAAAAGTAAAAGGAAAGTAACGAAACAAAGAAATTGTGTCTTTCAAAATATGTTATTTGTTATGTTGGTGTCAAACCCGTAAGGGTAATGCAAATGAGAAATGGGTGAATTTGAATTTATAGAAAAGAGTGAAGAGGATTGAAAAGAAATAGAGATGGAATTGAGAAAGAGAGGATGAATGAGAGGGGGGAATGAATTGATGAACTGTGTTAGCGATTTGTATAATAGAAAAGAAAAGGGTTGGTCAAAATAGGGCAATTGTTAAGCAATGATTTGCAAGTTGTGATGGGTAGATGGGAAATTCACATGAATTATGGCCGCCTTTTTCTCTTTTATTGTTTAATTTTATCCATTCATATATTCATATATTCATAATATTCATAATATTCATACATTCAATATTAATATTACAAATACAATGTTTTCCCCTGATCATCATATCAGCCGCGTGAAAAATGACATCTAACACATATTCAAACTATTATCTTTCTTACAAATAAATTAGTTAGCCACCAAAATTTCCTTTTTTCAACGGCAATTCTCATTTTTCTTCCTCCTTTCTTATGAATGGAGGTTTGATTTATAGTTTCTCATCTGATATTAAATATTTAAATAATTGATTGTGGAGTAATGTATAGGGTTGATATTGTACACCATTAGTTGAAAAGGGTATGTGAGAATCTGAAATAGTATACTATTTGTACATTGGGTTATATATAATAAAACTTTTTTATTGATTAGGTTGAAAATAATATTCCGTAAATATAATATAAGATGTTCCCACTACTTGACATTACTCTTTTGGATAAAACAAA

>Csa1M532310.1

TTAAGGTTGATTAAATTAATTAACAAAAACAATTAATATAATCCTTTCCTTTCTTAATCACAAAAAAAGTTTTATTACAATCCAACTCGAACATAACTAATTGGTTAAACATACGACATCTCTATTTAAAAGAACGAAGAAAACTAATAAATAAATATTCAAATACCATTTTCTTTTTAGATCTATAACCTTTCGTAATATATTATGTCTTCACCGACCAAAACAAAGAAGTCTAAGGAAATTTTTTATGGTATAATAAAAAGTGAATTATTAAGATTTGGATCTTCAACTTTGAAAAAAATACGAGTATATATGTCGATTACTGTGTTTAGTACAAAATATTCATGTGGGTAATTGAGTAAAGAGGTAATATTTTATAAGAATTTATTCATATTGAGGTACTCTTGGGTTCTCTTATTTATTATTGTTATTTTATTTAAATATTAATTTATTTTTTTTAAGAAAAAGGGATGATAAGAAATAAAAAAATGATAGTAATTGATAGAGGAATATAAAGCAAATCTTTGGAAGGATTTCCCAAAGATGTGGATCGCGAGGATATGGTTGGGGTTTTGCCAACCTTCCCAAATTAAAATTATTTTATTAAAAAGAAGAGAAAAAAAATCTAGTTAAAAATTGACGAAATTCGAGGATTAATTCAAAAATCAAGACTAACCAAGCATTGATTTAAATGTTGAAATTTTAACTTCTTTTTTAGAAAAAAAAAAAGTTTATTTTCTCACAAAAAAAAATTGTTTGTGATTTCCATACTTAAATAGACATTATAGTTTTTAGCCATTTTAAAAAATAAAGATTTTTTTTCTTGAAAATTACATTTTCTTTTGGTTTTAACAAATTAAAAATTGAAAGATGTAAACAATTTTAAAAATAGCAAAAAAAAAAAAATGATGTGGGTGAATGAATAAATTTAAAGAAAAGAAAAGAGAAAGGGTGAATGAATAAATTTATAAAAGAGAAAAAAAGAGAAGGAGAAGGTAATGGGAAAATGATGGGCCACGTCAACTAAATTCCAGCGGCAATTGTAAGACAGACATGTAATCGTGGGAATCCACTTCACACGTCCTTACTCATTTTCCCTTCTCTCCATTTCTTCCTTTTTATTTTCTTTTCCTTTGATAACTGACGTGGCATTCCCTGGTATTGACATGTCAGCATCCGGAGAAAGCTTATTTCTTTTTCTTTTAGTTTGTTTGCATATTTATCTCATGGAGTTTGCTTATCAGTTATCAGCTCTATATATATATATATATTTGCGGCTATAATCATGTGTGTACACTATAGACCGTAGTTTCAATGCATAAACTCCCACCATCTTAAAATAGTCAAAGTCAACTTCAAATCATACGGCTCTCACCTACCCACATAACCACGCACGCTCCCCACCCCCTCTAATTCTCCACTTAATTCATCATTAGTTACGGGGTTTAGTTACTCTTTTTCAAACCATTATATTTATCATCAACTAAAATCACCTTTGCTCTTCCACAATTATTCAATTTAGTGATTGAAGAGGGATGTAGGTTAATTAAGGAAAAAAACAAATTGATATGTAGTCAAATTGAGTTAAAAATAATATGGTTTGGTTGCTATATTTAAAAAATATTTATGATTGTATTAAGGGGGAGTAGATGGAGAGGAGAGGAGAGGAGAGGGCTTAGGAGGGGAAACGAAGATGGATAAGGATAAAACTAAAAAGAAAGTTAGAGAAACAAAATTTGAAATGTATACAAAATCTCAAAACTTGGTGCACATCCACCCAATAAAATTCCACCTCTCTTTCCTTTTTAACCGTTTCTTCAAATTATCCACATTTCTAATATCATTTTCTTTCCCCCATAATCAATGATCCACGCGCCTATATAAATACTCTCTCACCACCCAATTTCTCAAACCCCATTCAACGTTTCTTCAACCTTTTCCCTCTGTTTTCTCTCCTTTAATTTTCCTCTTTCTTTCTCTTCTTTTTCCCACTCTAAAAT

>Csa1M574260.1

CCCTCTTCTTCAATCTCTCTTTCCTTCACTTCCCTTCACTTCCTCAGCCACTTTCAATCACTCCACAGCTCCACCAACTTCAAACTCCTCTCTTTAAAACCACCGATTCTTCCTCTTTGATCCTCAAAACTCAATTCTAAACTCTCTCTGTCCCGTCTCCTGCTCGATTGTTCCTTCTCTCTCTCTCTCTTTCTCTATCTGCTGTTTGCTTTTCTTGGTCACGACGGTCAAGAGAGAATGGTTGGAACTTGGGAGGGAAAATGAATTACCGCGTGAAGAAAAAATTGCGCAGCTACGTTGTGTCTTTTTGCGCATACTCGTCTTTATATCCGTCAATATGGGCGTAGTGAGTTTCTTTATACGTGTTGGTTTGTGTGTCTACGGATGAAGTGGCCGAAATTCAGTAAATGGTAACAATTTTTCACATTCCACTAATCAACAGATCTACGCTGACTGTTCCCCGCACTGACCGTTACTGCCCAATCACAACCCCCCAACTCAGCACTAAATTCAATATAATCTGCCTGTTTGAGCAAAACCATCCATCTTCTTTTCCCAACAGAAAACATTATAAAATATAATTCTCTTTTTTTTTTGTAAGTAAATGGAAAATTTGCAAAAATAGCAAATATTTTTGTAAAATTTTTAAAAATAGCAAATTTTACAAAAAATACTTACAACTTATAGTAAATTTTTTACTAATAGTCGTTGATATGTTATAATGATAGAATACTATCATTTATATAATCTAAAACTATGATATAACTTATAAATATTTTAATTTATTTTTCTATATTTAAAAATGTCTCAACTTTTTAATGATAATAGAGTTAATGTCACTTACATTTCCAAAATTAAAAAAACAACAAATTTATAAGTTGATTGTTGTCTTATTATATCATATTTGTCAAATTTGCAATATAAACAAATATGTGTTATGAGCTTTTTTAAAAAAAAATTGTCCAATTTTCCAAAGTAAATTGTCAAAAATAACAAATTTTACCAAATATTTACAACATAAAGCAACATTTTCAAATTCTATCAATAATAGATATTGATAGACACTGATGTGTTTCTATAAGTAGTATGGATAGACTGTGATAAAAGTTTATCAGTTTCTATAATTGATATAATCTAAAATTTTGTTATAGCACGTAAATATTTCAATTTATTTTACTATTTTTAATAATGTTCATAGATGAAAATTGTATGTACTTTTCTTTAAACTTATATGTGGATCGTGGATGATGTATAATTAAATTCATATTTTATTTGTTAGTTTACAGTTTTTGGTCTATATACGGTAATTTAAGGTGGTATATCTATTTAGTTTTTGTGGGTTTTGATAGTGTAAAATTATATCTTAAAATCAATTGACAATGAAATCAGTAGTTTGTATATCTTATTATAATTGTGATTCGTCTTTTTTATTTTCTCATTTGATCTCAAATTTAACTTTCTATCTTAGATTGATAATATACCGATAAATAAAAAAACATGTTGGATTAAGTAGATTGTTGGTTTATTAGTAGCACAAGTGACACTACTACTGTATTGTGTTTGGGTAACGTGTTGAGTTGTGAACTTTTGGACCATGGTGTAGGAAGTTGGTAAGGTATAAAAATGGTTGTGTTTTTAGATATAGAACTCGCGAACTTTTTGAGTCAAACAACTAGTAGAGTTAATAGCTCCGCGCATGTTAACTCTTCCTTTTTGACTCGTTGAATCAAATATATCTTGTATATGTCGAGCTAAAGTTGGTCAATTATGTCTTTTTTGGTTGCTAGTTGGAATATAGGTGAGATGTGTATTGTTTAAGTACCACTCTATGTGTGATTTATTGATTGTGAACGTGTTTATTTTGACCGTAGTTTATAGCTTATGTGGGATTATGCATGTGTATGTTACACAAAATTATGATATTCACGTGTATCCAAATCTTCTTATTGTCGTTTCATCTGCATAACATACGTTTATAGTTGTGAGTTACATGAATTATG

>Csa1M695390.1

GTGAGAAAAAAATAAACACAATAACTTTGATAATCCAGTTCAGTAAGTATCGCCTACGTAAGCTTGGATGAAGAGATTAATTTATTAAGGTTCAAATGAGTACAGGAATGTAAAAATGCTAAGATCAATATGCATGGTATATAACAAGATACTAAATGTCAATTATCCAGATCTTCATATGATGCTCCCCTAAATTGGTACTCTGTAATAGTTACTATTATTAGATATCAGGTTTTCGTGACATTTATATTTGAATCTTCTGAAGATATTCGGCTTCCCTTGATTTTGATAATTGAATCTTCCAACGGTTAAAATCTTCTTTAATCACTTGTGCCACTTTTCATAGATCGGTCTTATTGAAGTATTTGATTCTTCATACAATTATTTGTTAGTTGAAGGTAAAGTCTTACCTTTCAGTTGAGATTCTGCTACATATTGTTCTCTTGTGTGTCTTCTTCCATTTACTTCTTATGAGTGAATCACCTAAGACAAAATTTGTTGTACTTGAAAAGAGAATAGAGAAACAACTTCATAGTTTTTTTGGTTAAAGATAATAAGAAAGAGATTCAAAGTTTCTGTCTTTAAAAATTTTGTCAACAATCTTGTGGAAGCTTTTCGACAAAGAAATTAAAATATCTTCTTGAAATATCTTATTGTAAATAAAAATTATTATCAAAAGATAATCTTGAAAAATAATTCTTTCTAAAACAAACAGTCCCAAAGAGAAATAAAAACTAAATAAATTTATTGTAGAAAGAAATAACACATAAAAAGAAAAGTAATAAGTAAATATATGGTAATATAGTATGTGTGTAGATATATATTTTGATCAAATATGGTAATATCATAAAAAATGAAAATTTCACTTTTTGGTTGAATTCCTCTTGACATATAATATAATCCATGGAAAAGAAAGGAGATAGTTGAAGAACATATTTTTAGAAGCATGATTATATTTTTGAATGATGATGATACAATTATTCAAAAAGAGAAATAATGATTGCGATTGATTTGCCTTTCTTTTTATTCCATTTTGACAAATAAGCTATGTGAATAAAATAAAATAAAAAGAGAACATGGATTGGTGAAAAAACAACTCTCAAACTTAATTTTATAATCAAATGCTTCAGATTTGTACAATAAACTAATAAGAAATTGAGTTCACGATCACTTGATTCATTTGATTCTGCTTTTTGCATACCCTCCCCTCTCAAACCCAACCTTTTTCTCGACCTATTCATTTTTTGTCAACATCTCCACATTTCTACATTCATTGACATTTTCATTTTATGATATATAAATCTTTGTGCAAAATAAACATTGAAGCATTATCTTATTGAAGAGATATATTCAGAGTTTCTAATGTTTATATCAGAGTTATTTTTATTTTTATTTTTTTAGCAACATATTTATATTCAAACTTAAACTGTATTTGTTTCTAAATAAAAGAAAATTCTTAATTTATTTTTAAATAAAAGAAAATTCTCAAGTTGATATGTATAAAACTTTATTAATTTTAAAAAATACAAATTGGCAAACAAAAAAAATATAAGAACTAAATATTTTAGAAACAAAAAATTTCCAAATATATGGAAAATAATATAAAATCACAAACATGCATAATTTTTGTTTAAATGGAAATTGATTATTAATATAGTTAGTATGTGAATTGGGGTATGGTGTGGAGACATGTAAATATCCAAAGTTACATAAATGTGTTAAAATGAAAACTTGTTTAAAAGTAGAGATAAGGGTATTAAAATGAAAAATGTGGAGGAATTACGGGTTTGTATTTACTTACAGTTAGAAATGAAATGAAAAGTTATGAAAACTGAAAAGGGGCCGTCACTTTCATTGCACAACTTTTTCTCTTTTCTCTCTTCTCTCTTAACTTTTTCTTCCCCAAAAATATGTGCTTTCCAAATACTTCTCTCCCCAAAATCTGAGGAGGAACCAAGGATTTCCTCTGCTCACCAGCAACCCAATTCCCTTCATATCAA

>Csa2M012110.1

TAAAAATACTTAAAATCAAGTAAAGATAAGAAACAAAACCAACCATGGAACTTTTTCAAAAACAAAATTTTAAAAAATAATCATATAACAAATTTCCTAAAAAAAATACTCGAGCCTTATTTTCAAAAACAAAATTTAAAAGAAAAAAAAAAAAAAAAAGAAGCCAAAACTCAACGTTTGATTTTTTTTTATAACAAAAATGTGACGAGGAAAAATTGAATTTTTGACTTAAAGGTCAGAGCCAATAGGAGAAGTTCTATACTGAAATGGCATGAACTCAAATCATCAATACTTCAAAAAATAGTTCGGTTTTGGTCCTCAAAATTGTATCCACTTTCTAATTTAGTGTTTTTAACTATTAGAGGGAGTTTGTAGCATCAAATCAGTTATTAATGGTACGTGATTATTATAATCCACAAATTATAATAGTTTTGTGTTTAAGATGCAAACTATTTTAGTCTAGATTATAATAGTTTGTATTTTAAGTGCATACTATAACGTGACAAAAAGAGAAAAAGAAGAGTATGAAATAGTAACATTTGTAATTCATAGTAAAAAAAACATTAAATATTATCGTTTAGGTTACTTAAATAACTCATTTTTCTTGTTTAATCTTTGAATTAATGAATACATTAAATCACCTAAGAAATGAAAAACTTATTATCGATCAATTTCAATATTAACGGACAGTCATAAAGTACATATATTTAACATTTGTATTACTAATGTCGAGGTTGAAAATTAAATCAACCCGTTTTAATCTAGAGGGGAAAAAAGGTTGGCCATTTTTTCAAGGGATCTTTTCACATTAGGACCCACAAGTGATTTGTTTCTGCAAAGAGAAGACCATTGTGACAAAGAAAATAAGGATTTGTCTCTCTTTTTTTCTTGTTTATATTTCTTGGAATTCTCATTGGCAATCTTTATTTTACAATTCTATGTCTTTGAAAAAGTCATCACATTGAAAAAAGAAAAAAAGTATGAAAAATGCCAATAATAAAATAAAATTTTCTTTTAGGGGATGTTTTGGCATAATCTTTATAATCTTTCCTTGTTGGAGACTGCTATCAAATAATATGTATAAAACAAAATTGAAACTTTCTTCTTACAAAAAGATAGTTGGAATTGAATGTGGTTATGGTGGGAATCTTTTTCTTTTTTTTTTTTTTTTAAAAAGGAAAACAATAAGTGTTTTTATTTATCAAACTTAAATAGATAAGAAGTTTTATAAAAATTGATATTAGAGATTTAAGCCATGTATTGGTTATTATATTTAAGATTGAGATTATTTTAGTTTGGGTAACAATATGGTGTTTAAGATACAAACTAGTTAGTTTATAATAACTAGCAGGGGACCTAAAACTAATTAGATTATAGTAACTTTCTCTACGTGGGATAAATTAGGAATCCATGTGCTCGAAACTTTAGTGATAATGACAATTTATTCGTTAAACTTTAGTTTATAATAATTTAGTTTGTAAATTTGAAACCATTTATTCCAAATTCTAACCCCTCACTCTCAATTTTGCCTGCCATGAAAAATGCTTTAAATTGTAGTTTATTAATTCATTTAGTTTATTTTTTTATTTTTCTATATGTTTTTCTTAATTTATTTTCTCTTTCTCTCTCTCTATAATTGTCACATTCTGGGAAGTGGTGGTTGGCTGGCTCTTTCTTCCTACTTTCAAAGGGTACCACTGTTCTTGCCTGTGGATTAACGTGCTTTCTCTCTCTCTCTCTCTCGATTGAACAATTATTATAATAATTCGTTTTTATTTCTATTTCCCCTTTGCTTTCTATCTTCATCTTCTTCACTCAGTCACTCTAAAAGGGTTATGGAAAAAATAAATATATTGTTTCATTTTATAAAATCACCCTTTTCTCTCCTTCCCTTCCTTGTGTAGTGTGTTTTTTAGGAACTTAACAACAACCCTTTTCAAGTTCAATCATATTAAAGAAGAATCAAAGGCTTTTGGGGTTGAAATTTTTGAGAAAAGAAA

>Csa2M021750.1

AAATCAAGTTTCTAAATTCTTTTCTCCTTATTTATTTATTTGTTCAAAAATAATTAAAAGTAGATATTATAAGTTAAGAAAACTAAGCTTACTTTATACAAAAAAAAAAAAAAGAAGTTATATTTATGATAGTCACTTGACTCTATTCACCTATTTTTTATTTTTATTTTTATTTACTTTATTATCTATTTTTTTAATCCACTTTAAAAGTTCTAAAAAGATTTAGAAAGTTTCTAATTTTTTTTAATCTTTTGAATTTAGCTAAGTATTGAATTAATATATTAGTAAAAATAATGAAAGAGGCTTGAATTCTATATAAAAAAAAGTAAAAACAAACCAATAATTTTCAAAAAACCATAATCAAAACCTAAAATGACGGGAAACACATGATCATACAGAAAAATCTAATCATTATCTGAAAATCGAATAAGTTATCAAGGACGAGGCAAAGTTTAAAACACTACACAAAAGGTTTGAATTGGATTTATTCGTGATAGGCTTTGAATAATTAATCATACTGTTTGGTATAAATATATATGTTTATTTATTTTTGTGTAAGGTACAGATCCATATGCAATACATTATCTGGGGTTAAAGCCATGGATGTGTTACAAAGATTATGATTGCAACTGGGACATGGAAGATCACCAAATATTTGCAAGTGATTCAGCTCATGCAAAATGGTGGCAAGTTTATGAATCCATGCCCACTGAATTACAACATTTTTGTGGTCTCACAAAGAAGATGGATTCAAGAATAAGAAAATGGAGAAGTATTGCAAGAAATAACTCCACTTTTACCGATGCTCATTGGAAGATCAATATCACTGACCCTAGACGACTCCGTTTTATGGATGATCAGGCTCCTCTTCATCAATAAAATTTTGCCTTCTTCTTCTTCTTCTTCTAGTAATAATTCTCTCTCTCTTTGTTTCTTTTCTTACACATATAAAAAACATACACACAGGGTTTGAACTTTAGATGATAAAAAAATAACTTGTTGATGAAATGAGAATAGTGTACAGTGGTATGGAGAAAATTTGGGAACTGATGTAATTGTAGACTTAACTAACCTTTTTCTTTTCTATTTTATTTTCATGGAAATTATGTTTTCTAACCATTTTCGAATACGCATGGAATATGACAGTCTACAAAATATAATAAAATTCGCCTAAAATATTAGTAAATATTGATGTAAAAGTTACATTATAGATATAAGAGTTTCCATATTCAAAAGTTTAGGTCAATTAATGATGAAGTGATATCAAAAAATTATAAACACTATATATCAATTACAATAACTTAACTCCCCCACTCATCCGGAGGGTGCTTAGAGCAGAAACTTCACTGTTATAATTATATATAATTTATTGTTAAGTTATATATAATAATTTATATTTAAAATATAATAATTTGTATTTGAATATAGTAAAATAGAATATGTTTAAGTATAAACTATTTTAATTGTTGAAGAAAAATAATAAACATGGAAGCAAATAAGAATTTTGAAATCGTGTTAGATTATTATTATATAGTTTATAGAACCATTACTTCAAACATGCTGGAAGAGTAAGGAACTCGATGTTATAATAACAAACTCAACACACCAACATTAAATAGTAAAAGTCAATTATTATGATCGTCCTAGTTTGACCTATTTATTAGACCATAAAATCTGGAGAAAATGAATAATAATAATTATCATTTCATCAGTTCTCCCCCCACCCCACCAGAGAGTGATTCCTTCAAAATTAAAAAAAAAAAGGCACTGTCGGCTTCTTTCTCTATTTCTTATTTTCATATATATTCTTTTTAAATAATTAATTTTCTTTTCTTTATTTTTGAATATTACTCTCCACTTTCTTTTCCCTCCTCTCTCTCTCTCTCTCTCTCCCCCTTTCGATTCTAATTTTTTCTTGCCGACCGCCATTAAATCAAAACTCTCCATTTCCTCTTACAATTCACTCTTCTTCTTCTTCTTCTTCTTCACTGATTCCTCAA

>Csa2M049880.1

CTCGTATGCAACACCCTCTCTTTTTTCTTCTTTCTTTTCCATTTCCTTAGCTGTCTCCATTTCTCCTTACTCGCAACTCACTTTGAAATTAGAATTTATCATTCTAATTTCTACCGTAAGATTTCAACATCCTTAAAGATCGATATATTATCCATGTCCTTCAGTTCTTAGTTACTTCATTTTATTCATTCAACTTCAACTTCATTTCATCGGATACTCTCCTTGTCGGTTACACTGTAATGATGGTCTGCAAATGACGCCTAGGTACTTTCAAATAAAGGTTAACTTGATTATTGCAACAGTGGTATGCAATTGTACTATATAACACAAGAGTATGAGATATGATTTCAAGTTTATATCATCTCATCCTGTGATTACACTTAAACCATGACATTCTTTTTTAAAAAATCTCTAATATTTTTTTGTTACAAGGGTAAATTAACTAAAAGTTGTGGGCTAAAGGTCAAGGTTGTTGAAAGTTGCAAAAAAAGGAAAAGAAAAATTATGATGACTCATCTTGCGGTTTGCATCGGTAAGAGTGCCTCGAGTCGTGAACTTAATATGTGTATCATTTTAGGAACTTGATGAAATGGTGAGCTTGTAACACAGTTTCACAAGTTGAAGTTCATTCATTCTCTCATATGTAAAATAGATGAGTGGTTCTTTTCAGTGTTAACTCAAAAACTTGAATATGAGGTATCACCTTCTCACTCGCCAAGTGACTTGAATTTATGATTGACTATAAACAAATTGTTCATTCGAGGAATTAGTGATACTCCAAGGGATAAAACCTAAAGATATTTATAGGAGTAAAACACTCTTTTGGACCCAATTATATCTATGAACAACTCGTAAATGATCAACTTGCTTATAATAGTTATATTTAAAGGAATTGAGTTTCGTGCAAAAGTATGTGCTTCCTTTGCTTCAATTAAGTTCAAAAGTTAAAACACATCATTAAAACAAAACATATCAGATTAAACATACTTTTTACATGTTACAAGGGATGAGTAAGTTACCTTTGAAAACTTTTCTTCAACTCTTCTCCCGGTTTTGAAAAGACATCGCACACTCTCTTTTGAATCGTGAATTAACACTCCAATATCTCCATAATGGTATGTGATATTGTCTACATTGGGCATCACTTTTGGTTTCACCCGAAAGACATCATACCATTGGAAATAGTTGTTGTCCTCCTCTCTTATATTTTCATCCATTTATTTATTTAATGTCGAACTTTGATCAATCCTCTTGTCGAACAAAGTATAATCAATCGATCCTTCCAACAATCATCAAATCTTCTTGCTAACTCAACTTTATCCATATACAATAGACCTTTTAAGTTAATACTTGAACATGATCCACTTGTAGATTTTCAACCACATATTGTTCAAGATTGCACATAAAATCTTGATTTTTTTTTTTTCAGTTAATTGATAAAAAAATACATGTAATGTTTTTTAACTTGTCACTCGATCTCCTATTTGGATTGTATATGAAAAAAAGATGTTATTTGAATAATCATTTTTAGTTTTTCAAAAGTTTGTTTCATTTGATAGATACTCAATTCATTTTCTATTTAATCACAAATTATATAAAAAAGTACTATTAAATACCCCTAATATAATAATTTTAATTTTATATTTAAAATACGTTCATCGATTGAGTTTTTGTATTTTATATTTTGTTTTTCTAGTATATATCGATCGACTTTAGCATTAGAGATAAGTGTTGGAGAGATGATTGATAGAATTGGTTTTCATGGATGATTATCAGGGTTAGCAGTCGAAAGTAACTACGTGGAGACCGTCATTGTACAAAATGGTTGTCATAGAGCTTTTCTTAAAGTGGTTCTCGAAGTTCGTGCAGACATATGGTATTTGTTGAAGTTGGTCTCCAAAACATTGTAGGATTGTGTCCTTACAGTTTGCGAGTTACAAATAAAGACATGGAAAACATTGAAGAGAAGGACAAATGGATTAAGTTGAGGGAAGTCAAAC

>Csa2M070870.1

TTTTCTACTTTACGAGAACCCTTTTGATTCCCTTTATATCTTTAGCATCACCGATGCTAGGTTCACAGCCGGCAAGTAGAATCTGTAACCAAACAACCAAATGGAAACACAAAGATCATTTCTTTAGTAAAAACAAGTATTTATATTACAGTCTTATCAAACAAAATAACAACTAACAAGTAGCGTACTAAAAAAAAAAACGAAAAAAACAAACAAGTCTACACAAACCTCCATGAAACTTATCAAGCAGGAGAAAGAAAACCCACAAAACAAAAACAAATAGCATAAACCCCAACTTCAAAAAAAGAAAACATATATGAAGATTTCCAACCATTTTCAAAACCAAAAGATTCACAACACTAACTAAAAAATCCAAACCAATTAAGTCAAAACCCCCACAGAAACCATCCCCAAACAGTAACAAAAAAGAAAAAGAATAGTATTTCGAAGACTTCAGTAACGACCCAGAAGAAGAAGAAGTTGTAAACACCAAAATTAACATGAAATTACCAGAACCCAAAACTCAACTCATGTATCGAACATCAAAACAAACTACCACAAGAAAAAGGAGAAAAAAAAAAAAAAAAAACATATCTGATAATCGAAGATGGAAGAATACCCTCAATTTATGAAGCTAAAGTCAAAAAGATACCTCGATCAGGAGGTCAAAGCGTGGGGATTCCGATTTGTCAAGTCTTCATCACAAATTGAAGAAGCTAAAAACAGATCGAATCGAAAAAGAAGAGATGAAAAAGGAGAGAAATCGCTCAAGTTGAGTATGAAGACGAAGAAGAAGGAGGAAAGAAGAAAGTGAGAGGAGGAAATTGGCTATGAACTACAGAGAGAGAGAGAGAGAGAGAGAGAGAGTTTCACAAGTCTTCTTGCTCCCGTTTTTCCATTTTATTTCTTCTTCTTTTTTTTTTTTTCTTCTTCGTTTTGATTTTAATTTTATAAATTTATTACAAAGACTGGAGAGGAACTATTCCCTGTGTACACTCATACCTTACTACCCCCGCCGGTCGTGGTACTGTCGTTGAAATTATCTCGCCGACATCCAATTATAAATCGCCACGGCGAGTCTTTATAGGGTTGTATCCTACTCTCACTAGACGCGTTTGTAGTTGAATGTTTCTTGTTTGTGCCTTTATTGCCTTGTTCTTAACCTTACCTTTCCTAAATATTAATCTTATACACAAAATTTCTTATGCTTTTTAACTTTTTCAAAAAATTAAAAATAAATGGTGATAATTAGATTAATTCATCTTTTCACCTCAAAAATGTTAATTTAAATTCCATTAATTATTGTAAAAAAAATTATTCTCGTTACAAAAATTAATCTAACATCACTTAAGTCTTCCGATCATAATTCTAAATAAAGATATTTGATAAATTTAATGGATAAGATAAAATGGATGAGATCTTTTTTTTTTGTCGTATATGTGTCGTTTATCAAAAAGATCATTAACGTGGGTTCATCAATTATCCGTACGTCTACGATCGTTCATAATGACGATTTATGTTTTATCTTAGATGTACGTTAAAAGTGAATCTAAGCTAGATATATAGTCTAAAATAAGTAACAAAGTCATTTGAGTTTTAATTTTCTGACAGGTAAATTGGACAAGTGTTACATCGGTTTGTTGCATTGAACGTGCGGATCGAGCTTTATCACTAACCTTTAATAAGTATATCATTTTCTACTAAATTAAAAACATCATTTTTTCAATTTTTACCTTCAATCTATGAGTATAATAACTACATCTTGTTATACTATAAGTCTCCAATTAGGTACAGTAAACATTATTTCAAAATTCTCAATTTGTTACCGTTTTTACTACTTGACCTTCATATTATGTACTATTTGCTACAGTGTTTACTATTTTTTTTTTATCAAACTAAAATAATGTGCACACCAACTATATACTATTATAACCCAAACTAAATTAATCTACACATCAAACACAAACTATCTTATTCAAACTATAATAACTCATTCATTA

>Csa2M278170.1

TGATGATGAAACTAAGGATGAATCTAAATGATGTTGAATTGAAGAAGGGATTTGAGATTTTGATTTTCTTGGGGAAGAAGGTGAAATTTAGACTTTGAGATGGGAATTTAGAGTTTAAAGATTAAAGATCGTGAAAAAATAATAATTCCATAAAGGAAACGGGAGTGTTAAGACGAAGATCTGTTGATTTTATATGGAAAGAAATAAGAATTTGAACAGAAGTATTTATGGCACATGATCTTCAACCGAGATTTTTGGCTTCTTTATTTATTTTATTTTATTTTTCATTATTTACATTGAATTCGCGATTTTAAGGATAGTTCTCATCATTCAATTTACCAATTTTATTTATTATATTTCTTCTACTTAAATCTTTTCATACTTTTAATATTCTCTCTCTCCTGAAAATGTATTTTGAATTTCTTTTATTTTTATATTTGTTTTAAATTTTAATTTTGATATTTCCAAACCTATTTTATCTCCAAAATTGGATTAATCTTTCCGTTTATTTTTCTATTGAAATCGGATATAATATCTACACTTTCGTGATAGTGTGATTTGGGATATTTTAGTATATCCTATTTTTTTCAAATTTGATCAAACCTCAAATTTATAGTTATTATTTGAACATACAAATAAAAATTTATGTCAACATAAACTTAACTCAACTCCCACTTATATGTATCTTACGACAAAATATAGTAAAATTGTCTATAGAGCATGACATGAACACAATCACATATACAAAGTTGAAAGATACAAAATTTAAAAAAAAATCCTTCCACAGTTAAAACTATATTTATAGTGATAATTATCTCTTTAGTTAATTTTTTTATTATCTAATTGTTTTCAAACTTTTTTTTATTAATATTATTTTGATATTTTTTCTTACTTACAAAAATTGTTGGAGAACTTTTGATTATCCTATTAGTTTTATTTATTTATTAACTTGTTTCAATTAATTGCACGAGTCTTCCTCGTATATTTGAATATCACACTATAAAACACACACGCGGAGTAAGAGAAAACAACGTGTAGATTGATTTTCTAAGTGCTTGAAAACATGTATGAGATGAGAATTCATTTGTTTTGAAGATGTACATATGCATCTATGCGTTTTGTTTTCATGTATTGTTATTGAGGTTTAGTCAATGTCGATCAGAATTTGAAATGTAAGTTCGACAATGCAACTACCTTTGGTGGTGGACTCACGTACAACATGGCAATGGTTTGATTGTTGGTAGATGAGGAGGAGGTGTTGTCCGTTGTAAAATACATGACATACGATATTTTTAGCTTTGAAAGCATGATCATGTGTGATCCAAATCATTCGACATATTTGACGTTCTTGTAACTTGTTGGTTGACGTAGAACGATTTTCCAATTTGAATTCAGTGCTCGGTTGAGATTGATTTTTCCTCAACTACATCTGTAGATGTATGAATTGGAAACTTTTTTTTTTTTTTTACATCAATAAAAACAAACTTTTTAAACACTATGTGCCCTTGAAAGTTAATGTTTGTGAAAATCAGTGCTTTTGGACATATGATATTGACGAGGCTATATGTATATTTGAATTTACAATAATACATATTATTTCCCTTCAAACAATTATAATTTACAAAACACACAAATTGTAGATTAATTGTAAAATAAAATTAATTTACAATACAAAAACAATGTGAAAATACAATAATTTCCCTTCAAAATTACATTTAAATAAAAAAATAGACAATAAAAATTTATTTTTCATTACAAATTTTCTATTGTTGTCACGTGAAGATCATATGAGTCTTTATTTATTCAAAGAAATAAATAAAATATGAAATTGATTAACTATAAGTCGATCATCAAATTAAGGTTATTAGATTAAATATTAAACTAATCTAAATCAATTTCTTTATATAAAAAAAAATCAAATCAATCATGGTAGGAAAACAAACCATTAAAATATAGCTTGAAAATCTATATCATAAATAAACTAATATTACTACATTTAT

>Csa2M360650.1

CTAGCTAAGCTGGTCGCGGTCTTCAGGCCGGAGCCGCCGCCGCCGCCCGCCGATTAAGGCCACATGTGCACAATCTCACCGTTTTTTTCCCCCCTTTTCCTTTTCCCGGTAAAAATTAAAAAAAAAAAAAAAAAAGGAAAAACAAATATTTGCTTTTTCTATAAACTGCCTTTAACCAGATTTGGCGGTGGTTCCGTTTTATCCCCCTCCGGGCTATCTTAGGAAACGCCCACTTTTATTTCCATTTTCATCTCCTTCACTTTTTTTTTTTAGGGTTTGAATAATCAATAATGAGAAATTCATACGACGACTGATTTGCTCGGCTCCCAAAAGACGACATATTGACTTTAAAAAAAGAAAAACGAAAAAAGAAATTAAAATTTGGAAGTTATTAAATTAATGGATATAGGTCAAAGTTGGTAAGTCCAAAGGCAACGTGTTCGTACGGAGGGATTGTAAAGTCATTAGAGGTGATTCTATTGTTTTGTCAGCGATGGTGCGTGGGTTACATTTTCCCTTCCAATATGGGCAAGTGGCCTCCACGCTTCCGACATGGAAACGGAGAGCCAGGGCCCAGGGGACACACCTTACAAATTTTTTATATTAATGACTCAGTCGTAGATACCGAGTCAAACACTTTACCTTTTCTTTTCTTTTTTTTTTCTTTTTTCCTTTTTGAGGTATTGTTTTATAATAATATTATTGAATTCCTACGGTGACAAAAAAGTCTTTTTATTTCTTCAATTTTACTTAGTCGTATTGGACACTGTCTCCCTCTCCTTTCCAAACAAATATTTTTGTATATTTGGAATTCAAATTTATTCAACAATACAGTATAATTATACCTTATCCTTCTGGAAGAACATGTGACAAATATTTTAACAAACTCACTATTTCAAATTAAGTTTTCTCTTTTATAATTTTAATAAAATAAATAAATAATAGTCATCGTTAACAAAAACAAACCCTTTATATATATGTAAACGTATATGTATATATGGATCAATTTTTTTATTTTTACTAAAACATTATAAAATTAATTAGAAAGACATAATTTACACAATCTAACAATAAAATGAAAACTAAATGGTTTCATTAAGGGATAATTTAAAATTTAAAATTTTCTATATGATAGAGATATATCATATGAAAACTAATATTTCAAAAAAATTATTGGTATAATGTTAAAAGTTCACACTTAAATATAAAAAAACCTCAAAGGTTAATGACAGTAAAAAAAATTCTAACAGTTAATCATTGCTACATTTAATATAGCAATGAATTACCATTGAATCATTCAATTACAAGTTGAGTTAATGATTTTTTATGGATCTATTTAGGTTTAGTCAGTTAAGTTTTTAAGAATGTGTTTTGAATATAGAAGTAATACTATATTAACTATTTCTTAACACATTAAAAATTAATTTATTAATTATCTTTTATAAAAGGTATAAATTCAAAATTGGAACTATCCATAGGTGTAGATTATTTTAATTTTAATTATCATATTATATAATTTGTGCAAAAAAAATGATTATAAAATAGTAAAAACGATGGCTACTTGAGAATTGTTAAATAGTTCTTACTGTGGCGAGGATTGTTATGACCTTACGTTCATTTTTAGTCTTAAACCCTAATTTTGCTTTTCTTTCTTTCTTTATTTTTGAAAGAAAAATATAAACCCTAATATTCGCTTGACAGCAGCGAAGCCACTGGGAGCAGCAAACGGACACGTCAGGAAACTGCCGCCAAGTACTTCTTCGAAATTGTCTTAAAACTCTGCCGAGAAAGTTAGACCGGATTGATCACCTTCCCCGGACCTCCACATCGCCGTTCAGTTACTATGAAGGCCGTTCGCCTTCTTTTTTCCCTTAGACCAGTAACTACCTTGCCTTCGCCATCGGTAGCGTTATTCTCTTCGTCTTCATCATTAATCACTAAGTCGATGAACTCCGTCGCCACTGAGGCAAAGAAGCAAGGTTCTGTATACGAACATCAGC

>Csa2M416770.1

TCTTTTATTTTTTTCAAACTTTATTTGGTTAATGATCGTGTACAAAATATTAAAAAAAATCTATTTTAATTATTATTTGGTTGTTTAAGATCATGTGTCAAATCTAATCGATAATGTTTATATTAATGATTGTGTACCAAATCTAAATGATTGTATGCAAAATCTAAACGATCATGTACCAAAAAATCTTGAAAAAAATTGTTGAGATTTGTCTACCCAAATTTAATTAAATGATCAAATCTAAACGAACATGTATAAATTTTTTTTGAAATAATTCTTTTAGATTTGACTACCCAAGTTTAAACGATCAAATCTAAACAATTATATACCAAAAAAATCTTTAAAAAAAATAGTTTAGATTTGTCTATCGATTAAATCTAAATGATAGAAAAGAATAGCTATATCTAAACGATTGTTTACGAAATATATTAGGTCCGTTGGATGCCAATTAATCACAACCATCTTTTTTGTATTTTTCATTGTCGACATGTGAGTTTTTTTTCATTTTCAAAATTATTCAACACAGTGTAAATATTTACCACCTTGTTATACTTTTTTTTTAAAAAAAATCATTTTTTAAAACTATAAATATAAATATAATGTAATTTTCTTTCAACTAAAATTAAAAGTATATCAACTAAAATTAAACAACCGTTTAAAAGCTACAATAATAATAAAAAAAATGTATATTTTAACTTTTTTTTGTTTTTAAATATGAGAAAATTAACCAAAATATAATATAGCAAAATTTGATAATGCATAAATGATAGAAACCAACGTTAGTGGCAAAATAGTTTTCCATTTCTTTTCTACCTTTTTCACCCCTTCTAACTGATATGATGTGTTTATTTGTTCCATAATTTAAATTGGGTTCTTATGGTTACTTTGGTAACAATTCTAAATGCAAGCAAAGATTTTGGTGCTTTTGGTTTCGAGTTTTTTTTGGCCCTTTGAGTGCCTCATCCAATTTATATCATGATTGCTTCACTTAGAGGATACAGATCCTCCAATTAAGTCCCTTTTACAGCCAACATAAAATAAATAAATAGATAATATGGTTTCTAGAAAGGTTTGATGAGTATTAATTATTATTTTAACAACAATATGTTTACTTATTTCAATTTCATTATCATCTATACTTCATTTGATTACTAAAGATTATATAAAAAGACCAAAAAGTTACCAAACTTTGTTGCTTGTTGACTTTGACGGATGACCCAATTCTTAAAATTTGACAAATGTTATTTCTTTTTTGTTGCATATGTGAGCTTCTCTCTATGTCTTACTTTATACAAAGTCACTTTTTAAAAAGATACCAAAACAATTTGCATTCATACATTTCATACAATTGCATTGCAATTATCACCCACACTGCTTATTAGTATTTGTACGAGTATTTATAAGATATATTGGTGAATATTTAAGATGGATAACAAATCATTGGAGTTACGAATGTATCCACGAGGGGTATATGGAGATATAAAAAAATTGAATTTAGGTCGGTTGAGGAAAAGGAGCAAGTAGGAAATAATAAAAGAATTAAAAGTGTGTCCAGCTCAAAACTAGCAAGTAGCTATCTACATCACTCCCACCCAACACAAAAAACACATTTAAAAGAAAAAAAAGATTTACATAAAAAATGTAAACAACTTGGAAAAGGCATTGGCATATATCCAAAGGACCAAAAATGGAACACATGCCTCTTTCCCATTTCGCCATGCCATTCATTAAGTAGGCAGCTTCGAACCGAACTTCTCTAAAATCACCCATCTAAAATCACGACACCTGGCTATCTCTCCGTAGAGAAATTGTCAACCCTTCTCAAACACATAACAAACCCAACCAATAAAACCAACCCTTTCTTTTACTATTCCCAACTTTTTCCTAAATTCACTCTCCCTTTATATATATACACGCACACACACATACAAATTCCCCTATTCCATTTCAATCTTCAGCTCATACTCCCATCAAACTTTCTTTTCTTTTCTCCCAACCAA

>Csa3M002480.1

TGATAAGAATAGTTAAGAAGAAGAAGAAGAAGAAGAAGAAGAAGAAGAAGAAGGAATGGAATCAGATAAAAAGCTTAGGTGTGGAAAATCAAAAGGGGCAAAAGAGAAGGTAACGAGGAATGATTGAAGAATCCCACATGGGAATTGGAAATTAGGAATTGCGATCAACTAAAGAGAGAAGAAAACAACAACAACAACAACAAGATGTAGAAGAAGAAGAAGAAGAAGAAGGTTGGTGTGAAGTGGAAATGGGTGAGAGAGTGGAAGGAGAGAGAAAGTAAAAGGAGAAATTTTCAGGCACTTTTTGGAGTCTTTGGGTTTCAGTGAACGAAGAAGTGGAGAAATGGGAATTGTCGCGGAGAGCACACAAATAAAAAATAATAACTTTATTATTATTTGTGTATTTTTAAAATAAAAATTTGGTATATAAAATGTAAGTATTATTTTCTTATTTAAAAAAACATTAAAAATTATCCTTTTAAAAAAAAAAGAGAAAATTATTAAAGAAGATTACAAATGAAAAAAATACTAAACATAAAAATGGGCATAGGATGATTTTTGTTTGGTGAGGTTGGGGATATTCAATCAATCACTATGCTATAATTATTGTAAATAAACAAATATTTGAATATTTGAATATTTGAATATTTAAAAGAGAGAAAAAAAAAGATTATATTAATGATGGCATCATCATGTACAGTCGGCATCGAGTCATATGGGCCCCGCAGAAACTATATAAAAATAGCCTTATTGGATATTACCTTTTTTTTTCTTTTTCTTTTTCTTTTTTAATTTTAAAAAGAAAAAAAGAAAACTAATTAATTAATTATGAATGTGTTTTTAATCATTTTTAATGCTTTCTTCAAATATCTTCCCCCCCCCCCCTTCTCTTTATTTATTAAAAAACCCTAAGTTTTATAACTTGGAATCAATTAATACTCTCAATTTCATTTCATTTAACCTTACAATTCGAATCTTAATCTTCTTAATTTTCTTTCCACTACTTTAATAAGTCTAAATTGGATTATTCCATAATGGAGAAGAACCAAACATACCCCAATTGCTTGCCTTTTATACTTATGTGATATATATATATATATATGACTTTGTTTTAGTGGAACTATAAGCCACTCTTCCTATACTTACCTAAATAAACATCTATTTTATGTCGTAATAATTATAATATCTTTTTTAGGCAACATGTACTGTAAATTAAAACATCTAATTATGCAAGTAGTTTTTAAATTAATTCATCCAGAAATCAACGTTATCATTAAAATATAAGATTGTTAATTAAAAGGACAAGAGGAACAAGAGAATGTGTAACGCCCAAGTCCAGGATTTGGAATTCGGATCTCCGACATTTCTGAGTCCTTCAACATGCTTTGTCCTCACTCACATGCATTCTAGAAAGATTCTCAAGAGATCACCCAACATAGAATTACTCCAAGTTAAGCACACTTAACTTTGAAATTCCTATGATTGAGCCATTGAAAAGAAATGTGCACGTTGTTGGTATATGTATTGACTTTTTAAGCTTTTTTTTAACATTACTTTCATGTCCTTAGGATCCCTCTCATTTAGATGTGATATCAGTTCGTTCATGTCTTCTTCCTAATTTCGAACGTTATAGAATGACTATCTGTATATATGTGTGTATATATATGAAAATGTGACTAATACAAGCGGGCCTAAGATTACTAGATCATGCGTTTCTTGGATCATATTTTTAGTGCATGAACTTAATTGGTTTATATTGATATATATCCTCAACATAAAAGGCTAAAGATTTGAATCATTTGACAATTGAACCTCACACATTTGCTCAACTAAAACAAATGAATGTGTTGGTGACAATGTCCATCATGCTCAATCAATATCAACTCTTTAAAAAAGAAAAAGAGCTTTTTGACCAAAGGACAAGTGATAAAATAAGTCATATCTAATCAACTAAAATTATTGCAAGAATAATCAATTTGTTTGCAAATGGGTCAACTACT

>Csa3M062560.1

TCATTTTCTAAAACTTGTGATGTTAAAATATATTGATTTCAATATATAAATTGGTAAATCAAACACTTCACATTAATTATTTATTGGATAAAATTAAAATACAAATAATTTAAGGCGTGATGAATATAAATTTTGAATTATGTTTTATGTTTTTAGATTTACTGAGTTCAGCACATACTTTTGACAGACAATCTCGATTATGTTTTCAAAAATTATTATTGTATTCCATCTCCAAAATAGTGCAAATTTTGAAAACAATAATTTTATATTTACATTGTATTCAAAATTTGAATTTTACATTTTTGAGAAACAAAATTATATTAAATACATATAAAAACATTTAGAAATACACCCGTACATGTTATAATTTAAAACAAATTATATAATAATATAAAATCATAGTTATATGAGCTTTTAATTTAGGTTAAAATACAAATTTGGTCCCTATTGTTTTAAAAGATAAAATTTAGTATGTTTAGTTCTTTTGGTTTGATAAAACCTTAGAAAATAATCTCGCTCAAACTATTTATAAAGTTTGTCAAACCATAGGGAATATATACGATATTTTATCAAACTATACACTAAACTCTAACTTTTCCCAAACAATAGAGATTAAAATTGCATTTTTAAACAAAAAAAATTACATTCATAAATAAATTAATAATAGCCTATTGACAACTAAATATTAGTGAATAAGTAAGACTAGTTTTATAATTACTTTGGGTGATTTTACAGTACTAACTCAATATTGTATATGGTGGATTAAAATATTAAAATATTTCTGGAGTTTAAAATACACGTGAAAAACTTTATTTTAAAATAAAAAACATGAAAATATATTCCAATTTGGAAGATGTTGATATTTTTACATCTCCACGAGTTGAATATTGAGAGGAAGAGCAAAGGAATATACTAATCCTTTTCTTGAGGGGTAAGAAATTAGAAAATTCAAAATTGGCTAATGGAGGAAGCGCATTCTAGCACACCACATCACCAACTCCATTTCTCCACACAATGTTTCTAATAATATTAATATTAATCCATATGCTTCTATACTCATTCATTGACTTTGTATATATATTTTCTTGGAACCCAAATAACATTATTTGCCAGATCATCCACTTTGCTTTTTCTTCTTTTTTCTATTATTACAAAACAAAAATTATTCTTTTATTGAATTTTACATCAATAAATTTCAAACAATAATTATTATTTCCACAAACAATGAATAAATAATGCTATCATTTCCACTAAGGAAAAGCAAAAAGCTATCGTTTTGTTGGCAGTTTGTTTCGTAGGCCCCACCTCCCAATGAACATATACTTAAATTTAAATCTAAAAATTCATTATTATATAATATCACATAATTTAGTCCTAAATTTATTAGTTGTATTTTATTTTAAAATACATTCCCTAATCCATCCATCTTCTAAGCTTAAAGTATTGCTTCATTATTAATCTACATTTTTATTCTTTTCAATATCTACTAATATATATATATATACCACCTCCTAAGTTTAATGCAATTCAAGAAAGAAACGAAATACCATTTCCATCCACTTTTTTTTCTTTTTTCTTTTTAGTAAATCTTAAATCATATTTTTTTACTTATTTTTTAAATAGATAATTAATTTGAAATAATCCATAACCAAAATAATTAATTACTCTTTCCCTTGGAAAAAAAAGATTAATTTAGCATAAAGTCAGTTACATAAATCATACATTAAAAAAATATTATTTTAATTTAAAAACTAAAAAATTGAATGAAAATAATATTTATTAAAAGAGAAAAATAGAAATAGAATCGTAAAAGGAAAAGAAAAGAGATTTATTTCTATATAAACCCACACCCACAAAACGTCTCATTAACACCACCAACCCTACTTTTATTCAACGAATATAAATAAATTGGAGCACTGCCAATTGCCAAAGCGCTCCAGCTCTCTCTTCAACCTTTTTTTTTTGTTCCCCCTTCAATTTCTTCATCCTCTTCCAAGTTT

>Csa1M003510.1

GTAGTAATTTAGTTTATAAATTTTAAAGGTTTTGATTATCTCTATAGAAATTAATTATGACTTTGATGTTTACATTCTTATCATATTATTAAACTTTCATTGATATTTCCACATTTTCAAGAGTTCAACGTTTAACATAATAGTTGAACCGACGTTTTTATTATATTATGTAAAACCATCCACTAAAAAAAGCAACAAAATGTTATAAATATAAATATAATCTAAACATTTTCACTAGACTAATATGTAATTTAATAAATGAATACACAAAGTCAAAGTGAGTTTAACTCAACGATAATTGACATGTATTATTTTATTTTGAGATCGTAGGTTAGATCTCTCATCTTCTAATTAATTTTCTAAAAACTTGGATAAACACTAATTATTGTCTCGAATGTCAACGATCCAATCCTCACCTTTACATTGATTTCAACTTGCAATACAACATACATGTAATTTAGTTTTCAATTTACGTACACTAAACAAACATGATATTTAGTACGTTTTTTTTAGTGATTTTAAAATTGTTAAAATTTTTATGATTATTTGGAGGATTTATAACATGTTCTAAAGCAAAATTTTGAAAATGTAAAAGTGATATTAACAATTTCATAGTTCCACCCATGGTTGCCTTGAATGTTTAGTGAGGAATATCTTTGCCTCCACATATCACAAGAAAAAAACAATCATATTAATAAGATAATACCATTAATGAACAACAACACAAACACAAAGGACTGTAAACCTGAACCACTCATCTTCAACCTATAACCACTTATGGAAAAAGCATTCAAAATTTGAAAATTTTCAAATACCATGAAGATTCAAACATCCAAAGCAAAAGTTGATGATCTACTACCCAAAACAGAGTCTAAAAATAGCTAAGTATGAATGAGTATGGAAAATAGATAGAAAAAGGAAAAAGAAACAGAAAAAAGTAATTGGATTAGTCAACAAAACCCACTTAATTGGTCAAACACATCTTCCTAATCAAGTTTGTTCGCATGAATTAACAATAATTAAACCCCAAAAAGGAAGAAAAAGTCAAGCTTTTTTATGATATTTGGACCAATCTGGACTGAAAGACCAAATATATATAAAAAAGATTGACTCTGAATTCGAAAAGGGGTCAGCAGTATCTGAACTCTGAAAGGCTATGAATGAATTAGATGTTGAACAGAATGATGGGTTTTGGAATGGGGAATTGGCAAATGATTAAATTTTGTATTCCCCATAAAAGCTTAGCCTTTAGTTTAAAGGAAGCTGCTTTAATTGAAGTGAATGAAACACACGAACGGGAAAGAGTTTTAGAGTTTTAGAAAGTGGGGGAAATGAAAGGGAAGTAATAGTCCAGGCAATTCCATATACAATGGCTTTGTCTCCACGTTGAAAAGGATCGGTTTCCATGTGCCTTTCATTAGTTTACGGCTCCCACAGTCTTTATTACTTTATTATTCATTCATAAACCCTTCTTCTTTTCTCTCTTTCTCATACCCTTTTCTTTACTCAACACACTCCTTCTCAGTCCTTTCCATCATTCTGTTTGTAGATCCACACCTATATCTTCCTTAATCACCATTGATTTTACTAGAAAAGCCATCAACCCCATTTCAGTTTTTGCTCCATTTTCTTTTATTCTAAGCTTTTTGGTTCTTAACTCTTGTGGGGTTTGCAATTGTTTCATCTTCTTCCCCTTTTGGGGATTTTTCTTCTTTCCCCATCAATTTTTTAAGATATCCCTCTTTAGCTTTCTGGGTTTTGAAGAATATGAGGGACGTTTCTCTCTTCCCTCCACATCGAGATTTTGGTCATTCAACTTTTCGATTGATTTCTGGGTCGGTTTTTGAGGTGTTTTTGTAGGCAGTACAGCGTACAATTGGAGCTCTTAATCATGTATTCTATTGAGCTTATCTACTTGTTGATTACTTCACATTTTAGATGGTAATTGCAATTGCTAGACTTTTTTCATTGATTAAAATGGTTTGTTCTTTTCTAGAAAC

>Csa3M119370.1

ACCATATAAAAAATTCCCTTGAATCCTAAAGCACCACACAATTCTCGAACACCATTTTGATCAAATGAATAAAGTCTCCCCCCCCAAAAAAAAAAAAACAAAAAACTCAGGCAGAAAACTAAAGAAAGTGCCGAAAGAAGAAAAGGGAAATGTAAAATGGAATTTGTATTGTGAATAAAAGAGAGATGGAAAACGAATCAGAAGGGAAGATTGGTCTCGATTCTGGTTTTCAGAGATTTCAGCGAAATTGAAGGGAAGAACAAACACTTGTTTGGTTGGAAGGAAACTCAAAAAGAATATTGTTTGAATATGAATTTGAATGTTACCATTTTTGGAAAATACCTTCATCTCATTAAAATTATTCCTTATTTTGTACATTTTCCAAAGTTTAGAGGATTAATTAGAATTAGGGTATGCTGATATGGATTATTGTCACGTCATATTTTTCTTCTTTTAATGAAAATTTGATAGAATGGTGATAACAGTTTTGATGTTTTGTTGTTTGTGTTTTAAAATTAAGTCGAACATACCAATTTCGTGTTGTTATCTATTGTTAATAAAGTAGCATTTGTGAAAGAAAACTCGTTTGCCTTTACTTCGGTCTCATTCGAGGTTGGGGTGGGCAAGTTCCCCTTGCATCCCTTGTCCCTTAGTCTAAATTAATGCACAACCTTTACTTACTAACATTTTTTTTAAAGTCTAGGACATACTTTTAAATTGAAAAATGGTTTTTAATAAGGAAAAAAACAATAGCATTGATTTAAATATCCACAAACTGTTGAAGTTAAAAAGAAAAAGTTGATTCCACAAATTTTGAATCCTCCAAACTAAAAAAATAAAATTATAAATTAAAATGTAAAATTTCAAAATCTCACAAAATTTTTAAAACAAGAACGTATTGTTAAAACAATAATCAGTCCTTAATTGTTTTAATATCTTTCAACTAAATGATTAGTTTAGTGATGTTTTTAGTAGTAAATAGTAAGAGTGATTTTGAAGAAAGGGCGGGCAAAATATGAGAAGGAATTGGAGACAACCAAAAGGGAAAAAAAGAGAGAGTAACATTTCCAAATATAGACGAAAATTAGCAAAAATGTTAAAACTTTAGATCCGCCGACGGTACCCATTTCTAACGAGAGGGTCCCGGTCCCGATGGTTCACTGGCCACCCTCACTCAGATCGCCGTTGTCGTTGTCTTCTTCTTCTCAATTCCCTTCAATTCCACACTCATCCATTTCAACACTCCTTTAAAGCTCTTGAGGTTTCTTCCCAAATTCTCAGGAAGTCGATGATGGAATCCAGGGTGTCGAGAACCAGACAGAAGAAGGCCGATCTTCACTCTATCCTAAGAAAATCATGGTACCATTTACGTCTCTCTGTGCGCCATCCCTCCAGAGTTCCTACTTGGGACGCAATTGTTCTCACCGCTGCTAGCCCTGAGCAAGCTCAGCTTTACGAGTGGCAGCTCAATCGGGCGAAGCGTATTGGTCGAATTGCTCACTCGACTATTACTCTCGCTGTGCCCGATCCTGATGGCCAGCGGATTGGCTCTGGAGCCGCTACTCTCAATGCCATTTACGCCCTTGCTAAGCATTATCATAACCTCGGCCTGGTTCATAGTACTGAGGTAAGTTTCATTTCAGCTCGTTCGAAACCTCGTTTTTGAGCCTCGGCATTGCTCTGGTTGTGGTTCTTTTCTAATTCTTACTTCCCCTTCATCAGCAACGCGATGTTGGATTGCGTGAATTAAAATTACTAGAAGTTCCAACTCCAATGTTAGTCCTGTTTTTAGCTTGCCATTGAAGGATGTATGAGAGTGCTGTTTTCTATATTGGTTTTATTTTAGTTCAAGTTTAATGGATTTAAGAATTCTGCTATTCTAGGTTTATCTGACATCATTTTGTTTTTGGGTACATCGTATTATCGATCATGTTATAGGTGGATTCTATAGGCAATGGATGTGGTGAGTCTGATCTGCTGCTGCCTAACTTGTCAAACAA

>Csa3M133150.1

CAATACCTCGTTCAGCAGAAGATCTACCTCTAAGGTTTTGAACCTCTTTGGGGGGGCAACCATTTTGTCGGTAGCCAGCATGTTGCAAATATCTGTATTTTTCTTCATCATTAAGTGATGGAGCATAAATAATTCCATTGAAATTATTCTTCTTTCCATCAAAATTGCATTCTTCATATGTTTCATTATCACTACCAGATGCCAACTTTCGATGATTCTCATATTTAGCATATTGAAATTCCACGCCATCTCTCTGTTTCCACAACGTTGGCAAATCCTGCAAGGAACTTGACATTGCAAGTGAAGGTGACTGATCACTATCATTGAATGAGGTTAGTGGATAGGGCCCTCCATGATTCGGCTTTTGATCCTGCTCCAGCAGTAGGGGCTCAGAATGCGAATGGATAATTCTTTGTCCCATATGGGTGGTTCCAGATCTTAGCTGCATCAAATACTCCGCGTCAGAGCATAGTTGTTCATAATTAACTGCCCGTTCCTTCAGCATAGTTTTCATTGGATGTGTATCACTTAGACCCCAGATGGGTGCAGGAACAAAATCCTCACTGGAGCTTCGTGGGAAATGAACATTAAGAACTTTGTATGTCTCATCAGTTTCCGCAGTAGATTTTTCATGATAATAATTCATCAACGGAGAACCATGGTAAAGATAATTGTGACGGCCCATTGCATCAACATAATAAACTGAATCACATGGATGTTTTCCTGAAACAATTGGAGAGAAGTTTCTAGCGTCTTCTGCATATGTTGCATCCACGTTCCTAAAATCTTTTTGCATTACAGTAACCGGAGAAATTAAAGGTGATTGATTTAATGACTTGCGTGGGACTTGGATGGGGTTTAACAATTGACCACCTGGTCTAGGTAACATTCCCATCAAATTTGGCATTAGTGAACTGACATCTTTCACGTCTGTAGCATGTGACGAATCAGTACGAAAATTGGGACTATGATCTGAGATGGCTCCCACCTGGCTATTCTGGCTTGTGAAACTCTGCCCACTGGAGCTCCTTTGAAGACTCGGGTCTAGAATACCGTTCACAGCCGCAACATATTGATAATCCACATCAATTGGTGGAACTACCCTTCCTTCAATAGAAGTAGGACTCTCACTACAATCATTCGAAGAAATAAGAAAAATTCTAAGTCTCTGAGAACCTTCTGCGTTTTCCAGCTCCTGATATTCCTCTATCATATGATGAAGATCCTCATCAGAGCAGACAGAGATAAGTGAATCAAGATCTTCACCAGGAAGCTGGTACTTTATTGTGTGGGCATATTTACAGACAGCGTATGTCTTTTTGGTAAGTTCTTCATATGAGATGTTTTTCCTAATGGATATGATGCGCGTTTCTCCCGCTACATATCTAAGCTTCCCATCATTAGGCCTCGGTAATATTCTACCTCCAAAACTACAGAGAAATTTCATCTTGTCATTGGCAGAGAAATCTCCAAATCCAGCCCCACAAGGATAACATGAATGAGGCGAATCCAATGGATACAAGGCCGACATCATGGGGCCAATTTGAACTCCTCGATTAGTTTCGTCGGCATATGCGGCAGATGCTTGTCCAGTACCATTGTTATATTCCCACTGACATTTGCTTGTGGTATTAGGATAAACCTTGTTGTCCTTTTCAGCTGCATAAGCAGTCATTGGCGTTGATGACATTTCTGAACTACTTTCAGAATTCATCCTCTTGAGTCCAAGAATTCCACTAAGATCCTCATACACCAATTGGTTGCTCGGATTGAACCCAAAACCTTTTCTCTTTTGCTGCTGTTGTTGTTGATCTCCATCACTCATGTCAGAAAATCTTCTTAACGCAACACGATCTCGCATAAACTGTGGAGAAAATACCTCACCCGTCTGTACACAAACATCACTCACACTATGATCCGCAGTAATCCGTACATCCGAAACAACACTTGGTCTTTCCATGTATAATTGTTGGCTTAAAACGCCATGTACTTCCCTAGCC

>Csa3M182770.1

TTTTCTTTTGTTTTAATCCAAAATTATGTGGAAGAAGCAAAGAAGCGGCTCTAAATTGAGCTCACTCTCCAGAGCGAAAATATTTTGTAACAATAGATGCTAAGAAGATGTTACACTTCCAAGGCATCTGACAAGTAAATGATACGCTTCAATACCAAAAGTAGCTTTCACCCCATAGGCACACCCTGGTAAAGCTTTGATTGAGTATCCAAACTACCACTTCAACTAAATTTTGAGAAGGCCCAATAATCAATTACATGTAACAAAATCCTGCATGAAAAGGAAGTGGAAGTTTGAGTACTTAAGATATCATGAAGATGATGTATCTATATATACAAACATATTACACACACACACTTCTAAGAAGAAATGTCCTGATAGCCAGAACGAAAAGTAAGCTTGTGCATGCAGGAAATTCAGGATTTCACCACTTCAGGTAAAACAAACATGTTTCAAGCAGACTCTGTATCCATTAAATTTATATTCTTCAGCTGGAATACTGCAAAAACTGGGTAAGTTTCTCTTAACCTTTGGAAATCTTCCACATCACATGAATAACCGACAATCACAATCTTACAGTTCGAGTCTGAGGTTGAAAGTTGAAAAGTAGAGGTAAGGCTATTGGACAATTAGAGCATTTCTTGGTTGATATGGTCTACAATAACAAAATACTTTTGAACATTTAAATTAAGAGAATAGTGGACAGATTTCAAGCACTATTTGACTCTCGTTAATAATCCCCAAATCTATTTCTTAATTGGAGACAGTCAGATTTCTATTATCTTATCATTGATAAATTTGAGATGCTTTCACAATCCCCAAATCTACTTCTTAATTGGGGTTATAACATTTTGTCCCATTTCTCACGTAATTACTTCTATTGATATAAAATATAGTCCATGATCGGCATTAAAAGAGAAGTTCCATGGATGGATATAAGAAGGAAAAAACAAAATATAAACAAATAAAGAATCCTCACAATAAAGGAAAAATTAATGGATAATGAGAGCTTGAGATCATTAATGTACACCGGCCGAACAAGTAAAAACGATAAATAGTCAACATACATATTACATAATCAAATCCAAGCTCTAATCACTTATATCATTCGATAGAAACCCAAATTCCCACTGCAACAAAATGAACTTAACCATTCACTTCCAAATTTACTCCTTTTCTTCCCCACGACTGTTCAAGAAATCAAACAACGAGCTACACGTCCATATCCCCCAAGACTCTACTCAACAATACAGATCTACCAAGAATCTAATATAGACAAACTAACCCACAGTGTCTAACGTAACTAAAACCAGATAATCAGTAATTTTACTAAAATTAGATTAGAATACTGAAATGAAGAAGTGGTACCTCAAATTTTGGAAACTGCAAATTCCAGTAGAATCCCACTATACTCGCCCAAATCCTAGTGAGGTAGACTTCAACAATAATTCAAAGTGAAGAGAAAAATTAGTGAAAATCGAAACCCTAGTAATCTGAAATCACAAATTATCAAAACCGAAGAAGCGTTGCATCAAACAATCTTTCTCCATTTTCATGTTGAATTGAATTTTGAGATTGAGAGAAAAATCGCCAAAAAAAAAAAAGAAAGAAAGAAAGAAAGAAAGAAAGAGAAAGACTAAAGAGCGTATGCATCAGCGAACGACATGGAGTTGTCCGAAAGTTCATATTTTATTAATTATATTTTAAACTTTAAAAATTAAAAAAAGGCGTAAATAAAATAAATTATTTCATCATTCATTCATTGCTTCTCTTTTCTCTTTTTTTTTTTCCTTTGTTTTTCATTTTCTCTCTCTCTATAACCTTTCGCTTTAATAAACTTTATTTTTAGTATTTGATTTTGTATCTAATTTTTCAAATTATTTTAATTCTTACGTAATTGTCATTCCAATCTTAGTTTATGTCAAACATATAATACTAATATTTTTTCATTCTAAATATTACTAATAAAGAGAAGGGATTATTGTGAAAAATATTTAAAT

>Csa3M146410.1

TACTGCAAACTCGATCTCGCCCGTCCTTTGTGAGGAGGTTTGTGTTGGTGGTGAGTGGCTTGAGTGCATTGTTATTGTTAAAAAAAAAACCTTAGAAGATATGAAAAATAGTCCTAACACACGTATACCTATGTGTATATGCCAATACAACTCTTGCTTATCATAATTTCTCAAATCATTTCTAAAATTTTAACTAGAGGATCAAATTTCTCTTTTTTCAATGAATCTCGTCTCTAACATGCACGTTTAAAATAGAGGCATTTTAAAAAACAACAAAATGTTGAAACTATTTACAAAATTATATTTTATATATAGTTCATTTTTCTTTCAATAACAATTCATATTTAAAATAAGGTAATTACCTTTCTTTCTATATATATAGTTATAAGTTAAAATATCATTTTGTTGTTCAATTTTGTTATATATAGTTCTAAATGTCTAATTTGAATTTGTGAACGTTTAATTAATCTTAAATTTAATTCTTCTGGTTTGTATACTTTTTTTTAATATAAATCATAAAAATATATTCACTTATTGTGTGTTTTTTTTTTAATTTATTTATAAAAATGACTGTGATTATAATTAATTAAAATAAAAATTAAGTTTAAGAGAATAAATTTAACCTTGATTAGCTCCAATTGGAACATTTAAAAACTTAAGCTAAACAAATTTCAAATGACGTAACCAAAGTTATATTATAACCATTATTGTAATATTGGCATGAAATTAAATTGAATTACATGATTGGAATTAGGTCAAAAAGAAAAAGAAAAAGGTAGAATTTTTGGCCCATCCATTCGTTGAATGTATACATACAGGTAATTCAGTTGAAACAGAACTCCATCTCCGGGTTTTGTGCTTATCCTTTTGCTAATTTGTTAATAAATTTTGTCAATGGATTTGAAAGAGTCAGTATCATTTCCGAACCCTAAGATATAGTGAAATCCCCGAGCTTTTCTCTTCATCTTTCCCAAAATTCTCTTGTGTTTACCATTCCCATTTGCTGGTTTCTCCATTTTTAGAAAATTTGGTTTTCTATTTTTGCATTGCCTTGCGGTTCTTTCTTCGTCCATAACCCTTCTACTCGTCCTGTGGGTTTGCTGCAGCGAACCCATTTGGACAAAAACAATTATTACAAATCCTTTTTTTTCTTTTAACTCTCTGGTTTTTCATGATAGCGCCATTGGCGCTTCAGTGATTTTCGTTGGCTTTGTATCATCATAAAAAGTATAATACCCCACTTACTATCTCTTTCATTTTCGTCTTTCATCTTCATTTGGCTGTCTTAGTCAACATTTACGGTATTCTTTTAGGGGCTGCGGGACCGATTTTGTTGAGAGGTAATTACTGTTATTGAATTGTCTTCTTCCTTTTTTTTTCTTTTTTTTTTTTGCCCTTTTCGTGTTTTACCTTGAATTTTAGGGTTATGGGTGCCTGTTATTGGCCTTTCAGTACTTATGATTTGGGTCGTTTTCTGAAGGTTTACTTTTATTTTTTAAAATCAATTTGGATTTTGGACTCCAATGTCAACTGGGGTTTTCCTGATAAAGTAGAATAATCTATTAGCTTTTCTCTATTCTCTGACTATTGTTATTTCTGATAGCTGCCTATTTTGTTCCTGAAGGTTAATTGTTATTATTATTTAATTGAGGCATTGGTCAACATTGGTGGTAGTTACTCGTTCCTGTTTGTATTGGTGGATGGTTGTGTTGGAAATTTTGGTTGCTCTTTCTTTTTTTCAAAGATTAAGCATGCCTATTGTTTTCGAGTTGAAATTGATTGGTGAAGTAGATTCAAATATGGGAGAAGTCTGAATTTGATCCTTTATTGGTTTTTTTTTCTTTTTTTTAACAGATTGAAGGATTATTCACGATAATGGAATTATGACAGACAACAGCTTTCTGCACAGTTGGCTGCATTCTTCACCCCGTTTGATCTCTCAGTTTTAGAATTCCTTGTACTCAAGAGTACTGTTTGAGTTATCTTTGTTGATCTTAGCA

>Csa3M728150.1

AGTGCAGCCTCAAATTAATCCCGAATTCCTCATAGTGATACAGAGTAAACCCAGTTTCCGGATTTGCAATCAGTTGAAAAGAGCATGGATGGTGGGTTACGTATATGAATTATGAAGGCATTCAGAGGAGCAAAAAAGTGGTAGATTCAAGCTTCCCATAAACGAAAAGAAAAGGGAGTGTTTAAGGTCCCGGGCGGGTGATGGGTTTGGAATAAAAACTAAAATTAAGTTGGCAATCGAGAGAATATAGGTCCAGTATCCGGGGAGTGTGTTTAAGGGTTAAAACTTAGTTGGGAATCAGTAGTTTGAAAGTATGGACACAGCTTTGCTTGATTTCAAAGCCTCCAAAGAAACCCTAGGAAAACTATATTTAAAAAAAATAAAAATAAAAAAATAACTTATAGTTGACGCCAAAAATAGAGAGGAAAAATTAAGAAAAATCCATGGGGGGGTGGAGAGGTCGTTATCTAAGTTGGAGTCTGGAACTTGATTCTCATTTGCATAGTTGAACAGAAGAACAAAGCATCTGTATTAAACACATAAAAAAAAAAAAAAAAAAAAAAAAAGGTGAAGAAAGGGGTTAGGGGGAAAAACAAAAACAAAAGAAGAGAGTAAAGCTCTTTCTCTCTCTGTACCCACTGAGGAGAAAGAAGCAGTCCACTGGGTGCGTCTGTGTTCCGACGACAAGTATGGATAGTTTTTGGAAAAAGAAAAGAAGAAGGTTAGTCCAATAGGAGTTGTAGAGGTAGAAGTGGCATGGAGTGGGTTTGACGTTCTTTGTTAGAATTGTGAATTGCGTTGGACTTTGGTTGGGTGGAGTTGTGGACTTAAATCCTATAATGGACAAACCCATCCTACTTGCTACCTACCATCTTCCTTTCTTTTTCTTTTTCTTTTTCCTTATTATTATTTCTTTCTCTCTATCTTTTTCCTCATCTCATCTCATCTTATTAAATTAAAGAAAGATTTTTAAAAAATAACTAAATTTAATACAAATTTTCACAAATTTATGATTTCCTATATTTTTAGCACAACTTTAAGTTTTTAACCTTATTTTTATTATTTATATACCATTGAATATATAGATTATGAAATCGGTATTTTTTTAATTCTTATATATATATTGTTGCATGATACATATATGTGATTTGTTACATATATATTGTTTTACATATTAAATAATACATAAATGATGTTAGCTTCAATACACCCTAATGTATTTCGAATACGAAATATAAATTATTACTAACCTTAAATTTTTGGCAATTTTTTTTCGAAATATCACTATATCAGTCAGCTACCGATGTTCTTCTTCTTCACCGTTTATACTTTTCCCAATCTTCTATTATTCGACGTCTTTTGTGAAGAAAATGGAATCAATGTATTCGAATAATGAAACTTTTACATTTTTTAAAAATATATATATATAAACCGTAACCCATATCTCTGATTTCTCTCGTTGTCGGCCCACCTTCTATAACTAAATTGATATAAAAGTCTTTCATTTATAAAAATATACACCGTATTATTCAATAGATACTATACTTAGGTATAATATACTAAAAACAATGCAAAATGAAAAAAAGAATTAACGAACTGAGAGATTAGAAAGTGAGAGATGTGGAGGGTTAATTTTTTTAACAAAATAACTCAAATAAAATTAAGGGATAGTTGCAAATTTGATAATTAAATTTAAAATAATTAAGTATATAGCAACATTTTTAAAAAATTGCAAATATAGCAAAACTATCACTGATAGACTTGTATCGNNNNNNNNNNNNNNNNNNNNNNNNNNNNNNNNNNNNNNNNNNNNNNNNNNNNNNNNNNNNNNNNNNNNNNNNNNNNNNNNNNNNNNNNNNNNNNNNNNNNNNNNNNNNNNNNNNNNNNNNNNNNNNNNNNNNNNNNNNNNNNNNNNNNNNNNNATATTCTTAATTATTATTCCTCAAATGGCCATCAATTACAATTACCCTAAAATTAATTAAGAAACAAATTTATTACAA

>Csa3M749850.1

TCAAAAGCAAAATTATTTTTTTCATTCGGTATTATGTATGAGATGGCCGCACATCACCCAATATTTAGAAGTATGCGCTCTCTAATTCTTTAAAAAATCCGCCCTTGTTTGTATTATGATAAATAAGTTTATAGTCTCCCGTGATTCACCCATAAATTATTACACTTTATTATATTGTAAATATTTTAAAATTTTATATGCTTTAAAATAATTTTCCTTTTCTTTTTAATTTTAAAATTTTCCTTTAATAATAAGTTTTTCATTTTTAGTTGGGTTTGGGGAATTTAATTTAAACTTGACTTCCTTCTCCTTTGCTTATTATTTATTTTCTCTCTTGGTTGAATGGCAATTGACGTGATTCCCTTCCACTTCCTTTAGATGACTAAAACTTAAGGTTTAGTATTCATTCACTCGTTTTGTAATAAATGGTTCAAACTATGTTTAGACCAACAAAATAGATTTATCAATTATTAGTAAGTTGGAGTCTCCTACTTTAATTGGTCCATTAATTTTCAATTTTTAGTTTAAATTTTGTGTACTTGCCAGTTGCAAGAACTCTAAAAATTGACTTGTTGAGTGTGGTTTATTTGATTTATGGTGTGAATATACGTATCTAAACTATATATAAAATGATATACGGAGACTAATCTTTACCTCGCAAAATTTTCCTTAAACTTGTTTATGGTTCGTATTTTTTCTTGCACTTGCATGTGATGTCAATATTGTATACAATCCAACTTATGAATACATGTTTAGACAAGCAAAGACATTTTCTGTTTTTTTTAAAAACAACAACGTGATACATTATCTCATTTACGTAGGTATGGCCTACCCAATTGATATGAAATTTATATCGTAGACACAATTCACTTGTCAAATTACGATTGTTGGTGTCACATGTATAATTTTACTTGCATTAACTTTGTCACAAACTTAAAAGTCAAAAGTTTTATCTTCTAACACTGTCTGTCGATAAAAAGTAGATAGGTTTTATTTCGTGCTTTGGTAACAATAATGACAATAGCAACAAATGCATGCACATTTTCTAATTCATAGTCGGTTAAAAAGGTAATGAACACATTCAATGTAGATAGTTTTTAACAACCATTTAATTTATTGTTTTTAGGCGTGTTTGATATGTCATAGTTTCCAACTCAAATTTTCACACCTTTGTAAGAATCAACTATCCATTCTAACATATTTGAATTACTAGTCAATTTTGTTCATATATTAAAAAATAAACTAAAATATCATATTTTATATAAGTTAGTTTTTATAAATAAACGAAATTGTTAACATATTTATGGACCATGGAATAAAATTAAAAAAGATTTCACATTTGTTATTCTTTTTCATCGTATTTTGTTTATAAATATTGTATTATTTGGTTTAGTAGTGGATCCGGAAAAAATTGGTAAATCTAAACTATGAAGTAGAATAAGGAAATCAAATATTTGAGAGGAACATTCTTTATATTTTATATTATAGGATTTTTTGTTTTTGAAAATGATGTATGGAATGCTAATATTTTTTGGTTTGGTTATATTTTAAAAATGCTTTTAAATAACTAAAAAAAGAAAAGAGAAAGGGATGGAGTAGAATGGTAATTGAACCACCCAATTTATTACACAAGGGGTGAAAATTATGCGCACGTGTCAGAGGAGGTGGAAGGGAAATGAGCTGCGTCGTCTCGAATAAGCTTAGTGGGCCTATTTAAAGGGCGATGGAGCCAACTGCGAATTTGATTTTTATTTTTAATATCTGATATTGGGCGAAATAAATTAATTAAGAATTTATTGGACGATAGAATATTACGAAAACGCCACTACGCCTAAAAAGTCAGTTCTGTATGGTTCTCAGTTTCTCTCTCCGACAATCGAATTCCAGAAAGAGACACACCGTAAAGGTGAAAAATCGAAGAGAGGGGAATTCTTCATCGATTCGATGGTGGATTTTAGGGTCCAAACGGATTCCAACTGATCGGAATTCTCAGAAGCTCG

>Csa3M829110.1

GAGGGCGAAATCTAGTACCTGCCGAAAAACAAAATAAAAAGATGACTTTTTTACATCACCAGAATGAGAAACGGAGAAGCGAAGTTGTGTTCGTACCTTTAGTTGGGCCGCCAACCATGATAACGGCAACCACTTTCTCCGAGCTCTCCATGCTGGAACAAAACCACAAAAAAAAAGGGAAAAAAAACCCTAGATCCTGTTTTTGAAATCGGTAGGTTTAAACTGAGTGCCTGAAGCTCTGCATTTGACTGCAAATTGAGAAAGTGGAAATTAACTGATAAATTAAGGATGAAGCAAAGTGAAATGAATGAGTTCGCAAATCCCAAAACTTTAATTTCTTTTCTTCAATTTGTATTTGTTCAAATTTTAGTTTGAAGAGAAACAGATCGGAAATCGGAGTAACGGTGGCGAAGGTAAAGTATGTGATACGAATCACTAAATAACACAAATTATAAATCTTATAATCTATTTCTTTTCTTTTCCGTATCTTATTGTTTCTTAATTTGCATTTTTTTATTCATTATTTACCTACATTTCAAAACTTTTAAATCATCTCTTTTTAAATGAAAATCATTCCTTAATTACTAACTTCATTCAGTTAATATAATTAGTTTAGTTATTTTTGCAAAAATATCTTTATAAAGTTTGTGATTATGTAAACAGACCAAAAATAATCAAATTCATTTCATTTTTTATATTTATATTGTGTGAGCTTGACTTTATTTTTTATCAATTTCACAAATAATAAAGTATCTATATTATAGACGTAATTAATAAAAATTATTTTGTCATTTTTGTAAGTGCGTCTATAATTTATTAATTTATTTTAAAACGTTAATATTAAATATACTTAAAAAGAAAAAGAAAAACAAAATACCACATTTGTATAATTTAAACTTAATTTTGTATTAGGATTGGAAGGTAATGTAATATTATATGTATACTAATATACTGATAGCATACATTTATTTACACACATGCAATATTCCATGTTCACATAAGGAAAACAATTCACTAAAAATATCTATAAAAACCAATAGAGTTTCTAACAACATTAATAAGTAAGCTATTTATTGAAATACATAACCTCTTATTGGATCATCTATGGTTTAAAGACTTAAGCTAATGAATCATGGACACTTTAACATCTAACCATTTTCTTTGCTAAAGCATGAGTATTATACTGACTTAACATTAAAAAAAACTATAAAAAATGATAGTGTTATAGTTATTTTTAGTGGTTTTTTAAAAAATAGAAAGAAAAAAAATAAAAATGATAGTGTTATAGTTATTTTTAGTGGTTTTTTAAAAAATAGAAAGAAAAAAACAGCCAATTATTTATAATCTATACAACAAAATGAATAAAAGATAAAATTAATTAAATTTGATTTTTATATGAAATGTTATTTTTTACCACTAATATACAAAACCAAATATACAGTTAAATTTTGTGATAAAAAAAGACAATTATTTTAAATGAGAAAATATATAAATATTGAAATTGTGTTATATTTATAAATAATATTGATTGAAAACGACCTTATATTTAATATATTTTAATATCAAACTTAATTCTAAAATTAGTTAATTAATTAATTCATAGCATAAATTGATTAAAACATAGCGCACGAAAGATGGAAAAATGATTCAAAAAAGGAAAAAATTAGAGCAGAGATATTTAAGGAAGAACACTGCGAATATTACAATAAAATTGTAAAATCAAATAAGGAAAGTTATTAGGAATCGGAATTTAGTATTATTTACATGTAAACCAACTCTTCCGAAATTTCCATGTTCTTGAAATTGTTGGATTTCTTCTACAAGTTCCTCCACCAGGTATGATATTATTTTATTACTTTCTTAATTGATGCATTGTTCATATTATGCTCTGTTTTTTCCTCCATAAATGGAGCATTAACAGAACAATAATAATGAGTAACTGAAGATTGTTATCGTTAATCACACTTGTTAGGTTCTCTAATTTTTTGCAGATTTAAACT

>Csa3M836460.1

AATTTCCCACCAATTTCAACAAACCAACGACAGAAATTGAAAGACACAGAAACAATGGTTCGTGTTCTGGGTCAGTTGAATAATGGGTTTGTTCGTGTGTGTATGGAGTTGAAGATTGAATTAAGAAAGAGATGAAAGGAGAAAGAGAAAATTAATAATAATAGTGAGCACAAGAAAAGTAGAAGTAGAGAGAGAGAGAGAGAGAGAGAGAGAAGATTGTGATTGGTTTCTACTCTGTGTTTCCTTTGTCCATCATCCATGTGGGCACATGGATTATGAAACCCACATCGGTTTCTTCTTTGCCCTTACGAACGCTCTTGTTTGGTTTTATACACACACACACAACACACACATAACACGCACACACAATTCTTTCTTTCTTTCTTTCTTGTGCTTTTCGTTCCTTTTGGGGAAAGTTTATGGTCAAATGGTTAATGGCTTTTTGTTTGACTGTGTTTTGTTATGGGTTTTTGAAGGTGTGTTTTGTAGGAGGGTACCAGCACTGTGCACTGTGCACTGTGCACTGCACATGCCAAAGCAGTTAAAATGGAAGTTATGGAATTTTATGATGTACTCTTATTTATTATTTTTAATTTAGAGTTTAGGGTTGATTTAGGAGGGAGGGGCAGGAGAAAATAACAATAATACTTTAAAATATGCCTTTGACTTGGCTTTCTGTACGAGAATTAGCTGTACATATCTTCTTTTTTTTAATCTTCTCACTCTTTCTTACGTTCCTTATAAATTATAAATTAATTATAGCTTAAGTTATGAAATTTAAAATAACATTCATTGTCTTTTTTTTCTTCTCTTTTTAACAATTCTTCTTTCAAACTTTAGTCTTTGAATTTATTATATCATAAATGATTTAAAGAAAGAAAACCATAACAATAAATTCAACAAAATATAACAAAAATTTAGATAAAAACCGATAGATTTAGTTAAAAAAATTATTTTATTTTATAAATATTTAAGCAATTTTACCATTTACAATCATTTCTCTTTCAGTTTTTGTTCAATAATTTTAAGAAAAATTTTGAAATGTCTATGAAAATTGAGGAATCGTATTGCTTAAATTACAACTTTGCTTATGATTAAAGAATTTAGAAAAAAACTGCTTACAATATTCTTTTTTTCATATTGCAAATTTGACAAATATAACCGTAATCATATGGTAATCTAACGACAATCTACTTTTAAATTTGCTAGTTTTACAATTCTGAAAAAATATAGGTGATATGAACTCTATTATTGTTATTTCTGCAAACTCTTCCGTTAATTTCACTTAGTATAAAATTCTTTCCATATTTGAAAGTGATGCAGAGACGATTATCCATTAGTACAAGGGTATAACAACTTTACTTGTATAACAACTTTAATCTCCAATCTCAAGAAAAAGGGTTTGTATCTCAAACTACATGCCAATAACAGATGACCATCAATCTTGAAAATAAAATAAAAATTAACATTATTATTTTACTAGAAACTAACATCGAATATATTGCAAAAATAACAATCATGCTAAAAGTATTAGTAATATAACAAATAAAATAAACTATAAATAAAGTAAAATTTAAATTCAATTCTTAAAATCTACCCGTTACCAATTCTAGTATTGATAGAAGATTATTATAGATTTTGTTTCATTTACAATTCTTTAATAAACGATGTTAAAAATTACGTTCAAAAGTGTACACTAGTAATAATGAAAAATTACCTTCAAAAGTATACACTAGTAATAATGAAAAGGAACCAATTTATTTATTAATCTAATTACAAAGTTGATTTTTCTTTAGTGCAAATGTTGATTTATGCAAGAACAAAAGCTGGTCAGTTTTGTATGTCCCCTATAAATTGTATCTAAAAAACTTTAGTTAGGTGGCCAAACAATGAATGCATATTATACAATCAATTTCCCATTAATGATTGAAGAATCTATGGCTTCCAATGTTTGTGGTGAGACAGAGCCCCACTGGCACCAAGCCTCCAAAAACGAGAAA

>Csa3M840390.1

TTCAAACATGATTATTATTTTTTTATATTCATAATGATTATTTTTCTTACGTTTTGTAAATGTTTTAGGATATAGTTGAAATGACTATTTACTATCTCAATATCAAATCTAAAAAATGTATATATATATAAACAAACAAAATTGTGAAATACTTATTTTCCTCTCTAAATAGTTTTTGAAAACAAAGTTTACTTTAGTCACATTCATTAAAATTTTGTTAATTCTCTAACGTAATAATAACGTGGATATTTTGATGTTTAAAATTCAGATTCATCATATTAAATATATTGATAAATAACTAATAAATCTTACTAATTTATTCTCTAATTTTTCTACGTCAACACCACACTTGAAATAAATAAAATTAATAAGAATTAGTATATGAGTTAAAGAACGTGAAGTAGAATGACAATAAGTCAAAATGGTGATGATCAAAATTCCGAAGACAGAGTAACCTTTTAAGTTATTCGATAAAACATCAATTTACCAAATAGTTTTTAATCAAAAATTAAAACCATATACTATTAAGAGTGGCTTTCTTATTTGATAGTCCTAGTTATTCATATATAGAAAAGTCACATGTGATTCTAGTAAAGTTAACCGAATATTTTAACTACAAAAGACTAAAATATACTTATTTTAAAAGTTTAAAGACTAAAACAAATACTTTTTAAATAAAAGTTTAATATCATAGAACATATAAATGAATGCAAACTTATAATAATAAAAAACATAGTACAAACGGAAAATGTTTGTAATAAATGATTGTGATTGAATTTTATTGTAAACTAATAGGTGTAAATAAAAATGTTGAGATTATTACTATTAAATATTATTATGAATTGAAAAAAAGAGAGAGTGTTTGGTGTACCAAATTAATTTAAAAAGATTAGGGAAAGAAAATAAAATGATTGGAGAGATGAATGTTAGAGAATATTAAATGTCCAATTTCTATAGAAAAATATTATGTAAAATTACGGTATTTAAATTTAACTTCTAATCCAATATTTATTTATAATAAAGTATGATAAATGATAGATAGAATAGAAAGAGTAGTGTATGAGGATCACGATTGATTGTAAATGAGATAGAATACAAATAGTAGTGTATGACGATCAGAAGGATTTGTATAAACGTATTTATGTTGATTTAGAAAGAAAAGAAAGGGAAGAATTGATAGTCAGAAAACAAAAAATAAAAGTTGGATTATGAAAGAAGACATTATGAACTTTGTCTCTATCTCTCTCTCTCTCACACACACACACACTATAAACAAAAACAAAGGGATTGTTTAGTGTTTTCTTAAATAGCAATTTTGGGCTATGCCCAACTTTGTCCTTATCCATGCATTTGGATTTCCCATTCTAAACCCCATCGTCTCTTCTCTCTCTCTCTCTCTTTGTTTTTATTCTCTCTATTTTTCTTTTTTTTGGCATTCAAAGTCAAATCACGAGACGATGTACAGTGACAATAAACTCACAAAGAAAGGGGAAAAAAATAAAGGGAAAAAAAACTCTCTTTTGCAGCTTTAAATTGCAGATGGCAACCGCATTCACTTCCATACGCGGAGAAGCATCAATTTCACACCTCAATTTTGTCCCTTCACTTTCATTTTCATTGCTTTCCTCCCATTCAATTCTTTATTTGCCTCCCCTGCTTCTGACACTAACTCTTTTTCTGATTTCTATTGTTTTTTCTTTTAATATTCTGGGGCTGCTGGATTTGCGTTTTCCTACATGTGGAGAGGCACTATATTCTTTTCCCACCAAGCACAAGAATCCGTTCGATTTCTGGATCGGTTACCGGGATTTTAGGGATGGGTCTGTTCAGTATCAAACATGGGTTTTTTTTAGTTTTTTTGTCTTTGGTTTAGTACTTTGTCCGTACCTCTTTGAATCTCTGATGGGTTCTTTTTGGGAAAGGTTTTCGTGTGTGAATTGAGAGAAGAAGGAAGTGAGGAAGTGCACTGAAGGTGTTTGATAAAAGTCGCGAGTGAAGAG

>Csa3M892210.1

TTTTCACAGAGTAGTCTCAAACACCACAAGAAAAATAATAACGGATGCTGCACACAACACAAATTCAACCAAATTACTACACGTACACCACTTCTTCAACTCTCTCTCCCTCCCTCCCTCTCTCTCTCCCTCTCTCAATCTCTAACTCACTCTAAATCCTTCGTAAATCACAATCTTCAGCTTCTTTCACCACCTCTGCATACGCACAAACAATGTCCGAAGTTCCATTAGCATAATCAAACAGTAACATATACTACAAAAACCACATGAAAAAAAAAAAAAACAACAACAACAACAAAAACACATAAATTAAAAACGAATTCCACAAACAAACCTAAATGAATCAAAACAGCTTCATAAACAACCAGAATTCGTGCAGTAGCTTATTCCGAAGAGTACAGGCAAAAAAAGGAAGAAGGAGATAGAGAGATTACTTTCCAGCAGCAGTAGAAATGAACGGAGCGCGTAAACCTAATTTATGGAGATGGAAAGAGGAAGATAGGAAGATGAAAATGGGGGAGAAGGAGAAGAGGTTGTGGGATTTGAGGGGGGAGAAAAGACGGTGTGGCGGAGGAGAGAGAAAATGGGAGAGCAATGGAGGGAGTTGGAAGTGAGTTTTTTAAGCACGAAAATTGGAAGGAAATGAGTGGCCTATTTTTGTCAATTCCCCGAAATGAGAATGAAGGGAATGAATAATAATGAAATACAAAAGAAACGTAATTTTGTAATATTATATGTAATGTAATGAAATAAAAAAGAGTGGTGGAAATAGGGAAAACCGGGGGCGTACATGCACGCCTTGCGTTGTTAAAGAACACGTAAGATTGAGCTCAACGCTCCTTACGACACGTAGCATTTAACTCCCCATCCCAACAGATCTGAACTCTGCTTTCCGATTCGCGGTTTCTTCTTTTTCTTTTTAATATTTTACCTTTTACTAATTATGTAAACGTACCACTCAAAAATTTGTCATATTCCTCAACAATTTACTTCAAATTATTATAGATAGAAATAAATAAACTATTTTTAAAATATAGAAAAATAATAATTAGGTTTAGGAGACATAATTATAAATTATCAATTATATTTAAAATAGTTAAAGATATATTAATATTTTAAAAAAAATTTGTAAAATCTATCGCTCGATTGATAACAAATAGACTATAAGGGCTTATTAGTCTAAATCTTGTAATATTTGAATTTTTAAAAAATGTTGTTATATACTTCATTATTATTTCTAAAATTATCATAAATTTTAAGGATCAAATGGCTTTTGATATGTTTTATAAATAGTTTGAATATTTTGTTGTTTTTTGAAAATCGATAAATTTTTTAAAAAATTATAAGTATCAATTTATGGTTGAAGTAGATATCAACTTGTACTTATAAATTTTGGACCTATATCGATTTAAACTCTCTAAGCTAATTATTGCATCATTTTAAAATATAAACTATTGGATAGTATCAATTAAATCTCAAGCTAATAGTTTTATTAGCTTAAACTTTAAACTTTCATCTTTTATATATACATCTATTAATATAATATTTCATTTATATAAGTTAATAGTTAAATTGATACAACCCTCACCGTTAATTGATTAAATTGATACAATTATTAATTCATGATTTAAATTGGTATAAATCTCATAATATAAGAATATAAATTGAAAATTTATCTTCTAAATTTAAACATTCTAGAGATTTATTAATTTGGTTAAGGAATGTTTATTTGAGTCTCAAACTTTTGAAGTTGTAAATTGATGTTTGTTTAGTAAAATTGTTGCATTTGTGCATGATAAATCTAACATGGTAAAAATATTTCCACGTTTAGTGTTTTAAAATCTTTGTTTTAATCCTTTAACTCTTTTTTTTAGAACAAATACTCGATGGAAATCAAACATCCGACCATTTAAAATACAAGTCGTGTTTATACCACTAAACCAAACTCATCTGACTAGCAGGTATCAATTATCTTATTCATATAAACACTAACATTAATT

>Csa4M332110.1

TCTTTAATTTCTCTTTTAATTTCACAAAACTTTGAATCAAAATAAAAACACAATATTATTATGTATAAAGAGAGAGAGATCAAAAAGAAAAGAAAAAGGGGGAAAAAAAAGAAAATAGATTTTGGAATAATAAATATGGTTTAATTTATGCTAGATGATCCGAGGCTTCCCTTGATAGTTTTCATTAGAGAAAAATCCAAATAAGAACCAAAAAAGGTTTGAGCAATCAACACAAATTAGCTTAATCCAACTCATTTCTACTACCCCACGTGAAAATTTGTGGTAATATTTTGAAACCATTTGTAACTCTAAATTCAAATTCAATAAAAATATAAATAAAAACCCCAAAATCACAAACACACACAAACCAAATGATCAAAAGCTTAATTCTTTAGAAAAAGAAAAAAAAATCAAAGGTTTTGAAATAATTAGATCAAATAATTTGAAATTGAAGAAGAAATTAGAGAAAATAATCTGAATTTGAAGAATCTCTGGCTTCTCTTTGCTATATTCATTTGTGGGTTTCTGAGAATTCTCTTTCAAGAAAAAAGAAAAAAGGAAAGTTTTTATTTCTTTGTTTTGTGGGGTTTTTGAAAGAGAATAATTTGGGAAATGAAAGTTTGTTTTGGAAACCTGTTGAATTATGGAAGAAAAACGATGAAGAAGAAGAAGTAGAAGAAGTAGAAGAGGAAGAGAGGAAAGAGGAAAGAGGCGTTAATTTTTTAACGGAACGGATGACGTCTCTGTTTTTGACGGAGTCTATGCCTCGCTGGTGTTAGTTGACGGTGACGGGGTTGATATTTTAATCGGCAGGCTTGGCTTGGCTTGGCTATTTTTCGGCTTCTAACTTTTGGTAAATTAGAGGTTTAAATATATTTTAATACAAACCCTAAAATTTTAAACTTTTAATTAGCTATAGGCAAAGAATGAAATGAACCCATATTAAGATACCAAATATTAACACCCAAAGTCCATTGTGATTCTTTCAATTTGAAATTTAGTGATGATTAAAATAAAATTAGAAAATTAAATACATGGAGATAGATAGAAAGAAAACAAACCAATTGTAATTGAGTCGTTATGTAACGTAACTTAGATCTCATTTGATAACCCTCCCATTTTTTTAGATTTTAAAAATTAAATTTATATTTTAATTTCTCACGATGATTTAAGAGTTGAATTCTTAGCTAAATTTTAAAAGCTAAAAAAAGAGTATGTTTTTGTTAAAAATAAATATATGAATTGATTCTTAAGATTAAAAAGTAGATGATAAAGTAAAAAATTTTAAAATGAAATGAGGATAAAGGATATTTATAAACTTAAATTTTAAGAATTAAAATTAAAAAATCAAATTGTTGTTGAAGGAAAGTGAGTAATTATTATGGAAAAAGTAGAGAGTTTAAAACTGTGGTTGAGAATTTAAAAAAGAAAAAAAGCACTTGAAGAAGGCAGGCAGCAGGTGCTTCAATGGCATCTCAATATAAATTAAAGATTGAGCTTAGCCGATTAATTAAAAATTAAATTATGAATAAACAAAGAGAGTAATATTCATAAGATTTAAGCTGACTTTTTTAAAGGGCCAAAGAGTGAAGAAGGCCAATTTGACCTCTTTTTTTTCTCTTCATTTTTAAATCTATATTGCTAAATATTAACTTTTAAATATCTTTTTCTAAAAAAATTTGACCACCTTTTCAATTTTTTTTATTTCAATACTAATAATAATATAGAATGAGTGATTCCACATGTAACCAACTTAGGGATTGCACCACATACCTTGGAAATATTTCTTTTCATTTATATTATAAATAGTCACCACAACACATTATCTATCATTTAAGTTCGTCAATTAAATGTGTTTGTATGTATTTCAATAACTAAAATTTATGTATCTTTTATAGGTTTATGAAAAATCAATCATACAACATATAGTTTAAGTAAGCACAATAAGTATATACATTTCAACTAAAAGTATTAGAAGCTAACGCGTATATGTATACACA

>Csa4M646020.1

TTTCGACATCGATCATCAAGCCACAACACACAAAACTCCTTCTCTATCTATCTATCAATCTCTCGCTCTCTCTCTCTCTCTCTCGCTCTCTCTTTCTCTCTCTATCTCACTGGTCTCTTCATACACAAGGTTCACAAAAACGCCTTTTTTTTCCCCTCTTTTTCCTTCTATTTTCCTCTATTTCTATCTCTATTACGCTAAAAACCTACGCTCATCTTTAACTCTGAAAAACCACAGATTCGAATAAACATGGCTACCCTTTTCTTTCTTCTTCAAGCTAACACCCACTTCAATACACTTAAAACTCCTCACCAATTCCCTCCATTACAATCCTACACCTTCCACGCCCTTTCTTTCTCTTCTCTTCTCTTCTCAAGTCAATGACGCCATCTCGATCCTCTGCAATCACATACACCATTCTTCCATTTACCTCTCTTCATCCTGACCCTTCATTTCTCCCTCCAAGTCCACGCCTTTATGAGTACCCACATGAAATTTATCAATTGGGTCCCCAAATCTGTCGGTTAATCTTCCTCCCCACCGTCTGATCAACACAAATAACTCGAATTCATCGATGTAAAGAAGTAGAGAATTGAAAAGAAACAAAAAAGAAAAAAAGAAACAGTCAGTTTGGGGAATTCTTATTAGTTATTATTATTATTCAATTCGGAATCTTAAGAAAGTAAGCGAATCCAAGTGTTAGAAACATCATCATCATCATCATCATCTGAGGTTACATGATATGATTTAATTGATACATAAGTTTGCTAGATAGAAATGTACCAAATCAAGAAAAAAATCACACTTTTCTTTTCCTTCTTTTAAATAAAATTGTAAAGTAGTCACCAGCACCACCCTCCCTTTAACTTGAAATTCAAAATTTGCCTCTATTTTGACTTAAGGAATATTATATTCACAAAATGAGATGTTGTTATTATAATTATTGTTTACATTTATTTGAAAAATGTGGTTAAGCACTTATGAAATTAGAAAATAGTTTTGTTAGATAGAATTTGAGGCTGATGAAGCCACGTTTGTGTGGGAGGGAATTGGAAGAATAAAAAGAGTAGGGTGAAGCTGGCATTGCAAGGGATTTGCCAGCTCATTTCTTACGTGTCCATATACATTTTCCAACTTTGCCAAACCTCGGACCATATAAATTTAATAACCACATTTCAATTCTTTCCCTAACTTTAAATTACTTTCCTTTACCCTTAACCCAATCCTCTCCTCACAACAGTTTAACATTTTTAATTACTCCTCATATTTACCATATTTACAATAAACAAAAAAGATGTATCATGAACTGAAAATATTTAATGAGCAAAATTTTAAAAATATAGAAACTTGACGAGTTATTAACAATATTACTAAAATATAAAATTTATTATATTTGATTTTACATTTTTGTTTTAAGATTGGTTTCGATTTAGATCATAAAAACGTATCACTCCATATTGATTCTCGTAACAATTTAGCATATAGATATGAAAGATTTATCTATTTAACTTTTATACATGCTGGGTTTTAGTACTGGAAGGGTTGAAGTTAGTGCAATTAAAGTAATTTTTATTAATAATATATGATTAAATTTGTTTTCAACCGTAAGTTTAAACTTTTAAATAAATTAGTGGTTTAATAACAAGCAATTGTCAAAATTAGCAAATTTGACAAAATCTTTATAACACATAGCCAAATTTAAAATTTTAACAATAATAAATATTGATACATTGATTAACAAAATCTATCAATAGTATAATCCAAAGAAATTTATAACCAATAAATATTTTAATTTAAAATGTCCCTTTAATAATTATAAAGTGAAATTTTAATAGTAATAAAAATGGTAAATTTTAATTAATTGGAAGTAGATTGAAATAAACTTAAAACTTTCATTGAAACTAAATCCAATGGGACGAAAAAGATGTTTTTCCTAAATATTTAAAAGAAGAAGTCAATATCTGCACTCGTAATCTAATTAAATGGGTAATGAAGGGACA

>Csa5M148620.1

CGAAGGCAGAGTGAAAGTGAAAACGACGACCCATGAAGAATTTCATGAAGAATTGAAATGGGTAAGTGGAGTTTTGAGGAACAGAGTCGTAGGTGAAGTTGATTATGAATGGGAAAGGAGGATCGTCGGCCATTTAAGCAAACGAATCTGCATAACTCCTAAAAACAAAATCGAAAGAGGAAACCCTAAAAGGTGAAGAGAGTTGAAAATAAGCGAAATTGAAAAAGGAAGAGGAAAAGGAAAAAAAAAAAAAAGGGAATAGGATTCGTTGAATGAATCGTTGAAGTGGTGGTGGTGGTGGTGGTGATTGTGATTGTGTTCTTTCTGTCCCGATTGAATTGGAAGTGTACAATTGGAAAAAACACTGAATTTAGCTCCGCCACCCTCTCTTTTGCATTTCGATTAACTTCATTATCTGTTTTTTCCAATTTACCTTTTTTCTTTCTTTTCTTAAATAGTTATTATTTCTTTTATTTTTAAAATATTGTTCAAACACTCTAACTTTCTTACATAACATTGTTACAAAAAATAACGTTGAATTTTATTTTATATTGGTTTCTTTTTTCTCTTTCAAAAATTATTTTTCTTCTAAAAAATCAAATTATCATCCTTTTACCCAACCTACAAATATTTCAACTCAACATATTAAATTTATTAAGTATTATTTAATATATATTATATTTTTAAATTGAAAATGTGAGAGAGACACGTACCTCTGACACCTCCTTAAATTAGTCTTTGTTGGATTGACAAATGTAACGATACTATACAAAAAAAATAGTCAAATCTAAACGAAAGTTTCAAATCTAAAAATCTAAATATTAATCACATGATACTTTTAGTATTTTTCATTGAATATTTTTTATTTTGAGACTTTTTTTTCGATTTCGAATTTGTTGGGTGTAAATATTTTGTTATAATTTTTAAAAGACTTGGTAATAATATTTTTTAGATTTTCTTTCAAACATTTTCTTATATTTATAAATAATTTGATTCATTTTCTTAGATTTGAAAACAATGTTTAGTATCATTTCTTTTTTTACTTTTAGTTTTACAATAAACATATTGGATTCCTAGTTAGTTTATTGATATATGTAGGTAAAAAATTATAATCAATTTCAAGCATTTCCATTTTGCATTATAAGGACTAAATCATTAGAGATTTGGAAGCTAAAAAGATTTGATTATTACAAAGTTAAAAGTATATTATAAACTAAATCGTTAATTACACCAAAACCAAAAATGAAGAACTAGAATTTGTAAAGGGAAAGAGTAGGAAGAAGAGAAACTAGTAAAGATAAAAGGTAATTTTGATTTTCTAAGAATGAAGTTATTGATCTAAATCTTTTTAAAGATCATAAACATATATACATATTTTGTTTTTTTAAGAAACATGAAAGTTATTCTAACATTTTCAACACATGAAAACTCAAATACACACTAAGAATTAAATTGTCAATAATTAAGTTCCGTGGACTAATTATTTAAGATAATCAACTATTCATCAAATACACAGTAAGATGTTTTTGGAAACAAAAAACAAAATAAAATGAGCACTTTATGAGGAAAGTGATGGCCATGAGAAGCACAAAGAAGAAGAAGATAGGAATCTGCGTTTTATGTGCATAAATAATTATCCACTAATTCATTGTTTTTATTTTTTATTCTTTTGGAAAAAGTCTAACGAATTTTTAACCTAATTTTACCTATCAATATTGAATGAACCATTCTATAACATATATATATATATATGATATGATATCTCAAATAACAAGATTATTTAAGATAATCAACCATGCTATTTAAAAAACAATTATTCAAAATTTTAAATATATACAAGTACAATATTATAAAAAAAATATATTCAAAATATAATGTAAAACAGGAAAAAGTATGTTGAAAGTTTGGCCGATAAGACTTAATAATTTTCGATCTATTTCCAAGCGGAATTCATAAATTATTTTGCCATAACTTATATGTAATTTCGAAATCCAAATCTT

>Csa5M166980.1

AATCTTTTGTCCTCGAGAAAAAATAATTGGTCCAAAATGTCAATAAAAAAAATTTAATCACAGTGTGAATTGGTCTAATGGTAAAAAGAAGACATAATCTCGATAAAATAACTAAAAAGTGAAGAGAGTAATGTTAACAAAAATGATGAAATATCACTAAAATTCATTAGATTTCAATGGTAAGTAGAAGAAATGGTTCTTCACTATCCACAAAAATAATATATATATTTTAGACAATGTTTTGAAAATGTTATGTCATCTTTTTCAAATAGTAATCATTTTGCTAAATACATTAAANNNNNNNNNNNNNNNNNNNNNNNNNNNNNNNNNNNNNNNNNNNNNNNNNNNNNNNNNNNNNNNNNNNNNNNNNNNNNNNNNNNNNNNNNNNNNNNNNNNNNNNNNNNNNNNNNNNNNNNNNNNNNNNNNNNNNNNNNNNNNNNNNNNNNTAATTATTATTATTAAAAAAAAAAATCAAATTTCAGACATTTTCATGTCTCTTTGCTTAGACATACTCTCTGTCTGTCCCCTCTTCTCTCCTAATTTCTTTAATCTGTTCTTTCTCTCTCTCTCTATCTCTCTCTCTCTGTACCGATCTCTGCCAATCCCAGAATTCAAAAAACAAAATTGGTACAAGTTCATGTTAATCCCCTTTTTTTTTCTGTTTCTTATTCGATAATTTGGACTCCAATTTCGAGGGTTTTTGATTTTTCATACTCACTCTTCAATTTGATTGATTCCCTTTGGTTCTTCATCTGATTTGGGAGAAATTACGAAGTGGGTTCTGCGGGATCTGTGTTTTCGAACTTCTTTTCGTTGAGTAAGTTTCTTGTGATATATAAATATATATGTTTATATATATATTTTCTCTAATACATGTACACACATCTGTATCAGTTGATAATTACTCAGTTGTTTAAGCTTAGTTAGTGATTATTTATTTCGAGTTGAGTGTTTTTCTTCCTTGAAAATTTAGTAGATCTGCATTTTTAAGTAGTTTTTGTGTGTTTTGAGGTCGAAACTCGTTGATGGGTTCCGAGAATATGGGGGAATGGAACGGAAAATGGATAAGAGTGTGCTCGGTTTGTTATTCTGTAACTGTTGGAAGCTGGGTGAATGCTTGCGTTTAGGTTCAAGGGGGTGGGAGTGATGAATTGAATTATCAAAATTGGTTGCGTTGTTGAGATATCTCGATTTTAACTGGGTACAGTGTAGTTTTTTTGGGAGTGGACATGCTAATTAAATTGGGGATTTAGATTGATGTGTTTTGAAATTTGGGAATTATAGAAGGCAGACTCTTAACGATTCCGATTCTTTTATAGCATGTATGTAAACCAGGAACAAAGTTTTCAAGTTTTACGATTTGGGGCTTTGAAAGGTCAGCTGGGAAATTAAAGTCATGACTATGTGAATGACCTGGTCATCCACTGGGTTCAAGTGTTGGGCTTAAGCCATATTCCACCAAGCTTAAACAGAGCTAACTAGGTTTTTCTATAAAAAAAATAAAAAAACAGTTGACTGTGGCTTAGAACAAATGCTGCCACAGCTATCTGGTATAGGTTTTTTGTTTTTTGATGATAGAGCTTAGAATAGATATCCAAACCTCCCATCGTGCTATGGTCTGGTCATATCCATCTTTTCTGCAATTGTATATACGAACAACTGTGCTTGCTTGTTACTAAGGATGCTAATTTTATTTTATGACTTTATCTAAATTGTATTTTAATCATCTTAACTCATTTGATCAATTTAAAGTCAACCATTCTCCGCTGCCCTGCTTGTTTATCTTTGTTAGTATGCAGCTTATACTTTCTGCATAACGTGCTACTTGGATACTTATCTTTTAACTTCCCTTTTACTCCTCGTTAAGATCATTGTATGGTTTTTAATATTAGCTGTTTAGAAGTTAGTAACCGAACGACATGATGGTATTAACATTGTGGAGATGGGTTTCCTTTAGGAAATATTATGTGCTGATTCTGTATTCTTTAAAGTAAAAGAAA

>Csa5M385380.1

CGAGACTCAAGCCCATTAAAACATCCCACCGGCAACGGCAAGAAGAATTTCCCGATGAAATCAAGAGAATCCCACAATAAAATTACCAAATTGAAAAAGGGAAAAAGAGATGTTTAGCGAATGAATCAGCCTCCAAGTTTGAGGAAAGGAAGCTGGGAAGAGAGAGGGAGAGGGGGAGAGAGAGAGAGAAGAAAAGGGTTGAAAGATGGGAGTGAATTGGACTGGTAAACGCCGGAGAGAGAGAAAAACGCGGTGCTACGTGGAAGTAAAAGTAGCCGAAAGAACAGCCGAGCCGAGGGCGACGGCGATTGGCTGGGGGATTGGGCGATTGGCTGGCGAATTGGATTGGGAATTTGGGGGAAATGGAGGGACCTTCCCTTTTTGTTGTTTGCTTGTCAGATACCAAGTCCAGTATATTTCTTCTTCCTCCTCTCCATGACGTATTCACTCACTCACCCGCTTCGATCATTCCTACGTTCTTTCTTTCTTTCTTTCTTTCTTTTAATTTCCATTTCTAAATCGGTGAAGTGAAGTTGGATCAATAGGAAATAACACTACTTTCCTTTTCTCTTTTTAACCATCCTCTCTTATTTATCATTATCTTCCTCCCAATTTCAAACCCCACTTCCCCACTCCCTCCATACACTCACCCACACAAACTTTCCTCCTTGCTTCCTCACGTTTTCCACACAATAAATTACTTTTTAAATAATAATAACAATAATTTATGAAAATTTCAATTGATAACTCATAACAGAATTATTGTCTCACAACTAAACAAAAACTTTACCTAATCTTCTATAACAAAAACTAAAATCACCTGTTATTAATTATGTGGCAAAGCTAAGCTAGTGTGAAATTGGTACTGTCAAAGTTAAATGTTTGGTTTTCTTGAATAACATTTTGTACCACAAAATCATTGTTATGCATTTCATTTCAAAATTGTTCAAAATTCAATTTTCATGTTATTTTAACCAAACAAAAAAATGTCTTATTCTTTTAATCAATTTTGAAGCTAATTTTGAAAAATATAGTTACATAAGTCTCTTCTCTTCAAAGTTTGAGGAATATTTTGTATGATGTAACTTCAATTATTTGGGTCAGATAACATTGTCCTCATCTACTTTTTAAATCAGTAACAACTTGTTTCATTGCACTACATTATTGCAACATTAACTTGGTCTTTATAACAATTTTATTACAACTATCACATTGCAAAGGATCTTATTGATTATAATTATCCCACTAAGTAAAGATTAGGCCCGTTTCCAAACCATTTTTATTTTTCCTTTTATGTTTTTAATTCTATTTTATTTTTTTATTAAAAACAAAAATCACATAAAGTTTGGTAAGTAAATTTATTTTGTGTTTTTGAAAACAAAATGTATTTTTTAAAAGGATATTTTTAAAAATAGTAAAATATTTACACGTTATACCAAAATTTTAGATTATATTGATAATAAAAACTTATAGACTTTTATCATTGTTATTGATAGAAGCATATATGTGTCTCTCAATTATTGATAGAATATGAAATTTTTACTATATGTTGTAAATATTTTTAAAAAAATTAAAATGTTTTTTCTTTTCAAATTCCTTCGTAATTTTAAAGCAAATGTCTTGTTTAAAAAACAAAAAGAGCAAGTTTCTTGTTCATTAAAAAATAATTTTCAAAAATGAAATAAATAGGTTACCAAACACGCCCAAACTATTTCAAAATTTAGCTTGGGTTGGTAGCTCACATTTAGAAATTCCATTGCGATGCATCATGTAAAGATGGGAAGGGAGTTGCAAGCTTTGGAATCGGTTTATATAGAGTTAATGATGGTTGATATTACTTTATGATTTTTTGGAGAGTCTTTATTTGTCAATTTGCATATAATATTTTTGAAAATTTTCTGGAGGAGTACGATAAGTAAAAAGTGTTCAATCGTGAGAACTAGAGGTACGATGAATCATAGTTGAGTAATAGTGGAATTGAATATGTTGCAAACCAACCC

>Csa5M523010.1

TTCTTATACTAAAGAAGACTCTCTTTAAGACATAATAATAAGAAAAAAAGAAAAAGTTTTTGAAGCAAAGTTAGATTAGAACTTCAGAGAGAAAATTAGTTGAATTAAACAGCAAGCTAATGAAGTTAAAGAACTATGAAAATATATGCATGATTCCATCCCTGTAATTTTTAGCGGACCAGAATCCAAAACTCATTTCAGTTCAATAGATACTGTAAAAGAAAATCAAGGACTCGGAATTCTAGAAGTTACGCCATTGGCAGGGATAATAAATGAAGGGAAAAAGGAAGAAGAAGGGAAAGGAAAAAGCTAACGAAAACACAAACGAACGACTTTTAAAGGATAGTTAGCAAAATAACAAAGTTCAGTTGGAAGAAAGCTGAAAGATCCAATTCAGCCTGGAAGTTAACCCCCAAATAATTGTTCATTCAAGTATGAAACATGTAAAACTTGGTATCAGAAAAGGAAAAGCAGCGGAAGGAACTGGGTCTGCCTCATAAAACAGTAAGAAACAACTAAAAATGTTGAAAGGAAAGGGAGAAAGACAGAGAGGGAAACAGAGCAACGGATACGTGGGGAAGAAAATCTGACGGTCTGAAAAGAAATGAAACCATACTCGACGATAATTCTAATCAATCCGATCGAATTCTTAAAGAATTACAAAGTGGGACCATAAAGAATCATCTTCAAAACGCACCCAAAAGAAAGAAAATGCTTTGGGTAGCTTTCACTACACGGATTTATTACCTCAGAACTCAGAAGCACCTCCGCCTGCAGGGGAGTTAGTTCAAACATTTTCACAAACAGTTGGAGTGCGTCAAAAGAAAGTAAAAGGGTTTGGCCACCAGGTGTTTGACTTTTCAATCAAAATAAAACCATTTAGAAAAACACCCAATTGAGAACCCATTTCAACCATTGGTAGGTTCAGATTCAACTTCCCAGACAAGTACAAATGAGAAGAAGAAGAATGAAGAGCAAGGAAATAAAGAGGGATTAAACGTAAGGTCATTGGGTGAAAGAGAAAGTTGGGCATGCTTGACAAATGGGCAAGAGAGATCATTCATGAAATCCAATTGAATGAAGTGAGAAATGGAACTCTATTGTCAAAGACTCAAAACGAGAATTCCTCAAACAAAATTTGGTCAAATTTGTAGGTTCTTCTTTGAAAATGTATGAGTGGAAAACGGTCAAAATCCAAATTGTGGTAGGTAAATATTGTTTTTTCTTTTGGAACAATTGGAACTCAAAATTTCAAACCACCGACCTGTAAGTTATAAGTATAACAAAAATATGTCGGTCTACATAGTTTAACATAGTTTTCAACAATTTTGCTATATTTAAAACGATCTTAAAAATATTGATAAAACTTCATGAAGAAATTACTTTAGAAAAGTTTGCCATTGTAATGACGCTTTTAATGGTAAAAACTAGATTCTCAAACTTTTACAAAAGTTAAGATTGAACCCTTAAATCTACGATAGTGATAGAGAATACACTCAAATCATAAAATTTTGAAGCCTCAAATTTATACATTTGTTTCAATTTTTATAATTATGTAGGTTCGAGAATCCAATTTCAACACTTTATAAGTTCAAAATGCTGAAAAGTGAACGTTAAAAGAGCATAATTGCAACTAACATCATATTTTAGAGTCAAAATTTAACTGTGAAAATCATAAAATTCAAGAAATATTTAAATCGATAAATTAAATTAATATGTTACGTTTATAAAATCAACAAATTTATTTTGTTTATTCTATTATATTATATTGACATTAACAACCATTCTTAGGATGATATCAAATTCACAGTTCAGGTCGATATCAAATATAGAAAAAATGTGTAAATATAGATGAAAAATTAATAAGGTGATTAAGACAATGGCGTTTGGTTTGGTTTTGTTTAAAGGGAATTGAAAAGAGATGATATAAAAGGGTGGAAAAAGGTGGCAGAAGATGAGAGAAAATGAGGGGATGCATTCTACATTGATTTTGGATTAGA

>Csa6M058190.1

TTTGGAGGCAGTCACACACAGACTACCAAACGTAACAATGGTGGACGGTCCCATGGCAGTGGGCAACTTTGACGATGGGCTGAATGAAGATAAAGGTCATCTCAATGTTAAATCTGAAGTCAAATTAGGCAAAATGTTTGCTCATTCCTTTTATTCAAACTTTGCCCACAACTTCCTTTCCTAATTAATTATGTACCAATGAAATGCTCACACTAATTCAATCAATTCATAATGCTACTCTAAAACAATATCTCTTTTAATCATTGTATTTGGTGGAGTGAATTTATTTCATCCTTCCAACAGAAATGATGTGGATGCGGATGTATATCTGACTCGACTTAAAGGTGAAAGTTTGATTTTTCATTTATGTAATCATAAAAAGTTAATTTTAATATTAAAATTTTATATTCTTATATTTGTAGCATGCAGTGAAATTTAATCTATACCAACATATACCAATATGATATAAATGGATATATGTTGTAGTTAAGTTAGCAATTGGGGTTTTTTCAACATTTTTATGGCTTAGTGTATAGTTTTGTTGGTTGAAACTGGTATGTGCAAGTTTATATTGGTTATCTATATTTTAGAAAATTGAAAGTTTAGTGCAATGCCTGGATACACACCTAAATTAAATTTGTAAAGACCAATTAATATTTCAAAGTTTGACCTATATTATTTGATACACAATTAAAATTTCTAGAACTTATTTAGCACATTTCATTTTACTAAATGAATTTTATAATTCAAAAAAAAAAAAAAAAATCAAATGTGAAAAATCACAATAACTAGAGTAATATTTGGTTTTTATAATAATTTTGGAATAAAGTTAACAACGGGAGTTGAGTCAATAAAATATTGATAAATGGGCAAGCAACAATGCCTAGAGATGAAATAAGCTTGGAGAGAATGATTGACTTGATCCAAGATGAAGTTACCATTGATCCCACCCTCTACTAAAATCTAGACCTAATCTACCATATCATGTAACTCATTCATGATTCGACTCAACAAAAATTTAGAAGAGATCTTTAACTCGGCCCAAAGCCAAATTAATCCACTCCACTTCGATGAGTCCACCAAATCTTGTTCTCATGTGATTATAATCCAACAACAAAAAAAAAAAAAGGATGGATGTGTGAACATCAATAAAATCTAACTGGAGAAAATATAGGTGATCCAACCTTGTAAAAATGCAAGTAAAATGACTTAAGAGATAAGTTGAGTCCACGGGAGATCGATAACAAAATTTATCTTTAGACTTTCTAATTGAAACATCAAAGTGTACGTGTAAGTTAGTTGGTTGTTTGTATATATTGTTATTCACATATCCACCTTATGCATGTATGTTAGGTACAATTAGCATAATCACATGTGGAAAAAAACCATATTATTTTGAAAAAGAATCCATCACATGTTAAAATGTGATGATGTGATTAGAGGAGAGAATTGAAAAAGTTGAGAGTGGAAAAACAAATAATATTAATTGTTATTCTTAAAAGAAACAATATTAATATTTATCTAAAAAGAAAAGAAACAGCAATCATTATTACTTTCTAGGATTACAGCTAAAATGAAAGAGGGAGTTGGAATTAAAAAAATTGATATCGTAATTAAAAGGAAAGGGAAGGGTATTTACGTAAATTGAATAAACATTCTCCTTTTCACACCTCAGGCCTTTCCGGACCCTATCCATTTCCATTATGGAAGCCTTTAAATATTAAATTCCAAAAATAGAAATATTTTATTAAAAAAACTTCATCTGAAATTCGTTAATTTGGTTTCGCCCTTAAATTTCAAACCTTAATGACCACATTACCCTCTATCTTCACCCCAAATACCCAACCACTCCATCTTCTTCCTCAAGATTCTATCAACAGTCAAAGATCCTCTCTACTGATCAATAAATATTCCTAATCTCATTTCTCTACTTCCTTTCTCCTCCAATTTTCCCTCTCTCGCTCTCTTTCCCATGGCCTTCCCTCGTCTTCCTTGATTCC

>Csa6M110320.1

ATCAACACTACAATATAAAATATAAAAAGAAATAAACGACGTAGTTTTCTTATTCCACTTTTCATTTCTTTTTGGATAATTATTCAAGATTAATTAATATTCAATTTTTTTCTTTTTTCTTTTACGTCTTAAACTTGTCCATTTTTTAAACTTTATTATTATACTATTTTAGATCTTCGAATTTTGAATTGCAATTCATTTCTTTTCTTAAATATGTATTTCTTATTTATTAGGTTTATACGTGGAAAATGCACTCTAACCATATGTATCCTACTTTTTATTATTATTATCAAAAAGTTAGTAGAATAGATTTAATTTCAAGTAATTTAAAAAGTGAGTTAAAAGTGTTTTGCATGTAATTCATATCTAGACATGATCCAATATTCATGCAAAAAAGAAAACAAACAAACATAAGGATTCCAAAATATTTTAATTAAAACAAAATGTCTTTTTGAAGCTTTTTGGTGCATAGAATCATTAGTTTGACTAAGAAGATAAAATCTATATATTCAAAATAGTAAGTACTTATTTGTATAACCATAACGTGAGAATTATGTAGTGCTTTCTAGTAAACCTTTTATGTCCTTTATAATGTCATAAACAAAAGTATTTTAAAGAAAAAACTACAAACAATTGAATGAATTTGATATAGTTTATTTGACCTTTATTTTTTTCTTTTTTTCTTTTTAACTTAAATAAATATGAAAATCATGCCAGAAAACAAAACATATTTCTAAAAAAAAAACTAAAAGAAAATTAAAAATCAAATGGTCGTCTTTATTTATAACTTTTAATTTTTAATTTTTAATTTAATTNNNNNNNNNNNNNNNNNNNNNNNNNNNNNNNNNNNNNNNNNNNNNNNNNNNNNNNNNNNNNNNNNNNNNNNNNNNNNNNNNNNNNNNNNNNNNNNNNNNNNNNNNNNNNNNNNNNNNNNNNNNNNNNNNNNTTTTTAAAATTATTCTTAGTTTTTCACAATTTCTTATTTGTGATTTTCATCTTTTATAATGTATATGTACAGATTTGAAGTTTTAGTCCATTTCTAAAAAGAAAGATAAGTTTCTTAAAACTACTTTCTTTTAACCATATATGTTTCACAATTATTTTTTTAAAATTAAAACGTATTTCTTTTTTATTTTACAATGATCTACATATTTACATATTTCTTAATCAAGTAAAATGATTTTTGTGAAGACCCACAGATCAAACCACTGACAAAGCTAAGCTAATTCATTCTCTCTCTCACTGTGAGAAATTCTTTTTCTGTTGTTAATTGATATAACTTTTCAAAAGCTTTTTTTTTATTTATTTATTATGGGTAGAAATTAGATATCAAAAGAGAAAAATTGAAGGTAGAAGTATATCTATAGACATGATTTTAAAAACTAAAACAAAAAGATAAAAGATTTTTGTTTTGTATTTCAAAACATTCTTAAAATGTATAGAATAACACAAAACATAAAAACCAATAGACATGAACAACATTTATGAGCTTGATTTGAAAAGTTGAAAAAGAATATCAAATGGAACCTTAGTTAAAATGTTTGACTTAATTTGATAGTGCATGCATATTGGTAGACAATGAATACGAAAACAAATAAATTTAAACATAGGAGTAATATTCACGGTTTATTTATTTATTATTTTCCTTTGTTTACAATATTATTATTATTGATCAAACATTTGATTTATATGTATAGAGGGGGAAAAAACCGTGGGAATTAAAAGAAAAAAGAAGTAAAAATGAAAAAGATTCACATGTAGAGAGGGAGAGTATTTCATGGACTGCTCGTGGCCATGAAATTGAGACACTGAATCTTTGGGTATCTTGTTCATGAGAAAGCTAAGAACATATCGGCTTTAGGCACCTTCCTCTTTAAGCCATATGCCTTTTACTTCTTTACCCTCTTCCTTCAAACTCTTTGTGATGACCACTTAAATATACATTCACATGATCTTCCTCTCCTCTGTTCAGTTATGCAAGATCTTTACCTTAACTCTGC

>Csa6M136540.1

ACCATCATGTTTTTAAACTTTAAAGTTAACTCGGCCAAACTTGAACTAGCTCCACCAGTCAAAAGACAACAAGGAAAGCTGCTTGAAAATCAGATTGCTAGCTCTACAATAGCAGGAAAGGGAATATATATTAAAAAAAAAAAGACCATAAGAGACAGTACAAAGTAACTAAATTAGCTGAAGCATTTCGACTTGCAAACAATTGAAATTTCCAGATATGATCTGATACATATAAGTATCTAGAAGAAATATATGTGTGCTTTAAATTGTTTGCTATATCCCAGAAGATAGTTGGCTGTGAAAGAAGACTTACATAAACCATCAAAACTACTATTCATCACTAAAGTAACCTGATAGCTCCAGCCAGAGAAGTATAAACACAGACACGAAGATAAAATAGCACAGATAACATGACACATGAAATACATATGATTGGTTAGTTTTAACATTTTTTTAGTTTACGATCTTTTCATAAGAATGAAAATTTAAAAGTGAAGTACAGGTGTGAGTAATATTTCAAGTGATCTAAGAGACTAGTTTCTTATTTAGAAATGTCTTACAATACCTGAGAAACGTCTAAAATCAGTTTAAAATTCTTCCAAATTATTTAGCATCTTATAAAATAGAAAATTTTGAGTCAATCATGGTCGTGTATTTGAATACTGAAAAAGGAAAGTTTAAGTATACTTGCACCAATATTCCAATATAGATCAATAATCAAGCACTTAAGTGTTTGACGCATGTCCGATAAGTGGAGAGGAATTTGAGTGTATCTGGCAGCGTCAGTGATAAACATGCTACCAAGCTTAAGTTCTTGTGATTCATAGACTCAAAACAATCAATTTTCTAAAACTAGTTGATCCACACAGAAATTCATAAATACTCTACATTCTTTATTTCCTTCATCTGATTAGAGTCAAACAAAGACTTACGATCTGTACAATCAAAGCCAAGTCAATCCATTTGAAACAAACTACGATACTCTAGTTTTGAGTAATCAAAACAAAACAACTAGACAACGAAGAGGATGGATAATAAGGATCCATCCACCATCTCAATACGTTCCCCCAAACTGTCAGTATACTTTACGATCACAAACACTCCGTTAGGTTTACAATAGAATTCAGATTCATCAAACCGAAAACTCAATTACAGAACAAGACACACACTAAACGAACATAAAATTTAACAGCAATGAAAGTAGTATCACACTAATAAACATCAGATCAAGCCAAAATCTCCAAAAGTTAAATCAGATCCACAAAAAAGAATAAAAACATAACGAATCGGAATACCTGATGAATTTGCAGAGTGACACCAGGGAAGAAACACCATATCTCAAAGCTCCCGAGAAGGCTTAAGTTAGTACTAAACAATAGTACTACCTCCACGAGAAGCTCGACAAACGAAATTTTCCTTGGATTTCTCTCAGTTCACTCACTCTTCCCAACACTTTCAGCCTTTGATTCGAGAAAAGAAAAAAAAAAAAAAAAACTAAAAGAAAAAACAGAAAAACGAAATTAAAAGACCTTTTATCTTCAGATTTCACGAACAGATTTCATTGAAGCTTCGTTTTGGGTTAGATTATATGCAATCGGAAGTTCGATCTTGGTTGAGGTTTGTATAAGTCGAAATTTGGTATGGTTTCTTGTTTTGAATGAGCGAATTGTTCACCAACAGTCAAACGAGAAGAAGGTAACCAAGACCATAAAAACTAACTTCCATAAATACCCCTACTAAACCGCATTCTTACACAAAAATGCCATTCTCATTTCAAACTGTTTTCCGCGTTGACTATAATTTACATTTTCCACTCTAAGCAAACTGCCCTCTTCCCTCTTCCCTTCTTCTTTGCAATTTTATCTTCGCCACCTGGCCTTATTACAACCCAACCATTGCTCTACAAACATCGGAGTGGGAACCACCAATGCTACCGTAGCATTTCTATCCTTCTTTTTCTTTATTTATTTATACCGTCTAATTTTGAAATATATTTTAAA

>Csa6M154510.1

GCCTTAAAATGTTTAGAATATAAAACCATTTCACCAAGTAGTTTCTATTTCTACCCCCATATAAGGTGCAATCGAACTTACATACTCCTCAGTCAACAAAAACCACTGAAAAAACCCTTTAAGGTTTCCTCAATATCTTCAAAGCTGGATGGCATAATACCCAAGAAACAGGAACGTCAAAATGATTGGGCATAAGCACTAATAATGAGAACAAACAAAAAGCGAAAAAAGAAAAGAAAAAAAAACGAAAGAAAGGACATTAACAAAGTCATGCTTACACAAGGAAACAAAGCAGAAATCAGAGACCCCAGTAAACAAAAACAAAAAAGAAGACCCACAAATACAATAAAAGCTAAATCATCATCATCATTATCATAATCAAGATCAAAATATGACATCCCATGTACAAAAACAGCGATAACCCAGAGAAAAAGAAAAAAGCGGTTTGAGAGAATGCGCAAAAGAGAGGAATTAAAGAGAAATTACAGACCTGAAGAAGTATGAAAAGGGAGGAAGAATGAGGAGAGAAGTAAAGTAGGGGAATAATGGGGGAAGGGGGATCGGAGGGAATTGAGAATGAATCTTGAAGAAGATGAGGATTTTGATTTGATAGAAAGCGATGGAGTGAGATTGAGAATCACGTGCAATGGAAATGGAAATGGAAATGGAAGAAGGAAGAAGGAAGAAAGAAGGAAGAAAGAAGAAGAAAGAAGAAAGGTAAAGAAGGGTTTGGGTTCTGAATTTTTTCTCCGGGACGCCTCTCTCCGACAGCCAGCTGGCGAACGGGGTGGCTTACCAACATCCACACCTTCCTCCGCGTGAACCCTTTTAATTCCCTCCTCCCCCTCTTCTCATTTCCTTTTTTTATAAATATCATTTTTACTTCTAAACTTTTCTCAATTCAAAATATTAATTTACCCTCTACTCTAACTTTCATCATACTAATCTACATGTCTTTGGTTTCACATCTTTTTTAGACTTACACTTTAATGGGTAATTATTGTATGTCAAAGGTTCCCAATTATAAAAAAAAAAAAATATAGATTTAGTTGTTTTTATTTTACTCCCTATATTTTGTATTTTGTAAAAATATAAATCTCTAATTACTTTATTCTCTAAATTATTACAAATAAAAGTTTAAGAGTTAAATTGTTACTTCTTGAAATTTAGGCTCTTGATGGATTCTATTATAAGTTTGTAAATCGTCAATCGATCCAAAAGCTTAAGTGAGTGAATGTGAATTTAATTATATATCATCTAACAACCAGCTTCACTTCTAAATTTGGAAGGAAATAACATTGCAAAGGATTAAAGATAGGGCATTATGAACCATACCATCCTGATATCATCTTAAATTGTCAATTGATCTAAGAGCAGTGTGGTTTGTGATGTCACGTCTTCAAGCCATTGCAACGTTGTTTACTCTCCATTTAATATTGGATTTTCCCTTGCTAGGAAAGGTCTTCAATAAAGGTGAATATAATGATAATATAATTAAAATTGGTAGTCTCTAAAACTTTTGTTATTATGTTAAATAAATATAGGACTTATGTGACAAGTTACTAAACTTTTAATTTTGTGTTTAATAGTTTTAAGGACCTCCATTTGTTAGATCTATTTGGTACACGTTTTTGTATATTCTTTTATATTTGGTACACGTTCTCGTAGATTATTTTATATTTGGTACACAATCTTGTATATTTGGTATACATGATTGTGTACCAAAATCTAAACAATTGTGTACCAATATTGTAACGATTTTTGTTCAAGATCTTTTATATTTGGAACATGAACTTGTTCCAATATCGCTTAGATTTGGTATACGATCATGTACCCAAATCTAAATGGTCGCGTGTCAATATTCCAACGTTTTTTTGCTTAAGATTGTGTGCTAAGATCTTTCATATTTGGTACACGATCTTCTACCAATATCTTAAATTTGGTACACACTCGTGTACCCAAATCTAAACAATCGTGTACCAATATTCCAAAGATTTTTA

>Csa6M212860.1

TTAAAATTTTATATTAATATGGTTTCATATTTTTTTATAAATATAATTGTGTTGTAAGTTGAAATTAAAAATGAATGAATGGCATTAGATTTTGAAAATGTTTCTAAATAGTAAATAGAAAAAAAAAAACATTTAAAGTGTAACAGTGTGTTAATAAGCCAGAATTAAAAAGGAAATTGAAATCAGACAAAGCTTTAGCTTCTATAGTTAGAGTTTTTGAATTATTTTAGTTTCTAAGTTTATACAAATTTTAAGTTTATTATTTATTTATTTATTTTGAGGAAAAAAAATGTCAGTTGGTGGTTTAATCAAGCCCTTGGTTTATATGGTAACAAAATGCTATTTTCTACTTGGACAATTGTATTAGTTTATTGTGTAATGATTTAATGAAAAGAAATTATTTAAATTTTGATATATAAGAATTTAGTTATTTATTGTAACAATTTAGTTTCAACCAAAATAAAATTTATAAAGTTGGATATCAATTTTTAGTAATTTATGATGTAGACACTGAATTTCATAAAAGCATGGAGTGTCTATTTGGTATGTAACTTTTGATCCATATTTGAAAAGAATTGGTGGAGTAAAAGGTTGATAAGACTTGGAAAAATATTGAGAATTGAAATAATATTAAAGTAGAGGCTCCAACGTTGGGCTTCTGCTACTTCTTCACACACTCATTCCACGTCCCATTTCCAACATAGTTTTAATATATGTATACATACATACATATAATTTAATTTACAGAATTTCAAAATTCTGTAAACCAAATTATCACAAATTTATTGGTTGCTCCTACGATACTTTAGTTGTGCTTACTTACATACTTTTGGTAAAAAAACATTTAAAAAGATTAACTTAACAATGGCATGTCAGTTGTAAAAAAAAAAAGAGTGAAAAGAGATCAATGGACAACATGCAATATGGTAAGGTAAGGATTGAATCCGTTGCTTGTGACTTAAGTGTGAGATTATGGTTAATTACTAAGAAGAAAACATTATTTTTATGGGATTTGTGACAGTTATCAAAGTCATTGCCATTATTTGAATGTGTTTCTTATGTCAAAACTAATTCAGTAATCATCTGAATGTAACTATGTGACACAGTTAAAAGAAGATTTGAACATTCAACAATCTTGGTGAGATTCGCGGATGGGTGGATTCGTTCTTTTGTTCCAAGTTGATTCGTCCTTGAAAGAAATTGTTTTAAAAGTAATAATAGTTTTATGGGTCCCCCCATTGCGTCTTGTTCACTGGCGGTGTCATTCCGTTTCATCTCCGTCTTCATCTCTAATATCTCTGTCTCTATATTACCACGACTCTCAATAAAATAAAATAAAACTCCGAATTTTGAATAATTCCCCACAATTTGTTGTTTGATCTGATTTTTGTTTTCCTGTTTCCGTTTCAAAATTCTCCATCTCTGTTTTTCGTCTCCTCTGCTCTTTCATCTTCTTCTTCTTCATTTCTTTCTCATTTTCTCAGCCCGCAGGTACTTTCATCCAAACATGGCCAGAATTCTTCAAGTTCCCTAGTGCTTTTCTTTAAATACAGAAACACCCAACAAACAGTTCCCAATTATTCCATCTGGGTTCTCTTTAATTTCTCATTTTGATCTTTCTTTTCCATCTTAATTTCATCTTTCTCACTTGCCAAACACAACCCACCCAAACCATAATACTGTTGTTGGGTTTTTATACATATTACAGAACTGGAACCTTTCGACCTAAATGGTTTTGAAGATTGGAAACTTCGAACGCTCCAGTACGGCGGTTGGGATGGGATGTATTATGCTTAGAATTGATTATTCAAGGCTTTTTTGGGTTTTATTCCGCCACTTTTCTTACGAGAATCACGACGGAAAGCCGGGCATTTAGTTCATCCTTCCGCCGCCTTCGCCGCCACCGCTGCCCTCGATTTGATTATCGCCTTTTTTTCTTTCTTTTTTTCGTTTTCCGTTTTTTCCTTCTTTCTATCAAACCCTCTGTTTGTTCGTCCATT

>Csa6M330990.1

AGGGTCGCGAGTTCTACTCTCTCTCTGAAGAGGAGAAGAATGTCGAAACGAGGAAGAGCCTCTTTTCCAGGCTGCAGCAGCGCCGTCTAAACCTCCGGTAGTGCCACCTCTTCTACGAGTGGCAGCAAATCTAGAACGTTGAGGTGATATGGATTGAGAACGTTGATGGTGATGGTGGTGATGGTGGATTTGGTGGTCGGAGTTGGAATCGGCAGCAGTTCCTGAGAGAAGAAGTTTGGACATCTCTTTCTTGAGATGCGCTTGACCCTCCTCCAGCTCTTGGACCTTCCTCAGAAGCTCCTCTGCTGGTGGAGTTCCCATAAAAACCAACAACAACGGATCAATATTCAACACTTCCACTGCCGCAATCTTTAAAAACAAAAACCAAAAGAAAAAAAGAAACAAAATACACACACACACACACACCCAAAAGAAGAAGAAGAAGAAAAAAAAACAATGCTCTGTTTTCCAATCTAACTCTCTGCTCTCAAACTCTTTCCCCTCTCTTCTCCTTCCTTCAATCTACCTCTGATCTTTCAAATCTCTTCCCTCTTCAAAAATGACCATCAAATCAATACACCCAGAGATGAAAGAAAGATTTCAATCTGTACCAAAAAAAAAAAAAAAGAGAAAAAAGAGCATCAAAATCAAGAAATTCCGTTGAACTCAGAGATAGATAGATAGATAGATCGTAATCTTAAAAGAAAAAAAAGAAAAGAACCAACCAACCGAATTGAATAAAACGTAATCCAATCGAAACCCAGTTGTTGTTGAGCAGAGTCAATCGGTTGAGACAGAAATTTTTGATAGAGAATGAAAGGGTTTTTCGTCTTGATGGGTCAAGAACCTGCTCACCCTCTTACTTTGAGTCTTCCTTCCTACAGACCTCCGCCTTTCTTTCTTTTTTTTTTTTAATAAACTTAATTTCATTTTACTCCAAATTTAATATAGAAATAGAAATAGAAATAGAAATAGAAATATATATATTGTTTTTTTTGTATTGAGGTAGGTTTTAATAAATGTTGTAGTCTTTTTTCTTCTTTTCTTTTTCTTTTTTTTTTCTCAAACTCTTATTCGTTCAATTTGGGCGGTTATGTTTTTCTATCTTATCATTATCATCTGAATCTATATATATATAAAAGAAAAAGAAAAAGAAAAAGAAAAGAGGTTAATTATAAGAAGAAGAAGAAGAAGAAGAAAAGGAAAAATGTTAAATAAGAGAGAAAAAGAGGAGAATTGGGAGTGTAGGTATCGTGGCCAATCACACACCTCCAATTTATTTTTATTACGCCCTCTAACTTCTCTGCTCCCAATTTCCATCCAAATCTTCTTCTTTTTCTTTCTTTTTAACTCTCTCTCTCAAAAATTATATATATTAAATGTCCCTTTAATTGTGGGCGACTTCCACACTTTTTTTTTTTTTTTTGTTTTTCAACCACTATACCAAAAAGAAAATGAGTGTATTAGTAATCAATAAAATCGTTCTGCCACCATATTTTGATTCACGAAACATGTTTTCAATCCTACAAAATTAAATAGTTGTAGTTGTATTAAACTCAGTTTTATGATATATAAAATTAATTTTGTAAAATTTATATTTAAAATTATAAAATTAACTTTTAACGAAAGTCTTTGAAAATAATTTTTGTGTATAATAACAAAGATGGATTTTACCAAACATATACTAGATATTATAGACTAGGATGTACCCTTTTCTTTTCTAAATTACATTTTAGTTTTTCTTTTTAATTATAAAATCCGTTCTATTTTAGTATTTTAAATGTTAGATTTTAATTTCTATCATAAATCTTAAACTTAGTCCAAGTTTATAATTAATTTTTTATTTTATTCTTTTCAAAAAATAATTTATTATATGTCTTATCATTCACAAATATATATATAACATAGAAGTTTATGTTAAAGTATTTTTATTTTATTTGGGATGTGATTTTATGAGTCAAAAATCCTTTTGTTTTAAATTGCTTTTGGTTCATCCATTG

>Csa6M425140.1

CGTAGTGAAAGACTCTCAGATCGCTAGAAGTTGATGGAATTTGGGGGAAATGAGTGTTTAGGGTTCTATGAGACCGAATCTAACTGTGCCAAAGTGGGAATTTGAATGGAGATAGCAACGGTCGAATTCCTATTTTTCAAAATTAAATAATTTGACGATTTTGATATCTTTGAGATGGAGGACTAATATTTTAGCGTTTTTGCATATCTGACCCTCTACTTTTCCTATTTTATATTTGTTTCGTCCTCTAAGGCTTCTTTGCTCAAAATTCTTCATTTCATTTATTTTTTTGTTATGAAATGTGGTATAAGAGGCTCAAGAGTCCAAACAAATATTTGGACCAATAAAATAAATTTACGATCCGATCAAAATTAATGAATCCAAACAGGTACGATCTTAAAAGAGCACGTTGAGAATGTCATGTTAGAAAAACCAAGATGATTCAAACACTTTAATGAAATTATCAAAACTTAATTTAATTTTTGAAAAAAATGGTAGAATTAAATTTTATGAAAATTAATTAATAGTAAAAAAAAAAAAATTTGAATCTCCGTCAAGACTAATTATCAATGTAATGCTAATTTTGACATTCCTTTGATTATCACTTAAACCAAAAAATTTAAATTGTAATTTAATATCTTTATTTGTTTAACATATTTTTTAATTTTAAAATTCATACTATAGTTATCTAGAGACTTTAAATACCAATATATTGCTTAGACTTAGTAAGAGCTTTTATATCTTCTGAGAAAAATATAAATTGAGATATTAATAATAATTATATTTGGTTATATCAAGATTAGATACATTTTTTAAAATAATTAAATAAATTAAAATATTTATAAGTTACAGTAAAAAAAATTATTAGATCAATAATAGCATGTTTGTTTTTGAAATTTTGATTGGTAAATATTTTTTCGAATTTACCTTTTTTTTTATCAGTTTTCTTAAAATTATCGATTTAAAAAATATATAAAATAAAAGATAAAAATATGTTAGATAAAAGAATAAAAGAAATTAAAATATCATAATAATAATTAGAAGAAAAAAAAATCAAAATATCGACGGAAGAGGAAGTACCGAAGGAAAGTTAACATCTTTAGATCTCAGTTTTGTCTTCTCAATCCCAATTTAACGATGATTTTCTTCAAAATTCACAGGTGAAATATAAATCCCGCACCCAAATCCAAAATGGGATTTCACTTTCGTTTTTACTGAGTTTCATAAATCCACGTTTCCGACTCCCAAGAAGATACGTATTCAACTGTTCCAGACCCATATACATTCCTTTTGTGTCACTTTCATTCGCTTATTGAGCCTGCACAATCGGATTATCATACTAGCTTCCTTCAATTCGTTTCATGATTTAGCTTCACTCAACCTCCGTTGCCGATTTGGGTGGGAATTGGGATGGGGTTTGATTTGGAGTCTTTGTCGGAGGCAACATCTGGAGCAATTGGATCTCTGGTGAGTACCACCGTTTTATATCCTCTTGATACTTGCAAGACTAAGTATCAAGCTGAGAATCAGTCTCAACATCAGCGGAAGTACAGGTTTGTTGATCTCAATTTCGTTCTTGCGCTTTAAGCAACTGTTTGACATTTCCCGAATTGTTAATTAGGTTGGACAGAATCTATTCTGTATTATTGGTTTGGATGAGGAAGTTCAATTTTTATATTCATTATCATAATTGGTGCTTTATGGAATTAGGGCGTTTGGTTAGTTAAATTCTATTGTCTTTGTCCTGGATAAACTTGTAGGGAAATAGAACATCTCTAGCTTATTCCAGGTTTAGATGGCAACTCTGCTGCATGTGGCGCTCTGAGTTACAATGCTAGACTCTCACTATTTTGTTTTTTTTTTTTCCCACTTGAGAATTTTCTTTGAGTTCTACATTAAATGTGGGGTTGGTAGAGGTCTAAATGAGTTGAGCTAAATTTAGTCACTAGATAATTCGAAAGGTTCAAACACTAATTTCTAGGAATCTTCACTCATATAAA

>Csa6M450400.1

CTTATTTTGAAATGAAATGGGTACAAAACATGAAACGGCGACGTAAAAAGGCAGTATGGGCTAGGGAATGAGATGCAATGTTTTGATGACGGTCTACTTTGAATTACACACAAACAACAAATTCAAATTTTCATAAAAAAGAAAAAGAAAAAGTGGGAAATCGGCTCCAAAATTGCAGCCTTTCCCCTGTTTCGGCGGTGGCACCGCCGACCACCTCAGCCACGGCGTCAGAGCAGAAGAAAAAGAAAATAATTTTATGTACCGTTTACAGCAAGCAATCGGCGGCGACAAGTTCCCATGTAACCAAAACCCCTAACCATTCGCCGCCATGGTGTGAACTCTTTATTCCTTTTGAGTTAGAACATAAATTATAGTAAATTGATTATAAAGTTGTTGATAATATTTACAAAATTATATCAAAATTTCAAATTTATAATACTCCATCTAAAATTTTACTTTATTTTATTAATATTTTAGTTCTATTTAAAAATACTAAAGTTTATGAACTAAATCTAATGTAACCATTTTATTTTCACACTTACTTGTTTAGTTTAGAGATAACTATATATAAATATAGAAATACATTTATAATATTTATAATTATAATAAAATTTTAAAAAATTCAAATACCACATTGTTAGTAGAAGTCTATTAATTTTTAAAAACATCGCTATTAAAAGTCTATCCTAATAGAATATGTCATAAAAACTTTGGTAAAGTCTATCCTAATAGAATATGTCATAAATTCTTTTGTTATATTTACCGATGTTGATATACATTTAATTGAGTATGTCAAAATAATAATTGAGACAATGTTTCACCTATTAAACTATTTATAGTTTAGGAAATGTGCCCTAAAAAAAAAAAAGTAGGTAAGCAAGTAGAGGGAAAAATAGGTTGGAATTGCAACTTTGGGTGTGGTATGATTATAGTCCCCATCCAGTGGGTTTGAGTTTGACAAAAGGTTAGTACGTAACTCAGCAAGCAATCATGGACTTGACTACAACTCCATTTCACCCTTTTTCTTCCAATATTCACCGTCACTCCAATCACCTTTCCTCCTTCCTTCTTTAAAGCCGGCCAAAGTCATGTACTAATGTATTGTTCATTTCTTTCTCGCTTTTTTCCACTCTTTGTCCTTTCTAATTCTTCCTTTTCCATTAGCTTGCTTAAAACAGAAACAAGTCAACCAAATGATTTTGCCTTAATTGTTACATTCATTGCCTTTTTCTCTTTTCATTTCTATCCTCAAATCTCACCTTTTACTTTTTATACCTCTTCCTCTATTTTATGCAATAGACAAATTTTAACAACTATTAATTTAAATTAATCGTTTTTTTCATATTTAGATTTTATCTAAACATGCTTTTATTATATAATTAAAATTATAGTAATATTTAACTTTATATTAAAAGATGGATTTTGAATAGTTAAAGGCATGTTAAAAAGCACAATGATAACGTGGTGTTATAAACTATTTCAAATATACTTACAAAGGTGTCTGTTTGCTAGATGTGTAAAACTAAATGATAATGATAAGCTTGTTAGGTTAATTTATGATTAGAGGAGGATGGTTATAATTTTGCATTATAGAATAGGAAGAGACGTCAATTAAAGAAGAAATTGATATATAGGTTAGGATGCATCTATGTGTCCAAAGAGGAAGTTGTATCATAATGTATGAATGGAAAGAAAAAATAAAAATTGAAAGCAGGATTGTGTCGTAAACAGTGTCGATGAGAAAAATAGTTGGAGAAAAAGGGGCATAGTGCCGTTCGTTACGCCACCCTATTAAAATTATTTTATGTCTCTCTCCCTCCCTCCCTCTCTCTCCCTCCCTCTCTCTCTCTCTCCCTCTTCTCTATAATTCATTCGATTGTCGGGAAGAGAGTGGAGAAAATAAAACCAGAAATTCCAACACACAAACCTTCCACCCCTTCAACAATGGCGGATTCTAGGGTTTCTAGGGGTTTAAGGTGATACGGATTCCTAATTTCTCC

>Csa6M483320.1

GATAGGGGAAATCAGGAAAAACCCAGAAGAAAGAATTAAGAGAGGGATTGGAATTGAGGGGAGGTTCGAGGAAACTCAAAGAAGAAGGTAGAGGAGTGTTGTTGTTAAGATGGTGGACGAAGAAGAAGAAAAGTTGAAGAAGGAAGATGAAAGAATTGGGAAAATTAGAAAGAAAGAAAGAAAGGATTGGAGTGAAATGGAGGAGGAGGAAGAAGAAGAAGAAGGAAGGACGGCAGACGGTGGTTGGATAAAGTGGGGGAAATGACGTGGCAACTTCATGAGAGGGAAGGATTTTAAGAGAGAGAGAAAGAAAGATGTCTTTGTTTACTGAGTTAAGAGAGAGAGAAAACAACCATGGAAGGGGAAGAGAAAGAGAAAGAGAAAGAGGAGCTTTGTGGTGATTTGTAAATAGAGCTTTGTGGTGATTTGTAAAAAGAAAGAGAAAAAAGGTGTGAATTTTAATTGAATTCCGTGTTTCCCAGTTGGCTGTTGGTGGCACCCAGTGAGAACCTTCTTCTTCTTTCTCTTTCTTTCACACGCACACACCATCACATACATTTAAAACACTCCCAATACACAATATCCTTTAAGTCTTATTGATAACATATCTCATCAATTAACCTACTCAATTTCATCTACTTTCGTTCGTTCCTTCATTCATTCATTCTCTACTTTTCCTTCTTCTATTTCTAATCAACCTTCAATTTTATCTTTAATCACACTTTATTGTTCATCTTTTCATATTCATTCCTTTTTCATTTTTATTTTTATTTTTCTTTACAATTGTTATAAATTAAATTTAAACACTAATACATAGTTATAAATATTTCTACCAAAAAATCAAATATTCTAAACATAACTTTAATACAATAATTTTTTACTATCGATCTTATGATCAAATGTTGAATTCTTTCTATTTATATATATATACCATTTCTTTGGAGTAATGAAAACATTTTAAGCTTGATATTATTTTAAGTTGACTTCCAAAATGGAATAAGTAAACAAAATTCCTATCAAATATTGTATACCTATTCAAACTTAAGTTTTTTTAAAATTAAAAAAAATACCATAAACTTACTAAAAATTTACACAAAACCTTGTCAAAATTTAATCACAAAATACCTTAATCTAAAAAAAATAGGCAAAAACACTTTAATTAAATCCCAACCAAACTACAAAAATAATATCAAATACATGTATTAAATTTTTCACTATTTATAAGTTTAAACTTCAATTAATGAAATCAAATTTGATGATTCAATAATTAAGAACGCCAACCAAAAAATGATAATTATTATTATTTTAGCTAATAAAGGCTTATGATAGTGCTTGTGTTTATCTTTTATTGTTAAAATACCTACATTTTTCCAATAAATAATTAAATCTCAAAAGATAAACTATAAAATATATATGGCTTCAATCAATCTTAATCCATCTCTATTATTACTATTTCCTTACCTAAAAATTTATTCTAATTATTTAATCCATTTAAAAAACACCTAAAAAATTATTGTAAATAAAAACATAGCAATATTTCATATCTTACTAATAATACTTTTATTTTATCAATATTTTAATTCGTTATAATTATAATATATTTGAAAATGGTTGATTTTATGGAGGGGATCAAAATTAGATTTTTAAACCAATAGAGACTGAAAGTAAAAAAAAAAAAAAAAGCTTTCTTTTAACGATATATTTTAACTTGAACATTGGGATATTTATATATAGCAATAAAAATAGAAATAATTTGGAAATTGAGATTATTAAAAGAAAAGAAAAGAAAAGAATGGAACAAACCCTGACAGTTGACTTAGGGAAATGCGTTGGACTTTGGCGAAGAGATTAAGTCTTTTTCTTCTTTTTTCTTAAATAGGAAATTTAATTCTAAAATCAATAAATACATAATTGACGATAAATTACGTCGCCAATGTACGGATATCCCACGTCAATAAATATTCCTATCGCAATTCAATTCTTTTTCCTCTTTAATTTTC

>Csa6M490220.1

TAAATATAATTAAGCATTATTAATGTTTTTGAGTTTGAAATTATGTTTGTGAATATGATTGGAGGAAGCTGGAGTTGGACTTTTTCTCCATCCTTTTCATTCTTCTTCCTTTCAACAAACAAAACACTCACATACTCACATTTATGAGACTCCATATTATATTATATTTTACCACTTTCATTTAACTTCTTAATTATTGCTTATAAACAACACTGAGTTGGTAGCCACGTGGAGGACGGCCCACACTTTTTCCAATTCCCCTAAACCCGATCCCACTAATTTCCCCCACTTAATTGCCCCTTAATCCTTCTACATTATTTTATGCACTAATTTAATTTCAATTTTAATCTTTTTTTTCGCACTTTGAAAAAGTTACTTTTATTCCTACAAAAATCAAATTCATTTACACATTTAAAACATGAAATATGTGTTTATAATTCACAAAACAAATTTAATCTTCATTTGGTCAATCGTCAACTCCTTATAGCCCAATAATTAGTCATATTTTTTTATCCACCAACAATAAATAAATAAATAAATAAATAAATATGTATTATATTTTTATAATTAGACGTTATTGATTAAGACTGTTTTATTAAAACTAATATAAAAAGAAATCTAAAAGATCCTCAATATATTCATAATAATATTAAAGGTATCGGGTATTCAAAACCTTCTAATATAATTGTGTTTTATAAGTTACAAGAACAGATAGGGATTGTATTAGATTAAAGAAAAAGGGGGTTATATTTGAATAATAAAGATATGATCAATGAACATAATGAAGTGTGTTTTTGTTAAATAATCTTAATTAAGGAAGTTGAGTTTAGAAGATAATTGAAATGAAAATGCATGTGCCGGCAAAGTCCACTATAATGTACGGTCAACCAACTTCATTTTATTCCCCAATTACTTCTACCCAAATGGTTTTGTTTTTACTTCACCTCCTTACATTTAACCTATTAATACCATTTTCTTATTCATGCCTAACTCATCTTTTTTTTTTTTTCTCTTCTTCTTCTTTAATTAATTATGTTTATTTTACCTTTTAAAACTTTCTAACTCAGTCAAACCAATTTTATTATTATCTTTTCTCATCTAGTTTAAACTTTAAAGATATACTTATAAAACCTACGTTAATAAAGATTTAAAGATTTAAAGTGTCCGCTCTCTCATTTACAAATTTGAAAATCAATGTTCGTTGAATATATCAGTAAAAATTGATGGTGTAATGACGAAATTGAATATTTATAACAAAACATTCAAAACTATGGAAGCTCTATTTGAAAATGGGGTTGTATTTTATGTATTTAGCAGTGGAATTATAATTGAATAATTATTGGGTTTTGTAATATTATTATTAATTTATAGAAAAAATAGAAAAGTAAATGGTATGATTTGGAATAAGTAAAAAAGAAAGTTTATAATTAGAATTAATTTGGTAAAAGTTTGATAGTAGACAGTGGATAGAGTAGGGATTATGAAAGATAAAAGTTGTAATTTGATGTAAATGATAAGAAGAAAAGTGAGAGTGGAATCCCTAAAATTGAATTTGGAAAAGAAATAGTTATACAGGTTGTTATGGAGATGATAGAAGTTGGGTTGAAGGATGTTGAGGGTTTAGGGTTGACATTGACTGACAACTTGAAAAGAAAATGGTACAGAGAGATTATAATGTAAAAATTAGTTTTATACATAAAAAGAAGGCAAACAGAATAGACAAAAATTGTTGGAATATAACGTTAGAAATGTGTTTAAAGGAGTTTTTGACTTTGTTCCACAAACAAACACCAAAAAGGCTTTTGTATTTTTAACCTCTCAAATGCTGACATCCCTTCCGTTTGGTATGCTGGGACGTTTCAAACCGCCTTCTTCTTTTTTCTTCTCTACAATCACTCTCCTTTTCTCTTTCTTTCTTCTTCTTCTTCTTCTCCTTCTTCTTCCTTAATTCCATTTCCATTTCATGATACATTTTGCTTTACAATGATTGATTGTTCACC

>Csa6M490950.1

TCCTACTCTCAATTTCCTAAAGTGTTTGTATCACTTTGAAGATGATGGTTAGTTTACTTGACAAACTCTTAAAGTGTTTTCATTATACACATTTTATAATATGGGTGATAAACCTACCTTTAGTTTTTGCGAATGATACCCATGACACGTCATTTTGCTATATGGGGTATTGTAAGGCCAATATGTTGATACGGCGGAGTTGGTGCATTCAAATTCATCATATTCTAAACATTTTTCATTTTTCATTTTTATAAATTTTACTCTTTAGATTCTTAATCAATAAAATAATAAAAAGGTAATATGACAAAACAAAATTACAAAATATATATACACCTCAAATGTGCAAAGTACCTGCAACCAATAATTGATGGCATGGATAGTGATGATTTTGTAGAAAGGAAATGAAGAAAAATAAAATAGAGCATAAAGTAGATTTACAAGGTGGGGGCTGCTATTATTTTTCTTTTTGAATACTCAAACTCAATCAATAGGGCTTTTAATGGAGGCCTCCATTTTCACTTTCGTTCTAAGTTATTGGCCTTACATATATATGTCAACATTCATTCTTTTTTTCTTTTAATTCCTAACCAGAAAGGAAGAGATACTTTAAGTCAATTACCATTAACTGTTTTACTATTTACTTTCTAATATACTTTTAAAATGCATGTTTTAGTTTCAGGTTATAAATCGATTCTTATTTAATTTTTAATAATATAAAGTTCTTGTTCTACTTTTCCAAAATCATTCTACTAAATCAGCAAGACCACAAAACCCTGTTTTTTTGTTCTAAAAGAAAAATATTTTTTTTTTCTTTAAAAAACCCTAAAGATCCGCTGCTTTGGACATTTATTAAAAGAACAAAACTTTTTAAATTTAAAGTATGAAAAATGGGTTCCGTAGGTAATTCTCTTTTATTTGCAAAACGAAATAATATTTTGCAATAATTTGAAAGAGTGGTACTACTTTTTGTGTAATAAATTTTCTTTTTCTTATTGAAGATCATTGCATCCACTACATTGTGAAGGGGGGTTGTCCATATTCAATTTCATAGTACAAAATTTACCGTCAATCGACATTATTTTCCAATTGATATTGATTCTTTTATTCTACATTTATTCTAGCTTTAGCTAAGAAAACATATATTTATTCTCTTTTTTAGTACATCTTTTTAAATATGTCTGTTTAACAAATATTTTAATATTGCTCACTTCCCTCCCAATGAAAGCTCCCATAGAGAGAGAGGTACATAGATATATTGTTAGGAAAACATGTTTTTAATTTTAAACCTATAAAAGGAAAGTAGATAGAGAGGAATATATATTATATTCCGAACAGAGATTAAAAAAGTGGTATGTAATTAAAGGTTTAGAAAAAAACGAAATGAAAAAGAAAAGAAAAGATGAGACTCCTTTCAAATTGATGTCAACTAAAGTAGTTTGTAATTGAAATTATTAACAAGTTCCTTAACTTTTTAAAAAGAAAAAATAAGTAGTAAATTTCTTATAATTTTACGCAAAAAAAGGTGGGGGGTTGGAGAGGAGTAGAAGAGTATGTTGTAGTGAAAGATTAAAAAGGGAGAAATTGTAGGCATAAATGGAAGTAAAAATAAATGTAATGTTAGGTAATAAATAAAAAAAATGGGGTGGCATTTTAAAAAGACAAAAAAGAAGAAGAGGAAGTATCGGTTTTAAATAAATAAATAAAAAAAAAAAAAAAAATCTTGGACGGTCCACCATATCAATAATCAACCACGTTTCTAAAATACTACCTTACGTTTAAATACTTTCTCTCCGCATGCAATTTTGTAAGTTTATCACGTGCTCTTATTTTTCTCCTTTTCTTATTCCGACTCCTTAAACCTTTCCTATAAATCACCCCTTCCTTAACCACCACTATTTGCTCTAAACTACCATTTCCCCCTTTTTCCCTTTCTTCTCTTCTTCAACCCAACAACAACTCAAAACCCGCCGACGATTTTTTATTCTGATTTCTACCCAACC

>Csa6M502000.1

TAGTTTGTGGATATAGAACCCTCATAGATTATATTAGATCATCTCTAGCTTAATATGTACATTTCTCTCTCTCCCTCTCTCTAACTAGTGTTATTTTTCAGAACTTGGAATTAATTAATATATATAATTAATTGTGTTATTAATTATATGTCTCTTTTTAGTCTCACTATGTTGGGGTCCTTTTTTAACCTTTTTCTTTTCTCATACATGTGCCTTTTATTTTCTCAGTAAAAAACCCAAAATACACAAAATTTAAAAAAATTATCTCAAACTTATGCTCAAAAAACTTTTTTTTTTAACCAATAACTATTTTCTTGTAACAATATATATTTTTCTAATTTCTTTTAATATGTTTTCCTCTCCCACAAGTTAAAAACAGGTTAAATTAGAAGCGTAAAAACATTCTTTTACTTTCTCTATGTAAATAAAAAACAACTATTCTACTATGTAGAAATTTTGAACTTGGGATTTATTGTTTTATTTTACTTTGTTAAAATTGTACATACATAGATGATCTATTAAACACAGATTTCATATATTTGATTGGAGAACTAAAGTTATTACACTTTTGGAAATTAAAAAATCAAATCCACATGTTTCACCTTAAAAGTATAGTTCATAAGTTTTGAACAAATTCTATTTAAAAAAACAAGTGATAAAAAACATTCAATTGTAAAAAATTGGTAAGTGGAAGAGTATTGAGAGATGTCCACATCTCAATGCTAGCTATTGGGCATTGGGCCTGTCATGAGTTTTAGCTCAGTGATACCCTGTTGATGGGCCTTCAACATTGGCCCATCAGCTATACCATAGATTAGATAGAAATGTGATTTTAGTTTATGAAAGAAAAAAAATATATATATGTATAGAGGTACACAAAAATGAAATAAAAGTAATTAATTGAATATGTGTATATTTATTATTGTAACCAATGGTGATCCAATAGAGCATAAATCCAAAACAAATTAGTGGGTGATGAATAGGATAAAGTGCAGCCCACAACAATATTGTCAATGCCAAAATAAGCAAATCTTACAACAATGTCAAAATAACCGTATTGTTTTATGAAACCCCATTGTGAGAAAAATGCCAAATATCAATACTATCTCAAATTTTCTTTCTTCTGCTTCTATTACAACATGATGGAGAGGGATTCGACTTCCGATCTCTCTTAACCAAACATCCTGTCTATGCTAACTAAGTAATTACACTCCAAATTGGCCACCTTTCTTTCTTTTAATGGTATATCAAGTCAATTTTTGATCACATACATTTGTGTCAAAATCATATTTTCTCTTCACTTGGTTCATCAGTTTGAAAGATTTTGTGGGGGTGAAAGGAAATTTACATACACTTATAATTTCTTCCCGATTAGTTGAACTTTGCAATCTTGAAAATAAATATAAATGGTCACGTTTATAATTAGGAACGACAAGTCGTGTCTCCCACTCCTCGATTACGTAGGGCCCACTGTACGGACTCCTCCAAGAAGAAACGTCAAGAAAATTTCATTATTACCAAACGTAGCCACACTCTCCTACATCTTTTTCTTTTGGACACCACGTGAGTGCACCAATCACCACCGGAGGTTCGAATCATTTGAAACGTGGCAACATCTGAACAATTCGATGAATGCAGCGCCGTCGCAACCTAATTAATCACAAGCAAACAAAAGCAACAGCAAAAAAAGTTTTAATCATTTGATTAATTCCAAAATTTATTGGTTTCACAAGAATGTACGTCTCAGATTTTTGGGTTTCGTTTTTTCCCTCTCTTTTGGCTTTGCCTCCTCCATAGTTTTTTTTTTTTTTTTTTTTTTTTTTTTTTTTTTTTTGAAAAATGAATGGTATTCTATTAAACCCTGATTAATTGTGATTATGGGGTTTCTTACTCTTTTGTTTTATTTCTACTTTTCGTGTTTCATGCTCTTGATTTTTTTATTTCCTTTCATTTCCCAATGCAATAGACTCAAACAACAGTGAAACGGGGCTGCTCCAACA

>Csa6M511830.1

GAAGATTCTATACGTGAATTGGACTTTGTCTATACCAAACACAGGCTTGTTTTTTTATGTCAAAGCAAACAATCGTAAGGAATTTTCCTTCTGAAAGTATGAATTGATTTTAAGAAATGCATGAACTGATTCAAAAACATGAGTTCTAAAGAAGAATGATAATTGGATGTGGATTTTCATGAATCAATGGTAGCAAGGACGAAGACCCAAAAGAAAAACTTAGCAGAAAAAACACATAGAACAGTTTTCCGCGTCAAGTATTAGCACCTCTTGTATAGTTAGGCAAATGTTCATGAAAAGACGGGGAACATATCAGAGAAAAAGCCAACCCTAAAGATAGAACTTGGGATTTCATGGCTACAGGGAGTGGAATCAAAGGGAGAAGAAAAAAAGGAAAGAAGAAAGGAAAATTTTGGAGGTAGAGGAGAGAAAAGGGTCTGATTTTCACGGTTCTGTATGATGATATGGAGAAGGATTAGGTCGTTTGGCAGTAGAAAGACAATGAAAAAGTGCAGATAGTTCAGAAGCGTCTCGTGATTTGGCGTCTCTTTGATCAAAAGTCGAACTTTTTAAAGAAGGAACCGCTTTATTTGTTCTTCTTTTTTCAACTGAATCCAGCTCCTCTGTTTCTGTTTCATTTCCCATCATCAATGCTAAGTGGCCGAAGTGGCAAAGTCTCAATGGATTAGCTTTCAACTCTTAAGAGAAAATAAGCATATATGCCAATACCAATCTTTTATCCCTTAAAAAAAACAAAACAAAACAAAATAATAATAAAAAAAAATACCCACATTTTTTCTACAAGTGGCAGGCACACGTGGGCCAGGCTAAAGTGAAAAAGCGACCATCTGAGCCGAGTCAATAGTGAAGAACATGGAGGTCTGTAGCTTAATGCAATGGAGATTAGATCCTGTGGTTCTTAGTTAGTGCTTGTCAAATCCTTATTCTGTTAGCTATTGTTTACAACAACCGTGATTTGTGTACTTTGATTGCAATTCTTGCAATTACTTGTCTGTTGTTATTGCATTCGTCGTTTTTTTTCTGGGTTTAATCATATTCATAGTTTTGTACTAAAGTTGTCAGTAGCCAATGTTGTATTGTTATTTAATAACTTGTTTTGAAATTCCTTTAACACTTTCTTCTGCTGGACTGCTCGTCTCCTTTGATTGTGGGACTCATTCTTTACTTTGTTCCATAGCTTTTGAATGTTTGAATTTTTTTGTTTATTCTAACTTATCTTTTTACTCACCCTTCGCCTTCAAATAAAAGTCGCTGTTATTTATTTGCTTTTTTTTAAATATTAATTTGTGAAAAGTCTCTTGAGTACTCTGGCTTGATGCTTCAATTATTTTCAATAGTGTAGTCTTTGTTAAATATGTTAAATAACATGTGGTTTCATCCCAAAATCAATTCACAAACCTGTCAACTTTGATTTTCTAACCATATGGATTTTGCAAATTAATAGTGTTAAAATATTTTAATTAAATAACAACTCAGACAAGTTCTTCCAGTCAAACCACGAAATCAAACCTATGAAATAAAACATTAAAACAAAGCAAATTGAAAAAAAAAAAGATCAATGATTAGGGGAATAATGTATGTATCATATTGTGTACCAAAATGTGTGGAGAGGACTGTGGCAATAACATAGGAATTATGTTCTGTATTGCAAATAATTTTGTCACAGTTGAATAGCATAAGTTTAGAACATGTGTGAAAAAGGGTGTTGGCCCAATCACTTTTGATACATTTTTTGAAAGGTAAGCAGCGTCTATATTCATCGTTTTTGTTTGATAGCAATCTATGAAGCTCATTTTTTGTGGAGACCTGTAATTCGATGATATATATCAATATCGATGTCCAACTCGGAAAAGTACGAGTCCTTTTTTTTCTCTCTCTCATTAGTATTATTATATATTAAAATAAAATAAAACAATATTCCAAGAAGTTCTGCCATTTTATTCGAGGGGATTGAATTTGAGTTTTGAAAAAGGAGTTGG

>Csa6M513560.1

TACTTTGTAACACCATAAAGAATTATTGAATGTGCTCGCTCTACAAGACTACAAGTCACGTCACTGGTAACAATTGTTGGATGTTAATTAAGAAACCCAAAGATTTTAAATCTGTATAAAACAGTTGTACGAATTGGTAGCAATCAAATTATAGAGATGACGATAATAAAAAAATTGTTTTCATTGGGATGCGAGTGAGAAGTCAGATTATAGATGGCACCGTTTTTGGTTATCTTTTTTGTGGGTAACTTTTATTTACGTTGATTATTAAAAAGAAAAGAAAAGAGCTTAAAGAAGGTGATACCTGACGCTGCGTCCACTCCACCATCTTCCTTTATTATTTTGTGTTCTTTTTTTCTATCAAAACTTTGCCGGCAATTCAAGTAATCTCTCATCCCTATTTTATTCTTTTTACCTTACAATTATGTTTCTTATCCATATTTTTCTAACTAAGTTTCAAATTAAAGGCACGAATTTTTATTTATTCATTTATTTATTTTATATTCTTTCCTTTTTCCCTTTCATTTAAATTGTTTGTGGGTCCAATTCTCTTTTTTTCTTTTCTTTTTAGATACATAAATAACTTAATAATATAATGTACTGACCAATAAAAGAGCATCTATTACAACCTCTTACTCACTTACTTACTAATGAGTGACCGTTACATTATATATACACACACGCTCTTTTTTGCTGTGTTGTGGTCCTCAAATTTACTTTTATTTCTAATCTAATCTTTTTCGTTTTTAAAATGTATTCGGATTATTTCTTTTGAGTAATCCCCAACTTGTCAAAATGTGTAAATTCAAGTTTTGGGTGAACAATTTACTACAATATGAGATGACAAGTTGAAGGAAAAATTTTAGATTATCTTAATGATAGAGAGATTAATAGAATCTAAAATGTTATGTGTTAGTTTAAAAATAAGTTAATTTGAGTACTTGATAACTATTTAAAATAGATTTTTTTAGTGTGTTTCAAACAATTTTTTATAAAAATAAGTTTTTTTACAAAAATGAAAAAAATTTCTTCATAACACTGAATCCAAACTAACCCTCAAAAGGAGAATGTGACTAAAGTATTCAAATTTGGAATTGTTGACAACAAGAAAACTAGTTGTATGCTTTTGTTTGTTTGTTTGGGGGTTAAGATGTTTAAGTGGCAATGATTGGATCAAGAAGTTATTAAGTCCAAGAGACTAAAACAAAACTTAGAAAAACCCAGATAATTGTATTAAATATAAAGAGTAGAGTAGAGTAGGTTTGATGCACCAAATTATATTATATTCCTAAAAAATAGGAAATCACAAAATTAAAAAAAGAGAGAAAAAAAAAGGTAAGAGGATAAGGTGGGTAGGCATCATTGGGGTGGGAAGGTAAAGCTCATCATAATCAATTATTAATGAAATTTACAGTTACATTGGTATTAATTATTCATAAAGTAAGACTTGAAAAAAGTCAATCTTTAGATTAAGAATTTAATTTACAACACATTACACTGAACCCCACAAAAGAAAGGAGAAAAAAGAAGTGTTTGGAAGAATATGCTTTTCCCTCAAAATTACAACCAATTATATACTTAACCCACCATAATCAACGTGATTAAATCACGTGATGAAAGCATGTGATGCCATGTGGATATTAGAATTAGCGTAAGCTCTTTAATTACATTTAACTTCAATTACCTTAATCACCGCCGCCCAAAAATTGCCATTATGCTTCCTTCTTTGCTTTGACAGTGTTGCAATTTGCTTTGATCAAAAGAAGAAGAAGACGGCCGCCGCCCATTGGCTACAATGTAAAGGACAAATTCACTAAAAGGTTAACCAAAAGAGAACCGAATAACTTGTTCTCATCAATTTTCAAATAAAACCACATTCCAATTTGCCAACTCAATTTTACTCATCAAACAAACAATTTTTAAATCATTTCAATCTGCACTATAAAACCATCCCTTTTTTTTCTTCATCTGATCTATCCTCACCCATCTCTGTTTTTTCA

>Csa6M517390.1

TTTTTTCCACCCGATTCCTATAATTCCTAGATGGACCTCTCAAAAGATCTTGCAACAACAAACGTCCTTGATTTTCTCCCCCACTTGCTCAGGAAGAGGACTAAACTGAAGTTGTTCTCGGAAACAAAAGGGTATGATTAATTTGATGGCCCCAAAAGCTGGATTTCTTCAAACCCATCAAGACTAAGGAAGAAACATTCTTTGTTTCATCGAATTAAAGAAGCATTGAGATCCACAAAGACCCAACTCAATAAATAATCGAGATTCTGGATCCCCCCAAAATCCTATTCATTCCCACCAGCAAAACAATCGCTTAAAGCAAAAACCAACAACCAGAAACAGGGATGACTCTCAAGCCACAAAGCAGCAGAGTTCTGTTTAACACCAAACTTCCCCTTCCTCCTTTAATCCATCTTTTTAATTTGGTTTCAAAGTCAAACTCCACCTTGGGTAAAAAGCCTGGCAAATCCCACACAGAGATCCACAATAAATAAAACTCCAAAATAAACAAATAAATAAATAACTCTCTTTTTTACTATTCAAACAAGACGATTAACCAGAATCCGGACAAAAGAGGAAAAAAAAATAAAAAAAAATAATCCCAAATACAGAAGAAAAAAAGAACCCATAATTCCAAAATCAAGCAGTAAGAAGGAAAATCCAGGAAGGACCAAGACCTTACTTGAACAAAACGTGTGGAAAACCATTCAATTCACTCCGCATTCACTGAAGAAATCTGTGGAATGATCGAAGATAAGAAAAGAAACAAAGAAACCCATCTGTTAGGGGAGAAGAAGGGGTTAGTAATAGAAATAGAATTCAGAAATGGAGGATTTAGTTTCGCCTTGAATTTTCTATCGTCGTAGAAGAAAAAAGTAATTTACGTATTGTAGTGGGGAAGCAAAAGAAGGAGAAGAATAAGGGGCGGCAGTAAATAAGATTCCATAGAAGGGGATATTTCTTTTTTTCTTAGTTTGACGAAGACAGCAGGGTATTAAGCCTCTGTTTCGCTATCTCCTCTAAAATAATCTCAAAAGTTGCTTTTTCTATATTTGAGAAACCAACTCAATTGAAAAGGTAATCTGTTTCAATTTATCTCACAATAACTTTTTTAAAGAACAAAAATGGTAAAATATTTATTTGTTAACGAAAAAATTTAATTTATTTGTCTTCAAATTTTAGTCAACCTAATAAAAATGCGTGCTTTCAGTTAATTTTTTTAATTAAAAACAACAATTTCATTTTATTTATCTTAAAACATTTCTAAATCGAATATATTTGGTCAAATAATAGTAATTAATTCACAAATAATAACATATAATTAAAAAGAAAGATCATGAAAAAAGTGAATTGAAGTTTTTTTATTAAAAATAGATGTAGTACGAGTGAGGACATAAAAATGATAGGGATTAATGTTATGATAAGGTAAGTTGTAAGGTTAAATATTATGTTAAAACTTTTGATTGGTTTTTATAATTAGAAAGAAAAATAAGGAGGAGGAGAGATGTGACGGTGCATGGTGGGGAACCACATAATGAAATAGTCGTAAAACACGGAAAGAGAGAGAGAGAGAGAGAGAATAATATTTTGGTTCCAATTTTAATTTTAGTTTGAAAATAAAAATTAGGAAGAAATTGTGGAATTGTTGAGGTCATTTTCGGCCATAAATAATAATATTGATTTATTTTGGTACGGACGGAACTTCCTCTTTACCCTTTACCCAAACGCCATCCCATTTCCCTCCCTGCTTTCTACCCTTTTTTATTTTGTACGGTTCGCACGGATACACACTCTCTACTCTCTTTATATAATGTTTTTTTTTTTTCTTTTTCTAATAGTTAAATATTAAAATATCAATTAAATAAGGATATATTTTTTTTAAAATTACATTAATGGTATTTTGATGTTTATTCTATTGTTTTTTTAAATTTGGTTTGTAGATTTACGGCTTATAGGGTTTGGTTGATTGAGTTTGATATGTTTGCGTTACGTGTCGGATG

>Csa6M520410.1

TTGTTTGATTCTTCTCTCGCCTCCTCTTTTTTTTTTTTTTTTTTTTCCTTTTCAGGTGGTGGAGACAAGTTCACATTTTTGTAAACATTTTGATTTCAAAAGCTCTACCCGTAAGTTCTTATTCTCCTGCTGAATTTTTAGTTTCCCAAAAAAAATGCTATATTCACATTCAATGCAGTATCTTCTTAAGGATATAAATTTTGACTTTGAAGTAGCAGTTTTGTTTGTTGATTTGCTAATTCAATCATGAATTACATTCAGAATGATGAATTGGTTCTACTATCTCTCTATAAAATCAAATTGATATATGCTTCAACCAGGGTGAGAGTTTCTGCGGGCCAGCTTATGTAATTTTAGACTAAGATTACCTGATGATCATATTTGGATTTTACATATTTTGTGTTAAAGATCCCTCAATACTTGGTTAATTAATGGGTCAAGGAAGATTCGTTAAACTTGTTGTGATTATTCATCTTGATGGTGATAGTAATGGCAATTATCTGCAAAATGATATACTCATATACGTGAGCAGACTAAGAAGTTGAGCTAAATTTCTTCTTTAAGATCAAATGATAATTTAATTGGATTAGTTATATGAAGGTAAATAAATGCAAGCGTGTTTTCTATTCAATTGGTGAAGTTTAGCATCTTTTGCCCTTCTTTGTTTCGAATTTCTTTACTTAGAGATGAACAGAGAATCCCTTGTATGAGATATAGGCTGCTACTCCTGTATTGCAACTTTCTTTAAATTGGTGAGGTTTGAGTTTTAAAATAGATCAAGTTCTCTGAAAGGTTCGGCTGGAGTAGAATAGAACAAAAAGTATGCTTGTTTAGCAACAAGGTAATGAGGATGCTTCTCGAATCTCAACTAATAACATGAATGACATTTCTGTATCAGCTTACATGCCGCATATGAGCCTACTGTATTCCCACATATCAGATGAAAAAAGGAAGCAGGCAAAAGAAATAGCAGACAAGCTCGATGAAGCCGTGAACGGCCTGAGATTCCCCATTACTCGACTTGCACTGTGCAAAACAGACACTGCGGATGAAACTCTGAAGTCCTGGGATAAAATTGTAGAACACGATCTTTCTTCAAGTTAGCTTCTGTTTTTTATCCAAGCCCCTTGAAGATCTAAGCTATGTTAGTTTACCATATTTGTTCCAACAATGTCGAAGATCTAGACAATGTTCTGTTACACAGTTTACCGTATTTGTTTCAACGATGTCTGTACTCCTCTAGACTTCTTAACTGGAAAAAAAATAAATGTCAGCCGAAGGTCTTGTTTGGCAGCTTACAAAGCTTCGTGATTAGATTGTTTTGAACCTTTTCTGTTATTTAGAAAACAAGTTTTAAAAGTAAACTTTATTTGAAACCAAAGAATTACAGCAGAAATTTCTCAATTTTCTATTTTACTCTTTTTTCGTTTTTTATACAGAAGAATTATGCATTTCTCTAGTTGCATCTAAGAATGGGAACGTTGACAAATTTATAGCAATACAAGTGGACAAAGTTTGTTTATTTATTTATTTTAAGAAATAAACTATACAACAAGTGGAGTTTATTGTATTTTTGCTAAAATCGAGTAAATGAGTTGGAAACATCAGCTTCAATGGCGTTTTGCAAGTCAATTGCCGAAAATTTAAACCAATCGCCATATCTCAAAACTCAAAGAACGAGAACTGTTGTACCGCGACATTCCTTTCTATCTACGCGCGAATACGTCGTCCTCATCATCATCACTAGGCCCACCTTTCCAATCCACTTTTCCTAAAAATCTTCTTGCTTTTTCTCATTAATTGTTGCCCTCACTATCTCTCTCTTTCTCTCTTCAATAAGCATATGCAAATTGCCTTCTGGATCGACTACCAAAGTTTACAGTGAAGCTTTTTCCAGATCCCACAAACCAACCAACTGGTTTTTAATAGTTTTGCCTTATAAGAAGGTTTACTCCGATTCCGACAGATACTTGTTCTTTCATTCTTTTGTTTCCGTGGCA

>Csa7M017160.1

TAGGGAAAAAAAAAAGCAATGGAAGAAAAAGCAAACGGCAAAGGAAAATGGAAGGCAAGAAGCTTTCGATCTTTATTGCACAGAGAAAAGGTTTTACGTGAAAAAGCAAAACGATGGTTGATTGTGAAAAAAGAAACCGATACCAAAACCAAAACCAAAAGCCAAAAGCCAAATCAAAATCCAAAACCAAAAACCCGAGTTTATATGAAGAGACACAGATTTAATATGGCCAAATTAAATACTCAAAAATGGAAATTCATATTCTAATAATGAAATAATTAAAGGACCAACAAAAAAAAAAAACTTTCTTCTCTTTATTTATTTATAAATGTTTTAATTTTTTTTAAAATTAAATTAATCAATTAAAGCGATTTTACGGAACGGAGGATCAGAGGCGAGGAGGATTCTATAGCTAATCCGGCGACGCCTTTTTTCATTTTCAATTAAAGGAGAGGAAAATTCTTGTGCGTTGTGGTGTGCTGTTGTTACCATGAGGATCAGCACTTGGCATCTTAATTTTATCTGAAAAAACACCATTGGGCCCCATCTGGAAAATGACCCTTTTTTGTATACGAAAATATAAATTTATTTCTTTATTTTTTTTAATTTCTTAGTTTGTTGGAGTCAAATTTGGAGAAGCTGTCAAAATATTCTCTTCCTACCATTAAATTATACAAAAACCAACATAATTTAATTTAGTCTCTCCATATCCAAATCCTTTTTCTGTCCAAAATTAGCCAATAGCAATCCAATTTTTAAACACTAACTTTATTGTTATCATTTATCAAAATATGAAGTAAAGATGTCGACACGTTAATAAAATATAGAATTAACAAATCTCTAATTACAACTTCCTGTTTAACTTAAAACAGTTTTTGGTTATTTTTATTTTGAAAGTATAACTTATTTTTTTTTGCAAAGGTATTGTATTAAATATTGGGTGGGAATTTGAATCCACAAATGTCAGTTGAGCTAAGCTCATGTTAGCGTATACTTTGTTTTGTCACAAATCACAAATTTTTAAAAGTATGAAATAAATTCAAACTATATACTTTGTTGAACGTATTAAAATCAAGTCTACTCGATAATGTTGTGATTTTTCATTTTTTTTAATGTTTTTTAACGGCTTTCAATTTTGTTTTTTTCGTGATTAAATTTTAAATATCATGATAAAACAGTTTTAAAAATATGATCTATACCTTTAAATCATCCCATTCACTCCAGAAAAATAAATTTTTGTTATGGGAAGATTTAATATTCTAGAAAAAAGAAAAATAACGATAAAACACTTAAAAAAGAAAAACAATGTATGAGCACCACCGCCACGTGTAAGATAGCTTTGCTAATTCACTCACAATTTTCTTTTCATTGGTATTAAATTTGGAATAAATCAAAGCTTCATGAATAAAATTAATGAGTTAGCTGAATAGCATACGTGAAAACTCTGTCTCTAAAAGGTTGAATCTCATATTTTTCTAACTATTGTACTAAAAAAGATTTAAAATTTAATAAAATATGGAGAAATTTAAAAAATTTATATTCTCCCTGAGGCTTATAAATTCCTTAAAGTATATTAGAATAACTTTAGAAAGGACCTGTAAAATTGCAACATTTTTTAAATATAGAGTAAAATATTAGAATATGTAACAAAAACATATTTTCGGTTATTTAATTTTGAAAAATAAGAAAAATAGGAAGGGGGGAGGAGGTGGCGGATAATGGAGGGTTCGAGCAGGATGAAGTGGAGGGAGGGGTTTTGGATAATGTAAAATTAGAAATCAGGATGTGTGTGGGTTTTATATTTTAAATTTTTTTTTAAAAAAAAATTGTAGAGAAAGAAAAAGGGGAAAAAGAGGGGGGTCCCACAATAATAATCTTGGAGAGGAGGATATGTATTATTTCTTTTTCTTTAGGGAGTCCACTGGGCATTTTGCCCACATGAGGTCCTCTCTCTCTCTCTCTCTCTCTCTCTCTCTCTCTCTCTCTCTCTCTCTCTCTCT

>Csa7M043040.1

CCTTCTTTAAGTTTTGTTTTCTATTAAGTTTAGAGAAATTATATGACGTGGGAGTCATAAAATAGTGATACATATTGTTGAAGTGACATATTTCAATAATTTGTAACCCTAAATTTCAAAATATGTTTCAATTACTACCCATATGGATTTCTTTTATTTTTCTACACATAGAATCTTTTAATGGAGCTAGAAGAGTAACGAGTAAAAGATGGTTTGAAACAAGTTTAAAGATTGATGGTCAAGATGATAAGTGATTAATTCTAATTAATTCTAAGTTCAAAAAAATGTTTTGTATAATTAATCATTATTGTTTAGGGGTAAAAAATACCTCACAAGTTAATTAAGGAGCTAAAGTGTTTTTTCTTTAATTTCAATTAGGTTAAAAAATAACTTCATATTTTACAATTTCTATGGATTCAACATTCAAACTTTCTTGATCAATATCCTATGATTTAACAACTATGATTGAGCTTGCCTCTTTTAATTAATTTCATTATTTGTACAAACAATTGTTTTAAGACCAATAAAAAATAACAATAATCAACTTTAAAAAATTATTACAACTCTTGAAAGAGATTAACTATAGCTATAACTTCTTGTTCAATGTGTTCTAACTTGATTTGAAATGTAATTAATTTATTAATTGTCTGAATCAATGAATAGTGGGTGGACTAGTAGTAGAAATAACTTTCCAAAATGGTTGCCCAAAAATCCAAAAAGAAAAAAAAAAAGATGCCAAACATATCCAAATTGGTTGTTGACCTTAGTATATTACTTAGGATTCCCACTTGACAAACATACAATGTCAAAGTCTCTCTATTTTAGGGAACAACACATTTTTTTAAACATATTGACACCTTCCCTACCTAAATTCAACTCTTTCACCTTAATAATATTCTTTTAAGTATCAATGTTTCAACCCCATTTGGTGGTTCAACCCACTCCATGCATTCCCATACTATTCCTACCTTCTCTCTTTAAAATTTTCTCCATTTTTACTTTTTGGAAACCAATCCCATTTCTTAAAATAAAGAATTTAACTTCTAATAATTTAAAACTTCCACCAATCAAAATATTAAAAGTTGATAATACAATTTTATAATCATAATATAATTAGAATAATGTTATTGACATATTAATGTTTGTTTAAAAAATCAAAATAAAACAATAAAACACTTAGCGGTAGAAAAGATTAAAAAAGTAAAGTACGTATTGTAAAATCAAATATAACAAATATCAAGATACGACTCGCAAAATTATTAAAGTTTATCTTTTAGAACGTGGGAATTTGTAATTTTGATATAATATACAAAGAGTTATATACTTTATTAGCACTAGTCTTTACTCATTTAATTTGATGTAGTTTGATTTAGTCTACTGCAAACCGACCCCAACTTAATTTAAACCTATGTCAAACTAAAAACACTCCTTCTCCTCATTTAATTTATATATAGTCTATATGTTAATTTAATTATTTATATGGTTTTGCTCGTTTTAGTGTATGTGCTTTTACATGGGTCTATTAATTCATTTTTTTGTACGACTGGTGTGCACTCAATATTTACTTGTTATACCCTAAAAATATGAGAAGAAACTTTATATTTAAAAAATATAAAGATGAAAGTGAAAGAAATAAAAGTATAAGGAAAAGTAATGAAGTAGAATTTAAAGTTGAGTGGAAACCCTAAAAAAAGAATTTAGTGTTTAGACCCCAAATCACATCCTTTCCCATCTCGGATATCTCTTCATTCTTTTTTCATACAACATTCCCAACACACGTACGAAGTAAAACAGTTTAAGCAACCAATAAGAACTCAAGTTCACACGTGCCAACCCTTCACCCGTTCTAGAAATTAGACACGTATCTTTTTCAAACCAAATTGTCAACTCTCAATAGATTCATAGACTCAACTGTCCACTACCAACTCCCCTCCTCAATTCCCACTATTTATACTCTTTCCCCCATTTTCCCCTTTCACCAAAACAACAAAACCTCCTCC

>Csa7M051390.1

TGAGGATGATGGAAGAAGATGGGGAGAAGGGGATCGAGAGCTTGAAGGAAAGGGAAAATGGAGGGGAAGCTCTCGATGCCGAGAAGGAGGATGGATGAAAGGGGTAACATGAAAGGGAAGAGAGGGAAAGAAGGGAGAGATCCGAATGAAGCGTAGACAAGGGGGAACAGAAAATTAAGGCAAACCAATTTTGTTGTTGCAATTTGTAAGCATTTTTGTTCAATGAAAATAATGGAAAAAATACAGAGCAGAAAATCCCTTTTTTTCTTTTTTTCTTTTTCTAAGACTTTCTCTAAACACCTTTTCTGTGGACCCCAACCCACTCTTTTGTTTGTTTGTTAAAATCAAATATCAAAATAGATTTTCTTTTCTTGTTTTTATCATTTTCCATGCATTTTTTTCCTTCAAAAATAGATTATTAAACCTTTTTAATTCCCTAATTACCATTTTCTTCAAATTTAGAAACAATAAATCTATCAATTATCAAACGGATAAGATTATTAAAACAATAATAATGATTATGCTATACATATAGTTCAGTGATATCATTATTTAGAACTAATTATCATTACACTCTTCCATTAATATAATTACACTCTTCCATTAGTGGCAAAATTTGTGTATCATATCTAATTTGCATATCTATTATATCTACCCACTCAAAATGGGCACACACAATCATCACTTACTAAAACTTTTTAACTCTTATAATATATTCATTTAGGTGTTACTTTGTAATTATGTTATTCACCATAATTCACATTATAAAAGTTTAGCTGCCTAGTATAGAAGTGTAAGCAACTTAAAAATGAATGAAACTGATGGTTGGTTTAGACTGAAATCGGTTGGTTTGAGATTTTTTTTGTTAGGGTTTTAGGTTTAAACAATCAAATAAAAAGTTTCATTCATTTACTTCTCGATTATGAAGTTGTCTATAGAGAATACAAGAGTTCCAATTATTTTCGCTTATCAAGTGAATGAGAAAAATGCTTAGATAACCTCTAAATTACGAGTTTCATTAAGAGAAAGAATACAACGGTACATTTAATTTAGTTTTACTTTAGACAAAAGATGTTCATAATTGTTTCCACCTCCTTAGTTGGCTCATAAACTGATCTAAGCAATTGAGTTGACTTGGTTTTTCAATTAACACAGTTTGATTTCGATTTCCAAATTGTTTTTCAATAATTAATCAATTCGGTTGGTTTACGAAATAGGAGCATTAGACAATAAAACAAATTAATTATATATCCTTAAAAATCTTACATATAGAGCCGTTTTGATAAACAAAAAATTTCTCACAACATTTAAGTTAAAGTAAGCTTAGCTTGACCATAACTAATATGAACTAATTTCTTCAAGTCAAATTCAAAATCCATATACTCCATTAATTATTATTGAATTAAAGAAAACAAATATATTATATATTTTTTTAATGGGAAGAAAAAGACTAATTTTAATGTGAAATATGAAATAAAAAGGCAATAAAGTATTTGTGAAAGCAACAATTCAAAAGAAACTGCAACACTCCTAGGTAACTTCATTCATGCATCAATTTTTTTTTTTTTTAAGCTATCCCAATTTCTCTTTTAATTAAAGAAAGATTGATATTGTTAAAGTAAGCTTAGTTAGGTATGGGCAAAAAAATTAATTATTATTACGTCAAATGAAGATGAACTTAACATGAGTTAATTAGTTTAAATTAGATTCATTCCTAAATTATTGTTCACTAATACATACCAACTTAAAATAATAATTAAAAAAAACAGGAACATGATAATATTTGTTTAAAGATTAATGGTAAATTACCTGAATTCACTTAATTAATTTATTTATTAAAAAGAAAATTAAACCCTACTAAATTGAGAAAAAGATAAATAGGAATTTTAAAAAGTAAAAATGTAAAGAAATACTTGTTAATAAAAGTTCAAAAAATGAAACTGGTTAAAAAAGTCAATTAGAGGAGATTTAGGGGGGAAAAGTAAAAAAAGGAAAAGGTG

>Csa7M234730.1

TCCTTGTTAATGATGTTTTCTTTAGTTGTGCTGAGGAAGATTTGAGGAATTTCAGAAGGGAAAAACTGAAAAGAAAAGAAAAAAAACAAAAACAGAAATTTTGAAATGGGTTTTGTTTGGATGAGAAGAAGAGAGAGAGAGAGAGAGAAAGAGAGAAAGAGAGAAAGAAAGCCTAGGAAAGTTCACCAAGAAGACAAGGCTTAATTGCCAACTACTCAGAGCCAACGAAGCAGAGGATTTCACAAACAAGCCAACCCCACTAAATAATCTGACCCCATAAAAACAAAAATGCAAAAACCACAGCTAATTACATCTTCAAACTTTCAAATACAATAATCGGGTCTTTGATATTTGGTCGTTGAACTTATCTAATGACAAAAACTGAACCTTTAAACTAACAATTATTACAATTTCTAAGTTTGAAGGCTTCAGCTACAGTTATATCTTTGGAATGGATTCTACAATTGGTTCAAAAAGAAAAAAGAAAAAAAAAAAAAAGTCTCTCCTCTTCACTTTTAGCTCTTTCTCTGTTTCATCACGAGCTGTAAAACCTCTAAAAAAACCCAACACTTTCTAGGGACCATAGAAGTTATGAAAATAAAATTAATAATTATTTTGTTTGTGAAGATTTTTCTTCTTCTTCTTTCCAATCACGAAAATCTTTTGAGAACTTACTTTCATTGGAAGTGCTAGAAGAAAGAAACAACAAACTAACCTCATCTCTTGTTGGTTTGTCTTAACTTTTCTCTTTTAAAGTGTAAAAAGTTTAAAAACAAAAAGAGAGATTTTTATTTTGTACAGAAAAAAGAAATCCAAAAGTAGGATATTTTGTAATTTGTTTTTGTATCTTTGCTTTGTTGGGTTTAATTTTGTGAAACTCAAACTAACTTTAATTCATTTATGATAACAACATGTCCTGTTTTTTTTGGATTGGCCACTCACTTAATGCTATAATTTATTTGTGTGTGTGTTCTTTTTGAGATTTACTCATCCAATCCCAAATAAATATCTAATAAGAGTTTGTTTGTTTATATCCAATTTTCCTATTTATGGATACAACATTTGGGATGAATTACATGTTTAGTGTGTATGGTCTATGGATCTATTAAATTTCACAACTTTCATCCTATAAACTTTCAAGTTTGATTTAAATACTTTTATACTTTCTTCAAAAACATACTCATATGATCTTAACCTTCTGATTTTGACCATAGCTACTTTAATCATCTATTAAGTATATAATTAAAAGTGAAAGTTGGATTAAAGTTAGAATTCAATTTTATATTCAATACATCTAAACTCAATAAACACAAATTTTAAATTTTAAATTGAACTTATAATTTTACTAAACATTTATTATGTTCTGTATAGTTTTACAATCTAATTAGTATGAGCTTTTTAGCTTGAGGTTATAGTTATTTTTATCATACCCTACTTTTGACATACTAAAAAAGATTGGCCGAATATTAATTTAGAAATTGAAAAGGAGGAGAAAGGGGTCATAGGCATAGGCATAGCCATGCAGATAGGTTTATGAAAAAGTAAAAACTTTTATTAAATGTTACCATTAATATTAAGAAAAACTGATGTGATCATATCAGTAATTAATATTACCTTTCATTCAAAAAGTAGAAAGTTCAATTGTACTAAAAAAAACTTGTTCCAATATTAAAAAAAACAAGACTTTTTGCCAAAAACAAATAACAAATCCAGTAAAAATTGGGTAGCTGATCACTAAGGTTGCTTAATGGATTTATGAGGCCTACTAACATTTAATTTGGTAAGCATTGTAAGTATACAACAACAAATATGTGGCAATATTGTTTTTACAATAAATCATTATAAACTAAGATTATTTTTAAATATAGTAAAAATAAGTTAAAAAATATTTATAAATATTTTCAATAATGTTGTGAATTACATCGATGAAATGTGTAATATCAGTTTGGAAATCAAAAGTTTTCATCCATATTGTAAAAGAGAAAAAAAAGAAGAGTGGG

>Csa7M378450.1

CTAAGAGTATATTTCAACATAGTTTGTATGGACATCTTTGATATAGGGTTGCCTGCCTTGGGAAAGAAAGGAGGAGTGAGGTGTTCAATGGAAGGAAAGGGAAATGGAGAAGTAGAGAACAAATCAAGCAACTTGGGAATGGGGGCATCGTTGATAGCTGCGGCGTGTGCTGCAACGATGTCAAGCCCGGCGATGGCTTTGGTGGATGAGAGACTGAGCACTGAAGGGACTGGGCTTCCATTTGGTTTGAGCAACAATCTTCTTGGTTGGATTCTGTTGGGAGTTTTTGCTCTCATTTGGGCATTCTACATTGTTTACACTTCTACTCTCGAAGAGGATGAAGAATCTGGCTTATCACTTTAAGCCAACATTTCTCCATCTTACTTACTATCATTAATTATCGCTACTTTTGTTATTGTTGTGGATATTTTGTCTTTGGCTTGAATTCTGTAGAAGCTTTTGAATTCATATCATACAAGCCTTTCGTTGGGTATCAATGTAGAAAGCCGTGAATGTGATTCATAACTATGTGTCATCGTTTCATTTCTCTGATCCTTTTTTCTGTATCGTAAATTACCATTAAGTTTTTGAAATCTAAGTTTATAAAACTCTTTTTCATTTCTAAATTTTTTTGGTGTGTTGTCCAAAAAACCGAATTAAGTTTTAAAAAGTAAAATACAAAACATAGTTAATTGATTGTTTAAGAAAAGGGAAAAATTAATTTAGGGGATTTGGGAAAAACCAAACATAAATATAATGCAAAAATTAAAATATTTGTTATATAGATTATATTTGACATCTTTTATAAATACGTATAAAATATAGAAAGTATCTTATTTTTCAATGAGAAGGTAGAAGGTAATAAGAAGTCTATAGCTTGGGTTGGAAATATCATTGAATTTGCTAATGTCTCTATTTTTGTCATTTTTTTAAACAAATATCGGTAGAATTAATCATATTTCAAATATAACCAAACGTAACAAAATATCATAATCTACCTACAGTTTTTTGGACAATTTATCAATAATTTTAGAAGTTTTTTCCTTTAAAATATTCTTTTTACAAATTTTTTTCTTTTATATAAAAGTATATTTTATTGAGACATAAAATGTGAAATTTATTTTTTTTAGAAACTTTTAAATTTGAAGTATAAATTTAGATATAAAATTTTGTTTTTTAGCGGTTTTTTAAAAAATATAACAAAAAGACATAAAAGTATTTACACTCTATAAAACTATTTTCAAAATGAAAAAACTCATAATCCTACCGTGAAAATTACCACATGCCCAATAAAAATTACAAAAAATGTCTCCATCAATATTTAGATACGGATACTTTTGCTACACTATCGTTTATTAAAAAAATAAAAGAAAAGAAAAATTGCATAAGAGTGAAAAAAAAATGATAGAAAGGAAACACAATCCTATAATATTTTTTTAAAAAAGGATTGACTTTGTCGTGACTTTTGATTTTATTACCCAATCAGTAATATATTTTAATGTTTTGTTATATTTATAAAAAATTTCCTTTTAAACTACTACTAGAAATTACTAATAATGGTATAGGAGAGTTTTTTATATAAAAAGATAGTATAAAGAAGATATAGGAGAATTTAGGTAGATAGAAAAAAATAGTGAGTATTTCCATCATATAATACTTTGGGCTTTTGTTAAAATTTAGATTTTTTCACGTGGATTTAGAGAATATATTATTTGATTTTTAGAAAAATAAACCTTAATTTAACATCAACTAATATGTGAATGTGAATTGTGAAATTAGGAATTTAGGAAACTTTTTAATGTTTTTAATTGTGAAATTACCTAATATACCCTTATAACGACCCACCTTCCAAAGCGAGAAAATAAGAGAAAATCTTTCCATTCAATTTCAAATAAGGAAGAGTAATGTTTAATTATTATTTTCCTTCTCCCTACAGGAAACTCATATGTTCTTAATAAAAATGCTTCATCTCCACACTCAAACAATCTCTCCTCCCAATA

>Csa7M387170.1

GTTTTAATAAAGCTTTATTGATAGATAGATAGATGGATTGAAAGTAGAGAGTATAAAAGATAACAAAGCAAATTTTGAAAACACTGGGGAACTTTTACAGAGGCATGGAAGACAATAAAAACCAAACCAAGCAATTGGTCAAAAAACCTTGATGAAGATGACAAGAAGAAGAAAATCAGTAAGTTACCAGTTCAACGAAACCACCATGAAATCAACTAAAACAAAAACAAAAAAAAACAAACAAACAACCAATAATCTCTCAGCCCTTCTTTTGTTTCTTTTATATAAAAAAAGGTTAACCAAGGAACTCAAACGTTTAATAAAATATTATGGTACTGAAAAATAAAAAAACTGGACTTGCAAATACTGTTCCAGCCAGCAGCCACCCCCACAAATCTAGGAGCTAAGAACTGTAAAATTTGAGTATCTTCGACCAAATTCTGCGAATTTGGCGATAAAGAAGGCTAGAGAAAATCAACCCATATGCAGAATCGTAAGAAAAAACAATAAAAAAGAAAGAAAGAATAACAGAAATGAAGGAGGCAAATACCCCTTCACAGATATGGAAGAGGGGTTGAAATGAAGAAGCCAAAGAAGGGAAATAGAGAAGAAAAGAGAAATGGGTATTGTTGAATTGGTGAAAGAAAGCGAAAGGGAGAGGCAGCTTTTGTGGACAACAATTCACAGAGCAAAAAAATGGAGAGTAAAGAATCGGAAAAAGGGAAAAATAAAAATAAAAATAAAAATAAAAATAAAAATAAAAATAAAAATAAAAATAAAAATAAAAATAAAAATAAAAATAAAAATAAAAATAAAAATAAAAATAAAAATAAAAATAAAAATAAAAATAAAAATAAAAATAAAAATAAAAATAAAAATAAAAATAAAAATAAAAATAAAAATAAAAATAAAAATAAAAATAAAAATAAAAATAAAAATAAAAATAAAAATAAAAATAAAAATAAAAATAAAAATAAAAATAAAAATAAAAATAAAAATAAAAATAAAAATAAAAATAAAAATAAAAATAAAAATAAAAATAAAAATAAAAATAAAAATAAAAATAAAAATAAAAATAAAAATAAAAATAAAAATAAAAATAAAAATAAAAATAAAAATAAAAATAAAAATAAAAATAAAAATAAAAATAAAAATAAAAATAAAAATAAAAATAAAAATAAAAATAAAAATAAAAATAAAAATAAAAATAAAAATAAAAATAAAAATAAAAGTAGATTGAGGAAGAACAATAACAAAATCGACAAAATGGAAAAAGGGGGGAAAAAAAGAGGAGATTTTATATTTCCCTTAAAATTTAGAAAAAAGGATTAAGCAAAAGAAAAGAAGTTTTGTTTCCAAAAGAAAAAGAAAAGAAAAGAAAAGAAGGCAAAATTGGGGCTTATGAAACGCATACGCCACGCCTTGCCTTCAACGGATTTAACGGTATTTTATTTAATTCTTGTTTTCTTTGTTTTATTTATTCCTTTTATTCCTCTTGGATGTTCCGTACAACATTTGGTTTCTTTTATGAAATTCCTTAAATCACCCTCTCCCTCTCCCTCTCCCTCTCCCTTTTACTTTTCTTACCAATTACATCTCATCTTTTCAACTCTAAATTAATTCTAATATTTCAATATAAGTATACATAATTTGATGGGATGCCTTACTGCATTTCAAACCCGCTTAATCATTTCCCTCAAATTTTTAATAACAAATAATATTTGATAGTCAATCATATTCTTTTATCACTAACGTTCATAGCTAGTAATAAAAGTTTATCAAATTGCTATCATTGATAAAATCTCATAACTTGTAAATATTTTATTTTTTTTCTATTTTTAAATATGCCCCTTATTTTTACGAGAAAATATTGTTTTTGTCGCTTTTTACTTTTGGATGGTTTAATTTTAATTCCTACATTAGTATATCTTAAATTTAATTCTTCAAAATTGGTTAAATAATAATAACATTATAAATTAACAACAAATTTGCAATATACT

>Csa7M407720.1

AATTTTGGTATCTAAAATGACCAATAATTAAACTTATAATTATGATAGTTAAAAATGGATGCAAATCTCAAAATTGGAGAACTAAAGTTACCGTTATCGTTTATTTTATTGAAAGTCGAAGATTTGAATCCCGTCTATATAAATAGAAACACTTTTATCGAGAAAAGTGATGAGATTTGTAGTAATGTAATTGAATGCATTAAATGTAACATGTATATGTTGTTAAGCAAAAGGTCAAACGTGCCACTTCAAATATGGTAGATGATTATTATCATATTAAATGTTTATGATCTAATACCAATTATTAAACTACAAACTTTAATATAATGTCCTTATGAATATTTAGATTATTATAAGGATACATATACAACGTCACATTATTGCACAATAATTAAGTGATTTATTACTATATAATTTTGAATAAGAAGAATTTTATTGTCAAACTTAGCCTAACAACTTAATTGACATTTGAGGGAAATTTTCCTCATCTTTAGAAATATTTATTTTAATTTTGGAATAAAATTACCATATTATGTCATAATACTCTTTTAAAAAAATTATCTTCAAATTTTAATGTGAATCTAAAAAAAATTATAATAATAGAATCATCTCACGTCATTTTCTAAATTTACAAAAGTAATAAACTTGAAAGCGAATAGTTCTTAGAGAGTCATATGGTGCTTTTTTTAAATTTTTTTTTTTTGTCATCTAATACAATTTTTTATTTTAAAAAACTTATTAAGTTACTTGAAATTTTAATTTAAATAATTTGAATGAGGAAAACAAAAGTAAAATAAAATATTTAGGTATTATTATAATAAAGATTGAATACAAATTTAAAATTGCATGCATGTGGGGGGTTTTGAATTTGCAAATATATATATAGAGAATTTTGGTAAACATATAAACAATGATATTTTTTTGAATAAAAAAGGTGAAGGTATGTTCATCTCTTTTAATTAATCCAACCCATATCAATTGATGATTGTTTATAAATTTGTTAGAACAATTATTCTTTATTTCTTGTATGGTAAAGTGGTTGGTTAGTAGTTCTTAGCTTCAAGTGCAAATATTATTCAATGATTAGAATCTTACTTTTCATTTTTGAAGCAATCATATTCTTCTGCCTGATGAACCAACGTTATAGACCTTAAGTCTTTGTTGGATAATAACTTTGACCATATACATTAATTTGTAATAAAAATTGGTATAATTGTAATAAAATTTAATTAAAAGTCCAATCGTAATTGAGTTCAACGCAAGCATATTTGAAATATTATTTACAAAAGAAGAGTAAAATTGATTTCATATTCTATTGTATAGACACTCACAAACTTTGATTAGTATATTAATAGACATATGAAATATGAAATTTACATAGTTAATTTATTTCATAAATAGAAAATTAAAACAATTTCGATTTTAAAGTGTTACTTTTAAATTTTCATCTTATATCTTTGTTTTTCAAAACATATTTCACCTCTTAATTTGATGACCATCCAATTCCAACCTTTTGATAATTTTATCACTTAAAACAATGGTTCTAATTCAAATATTTAATTGAATTTAAAATAACTAATGTCAACATCGAAGCTAATAATAAATATTTAGTAAAAAATTAAAATTAAAAATGTAAGTGTTGAGTTGAAAAAAAAAAAACTCAAAAACAAATTACAACCTTTCCATAAATTAAATTAAAATCAAACTAAAATTACCTATAATATTTTAAAGAAAAAGGTATTCTAGAAAACAAATTTAAAAACTAGAAAAAGGTTATTATTTAGAAATAAATTTAAAAGATACGATAAAAGAAAATATTTAGATTTGTAAGCTTAATAAAGTCGATCTTCGAATAAGGAAAAGAAAGTCGAAAGATTATTTTCCTTTTCCTTAATGGAAACACATTTTTCCTTTATAAAACTCAATCTCTCCACGCTCTAACCATCCATTCTTCTTCACACATATTCATTCATCCTTTTCAAGAGCTTTCTGTTTCAACA

>Csa7M430790.1

TGTGTATTTGGAAGGAAGAATGAATTGAAAAGGGCAAAGAAATTGGGGCCGGGGAAAATAGGATTATGAGAATTGGTAGTACGAGGGGATGATTTTGAGAAATAGGGTTTTAAGAGAAAGAAAAGGAAAGAGTTACGATTAGCTAGATCGCAGGTGGCGAGGAGCTCGGACGGCCTGCACGGTCAGCACTGGACGGCAACGGAATCGGAATGTAAATTTAATAAAGCGAGAATTCATAATGAATTTTTGTTAACCTACATCGCAGGTAGCAGGTAGTTAATTTGGGTTACGGTCACTGTACTCCCCGTCACCTACGTACCAAATATTTACTAATATGATATAATTTTTCTTTTCTATAACTCTATGATTAAATTGCAGTTTATATCCTTTATTTCTTTTTAGGTTTAATATCTATTTGTTCCTTAATTATTATGTTTCAAAATATATTATTTATTTAGTTTATAAGCTTTTAATTTTGTTATTTTGTTTTAAAATGTCGTAATTTTATTCTTTACATTCGAGTCTAGTTTGAATTTGTCTTGAATTTCAAGATTTTTACATTATTAACCTTCACTTTTTGCTAAATATTCACTTTGAATATTGACGTTAGTGCTATTGATTAATTTAAAACAATTATGAAGCATTTTTTTTATTATTTTCAAAGTTATGAAAATTTGTATTTATTCTTAAAAAAATACTTACTATAGTCATCAACATAAACTAAACTAATTTATATTTTGAATACAAATTATTATAACGGAAGAATTTAAGATGATAATAGTTCGCTCTTTGTCTCAAATATTTCACGGAAAAAGTGTTTAACATTTTTTACCCTACAGTTAATAAAGATGAATTTAATTATTAGTAATATGTATCTGTGGTGTATCAAATCTATATCTAGTGTAAATGTCTTTATTTTAGGAAGCAGGTTAGAAGTCCTAATTATTTTGTCAATTCTATTTATGTTAAATTGATTAACAACCTAAAATCACCTCTTGAAATTAAGTATTTAGTGAAAAACAATCTAGGTTGATAGGTATATAAGAGTAAATTAGATGAAAATATGAAACAAAAATAAAAATTGAAGATAACTTGATAACATTTTGAAACTTAAATCAATGGAAAAAATTTCAAAAATCAAAGATAGTGTAATATTTTCAAACCTTAGTATTAAAAAATGTACCTTTTTTTCCTTATTAATTTAGACATTAAGGCGAAAATACAACTGGAAAATGATTTTACCTCAAAAAAGAAAAAAATAAGAAAAAAGAAAAAAGAAAAAAAATTGACGGAAACAAAATATATTCAGTTGGGTGTAGATTAGTAGACAATGAAAATCTCGCAGGAAACACGAAATTTTCTCTCGCTCTCTCTCTCTCTCTCTCAAAACCCTATCAATTAAGAAAAACATCCCCCTCTTTAATTCATCACCATTACGAATACCAATTCACTCTCAAATCCCACTGATCTTCACCATAATTCCTTTCTCAATTCTTTCTTCCTTCCAAACACACAACAATGAAGAGAAAATCAATGTTGGAAAAAGAAGCAGCCCAAGTAGAAGACCGGTCATCGGAGTTCAATAATGGAATCCAATGGAAACAAGGTCGGCTCATCGGAAAAGGAAGCTTTGGATCAGTTTTCTTGGCTTCTCTCAAACCACACTTCACTAAATACAGCATTTTCCCTCCCGTAATGGCTGTTAAGTCCGCTGAAATTTCCGTTTCCGAAACCCTCCAAAAGGAAAAGCAAAACTACGATAACTTGAAAGGATGCAATTCCTTGATCCAATGCTTCGGCGAAGAGATTACTACTGACCATAACGGTCACATGATCTATAATTTGCTGCTTGAAGTTGCCACTGGAGGAACCCTAGCTCACCATATTAAAAACACTGGTAATTTCATTTATCTTATTAATTTCGTAATTAATCCTTCAATTACTGCATGCCATAATTCTTTATTGGTTTAGAATTGAATTAGGGTTATTGGCTTAGAATT
