# Supplementary material for: Genome-wide identification of MAPK, MAPKK, and MAPKKK gene families and transcriptional profiling analysis during development and stress response in cucumber
Source: BMC Genomics. 2015 May 15;16(1):386. doi: 10.1186/s12864-015-1621-2 (PMC4432876; doi:10.1186/s12864-015-1621-2)

**Additional file 1.**

**Sequence alignment analysis of the CsMAPK cascade genes with two or more copies.** A: Alignment analysis of the nucleotide sequences of the CsMAPK cascade genes with two or more copies. B: Alignment analysis of the peptides sequence of the CsMAPK cascade genes with two or more copies.

A


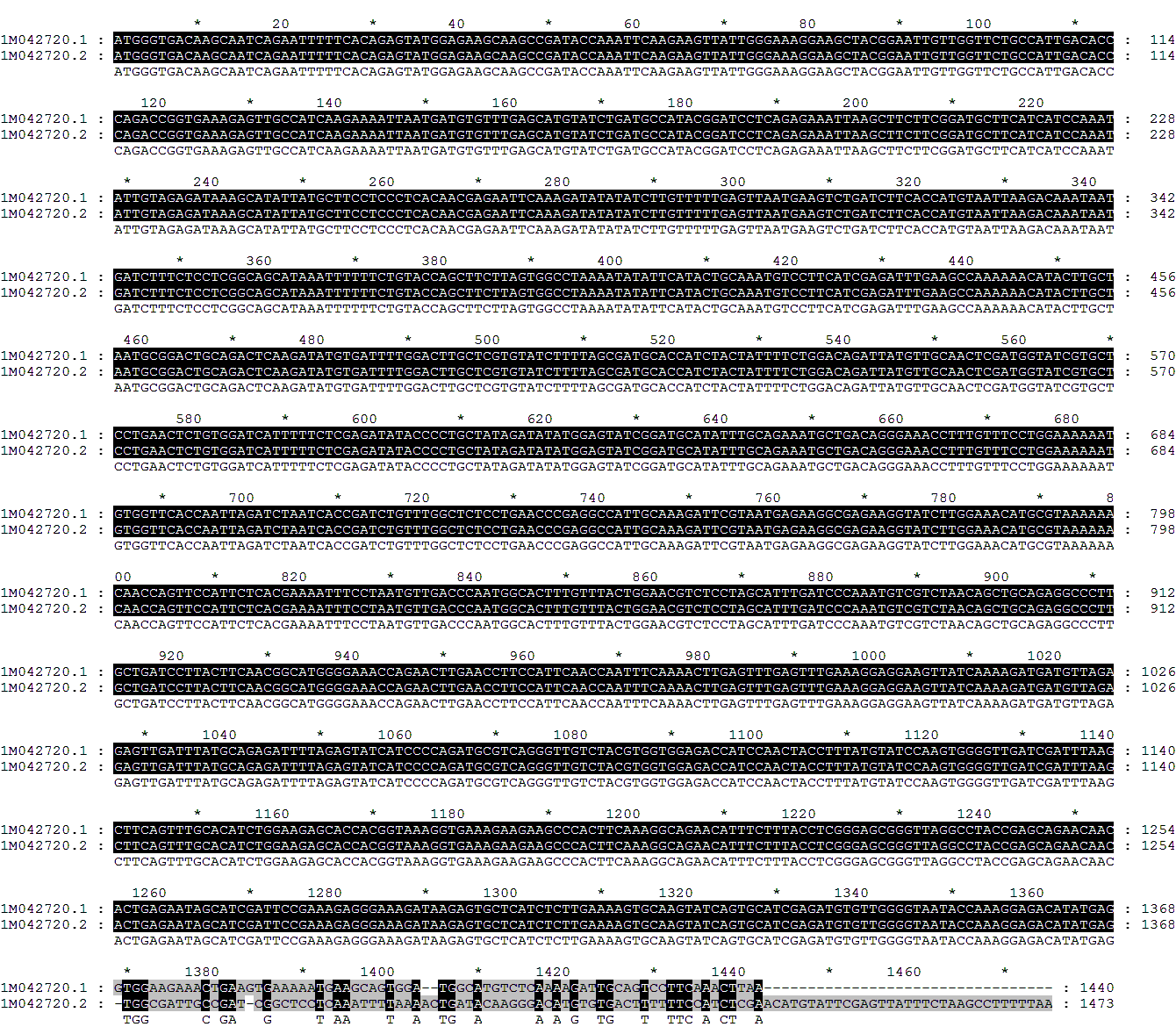


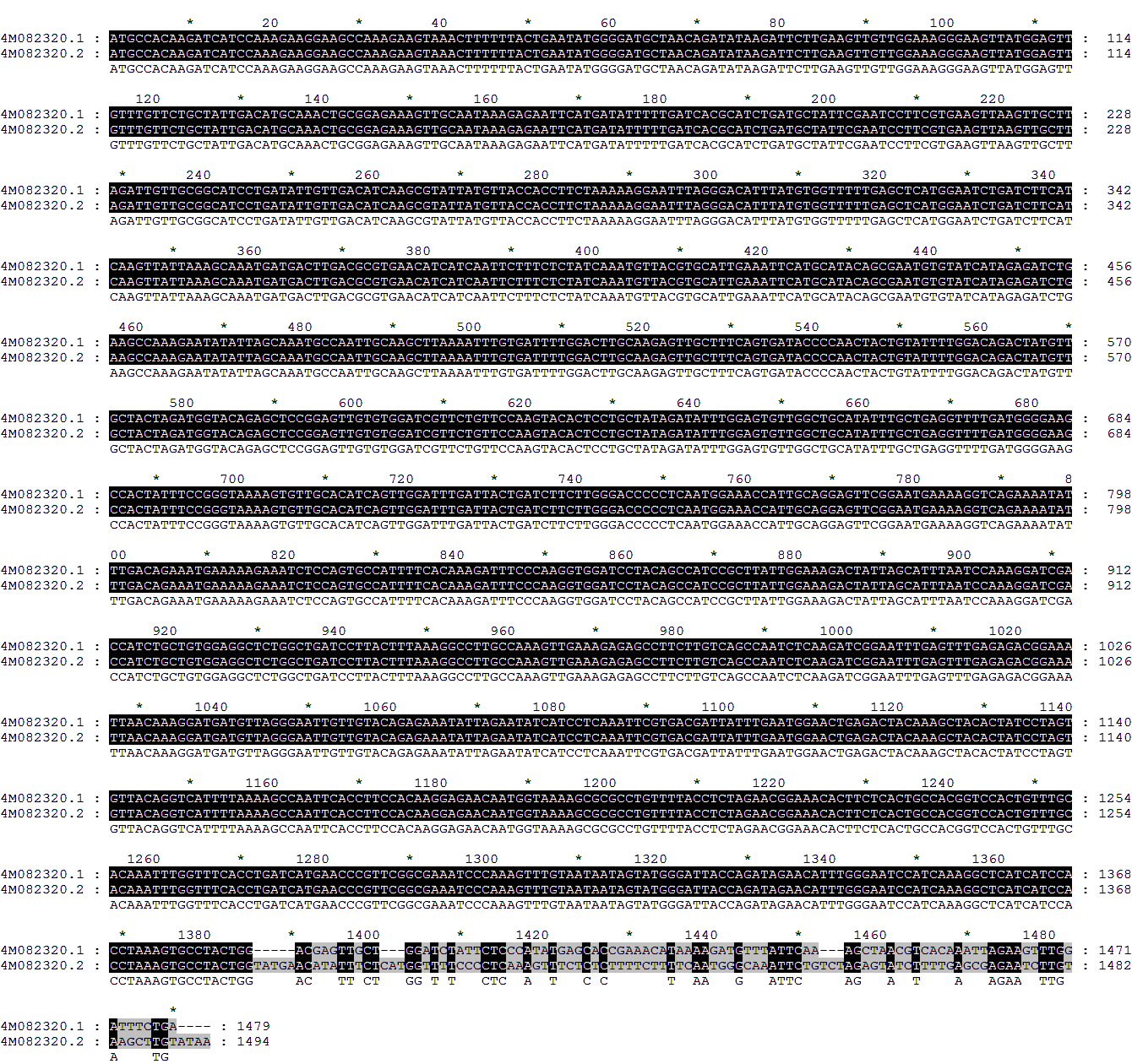


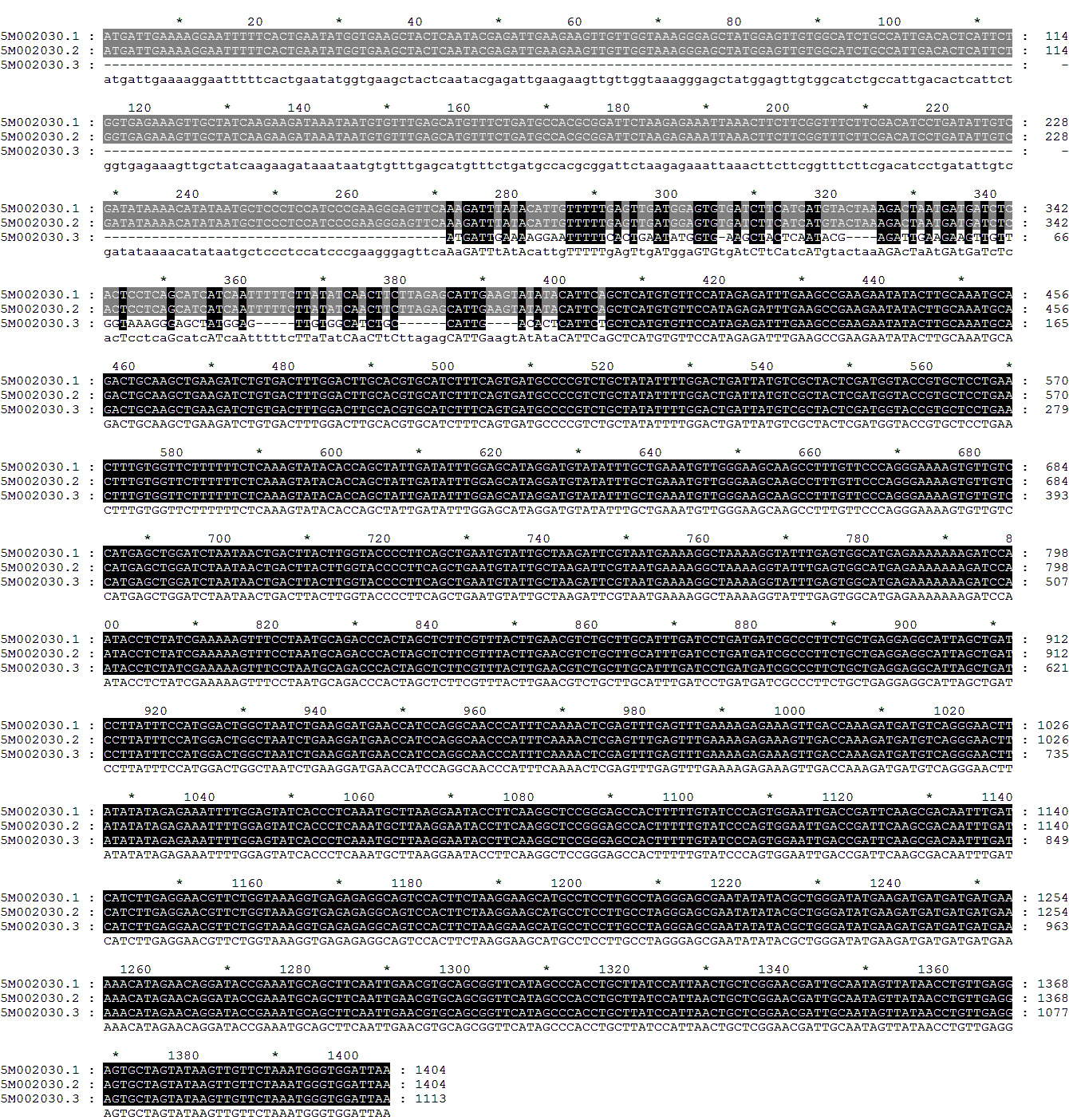


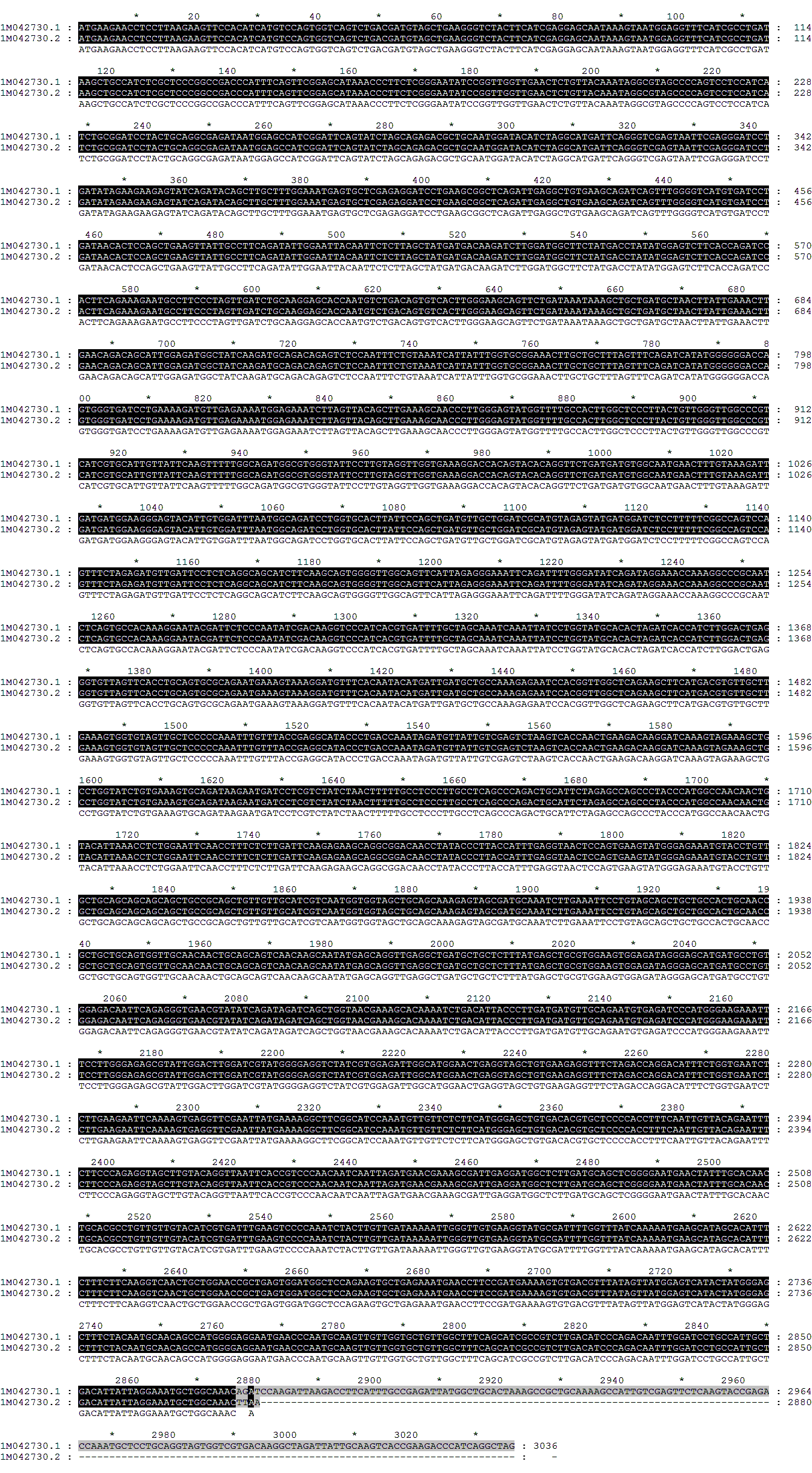


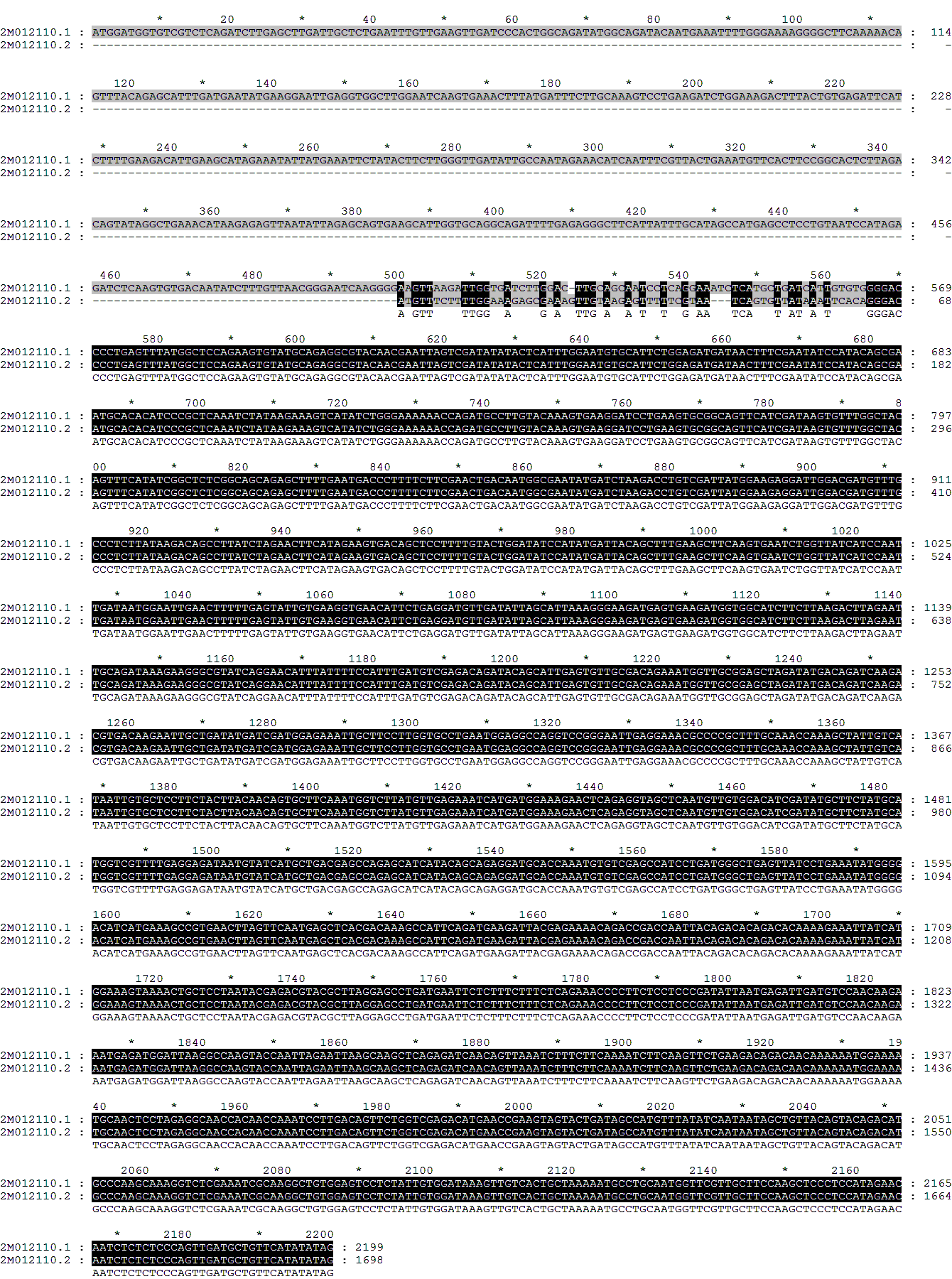


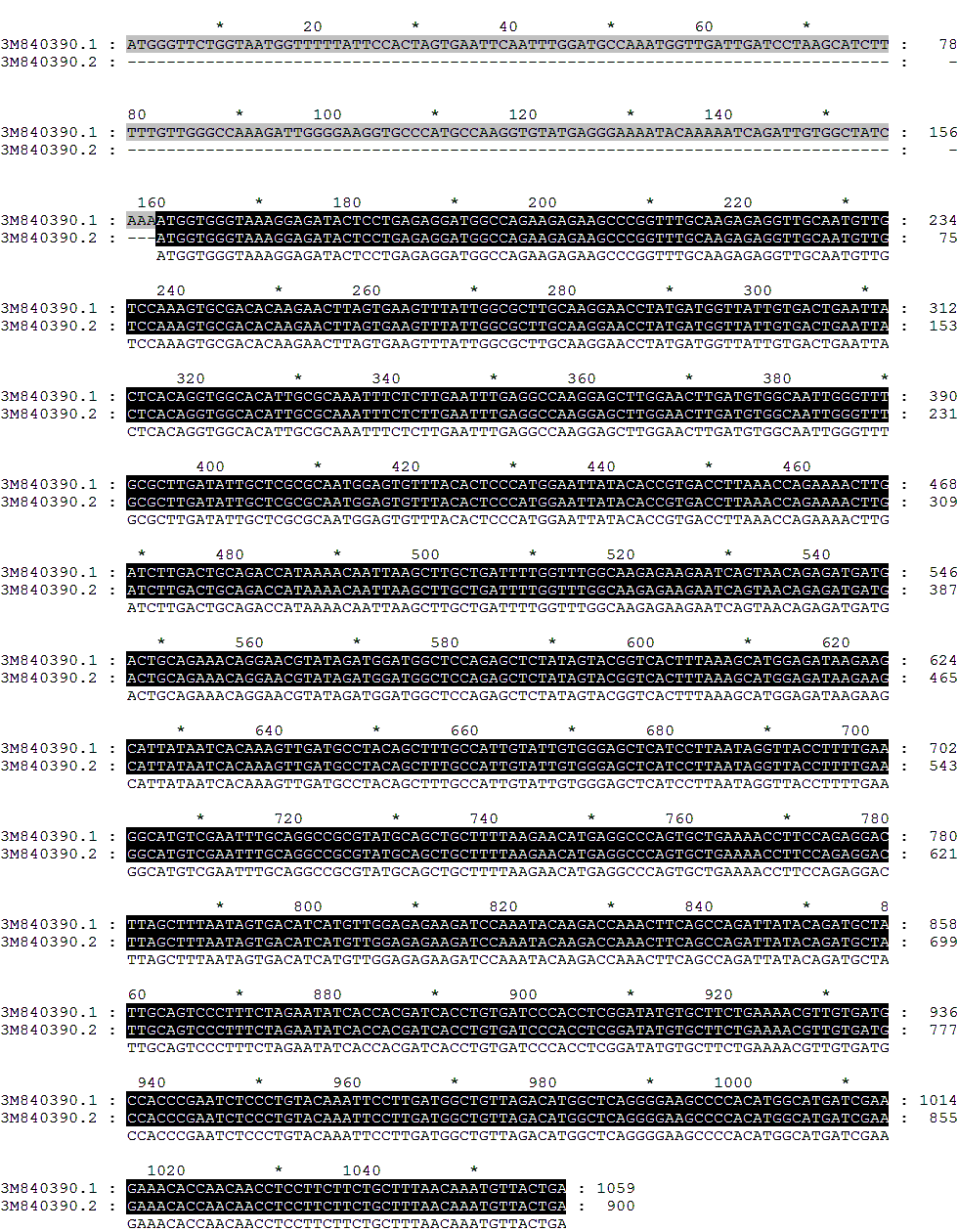


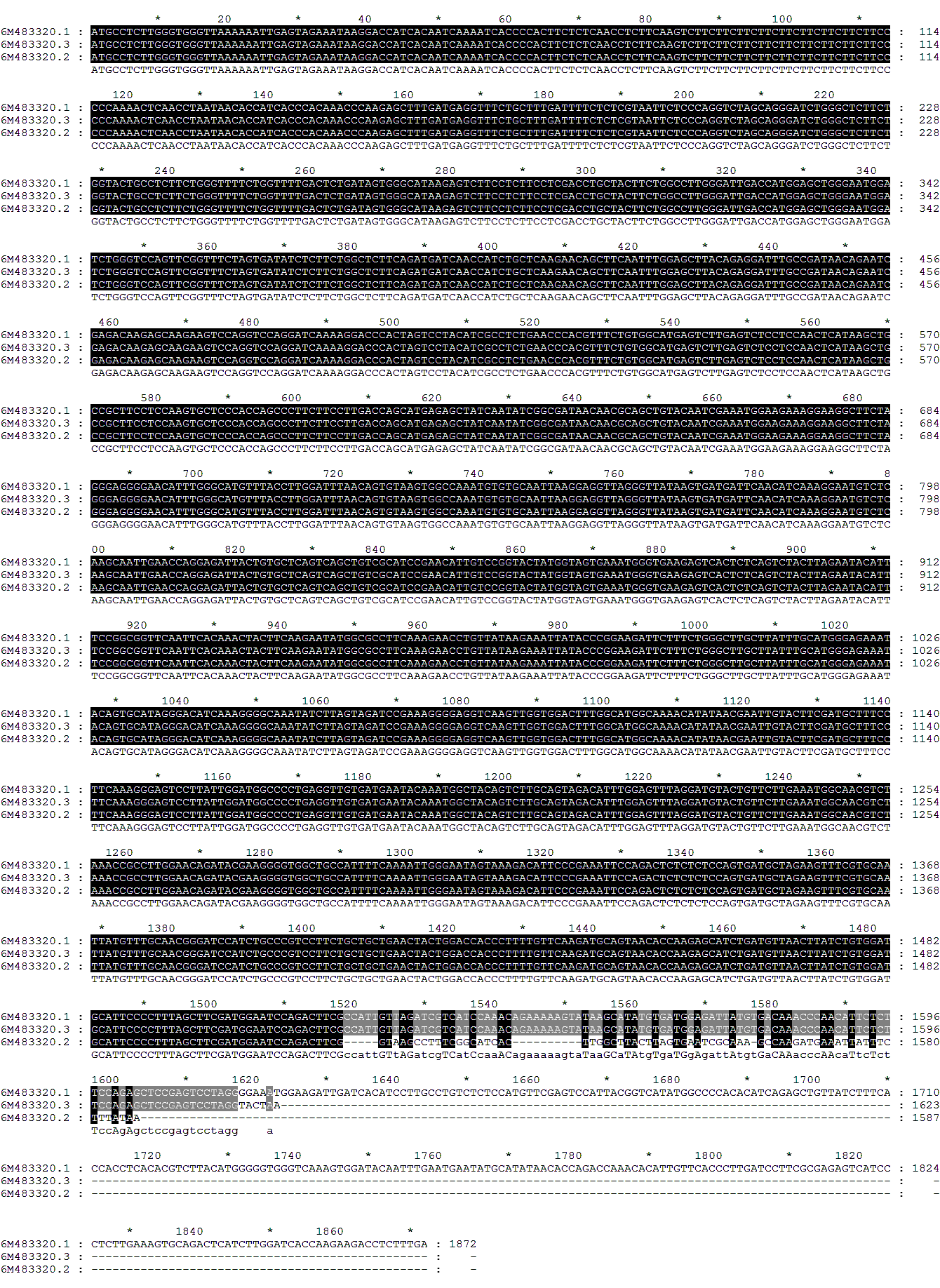


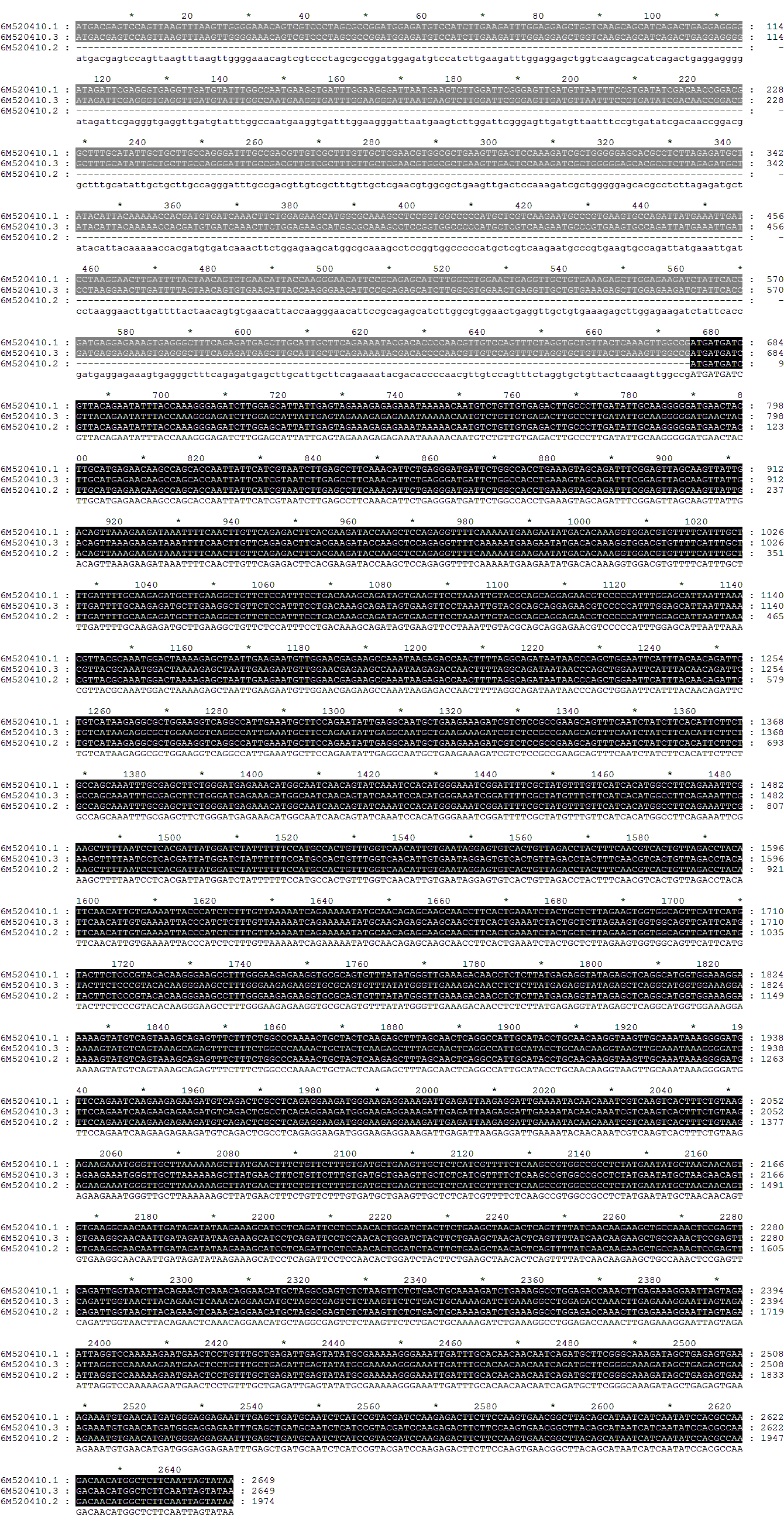


**B**


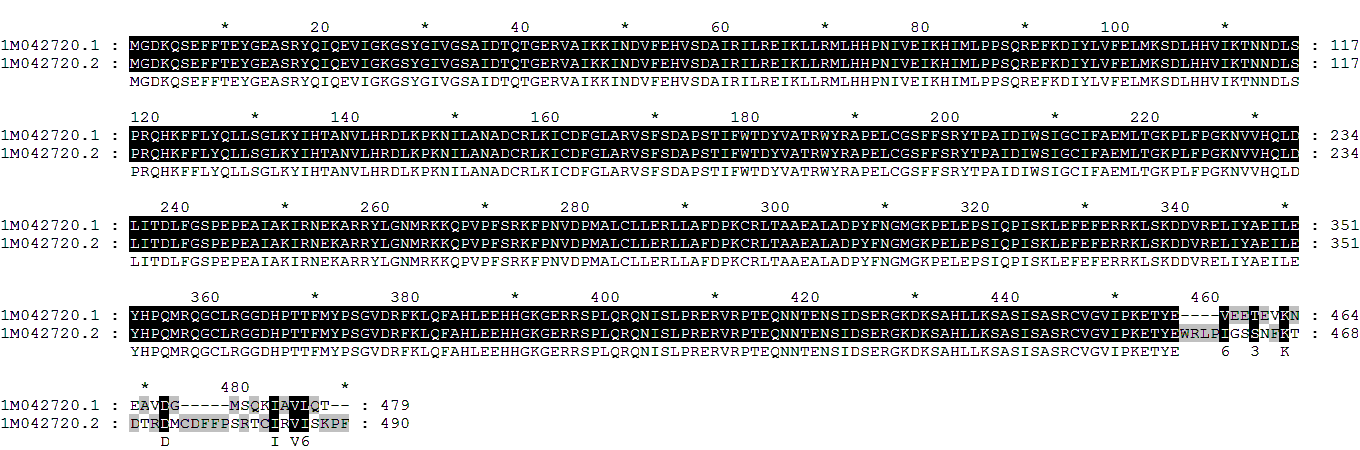


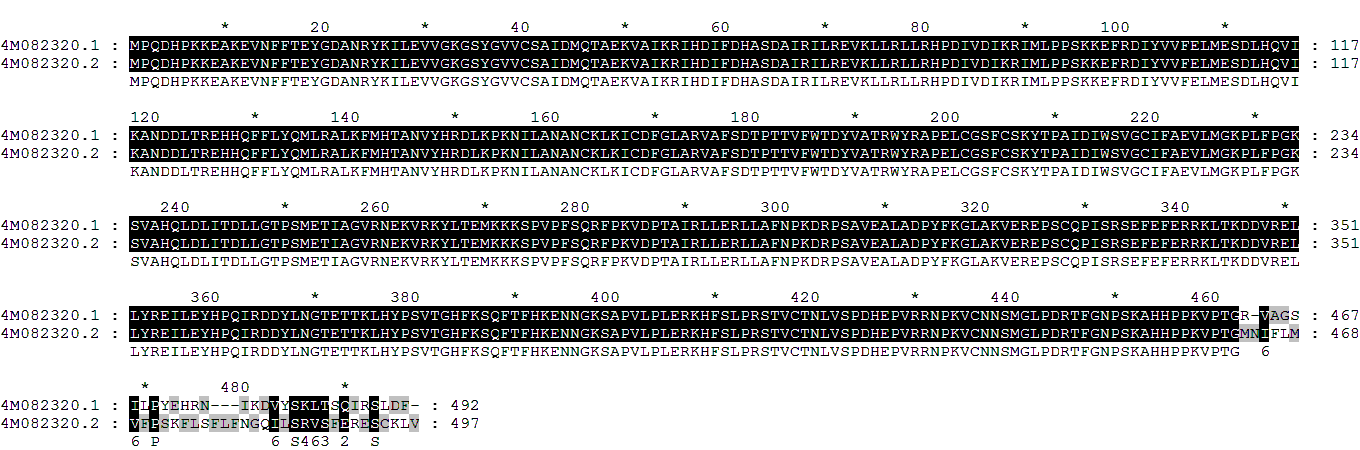


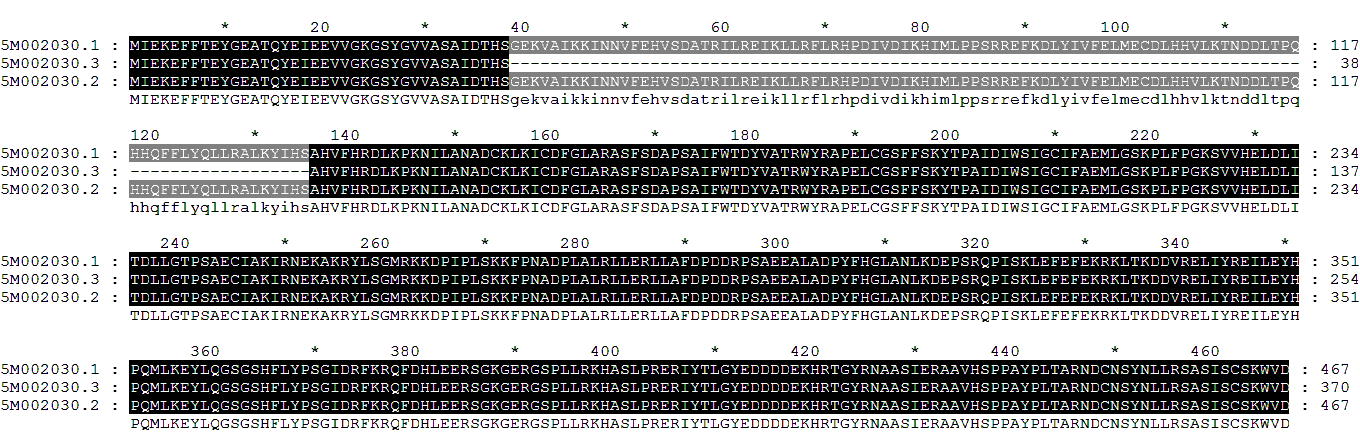


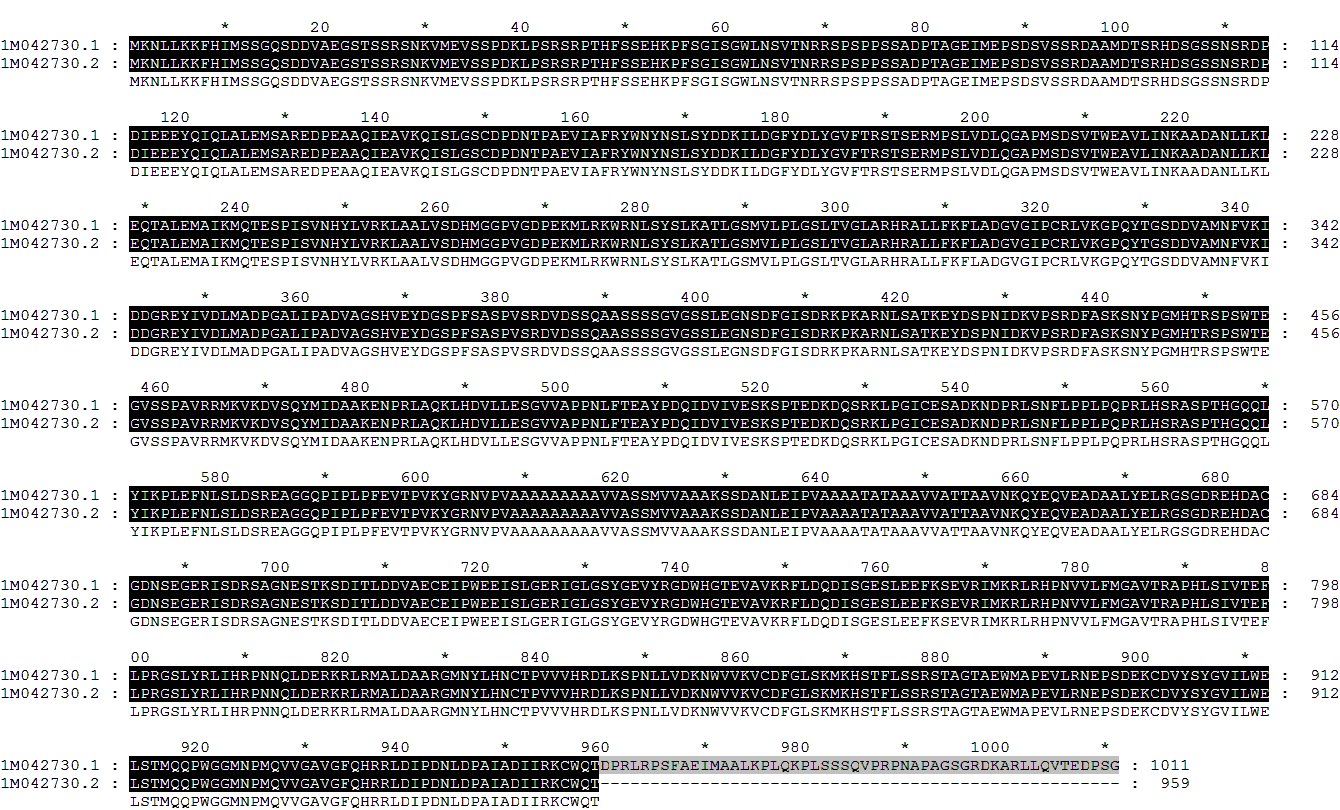


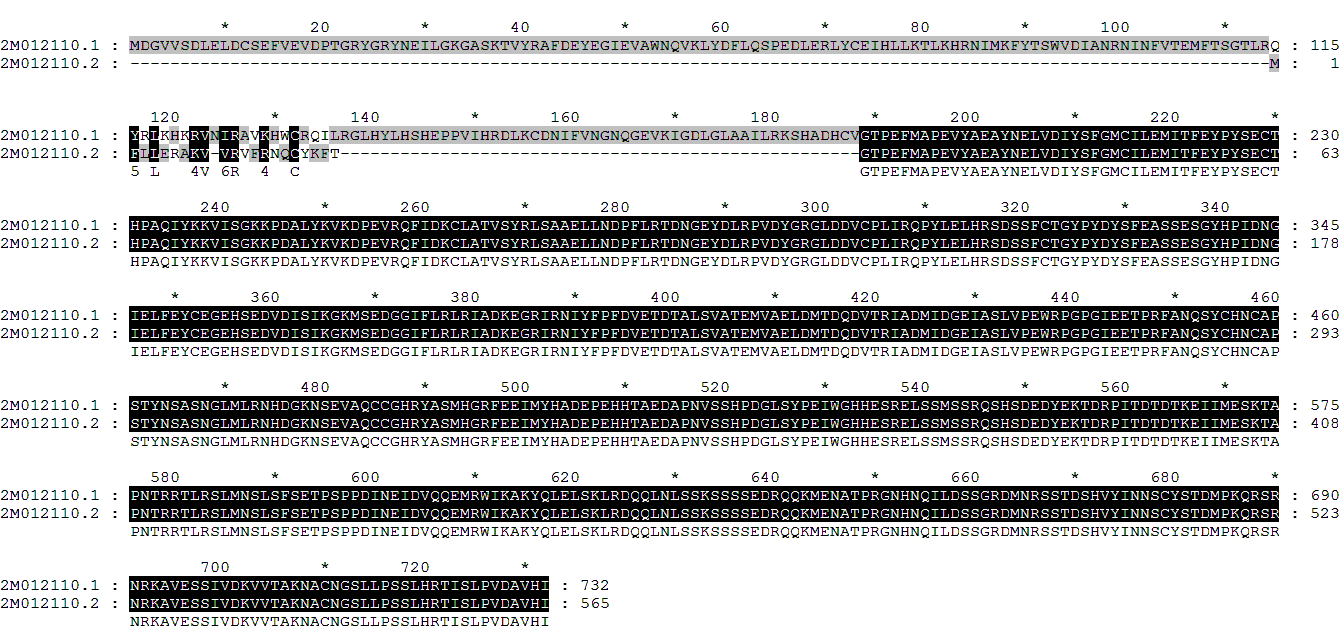


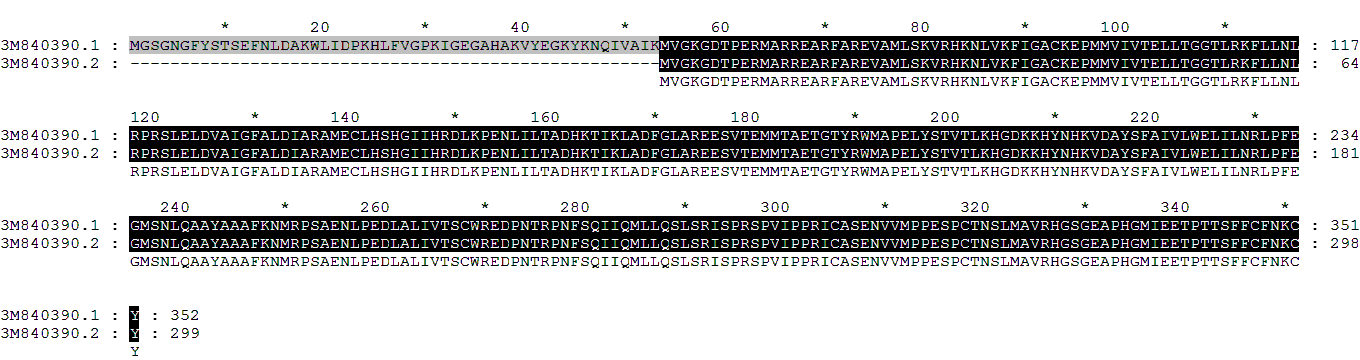


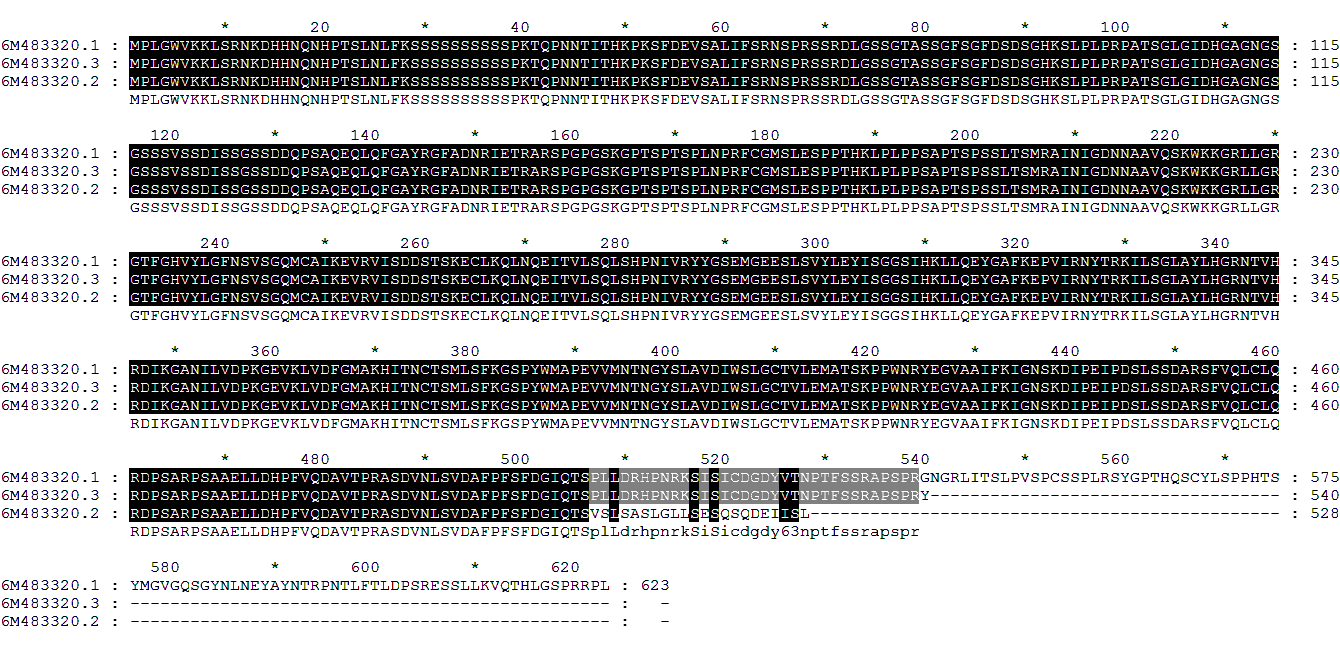


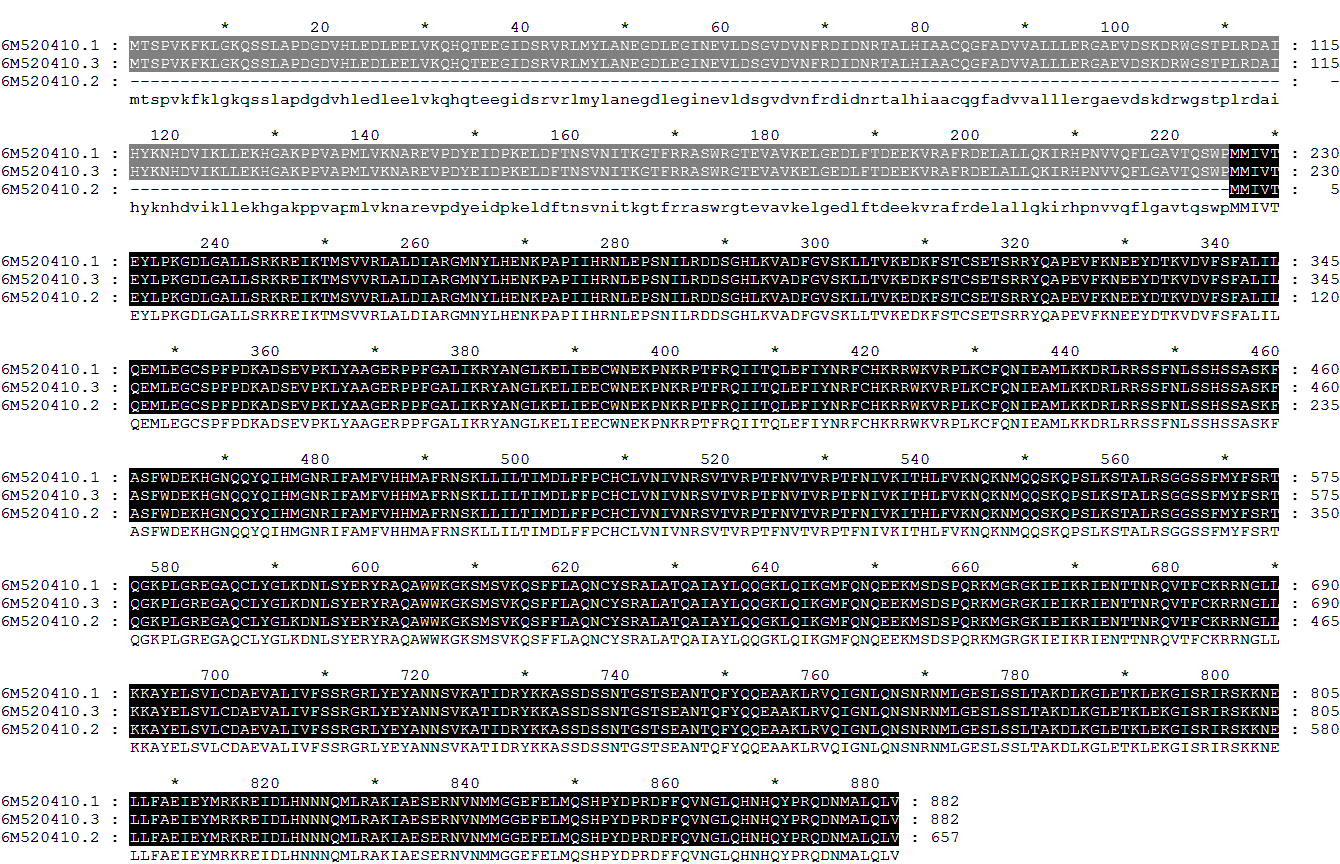

Supplement: Additional file 2: — Sequence alignment analysis of the CsMAPK cascade genes with two or more copies. A: Alignment analysis of the nucleotide sequences of the CsMAPK cascade genes with two or more copies. B: Alignment analysis of the peptides sequence of the CsMAPK cascade genes with two or more copies. [file 12864_2015_1621_MOESM2_ESM.doc]
