# Supplementary material for: Genome-wide identification of MAPK, MAPKK, and MAPKKK gene families and transcriptional profiling analysis during development and stress response in cucumber
Source: BMC Genomics. 2015 May 15;16(1):386. doi: 10.1186/s12864-015-1621-2 (PMC4432876; doi:10.1186/s12864-015-1621-2)
Supplement: Additional file 3: — Classification and summary of the functions or predicted fuctions of the cucumber MAPK cascade gene families. The putative functions of cucumber MAPK cascade genes were predicted based on the experimentally characterized homologues from Arabidopsis. [file 12864_2015_1621_MOESM3_ESM.doc]

**Additional file 2**. Classification and summary of the functions or predicted fuctions of the cucumber MAPK cascade gene families.

| **Clade** | **CsMPK** | **Gene** | **Synonyms** | **Genebank Accession** | **(Putative) Function** | **Ref** |
| --- | --- | --- | --- | --- | --- | --- |
| C | 1 | Csa2M361890.1 | CsMAPK1 | FJ036898 | stress tolerance; defense response | S1,S2 |
| A | 3 | Csa1M479630.1 | CsTIPK/CsNMAPK | NM_001280724 | stress response | S3-S5 |
| B | 4-1 | Csa5M152810.1 |  |  | PREDICTED：defense signaling; ROS signaling; abiotic and biotic stress response; cytokinesis; microtubule organization | S6-S12 |
| B | 4-2 | Csa6M006730.1 |  |  |
| A | 6 | Csa6M365750.1 |  |  | PREDICTED：defense signaling; abiotic and biotic stress response; seed formation; root development; ovule development; anther, inflorescence and embryo development; stomata development and patterning; leaf senescence; floral organ abscission; cell death; ethylene signaling; JA signaling | S13-S32 |
| C | 7 | Csa4M045070.1 |  |  |  |  |
| D | 9-1 | Csa1M024990.1 |  |  | PREDICTED：ABA/Chitosan/Yeast elicitor-induced stomatal closure; ROS-mediated ABA signaling | S33-S35 |
| D | 9-2 | Csa5M002030.1 |  |  |
| D | 9-3 | Csa1M042720.2 |  |  |
| B | 13 | Csa1M077220.1 |  |  | PREDICTED：lateral root formation | S36 |
| D | 16 | Csa6M061230.1 |  |  |  |  |
| D | 19 | Csa4M082320.2 |  |  |  |  |
| D | 20-1 | Csa6M179480.1 |  |  |  |  |
| D | 20-2 | Csa6M423420.1 |  |  |  |  |
| **Clade** | **CsMPKK** | **Gene** | **Synonyms** |  | **(Putative) Function** | **Ref** |
| A | 2-1 | Csa1M589750.1 |  |  | PREDICTED：defense response;abiotic and biotic stress response; | S9, S11, S37-S38 |
| A | 2-2 | Csa2M000340.1 |  |  |
| B | 3 | Csa3M839800.1 |  |  | PREDICTED：pathogen signaling; JA signaling; blue light-mediated seedling development | S20, S28, S39 |
| C | 4 | Csa3M651720.1 |  |  | PREDICTED：defense response; inflorescence architecture; floral organ abscission; | S29-31, S40-S41 |
| A | 6 | Csa2M000780.1 |  |  | PREDICTED：lateral root formation; cytokinesis | S6, S36 |
| D | 9 | Csa1M042980.1 |  |  | PREDICTED：leaf Senescence; ethylene and camalexin biosynthesis; sress respose | S18, S42 |
| **Clade** | **CsMEKK** | **Gene** | **Synonyms** |  | **(Putative) Function** | **Ref** |
| MEKK | 1 | Csa2M021750.1 |  |  | PREDICTED： cytokinesis; elicitor-induced oxidative burst and immunity | S6, S43 |
| MEKK | 3 | Csa6M483320.1 | CsMAP3Ka | FJ036902 | stress tolerance; defense response |  |
| MEKK | 4-1 | Csa3M182770.1 |  |  | PREDICTED：inflorescence architecture; stomatal development and patterning; extra-embryonic suspensor differentiation; root cell division plane orientation | S29-S32, S44 |
| MEKK | 4-2 | Csa5M166980.1 |  |  |
| MEKK | 5-1 | Csa6M490220.1 |  |  |  |  |
| MEKK | 5-2 | Csa2M360650.1 |  |  |  |  |
| MEKK | 8 | Csa5M385380.1 |  |  | PREDICTED：defense signaling; ROS signaling; stress response; | S7, S9, S11 |
| MEKK | 12 | Csa6M425140.1 |  |  |  |  |
| MEKK | 13 | Csa1M532310.1 |  |  |  |  |
| MEKK | 15 | Csa6M513560.1 |  |  |  |  |
| MEKK | 17-1 | Csa2M416770.1 |  |  |  |  |
| MEKK | 17-2 | Csa7M043040.1 |  |  |  |  |
| MEKK | 20 | Csa7M430790.1 |  |  |  |  |
| MEKK | 21-1 | Csa6M490950.1 |  |  |  |  |
| MEKK | 21-2 | Csa2M278170.1 |  |  |  |  |
| MEKK | 21-3 | Csa7M378450.1 |  |  |  |  |
| MEKK | 21-4 | Csa7M407720.1 |  |  |  |  |
| MEKK | 21-5 | Csa3M829110.1 |  |  |  |  |
| **Clade** | **CsRAF** | **Gene** | **Synonyms** |  | **(Putative) Function** | **Ref** |
| RAF | 1-1 | Csa6M450400.1 | CsCTR1 | JQ277220 | negative regulate in the ethylene signaling pathway | S45 |
| RAF | 1-2 | Csa3M749850.1 |  |  | PREDICTED: ethylene signaling | S45 |
| RAF | 2 | Csa1M574260.1 |  |  | PREDICTED: plant innate immunity; plant disease resistance, stress responses, cell death, and ethylene signaling | S41, S46-47 |
| RAF | 3 | Csa4M646020.1 |  |  |  |  |
| RAF | 4 | Csa1M042730.1 |  |  |  |  |
| RAF | 6 | Csa3M892210.1 |  |  |  |  |
| RAF | 10 | Csa6M330990.1 |  |  |  |  |
| RAF | 15 | Csa6M154510.1 |  |  |  |  |
| RAF | 16 | Csa3M133150.1 |  |  |  |  |
| RAF | 18 | Csa6M136540.1 |  |  |  |  |
| RAF | 19-1 | Csa6M511830.1 |  |  |  |  |
| RAF | 19-2 | Csa1M046040.1 |  |  |  |  |
| RAF | 22 | Csa2M070870.1 |  |  |  |  |
| RAF | 24 | Csa1M057040.1 |  |  |  |  |
| RAF | 25 | Csa7M051390.1 |  |  |  |  |
| RAF | 27 | Csa1M467120.1 |  |  |  |  |
| RAF | 29 | Csa3M002480.1 |  |  |  |  |
| RAF | 30-1 | Csa7M017160.1 |  |  |  |  |
| RAF | 30-2 | Csa6M058190.1 |  |  |  |  |
| RAF | 31 | Csa3M728150.1 |  |  |  |  |
| RAF | 34 | Csa1M003510.1 |  |  |  |  |
| RAF | 35 | Csa2M049880.1 |  |  |  |  |
| RAF | 36-1 | Csa6M517390.1 |  |  |  |  |
| RAF | 36-2 | Csa1M074900.1 |  |  |  |  |
| RAF | 37 | Csa6M502000.1 |  |  |  |  |
| RAF | 38 | Csa3M836460.1 |  |  |  |  |
| RAF | 39-1 | Csa7M387170.1 |  |  |  |  |
| RAF | 39-2 | Csa3M146410.1 |  |  |  |  |
| RAF | 41-1 | Csa3M840390.1 |  |  |  |  |
| RAF | 41-2 | Csa5M523010.1 |  |  |  |  |
| RAF | 47 | Csa6M520410.1 |  |  |  |  |
| **Clade** | **CsZIK** | **Gene** | **Synonyms** |  | **(Putative) Function** | **Ref** |
| ZIK | 1 | Csa4M332110.1 |  |  |  |  |
| ZIK | 2 | Csa6M212860.1 |  |  |  |  |
| ZIK | 4-1 | Csa2M012110.1 |  |  | PREDICTED: abiotic stress; internal circadian rhythm | S48 |
| ZIK | 4-2 | Csa7M234730.1 |  |  |
| ZIK | 4-3 | Csa1M695390.1 |  |  |
| ZIK | 5 | Csa3M119370.1 |  |  |  |  |
| ZIK | 6 | Csa5M148620.1 |  |  |  |  |
| ZIK | 8-1 | Csa3M062560.1 |  |  |  |  |
| ZIK | 8-2 | Csa6M110320.1 |  |  |  |  |
| ZIK | 11 | Csa1M046910.1 |  |  |  |  |

Note: The putative functions of cucumber MAPK cascade genes were predicted based on the experimentally characterized homologues from *Arabidopsis*.

**Supplementary references**

S1. Xia XJ, Wang YJ, Zhou YH, Tao Y, Mao WH, Shi K, Asami T, Chen Z, Yu JQ: **Reactive oxygen species are involved in brassinosteroid-induced stress tolerance in cucumber**. *Plant Physiol* 2009, **150**(2):801-814.

S2. Zhou J, Xia XJ, Zhou YH, Shi K, Chen ZX, Yu JQ: **RBOH1-dependent H2O2 production and subsequent activation of MPK1/2 play an important role in acclimation-induced cross-tolerance in tomato**. *J exp bot* 2014, **65**(2):595-607.

S3. Xu HN, Wang XF, Sun XD, Shi QH, Yang FJ, Du DL: **Molecular cloning and characterization of a cucumber MAP kinase gene in response to excess NO_3_^-^ and other abiotic stresses**. *Sci Hortic* 2008, **117**(1):1-8.

S4. Xu HN, Sun XD, Wang XF, Shi QH, Yang XY, Yang FJ: **Involvement of a cucumber MAPK gene (CsNMAPK) in positive regulation of ROS scavengence and osmotic adjustment under salt stress**. *Sci Hortic* 2011, **127**(4):488-493.

S5. Shoresh M, Gal-On A, Leibman D, Chet I: **Characterization of a mitogen-activated protein kinase gene from cucumber required for Trichoderma-conferred plant resistance**. *Plant physiol* 2006, **142**(3):1169-1179.

S6. Takahashi Y, Soyano T, Kosetsu K, Sasabe M, Machida Y: **HINKEL kinesin, ANP MAPKKKs and MKK6/ANQ MAPKK, which phosphorylates and activates MPK4 MAPK, constitute a pathway that is required for** **cytokinesis in Arabidopsis thaliana**. *Plant Cell Physiol* 2010, **51**(10):1766-1776.

S7. Pitzschke A, Djamei A, Bitton F, Hirt H: **A Major Role of the MEKK1-MKK1/2-MPK4 Pathway in ROS Signalling**. *Mol Plant* 2009, **2**(1):120-137.

S8. Kosetsu K, Matsunaga S, Nakagami H, Colcombet J, Sasabe M, Soyano T, Takahashi Y, Hirt H, Machida Y: **The MAP Kinase MPK4 Is Required for Cytokinesis in Arabidopsis thaliana**. *Plant cell* 2010, **22**(11):3778-3790.

S9. Gao MH, Liu JM, Bi DL, Zhang ZB, Cheng F, Chen SF, Zhang YL: **MEKK1, MKK1/MKK2 and MPK4 function together in a mitogen-activated protein kinase cascade to regulate innate immunity in plants**. *Cell Res* 2008, **18**(12):1190-1198.

S10. Wang FZ, Jing W, Zhang WH: **The mitogen-activated protein kinase cascade MKK1-MPK4 mediates salt signaling in rice**. *Plant Sci* 2014, **227**:181-189.

S11. Furuya T, Matsuoka D, Nanmori T: **Membrane rigidification functions upstream of the MEKK1-MKK2-MPK4 cascade during cold acclimation in Arabidopsis thaliana**. *FEBS lett* 2014, **588**(11):2025-2030.

S12. Beck M, Komis G, Muller J, Menzel D, Samaj J: **Arabidopsis Homologs of Nucleus- and Phragmoplast-Localized Kinase 2 and 3 and Mitogen-Activated Protein Kinase 4 Are Essential for** **Microtubule Organization**. *Plant cell* 2010, **22**(3):755-771.

S13. Wankhede DP, Kumar K, Singh P, Sinha AK: **Involvement of mitogen activated protein kinase kinase 6 in UV induced transcripts accumulation of genes in phytoalexin biosynthesis in rice**. *Rice* 2013, **6**.

S14. Lopez-Bucio JS, Dubrovsky JG, Raya-Gonzalez J, Ugartechea-Chirino Y, Lopez-Bucio J, de Luna-Valdez LA, Ramos-Vega M, Leon P, Guevara-Garcia AA: **Arabidopsis thaliana mitogen-activated protein kinase 6 is involved in seed formation and modulation of primary and lateral root development**. *J exp bot* 2014, **65**(1):169-183.

S15. Kumar K, Sinha AK: **Overexpression of constitutively active mitogen activated protein kinase kinase 6 enhances tolerance to salt stress in rice**. *Rice* 2013, **6**.

S16. Han L, Li GJ, Yang KY, Mao GH, Wang RQ, Liu YD, Zhang SQ: **Mitogen-activated protein kinase 3 and 6 regulate Botrytis cinerea-induced** **ethylene production in Arabidopsis**. *Plant J* 2010, **64**(1):114-127.

S17. Beckers GJM, Jaskiewicz M, Liu YD, Underwood WR, He SY, Zhang SQ, Conrath U: **Mitogen-Activated Protein Kinases 3 and 6 Are Required for Full Priming of Stress Responses in Arabidopsis thaliana**. *Plant cell* 2009, **21**(3):944-953.

S18. Zhou CJ, Cai ZH, Guo YF, Gan SS: **An Arabidopsis Mitogen-Activated Protein Kinase Cascade, MKK9-MPK6, Plays a Role in Leaf Senescence**. *Plant physiol* 2009, **150**(1):167-177.

S19. Wang HC, Liu YD, Bruffett K, Lee J, Hause G, Walker JC, Zhang SQ: **Haplo-insufficiency of MPK3 in MPK6 mutant background uncovers a novel function of these two MAPKs in Arabidopsis ovule development**. *Plant cell* 2008, **20**(3):602-613.

S20. Takahashi F, Yoshida R, Ichimura K, Mizoguchi T, Seo S, Yonezawa M, Maruyama K, Yamaguchi-Shinozaki K, Shinozaki K: **The mitogen-activated protein kinase cascade MKK3-MPK6 is an important part of the jasmonate signal transduction pathway in Arabidopsis**. *Plant cell* 2007, **19**(3):805-818.

S21. Menke FLH, van Pelt JA, Pieterse CMJ, Klessig DF: **Silencing of the mitogen-activated protein kinase MPK6 compromises disease resistance in arabidopsis**. *Plant cell* 2004, **16**(4):897-907.

S22. Liu YD, Zhang SQ: **Phosphorylation of 1-aminocyclopropane-1-carboxylic acid synthase by MPK6, a stress-responsive mitogen-activated protein kinase, induces ethylene biosynthesis in Arabidopsis**. *Plant cell* 2004, **16**(12):3386-3399.

S23. Lee JS, Ellis BE: **Arabidopsis MAPK phosphatase 2 (MKP2) positively regulates oxidative stress tolerance and inactivates the MPK3 and MPK6 MAPKs**. *J Biol Chem* 2007, **282**(34):25020-25029.

S24. Hord CLH, Suna YJ, Pillitteri LJ, Torii KU, Wang HC, Zhang SQ, Ma H: **Regulation of Arabidopsis early anther development by the mitogen-activated protein kinases, MPK3 and MPK6, and the ERECTA and related receptor-like kinases**. *Mol Plant* 2008, **1**(4):645-658.

S25. Galletti R, Ferrari S, De Lorenzo G: **Arabidopsis MPK3 and MPK6 Play Different Roles in Basal and Oligogalacturonide- or Flagellin-Induced Resistance against Botrytis cinerea**. *Plant physiol* 2011, **157**(2):804-814.

S26. Ye Y, Li Z, Xing D: **Nitric oxide promotes MPK6-mediated caspase-3-like activation in cadmium-induced Arabidopsis thaliana programmed cell death**. *Plant Cell Environ* 2013, **36**(1):1-15.

S27. Bush SM, Krysan PJ: **Mutational evidence that the Arabidopsis MAP kinase MPK6 is involved in anther, inflorescence, and embryo development**. *J exp bot* 2007, **58**(8):2181-2191.

S28. Sethi V, Raghuram B, Sinha AK, Chattopadhyay S: **A mitogen-activated protein kinase cascade module, MKK3-MPK6 and MYC2, is involved in** **blue light-mediated seedling development in Arabidopsis**. *Plant Cell* 2014, **26**(8):3343-3357.

S29. Meng X, Wang H, He Y, Liu Y, Walker JC, Torii KU, Zhang S: **A MAPK cascade downstream of ERECTA receptor-like protein kinase regulates Arabidopsis** **inflorescence architecture by promoting localized cell proliferation**. *Plant Cell* 2012, **24**(12):4948-4960.

S30. Khan M, Rozhon W, Bigeard J, Pflieger D, Husar S, Pitzschke A, Teige M, Jonak C, Hirt H, Poppenberger B: **Brassinosteroid-regulated GSK3/Shaggy-like kinases phosphorylate mitogen-activated protein (MAP) kinase kinases, which control stomata development in Arabidopsis thaliana**. *J Biol Chem* 2013, **288**(11):7519-7527.

S31. Cho SK, Larue CT, Chevalier D, Wang H, Jinn TL, Zhang S, Walker JC: **Regulation of floral organ abscission in Arabidopsis thaliana**. *P Natl Acad Sci USA* 2008, **105**(40):15629-15634.

S32. Smekalova V, Luptovciak I, Komis G, Samajova O, Ovecka M, Doskocilova A, Takac T, Vadovic P, Novak O, Pechan T *et al*: **Involvement of YODA and mitogen activated protein kinase 6 in Arabidopsis post-embryogenic root development through auxin up-regulation and cell division plane orientation**. *New phytol* 2014, **203**(4):1175-1193.

S33. Salam MA, Jammes F, Hossain MA, Ye WX, Nakamura Y, Mori IC, Kwak JM, Murata Y: **MAP Kinases, MPK9 and MPK12, Regulate Chitosan-Induced Stomatal Closure**. *Biosci Biotech Bioch* 2012, **76**(9):1785-1787.

S34. Salam MA, Jammes F, Hossain MA, Ye W, Nakamura Y, Mori IC, Kwak JM, Murata Y: **Two guard cell-preferential MAPKs, MPK9 and MPK12, regulate YEL signalling in Arabidopsis guard cells**. *Plant biology* 2013, **15**(3):436-442.

S35. Jammes F, Song C, Shin DJ, Munemasa S, Takeda K, Gu D, Cho D, Lee S, Giordo R, Sritubtim S *et al*: **MAP kinases MPK9 and MPK12 are preferentially expressed in guard cells and positively regulate ROS-mediated ABA signaling**. *P Natl Acad Sci USA* 2009, **106**(48):20520-20525.

S36. Zeng Q, Sritubtim S, Ellis BE: **AtMKK6 and AtMPK13 are required for lateral root formation in Arabidopsis**. *Plant Signal Behav* 2011, **6**(10):1436-1439.

S37. Teige M, Scheikl E, Eulgem T, Doczi F, Ichimura K, Shinozaki K, Dangl JL, Hirt H: **The MKK2 pathway mediates cold and salt stress signaling in Arabidopsis**. *Mol Cell* 2004, **15**(1):141-152.

S38. Brader G, Djamei A, Teige M, Palva ET, Hirt H: **The MAP kinase kinase MKK2 affects disease resistance in Arabidopsis**. *Molecular Plant-Microbe In* 2007, **20**(5):589-596.

S39. Doczi R, Brader G, Pettko-Szandtner A, Rajh I, Djamei A, Pitzschke A, Teige M, Hirt H: **The Arabidopsis mitogen-activated protein kinase kinase MKK3 is upstream of group C mitogen-activated protein kinases and participates in pathogen signaling**. *Plant cell* 2007, **19**(10):3266-3279.

S40. Kim SH, Woo DH, Kim JM, Lee SY, Chung WS, Moon YH: **Arabidopsis MKK4 mediates osmotic-stress response via its regulation of MPK3 activity**. *Biochem Bioph Res Co* 2011, **412**(1):150-154.

S41. Zhao C, Nie H, Shen Q, Zhang S, Lukowitz W, Tang D: **EDR1 physically interacts with MKK4/MKK5 and negatively regulates a MAP kinase cascade to modulate plant innate immunity**. *PLoS Genet* 2014, **10**(5):e1004389.

S42. Xu J, Li Y, Wang Y, Liu H, Lei L, Yang H, Liu G, Ren D: **Activation of MAPK kinase 9 induces ethylene and camalexin biosynthesis and enhances sensitivity to salt stress in Arabidopsis**. *J Biol Chem* 2008, **283**(40):26996-27006.

S43. Savatin DV, Bisceglia NG, Marti L, Fabbri C, Cervone F, De Lorenzo G: **The Arabidopsis NUCLEUS- AND PHRAGMOPLAST-LOCALIZED KINASE1-Related Protein Kinases Are Required for** **Elicitor-Induced Oxidative Burst and Immunity**. *Plant physiol* 2014, **165**(3):1188-1202.

S44. Musielak TJ, Bayer M: **YODA signalling in the early Arabidopsis embryo**. *Biochem Soc t* 2014, **42**:408-412.

S45. Bie B, Sun J, Pan J, He H, Cai R: **Ectopic expression of CsCTR1, a cucumber CTR-like gene, attenuates constitutive ethylene signaling in an Arabidopsis ctr1-1 mutant and expression pattern analysis of CsCTR1 in cucumber (Cucumis sativus)**. *Int j mol sci* 2014, **15**(9):16331-16350.

S46. Shen X, Liu H, Yuan B, Li X, Xu C, Wang S: **OsEDR1 negatively regulates rice bacterial resistance via activation of ethylene biosynthesis**. *Plant cell environ* 2011, **34**(2):179-191.

S47. Tang D, Christiansen KM, Innes RW: **Regulation of plant disease resistance, stress responses, cell death, and ethylene signaling in Arabidopsis by the EDR1 protein kinase**. *Plant Physiol* 2005, **138**(2):1018-1026.

S48. Kumar K, Rao KP, Biswas DK, Sinha AK: **Rice WNK1 is regulated by abiotic stress and involved in internal circadian rhythm**. *Plant Signal Behav* 2011, **6**(3):316-320.
