# Supplementary material for: Genome-wide identification of MAPK, MAPKK, and MAPKKK gene families and transcriptional profiling analysis during development and stress response in cucumber
Source: BMC Genomics. 2015 May 15;16(1):386. doi: 10.1186/s12864-015-1621-2 (PMC4432876; doi:10.1186/s12864-015-1621-2)
Supplement: Additional file 5: — Alignment of multiple cucumber, Arabidopsis and rice MAPK, MAPKK, and MAPKKK domain amino acid sequences. Alignment was performed using ClustalX. The conserved amino acid signature of each subgroup is highlighted in red box. [file 12864_2015_1621_MOESM5_ESM.doc]

**Additional file 4**

**Alignment of multiple cucumber, *Arabidopsis* and rice MAPK, MAPKK, and MAPKKK domain amino acid sequences.**

Group A MAPK


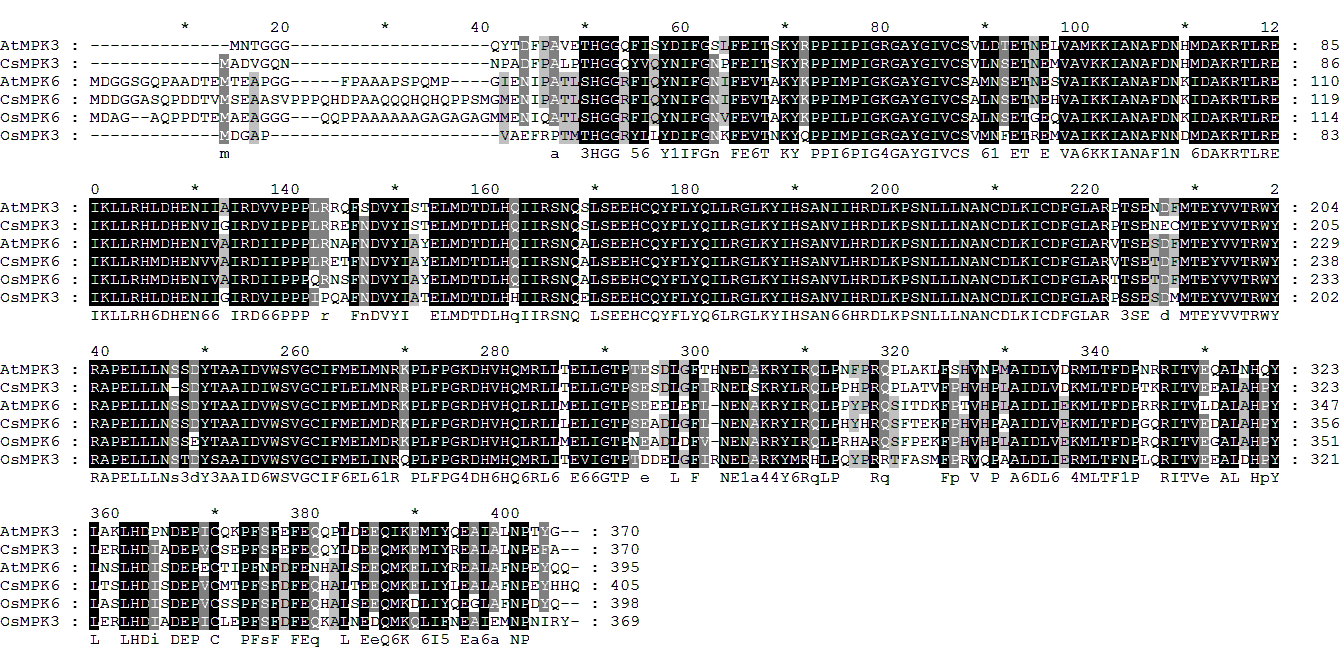


TEY

Group B MAPK


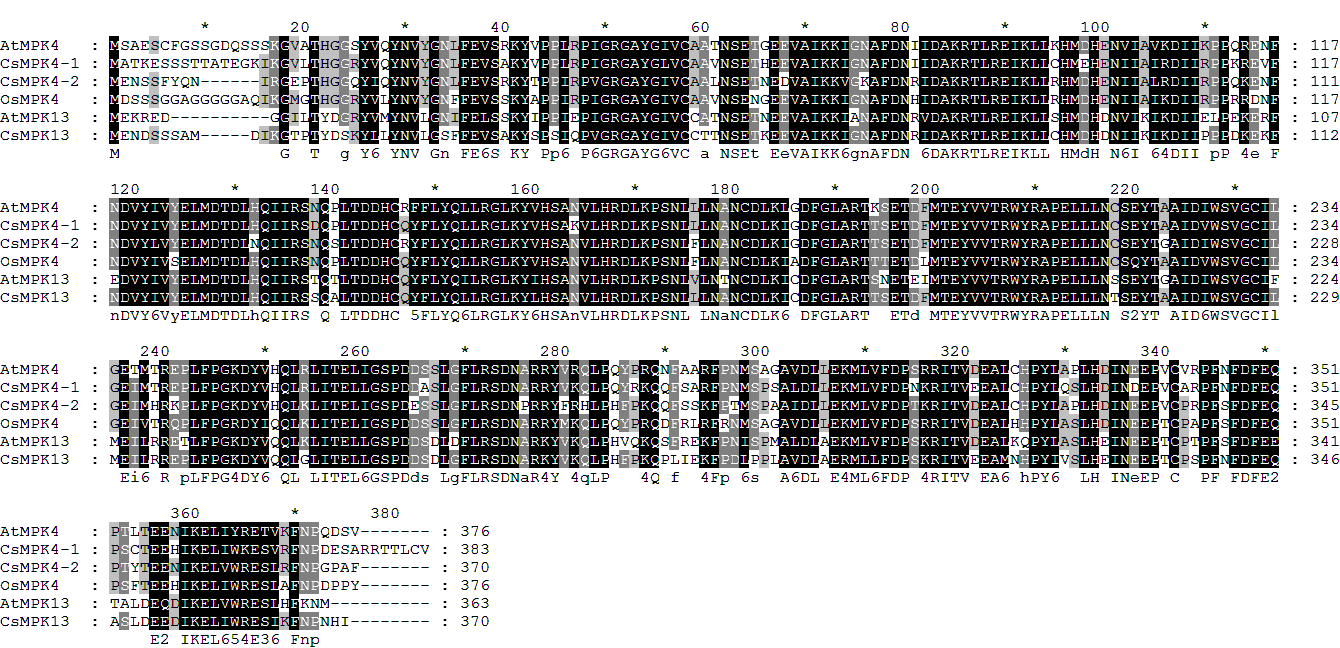


TEY

Group C MAPK


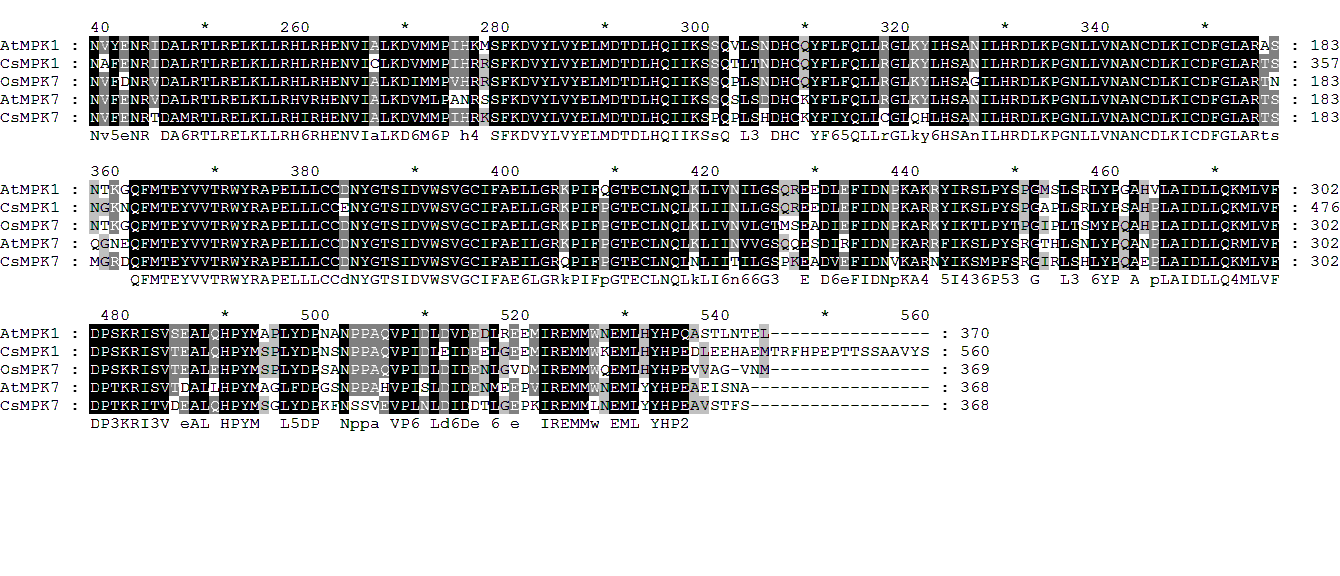


TEY

Group D MAPK


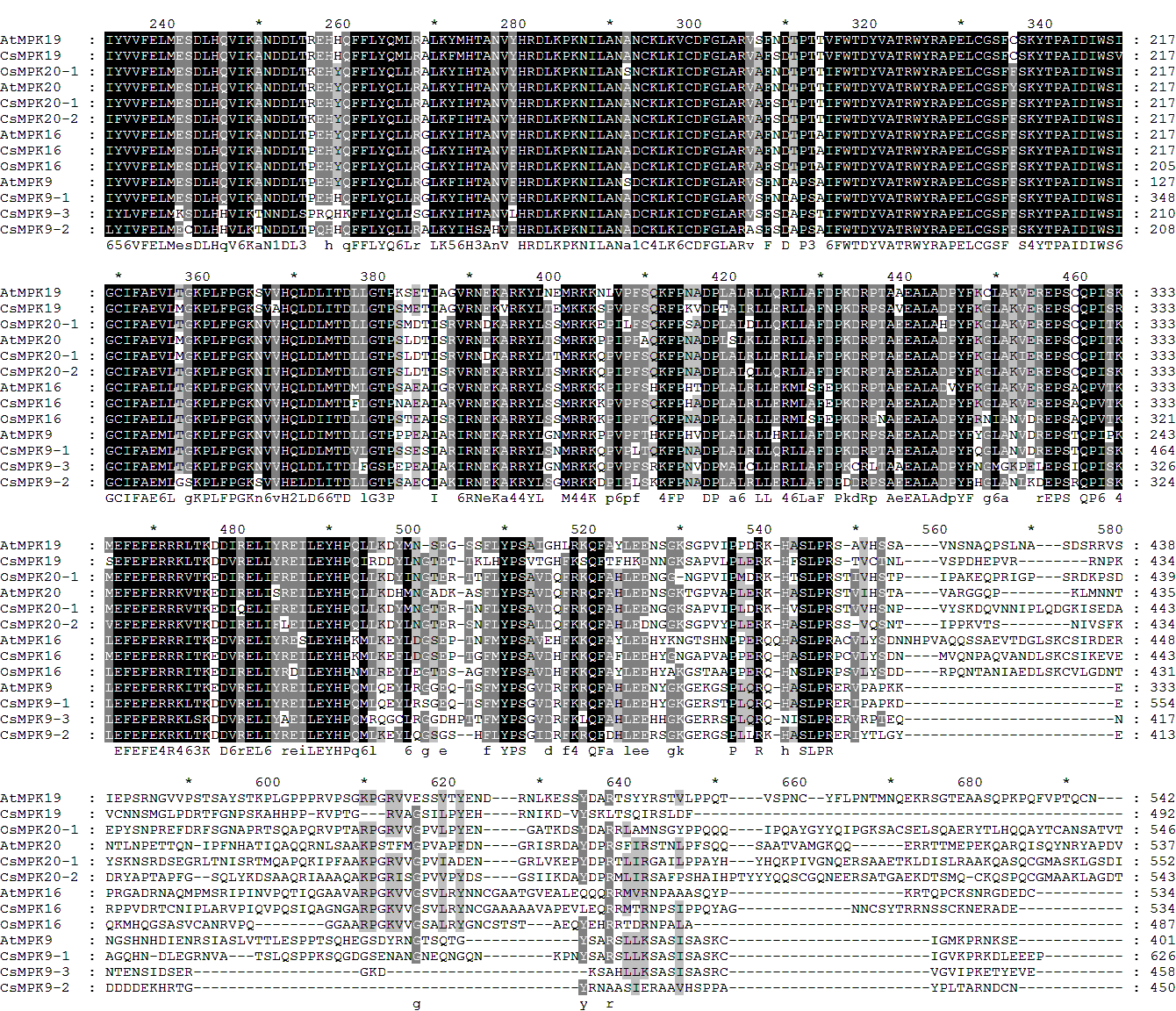


TDY

Group A MAPKK

D(L/I/V)K


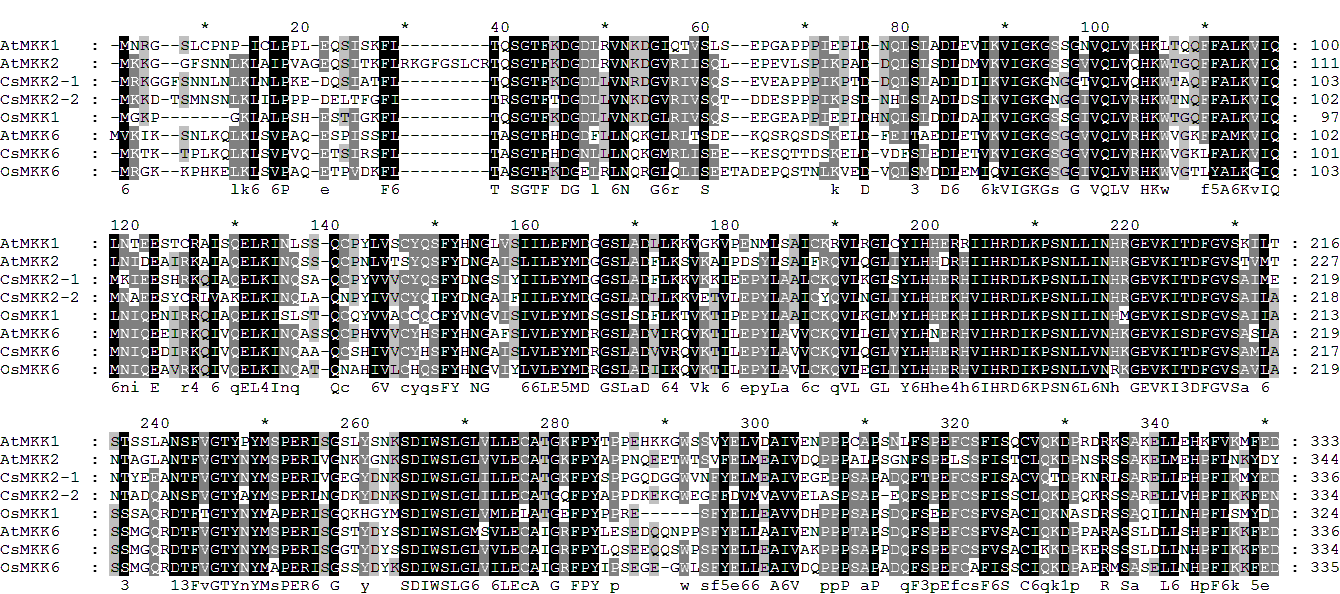


S/T-X5-S/T

D(L/I/V)K

S/T-X5-S/T

Group B MAPKK


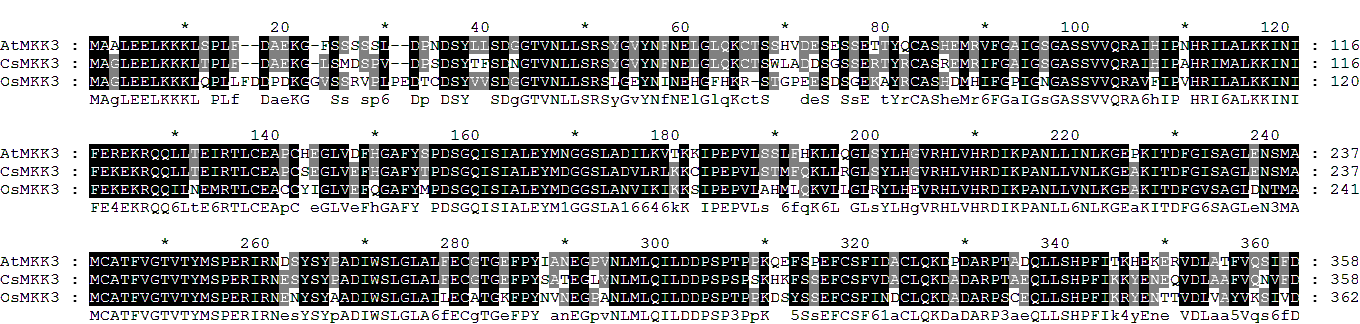


Group C MAPKK

D(L/I/V)K


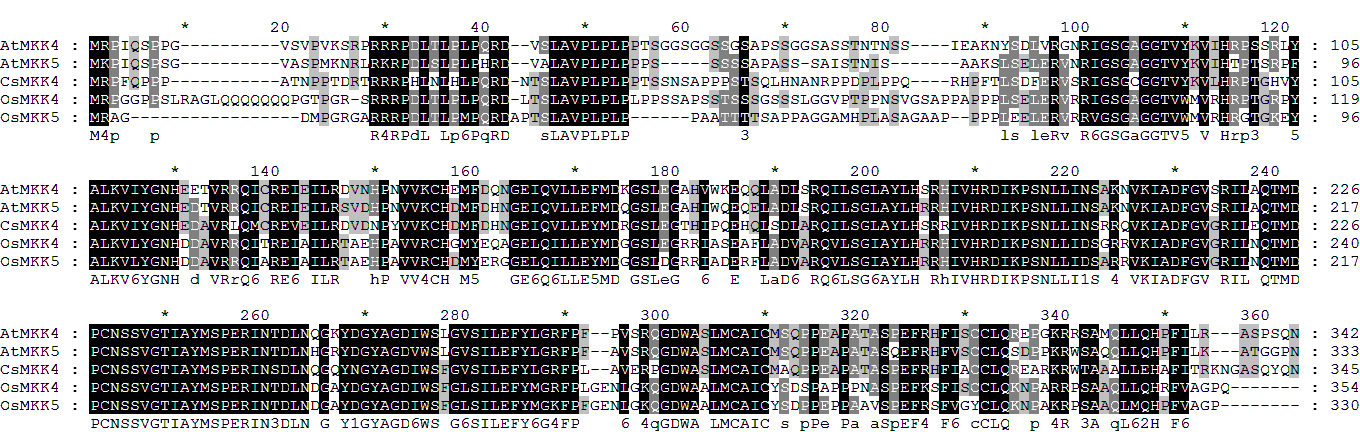


S/T-X5-S/T

Group D MAPKK

D(L/I/V)K

S/T-X5-S/T


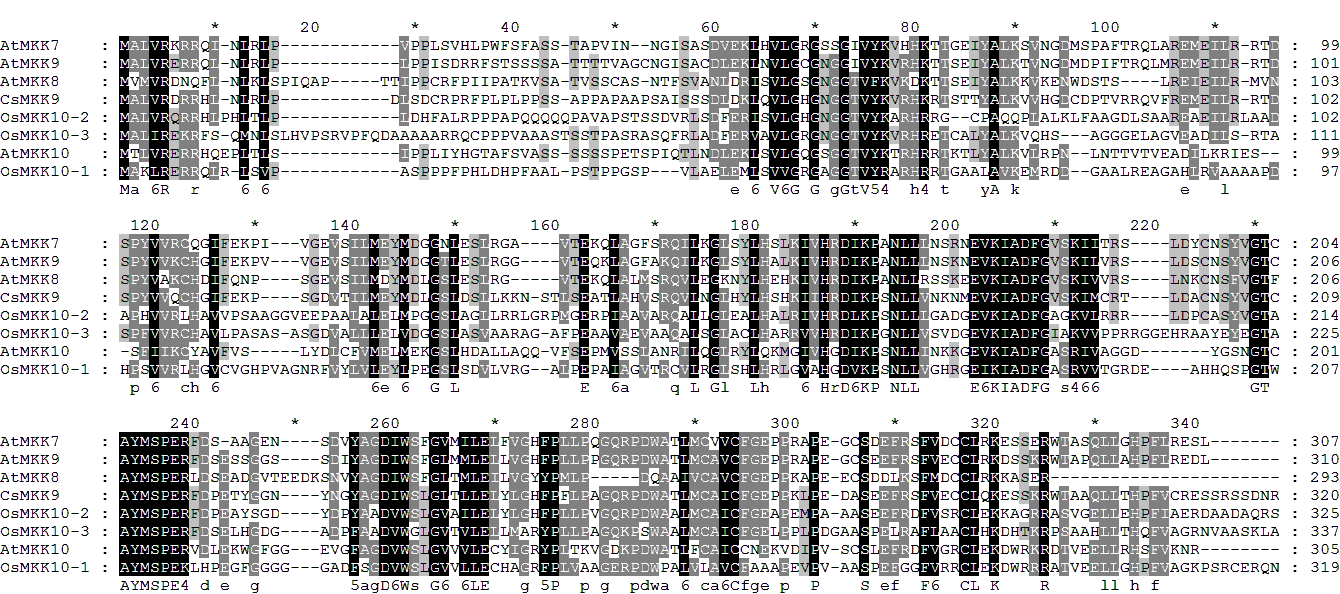


Group ZIK MAPKKK

GTPEFMAPE(L/V)Y


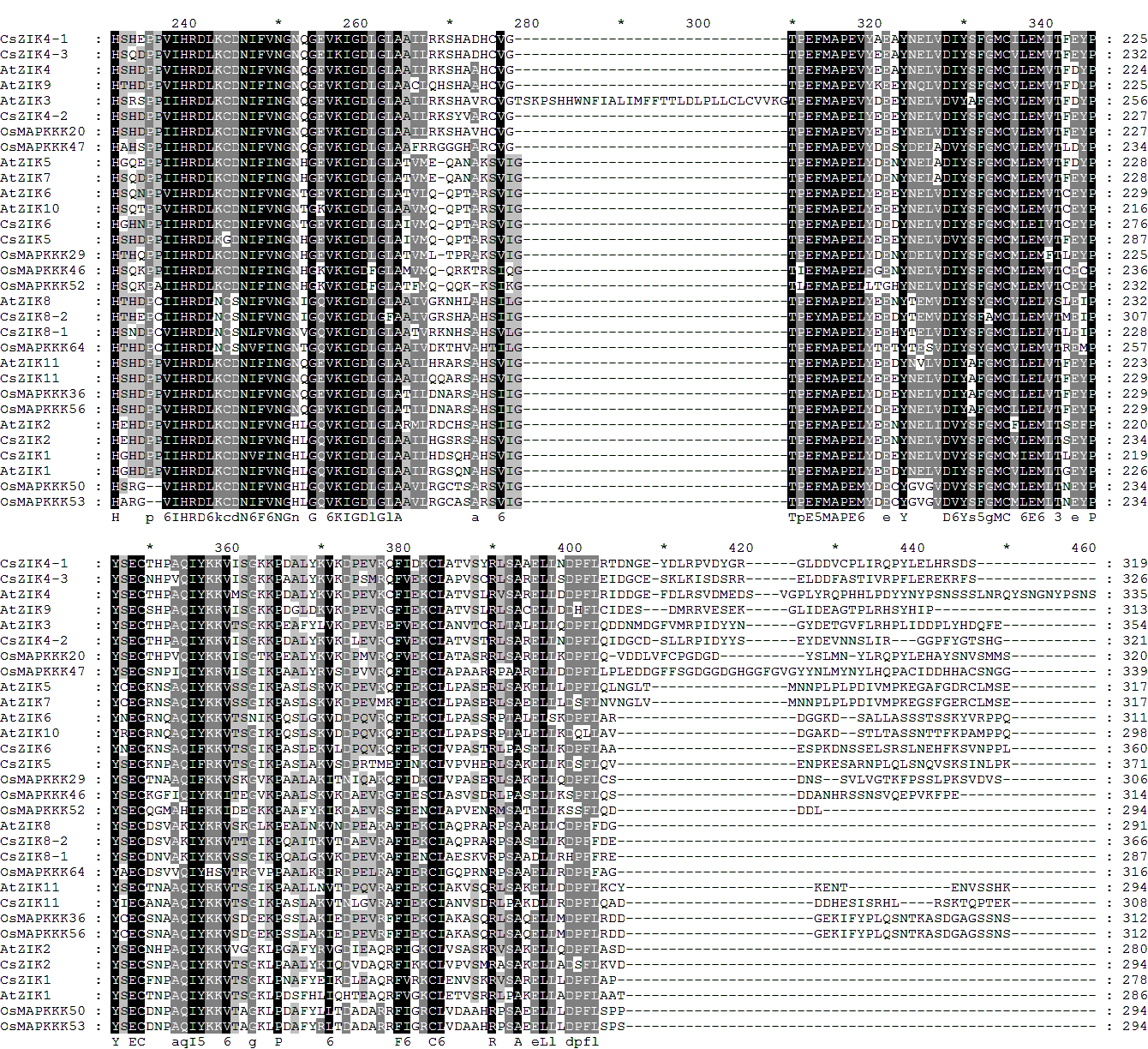


Group MEKK MAPKKK

G(T/S)Px(W/Y/F)MAPEV


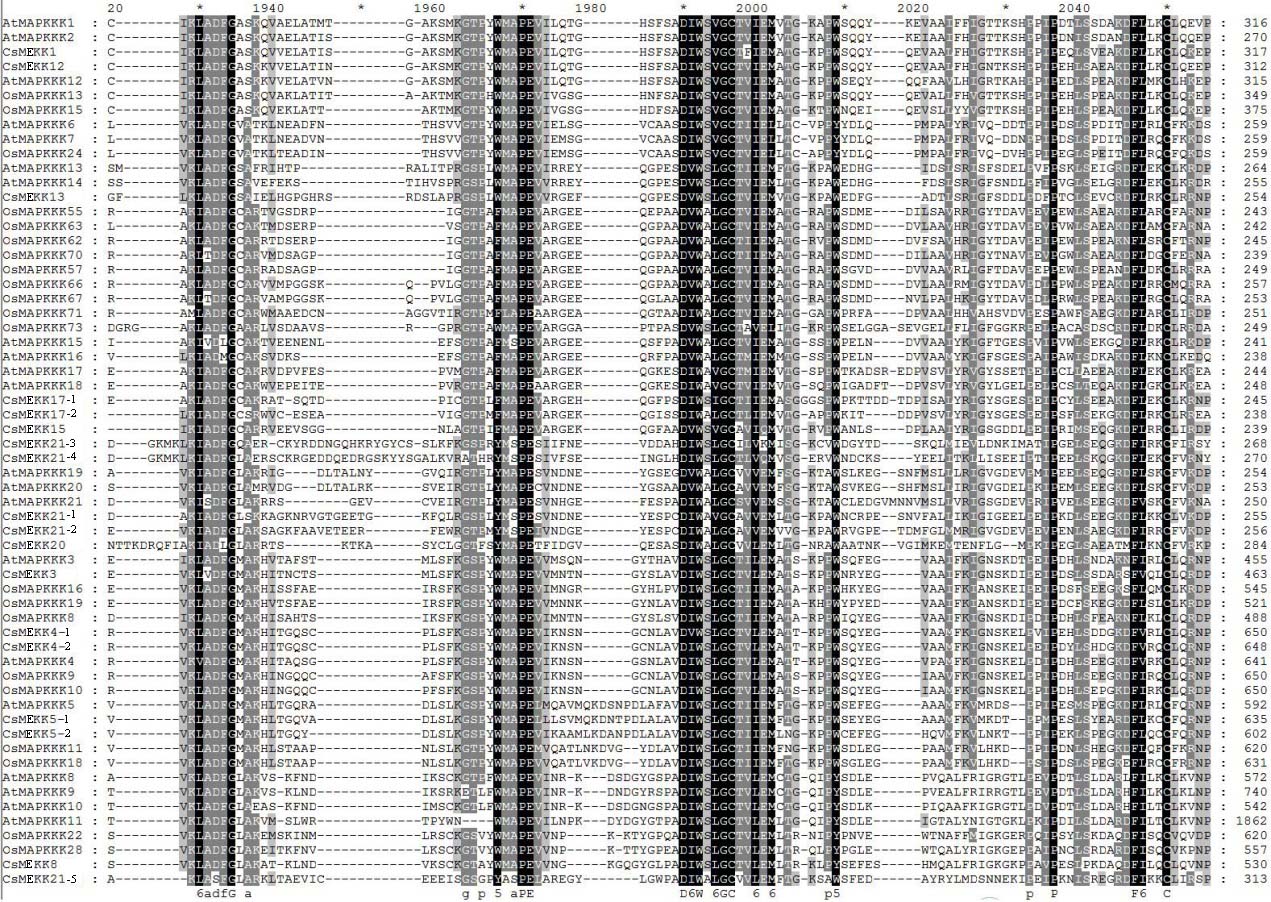


Group RAF MAPKKK

GTxx(W/Y)MAPE


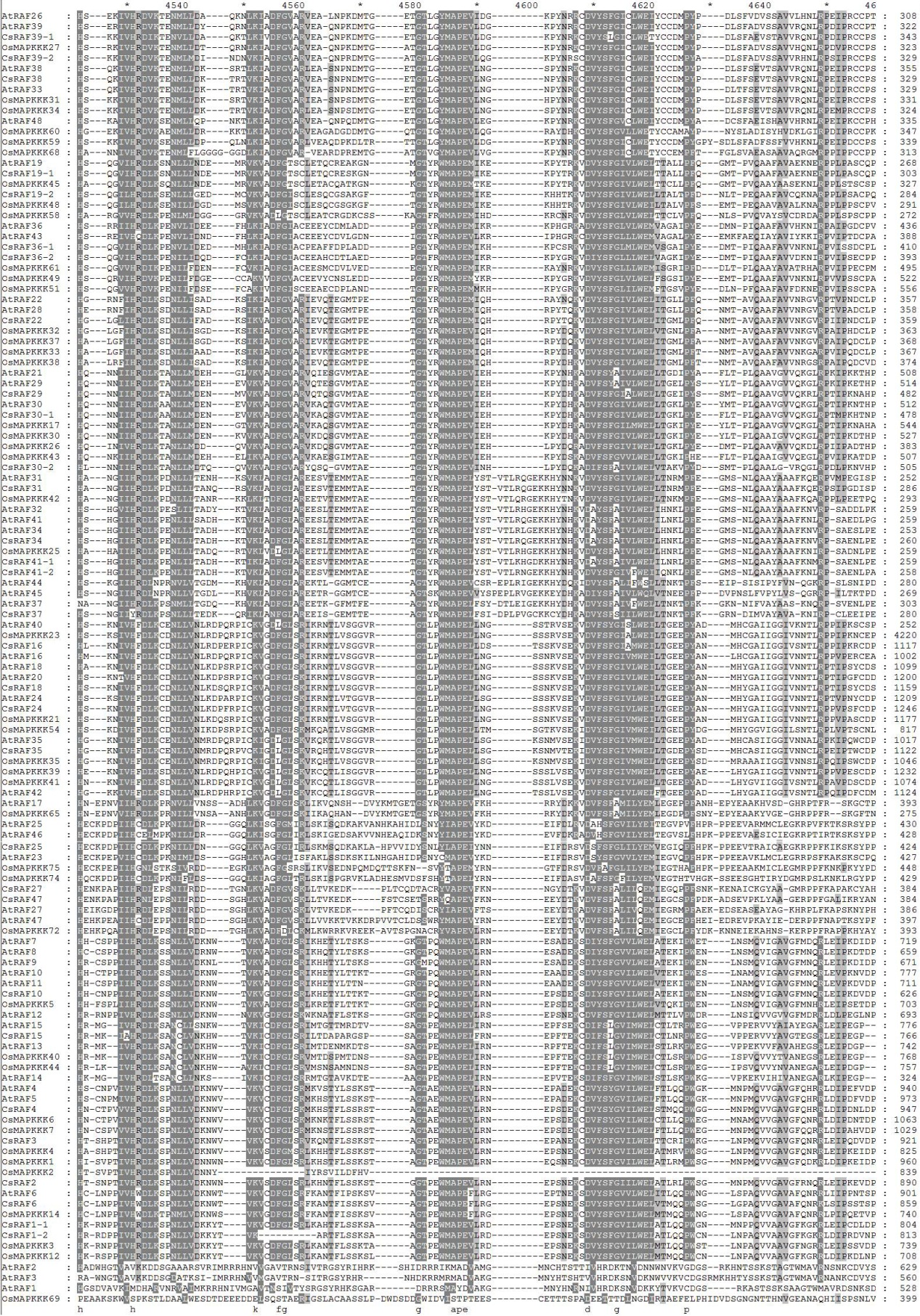


Alignment was performed using ClustalX. The conserved amino acid signature of each subgroup is highlighted in red box.
