# Supplementary figures and images for: Genome-wide identification of MAPK, MAPKK, and MAPKKK gene families and transcriptional profiling analysis during development and stress response in cucumber
Source: BMC Genomics. 2015 May 15;16(1):386. doi: 10.1186/s12864-015-1621-2 (PMC4432876; doi:10.1186/s12864-015-1621-2)

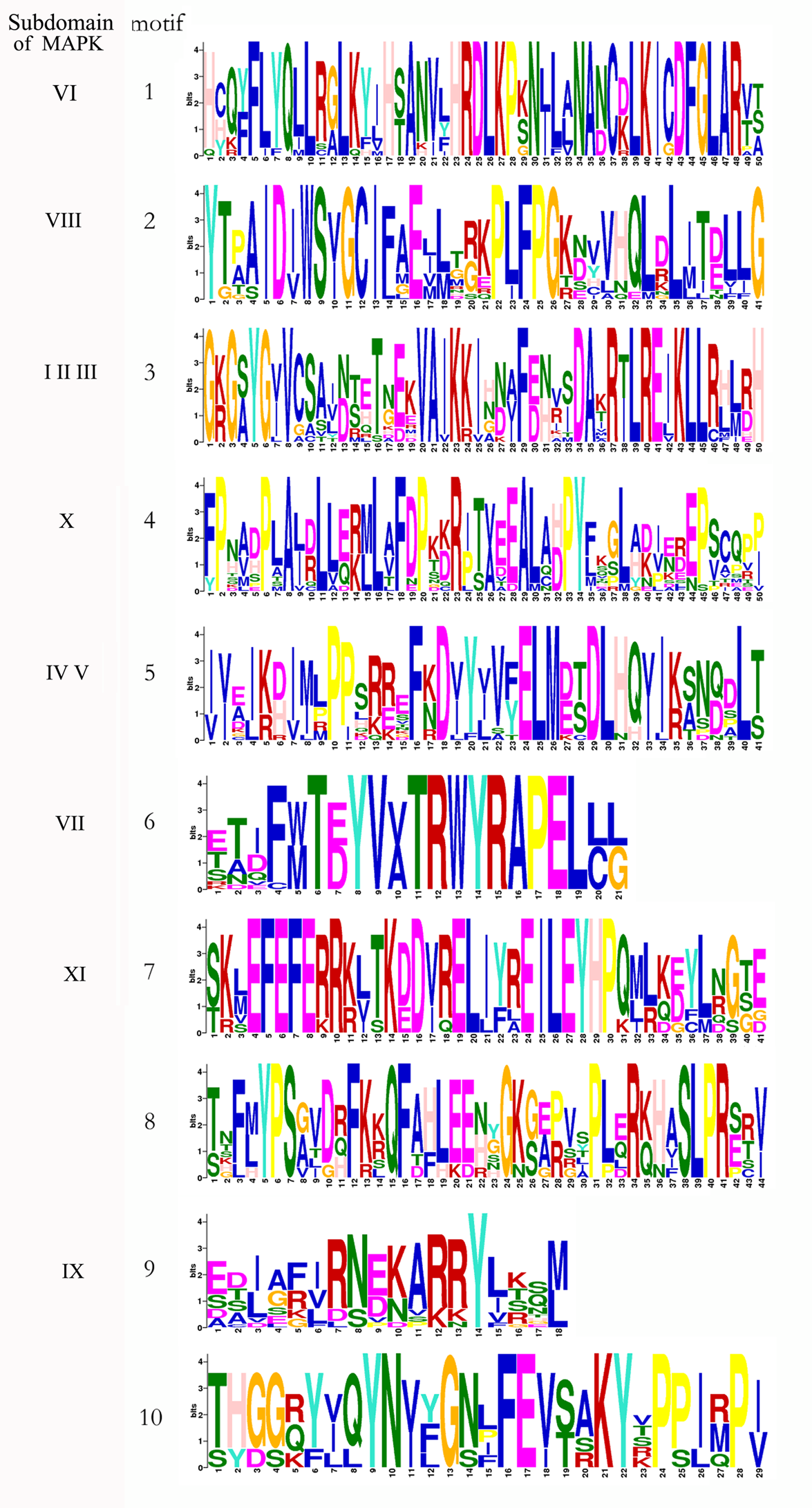

Supplement: Additional file 6: — The ten conserved motifs of MAPKs detected by the online tool MEME. [file 12864_2015_1621_MOESM6_ESM.tiff]

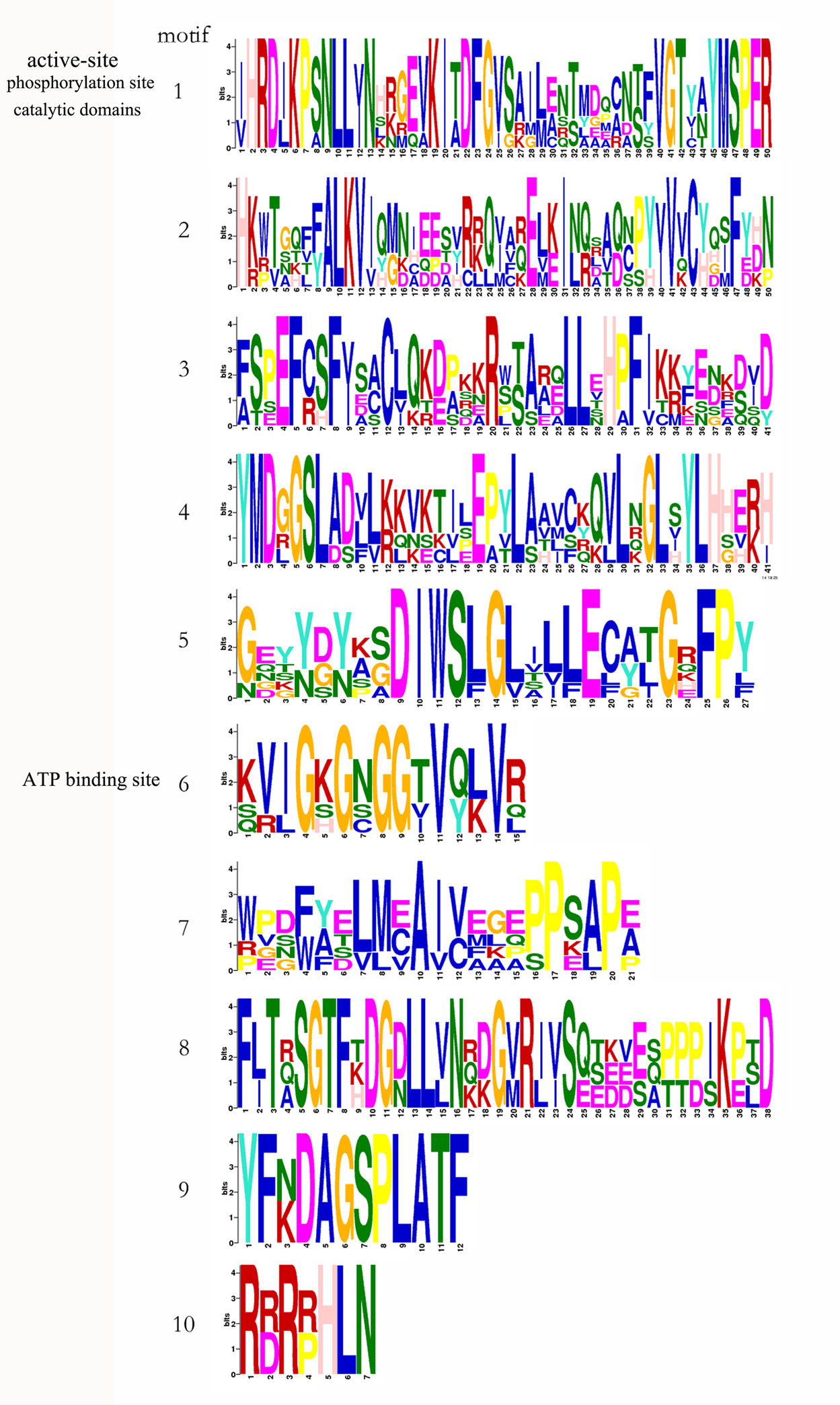

Supplement: Additional file 7: — The ten conserved motifs of MAPKKs detected by the online tool MEME. [file 12864_2015_1621_MOESM7_ESM.tiff]

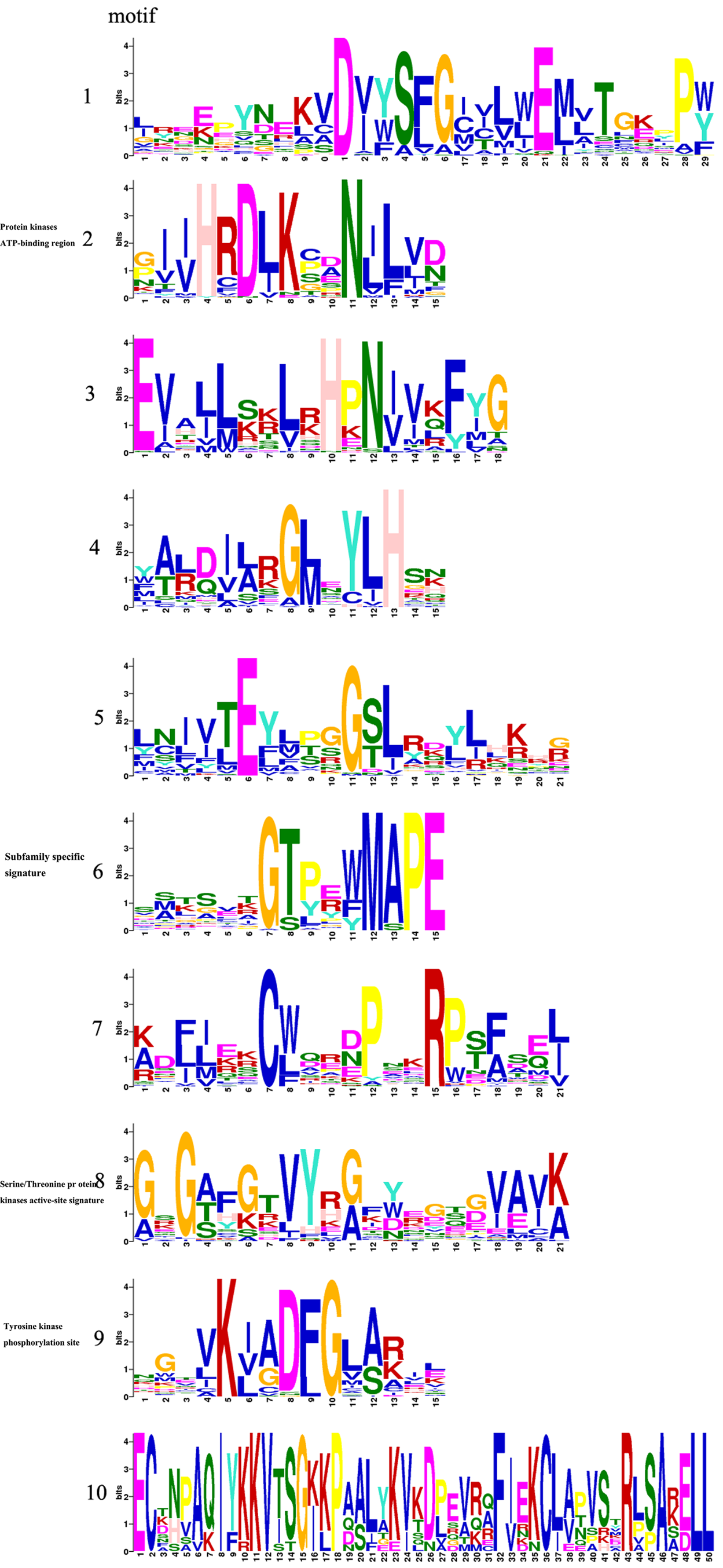

Supplement: Additional file 8: — The ten conserved motifs of MAPKKKs detected by the online tool MEME. [file 12864_2015_1621_MOESM8_ESM.tiff]

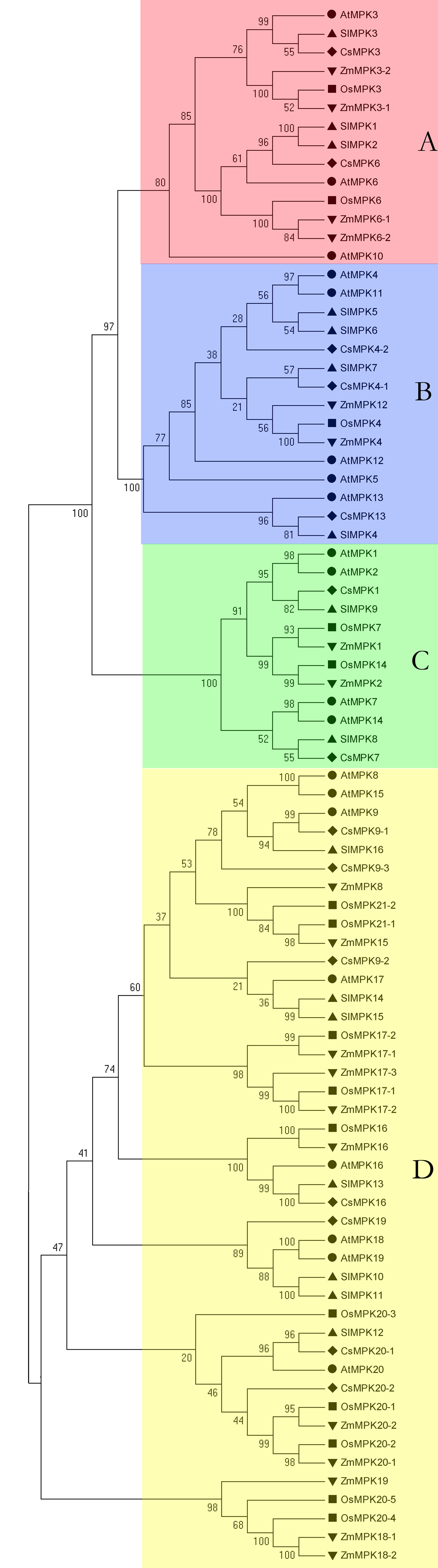

Supplement: Additional file 13: — The phylogenetic tree of MAPK genes from cucumber, Arabidopsis , tomato, rice, and maize. [file 12864_2015_1621_MOESM13_ESM.tiff]

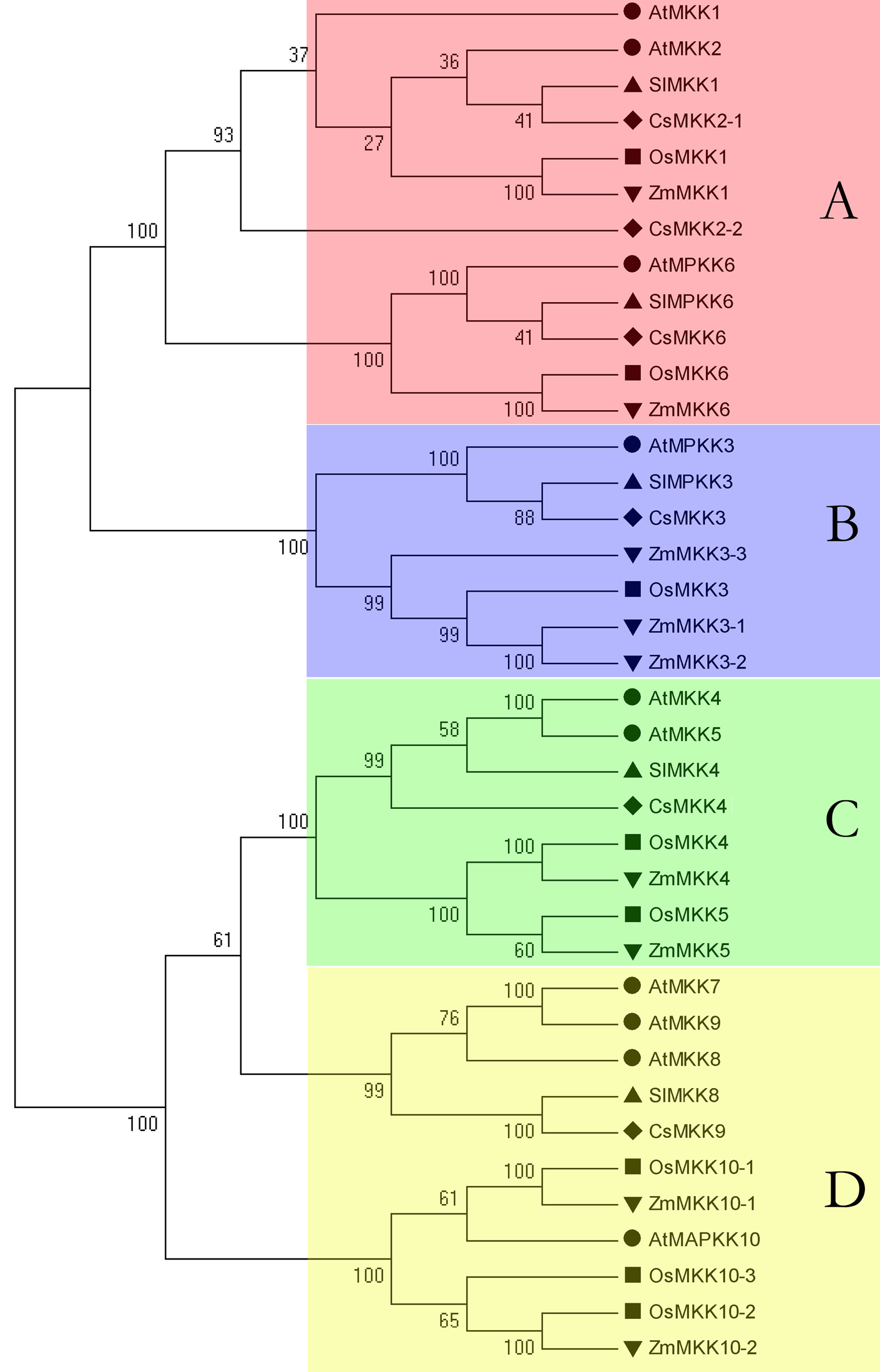

Supplement: Additional file 14: — The phylogenetic tree of MAPKK genes from cucumber, Arabidopsis , tomato, rice, and maize. [file 12864_2015_1621_MOESM14_ESM.tiff]

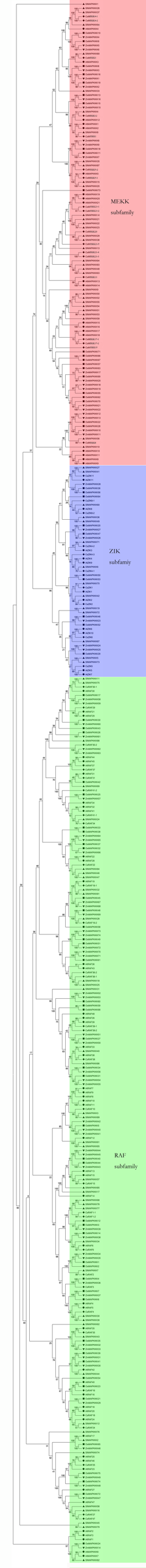

Supplement: Additional file 15: — The phylogenetic tree of MAPKKK genes from cucumber, Arabidopsis , tomato, rice, and maize. [file 12864_2015_1621_MOESM15_ESM.tiff]

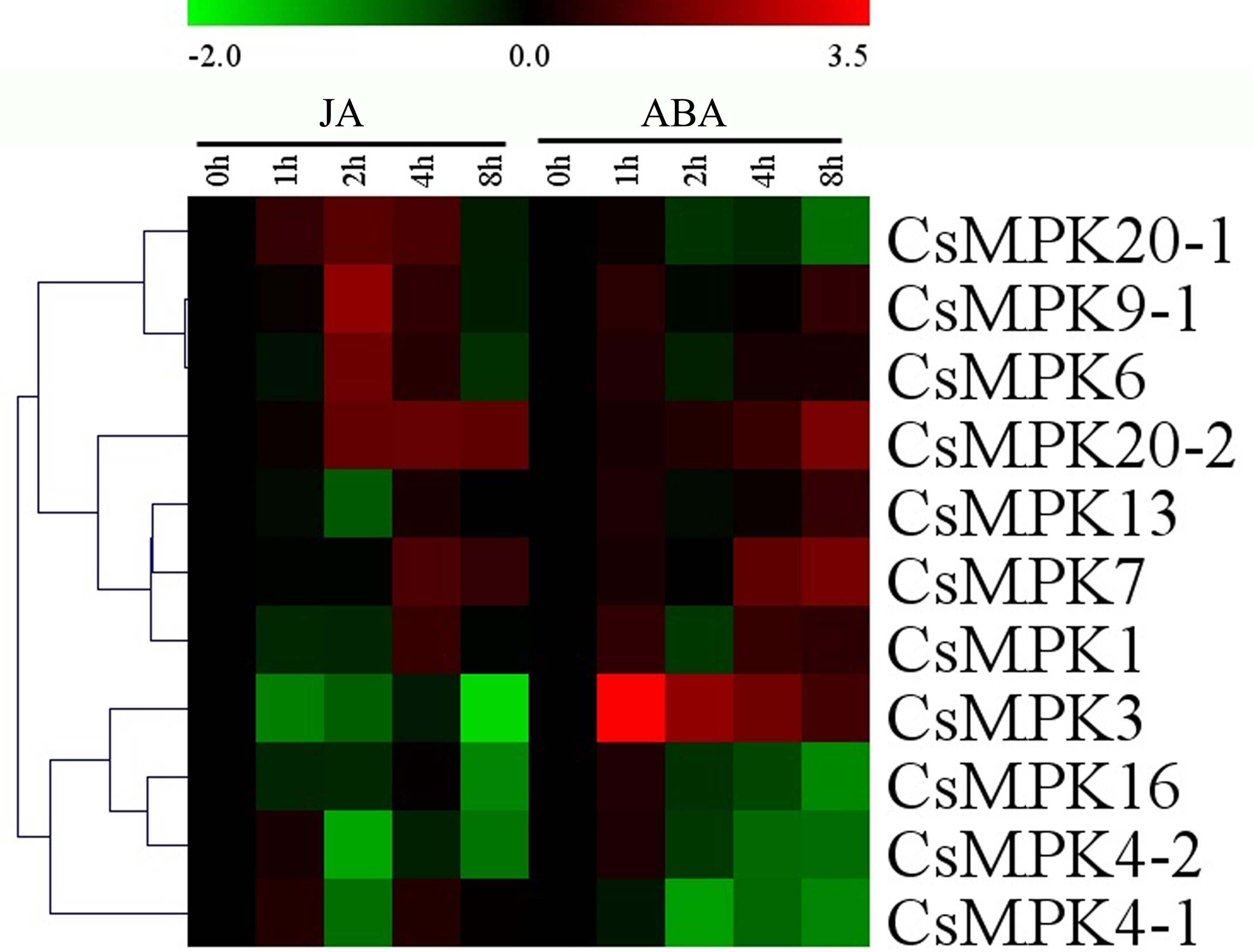

Supplement: Additional file 20: — Expression patterns of CsMAPKs with exogenous JA and ABA treatments by qRT-PCR analysis. [file 12864_2015_1621_MOESM20_ESM.tiff]

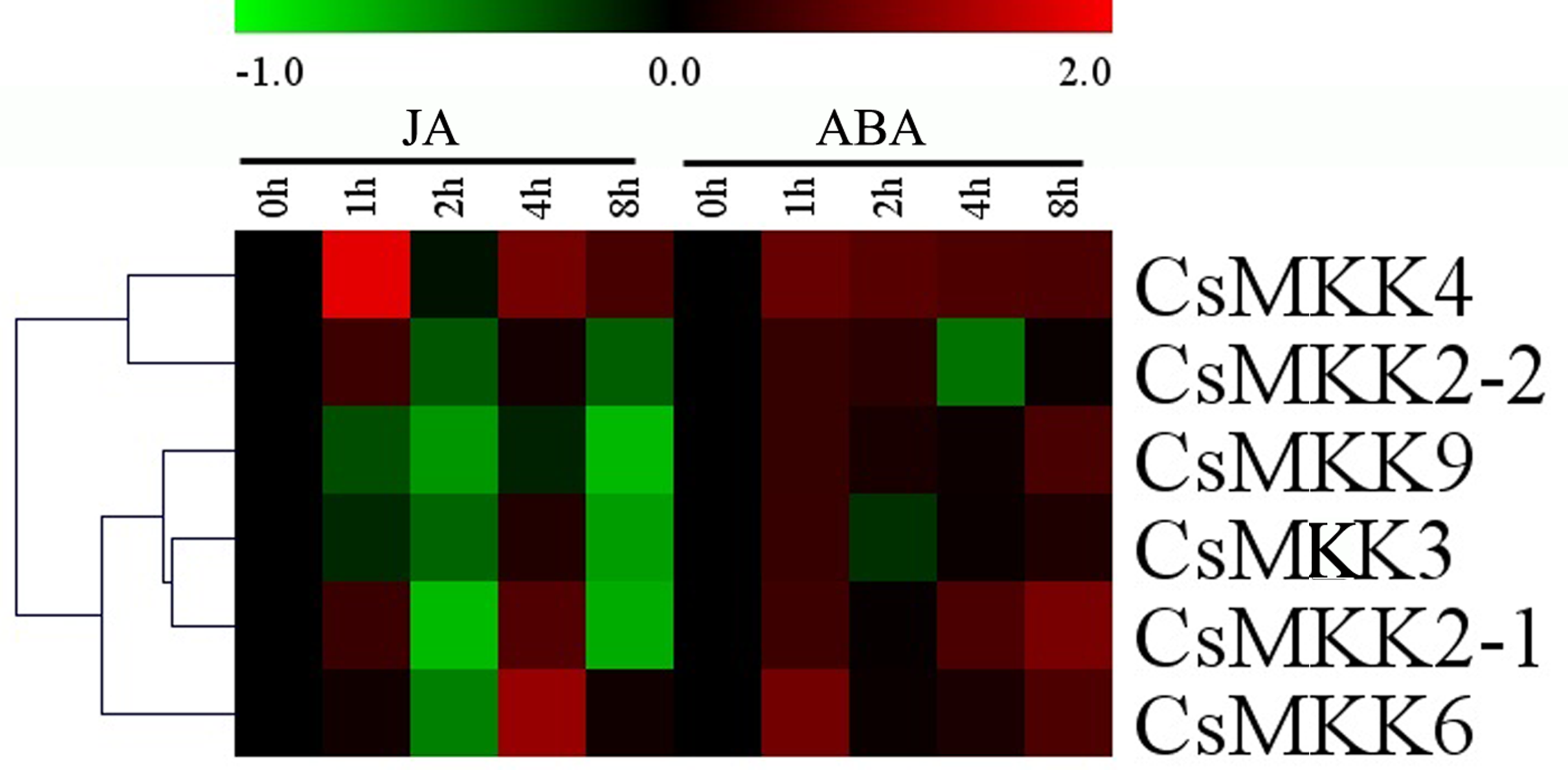

Supplement: Additional file 21: — Expression patterns of CsMAPKKs with exogenous JA and ABA treatments by qRT-PCR analysis. [file 12864_2015_1621_MOESM21_ESM.tiff]

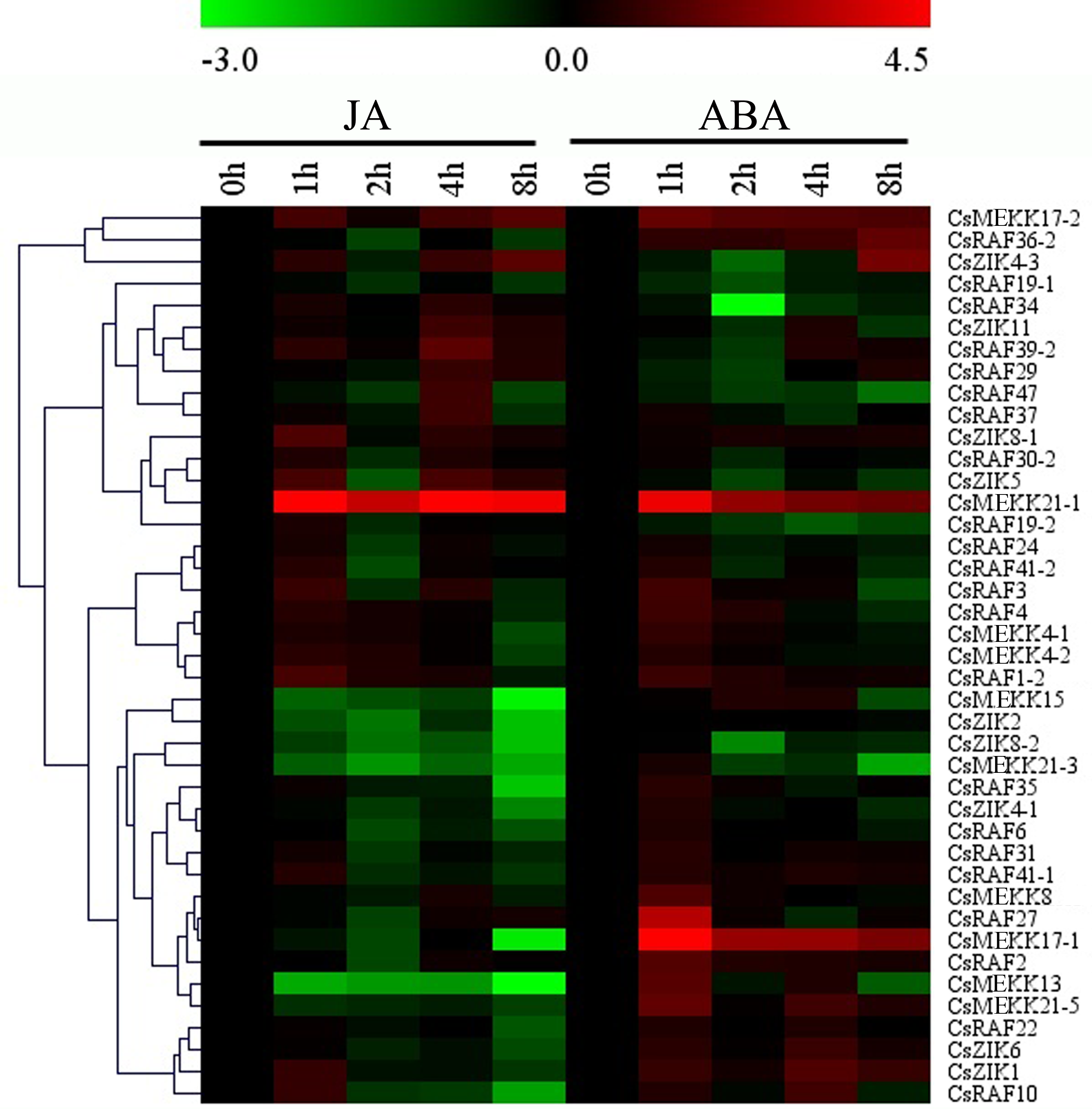

Supplement: Additional file 22: — Expression patterns of CsMAPKKKs with exogenous JA and ABA treatments by qRT-PCR analysis. [file 12864_2015_1621_MOESM22_ESM.tiff]
