# Supplementary material for: Genome-wide identification of MAPK, MAPKK, and MAPKKK gene families and transcriptional profiling analysis during development and stress response in cucumber
Source: BMC Genomics. 2015 May 15;16(1):386. doi: 10.1186/s12864-015-1621-2 (PMC4432876; doi:10.1186/s12864-015-1621-2)
Supplement: Additional file 19: — Change of expression levels of CsMAPKKKs under abiotic and biotic stress treatment in cucumber by qRT-PCR analysis in line map. [file 12864_2015_1621_MOESM19_ESM.doc]

A：cold

B:heat

C：drought

D：*Pseudoperonospora cubensis*
